# BromoCatch: a self-labelling tag platform for protein modification and live cell imaging

**DOI:** 10.1038/s41467-026-72539-w

**Published:** 2026-05-13

**Authors:** Maria Rodriguez-Rios, Conner Craigon, Mark A. Nakasone, Gajanan Sathe, Adam G. Bond, Mark Dorward, Anthony K. Edmonds, Mark C. Norley, Robert E. Arnold, Paul M. Wood, Stephen J. Reynolds, Joel O. Cresser-Brown, Graham P. Marsh, Hannah J. Maple, Alessio Ciulli

**Affiliations:** 1 Centre for Targeted Protein Degradation, School of Life Sciences, University of Dundee, Dundee, UK; 2 Bio-Techne (Tocris), The Watkins Building, Avonmouth, Bristol, UK

**Keywords:** Chemical tools, Chemical modification, Microscopy, Structure-based drug design, Protein design

## Abstract

Visualizing and manipulating proteins in live cells is crucial for studying complex biological processes. Self-labelling protein (SLP) tags such as HaloTag and SNAP-tag are widely used for protein labelling, and new systems are needed to expand multiplexing capabilities and broaden the scope of applications. Here we present BromoCatch, a small ~13 kDa bromodomain (BD)-based SLP platform, engineered with a nucleophilic cysteine for covalent ligand engagement. A structure-based designed library of electrophilic ligands was screened against two cysteine-containing mutants using differential scanning fluorimetry and intact protein mass spectrometry to assess covalent complex formation. We identified a para-acrylamide bumped derivative MR116 and the Brd4-BD2 double mutant L387A,E438C as the optimal protein-ligand pair, and reveal the binding mode through an X-ray co-crystal structure solved to 1.3 Å resolution. BromoCatch demonstrated potent and irreversible cellular target engagement in NanoBRET and residence-time assays. Its versatility was demonstrated through the design of a biotinylated conjugate, PROTAC-based degraders, and fluorescent full-on and “switch-on” probes for ex-cellulo and live-cell imaging, including side-by-side comparison and orthogonality with HaloTag. Together, these results establish BromoCatch as a robust, modular, and orthogonal SLP tool with broad potential for multiplexed labelling and targeted protein manipulation.

## Abstract (web summary)

The ability to observe and control proteins in living cells is essential for biological research and drug development. Here, the authors engineer the self-labelling protein tag BromoCatch, enabling selective covalent labelling of fused proteins with biotin, clickable, fluorogenic, and degrader probes for protein manipulation and multiplex cell imaging.

## Introduction

**Fig. 1:**
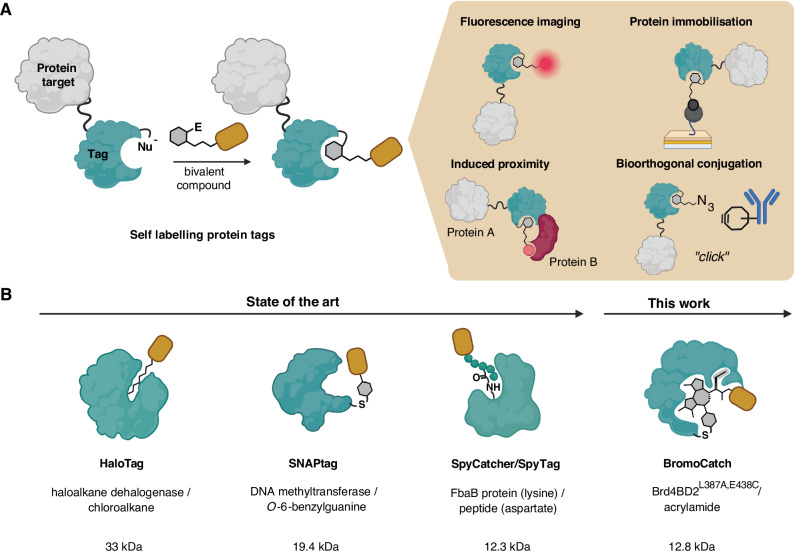
Self-labelling tag platforms for protein analysis and manipulation. **A** Principle of self-labelling tags. **B** Current self-labelling protein tags prior art and introducing BromoCatch described herein. Illustration created in BioRender. Ciulli, A. (2026) https://BioRender.com/hw0oi05.

The ability to visualize and manipulate proteins in live cells is critical for studying biology and supporting drug development. Multiple technology platforms have been developed for this purpose. Self-labelling protein tags (SLP) have emerged as powerful, versatile and technically accessible tools that have been widely adopted for both in vitro and in vivo protein research^1^. The principle behind SLPs is the combination of a protein tag, expressed as a fusion to the protein-of-interest, and a complementary small molecule ligand (‘tag ligand’) that binds (typically covalently) selectively to the protein tag (Fig. 1). The key advantage of the technology is the intrinsic versatility of SLPs due to the small molecule ligand component, which can be modified to incorporate a wide variety of handles including fluorophores, biotin, click-chemistry handles, E3 ligase ligands for targeted degradation, and orthogonal domain-binding ligands for induced proximity applications (Fig. 1A). SLPs have a broad range of applications, including fluorescent labelling for live-cell imaging, Förster resonance energy transfer (FRET) assays for studying protein–protein interactions, protein turnover and degradation studies, biotinylation, and affinity purification^2^. Their utility even extends to in vivo imaging, making them powerful and versatile tools for both basic research and translational applications^3–8^.

An important application of SLPs is in live-cell imaging experiments, enabled through pairing tag ligands with fluorophores. This approach offers several benefits over the creation of fusion fluorescent proteins such as GFP for the same application, primarily the superior photophysical properties offered by synthetic fluorophores (specifically brightness and photostability), and the ability to switch on the fluorescent signal with temporal control upon simple addition of the compound. Breakthrough advances in imaging technology and instrumentation now place greater demands on the fluorophore used and render fluorescent proteins fundamentally sub-optimal for many advanced and super-resolution microscopy techniques. Further, it is well documented that the relatively large molecular weight of GFP (27 kDa, often larger than the fusion partner protein of interest) can confound experiments by altering the interactions, localization and/or function of the protein partner^9–13^.

The most popular SLPs currently in use are HaloTag^14,15^, SNAP-tag^16^, CLIP-tag^17^ and SpyTag^18,19^ (Fig. 1B). HaloTag, developed by Promega, is engineered from the bacterial haloalkane dehalogenase (DhaA) from Rhodococcus. Through mutagenesis, Wood et al. ^14,20^ engineered DhaA (H272F) to irreversibly trap the covalent ester intermediate within the enzyme mechanism at the nucleophilic residue D106, by deactivating the second step in the mechanism through the catalytically inactive phenylalanine at position 272. Despite its relatively large size (33 kDa), HaloTag’s net negative charge at p*I* = 5 enables it to function as a solubility tag in protein purification with reduced tendency towards aggregation. SNAP-tag, developed in Kai Johnsson’s lab, is derived from human O-6-alkylguanine–DNA alkyltransferase (hAGT). Through directed evolution and random mutagenesis, Johnsson et al. developed a SNAP-tag with high specificity for O-6-benzylguanine-equipped ligands. At a size of 19.4 kDa, SNAP-tag is smaller, so less likely to cause perturbation of tagged proteins compared to HaloTag^21^. A more recent derivation of SNAP-Tag is CLIP-tag^17^. Through mutagenesis of eight amino acids in SNAP-tag, CLIP-tag was created, reacting with high speed and selectivity with O-2-benzylcytosine derivatives. The creation of CLIP-tag was motivated by the need for a tag compatible with SNAP-tag, allowing for multiplexing of two proteins in the same cell, given the limited number of SLPs available at the time^17^. SpyTag/SpyCatcher^19^ uses a short peptidic sequence (SpyTag, 13 amino acids) that covalently attaches to a larger sequence (SpyCatcher, 12.3 kDa) via isopeptide bond formation. More recently, the covalent TMP-tag^22^ has gained popularity following the optimization of its originally reversible version^23^. This system utilizes *Escherichia coli* dihydrofolate reductase (eDHFR; 18 kDa) as a fusion tag and employs the folate analogue trimethoprim (TMP) for labelling.

The HaloTag and SNAP/CLIP-tag platforms have today emerged as the gold standard and are an integral part of the chemical biologist’s toolbox. There are limitations, however, associated with both systems. The relatively large size (33 kDa) of HaloTag may perturb the interactions and function of the protein of interest^22,24,25^. Compared to HaloTag, SNAP-tag typically exhibits slower labelling kinetics and reduced fluorescence with rhodamine substrates^26^. Additionally, the benzyl guanine-based probes may need optimization to overcome the poor cell permeability of the benzyl-guanine ligand^27^, though recent improvements have begun to address some of these limitations^28^. Similarly, the SpyCatcher/SpyTag platform can be limited by slow covalent bond formation and the poor cell permeability of the SpyTag peptide, requiring conjugation to cell-penetrating peptides for intracellular targets^29^. The TMP-tag is the smallest non-peptide-based SLP, but its covalent reaction depends on NADPH, the native cofactor of DHFR, which limits its use to intracellular applications. Finally, the SLP ligands typically used are not considered ‘drug-like’ and are associated with poor pharmacokinetic properties, specifically rapid clearance, which can present challenges during in vivo application^30^. To address these limitations and expand the arsenal of SLPs, it is therefore important to innovate additional orthogonal systems that can be used as alternatives or simultaneously to the currently used tags.

In this work we expand the toolbox by developing an orthogonal SLP platform for multiple applications (Fig. 1B). Inspired by our previous “bump-and-hole” approach applied to Brd4-BD2 to generate a functionally silent, ‘hole-containing’ point mutant (Brd4-BD2^L387A^) and a highly specific and potent small molecule ligand (ET-JQ1-OMe)^31,32^ we sought to modify this system to introduce covalency, while simultaneously reducing the molecular weight of the protein tag. Our lab’s BromoTag degron system^33^, based on the “bump-and-hole” approach, enables selective targeting of a mutant Brd4-BD2^L387A^ bromodomain using a complementary “bumped” non-covalent PROTAC^33^. This platform facilitates potent, selective, and rapid degradation of tagged proteins, proving valuable in targeted protein degradation (TPD)^34–36^ and for induced proximity approaches beyond TPD^37^. Here, we describe our development of BromoCatch, a self-labelling tag platform expanding applications beyond degradation. By engineering Brd4-BD2^L387A^ to introduce a nucleophilic cysteine amino acid (Brd4-BD2^L387A,E438C^) at a suitable location and incorporating an electrophilic warhead into the “bumped” ligand, we created a covalent and highly specific SLP system. Through systematic biophysical screening of a structure-guided electrophilic ligand library, we identified MR116 as the optimal covalent ligand, validated by a 1.3 Å co-crystal structure. The resulting BromoCatch platform demonstrates potent and irreversible target engagement in cells and supports biotinylation, degradation, and fluorogenic imaging applications, establishing it as a versatile and orthogonal self-labelling tool for protein analysis and live-cell studies.

## Results

**Fig. 2:**
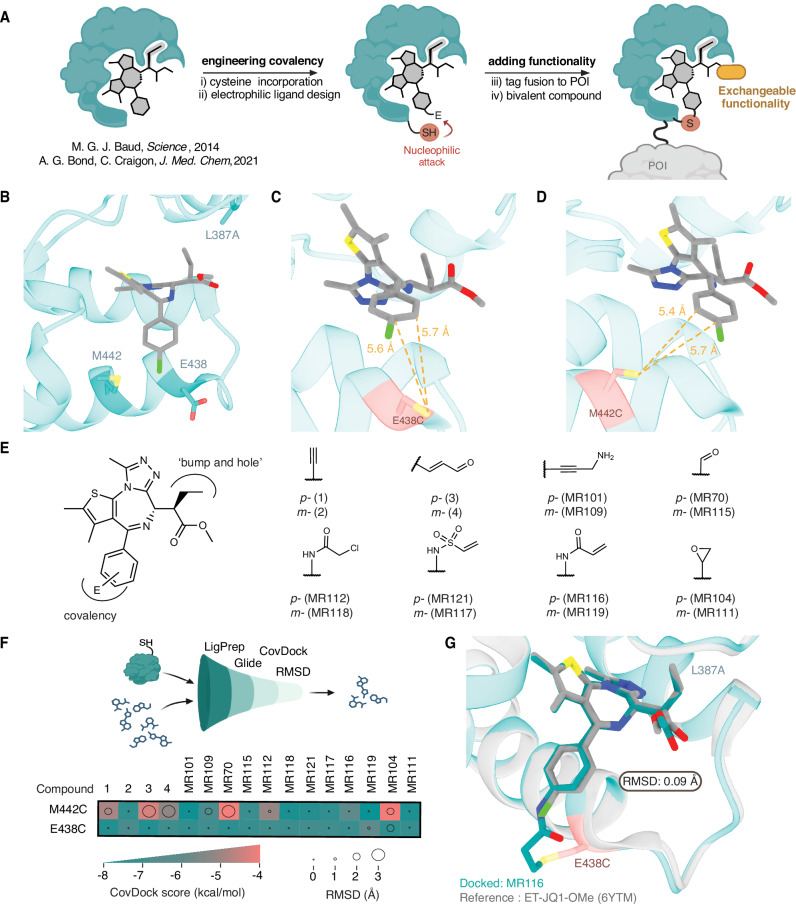
BromoCatch design, development and covalent docking. **A** Rational design of the system. **B** Brd4-BD2 M442 and E438 identified as amenable residues for electrophilic conjugation. **C** Modelled distances between the sulfur atom of the E438C mutant cysteine and the carbon atoms at the *meta* and *para* positions of the ligand phenyl ring. **D** Same modelled distances as in panel (**C**) for the M442C mutant cysteine to *meta* and *para* carbons of the ligand. **E** Library of *meta* and *para* electrophilic compounds. E = electrophilic warhead. **F** In silico evaluation workflow and heatmap summarizing covalent docking scores and RMSDcs ((root mean square deviation over common structure) for E438C and M442C mutants. **G** Covalent docking of MR116 (teal carbons) bound to Brd2-BD2^L383A,D434C^ and structural alignment with reversibly bound ET-JQ1-OMe (grey) in complex with Brd2-BD2^L383V^ in the original co-crystal structure (PDB: 6YTM). Illustrations in panels **A** and **F** created in BioRender. Ciulli, A. (2026) https://BioRender.com/8cdbft4.

The initial design of the system focused on finding residues in or around the binding pocket of the ‘hole-containing’ protein mutant Brd4-BD2^L387A^ that could be mutated to incorporate a cysteine that oriented near a suitable vector of the ET-JQ1-OMe ligand (Fig. 2A)^33,38^. To evaluate the Brd4-BD2 binding pocket, we used an existing co-crystal structure of Brd2-BD2^L383V^ bound to ET-JQ1-OMe (PDB code: 6YTM). Brd2-BD2 has exquisite structural homology and sequence identity at the ligand binding site compared to Brd4-BD2, thus offering an appropriate surrogate to structurally interrogate the Brd4-BD2 binding pocket (Supplementary Fig. 1). Residues Met442 and Glu438 in Brd4-BD2 are located within 6 Å of the pendant aryl ring in ET-JQ1-OMe and were therefore identified as attractive sites for introducing cysteine mutations. We rationalized that the distance to the pendant ring (6 Å) would be sufficient to enable nucleophilic attack of a suitable electrophilic warhead, introduced into the phenyl ring. (Fig. 2B). To test this hypothesis, two Brd4-BD2 mutants were designed, keeping the ‘hole’ (L387A) and incorporating a nucleophilic cysteine by additional mutation of E438C (Fig. 2C) or M442C (Fig. 2D). Complementarily, a series of 16 electrophilic derivatives of the ET-JQ1-OMe were designed that maintain the ethyl “bump” and incorporate an electrophilic warhead either at the *para* or *meta* positions of the pendant aryl ring (Fig. 2E).

To gain an indication of the system's viability, the designed protein mutants and the library of ligands were prepared in silico (Schordinger Suite, LigPrep) and evaluated by reversible (Glide)^39^ and covalent docking (CovDock)^40^. Starting from the ET-JQ1-OMe:Brd2-BD2^L383V^ co-crystal structure (PDB code: 6YTM), both L387A,E438C and L387A,M442C double mutant analogues were generated and screened in silico against the entire library of *meta* and *para* ligands. The results of this screen are shown in Fig. 2F, where CovDock scores and RMSD over the bound ligand atoms are monitored to predict the likelihood of a given system to form strong covalent binding while retaining the known favourable ligand-binding mode and non-covalent interactions. The most striking trend observed in the covalent docking screen is that the E438C mutation (D434C in Brd2-BD2, Supplementary Table 1) resulted in better covalent binding scores and much lower RMSD values than M442C, suggesting that the cysteine residue position/orientation in the binding pocket is important and supporting E438C as the preferred mutant. Of note, the differential predicted reactivity for the two cysteines, based on calculated pK_a_ values of E438C = 8.4 and M442C = 8.9, may contribute to the trend observed. When comparing the *meta* or *para* position for a given electrophilic warhead (e.g., MR116 vs. MR119), we did not observe major differences or trends between the two, and both analogues yielded highly favorable energy scores and very low RMSD, especially for the E438C mutant (Fig. 2F), suggesting that the electrophilic warhead can react from either position. Overall, the covalent docking screen suggests that both engineered cysteine mutants should react well with most of the designed electrophilic ligands, with the E438C predicted as preferred. As an example of the promising system design, the docked acrylamide MR116 showed exquisite overlap in binding mode when the common thienotriazolodiazepine structure was superposed with the reference non-covalent ligand (RMSD_CS_ = 0.09 Å) and a high CovDock score of −7.29 kcal/mol (Fig. 2G).

With the promising results from the in silico studies, the newly designed mutant proteins were taken forward for recombinant protein expression. To achieve our goal of minimizing tag molecular weight, we removed 17 amino acids of the N-terminus before the bromodomain (333–350) in a region that was expected to have no detrimental effect on stability while maintaining all the structural regions of Brd4-BD2 intact (Supplementary Fig. 2). The short mutants Brd4-BD2^L387A,E438C^ (351–459) and Brd4-BD2^L387A,M442C^ (351–459) were successfully expressed and purified from *E. coli* and their stability tested compared to the longer Brd4-BD2^WT^ and Brd4-BD2^L387A^ (333–459). The shortening of the sequence and introduction of the double mutation (L387A and M442C or E438C) had no apparent detrimental effect on overall stability, with the mutant showing relatively similar melting temperature (*T*_m_) values to those of the Brd4-BD2^WT^ and Brd4-BD2^L387A^ (Supplementary Fig. 2). Based on these results, both double mutants Brd4-BD2^L387A,E438C^ and Brd4-BD2^L387A,M442C^ were taken forward.

**Fig. 3:**
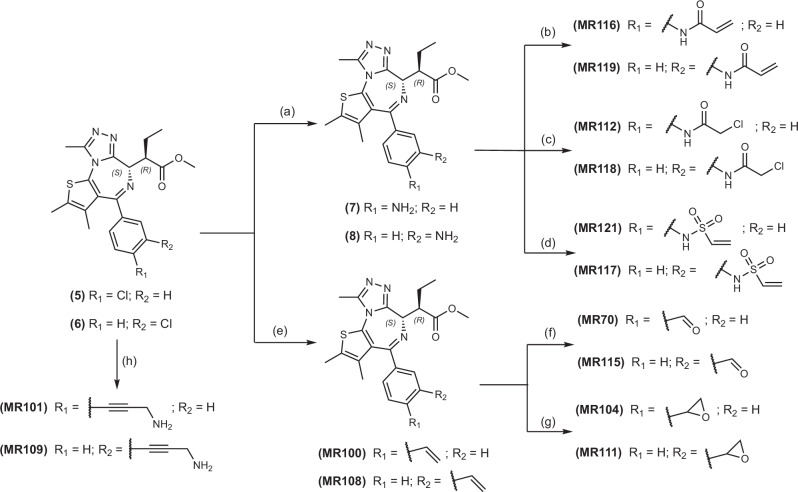
Synthesis of electrophilic “bumped” ligand library. Reagents and conditions: (**a**) (i) benzophenone imine, Pd_2_(dba)_3_, *t*-BuXPhos, K_3_PO_4_, 1,4-dioxane, 85 °C; (ii) 1 M HCl (aq.), THF, RT; (7, 50%; 8, 50%); (**b**) acryloyl chloride, DIPEA, DCM, RT; (MR116, 71%; MR119, 59%); (c) chloroacetyl chloride, DIPEA, DCM, RT; (MR112, 67%; MR118, 71%); (**d**) vinyl sulfonyl chloride, pyridine, DCM, 0 °C; (MR121, 30%; MR117, 54%); (**e**) potassium vinyltrifluoroborate, XPhos Pd G2, DIPEA, DMF/H_2_O (1:1), 90 °C; (MR100, quant.; MR108, 91%). (**f**) OsO_4_, NaIO_4_, acetone/H_2_O (5:1), RT; (MR70, quant.; MR115, quant.). (**g**) *m*CPBA, DCM, RT; (MR104, 10%; MR111, 7%). (**h**) propargyl amine, XPhos Pd G2, Cs_2_CO_3_, THF, 90 °C; (MR101, 20%; MR109, 13%).

A library of in silico-designed electrophilic ligands was synthesized, functionalising the ET-JQ1-OMe scaffold by incorporating a range of electrophilic handles. The complementary diazepine scaffolds (5) and (6) were prepared analogously to the protocols of Bond et al.^38^, with subsequent derivatization at the *para-* or *meta-*position of the pendant aryl ring, respectively (Fig. 3). Buchwald–Hartwig amination with benzophenone imine and subsequent hydrolysis produced anilines (7) and (8), which could readily be converted to the respective acrylamide (MR116 and MR119), chloroacetamide (MR112 and MR118) and vinyl sulfonamide (MR121 and MR117) ligands in high yield. In a similar fashion, a potassium vinyl trifluoroborate-promoted Suzuki–Miyaura coupling was used to access styrene intermediates (MR100) and (MR108), in turn serving as convenient precursors of epoxides (MR104) and (MR111) and offering facile access to aldehydes (MR70) and (MR115) by means of oxidative cleavage. Finally, Sonogashira cross-coupling with propargyl amine gave rise to the GNE-0011 analogues^41,42^ (MR101) and (MR109), completing the panel.

**Fig. 4:**
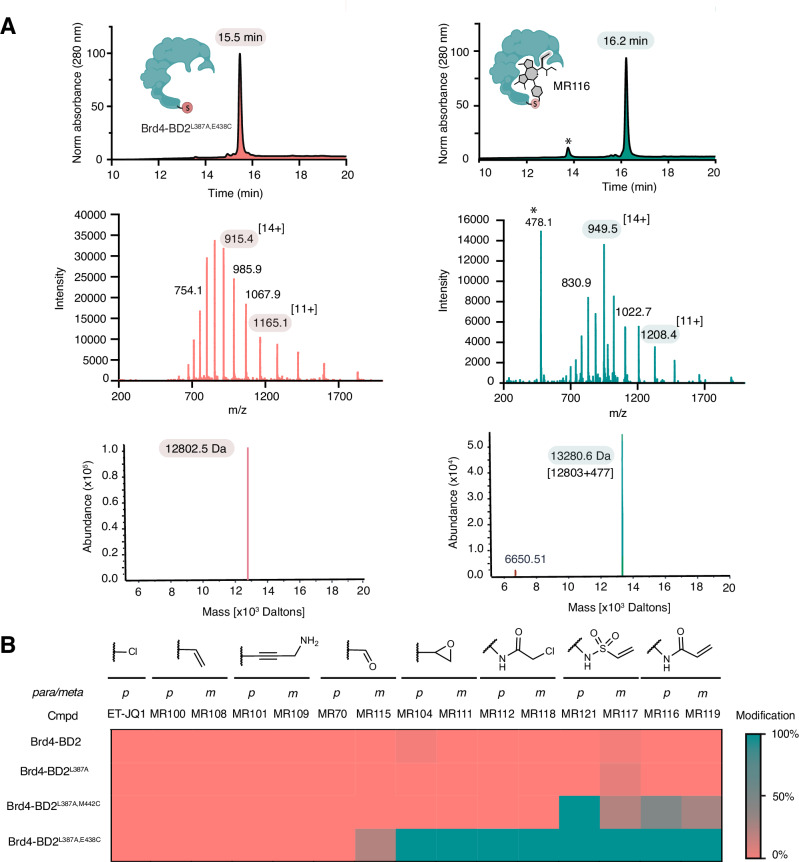
Library screen by intact protein ESI-MS to monitor covalent adduct formation. **A** Brd4-BD2^L387A,E438C^ protein was analysed by LC-MS (ESI) either in the absence (left) or presence (right) of MR116. The UV (280 nm) peak shift (top), *m*/*z* envelope change (middle) and deconvoluted mass (bottom) are shown. Asterisk indicates the mass of the MR116 ligand. Illustration created in BioRender. Ciulli, A. (2026) https://BioRender.com/cq86igq. **B** Heatmap shows the results of the systematic studies for covalent modification of all the proteins against all synthesized ligands, including controls of non-electrophilic compounds (ET-JQ1-OMe and styrene compounds MR100 and MR108).

To evaluate the reactivity of the different electrophilic ligands towards both engineered cysteine-containing mutants, covalency was systematically assessed using intact LC–MS (Fig. 4A and Supplementary Data 1). Binding resulted in a shift in LC retention time (280 nm) giving resolvable peaks that could be separately processed to obtain the corresponding mass spectrum, that could be subsequently deconvoluted to calculate the mass of the protein (Fig. 4A). Ligands that exhibited poor or no covalent binding showed no retention time shift, and no mass changes were detected or were only present in trace amounts (<5%). Several electrophiles (acrylamide, chloroacetamide, vinyl sulfonamide and epoxide) showed covalent modification of both cysteine-containing mutants Brd4-BD2^L387A,E438C^ and Brd4-BD2^L387A,M442C^ (Fig. 4B, Supplementary Table 2). Importantly, no covalent adduct formation was detected for Brd4-BD2^WT^ or Brd4-BD2^L387A^, confirming high selectivity for the engineered cysteines over other cysteine residues present within the tag protein (Cys356, Cys391 or Cys429). Brd4-BD2^L387A,E438C^ exhibited excellent reactivity towards both *meta* and *para* electrophilic ligands, likely due to the position of the engineered cysteine 438 in a relatively flexible region of the binding pocket. Conversely, cysteine 442 in Brd4-BD2^L387A,M442C^ was substantially less reactive for many of the compounds tested, possibly because this cysteine is located in a more rigid region (alpha-helix C) and has a slightly higher calculated p*K*_a_ (8.9) compared to cysteine 438 in Brd4-BD2^L387A,E438C^ (calculated p*K*_a_ = 8.4). Interestingly, *para*-substituted compounds performed better than *meta*-substituted compounds for Brd4-BD2^L387A,M442C^.

**Fig. 5:**
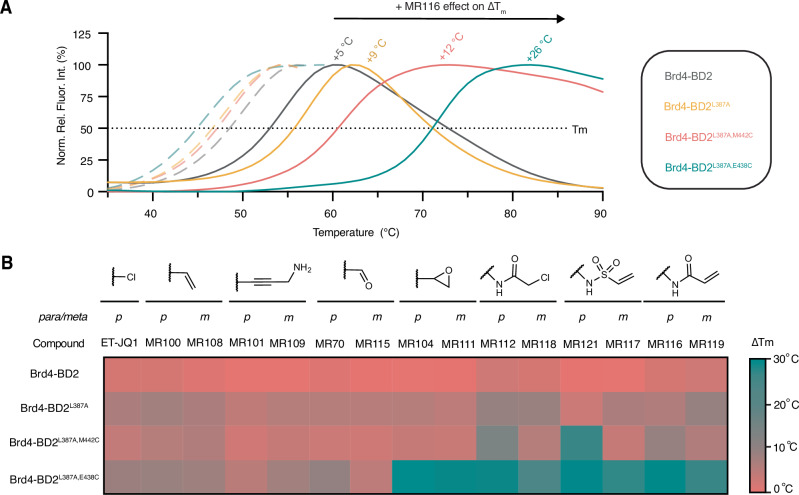
Library screen by differential scanning fluorimetry to monitor ligand-induced protein thermal stabilization. **A** DSF melting curve for the different mutant and wild-type proteins in the absence or presence of the compound MR116 (data are mean ± SD, *n* = 3 technical replicates). **B** Heatmap shows the results of the systematic DSF profiling, reporting ∆*T*_m_ (increment in melting temperature) for the different proteins against all synthesized ligands, including controls of non-electrophilic compounds (ET-JQ1 and styrene compounds MR100 and MR108).

To estimate the relative binding affinity of the component of the ligand library, a differential scanning fluorimetry (DSF) screen was performed to measure the compound-induced thermal stabilization of the different variants of the bromodomain protein (Fig. 5, Supplementary Fig. 3, Supplementary Table 3). The increment in melting temperature (∆*T*_m_) for the protein in the presence of the ligand was measured relative to that of the corresponding unliganded protein (DMSO-only control)—representative DSF traces of four protein variants with compound MR116 are shown in Fig. 5A. All tested compounds provided poor stabilization of Brd4-BD2^WT^, confirming the expected effect of the ethyl “bump” abrogating reversible binding to the wild-type bromodomain. Most of the compounds maintained the “bump-andhole” reversible binding against the Brd4-BD2^L387A^ (∆*T*_m_ ~ 10 °C), as expected. Crucially, much greater stabilization of the cysteine-bearing mutant could be observed for several ligands, with ∆*T*_m_ values measured as high as +28 °C and more prevalently with the Brd4-BD2^L387A,E438C^ mutant (Fig. 5B, Supplementary Table 3). This augmented thermal shift is consistent with the enhanced stability from the formation of covalent adducts. Of note, the pattern of extra stabilization observed on ∆*T*_m_ (Fig. 5B) correlated remarkably well with the trends in reactivity observed by LC–MS (Fig. 4B). The non-electrophilic and poorly electrophilic ligands maintained the “bump-nd-hole” non-covalent binding mode for both cysteine-containing mutants without showing any additional stabilization (∆*T*_m_ = +10 °C), confirming lack of covalent adduct formation. In a similar trend to that observed in the ESI-MS analysis, the stabilization effect for Brd4-BD2^L387A,M442C^ was less pronounced, with some of the strong electrophiles showing comparatively poor stabilization (*meta* chloroacetamide and *meta* sulfonamide). GSH stability assays revealed that the acrylamides and chloroacetamides were more stable than the vinyl sulfonamides (Supplementary Table 4). Taken together, our structure-activity screens reveal that the mutant Brd4-BD2^L387A,E438C^ in combination with the *para* chloroacetamide (MR112), vinyl sulfonamide (MR121) and acrylamide (MR116) performed as the best combinations, providing the highest percentage of covalent engagement.

Based on its high reactivity and superior stabilization with most ligands tested, we elected Brd4-BD2^L387A,E438C^ as our protein tag of choice. The acrylamide MR116 was selected as the specific complementary covalent ligand, given the optimal specific covalent modification and superior stabilization observed with Brd4-BD2^L387A,E438C^. Although the chloroacetamides MR112 and MR118 demonstrated a slight improvement in potency, we ultimately prioritized the acrylamide scaffold because of its milder intrinsic reactivity. This choice was intended to minimise off-target covalent interactions and to enhance stability *in cellulo*, thereby providing a more selective and robust chemical probe. Indeed, acrylamides have excellent stability *in cellulo*, low cross-reactivities and are widely used in chemical probes^43,44^, with multiple compounds in clinical development and FDA-approved drugs containing acrylamides as covalent warheads^45,46^. We named the system ‘BromoCatch’, with the Brd4-BD2^L387A,E438C^ as our BromoCatch fusion protein.

**Fig. 6:**
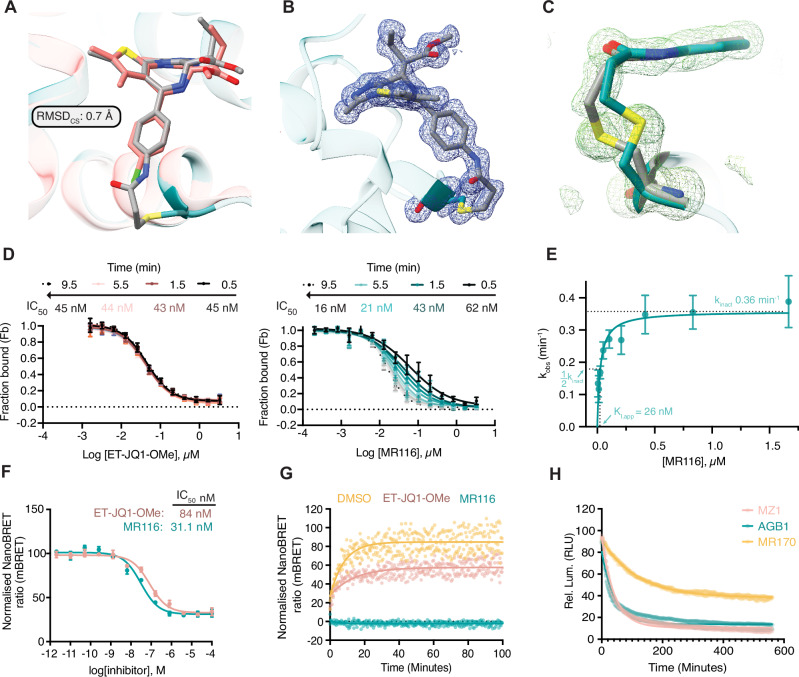
Covalent BromoCatch-ligand adduct formation characterized in vitro and in cells. **A** Overlay of the Brd2-BD2^L383V^/ET-JQ1-OMe co-crystal structure (PDB: 6YTM) and the Brd2-BD2^L383A,D434C^/MR116 (PDB 9QRK) co-crystal structure here solved to 1.3 Å of resolution (RMSDcs 0.7 Å). **B** 2Fo-Fc electron density contoured to 1σ around the MR116 ligand and the bonded cysteine (rotamer 1). **C** The polder map (Fo–Fc) contoured to 3σ around the covalent bond reveals electron density that supports two possible conformations. **D**) Time-dependency of binding affinities is observed for covalent ligand MR116 but not for the non-covalent ligand ET-JQ1-OMe in a competition-based FP assay with incubation times run up to 10 min (data shown as mean ± SD, *n*  =  3 technical replicates). **E** Plot of *k*_obs_ over ligand concentration and resulting *k*_inact_ and *K*_I,app_ values obtained from the data fit (data are mean ± SD, *n* = 3 technical replicates). **F** Quantitative analysis of MR116 ligand binding to NanoLuc-tagged Brd4-BD2^L387A,E438C^ expressed in HEK293 cells. Binding of 1 µM ET-JQ1-PEG_3_-BODIPY to NanoLuc-tagged Brd4-BD2^L387A,E438C^ expressed in HEK293 cells, displaced upon increasing concentrations of MR116 or ET-JQ1-OMe. IC_50_ values calculated as mean ± SEM from three independent repeats using “log(inhibitor) vs. response (three parameters)” using Graphpad Prism Version 10.2.3. mBRET: milli-BRET units of energy transfer. **G** Real-time kinetic analyses of ET-JQ1-BDP binding to NanoLuc-tagged Brd4-BD2^L387A,E438C^ expressed in HEK293 cells and pre-incubated for 2 h with either DMSO or 250 nM ET-JQ1-OMe or MR116. Data are presented as individual replicates alongside the mean (*N* = 2). *X*-axis shown to 100 min of the ~119 min monitored in the experiment. Data are plotted and fitted using Graphpad Prism Version 10.2.3. **H** Live-cell HiBiT kinetic degradation assay. HiBiT-BromoCatch-Brd4 knock-in HEK293 cells were transiently transfected with the pCMV-LgBiT construct prior to treatment with either MZ1 (1 μM), AGB1 (1 μM) or MR170 (2.5 μM) and the luminescent signal was continuously monitored for 9 h. Data are presented as individual replicates alongside the mean (*N* = 2). Source data are provided as a Source Data file. RLU relative light units.

To confirm covalency at a molecular level and establish the expected binding mode, the BromoCatch protein was co-crystallized in the presence of the acrylamide MR116. Since no crystals were obtained for the Brd4-BD2^L387A,E438C^: MR116 complex despite multiple co-crystallization attempts, we decided to switch to the highly homologous Brd2-BD2^L383A,D434C^, because Brd2-BD2 had previously been successful in our hands in yielding co-crystal structures with “bump-and-hole” ligands^31,32,38,47^. The screens with the Brd2-BD2^L383A,D434C^: MR116 complex resulted in high-quality crystals within hours. The Brd2-BD2^L383A,D434C^/MR116 co-crystal showed exquisite overlap with the reference crystal structure (Brd2-BD2^L383V^/ET-JQ1-OMe, PDB code: 6YTM) with an RMSD value for the common structure of the thienotriazolodiazepine below 1 Å (RMSDcs) (Fig. 6A), suggesting that the binding mode of the thienotriazolodiazepine scaffold is maintained. Inspection of the electron density around the ligand clearly evidences a covalent bond between the ligand and the engineering cysteine (Fig. 6B), as continuous electron density (1σ 2Fo–Fc) at an atomic level is observed, consistent with the formation of a thioether bond. Closer inspection of the omit difference map (polder map) suggests two alternate conformations are possible for the thioether bond of the adduct (Fig. 6C and Supplementary Fig. 4).

MR116 exhibited rapid covalent binding kinetics, as evidenced by complete conversion to the covalent adduct that was observed by intact MS upon as short as 5 min incubation time at room temperature (Supplementary Fig. 5 and Supplementary Data 1). To further evaluate the kinetics of covalent engagement of MR116 with the BromoCatch protein in vitro, we adapted a competition-based fluorescence polarization assay previously described by us^48^, using ET-JQ1-PEG₃-sCy5 as a reversible tracer. We compared the binding kinetics of reversible ligand ET-JQ1-OMe and covalent ligand MR116. Time-dependent monitoring of the IC₅₀ revealed two distinct binding behaviours: ET-JQ1-OMe maintained a constant IC₅₀ over 12 min, consistent with rapid equilibrium and reversible binding (Fig. 6D). In contrast, MR116 exhibited a pronounced decrease in IC₅₀ within the first 5 min, indicative of covalent inhibition. The anisotropy decay curves were fitted to a one-phase exponential decay model to derive *k*_obs_ values across the different MR116 concentrations (Supplementary Fig. 6). Plotting *k*_obs_ against MR116 concentration allows to obtain the values of *k*_inact_ and *K*_I,app_ to calculate the second order rate constant *k*_inact_/*K*_I_ of 3.9 × 10⁵ M⁻¹ s⁻¹ (see the “Methods” section, Supplementary Fig. 6 and Source Data) reflecting efficient covalent binding (Fig. 6E). In the context of established self-labelling tags, the BromoCatch ligand exhibits labelling kinetics rates in the range of 10⁵ M^−^¹ s^−^¹, comparable to SNAP-tag probes such as BG-TMR, while HaloTag7 can reach ~10⁷ M^−^¹ s^−^¹ with optimized probes^26^. The apparent *K*_i_ for MR116 was estimated to be 6.8 nM, as calculated from the Y₀ intercepts assuming reversible pre-equilibrium at the time of the first read. Importantly, the estimated *K*_i_ for MR116 was comparable to the *K*_i_ measured for ET-JQ1-OMe (4.9 nM)^40^ suggesting that MR116 retains high-affinity reversible binding prior to covalent bond formation (Supplementary Fig. 6).

Having established and validated the binding mode for the BromoCatch mutant protein, we next moved to assess intracellular target engagement of the BromoCatch tag across our 10 most effective covalent compounds using nanoBRET (Supplementary Fig. 7 and Supplementary Table 5). A Nanoluciferase-BromoCatch (NanoLuc) construct was generated and subsequently transfected into HEK293 cells. We synthesized a reversible tracer molecule, ET-JQ1-PEG_3_-Bodipy (560/590 nm), which binds to the BromoCatch binding site reversibly and can be competitively displaced by BromoCatch covalent ligands, resulting in a measurable loss of BRET signal. HEK293 cells expressing NanoLuc-BromoCatch were pre-incubated with ET-JQ1-PEG_3_-BODIPY (1 µM) for 5 minutes before adding the electrophilic compounds. After a 10-min incubation, the BRET signal was recorded (Fig. 6F). Among the tested ligands, MR116 exhibited optimal intracellular potency, with a pIC₅₀ of 7.5 ± 0.12 (IC_50_ = 31.1 nM), comparable to that of ET-JQ1-OMe pIC₅₀ of 7.1 ± 0.08 (IC₅₀ of 84 nM) (Fig. 6F, Supplementary Fig. 7 and Supplementary Table 5). Given the short incubation time, this result suggests rapid cellular uptake of MR116, reflecting good cell permeability of the BromoCatch ligand. To confirm covalent adduct formation within the cell, we next performed a nanoBRET-based residence time experiment. HEK293 cells expressing NanoLuc-BromoCatch were first equilibrated with a saturating concentration of ET-JQ1-OMe (250 nM), MR116 (250 nM) or DMSO for 2 h. Additional controls were performed, including BRET_max_, BRET_null_ and no tracer conditions. Following this, the ligand was removed, cells were washed and ET-JQ1-PEG3-Bodipy tracer (2.5 µM) was added. An increase in BRET signal would indicate tracer binding to NanoLuc-BromoCatch, occurring only after ligand dissociation, thereby providing a residence time measurement. This was indeed observed for the DMSO and ET-JQ1-OMe pre-incubations. In contrast, no increase in BRET signal was observed for the MR116 condition, over a 100 min time-course (Fig. 6G), indicating covalent engagement of MR116 with the BromoCatch tag in live cells.

With the best protein–ligand pair identified, we moved to derivatise the covalent binder MR116 into bifunctional compounds to incorporate additional functionalities. The methyl ester in the JQ1 scaffold represents a suitable and widely precedented exit vector to append a diverse range of functional handles^33,49,50^. Since BromoCatch is directly derived from BromoTag, which allows rapid and selective degradation of tagged protein upon treatment with potent degrader AGB1^33^, we were curious to assess the compatibility of the BromoCatch tag to also be recruited for degradation. To this end, a HiBiT–BromoCatch–Brd4 HEK293 cell line was generated by first performing site-directed mutagenesis on a pMA-RQ vector harbouring a donor sequence encoding eGFP–IRES–HiBiT–BromoTag–Brd4 to introduce the E438C mutation into the construct (Supplementary Fig. 8). The resulting construct was co-transfected with two Brd4-targeting gRNA-containing pX459 vectors into HEK293 cells, followed by fluorescence-activated cell sorting (FACS) to enrich for eGFP-positive cells (Supplementary Fig. 9). Successful generation of a heterozygous HiBiT–BromoCatch–Brd4 knock-in in HEK293 cells was subsequently confirmed by detection of luminescences using a HiBiT lytic assay and by immunoblotting (Supplementary Fig. 10). Following this, we subsequently performed a live-cell HiBiT kinetic degradation assay in which the AGB1 PROTAC degraded >85% of BromoCatch-tagged Brd4 at 1 µM over 9 h, confirming its suitability as a degrader in our new BromoCatch system (Fig. 6H, Supplementary Table 6). To assess the impact of covalent target engagement on the tag degradation performance, we synthesized the covalent PROTAC MR170 as an AGB1 analogue where the MR116 warhead is conjugated to our VHL ligand via a PEG_3_ linker (Supplementary Fig. 10, Supplementary Table 6). Compared to AGB1, MR170 exhibited slower degradation and achieved lower *D*_max_ ~ 60% at 2.5 µM over 9 h. Together, our findings demonstrate that the reversible AGB1 PROTAC, despite being originally designed for BromoTag, retains efficacy at degrading BromoCatch, suggesting the newly introduced cysteine does not disrupt the PROTAC mode of action. This means the AGB1 degrader could be readily deployed to rapidly deplete any pool of BromoCatch-tagged protein that remains unbound or unreacted in a self-labelling experiment. BromoCatch could also be degraded with covalent PROTAC MR170, although much less effectively than by AGB1. This result is consistent with the known disadvantage of PROTACs that are covalent at their target protein, such as HaloPROTACs^51–53^, due to their loss of the catalytic, substoichiometric mode of action^54^.

We assessed a common application of SLPs: click-chemistry-based conjugation. We synthesized an alkyne bearing BromoCatch probe (MR155) that can be ‘clicked’ to an azide-bearing molecule using copper-catalysed azide-alkyne cycloaddition (CuAAC). Upon incubation of MR155 with BromoCatch or Brd4-BD2^WT^ followed by click-reaction with the sulfonated Cy5 azide, we confirmed that BromoCatch but not wild-type (lacking the nucleophilic cysteine) could be fluorescently labelled covalently in vitro (Supplementary Fig. 11).

**Fig. 7:**
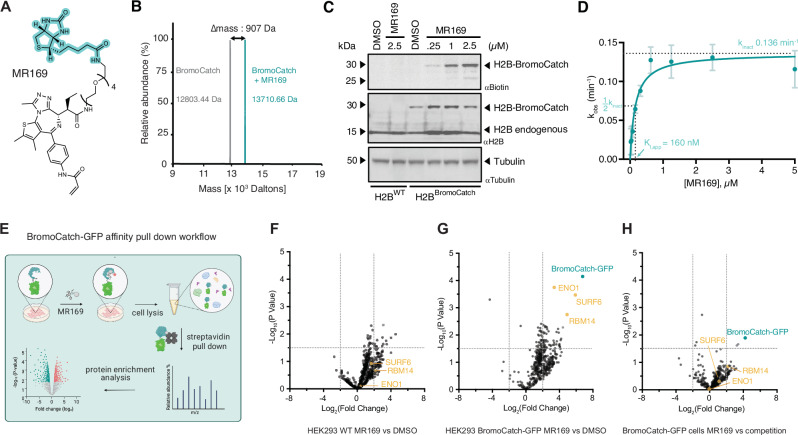
Biotin probe for cellular affinity-based pull-down applications. **A** Chemical structure of biotinylated probe MR169 (MW 908.1 g/mol). Cyan shadowing highlights the biotin moiety. **B** Formation of covalent adduct BromoCatch:MR169 evidenced by intact protein MS. **C** Cell lysate experiments show that MR169 specifically detects H2B-BromoCatch at 0.25 µM concentration of probe. **D** *k*_obs_ plotted against the concentration of MR169 and resulting *k*_inact_ and *K*_I,app_ values obtained from the data fit, allowing to calculate the second order constant *k*_inact_/*K*_I_ (data are mean ± SD, *n* = 3 technical replicates). **E** Biotin affinity pull-down mass spectrometry analysis in a transiently transfected BromoCatch-GFP HEK293 line. Illustration created in BioRender.com, Ciulli, A. (2026) https://BioRender.com/d4r5l4c. Parental or BromoCatch-GFP expressing HEK293 cells were treated with MR169 (1 µM), and probe-interacting proteins were pulled down using streptavidin-coated beads and analysed by mass spectrometry protein enrichment to demonstrate (**F**) specificity of MR169, (**G**) ability to pull down the BromoCatch-GFP construct, and (**H**) specific blockade by ET-JQ1-OMe pre-treatment. Statistical analysis and volcano plot visualization were performed using Perseus. Quantitative values were log2-transformed and compared between conditions using a two-sample Student’s *t*-test, with four biological replicates per condition. For volcano plot representation, the log2-fold change between the mean intensities of the two groups was plotted against the –log_10_ transformed *p*-value.

We next explored the applicability of BromoCatch for biotin-based protein analysis^20,55,56^. To this end, we developed a biotin-functionalised bivalently probe (MR169, Fig. 7A). The biotin probe maintained the high stabilizing effect for BromoCatch (∆*T*_m_ of +20 °C, Supplementary Fig. 12) and covalent adduct formation of the expected Δ_mass_ was confirmed by intact protein MS analysis when tested in vitro with the recombinant BromoCatch protein (at 2:1 ratio, Fig. 7B and Supplementary Data 1). The utility of the probe for protein analysis in cell lysates was evaluated in HEK293FT cells transfected with an H2B-BromoCatch mammalian expression construct. The H2B-BromoCatch-tagged protein was specifically biotinylated by treatment at increasing concentrations of MR169 for 2 h. Western blot analysis shows specific biotinylation of H2B-BromoCatch at all concentrations tested and no cross-biotinylating activity in parental untransfected or transfected cell line, even at the highest concentrations of probe (Fig. 7C). The anti-H2B antibody detection demonstrates the successful transfection of the H2B-BromoCatch construct (31 kDa) and its band co-localizes with the anti-streptavidin band, confirming protein biotinylation in the cell. We sometimes observed additional bands at 10–15 kDa, in both the anti-H2B and anti-streptavidin blots (Supplementary Figs. 28A and 29D). These lower molecular weight bands are absent in the samples from untransfected cells treated with the MR169 probe, suggesting they are not off-targets of the probe, and are likely fragmentation products of the constructs. Another band was observed at ~65 kDa in the anti-streptavidin blot in both untransfected and transfected cells, only when treated with the compound, suggesting this could be an off-target of MR169 (Supplementary Fig. 29D).

The kinetics of MR169 were evaluated using the same method as for MR116. The second-order constant *k*_inact_/*K*_I_ for MR169 was found to be 3.7 ± 0.5 × 10^4^ M^−1^ s^−1^ (Supplementary Fig. 13, see the “Methods” section, source data). Chemical conjugation of the covalent ligand thus results in a slight reduction of covalent binding efficiency when compared to the ligand alone. The rate of labelling of this non-fluorescent biotin probe is consistent with the labelling rate reported for non-fluorescent probes for SNAP-tag and HaloTag (10^4^–10^5^ M^−1^ s^−1^)^26^.

To evaluate the cellular target selectivity of the BromoCatch biotin probe MR169 and its ability to covalently engage BromoCatch-fused proteins, we performed an affinity (streptavidin) pull-down assay in HEK293 cells transiently transfected with a BromoCatch-GFP construct followed by mass spectrometry analysis of the recovered biotinylated proteins (Fig. 7E). GFP was selected as the exogenous fused protein of interest because of its low background of cellular interactors, minimizing false positives. To ensure confidence in the identified hits, during data analysis we used stringent cut-offs of log_2_ (fold change) of 2 (corresponding to a 4-fold increase/decrease in quantitation) and a statistical cut-off of −log_10_ (*p*-value) of −1.5 (Fig. 7G, H, and Supplementary Data 2). We observed significant enrichment of BromoCatch-GFP protein in transfected cells treated with MR169 (Fig. 7G) compared to untransfected cells (Fig. 7F), consistent with independent western blot analysis of BromCatch-GFP trasnfected HEK293FT cells (Supplementary Fig. 14). Importantly, pre-treatment with the competing ligand ET-JQ1-OMe at high concentration (25 µM) substantially diminished pulldown of BromoCatch-GFP, confirming the probe’s specificity for BromoCatch (Fig. 7H). Interestingly, in addition to BromoCatch-GFP we see enrichment of another 54 proteins (Fig. 7G and Supplementary Data 2), none of which were detected in the untrasfected cells, and which were all displaced with pre-incubation of the competing ligand (Fig. 7F, H and Supplementary Data 2). These proteins could be recruited to BromoCatch-GFP, as non-specific interactors of the fusion protein, likely an artefact of the construct overexpression. The most enriched amongst these hits were ENO1, SURF6 and RBM14. Interaction database searches, e.g. BioGrid5.0, showed no evidence for specific interactions with Brd4, beyond minor associations^57,58^, suggesting these proteins are also artefactual non-specific interactors. We also detected 13 proteins in the untrasfected control cells (Fig. 7F). None of these proteins were pulled down in the transfected cells (Fig. 7G and H), and most of them appear as known frequent contaminants in streptavidin-based pulldowns^59^, suggesting minor background labelling. Together, the data show that BromoCatch is efficiently and highly selectively labelled by our biotin probe MR169 and pulled-down MS-proteomics applications qualified its clean profile suitable for imaging applications in overexpression settings.

**Fig. 8:**
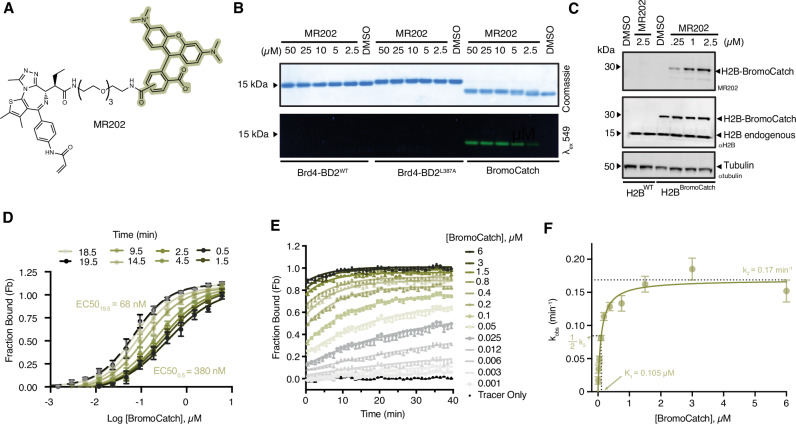
Fluorescent probe for cell lysate studies. **A** Chemical structure of the TMR fluorescent probe MR202. Green shadowing highlights the TMR fluorophore. **B** In-gel assay titration of the probe with purified proteins BromoCatch, Brd4-BD2^WT^, or Brd4-BD2^L387A^. **C** HEK293FT H2B-BromoCatch cell lysate experiment. MR202 specifically detects H2B-BromoCatch at 0.25 µM concentration of the probe. The probe showed no unspecific binding in HEK293FT WT cells when incubated at up to 2.5 µM. **D** EC_50_ changes of probe MR202 (50 nM) obtained from titration with BromoCatch protein (EC50_19.5_ and EC50_0.5_ correspond to the calculated EC_5o_ at 0.5 or 19.5 minutes) (mean ± SD, *n* = 3 technical replicates). **E** Increase in anisotropy values (as fraction bound Fb) of the MR202 probe with increasing concentrations of BromoCatch over 40 min fit to a one-phase association model to obtain the pseudo first order rate constant *k*_obs_ values (mean ± SD, *n* = 3 technical replicates). **F** The *k*_obs_ plotted against the protein concentration and resulting covalent constant *k*_2_ and the affinity constant *K*_1_ values obtained from teh data fit, allowing to calculate the apparent second order constant *k*_app_ (data are mean ± SD of *n* = 3 technical replicates).

One of the most widely used applications of SLPs is fluorescent labelling of proteins of interest. We built a BromoCatch-specific tetramethylrhodamine (TMR) probe based on the MR116 ligand (MR202, Fig. 8A) and tested it in vitro using purified recombinant protein. When the probe was titrated at increasing concentrations against the protein, maximal fluorescence intensity was achieved at stoichiometric levels of protein to probe (10 µM), selectively over Brd4-BD2 and Brd4-BD2^L387A^ (Fig. 8B). To validate utility in a cellular context, HEK293-FT cells transiently expressing H2B-BromoCatch were treated for 2 h with MR202 and the cell lysates analysed by western blotting (Fig. 8C). The membrane was co-stained with anti-H2B antibody (IR800 channel) and tubulin (Alexa546 channel). The transfection was confirmed by the appearance of two H2B bands (H2B^WT^ at 15 kDa and H2B-BromoCatch at 30 kDa) in the H2B-BromoCatch-transfected HEK293FT lysates, with only the lower molecular weight band, corresponding to the endogenous H2B, observable in the non-transfected HEK293FT cells. TMR fluorescence was detected in the Alexa 546 channel, both before and after tubulin co-staining. Bands corresponding to H2B-BromoCatch were observed at all tested probe concentrations and their identity was confirmed by co-localization with the H2B antibody upper band. The labelling efficiency in H2B-BromoCatch U2OS cells with the MR202 probe was compared to that for H2B-HaloTag-transfected U2OS cells using the haloalkane TMR probe, under the same conditions. We observed that the HaloTag construct was labelled more efficiently at lower concentrations than the corresponding TMR probe, which could suggest better permeability of the haloalkane (Supplementary Fig. 15). Ongoing work is focused on systematically evaluating the permeability of BromoCatch probes to optimize intracellular labelling efficiency.

The kinetics of MR202 were determined by incubating a fixed concentration of the probe (50 nM) with increasing concentrations of recombinant BromoCatch protein (1 nM–6 µM). The time-dependent decrease in EC₅₀ are consistent with a covalent binding mechanism (Fig. 8D). The time dependent fluorescence anisotropy increases were monitored over time for each protein concentration (Fig. 8E). The observed rate constants (*k*_obs_) were plotted against protein concentration to derive the apparent kinetic constant *k*_app_ (*k*_2_/*K*_1_) that was calculated to be 2.06 ± 0.36 × 10^4^ M^−1^ s^−1^ (Fig. 8F, see the “Methods” section and Source Data). Consistent with observations for the MR169 probe, the BromoCatch system displayed similar labelling kinetics for the TMR-based MR202 probe. This contrasts with the SNAP-tag and HaloTag systems, where rhodamine-based fluorophores markedly enhance the labelling kinetics, while no fluorophore-induced kinetic improvement was observed for BromoCatch^26^.

**Fig. 9:**
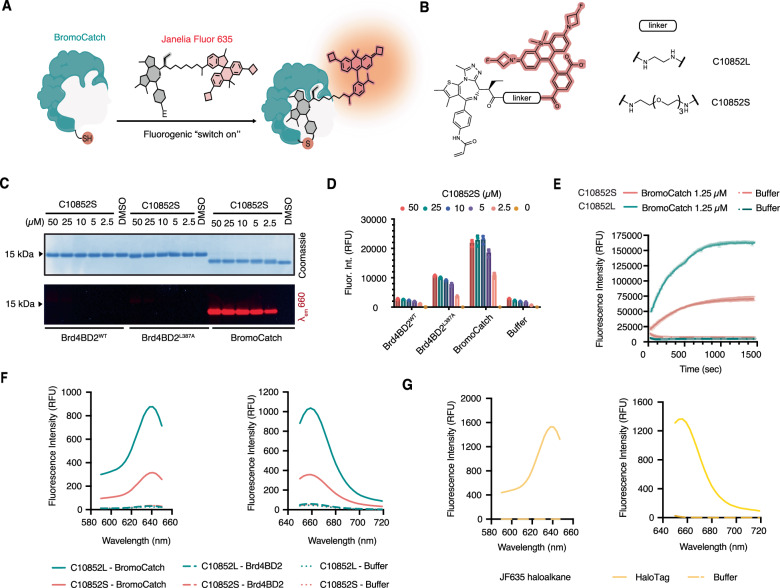
In vitro validation of BromoCatch covalent ligand MR116 functionalized with Janelia Fluor^®^ 635. **A** “Switch-on” principle of Janelia Fluor^®^ 635 BromoCatch probes. Illustration Created in BioRender. Ciulli, A. (2026) https://BioRender.com/eg7i2eu. **B** Structure of C10852S and C10852L probes. Red shadowing highlights the JF635 fluorophore. **C** In-gel assay, titrating the different proteins with increasing concentrations of the C10852S probe. **D** Fluorogenic “switchon” in the presence of the different proteins with increasing concentrations of the C10852S probe (data are mean ± SD, *n* = 3 technical replicates). **E** Time-dependent activation of probes C10852S and C10852L (100 nM) in the presence of 1.25 µM BromoCatch (data are mean ± SD, *n* = 3 technical replicates). **F** Excitation and emission spectra of probes C10852S and C10852L (1 µM) in the presence of BromoCatch or Brd4-BD2^WT^ (10 µM) (data are mean of *n*  =  2 technical replicates). **G** Excitation and emission spectra of JF635 haloalkane in the presence or absence of GST-HaloTag (10 µM) (data are the mean of *n*  =  2 technical replicates). RFU Relative fluorescence units.

To assess the compatibility of BromoCatch with live-cell fluorescence-based applications, a fluorogenic BromoCatch probe using Janelia Fluor^®^ 635 (JF635) was prepared (Fig. 9A)^60^. Janelia Fluor dyes are fluorogenic rhodamine-based dyes with excellent photophysical properties, including high brightness and photostability. These dyes are small, cell-permeable, thanks to their lactone-zwitterion *K*_L–Z_ equilibrium, and have excellent environmental fluorogenic properties, enabling no-wash, live-cell imaging applications. For example, JF635 has been widely used in conjunction with other SLP systems such as Halo- and SNAP-tag^5,61,62^. It is particularly suited to these applications due to its red-shifted emission (away from cellular autofluorescence and enabling less destructive, higher wavelength activation) and preferential *K*_L–Z_ providing a high degree of fluorogenicity. To develop a JF635-based fluorescent probe, we exchanged the TMR fluorophore in MR202, while retaining the original linker (Fig. 9A, B). The resulting JF635 probe C10852S was titrated against a fixed concentration of recombinant BromoCatch protein (10 µM) and covalent labelling analysed by SDS gel. Conveniently, the JF635 fluorophore can be environmentally “switched-on” in the presence of SDS micelles, a useful feature for in-gel protein analysis. This feature allowed us to evaluate the concentration-dependent increase in fluorescence ‘in-gel’, which presented the same concentration-dependent response to that of MR202. These results confirm that the covalent binding to BromoCatch is maintained despite the change of fluorophore (Fig. 9C). Additionally, we assessed the fluorogenicity of the probes in solution prior to SDS-PAGE separation (Fig. 9D, Supplementary Fig. 16). Concentration-dependent increase in fluorescence was observed upon titration against BromoCatch (10 µM), confirming activation upon binding. No fluorogenic activation occurred in buffer alone, where only a low level of fluorescence was observed. The fluorescence intensity remained low and comparable to that in buffer when the probe was titrated against Brd4-BD2^WT^, but not in the case of Brd4-BD2^L387A^, which exhibited a noticeable “switch-on” effect, albeit weaker than that observed with BromoCatch, due to non-covalent binding.

The fluorogenic character of JF635 probes relies on switching on upon binding protein surfaces^62^. In the initial probe design (C10852S), we kept the PEG_3_ linker that was originally used in the AGB1 PROTAC degrader. We hypothesized that the length of the linker could play a part in the efficiency of fluorogenic “switch-on”. Hence, we also prepared a probe with a shorter linker between the MR116 ligand and JF635 (C10852L). The covalency and fluorogenicity of C10852L were confirmed by SDS–PAGE analysis and probe titration using the same protein panel as for C10852S, and we observed comparable results to those obtained with C10852S (Supplementary Fig. 17). To compare fluorogenic performance, activation of both probes was assessed in vitro at low probe concentrations to better resolve differences in signal. Both probes resulted in an increase in fluorescence intensity when the BromoCatch protein was present, but C10852L demonstrated an improvement in signal-to-noise when compared to C10852S probe, likely due to the shorter linker (Fig. 9E). The excitation and emission spectra of the probes (C10852L and C10852S, 1 µM) was measured in presence and absence of BromoCatch or Brd4-BD2^WT^ (Fig. 9F). Both probes activation by an excess BromoCatch (10 µM) resulted in an increment in the fluorescence intensity emitted across the expected emission spectrum, that displayed a maximum *λ*_em_ of at 660 nm. A similar trend was observed across the excitation wavelengths of the JF635 with a maximum *λ*_ex_ at 640 nm upon activation. Importantly, incubation with Brd4-BD2^WT^ showed comparable fluorescence emission to that in buffer. The observed spectra are consistent with the reported JF635 fluorescence spectrum and comparable to that of the HaloTag equivalent system (JF635 haloalkane/GST-HaloTag, Fig. 9G). C10852L gave threefold improvement over C10852S in signal-to-noise ratio, calculated to be 20- and 7-fold for C10852L and C10852S, respectively (Fig. 9F). Indicating that the shorter linked C10852L probe is superior. Notably, the JF635 haloalkane probe achieved the greatest signal-to-noise ratio (100-fold, Fig. 8G), attributable to its minimal background fluorescence in PBS, corresponding to an overall approximate fivefold improvement over C10852L. Together, the data support enhanced fluorogenicity of the shorter-linked C10852L probe.

Recognizing the similarity in chemical structure between our BromoCatch covalent binder MR116 and JQ1-based molecular glues that induce DCAF16-dependent degradation of endogenous Brd4 protein via a template-assisted covalent cross-reactivity towards DCAF16^41,42^, we assessed any undesired on-target DCAF16-mediated degradation of BromoCatch by our JF635 probe compound. We monitored degradation of endogenously HiBiT-tagged Brd4, Brd3, and Brd2 or HiBiT-BromoCatch-Brd4 expressed in HEK293 cells, upon treatment with AGB1, MZ1 and C10852S. While MZ1 and AGB1 rapidly and potently degraded BromoCatch (DC_50_ [4 h] = 151 and 21.2 nM, respectively), C10852S showed no degradation at 4 h in either BromoCatch or untagged BET cell lines (Supplementary Fig. 18 and Supplementary Table 7). This result reassures that our probe does not trigger DCAF16-mediated protein degradation, likely due to a combination of at least two of our design features (i) the “bump”, substantially weakening engagement to wild-type bromodomains and (ii) the functionalisation of the ligand via the carboxylic acid vector, which would disrupt the molecular glue ternary complex^35,41,42^.

**Fig. 10:**
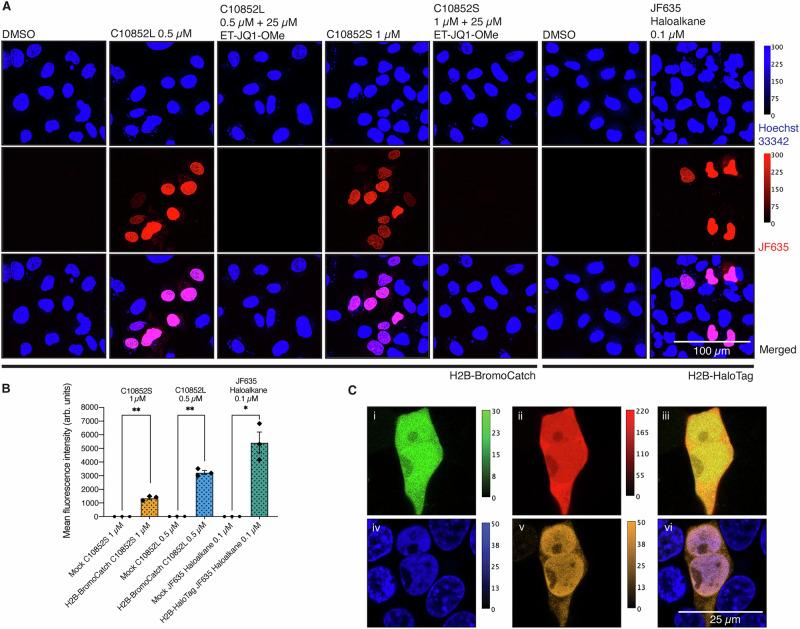
Live-cell imaging, quantification, and orthogonal multiplexed labelling of BromoCatch and HaloTag systems using functionalised Janelia Fluor probes. **A** Live-cell confocal imaging of U2OS cells transiently expressing H2B-BromoCatch /HaloTag to assess probe activation and specificity of C10852L, C10852S, and JF635 Haloalkane. Cells were treated with C10852S (1 µM), C10852L (0.5 µM), or JF635 Haloalkane (0.1 µM) for 4 hours and counterstained with Hoechst 33342. Controls included vehicle-treated cells, mock-transfected cells, and pre-incubation with the competitive inhibitor ET-JQ1-OMe (25 µM, 30 min) for the BromoCatch-expressing cells. Data is shown as *N* = 3 repeats, with two technical fields of view acquired per condition. Images were collected as Z-stacks; representative single z-slices through the centre of the nucleus are shown with intensity scaling set for best display and consistently applied in each experiment. Hoechst 33342 (blue channel) and JF635 channel (red channel). **B** Quantification of mean fluorescence intensity of the Janelia Fluor “switch-on” probes within Hoechst-defined nuclear regions between probe-treated H2B-BromoCatch/H2B-HaloTag-transfected U2OS vs. probe-treated mock-transfected U2OS controls. Data is presented as mean ± SEM, with all individual replicates present in each condition. Statistical significance was determined using an unpaired two-tailed *t*-test, with Welch’s correction; *p*-values reported comparing mock transfection treated and transfected treated conditions being *p* = 0.0049 for C10852S, *p* = 0.0022 for C10852L, *p* = 0.0194 for JF635 Haloalkane, arb. units = arbitrary units. **C** HEK293 cells co-transfected with BromoCatch-GFP and H2B-HaloTag to demonstrate orthogonal multiplexed labelling. Cells were treated simultaneously with C10852L (1 µM) and JF549 Haloalkane (0.1 µM) for 2 h prior to Hoechst 33342 counterstaining. GFP fluorescence was used to localize BromoCatch-GFP. Single-probe validation confirmed that H2B-BromoCatch and H2B-HaloTag cells exhibited robust fluorogenic “switch-on” fluorescence in their respective JF635 and JF549 channels, whereas vehicle-treated or ET-JQ1-OMe–pre-incubated cells showed loss of C10852L activation, confirming specificity. Panels **C** (i–iii) show (left to right): BromoCatch-GFP (green channel), C10852L (red channel), and the merged image. Panels **C** (iv–vi) show (left to right): Hoechst 33342 (blue channel), JF549 Haloalkane (orange channel), and the merged image. Controls included single-probe treatments, ET-JQ1-OMe competition (blocking C10852L binding and activation), non-transfected controls, and vehicle-treated cells (*N* = 2 independent biological repeats). Source data are provided as a Source Data file.

To validate BromoCatch for live-cell fluorescent labelling of tagged proteins and in the process, benchmark its performance against the HaloTag system, we assessed fluorogenic “switch-on” of Janelia Fluor 635-conjugated probes by live-cell confocal microscopy (Fig. 10A). U2OS cells were transiently transfected with previously described H2B-BromoCatch (Figs. 7C and 8C) or H2B-HaloTag, then incubated for 4 h with either C10852S (1 µM), C10852L (0.5 µM), or HaloTag JF635 haloalkane probe (0.1 µM), followed by Hoechst 33342 counterstaining. All probe treatments in transfected cells produced robust nuclear fluorescence, consistent with high permeability and rapid, efficient, and highly specific tag labelling. This nuclear “switch-on” of the BromoCatch probes was prohibited by a 30 min pre-incubation with a saturating concentration of ET-JQ1-OMe (25 µM), indicating that engagement with the tag is necessary for probe activation (Fig. 10A, Supplementary Fig. 19). Further, it was observed that mock-transfected U2OS cells treated with C10852S, C10852L, or JF635 haloalkane showed no fluorescence, like the ET-JQ1-OMe pre-treatment control, confirming that BromoCatch Janelia Fluor activation requires tag expression and binding-site engagement. This is further supported by quantification of mean fluorescence intensity (MFI) from summed Z-stack projections, which showed that both ET-JQ1-OMe and mock treatment controls had ~100–1000’s fold lower mean fluorescence than in transfected probe treated cells (Fig. 10B, Supplementary Table 8). Pearson’s correlation coefficients further support these findings. We observed modest Pearson’s *r* values (≈0.2–0.3), which we attribute to our whole-field quantification that includes the non-transfected cells to avoid selection bias. Nevertheless, because this approach was applied consistently across all conditions, the comparisons remain valid. Pearson’s *r* value correlation coefficients of 0.25 ± 0.03, 0.29 ± 0.06, and 0.30 ± 0.03 were observed for C10852S, C10852L, and JF635 haloalkane, respectively (Supplementary Fig. 20). In contrast, ET-JQ1-OMe pre-treatment or mock transfection reduced Pearson’s r values by ~10 fold compared with transfected, probe-treated cells, owing to a loss of fluorescent “switch-on” in Hoechst 33342 stained nuclear regions (Supplementary Fig. 20). Comparing the MFI values of the probes showed that C10852L (3236 ± 150) is 137% brighter than C10852S (1366 ± 96), despite being used at half the concentration, reflecting the enhanced fluorogenic response observed previously with recombinant BromoCatch (Fig. 9E–F). The JF635 Haloalkane probe was again a further 68% brighter compared to C10852L (5434 ± 767), despite having a 5-fold lower concentration, consistent with the high fluorogenic efficiency of the HaloTag system and supported by side-by-side immunoblotting (Supplementary Figs. 15 and 30).

Fluorescence activation was predominantly confined to the nucleus, however, a small but detectable extranuclear signal was also observed (Fig. 10A). Cytoplasmic-to-nuclear (C/N) ratio analysis of extranuclear fluorescence in the JF635 channel revealed values of 7.1 ± 1.6% for C10852S, 3.1 ± 0.3% for C10852L, and 1.9 ± 0.3% for JF635 Haloalkane (Supplementary Fig. 21). This result indicates that both C10852L and C10852S display predominantly nuclear fluorescence with minimal cytoplasmic signal (especially C10852L), evidencing their high target specificity and low off-target activation. This result highlights that, given ET-JQ1-OMe and mock-treated controls display minimal fluorescent activation (Fig. 10A), very low to non-existent MFI (Fig. 10B, Supplementary Table 8), no colocalisation correlation (Supplementary Fig. 20), and largely equal (C/N) ratios in mock controls (Supplementary Fig. 21), we can reasonably infer that the extranuclear fluorogenic signal arises from true target engagement of our probes with non-nuclear, transiently expressed H2B-BromoCatch and H2B-HaloTag. Collectively, these data establish C10852L as our current best-in-class probe for live-cell imaging of BromoCatch-tagged proteins. Through quantitative MFI measurements, colocalisation analysis, and (C/N) ratio assessment, C10852L demonstrates efficient cell permeability, high intrinsic brightness, and fluorogenic activation that is both highly specific and strongly dependent on BromoCatch engagement.

Demonstrating BromoCatch’s orthogonal compatibility with established self-labelling protein (SLP) systems is essential, particularly for live-cell imaging applications where simultaneous tracking of multiple proteins is increasingly required. To assess whether BromoCatch functions independently of HaloTag without cross-reactivity, we performed multiplexed labelling in HEK293 cells co-transfected with BromoCatch-GFP and H2B-HaloTag. Cells were co-treated with C10852L (1 µM) to label BromoCatch-GFP and JF549^®^ Haloalkane (0.1 µM) to label H2B-HaloTag, followed by a 2-h incubation prior to imaging (Fig. 10C; Supplementary Fig. 22). Hoechst 33342 was used as a nuclear marker to confirm nuclear localization of H2B-HaloTag, while GFP fluorescence verified BromoCatch-GFP expression and enabled comparison with C10852L probe activation. Following co-treatment, both probe signals were independently detected: C10852L fluorescence (panel ii) specifically labelled BromoCatch-GFP, and JF549 HaloTag fluorescence (panel v) selectively labelled H2B-HaloTag. H2B-HaloTag fluorescence was observed within the nucleus, consistent with H2B localization, although a cytoplasmic signal was also detectable. A similar cytoplasmic signal was observed with transiently expressed H2B-BromoCatch (Fig. 10A), suggesting partial mislocalisation or overexpression-associated leakiness of the constructs. In contrast, C10852L fluorescence was distributed throughout both the nucleus and cytoplasm, consistent with the broader cellular distribution of BromoCatch-GFP. The higher concentration of C10852L (1 µM) was chosen to ensure efficient labelling within the more diffuse cytoplasmic environment of BromoCatch-GFP, compared with the spatially confined nuclear localization of H2B-HaloTag. Specificity was confirmed through multiple controls, including single-probe treatments and competitive inhibition with 25 µM ET-JQ1-OMe prior to C10852L incubation. Importantly, no non-specific fluorogenic “switch-on” or signal bleed-through was observed under co-labelling conditions. In an independent experiment, H2B-HaloTag expressing U2OS cells were treated with 1 µM C10852L and H2B-BromoCatch or BromoCatch-GFP expressing U2OS treated with 100 nM JF635 Haloalkane and left to incubate for 2 h. No detectable JF635-channel fluorescent activation was observed for all three conditions, thereby confirming chemical independence between the two labelling SLP strategies (Supplementary Fig. 23). Pearson’s correlation coefficients were also calculated for single- and dual-labelling of dual-transfected HEK293 cells treated with JF549 Haloalkane (100 nM). 100 nM JF549 haloalkane treatment alone had a Pearson’s *r* value of 0.22, while dual treatment of 1 µM C10852L and 100 nM JF549 haloalkane yielded a Pearson’s *r* value of 0.19, indicating that HaloTag labelling efficiency and localization are not perturbed by co-expression of BromoCatch, thus further establishing that both systems function independently without competitive interference (Supplementary Table 9). Together, our findings and data in live cells qualify BromoCatch as suitable for deployment in tandem with HaloTag or other SLP systems such as SNAP-tag in an orthogonal manner, enabling robust multiplexed labelling and expanding the versatility and practical utility of SLPs as widely used chemical biology tools.

## Discussion

We present BromoCatch, a self-labelling protein tag (SLP) platform for protein modification and live cell imaging applications. Leveraging our “bump-andhole” BromoTag system, we engineered a cysteine into the bromodomain ligand binding pocket, creating the BromoCatch double-mutant protein Brd4-BD2^L387A,E438C^. The BromoCatch covalent ligand MR116, designed by introducing an acrylamide electrophilic group at the *para* position of the pendant aryl ring, proved to be the most effective covalent binder. Functionalisation of MR116 allowed the synthesis of a panel of bifunctional probes for diverse applications, including biotinylated probe MR169 for biotin-based detection and pull-down proteomics assays, and the TMR probe MR202 for fluorescence-based detection of BromoCatch in cell lysates. We present two environmentally responsive “switch-on” probes, C10852S and C10852L, which feature selective binding of BromoCatch in cells, with excellent “switch-on” activation and minimal background fluorescence, demonstrating utility and suitability for live-cell imaging. We have also established the compatibility of the BromoTag degrader AGB1 degrader with the BromoCatch system, thus allowing rapid and durable catalytic degradation of BromoCatch-tagged proteins. This offers a key advantage for BromoCatch over systems that rely on covalent PROTAC degradation, such as HaloPROTACs. Together, our data establishes BromoCatch as a versatile new tag for protein labelling and live-cell imaging, with the potential to expand applications in protein manipulation and for multiplexing and induced-proximity using orthogonal SLP platforms.

Our goal in developing BromoCatch was to provide an additional self-labelling tag of small size and specific chemistry, and show utility for cellular protein labelling orthogonal to existing ones, such as HaloTag or SNAP-tag. Here, we provide extensive characterization in vitro and in live cells that qualify BromoCatch as a valuable addition to the field, demonstrating multiplexing and orthogonality between BromoCatch and HaloTag as a representative second tag. HaloTag was selected due to its rapid kinetics, well-characterized ligands, and extensive applications in multicolour live-cell imaging, making it a stringent reference for testing compatibility. Looking forward, it will be interesting to investigate a triple multiplexing experiment with both HaloTag and SNAP-tag. To make it feasible to implement three or more SLP systems in parallel, each SLP must employ orthogonal labelling chemistries to prevent cross-reactivity and must exhibit sufficient spectral separation to minimise bleed-through and cross-excitation. Optimization of treatment conditions should preclude over-labelling or increased background fluorescence. Here, we show BromoCatch to be well-suited to support such applications, as BromoCatch functions by a ligand-induced covalent engagement of a modified bromodomain, distinct from the chloroalkane-functionalised ligands of HaloTag and benzylguanine (BG)-modified substrates of SNAP-tag. Probes disclosed in this work include JF635 probes C10852S/L in the far-red spectrum, but conceivably, the modular small-molecule design of BromoCatch probes enables adaptation for green-to-orange spanning fluorophores. Triple multi-plexing could be accomplished by positioning BromoCatch in the far-red spectrum, while using shorter wavelength probes for HaloTag (e.g. orange emission) and SNAP-tag (e.g., green emission). As this study has already demonstrated BromoCatch–HaloTag multiplexing, extending this to include SNAP-tag would be a logical next step. If implemented successfully with BromoCatch, the triple multiplexing approach would allow simultaneous visualization of multiple targets within the same cellular context under identical experimental conditions, to expand the capacity to interrogate complex biological processes, while reducing the need for sequential experimentation.

We are currently exploring the benefits of the small size of the tag to minimise its impact on cellular proteins. Further optimization of the fluorogenic probes is underway to improve the signal-to-noise ratios of the JF635 series, and efforts are being directed towards gaining structural information on the fluorogenic BromoCatch-probe complex. Future work will explore further applications of probes for this system utilizing different fluorophores, as well as further protein engineering of the BromoCatch protein. It will be warranted to explore the suitability of BromoCatch as a tag to be fused in protein loops^63^ other than at the N- or C-terminus as those sites are often vital to protein function and therefore tagging can preclude endogenous protein function. Future probe conjugates could be designed to bear other functions and activities beyond those exemplified here. In immediate applications, BromoCatch could be used to tag proteins where other tags, such as HaloTag7 have been reported to impair function, such as CB2 receptor^64^, AGO2^65^, COX8A and ATP5ME^21^. More broadly, we envisage a wide range of future applications of BromoCatch, including assay development and enablement of induced-proximity modalities beyond targeted protein degradation, in combination with existing orthogonal SLP platforms and other non-covalent tag systems such as dTag ^66,67^; novel labelling approaches to aid protein detection in cellular structural biology approaches using Cryo-Electron Tomography (cryo-ET) and Cryo correlative light and electron microscopy (CLEM)^68,69^; and new labels for in vivo applications^70^. We anticipate that our BromoCatch system’s favorable properties demonstrated herein will underpin establishment as a broadly used tag for protein labelling and chemical manipulation of biological systems.

## Methods

### Chemistry

Unless otherwise stated, all reagents and solvents were purchased from commercial sources and used without further purification. Full details of the synthetic procedures are included in the Supplementary Methods File.

### Plasmids for recombinant protein expression in *E. coli*

To generate plasmids for recombinant expression in *E. coli*, DNA encoding the respective Brd2-BD2 or Brd4-BD2 mutant was PCR amplified using primers encoding 5’ BamHI and 3’ EcoRI restriction sites. The PCR fragments were double-digested with BamHI and EcoRI, gel-purified, and extracted. Mutant fragments were ligated into pRSF-DUET1 vectors with N-terminal 6xHis-TEV or His-SUMO. All sequences were confirmed by conventional sequencing. Additional mutations were introduced using site-directed mutagenesis with the relevant primer pairs for the desired point mutation (Supplementary Tables 11 and 12).

### Protein expression and purification

Human Brd2-BD2 or Brd4-BD2 mutants were expressed with an N-terminal His_6_-tag in *E. coli* BL21(DE3) at 37 °C with LB supplemented with 50 µg/mL kanamycin once OD_600_ reached 0.8. Protein expression was induced with 0.5 mM IPTG with 50 µM MgCl_2_ added at induction and grown overnight at 18 °C. After harvesting, the cells were resuspended in buffer containing 120 mM sodium phosphate, 500 mM NaCl and 40 mM imidazole and MgCl_2_ (1 mM) and DNase I (10 µg/mL) was added, and cells lysed using Continuous Flow Cell Disruptor (Constant Systems) at 30,000 psi. Cell lysates were clarified by centrifugation at 18,000×*g* for 30 min at 4 °C. The lysate was filtered and loaded onto the HisTrap FF affinity column (GE Healthcare) and eluted with 120 mM sodium phosphate buffer, 500 mM NaCl and 500 mM imidazole. The proteins were then dialyzed against 25 mM HEPES, pH 7.5, 150 mM NaCl, and 1 mM TCEP with protease (1:100 ratio) overnight. This mixture was passed through a HisTrap FF column, concentrated in a 3500 MWCO centrifugal unit (Amicon), and loaded on a Superdex 16/600 size-exclusion column pre-equilibrated in 25 mM HEPES, 150 mM NaCl, 1 mM TCEP, pH 7.5. Pure variants of Brd4 eluted ~0.74 cv and were confirmed using SDS–PAGE. The final proteins were all >95% pure, concentrated, and stored at −80 °C until further use. All chromatography purification steps were performed using a BioRad NGC system at 4 °C. Purity and mass of proteins were confirmed by LC–MS (sequences can be found in Supplementary Table 13).

### Docking studies

A model of the mutants Brd2-BD2^L383V,D434C^ and Brd2-BD2^L383V,M438C^ was generated by introducing the mutation with the Maestro Schrodinger editing tools, using the x-ray structure of Brd2-BD2^L383V^ co-crystallized with ET-JQ1-OMe (6YTM) as a template. The Brd2-BD2^L383V^ co-crystal and the corresponding mutations were prepared using single-point mutations and the Protein Preparation Wizard from Schrodinger, and the corresponding grids were generated with Glide. Ligands were prepared (Ligprep) and docked reversibly (Glide) or covalently (CovDock) in either mutant or reference crystal. The best 5 scored poses from each docked ligand were filtered and analysed visually with Maestro. Root mean square deviation (RMSD) was obtained with respect to the reference crystal for the common structures. All molecular model figures were generated in ChimeraX.

### Differential scanning fluorimetry

Differential Scanning Fluorimetry (DSF) experiments were performed on a Biorad CFX96 RT-PCR machine or in a Nanotemper Phanta (NanoTemper Technologies GmbH). For assays in the Biorad CFX96 RT-PCR machine, ligand (50 µM) or DMSO stock was incubated with Brd4-BD2^WT^, Brd4-BD2^L387A^ Brd4-BD2^L387A,E438C^, Brd4-BD2^L387A,M442C^ (10 µM, 50 µL) in 25 mM HEPES, 100 mM NaCl, 1 mM TCEP for 2 h at room temperature, followed by the addition of SYPRO Orange (1 µL) to give a final dilution of 5 × SYPRO Orange, the final DMSO concentration was 4%. The temperature was ramped up in 1 °C steps between 25 and 95 °C with 30 s incubation at each step. Melting curves were analysed by determining the minimum of the first derivative using the Biorad CFX Manager. For analysis in the Nanotemper Phanta (NanoTemper Technologies GmbH), a similar procedure was used, but no addition of SYPRO Orange was required. Samples containing a mixture of the protein (10 µM) and ligand (50 µM) were taken into capillaries for subsequent reading by NanoTemper, the temperature was similarly ramped up in 1 °C steps between 25 and 95 °C with 30 s incubation at each step. Values of *T*_m_ and were obtained from the melting curve at 350 nm emission (inherent fluorescence emission of aromatic amino acids). Data are average ± SD from *n* = 3 technical repeats.

### Covalent modification of recombinant protein, intact LC–MS analysis

Brd4-BD2, Brd4-BD2^L387A^, Brd4-BD2^L387A,E438C^ and Brd4-BD2^L387A,M442C^ (50 µM), were incubated with a 1:2 P:L concentration of ligands (100 µM) in activity buffer (Supplementary Table 10) at room temperature or at 37 °C. At 2 h, the sample was precipitated by the addition of 4 volumes of cold methanol. The precipitated protein was pelleted by centrifugation and resuspended in an aqueous solution of 15% acetonitrile and 0.1% TFA. Samples were separated using a UHPLC Agilent 1290 Infinity II or UHPLC Agilent 1290 Infinity III over 20 min on a zorbax 300 SB C3 21 × 150 mm, 5 µm column using a 10–75% gradient of acetonitrile 0.05 % TFA in water 0.05% TFA and analysed using an Agilent 6130 or 6135 single quadrupole MS, unless otherwise stated. Spectra were deconvoluted and integrated manually using Agilent LC/MSD OpenLab CDS or ChemStation. The quantification of modified protein was done by integration of peaks on the 280 nm UV or MS chromatogram traces for the unmodified and modified protein. Reported measurements are representative values of at least 2 independent experiments.

### Crystallization of Brd2-BD2^L383A,D434C^

For crystallization, Brd2-BD2^L383A,D434C^ (1.2 mM) was diluted into crystallization buffer (25 mM HEPES, 120 mM NaCl, 0.5 mM TCEP, pH 7.5) at a final concentration of 100 µM in 1 mL. From a 10 mM stock, 12 µL of MR116 was added in 2 µL increments with gentle mixing in between to reach a final concentration of 120 µM (1.2-fold molar excess). Brd2-BD2^L383A,D434C^ and MR116 were allowed to react at ambient temperature for 2 h. The reaction was spun at top speed in a microcentrifuge for 10 min and the supernatant (1 mL) was injected into a 5 mL sample loop and loaded on a 16/600 Superdex s75 pg size-exclusion column (SEC) pre-equilibrated in crystallization buffer. The run was carried out on a BioRad NGC equipped with a multi-wavelength detector and monitored at 215, 255, and 280 nm. Fractions with UV absorbance at 280 nm were analysed using NuPAGE Bis–Tris mini protein 4–12% gels (Invitrogen) with MES running buffer (Supplementary Table 10), run at 200 V for 34 min. The gel was stained using colloidal Coomassie Instant Blue Stain (ISB1L, Abcam), revealing pure BRD2-BD2^L383A,D434C^. The additional UV absorbance at 215 nm suggested the presence of MR116, which was confirmed from the Δ*T*_m_ over 25 °C (*T*_m_ Brd2-BD2^L383A,D434C^ = 45.37 °C and *T*_m_ MR116/Brd2-BD2^L383A,D434C^ = 71.72 °C) using nanoDSF (20–95 °C, at 1 °C/min) on a Prometheus Panta (Nanotemper). With this verification, MR116/Brd2-BD2^L383A,D434C^ complex was concentrated to 610 µM (8.2 mg/mL) using a 3000 MWCO centrifuge concentrator (Amicon). Because MR116 did not exhibit UV-absorbance at 280 nm on the SEC run, the concentration was determined using the molar extinction coefficient at 280 nm(*ε*_280_) = 15,930 cm^−1^*M^−1^. Aliquots of MR116/Brd2-BD2^L383A,D434C^ were stored at −80 °C until use.

Initial crystal screens of MR116/Brd2-BD2^L383A,D434C^ (8.2 mg/mL) were set against commercially available screens (JSCG+, PACT, ProPLEX, Morpheus-I, PGA, Index-HT, PEGion, MIDAS, Classics, and BCS) in 96-well MRC 2-drop sitting well plates with 50 µL reservoirs, using a Mosquito (SPT LabTech) dispensing 200 nl of reservoir and 200 nl of MR-116/Brd2-BD2^L383A,D434C^ for each drop. Plates were sealed and crystals were monitored at 19 °C in a Rock Imager (Formulatrix). Numerous conditions yielded suitable crystals between 1 and 3 days. Crystals were harvested with Dual Thickness MicroLoops (MiTeGen) ranging from 50 to 200 µm. Our reported structure was obtained from a single ~100 µm crystal grown after 2 days in the Morpheus-I condition E9 (PEG 500 MME 40% (v/v), PEG 20,000 20% (w/v), 0.12 M ethylene glycol, 0.1 M Tris–BICINE pH 8.5) and was harvested without additional cryoprotection.

### Macromolecular X-ray data collection

Data sets for all crystals were collected at Diamond Light Source (Didcot, UK) on MX beamline I24 under bag proposal MX-35324-32. A total of 3600 images were recorded on the EIGER2 X CdTe 9M detector positioned at a distance corresponding to a maximum resolution of 1.1 Å, total exposure time of 0.005 s/image, 0.1° oscillation over 360°, wavelength of 0.6199 Å, and beam size of 20 × 20 µm. Data sets were automatically processed SynchWeb/ISPyB for indexing (XDS), scaling, and merging. The crystal was processed in space group *P*2_1_2_1_2 with unit cell dimensions of 32.05, 52.12, 71.64 Å in length and angles of 90° 90° 90°. We estimate that this crystal achieved diffraction at the limit of the beamline and detector, but the highest resolution shells were incomplete. Therefore, we reprocessed the images with a resolution cutoff of 1.3 Å to obtain a complete dataset for structural determination.

### Structure determination of MR116/Brd2-BD2^L383A,D434C^

To facilitate molecular replacement, we generated an atomic model of Brd2-BD2^L383A,D434C^ without MR116 using Boltz-1^71^ and automatic MSA generation^72^. Crystallographic analysis was carried out in CCP4 v8^73^. Initial inspection of the crystal had a Matthews coefficient of 2.28 and a high probability of 1 copy of Brd2-BD2^L383A,D434C^ per unit cell with a solvent content of 46%. Running CCP4 pipeline (Data reduction to complete structure with ligand fitting) with Aimless for data reduction, Phaser for molecular replacement using the Blotz-I model, Buccaneer autobuild, and REFMAC5. This initial attempt resulted in structure factors of *R*_work_ = 0.20 and *R*_free_ = 0.23 and a clear density for MR116. The SMILES string with the saturated CH_2_–CH_3_ analogue of the MR116 acrylamide was used to generate restraints for the ligand and for subsequent linkage with the right carbon valency. The covalent bond was formed using coot, between the beta CH_3_ carbon in the ligand and the sulfur in C434 of Brd2-BD2^L383A,D434C^ in CPP4, and the hydrogen was removed manually. This was used as a covalent restraint for further refinement. After several rounds of manual model building in COOT and REFMAC, the final structure factors were *R*_free_ = 0.16 and *R*_work_ = 0.15 for the predominant bond conformation. An alternate conformation of the covalent bond was built and further refined with REFMAC5 to give a final *R*_free_ = 0.16 and *R*_work_ = 0.15. The polder map (omit map) was prepared in PHENIX, omitting the ligand density MR116 and the cysteine, and then visualized in UCSF ChimeraX.

### Sodium dodecyl sulfate polyacrylamide gel electrophoresis of recombinant (modified) proteins

Protein samples were separated by gel electrophoresis on NuPAGE™ 4–12%, Bis–Tris precast polyacrylamide gels (Thermofisher) unless otherwise stated. Protein or cell lysate proteins were separated by SDS–PAGE gel electrophoresis. Samples (10 µL) were prepared by the addition of loading buffer (10 µL, 4×LDS sample buffer, 2% DTT, see Supplementary Table 10) and then heated at 95 °C for 5 min. Protein samples (15–28 μL) were loaded into NuPAGE 4–12% Bis–Tris Gels and electrophoresed for 45 min at 180 V in MES buffer. The Protein Standards ladder (Bio-rad) (5 μL) was used as a molecular weight reference. Following electrophoresis, gels were imaged with ChemiDoc MP Imaging System for fluorescence read-out at the appropriate wavelength (for fluorescent labelling experiments) or stained for 15 min with Instant Blue Coomasie® stain (Abcam), destained, and imaged using Bio-Rad molecular imager. Bio-Rad Image Lab 6.1 was used to process the data. Protein concentrations were measured prior to use in assays using a NanoDrop Microvolume Spectrophotometer.

### In vitro protein labelling with fluorescent covalent ligands

Brd4-BD2, Brd4-BD2^L387A^ or Brd4-BD2^L387A,E438C^ (10 µM) were co-incubated with either DMSO or increasing concentrations of MR202 or C10852S in activity buffer for 1 h. Concentrations covered a range of concentrations from sub-stoichiometric up to excess (2.5 to 50 µM). Samples (10 µL) were mixed with a solution of LDS/DTT (10 µL), loaded (15 µL) and separated by NuPAGE™ 4–12%, Bis-Tris precast polyacrylamide gels as described above and imaged for fluorescence before staining with Coomassie. In-gel data is representative of 2 independent experiments. In-gel fluorescence ChemiDoc (Bio-Rad) channels: (TAMRA channel *λ*_ex_: 500–550 nm, *λ*_em_: 550–600 nm; JF635 channel *λ*_ex_: 625–650 nm, *λ*_em_: 675–725 nm).

### Cell culture

HEK293, HEK293FT and U2OS cell lines were used in this paper. U2OS and HEK293 were purchased from the American Type Culture Collection (ATCC). HEK293FT Cell Line was obtained from Invitrogen (R70007). All cell lines were cultured in 10 cm cell culture plates with Dulbecco’s Modified Eagle Medium high glucose, GlutaMAX™ Supplement (DMEM high glucose, GlutaMAX™ Gibco^TM^ Catalogue number: 10566016) in a humidified incubator with 5% CO_2_ at 37 °C. DMEM media was supplemented with 10% foetal bovine serum (FBS, Gibco^TM^ Catalogue number: A5256701).

### Construct design

DNA constructs for this project were all designed in SnapGene (Version 8.0.2). Constructs were purchased as custom gene synthesis products that were integrated into pCDNA 3.1(+) using Invitrogen’s geneART gene synthesis services. During synthesis, codon optimisation was used for improved expression. Construct used for NanoBRET validation of electrophilic warheads consisted of pcDNA3.1(+) vector harbouring a Nanoluciferase-Brd4-BD2^L387A, E438C^ 3’ to the CMV promoter (containing residues 333-460 of Brd4-BD2). Construct used to probe functionality of the bifunctional probes using the elongated version of BromoCatch consisted of a pcDNA3.1(+) vector harbouring a H2B-Brd4-BD2^L387A,E438C^, or Brd4-BD2^L387A,E438C^-GFP (containing residues 333–460 of Brd4-BD2) 3’ to the CMV promoter. Construct used for comparative analysis with the HaloTag SLP consisted of a pcDNA3.1(+) vector harbouring a H2B-HaloTag 3’ to the CMV promoter. Construct used to probe functionality of the bifunctional probes using the shortened version of BromoCatch) consisted of a pcDNA3.1(+) vector harbouring a H2B-Brd4-BD2^L387A,E438C^ (containing residues 351-460 of Brd4-BD2) 3’ to the CMV promoter. Donor construct used to produce the endogenously tagged BromoCatch-Brd4 cell line consisted of a pMA-RQ vector with a donor insert consisting of an eGFP-IRES-HiBiT-BromoTag-Brd4 insert modified into a eGFP-IRES-HiBiT-BromoCatch-Brd4 donor by introducing the E438C mutation, the insert is flanked in-between left and right homology arms for the N-terminus of Brd4. Two single pX459 vectors harbouring U6-driven Brd4 gRNA sequences and a pCMV-driven Cas9 cassette were also developed by the MRC-PPU reagents and services. Site-directed mutagenesis was performed to convert the BromoTag construct in the original pMA-RQ donor into a BromoCatch donor by introducing the E438C substitution. Reactions using the Q5^®^ Site-Directed Mutagenesis Kit (NEB #E0554S) contained 25 ng of the original pMA-RQ plasmid, Q5 Hot Start High-Fidelity master mix (used at 1x final concentration from 2x stock), and 0.5 µM forward (TCCTGACCATTGTGTGGTGGCCATG) and reverse (GGGTTGTACTTATAGCAGTTG) primers. PCR conditions were: 98 °C initial denaturation; 25 cycles of 98 °C for 10 s, 63 °C for 30 s, and 72 °C for 300 s; followed by a final extension at 72 °C for 120 s. PCR products (1 µL) were treated with 5 µL KLD reaction buffer, 1 µL KLD enzyme mix, and 3 µL nuclease-free water at room temperature for 5 min. The reaction (5 µL) product was subsequently transformed into DH5α *E. coli* by heat shock at 42 °C for 30 s, recovered in SOC media at 37 °C, and plated on ampicillin selection plates overnight at 37 °C. Colonies were expanded, plasmids purified using the Monarch^®^ Plasmid Miniprep Kit (#T1010), and submitted for sequencing (MRC PPU), DNA sequencing and Services using the pEGFP-C1 forward primer (CATGGTCCTGCTGGAGTTCGTGAC). Sequencing confirmed successful introduction of the E438C substitution, and the construct was subsequently used in the downstream CRISPR knock-in workflow. Plasmid maps for mammalian expression vectors can be found on figshare. Constructs used in this manuscript (Supplementary Table 14) are available upon request.

### Treatment of pCMV H2B-BromoCatch and BromoCatch-GFP transfected HEK293FT cells with MR202 and MR169

HEK293FT cells were plated at a density of 1 × 10^6^ cells per well of a six-well plate, which were subsequently incubated at 37 °C, 5% CO_2_ overnight. These cells were subsequently transfected with 2 µg of pCMV_H2B-BromoCatch or pCMV_BromoCatch-GFP at a 3:1 ratio of DNA to transfection reagent using FuGENE HD, left to incubate at rt for 20 min and subsequently added dropwise to cells. The cells were then incubated at 37 °C, 5% CO_2_ for 72 h. Following incubation, the cells were then subsequently treated with either DMSO (Vehicle), 2.5, 1 and 0.25 µM MR169 (H2B-BromoCatch and BromoCatch-GFP) or MR202 (H2B-BromoCatch). In parallel to this, non-transfected HEK293FT cells were treated with DMSO (Vehicle) and 2.5 µM MR169 or MR202. These treated cells were incubated at 37 °C, 5% CO_2_ for 2 h prior to harvesting.

### Cell harvesting and lysis

To harvest adherent HEK293FT cells, the medium was removed, and the cells washed with rt PBS. Cells were detached by incubating with RIPA buffer (1% Triton X-100, 0.1% SDS, and 1:200 Protease inhibitor cocktail, Supplementary Table 10) and incubating at 4 °C for 15 min. The lysate was centrifuged (13,000×*g*, 4 °C, 10 min) to pellet cell debris, and the resulting supernatant was transferred to fresh Eppendorf tubes.

### Determination of total protein concentration

Total protein concentration of the lysate was determined by a BCA protein assay (Thermo Scientific) and the absorbance at 562 nm was measured and plotted using a PHERAStar plate reader. Cell lysates were diluted to 2 mg/mL with miliQ water.

### Western blotting

Proteins in the electrophoresed gels were transferred onto a nitrocellulose membrane for 90 min at 90 V, at 4 °C. The primary antibodies were diluted in 5% milk in TBS-T (TBS, 0.1% Tween). The membrane was incubated with the primary antibodies (Histone H2B (D2H6) Rabbit mAb #12364 (Cell Signalling Technologies, 1:1000 dilution), and IRDye^®^ 800CW Streptavidin (LICORbio, 926-32230), BRD4 EPR5150(2), Anti-HiBiT Monoclonal antibody N720A, anti-GFP polyclonal antibody DU 1574) overnight at 4 °C. The membrane was washed with TBS-T (3 × 5 min), and then treated with the appropriate secondary antibody (IRDye® 800CW anti-rabbit (no. 926-32211, LiCor, 1:10,000 dilution), and hFABTM rhodamine anti-tubulin (no. 12004165, Biorad, 1:5000 dilution), IRDye® 800CW anti-sheep (Thermo Fisher, SA5-10060) diluted in 5% milk in TBS-T (TBS, 0.1% Tween), and then incubated for 1 h at rt. The membrane was washed with TBS-T (3 × 5 min) before being imaged on a Bio-rad molecular imager. Western blot data are representative of two independent measurements.

### Sodium dodecyl sulfate polyacrylamide gel electrophoresis of lysates

Cell lysates (20-30 µg) were separated by SDS-PAGE gel electrophoresis on NuPAGE™ 4–12%, Bis–Tris precast polyacrylamide gels (Thermofisher) unless otherwise stated. Samples (15 µL) were prepared by the addition of LDS/DTT loading buffer (5 µL, 4 × LDS sample buffer, 2% DTT) and then heated at 95 °C for 5 min. Protein samples (20 μL) were loaded into NuPAGE 4–12% Bis–Tris Gels and electrophoresed for 45 min at 180 V in MOPS buffer. The Protein Standards ladder (Bio-rad) (5 μL) was used as a molecular weight reference. Following electrophoresis, gels transferred into nitrocellulose membranes as stated above.

### Copper catalysed Alkyne Azide Click (CuAAC) recombinant protein labelling experiments

Copper-catalysed Alkyne Azide Click (CuAAC) assays were performed in 1.5 mL Eppendorf tubes at 37 °C or at room temperature, in the dark and gently shaken for 1 or 16 h, unless otherwise stated. The wild-type Brd4-BD2 or the mutant protein Brd4-BD2^L387A,E438C^ (50 μM, 200 μL) were incubated with the alkyne-containing ligand MR155 (75 μM) for 1 h in 25 mM HEPES 7.5 pH, 100 mM NaCl and 1 mM TCEP. To this mixture, sCy5-azide (300 μM) was added, followed by a premixed solution of THPTA/CuSO_4_ (5:1, 0.5 mM/0.1 mM) in 25 mM HEPES 7.5 pH, 100 mM NaCl 1 mM TCEP and NaAsc 20 (5 mM) as added. The reaction was mixed for 1 hour at 37 °C then left at room temperature and samples were taken at 1  and 16 h. Samples were diluted 5-fold and prepared for gel electrophoresis by mixing the sample (10 μL) with a mixture of LDS/ DTT (10 μL). Prepared samples (10 μL) were separated by NuPAGE^TM^ 4–12%, Bis–Tris precast polyacrylamide gels. The gels were imaged with ChemiDoc (sulfoCyanine 5 fluorescence in the Cyanine 5 channel (excitation range of 625–650 nm; emission range of 675–725 nm). The gels were subsequently stained with Instant Blue Coomasie® stain, destained, and imaged in the GelDock.

### Activation of probes C10852S and C10852L with BromoCatch measured by time-dependent increase in fluorescence intensity

A fixed concentration of the probe C10852S or C10852L (100 nM) was incubated with a saturating concentration of the BromoCatch protein (1.25 µM) or in activity buffer in a final volume of 15 µL. The time-dependent increase in fluorescence intensity was measured in one-minute intervals over 30 min (ex 635–720, em 670–740).

### Fluorogenicity of C10852S and C10852L probes

For quantification measurements to evaluate the fluorogenic character of probes C10852S or C10852L, Brd4-BD2, Brd4-BD2^L387A^, Brd4-BD2^L387A,E438C^ (50 µL, 10 µM) in activity buffer were co-incubated for 2 h with DMSO (1 µL) or in the presence of increasing concentrations of the probe (1 µL) to give final concentrations of the probe ranging from 2.5 to 50 µM. The probe (2.5–50 µM) in 0.1% TFA in ethanol or 4% SDS was used as a positive control of fully switched on C10852S or C10852S. Samples (10 µL) were plated in triplicate in a CORNING^®^ black 384-well low volume plate and relative fluorescence units (RFU) measured using a GloMax Promega microplate reader (JF635 channel *λ*_ex_: 625 nm, *λ*_em_: 660–725 nm).

### Excitation and emission spectrum measurements experiment for C10852L, C10852S or HaloTag

C10852L or C10852S (1 µM) were co-incubated with either Brd4-BD2, or Brd4-BD2^L387A,E438C^ (10 µM) or activity buffer for 2 h, in duplicate. The excitation spectrum for each sample was measured across the 500–750 nm wavelength range and the emission spectrum from 650 to 720 nm.

JF635 Haloalkane probe (1 µM) was co-incubated with GST-Halotag (10 µM) or PBS for 2 h, in duplicate. The excitation spectrum for each sample was measured across the 500–750 nm wavelength range and the emission spectrum from 650 to 720 nm.

### CRISPR knock-in of BromoCatch-Brd4 HEK293 cells

To perform the knock-in of BromoCatch into the N-terminus of BRD4, 1 mL of HEK293 cells in DMEM:10% FBS was plated at a density of 1 × 10^6^ cells per mL into a single well of a six-well plate in the 24 h leading up to the initiation of the experiment. The cells were subsequently transfected the following day using Fugene HD using the donor pMA-RQ vector containing the eGFP-IRES-HiBiT-BromoCatch (333–460) sequence flanked by homology arms. Simultaneously, the cells were transfected with two single gRNA/cas9 containing pX459 vectors containing Brd4 gRNA_1_(TGGGATCACTAGCATGTCTGCGG) and gRNA_2_(ACTAGCATGTCTGCGGAGAGCGG) (Supplementary Table 15). The HEK293 cells were transfected with 1 µg of the pMA-RQ donor vector and with 0.5 µg of each pX459 gRNA vector. The DNA was mixed in 100 µL of Optimem media and Fugene HD was added at a ratio of 1:3 DNA:Fugene HD or 2 µg:6 µL total and this mixture was left to form complexes for 20 min at rt. This mixture was added dropwise to the adherent HEK293 cells in 2 mL of fresh DMEM:10% FBS and the cells were left to incubate in a humidified incubator with 5% CO_2_ at 37 °C overnight. The following day, cells were washed in PBS before fresh DMEM:10% FBS was applied. The HEK293 cells were left to incubate in a humidified incubator with 5% CO_2_ at 37 °C for 72 h to allow for recovery post-transfection. The surviving HEK293 cells were subsequently re-transfected with the CRISPR reagents for BromoCatch insertion into Brd4 using the same conditions stated above, allowing for a further 72 h to recover post-transfection in a humidified incubator with 5% CO_2_ at 37 °C. Following this, the surviving HEK293 cells were subsequently prepared for fluorescence-activated cell sorting (FACS).

### Cell sorting

The surviving HEK293 cells from the previous stage were subsequently trypsinised using trypsin–EDTA (0.25%). Once in suspension, the trypsin–cell mixture was neutralized with DMEM:10% FBS. The cell suspension was pelleted at 233×*g* for 5 min. The cell pellet produced was subsequently resuspended in Optimem supplemented with 1% FBS at a concentration of 1 × 10^6^ cells per mL. Parental non-transfected HEK293 cells were used as a baseline control for GFP expression. Single-cell clones were generated by FACS using an SH800 cell sorter (Sony Biotechnology) at Dundee University’s Flow Cytometry and Cell Sorting Facility. A 488-nm laser was used for the excitation of fluorescence and the generation of light scattering. Forward angle light scatter (FSC) and backscatter were detected using 488 ± 17 nm band-pass filters. Cells were distinguished from debris based on FSC-area (A) and SSC-A measurements. Single cells were distinguished from doublets and clumps based on FSC-A and FSC-width measurements. GFP fluorescence was detected using a 525 ± 50 nm band-pass filter, and autofluorescence was detected using a 600 ± 60 nm band-pass filter. GFP-positive cells were identified by first assessing the background GFP and autofluorescence of parental non-transfected HEK293. Using the measurements for GFP and autofluorescence of this sample, a collection gate was set, which identified GFP-positive cells. The transfected HEK293 cells were then analysed, and GFP-positive cells were sorted for collection. A single GFP +ve cell was sorted into individual wells of 96-well plates in 200 μL of 50% filtered pre-conditioned media from healthy cells and 50% fresh DMEM:10% FBS. Sorted plates were subsequently spun down at 233×*g* for 5 min and left to grow in a humidified incubator with 5% CO_2_ at 37 °C for 2 weeks. After 2 weeks, all visible colonies were expanded to 24, 12, and then subsequently to six-well plates and cryogenically preserved prior to validation.

### CRISPR validation

Validation of BromoCatch-Brd4 knock-in was accomplished initially by performing Nano-Glo^®^ HiBiT Lytic Detection (Promega; N3030) on the post-FACS single-cell sorted clones. The clones were initially trypsinised and the cell suspension was plated out onto individual wells of a white-walled 96-well plate at a density of 2 × 10^4^ cells per well in 100 µL of DMEM:10% FBS. The HiBiT lytic reagent, consisting of a lytic buffer, fumarizine substrate and the complementary LgBIT protein, was then prepared and added to the plate containing the CRISPR cell suspension following the manufacturer’s instructions. Upon addition of the lytic substrate, the plate was spun on an orbital shaker for 5 min to encourage lysis and left for a further 5 min to reach peak luminescence. Luminescence was then recorded on a BMG Labtech PHERAstar luminescence plate reader. Data extracted from this analysis was analysed to identify clones that contained >100× higher luminescence relative to the parental wild-type control. A FACS-sorted clone that displayed a high luminescent signal was chosen and subsequently validated by western blotting.

The luminescent clone was plated at a density of 1 × 10^6^ cells per well into three wells of a six-well plate using 2 mL of DMEM:10%FBS and left to incubate in a humidified incubator with 5% CO_2_ at 37 °C for 16 h prior to initiation of experimentation. This adherent clonal cell line was subsequently treated with DMSO (vehicle), MZ1, or MR170 at 1 µM for a total treatment time of 6 h. The clone was washed with PBS and 100 µL of RIPA lysis buffer containing cOmplete™ Protease Inhibitor Cocktail (11697498001, Roche) and Benzonase^®^ Nuclease (E1014, Millipore) at manufacturer-specified concentration. Total protein quantity was determined using the BCA protein assay (#23225, Pierce, Rockford, Illinois). The samples were then prepared and loaded onto a NuPAGE 4–12% bis–tris midi gel along with PageRuler™ Prestained Protein Ladder, 10–180 kDa (26817) as a molecular weight marker (Thermo Fisher Scientific), followed by the transfer of proteins onto nitrocellulose membranes at 80 V for 80 min (EMD Millipore). The nitrocellulose membrane was split, and each was blocked for 1 h at rt using 5% milk in TBS-T. The membranes were probed for Brd4 (Abcam, Ab128874, 1:1000), HiBiT monoclonal antibody (N7200) and left overnight to incubate at 4 °C on an orbital shaker. Following overnight incubation with the primary antibodies at 4 °C, the membranes were incubated with secondary antibodies (anti-rabbit, Abcam AB216773, 1:5000 or anti-mouse, Abcam AB216774, 1:5000) and hFABTM rhodamine anti-tubulin antibody (Biorad, 12004165, 1:10,000) for 1 h and then imaged with a Bio-Rad imager (LI-COR Biosciences). Western blots were prepared in Image Lab v6.1.0 from Bio-Rad (LI-COR, Biosciences). Using this validation approach, it was determined that this clone contained a heterozygous insert of HiBiT-BromoCatch-Brd4 and is thus referred to as an endogenous BromoCatch-Brd4 HEK293 cell line for all subsequent experiments.

### HiBiT live-cell kinetic degradation assay of MR170

The endogenous BromoCatch-Brd4 HEK293 cell line was used to perform a live-cell kinetic degradation assay to determine the activity of the functionalised degrader probes for BromoCatch MR170. Cells were plated at a density of 1 × 10^6^ cells into two wells of a six-well plate in 2 mL of DMEM:10% FBS and left to adhere for 16 h in a humidified incubator with 5% CO_2_ at 37 °C. The cells were then transfected with 2 µg of pCMV-LgBIT expression vector (Promega, N2681) using Fugene HD at a ratio of 1:3 DNA: transfection reagent. The DNA: transfection reagent mixture was incubated for 20 min at room temperature and then subsequently added dropwise to the cells and left to incubate in a humidified incubator with 5% CO_2_ at 37 °C overnight.

The following day, the transfected cells were trypsinised, pooled, counted, and plated at a density of 2 × 10^4^ cells per well into an adherent white-walled 96-well plate in 100 μL of DMEM and incubated overnight. The following day, fresh DMEM was added containing 1x Endurazine (Promega) and incubated at 37 °C, 5% CO_2_, for 2 h. The plate was then imaged for luminescence using a GloMax Discover (Promega) imager as a baseline pre-read, then subsequent addition of 2.5 µM of MR170 or 1 µM MZ1, AGB1 or equivalent DMSO was added to individual wells. A breathable film was placed over the plate and continuously imaged for a period of 9 h on a GloMax Discover (Promega) set to 37 °C. This was performed with two technical replicates per condition in each independent repeat and performed as two independent repeats. The data produced from this experiment were subsequently analysed in Graphpad Prism 10.2.3. Measurements were first normalized to the pre-treatment baseline (*t* = 0) for each well, followed by normalization to the DMSO vehicle control at each time point. Data is expressed as a percentage of the DMSO control. The first post-treatment image was defined as *t* = 0 due to manual loading; subsequent time points are set as 2.8 min per read, accurate to machine time at one decimal place. Data were fitted using “a one-phase exponential decay model” to extract the maximal degradation (*D*_max_) and degradation half-life (*t*^₁/₂^) for each bifunctional molecule.

### HiBiT lytic degradation assay of MZ1, AGB1 and C10852S

The endogenous BromoCatch-Brd4 HEK293 cell line, along, with endogenous Brd4, Brd3 and Brd2-HiBiT lines, previously published from our group^35^, were plated at a density of 2 × 10^4^ cells per well of a white-walled 96-well plate, 16 h prior to initiation of experiment to enable adherence. At initiation of the experiment, cells were incubated in DMEM:10% FBS containing DMSO (vehicle), MZ1, AGB1 and C10852S at a concentration range of 10 µM–0.1 nM. Cells were left to incubate for 4 h at 37 °C, 5% CO_2_, and 95% humidity. At the assay endpoint, cells were treated with HiBiT lytic reagent following the manufacturer's instructions. The plates were then imaged for luminescence on a BMG Labtech PHERAstar plate reader. The data produced from this experiment were subsequently analysed in Graphpad Prism 10.2.3, with degradation curves being produced using a “log(inhibitor) vs. response (three parameters)” model to extract the DC_50_ nM for each bifunctional, normalized to the DMSO (vehicle) control.

### NanoBRET competition ligand binding assay of electrophilic ligands

To perform the NanoBRET competition assay, HEK293 cells were first transfected with the pCMV Nanoluciferase-Brd4-BD2^L387A, E438C^-(333-460) pCDNA (+) 3.1 vector, described above. HEK293 cells were first plated at a density of 1 × 10^6^ cells into each well of a six-well plate and incubated at 37 °C, 5% CO_2_ overnight. To perform the transfections, 2 µg of the Brd4-BD2^L387A, E438C^-Nanoluciferase vector was added to 100 µL of OptiMEM media and 6 µL of Fugene HD was added for a 1:3 DNA:Fugene HD ratio and gently mixed and left at room temperature for 20 min. After this incubation period, the mixture was added dropwise onto the HEK293 cells plated the day prior and then left to incubate in a humidified incubator with 5% CO_2_ at 37 °C for at least 16 h.

Transfected HEK293 cells were trypsinised, pooled, counted, and resuspended in Opti-MEM with 10% FBS. Cells (85 µL) were plated into non-adherent white 96-well plates at 2 × 10⁴ cells per well. 1 µM ET-JQ1-BODIPY probe final concentration, 5 µL, was added and incubated for 5 min at rt with shaking. Electrophilic warheads (10 µL, 20× stocks in OptiMEM; final range 100 µM–2 pM) or DMSO control were added, followed by incubation at 37 °C, 5% CO₂ for 10 min. NanoBRET Nano-Glo live cell substrate with extracellular NanoLuc inhibitor (50 µL, 3×) was then added, and luminescence (460 nm) and fluorescence (610 nm) were measured using a PHERAstar plate reader. BRET ratios were normalized and analysed in GraphPad Prism (v10.2.3) using a three-parameter log(inhibitor) vs. response model to determine IC₅₀ values relative to the DMSO control.

### NanoBRET residence time of electrophilic ligands

To perform the NanoBRET residence time assay, HEK293 cells were first transfected with the pCMV Nanoluciferase-Brd4-BD2^L387A, E438C^-(333-460) vector, described above. Two days prior to initiation of the experiment 1 mL of HEK293 cells was plated at a density of 1 × 10^6^ cells per mL into one well of a six-well plate and the cells were incubated at 37 °C, 5% CO_2_ overnight. To perform the transfection, 2 µg of the vector was added to 100 µL of OptiMEM media and 6 µL of Fugene HD was added for a 1:3 DNA:Fugene HD ratio and gently mixed and left at room temperature for 20 min. After this incubation period, the mixture was added dropwise onto the HEK293 cells plated the day prior and then left to incubate in a humidified incubator with 5% CO_2_ at 37 °C for at least 16 h prior to initiation of experimentation.

Transfected HEK293 cells were trypsinised, counted, and resuspended in Opti-MEM containing 10% FBS at 1 × 10⁵ cells/mL. Cells were distributed into tubes and treated with either 250 nM MR116, 250 nM ET-JQ1-OMe, or DMSO, alongside controls: BRET_null_ (25 µM ET-JQ1-OMe), BRET_max_ (no probe), and a no-tracer condition in 1x Endurazine DMEM:10% FBS. After 2 h incubation (37 °C, 5% CO₂), cells were washed three times and plated into white 96-well plate at 2 × 10⁴ cells/well in triplicate. In the BRET_null_ control, cells were replated in 25 µM ET-JQ1-OME final concentration and sustained this throughout the total BRET kinetic analysis.  1x Endurazine and extracellular inhibitor were added, and baseline luminescence (450 nm) and fluorescence (600 nm) were measured. ET-JQ1-BODIPY tracer (2.5 µM) was then added (excluding no-tracer wells), A breathable film was then placed on the plate and the plate was imaged continuously for ~119 min at 37 °C. Data were analysed in GraphPad Prism (v10.2.3) using a one-phase decay model, with background subtraction (no-tracer control) and normalization to BRET_max_ (100%) and BRET_null_ (0%). Experiments were performed with three technical replicates per condition within each independent repeat, with a total of two independent repeats (*n* = 2). Data were analysed using GraphPad Prism (v10.2.3). Measurements were first normalized to the pre-treatment baseline (*t* = 0) for each well, followed by background subtraction using the no-tracer control. Data were then normalized to BRET_max_ (100%) and BRET_null_ (0%). Due to a manual loading step, the first post-treatment measurement was defined as *t* = 0; subsequent time points are set as 0.6 min per read, accurate to machine time at one decimal place. Normalized data were fitted using a “one-phase exponential model” for qualitative comparison of NanoBRET signal increase between conditions.

### BromoCatch and HaloTag immunoblotting comparison

U2OS cells were plated at a density of 1 × 10⁶ cells per well in six-well plate one day prior to transfection. Cells were transfected with 2 µg of either pCMV-H2B-BromoCatch (351-460) or pCMV-H2B-HaloTag using FuGENE HD at a 3:1 reagent-to-DNA ratio, following a 20-min pre-incubation in Optimem, with the transfection mixture added dropwise to the cells. Transfected cells were incubated for 48 h under standard culture conditions, after which they were placed in DMEM containing 400 µg/mL geneticin for 2 weeks to enable selection for stable integration of the constructs.

Following selection, cells were treated with vehicle, 0.25, 1, or 2.5 µM MR202 (for H2B-BromoCatch) or HaloTag TMR ligand for 2 h. Cells were then washed and harvested in RIPA lysis buffer. Parallel mock-transfected U2OS cells treated with 2.5 µM of each probe served as controls for non-specifc labelling.

Cell lysates (20 µg per sample) were prepared by the addition of 4× LDS sample buffer containing 2% DTT and heated at 95 °C for 5 min. Proteins were separated by SDS–PAGE using NuPAGE™ 4–12% Bis–Tris gels in MOPS running buffer (Supplementary Table 10) at 120 V for 2 h using PageRuler™ Plus Prestained Protein Ladder, 10–250 kDa (26619) as a molecular weight marker. Proteins were subsequently transferred to nitrocellulose membranes at 80 V for 80 min, and membranes were blocked in 5% milk in TBS-T at rt for 1 h prior to imaging. MR202 and HaloTag TMR probe binding was visualized using a Bio-Rad imager (emission 602–650 nm). Membranes were then incubated with a rhodamine-conjugated anti-tubulin secondary antibody for 1 h in 5% milk in TBS-T at rt to confirm equal loading and imaged again using the rhodamine channel on the Bio-Rad imager. This experiment was performed as three independent biological replicates.

### Cell culture and treatment conditions for affinity pull-down mass spectrometry analysis of MR169 against GFP-BromoCatch

HEK293 cells were seeded at a density of 1 × 10⁶ cells per well in six-well plates and maintained under standard culture conditions (37 °C, 5% CO₂). Cells were transiently transfected with 2 µg pCMV-BromoCatch-GFP (333–460) using FuGENE HD at a 3:1 reagent-to-DNA ratio, added dropwise to each well. Following a 72-h transfection period, BromoCatch-GFP-expressing cells were treated with 1 µM MR169 for 2 h. Control conditions were run in parallel and included vehicle-treated mock-transfected cells, vehicle-treated GFP-BromoCatch-transfected cells, mock-transfected cells treated with 1 µM MR169, and a competition condition in which GFP-BromoCatch-expressing cells were pre-incubated for 15 min with 25 µM ET-JQ1-OMe prior to the addition of 1 µM MR169 (Biotin) for 2 h. At the end of all treatments, cells were washed gently three times with PBS and harvested in RIPA lysis buffer. Experiments were performed across *N* = 4 independent biological repeats.

### Proteomics analysis

The cells were lysed in RIPA buffer. The amount of protein was measured using the BCA protein assay. The lysate was precleared with Agarose Control Resin (Pierce, 26150) 30 µL of the beads were added to the cell lysate (300 µg protein at 1000 µL volume RIPA), incubated in a rotating wheel (4 °C) for 15 min, centrifuged (16,200×*g* for 5 min) and the supernatant was taken into a new tube. High-Capacity Streptavidin Agarose beads (Pierce, 20359) were washed in RIPA buffer and 30 µL of the beads were incubated with 300 µg of the precleared cell lysate and rotated at RT for 1 h. After incubation, the beads were washed 5 times with RIPA buffer (50 mM Tris–HCl + 0.5% NP40). The elution was performed by boiling each sample in 50 µL 5% SDS at 95 °C for 10 min. Beads were pelleted by centrifugating at 16,100×*g* for 1 min, and the eluate was collected into a new Eppendorf. Elutes were reduced using a final concentration of 10 mM Pierce DTT (A39255, Thermo Fisher Scientific) in Triethylammonium bicarbonate buffer (TEABC) (T7408, Sigma Aldrich) at 60 °C for 40 min. Then, the samples were incubated at room temperature for 20 min in the dark with 20 mM iodoacetamide. (A39271, Thermo Fisher Scientific). Afterward, samples were processed with S-Trap™ mini spin column (PROTIFI, C02-mini) according to manufacteŕs instructions. Finally, tryptic digestion was performed by incubating the processed sample with 2 µg Pierce Trypsin Protease MS-grade (90058, Thermo Fisher Scientific) dissolved in 100 µL 100 mM TEABC overnight at 37 °C. Samples were then eluted from the column and transferred to a fresh Eppendorf tube. The dried peptides were reconstituted in 1% formic acid and analysed on an Orbitrap Ascend Tribrid mass spectrometer coupled with Thermo Fisher Scientific Vanquish Neo UHPLC. The peptides were enriched on a trap column and separated on an analytical column (Easy-Spray PepMap Neo C18, 2 μm, 75 μm × 150 mm) at 300 nl/min. Chromatographic separation was performed using a gradient elution: 5% BufferA (0,1% formic acid) and 35% buffer B [90% ACN, 0,1% Formic Acid] at 64 min. The total run time was 100 min. The mass spectrometer was operated in a data-dependent acquisition mode. A survey full scan MS (from *m*/*z* 300 to 1350) was acquired in the Orbitrap at a resolution of 120,000 at 200*m*/*z*. The AGC target for MS1 was set as 10^6^ and the ion filling time as 251 ms. The most intense ions with charge state ≥2 were isolated and fragmented using higher collision dissociation (HCD) fragmentation, with 25% normalized collision energy, and detected at a mass resolution of 30,000 at 200*m*/*z*. The isolation window was set at 1.1. The AGC target for MS_2_ was set as 1.5 × 10^5^ and ion filling time set at 59 ms, while dynamic exclusion was set for 60 s. The proteomics raw data were searched using SEQUEST HT search engines with Proteome Discoverer 3.0 (Thermo Fisher Scientific). The following parameters were used for searches: Precursor mass tolerance 10 ppm, Fragment mass tolerance 0.02, Enzyme: trypsin, Mis-cleavage: −1, Fixed modification: carbamidomethylation of cysteine residues, Dynamic modification: oxidation of methionine. The data were filtered for 1% PSM, peptide and protein level FDR. The precursor-based LFQ workflow was used for the label-free quantitation. Furthermore, the data were transformed on a log2 scale, followed by imputation of missing values from a normal distribution with “Processing → Imputation → Replace missing values from normal distribution.” Then, Perseus will shrink the distributions to a factor of “0.3” (width), shift it down by “1.8” (down shift) SDs, and simulate some random values that make up values to fill up the missing values. In addition, we used the whole matrix (mode). These analysed data were used to generate volcano plots using GraphPad Prism software.

### Confocal live-cell imaging of BromoCatch and HaloTag probe activation

Live-cell confocal imaging was performed to evaluate activation and specificity of the BromoCatch probes C10852S and C10852L, as well as the HaloTag-reactive JF635 Haloalkane dye, in U2OS cells expressing nuclear-localized H2B-BromoCatch or H2B-HaloTag. Three days prior to imaging, 1 × 10⁶ U2OS cells were plated per well in six-well plates and allowed to adhere overnight. 48 h prior to imaging, adherent cells were transfected with 2 µg of either pCMV-H2B-BromoCatch (351-460) or pCMV-H2B-HaloTag using FuGENE^®^ HD at a 1:3 DNA-to-reagent ratio and incubated overnight. The following day, transfected cells were trypsinised and re-plated at a density of 2 × 10⁴ cells per well into ibidi µ-Slide 18-well chambered coverslip alongside mock-transfected U2OS cells. Cells were allowed to adhere for a further 16 h under standard culture conditions (37 °C, 5% CO₂).

For probe activation experiments, culture medium was replaced with Opti-MEM supplemented with 10% FBS containing either 1 µM C10852S, 0.5 µM C10852L, or 0.1 µM JF635 Haloalkane. Cells were incubated with probes for 4 h (37 °C, 5% CO₂). Vehicle controls consisted of DMSO-treated transfected and mock-transfected cells. Mock controls comprised probe-treated U2OS cells lacking transfected constructs. For competitive binding controls, cells were pre-treated with 25 µM ET-JQ1-OMe for 30 min prior to the addition of C10852S 1 µM or C10852L 0.5 µM to block BromoCatch engagement. Following probe incubation, cells were washed three times with PBS to remove unbound dye. Hoechst 33342 (5 µg/mL) was added for 20 min to label nuclei in Opti-MEM;10% FBS, after which cells were washed once with PBS and replenished with pre-warmed Opti-MEM;10% FBS prior to imaging. Chamber slides were transferred immediately to the microscope for live-cell imaging.

Confocal images were acquired using a Zeiss LSM 880 confocal microscope equipped with a Plan-Apochromat ×63/1.4 NA oil-immersion objective. Imaging was performed under live-cell environmental control at 37 °C and 5% CO₂. Laser excitation wavelengths were 405 nm for Hoechst 33342 and 633 nm for JF635. Emission detection windows were set to 431–493 nm for Hoechst and 635–721 nm for JF635. Images were acquired sequentially to prevent spectral crosstalk. For each experimental condition, two technical repeats were captured. Z-stack images were collected at 1.3 µm intervals across the full nuclear volume, spanning a total depth of 17.29 µm with 14 slices captured. Laser power, detector gain, and all acquisition settings were maintained constant across experimental conditions, technical replicates, and independent biological repeats. Acquisition parameters were standardized across experiments, with a field of view of 134.95 µm × 134.95 µm, a pixel size of 0.13 µm, 12-bit image depth, a scan time of 3.77 s, and line averaging set to 2.

Raw images were acquired using Zen Black Zen 2.3 sp1 [black] (Zeiss). Representative single optical sections through the centre of the nucleus were used for figure presentation, using a defined 0–300 (main figure) and 0–600 (Supplementary Fig.) colorimetric intensity scale for the Hoechst (431–493 nm) and 0-300 for the JF635 (635–721 nm) channel for best display and was performed consistently for all presented images. While summed Z-stack projections were generated where indicated for quantitative analysis. Downstream image analysis, including region-of-interest definition, fluorescence intensity measurements and colocalisation analysis, was performed using Fiji (ImageJ, v1.54j), with identical analysis parameters applied across all conditions and replicates to ensure consistency and reproducibility.

### Analysis and quantification of live-cell imaging of BromoCatch and HaloTag probe activation

#### Mean fluorescence intensity (MFI) measurements

Z-stacks were first split into their respective single channels (Hoechst and JF635) and converted into summed Z-projections. These flattened images were used to define regions of interest (ROIs), with nuclei in the Hoechst channel marked as ROIs to measure MFI in the JF635 channel. 4 ROIs were captured per field of view, and two fields of view were analysed per independent repeat. JF635 intensities for each field were background-subtracted and averaged to generate an average MFI per independent replicate per treatment. This analysis was performed for C10852L, C10852S, and JF635 Haloalkane treatments, along with their mock-transfected controls, as well as the ET-JQ1-OMe competitive controls. Replicate averages were combined in GraphPad Prism v10.4.1, and a two-tailed unpaired *t*-test with Welch’s correction was performed to compare MFI between transfected and mock-transfected U2OS cells.

#### Colocalisation analysis

Individual Z-slices centred on the middle of nuclei with optimal focus were selected for each channel. These Z slices were analysed using the JaCoP plugin in Fiji v1.54p to calculate Pearson’s *r* values, applying the Costes automatic thresholding method. Pearson’s *r* values obtained for each technical replicate were averaged to generate a single Pearson’s *r* value for each independent repeat per condition. The averages from three independent repeats were then combined and graphed in GraphPad Prism v10.4.1 with S.E.M calculated for all three independent replicates.

#### Cytoplasmic vs. nuclear fluorescence analysis

Z-stacks were split into their respective single channels (Hoechst and JF635) and converted into summed Z-projections. ROIs were defined with nuclei in the Hoechst channel marked for nuclear MFI in the JF635 channel. A second ROI was captured for the brightest regions outside each nucleus in the JF635 channel to serve as a surrogate cytoplasmic measurement, as saturating the JF635 signal highlighted cytoplasmic regions above background. Four nuclear and cytoplasmic ROIs were captured per field of view, and two technical replicates were analysed per condition for each independent repeat. To estimate the relative cytoplasmic vs nuclear fluorescence, all intensities were background subtracted and then the cytoplasmic intensity was divided by the nuclear intensity per cytoplasmic/nuclear pair, presented as a percentage, the four percentages were averaged to provide a single percentage per image. These values from two technical repeats were then averaged to produce a single percentage for each independent repeat. This analysis was performed for C10852L, C10852S, and JF635 Haloalkane treatments, along with their mock-transfected and ET-JQ1-OMe pre-treatment controls. The averages from the three independent repeats were combined and graphed in GraphPad Prism v10.4.1 with mean ± SEM calculated for all three independent replicates.

### Live-cell multiplexed imaging of BromoCatch and HaloTag

#### Cell culture and transfection

HEK293 cells were plated at a density of 1 × 10⁶ cells per well in six-well plates and maintained under standard culture conditions (37 °C, 5% CO₂) prior to transfection. Cells were co-transfected with 1 µg BromoCatch-GFP and 1 µg H2B-HaloTag using FuGENE HD at a 3:1 reagent-to-DNA ratio, added dropwise to each well, and incubated for 48 h to allow robust expression of both constructs. Mock-transfected HEK293 cells, maintained under identical conditions but without plasmid DNA, were prepared in parallel as controls. Following transfection, cells were harvested by trypsinisation, counted, and replated at 2 × 10⁴ cells per well into ibidi µ-Slide 18-Well chamber coverslip, where they were allowed to adhere for 16 h under standard culture conditions (37 °C, 5% CO₂).

#### Multiplexed orthogonal labelling

For live-cell labelling, cells were treated simultaneously with 1 µM C10852L (for BromoCatch activation) and 0.1 µM JF549 HaloTag ligand for 2 h at 37 °C. After incubation, cells were washed gently three times with warm Opti-MEM;10% FBS to remove unbound dye, and Hoechst 33342 (5 µg/mL) was added for 20 min to stain nuclei prior to imaging. Control conditions included: (i) co-transfected cells treated with only C10852L or only JF549 HaloTag ligand, (ii) competition controls in which cells were pre-incubated for 30 min with 25 µM ET-JQ1-OMe prior to 1 µM C10852L treatment, and (iii) mock-transfected cells exposed to both probes, verifying probe specificity and orthogonality.

#### Confocal imaging

Live-cell imaging was performed on a Zeiss LSM 880 confocal microscope equipped with a Plan-Apochromat ×63/1.4 NA oil-immersion objective under environmental control (37 °C, 5% CO₂). Laser excitation wavelengths were 405 nm for Hoechst 33342, 488 nm for eGFP and JF549, and 633 nm for JF635. Emission detection windows were set to 437–467 nm for Hoechst 33342, 499–523 nm for GFP, 576–624 nm for JF549, and 657–688 nm for JF635. Acquisition parameters were kept constant across experiments: 33.74 µm × 33.74 µm image size, 0.07 µm pixel size, 8-bit depth, 28.05 s scan time, line averaging of 8, and fixed detector gain and laser power across replicates. Images were captured as single Z-slices through the centre of nuclei with sequential acquisition for each channel.

#### Image analysis

Raw images were acquired using Zen Black Zen 2.3 sp1 [black] (Zeiss). All image analysis, including colocalisation measurements (Pearson’s *r* values), was performed in Fiji/ImageJ (v1.54j) using the Costes automatic thresholding method as described above. All multiplexing experiments were performed across *N* = 2 independent biological replicates, with three technical repeat images captured for each condition.

### Live-cell imaging of BromoCatch and HaloTag cross reactivity

Additionally, a separate imaging experiment was performed to determine if there was any cross-reactivity of BromoCatch to the HaloTag system. HEK293 cells were plated at a density of 1 × 10⁶ cells per well in six-well plate and maintained under standard culture conditions (37 °C, 5% CO₂) prior to transfection. Cells were transfected with either 2 µg BromoCatch-GFP (333-460), H2B-Bromocatch (351–460) or H2B–HaloTag using FuGENE HD at a 3:1 reagent-to-DNA ratio, added dropwise to each well, and incubated for 48 h to allow robust expression of the constructs. Following transfection, cells were harvested by trypsinisation, counted, and replated at 2 × 10⁴ cells per well into ibidi µ-Slide 18-Well chamber coverslip, where they were allowed to adhere for 16 h under standard culture conditions (37 °C, 5% CO₂). For live-cell labelling, cells transfected with H2B-BromoCatch or GFP-BromoCatch were treated with 100 nM JF635 Haloalkane ligand and cells transfected with H2B-HaloTag were treated with 1 µM C10852L for 2 h at 37 °C. After incubation, cells were washed gently three times with warm Opti-MEM supplemented with 10% FBS to remove unbound dye, and Hoechst 33342 was added for 20 min to stain nuclei prior to imaging. Live-cell imaging was performed on a Zeiss LSM 880 confocal microscope equipped with a Plan-Apochromat ×63/1.4 NA oil-immersion objective under environmental control (37 °C, 5% CO₂). Laser excitation wavelengths were 405 nm for Hoechst 33342, 488 nm for eGFP, and 633 nm for JF635. Emission detection windows for H2B-BromoCatch and H2B-HaloTag were set to 431–493 nm for Hoechst 33342, and 635–721 nm for JF635. Images were acquired sequentially to prevent spectral crosstalk. For each experimental condition, two technical repeats were captured. Z-stack images were collected at 1.33 µm intervals across the full nuclear volume, spanning a total depth of 17.29 µm with 14 slices captured. Laser power, detector gain, and all acquisition settings were maintained constant across experimental conditions, technical replicates, and independent biological repeats. Acquisition parameters were standardized across experiments, with a field of view of 134.95 µm × 134.95 µm, a pixel size of 0.13 µm, 12-bit image depth, a scan time of 5.66 s in the GFP-BromoCatch experiment and 3.77 s in both the H2B-BromoCatch and H2B-HaloTag conditions, and line averaging set to 2.

Raw images were acquired using Zen Black Zen 2.3 sp1 [black] (Zeiss). All image downstream optimisation was performed in Fiji/ImageJ (v1.54j). Cross-reactivity experiments were performed as *N* = 2 independent biological replicates, with two technical repeat images captured for each condition.

### In vitro kinetics—competition-based assay for *k*_app_ determination on MR116 and MR169

Kinetic data from microplate reader assays to evaluate the kinetics of the MR116 ligand and probe MR169 were obtained using a competition assay previously described in our group^48^. The data was analysed using a simplified pseudo-first order model to fit the data, to calculate *k*_inact_/*K*_I_. Extended kinetics method and text equations are available in the Supplementary Information file.

1 $$P+I{\to }^{{k}_{{{inact}}}/{K}_{{{{\rm{I}}}}}}\,{PI}$$

To monitor the MR116 ligand binding kinetics, we synthesized a sulfonated sCy5-labelled reversible tracer (MR133). The fluorescence polarization (mA) of a 2-fold serial dilution from a top concentration of 3 µM BromoCatch in 10 nM of the probe in activity buffer was measured to determine the *K*_L_ (BMG Labtech PHERAstar— firmware v1.33).

Protein titration and the curves were fitted using GraphPad Prism 9, using one-site fitting models to determine the tracer binding constant, *K*_L_ and decide the protein:tracer ratio to use in the competition assay. Samples were run in triplicate in 384-well plates, using a total volume per well of 15 μL. All conditions were run in triplicate (*n* = 3 technical replicates). The bound fraction of the probe is measured using the equation:

2 $${F}_{{{{\rm{b}}}}}=\frac{{A}_{{{{\rm{f}}}}}-A}{{A}_{{{{\rm{f}}}}}-{A}_{{{{\rm{b}}}}}}$$

where,

*A* is the measured anisotropy.

*A*_f_ is the free anisotropic value for the fluorescent probe

*A*_b_ is the bound anisotropic value for the fluorescent probe

The time-dependent reduction in anisotropy was measured for a 2-fold serial dilution of compounds MR116 or MR169 in a mixture of 10 nM probe and 30–60 nM Brd4BD2^L387A,E438C^ (*F*_b_ vs. time plot).

The *k*_obs_ from *F*_b_ versus time graph were obtained by fitting a one-phase exponential decay for each concentration curve. The *k*_obs_ obtained at each of the compound concentrations were plotted against the concentration of inhibitor using Michaelis–Menten model of GraphPad Prism 9, which provides the *k*_inact_ (maximum potential rate of inactivation, *V*_max_) and the apparent *K*_I_ (*K*_m_, concentration of MR116 at which *k*_obs_  =  *k*_inact_/2). The observed rate constant, *k*_obs_ is related to *K*_I_ and *k*_inact_ by the equation:

3 $${k}_{{{{\rm{obs}}}}}=\frac{{k}_{{{inact}}}[I]}{{K}_{{{{\rm{I}}}}}+[I]}$$

Taking into account that this is a competition assay. The *K*_I_ calculated using the Michaelis–Menten model yields *K*_I,app_ and is converted into *K*_I,_ using the following relationship.

4 $${K}_{{{{\rm{I}}}}}=\frac{{K}_{{{I}},{{app}}}}{1+\,\frac{[L]}{{K}_{{{{\rm{L}}}}}}}$$

### In vitro kinetics for direct binding of TMR probe MR202 *k*_app_ determination

We qualified the binding kinetics of the TMR probe MR202 to BromoCatch by assessing the direct binding using FP. Time-dependent measurements of the protein titrations were analysed in GraphPad Prism 9 using one-phase association model to obtain the apparent binding constant (*k*_app_, M^−1^ s^−1^), using a protocol adapted from Wilhem et al. ^26^. Extended kinetics method and text equations are available in the Supplementary Information file.

Time-dependent binding was interpreted using a simplified pseudo-first-order kinetic scheme, where *P* is BromoCatch and *I* is MR202.

5 $$P+I{\to }^{{k}_{{{app}}}}\,{PI}$$

Assays used a fixed MR202 concentration (50 nM) and titrated the BromoCatch protein from a top concentration of 6 µM with a two-fold dilution factor. Probe’s anisotropy was recorded over time to capture the approach to steady state at each protein concentration, with data fitted to a one-phase association model in Prism 9 to obtain the observed first-order rate constant, k_obs_ that is related to *K*_1_ (affinity constant) and *k*_2_ (covalent constant) by the equation:

6 $${k}_{{{{\rm{obs}}}}}=\frac{{k}_{2}[P]}{{K}_{1}+[P]}$$

The resulting ${k}_{{{{\rm{obs}}}}}$ values were then plotted versus [BromoCatch] and fitted to the standard hyperbolic Michaelis–Menten model using GraphPad Prism 9 to obtain the *k*_app_ (*k*_2_/*K*_1_).

Assays were performed in triplicate in 384-well plates (total volume 15 µL per well). Probe binding was reported as the fraction bound ${F}_{{{{\rm{b}}}}}$, calculated using Eq. (2). All conditions were run in triplicate (*n* = 3). Free probe wells defined ${A}_{{{{\rm{f}}}}}$; saturating protein defined ${A}_{{{{\rm{b}}}}}$. “Probe only” and DMSO controls were included on each plate.

### Glutathione (GSH) reactivity assay

The covalent ligands (1/10 μM) were incubated at 37 °C with 5 mM GSH in 100 mM potassium phosphate buffer, pH 7.4 (0.1% DMSO). The analytes were separated with ACQUITY UPLC BEH C18, 1.7 µm 2.1 × 50 mm Column (Part No. 186002350), and the mobile phase consisted of 0.1% formic acid in water/acetonitrile with a linear gradient of organic phase. Samples were then analysed with an LC (Shimadzu LC-40)/MS/MS (Triple Quad 6500+). From the mass spectrum, peak area ratios (peak area analyte/peak area of a stable internal standard) were calculated and the percent compound remaining was determined relative to time zero. Rate of disappearance of analyte (*k*_e_) and half-life (*T*_1/2_) were calculated by fitting to a pseudo-first-order kinetic equation. *C*_*t*_ is the Concentration of analyte after time, *t*. *C*_0_ is the concentration of analyte at time = 0.

7 $${C}_{t}={C}_{0}\cdot {{{{\rm{e}}}}}^{-{k}_{e}\cdot t}$$

8 $${C}_{t}=\,\frac{1}{2}{C}_{0}$$

9 $${T}_{\frac{1}{2}\,}=\frac{{Ln}2}{-k}=\frac{0.693}{-k}$$

### Statistics and reproducibility

Data are presented as average (±SD) for technical replicates, or mean (±SEM) for biological replicates. For representative data in Figs. 4, 5, 7c, 8c, b and 9, experiments were repeated independently with similar results at least twice, uncropped gels and blots and independent repeats are provided in Supplementary Figs. in the Supplementary file.

### Reporting summary

Further information on research design is available in the Nature Portfolio Reporting Summary linked to this article.

## Supplementary information

Supplementary Information

Description of Additional Supplementary Files

Supplementary Data 1

Supplementary Data 2

Reporting Summary

Transparent Peer Review file

## Source data

Source Data

## Data Availability

X-ray crystallographic data have been deposited in the Protein Data Bank, accession code of Brd2-BD2^L383A,D434C^, in complex with compound MR116 is PDB 9QRK. All other data supporting the findings of this study are provided in the main figures and in the Source data file and Supplementary Information files, including uncropped gels. Plasmids generated in this study are available from the corresponding author upon reasonable request. All mass spectrometry proteomics (Biotin affinity pull-down mass spectrometry analysis in a transiently transfected BromoCatch-GFP HEK293 line) data have been deposited in the PRIDE data repository under accession codes: PXD074567. All microscopy images produced in this publication are available via Figshare [10.6084/m9.figshare.32174592]. Source data are provided with this paper.
