# Supplementary material for: BromoCatch: a self-labelling tag platform for protein modification and live cell imaging
Source: Nat Commun. 2026 May 13;17:6406. doi: 10.1038/s41467-026-72539-w (PMC13376172; doi:10.1038/s41467-026-72539-w)
Supplement: Supplementary file 1 — Supplementary Information [file 41467_2026_72539_MOESM1_ESM.pdf]

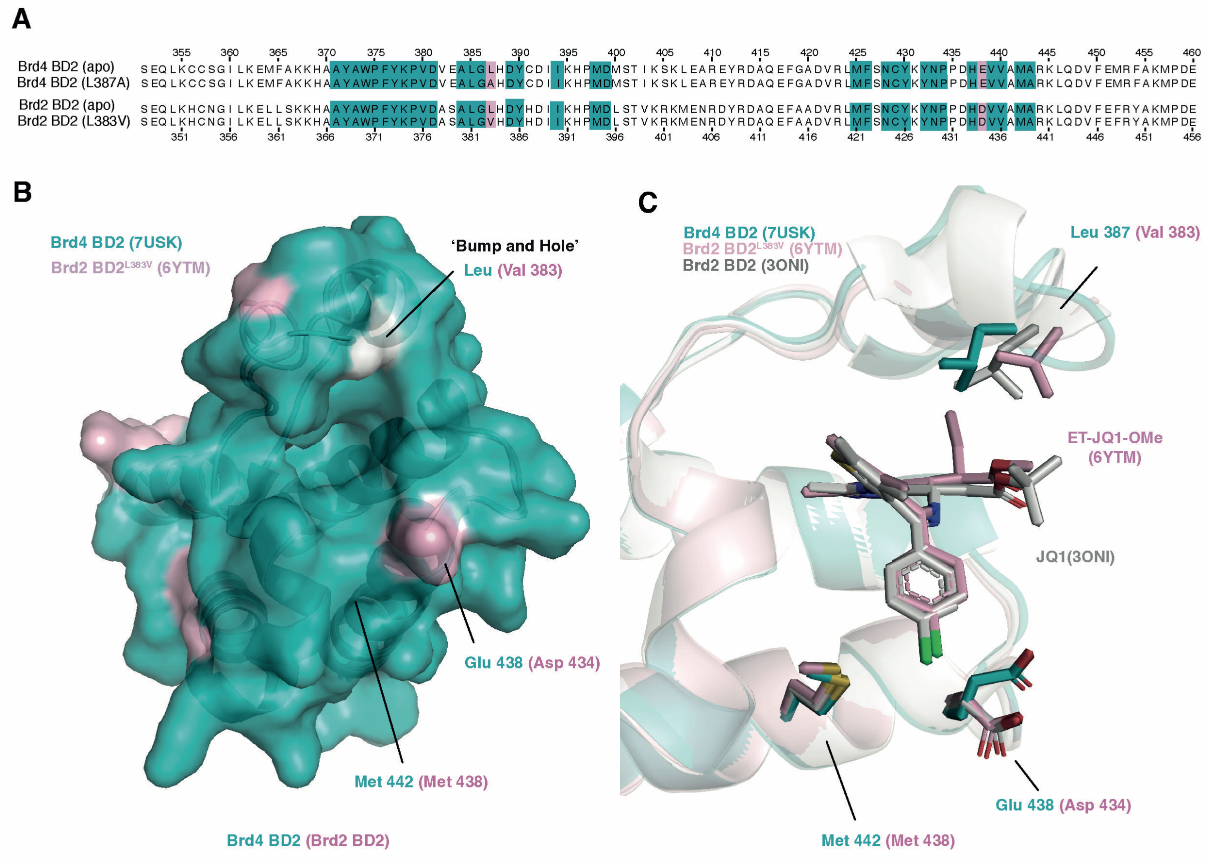

Supplementary Figure 1. Sequence identity and structural conservation of the second bromodomain (BD2) of Brd2 and Brd4. A) Sequence alignment shows the homology of the JQ1 binding pocket in Brd4-BD2 and Brd2-BD2 and the corresponding 'bump and hole' mutants Brd2-BD2^{L383V} and Brd4-BD2^{L387A}. Highlighted in cyan or pink are residues that are within 15 Å from the ligand in the acetyl-lysine binding pocket of the bromodomain. B) Surface conservation of Brd4-BD2 (cyan) and Brd2-BD2 (pink), aligned from crystal stuctures 7USK and 6YTM, respectively. Non-coserved residue positions highlighted in pink. Leu387 in Brd4-BD2 (L387A mutant in BromoCatch) corresponds to Leu383 in Brd2-BD2, that is mutated into Val (L383V) in the 6YTM crystal structure which was used as the structure for the *in silico* docking studies. C) Structural superposition of Brd4-BD2 (cyan) and Brd2-BD2 (pink) illustrates the position of the amino acids chosen to engineer the mutations in BromoCatch.

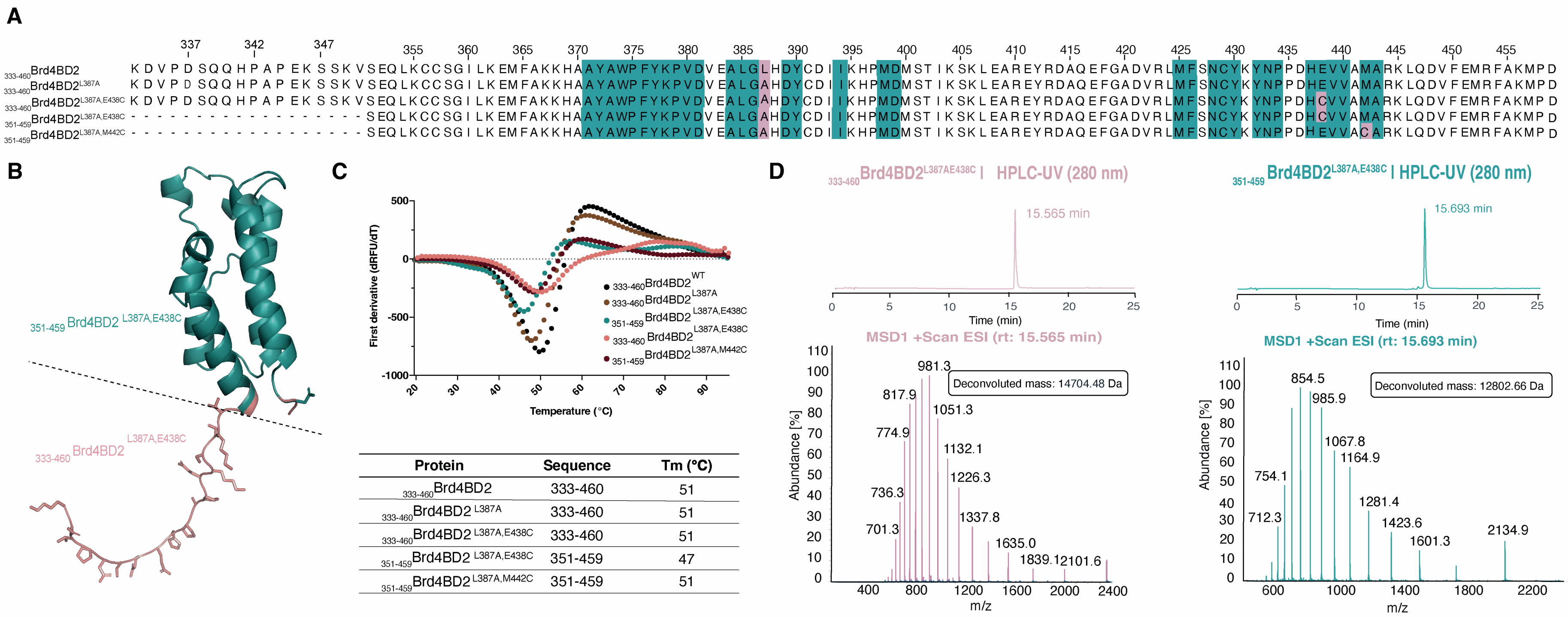

Supplementary Figure 2. Design and characterization of a short construct of Brd4-BD2 for BromoTag/Catch. A) Sequence alignment of the relevant region of Brd4-BD2 where the corresponding mutations are highlighted. B) Structural superpositioning of Brd4-BD2 for the long (333-460) and the short construct (351-459), highlighting the constructs boundaries. C) Melting temperature (Tm) for the different cysteine-containing mutants of Brd4-BD2 was determined by nanoDSF. The data shows that the designed mutants have similar stability to that of Brd4-BD2 wild-type (WT). We observed a decrease of 4 °C in the Tm of the short construct of Brd4-BD2^{L387A,E438C} compared to WT, but no shift in Tm with the long construct of Brd4-BD2^{L387A,E438C}. Tm values are mean of N=3. D) Purity of recombinant proteins and corresponding observed mass by intact protein MS for Brd4-BD2^{L387A,E438C} long (pink) and short (cyan) constructs (N=1).

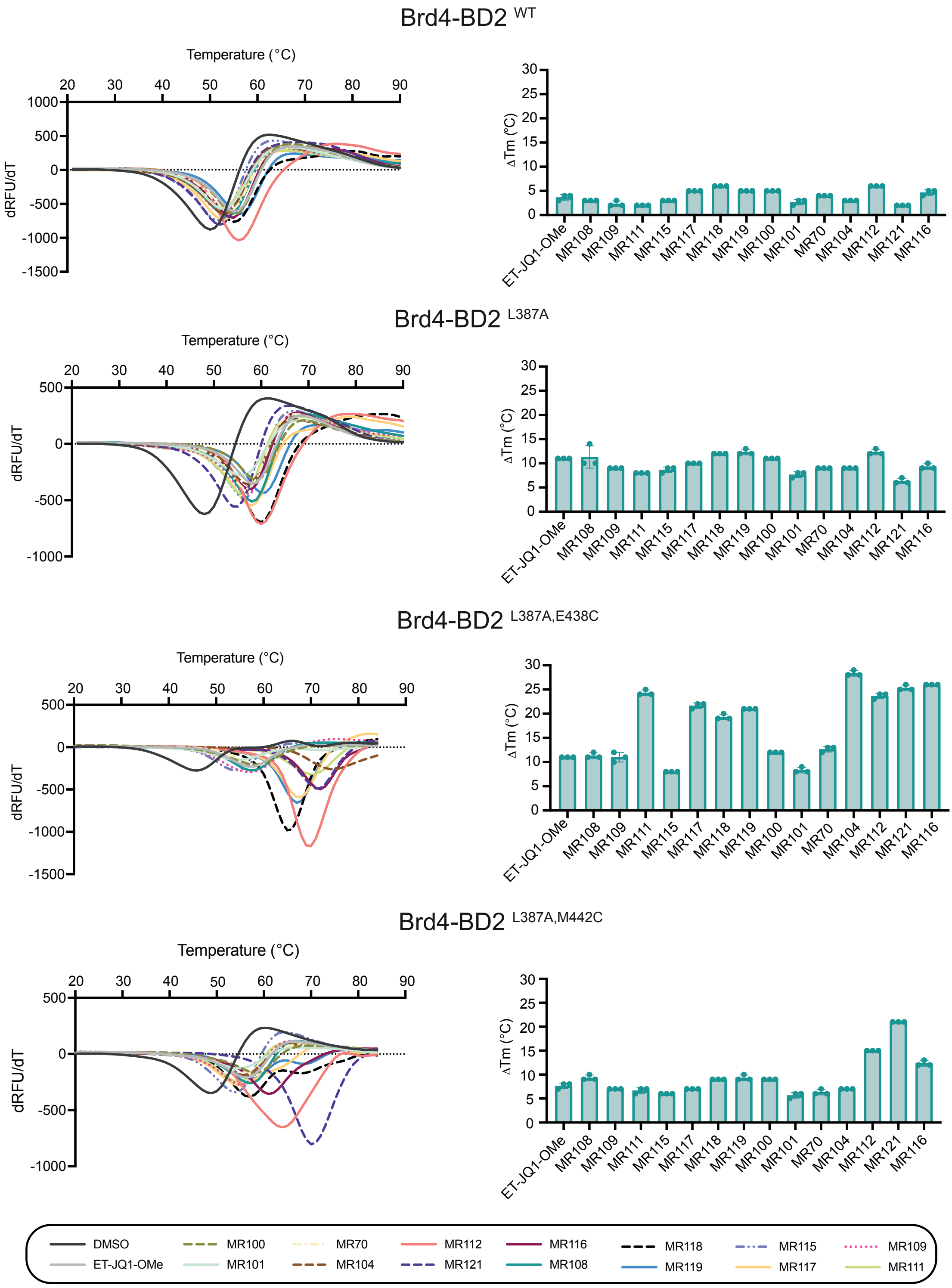

Supplementary Figure 3. Compounds screening by differential scanning fluorimetry. Monotoring protein-ligand binding by differential scanning fluorimetry (DFS) for (top to bottom): long Brd4-BD2^{WT}, long Brd4-BD2^{L387A} (BromoTag), short Brd4-BD2^{L387A,E438C} (BromoCatch), and short Brd4-BD2^{L387A,M442C}. Left: DSF plots for each protein mutant in the absence (DMSO, solid black line) and presence of compound (5:1 ligand:protein ratio) following pre-incubation at room temperature for 2 hours. The derivative of the fluorescent signal (dRF) is plotted against temperature. Right: Increments in melting temperatures ΔT_m (°C, thermal shift) for each ligand tested, relative to DMSO. The minimum of the dRFU/dT derivative plots identified the melting temperature T_m. Data are mean from N=3 technical replicates. RFU: Relative Fluorescent Units.

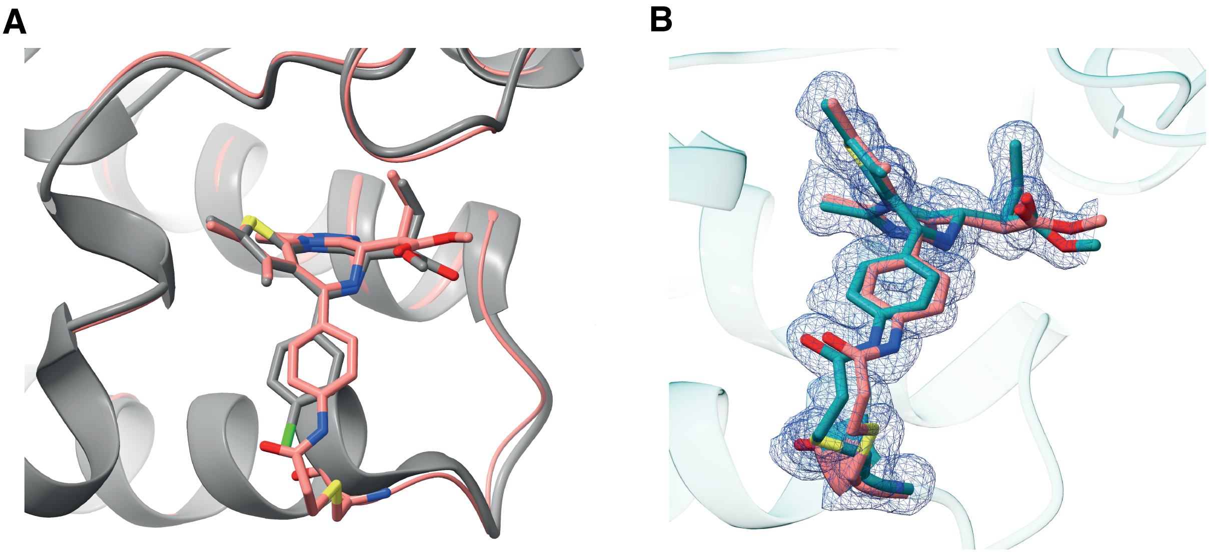

Supplementary Figure 4. The co-crystal structure of BromoCatch covalent adduct with MR116, solved to 1.3 Å of resolution. A) Overlay of the Brd2-BD2^{L383V}: ET-JQ1-OMe complex (6YTM) and rotamer 2 of the Brd2-BD2^{L383A,D434C} : MR116 complex (9QRK). The aromatic ring in rotamer 2 is slightly more shifted than rotamer 1 relative to 6YTM, with a calculated $RMSD_{CS}$ (root mean square deviation over common structure) of 1.13 Å. B) The electron density map (2Fo-Fc) superimposed around each of the two conformations of the bound MR116 ligand is shown in blue contoured at 1 σ.

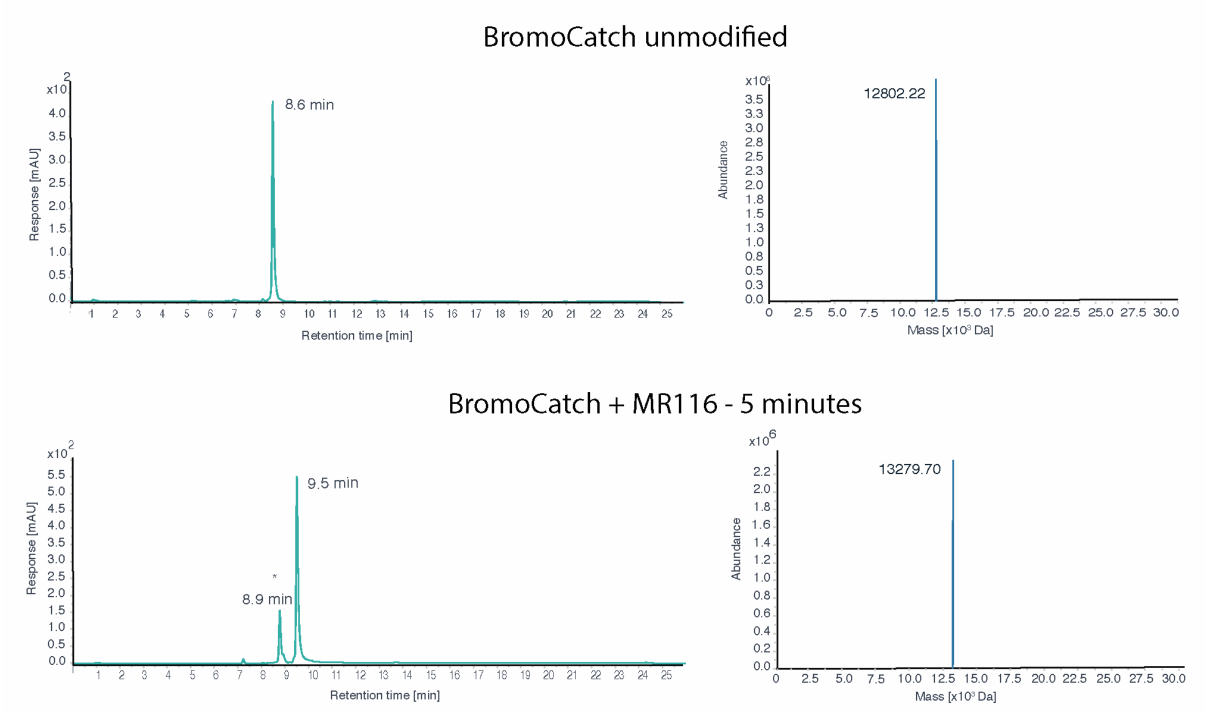

Supplementary Figure 5. Rapid kinetics of BromoCatch covalent adduct formation. BromoCatch protein (50 µM) is modified within 5 min of incubation with acrylamide ligand MR116 (100 µM). Intact MS analysis after 5 min incubation at a 2:1 (ligand:protein) shows full modification within 5 min of incubation. *no protein mass envelope detected (data is representative of one independent experiment; the experiment was repeated twice with similar results). Samples were run on an UHPLC Agilent 1290 Infinity III over 20 minutes on a zorbax 300 SB C3 21 x 150mm, 5µm column using a 10–95% gradient of acetonitrile 0.05 % TFA in water 0.05 % TFA and analysed using an Agilent 6135 single quadrupole MS, unless otherwise stated.

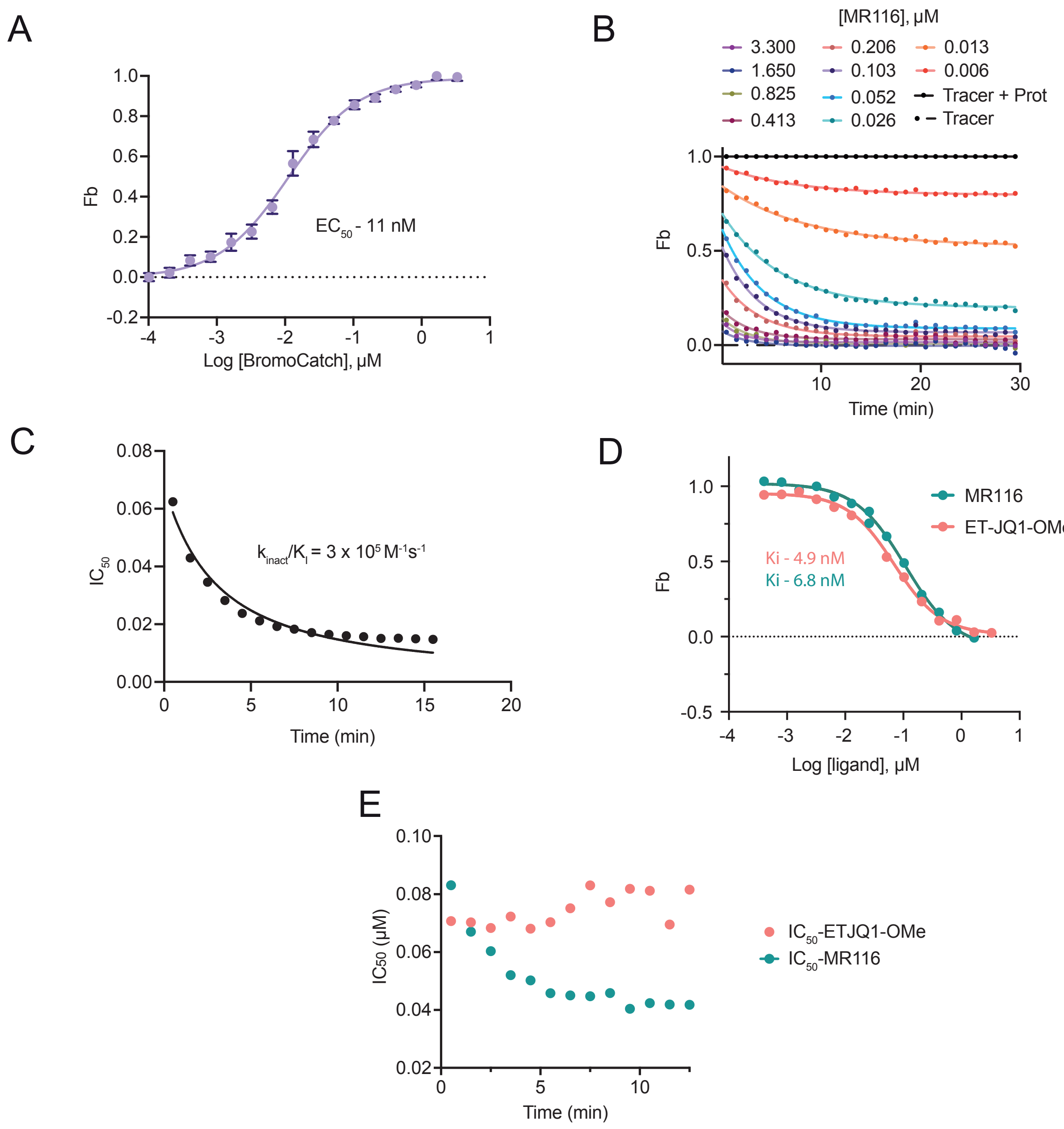

Supplementary Figure 6. Kinetics of covalent adduct formation by MR116. A) Titration curve of BromoCatch protein with the sulfoCy5-ET-JQ1 reversible tracer (MR133) used in the competition assay to determine tracer binding affinity (K_L). Data are average ± s.d. of N=3 technical replicates. B) Time dependent decrease in bound fraction (Fb) of tracer (10 nM) bound to the BromoCatch protein (30 nM) upon incubation at different concentrations of MR116. The observed time-dependent profile is consistent with covalent engagement of MR116. The decrease in anisotropy (shown as Fb) over time allows to obtain the k_{obs} for each concentration. Data are average of N=3 technical replicates. C) Calculation of k_{inact}/K_I from a plot of IC_{50} versus time using Krippendorff's equation as a validating approach of the Michaelis Menten fit. This is useful for potent inhibitors such as MR116 ($K_i \approx 6$ nM), where the k_{obs} at the low inhibitor concentrations (<<Ki) fall below the limit of detection of the assay set up. Data shown is average of N=3 technical replicates. D) Plos of Fb over ligand concentration for ET-JQ1-OMe and MR116 (at t = 0 min, to account for its reversible binding) evidences comparable profiles and K_i values. Data are average of N=2 technical replicates. E) Comparative time-dependent decrease in IC_{50} for the reversible binder ET-JQ1-OMe and MR116. Data shown are average of N=2 technical replicates.

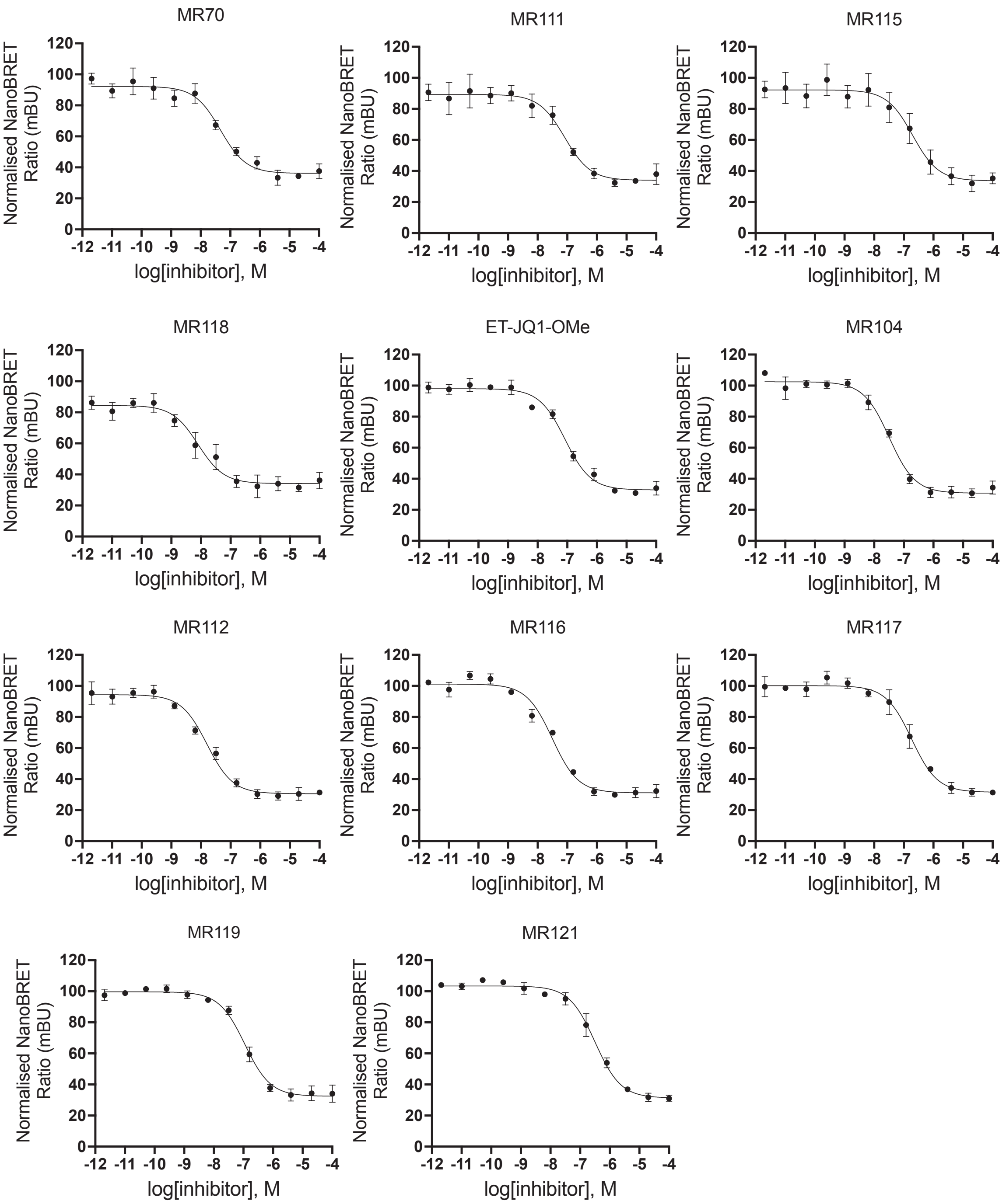

Supplementary Figure 7. Compound screening by nanoBRET cellular target engagement. Displacement of 1 μM ET-JQ1-PEG$_3$-BODIPY (MR141) bound to NanoLuc-tagged Brd4-BD2^{L387A,E438C} expressed in HEK293 cells upon increasing concentration of covalent ligands and ET-JQ1-OMe. Data is mean ± s.e.m. from N=3 independent experiments. Linear regression curve fitting of titrations was performed in Graphpad prism v10.3.2. as "log(inhibitor) vs. response (three parameters)" that were used to calulate pIC$_{50}$ and IC$_{50}$.

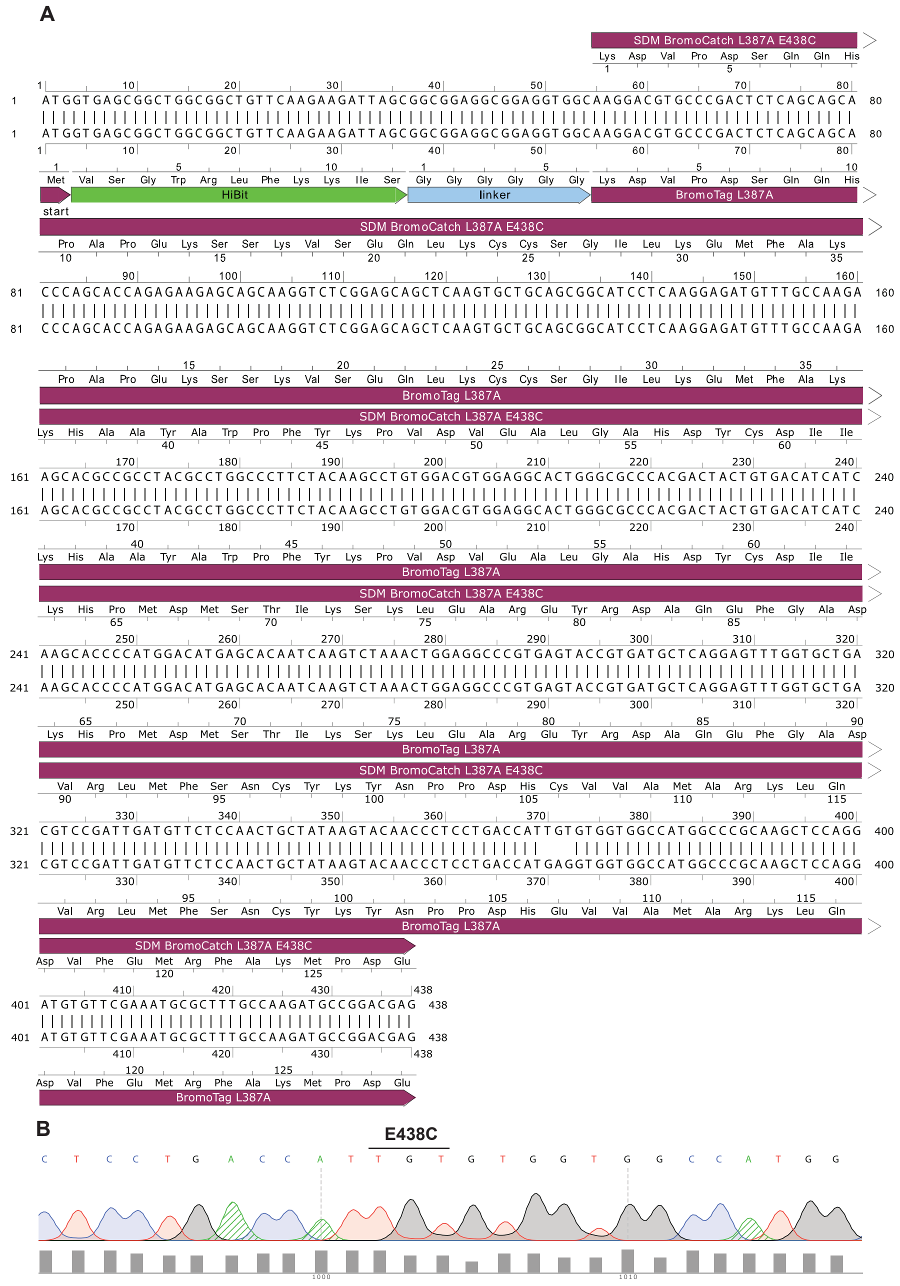

Supplementary Figure 8. Sequence confirmation of SDM-modified BromoTag^{L387A}–Brd4 donor to BromoCatch^{L387A,E438C}–Brd4. (A) Alignment of pEGFP-C1 forward primer–driven Sanger sequencing read from plasmid DNA obtained following site-directed mutagenesis of the eGFP-IRES-HiBiT-BromoTag^{L387A} pMA-RQ donor plasmid, compared with the original reference sequence. The alignment, performed using a Smith–Waterman algorithm in SnapGene v8.0.2, confirms the presence of the E438C mutation in BromoCatch. Top strand is a sequencing read of a post SDM modified pMA-RQ containing the eGFP-IRES-BromoTag^{L387A}-Brd4 donor. (B) Representative chromatogram of the SDM-modified eGFP-IRES-HiBiT-BromoTag pMA-RQ construct, demonstrating an unambiguous substitution of glutamate (E) to cysteine (C) at position 438. Chromatogram analysis was performed in SnapGene v8.0.2. This analysis was performed N=1.

Parental Wild type HEK293

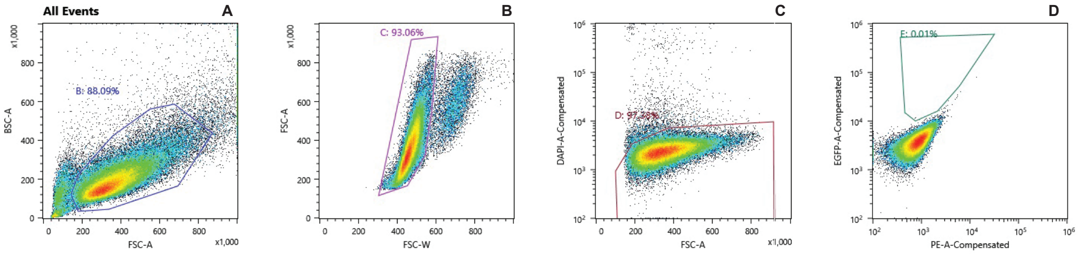

Gates and Statistics

Name	Events	%Parent	%Total
All Events	100,000	0.00%	100.00%
B	88,086	88.09%	88.09%
C	81,970	93.06%	81.97%
D	79,819	97.38%	79.82%
E	4	0.01%	0.00%

eGFP-IRES-HiBiT-BromoCatch-Brd4 transfected HEK293

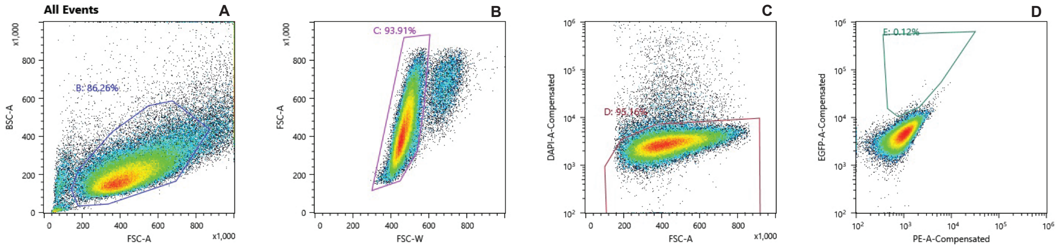

Gates and Statistics

Name	Events	%Parent	%Total
All Events	100,000	0.00%	100.00%
B	86,256	86.26%	86.26%
C	81,007	93.91%	81.01%
D	77,088	95.16%	77.09%
E	89	0.12%	0.09%

Supplementary Figure 9. Fluorescence activated cell sorting (FACS) gating strategy for knock-in of eGFP-IRES-HiBiT-BromoCatch at the N-terminus of Brd4 in HEK293 cells. Going from the left to right hand panels: (A) Parental and BromoCatch donor–transfected cells were first distinguished from debris based on forward scatter area (FSC-A) versus side scatter area (SSC-A). (B) Single cells were then identified by gating on FSC width (FSC-W) versus FSC-A. (C) Live cells were selected based on exclusion of DAPI-positive events (DAPI-A). (D) The parental, non-GFP-expressing control was subsequently used to define cellular autofluorescence using GFP-A versus PE-A (585 nm) parameters. This gate was then applied to identify GFP-positive cells in the BromoCatch-transfected population, which were subsequently sorted into individual wells of a 96 well plate. FACS was performed using an SH800 cell sorter (Sony Biotechnology). FACS sorting was performed N=1.

A

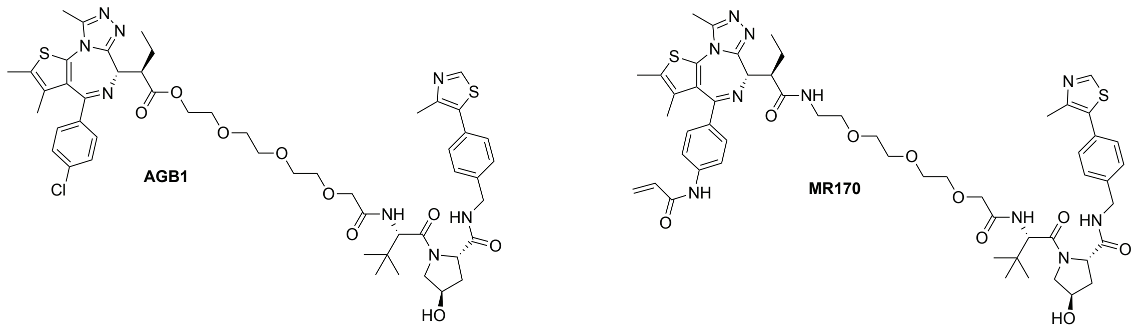

B

	Relative luminescence (normalised to parental line)
Parental line	1
Clone 1	1939.5
Clone 2	25.5
Clone 3	1246.9
Clone 4	20.5
Clone 5	17.5
Clone 6	1583.8
Clone 7	1616.8
Clone 8	956.4
Clone 9	490.3
Clone 10	388.2
Clone 11	642.7
Clone 12	1850.1
Clone 13	1106.0
Clone 14	1822.7
Clone 15	633.6

C

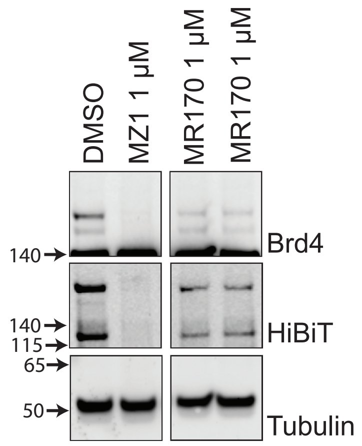

Supplementary Figure 10. Chemical structures and degradation activity of PROTAC MR170. (A) Chemical structures of PROTACs AGB1 and MR170 used to assess degradation activity against BromoCatch in an endogenous HiBiT-BromoCatch-Brd4 HEK293 cell line. (B) Fold change in luminescence of single-cell clones produced from the FACS sort (Supplementary Figure 9) of transfected HiBiT-BromoCatch-Brd4 compared to a wild-type parental control, measured using the Promega HiBiT lytic assay and used to identify endogenously tagged clones. N=1 biological replicate. Raw data can be found in the Source Data File. (C) Immunoblot validation of BromoCatch knock-in at the Brd4 locus following treatment with 1 µM MZ1 (positive control), DMSO (vehicle control), or MR170 for 6 hours prior to lysis. Western blots were acquired using a Bio-Rad ChemiDoc™ MP imaging system and processed using Image Lab v6.1.0. Immunoblot was performed as a N=1 biological experiment.

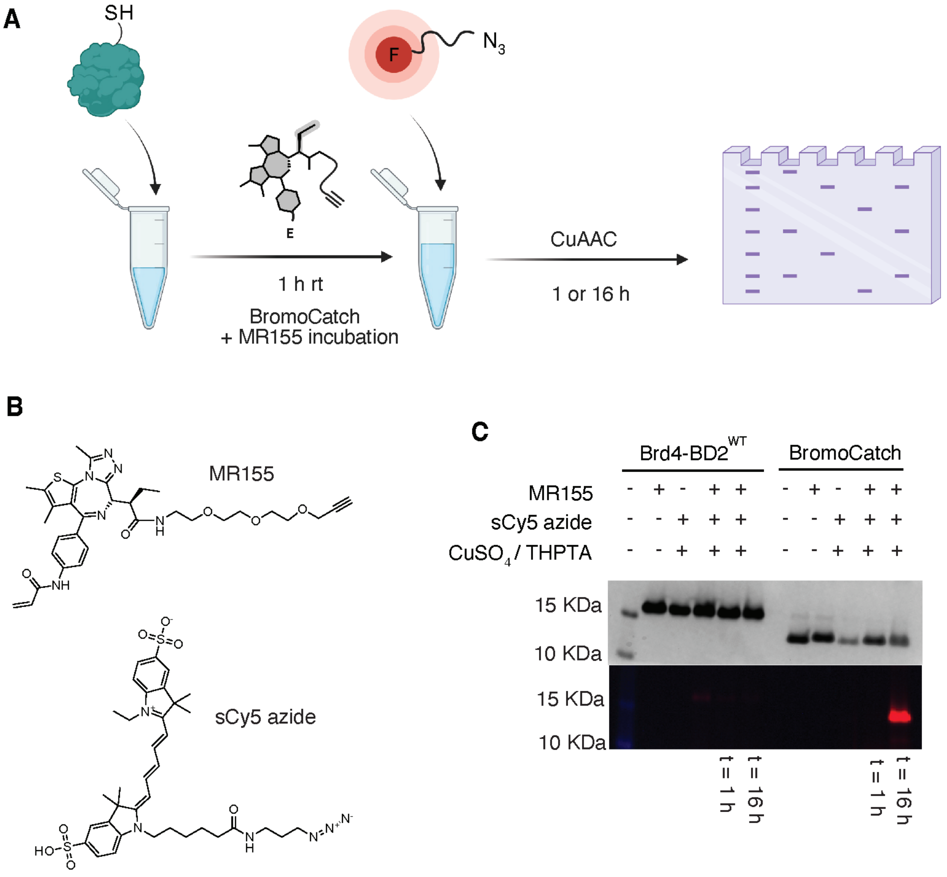

Supplementary Figure 11. BromoCatch can be used for clicking fluorophores in a two-step CuAAC mediated reaction. A) Workflow for the CuAAC experiment. Illustration generated with Biorender.com (https://BioRender.com/rn4pgs8) B) The alkyne bearing probe MR155 (1.5 eq) was pre-incubated with BromoCatch or Brd4-BD2^{WT} (purified protein) for 1 h before adding azide sulfonated Cy5 (excess) and the CuAAC reagents ($CuSO_4$/THTPA/NaAsc) and samples were taken at 1 h and 16 h and analysed by SDS-PAGE. The conjugation selectively happens for BromoCatch, with no labelling observed for the Brd4-BD2^{WT} protein. Data is representative of one independent experiment; the experiment was repeated twice with similar results.

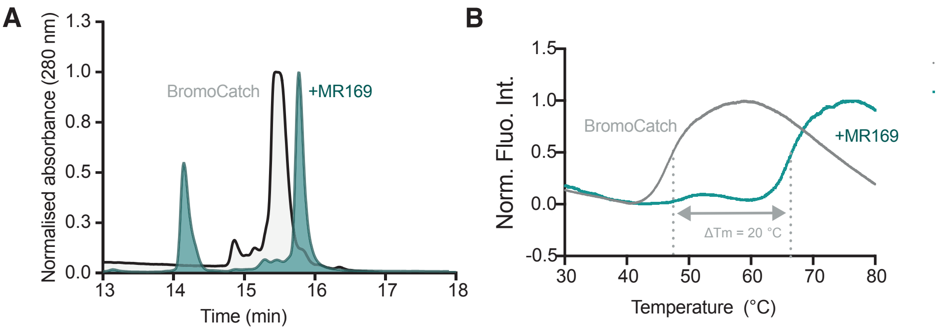

Supplementary Figure 12. MR169 biotin probe *in vitro* validation. A) HPLC-UV (280 nm) showed full covalent modification of BromoCatch when incubated in a 2:1 ratio (ligand:protein) for 2 hours at room temperature. Data is representative of one independent experiment; the experiment was repeated twice with similar results. B) The significant protein stabilisation effect was measured by nanoDSF and

confirmed to be maintained for the biotin probe MR169. Data is representative of one independent experiment; the experiment was repeated twice with similar results.

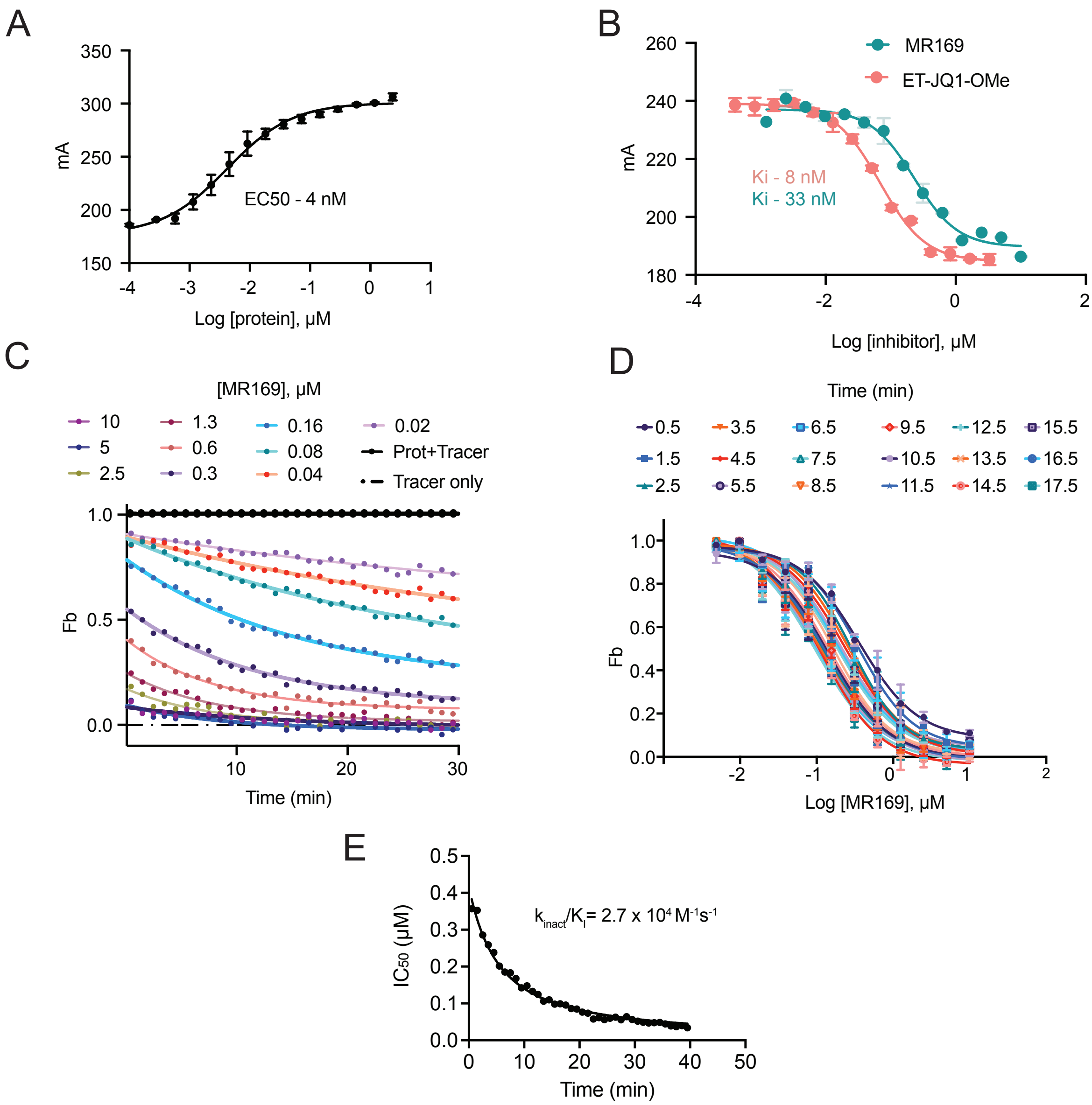

Supplementary Figure 13. Extended data on MR169 kinetics. A) Titration of sulfoCy5 reversible tracer MR133 (data are average ± s.d. from N=3 technical replicates). B) Comparative of ET-JQ1-OMe K_i with the K_i at t=0 for MR169 extracted from Y_0 intercepts from the time dependent Fb decrease plot (data are mean from N=2 technical replicates). C) The decrease in anisotropy (shown as Fb) over time allows to obtain the k_{obs} for each concentration, shown in Figure 7D in the main manuscript (data are calculated values from N=3 technical replicates). D) Time-dependent decrease of binding affinity (IC_{50}) observed for covalent MR169 (data are average ± s.d. from N=3 technical replicates). E) IC_{50} decrease over time was monitored over 40 min and calculation of k_{inact}/K_I using the Krippendorff's equation. Data shown are mean from N=3 technical replicates, and the quoted k_{inact}/K_I value is from data fitting of this N=1.

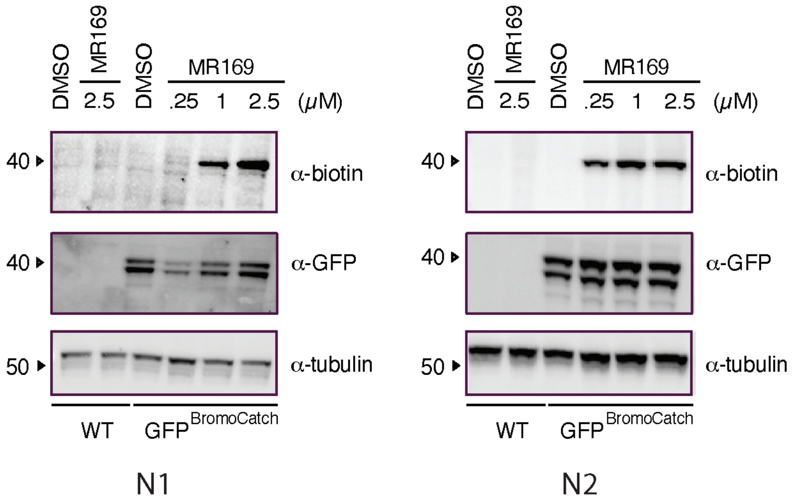

Supplementary Figure 14. HEK293 FT cells overexpressing BromoCatch-labelled GFP with increasing concentrations of MR169. N=2 biological repeats.

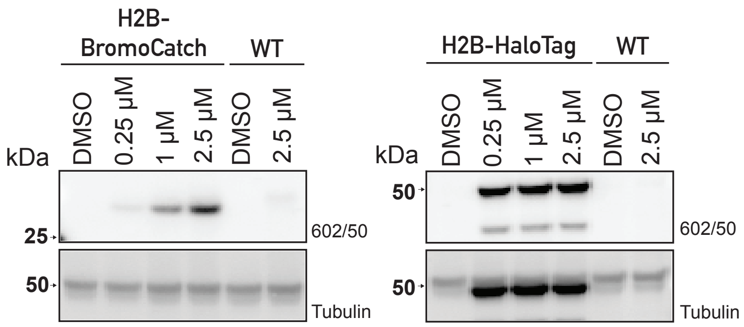

Supplementary Figure 15. Ex-cellulo comparison of BromoCatch and HaloTag TMR labelling in stably transfected H2B-BromoCatch and H2B-HaloTag U2OS cells by immunoblotting. Stably transfected H2B-BromoCatch and H2B-HaloTag U2OS cells were plated and treated with 0.25, 1, and 2.5 µM MR202 or HaloTag TMR probe for 2 hours prior to harvest. Mock-transfected cells were treated with 2.5 µM of the corresponding probe to assess selectivity. 20 µg of lysate were loaded per lane and resolved on a 4–12% SDS-PAGE gel in 1× MOPS buffer, transferred to nitrocellulose, and imaged using a 602–650 nm emission filter on a Bio-Rad imager. Rhodamine one-step anti-tubulin secondary antibody was used for loading control assessment. N=3 independent blots.

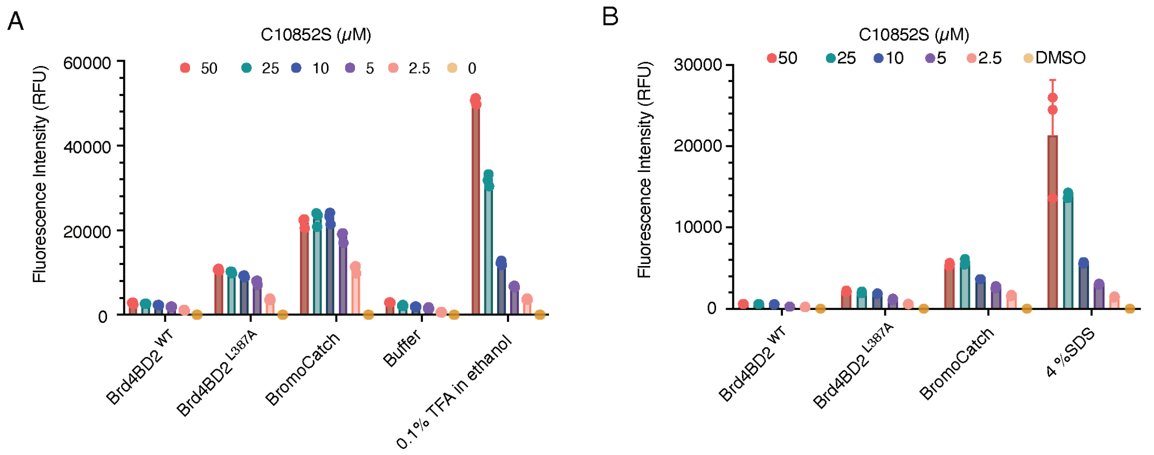

Supplementary Figure 16. Fluorogenic switch on with the C10852S probe. Positive controls 0.1% TFA in ethanol or 4% SDS are included. A) Extended data shows the fluorogenic response in presence of the different proteins and including the positive control of 0.1% TFA in ethanol. Interestingly, the maximal fluorescence emission (RFU) at 10 µM of C10852S in acidic conditions is slightly lower than the maximal achieved upon binding BromoCatch. Data are average ± s.d. from N=3 technical replicates. B) Fluorogenic response in presence of the different proteins and including the positive control of 4% SDS in activity buffer, that serves as a more efficient positive control for full activation of C10852S. Data are average ± s.d. from N=3 technical replicates.

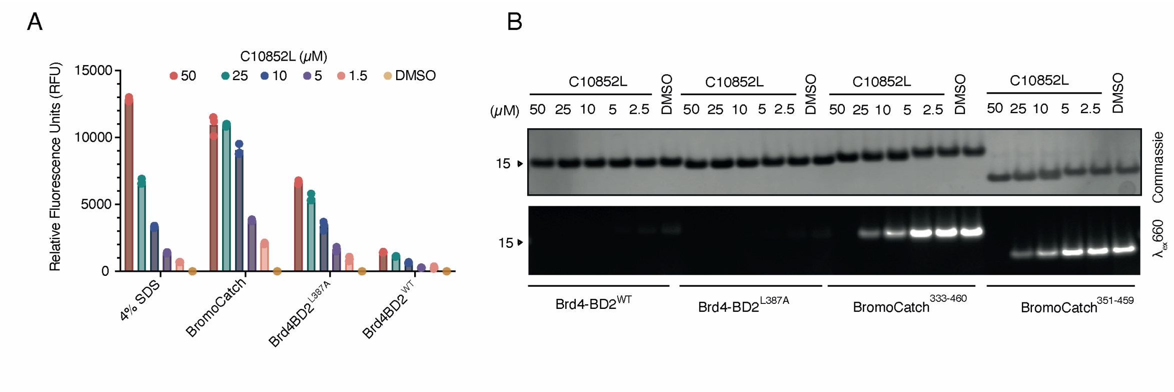

Supplementary Figure 17. Fluorescent labelling of BromoCatch C10852L probe including positive control. A) Extended data shows the fluorogenic response in presence of the different proteins and including the positive control of 4% SDS. The increasing concentrations the C10852L probe in presence if BromoCatch (10 µM) correlated with the increase of fluorescence as it was observed for C10852S. Data are average ± s.d. from N=3 technical replicates. None of the two positive controls (4% SDS, panel A or 0.1% TFA in ethanol) were able to achieve full activation of the C10852L probe. B) SDS gel analysis of the samples after 1 hour incubation. Data is representative of one independent experiment. The experiment was repeated twice with similar results.

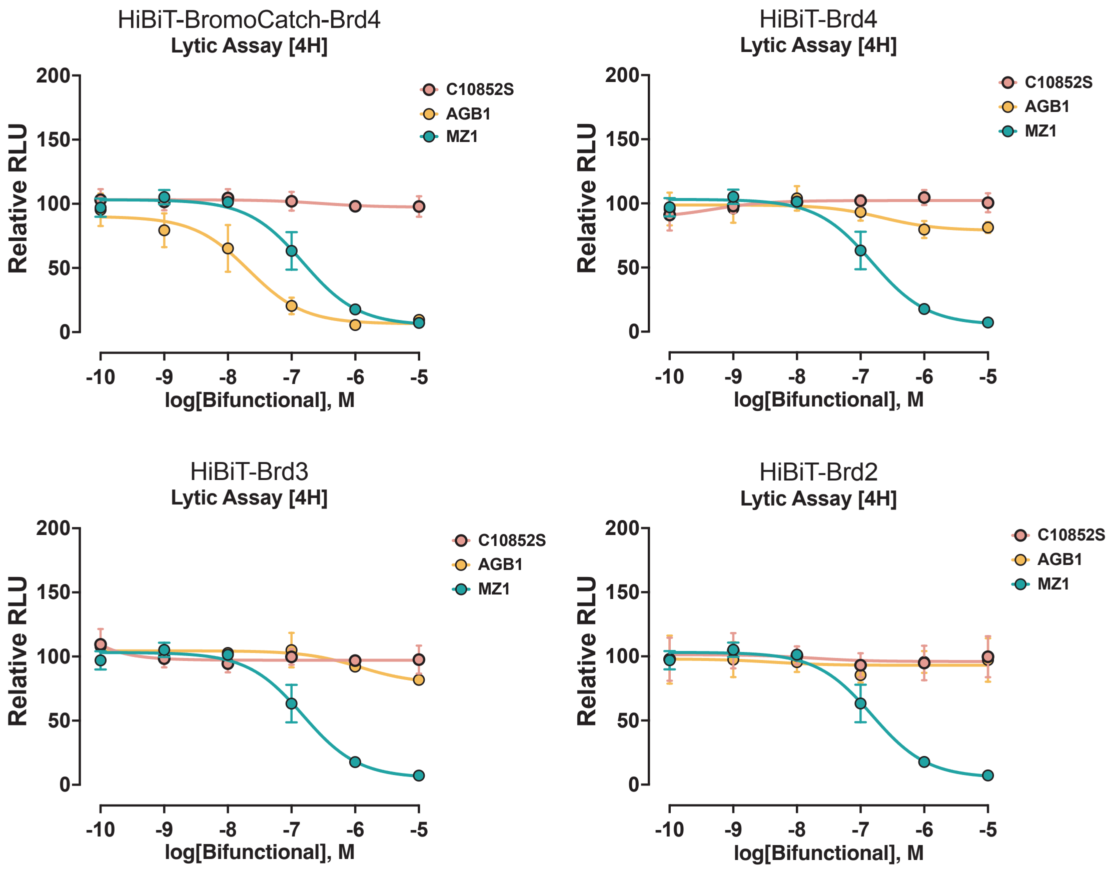

Supplementary Figure 18. BromoCatch bifunctional probe does not degrade BromoCatch-tagged or endogenous BET protein. HiBiT lytic degradation of HiBiT-BromoCatch-Brd4 and HiBiT tagged Brd4, Brd3 and Brd2 using C10852S, MZ1 and AGB1 at 10 µM-100 pM. Cells were incubated with compounds for 4 hours prior to addition of HiBiT lytic reagent and subsequent imaging on a BMG Labtech PHERAstar luminescence plate reader. Plots created in Graphpad Prism Version 10.2.3 using log(inhibitor) vs. response (three parameters). Data are mean ± s.e.m. from N=3 independent repeats.

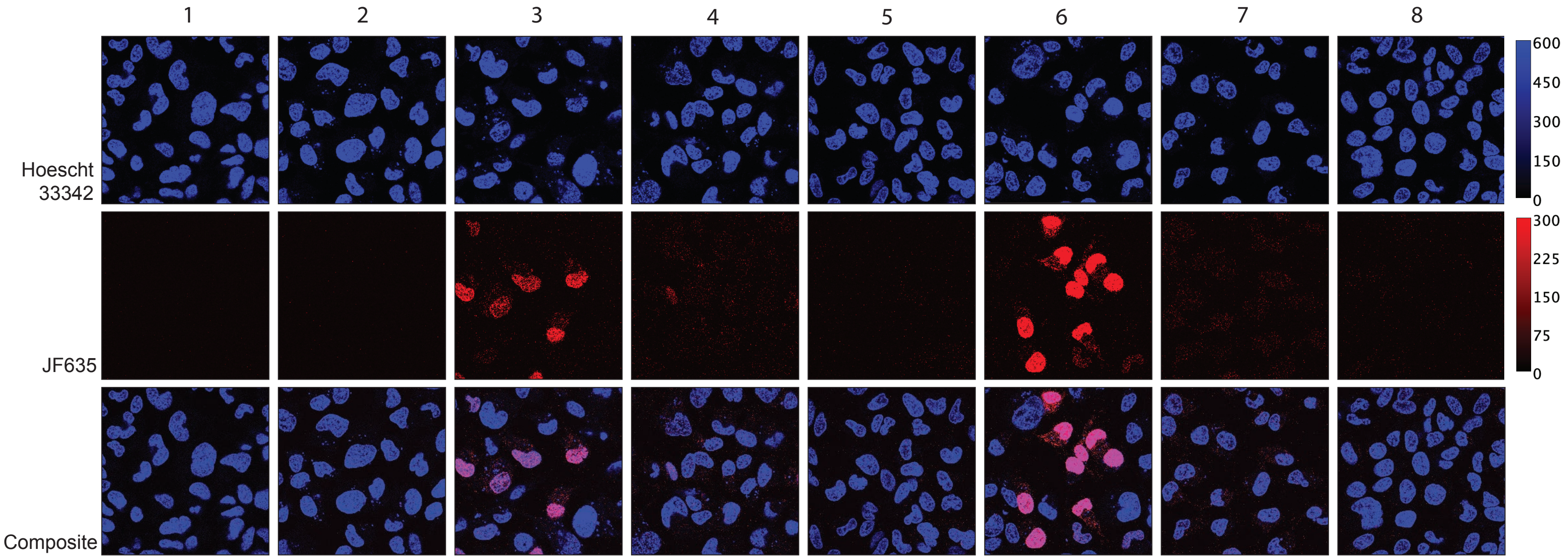

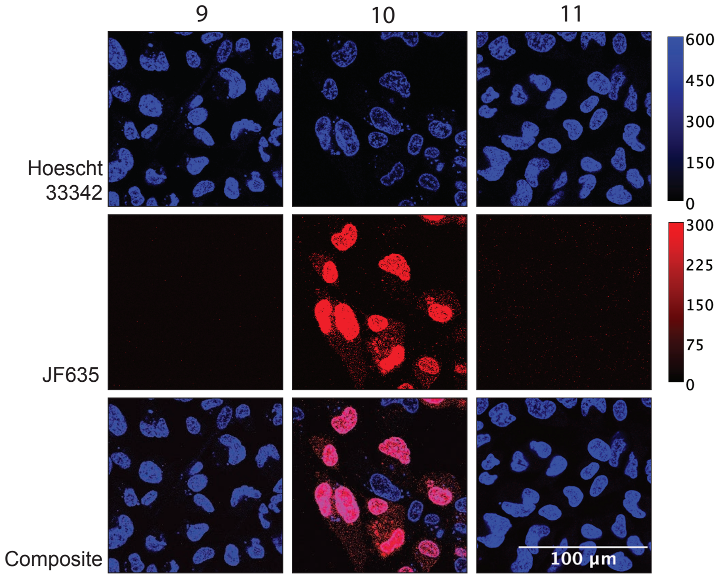

Independent Repeat 1

4-hour treatment

1. Vehicle-treated mock-transfected U2OS
2. Vehicle-treated H2B-BromoCatch U2OS
3. C10852S (1 µM)-treated H2B-BromoCatch U2OS
4. C10852S (1 µM) + ET-JQ1-OMe (25 µM)-treated H2B-BromoCatch U2OS
5. C10852S (1 µM)-treated mock-transfected U2OS
6. C10852L (0.5 µM)-treated H2B-BromoCatch U2OS
7. C10852L (0.5 µM) + ET-JQ1-OMe (25 µM)-treated H2B-BromoCatch U2OS
8. C10852L (0.5 µM)-treated mock-transfected U2OS
9. Vehicle-treated H2B-HaloTag U2OS
10. JF635 Haloalkane (100 nM)-treated H2B-HaloTag U2OS
11. JF635 Haloalkane (100 nM)-treated mock-transfected U2OS

Representative images of 2 technical Z-stack images

The scale bar, shown in the final panel represents the scaling in all panels. Channels were scaled independently, but settings were kept consistent across conditions within each channel; the calibration scales are therefore representative of all panels shown.

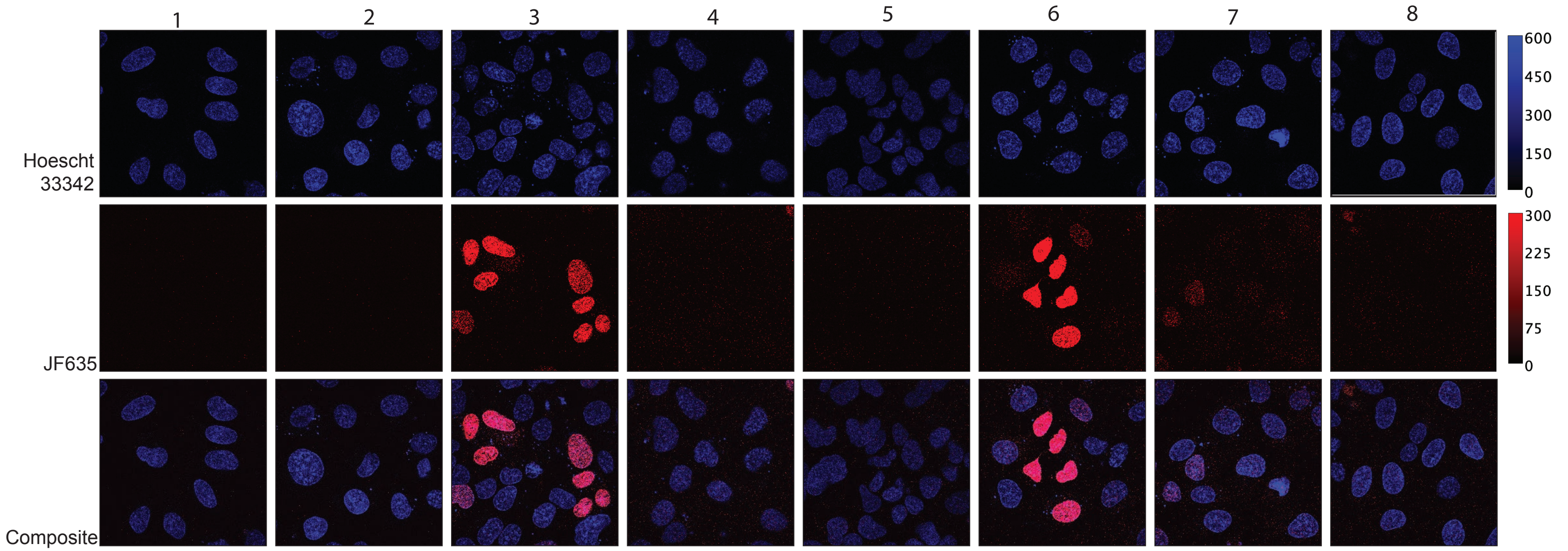

Independent Repeat 2

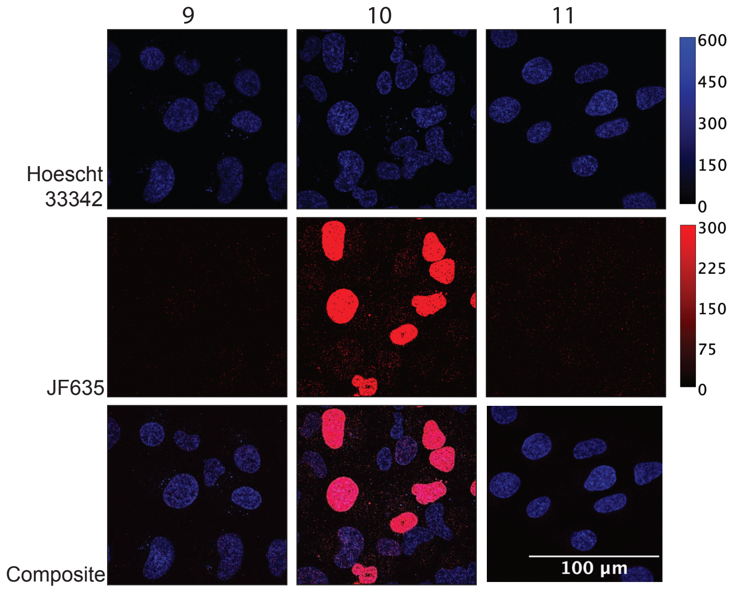

4-hour treatment

1. Vehicle-treated mock-transfected U2OS
2. Vehicle-treated H2B-BromoCatch U2OS
3. C10852S (1 µM)-treated H2B-BromoCatch U2OS
4. C10852S (1 µM) + ET-JQ1-OMe (25 µM)-treated H2B-BromoCatch U2OS
5. C10852S (1 µM)-treated mock-transfected U2OS
6. C10852L (0.5 µM)-treated H2B-BromoCatch U2OS
7. C10852L (0.5 µM) + ET-JQ1-OMe (25 µM)-treated H2B-BromoCatch U2OS
8. C10852L (0.5 µM)-treated mock-transfected U2OS
9. Vehicle-treated H2B-HaloTag U2OS
10. JF635 Haloalkane (100 nM)-treated H2B-HaloTag U2OS
11. JF635 Haloalkane (100 nM)-treated mock-transfected U2OS

Representative images of 2 technical Z-stack images

The scale bar, shown in the final panel represents the scaling in all panels.

Channels were scaled independently, but settings were kept consistent across conditions within each channel; the calibration scales are therefore representative of all panels shown.

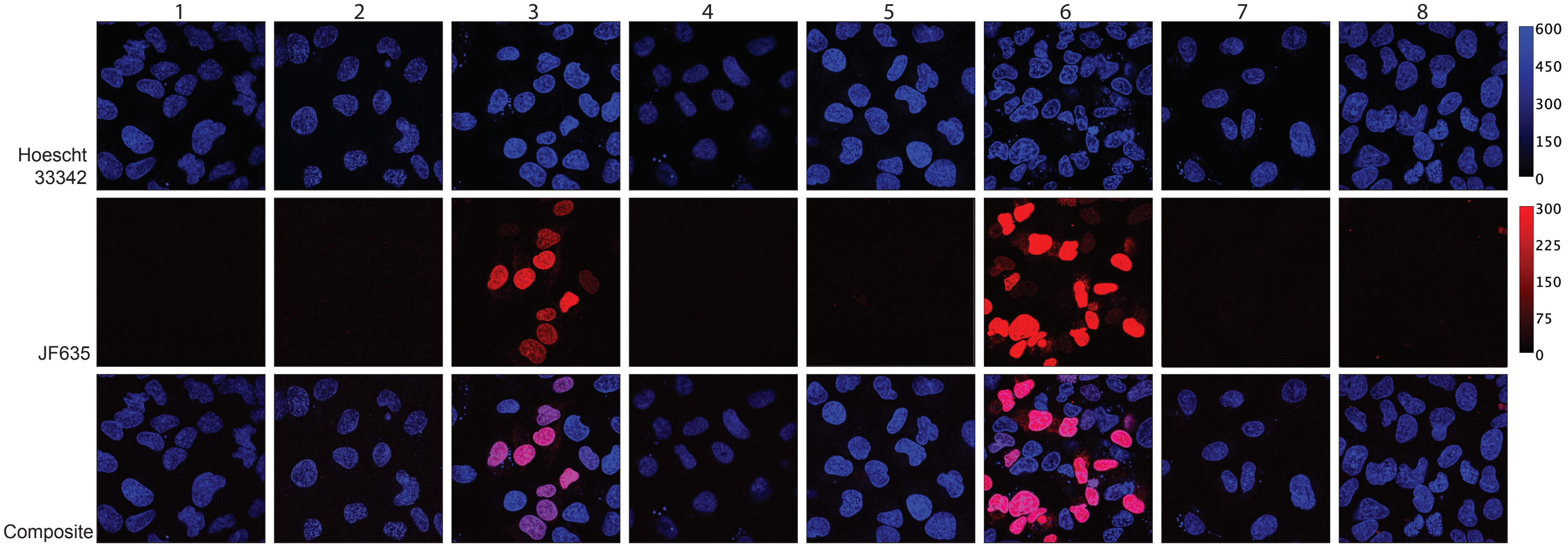

Independent Repeat 3

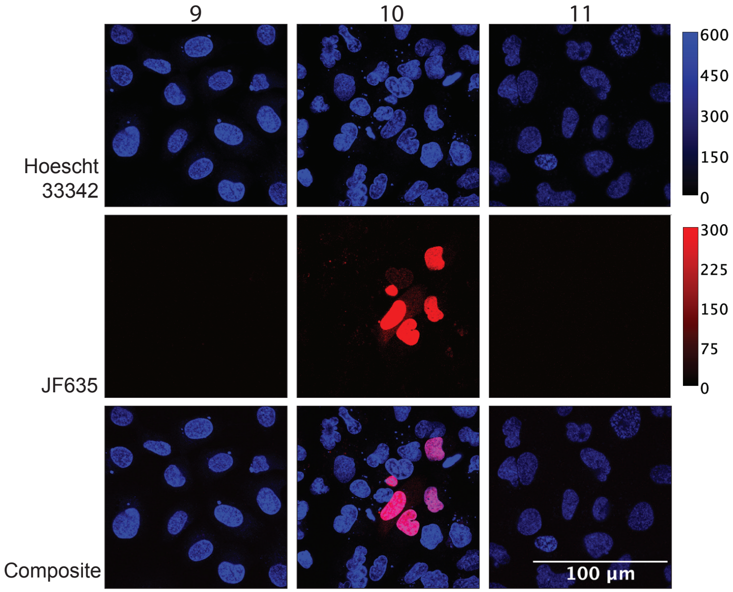

4-hour treatment

1. Vehicle-treated mock-transfected U2OS
2. Vehicle-treated H2B-BromoCatch U2OS
3. C10852S (1 µM)-treated H2B-BromoCatch U2OS
4. C10852S (1 µM) + ET-JQ1-OMe (25 µM)-treated H2B-BromoCatch U2OS
5. C10852S (1 µM)-treated mock-transfected U2OS
6. C10852L (0.5 µM)-treated H2B-BromoCatch U2OS
7. C10852L (0.5 µM) + ET-JQ1-OMe (25 µM)-treated H2B-BromoCatch U2OS
8. C10852L (0.5 µM)-treated mock-transfected U2OS
9. Vehicle-treated H2B-HaloTag U2OS
10. JF635 Haloalkane (100 nM)-treated H2B-HaloTag U2OS
11. JF635 Haloalkane (100 nM)-treated mock-transfected U2OS

Representative images of 2 technical Z-stack images

The scale bar, shown in the final panel represents the scaling in all panels.

Channels were scaled independently, but settings were kept consistent across conditions within each channel; the calibration scales are therefore representative of all panels shown.

Supplementary Figure 19. Live-cell z-stack confocal imaging of C10852S (1 µM) or C10852L (500 nM) in H2B-BromoCatch–transfected U2OS cells and JF635 (100 nM) Haloalkane labelling in H2B-HaloTag–transfected U2OS cells. Live-cell confocal imaging was performed on U2OS cells expressing H2B-BromoCatch(351-460) to assess probe activation following 4 hour treatment with DMSO (vehicle), C10852S (1 µM), or C10852L (500 nM). In parallel, U2OS cells expressing H2B-HaloTag were incubated with 100 nM JF635 Haloalkane for 4 hours to serve as a reference self-labeling tag system for direct comparison with BromoCatch. Hoechst 33342 (blue channel) was used as a nuclear counterstain. Fluorescence emission at ~650 nm was collected as the readout for "switch-on" activation of the probes (red channel), enabling selective visualisation of probe engagement with H2B-BromoCatch or H2B-HaloTag. Our intensity scaling for our images were was set for best display and consistently used on all displayed images. Images were captured as a Z-stack and subsequently presented as individual z-slices through the centre of the focal plane here for better visual representation. Controls included: (i) vehicle treated cells, (ii) 30 minute pre-treatment with ET-JQ1-OMe (25 µM) prior to probe treatment to block BromoCatch engagement and confirm target-dependent activation, and (iii) mock-transfected cells treated with each probe to assess non-specific background fluorescence. Image panels shown are representatives of two technical replicates per condition per independent experiment. A total of N=3 independent biological repeats were performed. All microscopy images were processed in FIJI v1.54p.

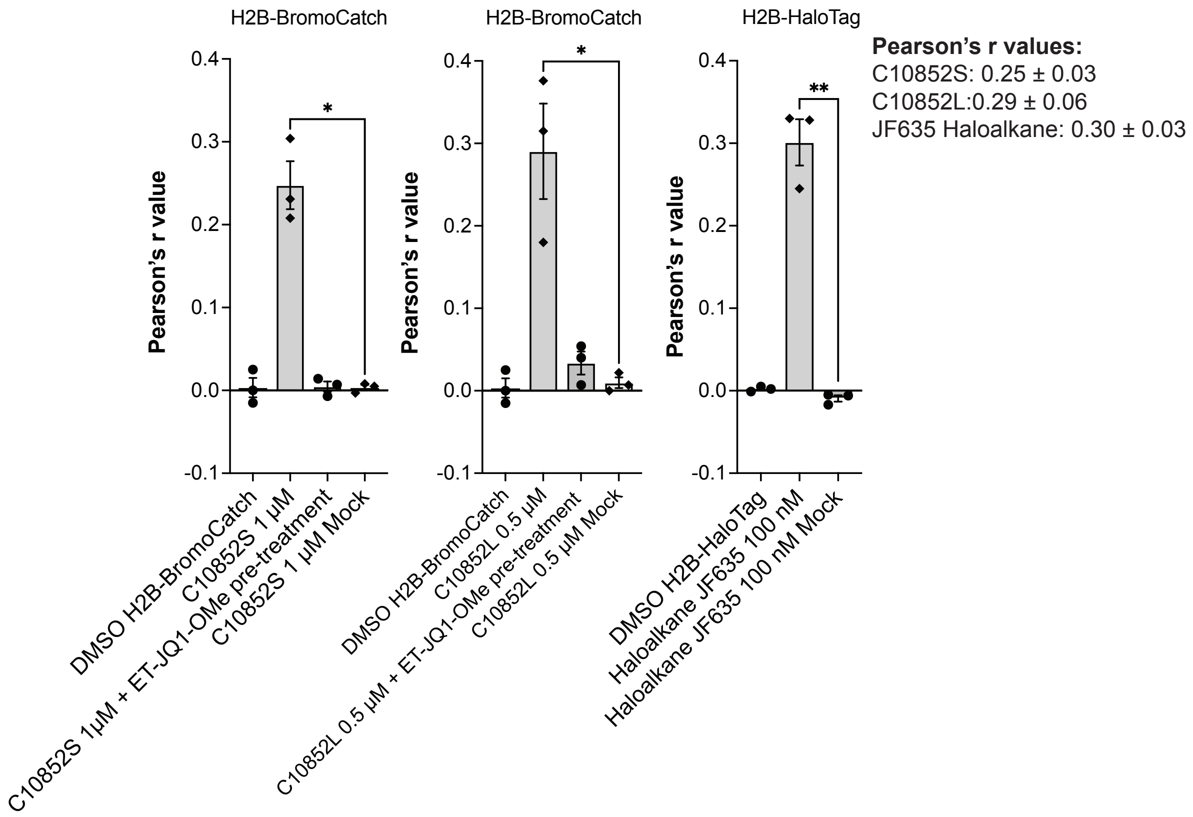

Supplementary Figure 20. Colocalisation analysis of BromoCatch probe activation. Individual z-slices centred on the middle of nuclei with optimal focus were selected for each channel and analysed using the JaCoP plugin in Fiji v1.54p to calculate Pearson's r values with Costes automatic thresholding. P-values from unpaired t.test with welch's correction for C10852S 1 µM treatment in transfected U2OS vs C10852S 1 µM mock transfection conditions is 0.01, C10852L 0.5 µM treatment transfected U2OS vs C10852L 0.5 µM mock transfection is 0.04, and for JF635 Haloalkane 100 nM treatment transfected U2OS vs JF635 Haloalakne 100 nM mock treatment is 0.007. Pearson correlation coefficients from two technical replicates were averaged to generate a single value per independent repeat, and averages from three independent repeats were plotted in GraphPad Prism v10.4.1. Data were

derived from the imaging experiments shown in Figure 10A (main text) and Supplementary Figure 19. A total of N=3 independent biological replicates were performed. Bar charts represent the mean values, with all individual replicate data points displayed. Error bars indicate the standard error of the mean (s.e.m).

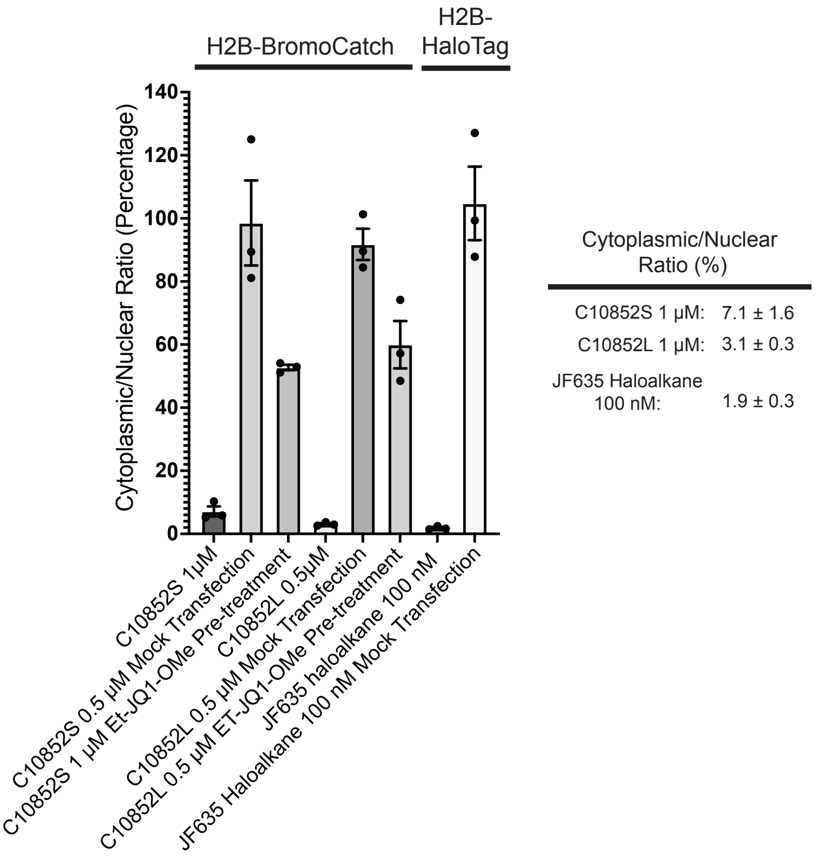

Supplementary Figure 21. Cytoplasmic vs nuclear ratio fluorescence analysis. Z-stacks were split into single channels (Hoechst and JF635) and converted into summed z-projections. Nuclear ROIs were defined using Hoechst 33342, and corresponding JF635 channel intensities were measured. Bright regions outside of the nuclei in the JF635 channel were used as surrogate cytoplasmic ROIs. Four nuclear and cytoplasmic ROIs were captured per field, with two fields analysed per condition for each independent repeat. Relative cytoplasmic fluorescence was calculated as the cytoplasmic intensity divided by the nuclear intensity for each cytopmasic and nuclear pairing and then this ratio was average for the 4 per image, and expressed as the average ratio percentage per image. Two technical replicates were averaged to yield a single value per independent repeat. This analysis was performed for C10852L, C10852S, and JF635 Haloalkane, as well as mock-transfected controls, with averages from three independent repeats plotted in GraphPad Prism v10.4.1. Data were derived from the imaging experiments shown in Figure 10A (main text) and Supplementary Figure 19. A total of N=3 independent biological replicates were performed. Bar charts represent the mean values, with all individual replicate data points displayed. Error bars indicate the standard error of the mean (s.e.m.).

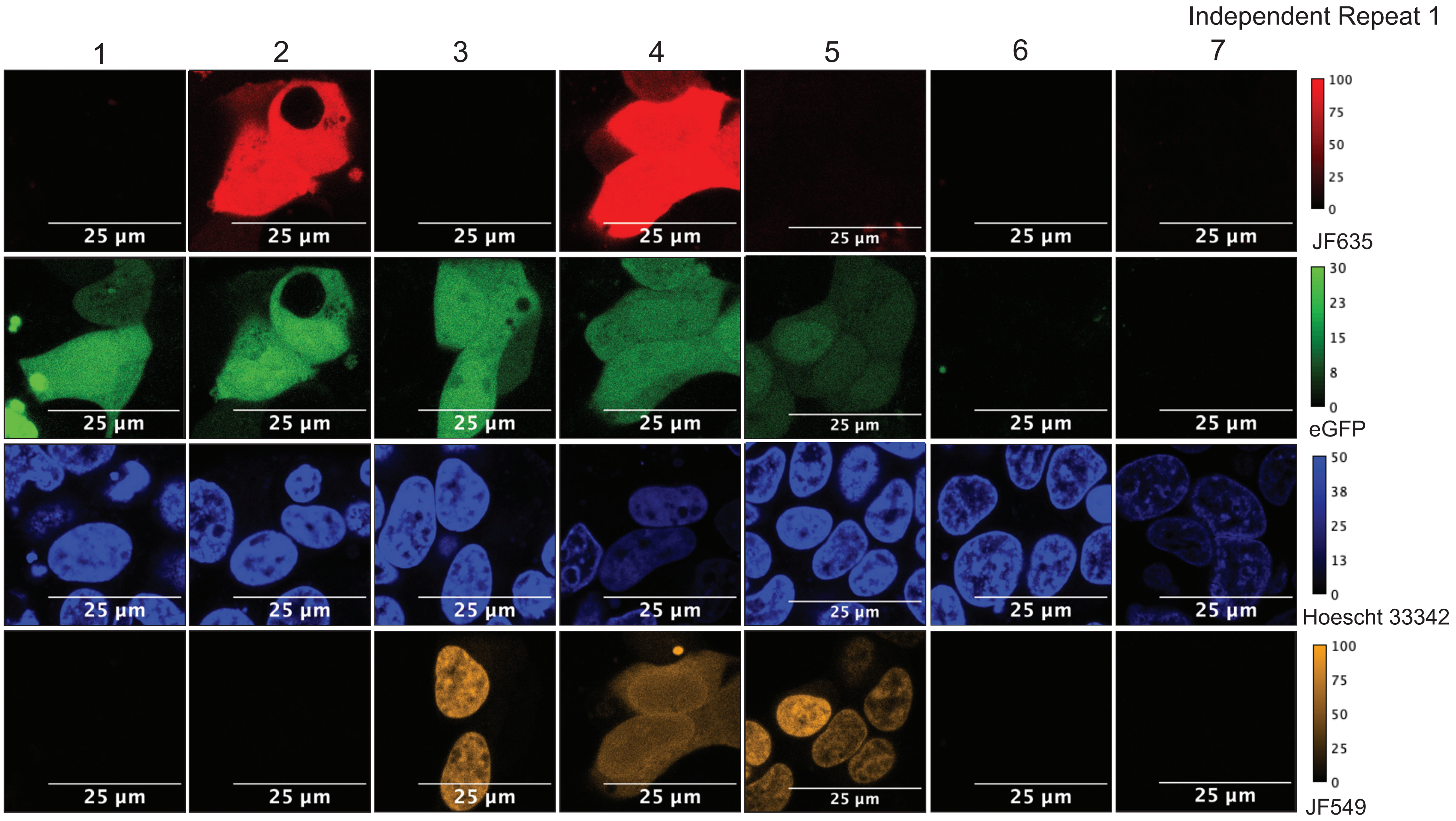

2 hour treatment

Representative blots of 3 technical repeats

1. Vehicle-treated GFP–BromoCatch / H2B–HaloTag HEK293 cells
2. C10852L (1 μM)–treated GFP–BromoCatch / H2B–HaloTag HEK293 cells
3. JF549 Haloalkane (100 nM)–treated GFP–BromoCatch / H2B–HaloTag HEK293 cells
4. C10852L (1 μM) + JF549 Haloalkane (100 nM)–treated GFP–BromoCatch / H2B–HaloTag HEK293 cells
5. C10852L (1 μM) + JF549 Haloalkane (100 nM) + ET-JQ1-OMe (25 μM, 30-min pre-treatment) GFP–BromoCatch / H2B–HaloTag HEK293 cells
6. Vehicle-treated mock-transfected HEK293 cells
7. C10852L (1 μM) + JF549 Haloalkane (100 nM)–treated mock-transfected HEK293 cells

Channels were scaled independently, but settings were kept consistent across conditions within each channel; the calibration scales are therefore representative of all panels shown.

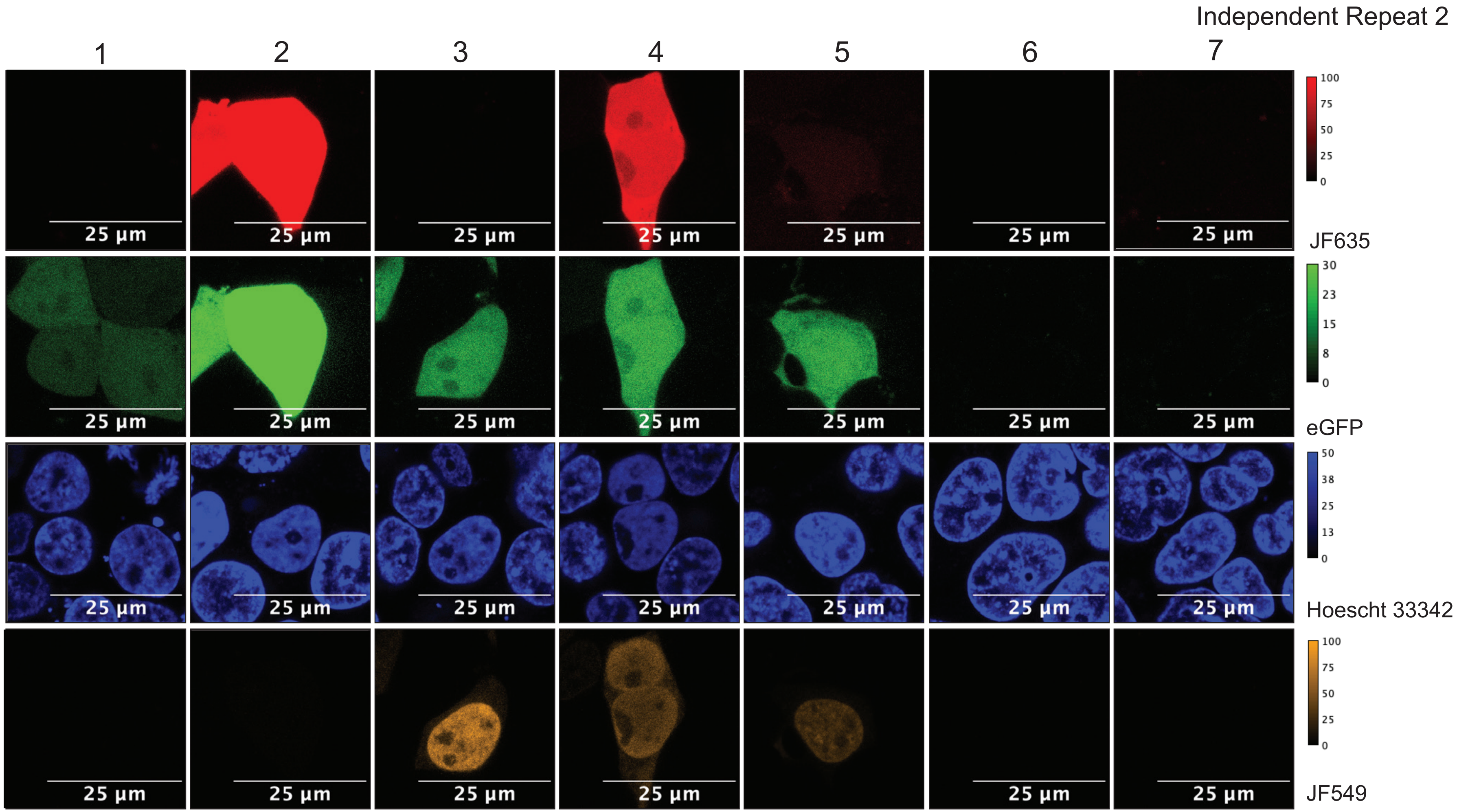

2 hour treatment
Representative blots of 3 technical repeats

1. Vehicle-treated GFP–BromoCatch / H2B–HaloTag HEK293 cells
2. C10852L (1 µM)–treated GFP–BromoCatch / H2B–HaloTag HEK293 cells
3. JF549 Haloalkane (100 nM)–treated GFP–BromoCatch / H2B–HaloTag HEK293 cells
4. C10852L (1 µM) + JF549 Haloalkane (100 nM)–treated GFP–BromoCatch / H2B–HaloTag HEK293 cells
5. C10852L (1 µM) + JF549 Haloalkane (100 nM) + ET-JQ1-OMe (25 µM, 30-min pre-treatment) GFP–BromoCatch / H2B–HaloTag HEK293 cells
6. Vehicle-treated mock-transfected HEK293 cells
7. C10852L (1 µM) + JF549 Haloalkane (100 nM)–treated mock-transfected HEK293 cells

Channels were scaled independently, but settings were kept consistent across conditions within each channel; the calibration scales are therefore representative of all panels shown.

Supplementary Figure 22. Live-cell confocal imaging of BromoCatch and HaloTag multiplexing in HEK293 cells co-transfected with GFP–BromoCatch and H2B–HaloTag and co-treated with C10852L (1 µM) and JF549 Haloalkane (100 nM). HEK293 cells transiently co-expressing GFP–BromoCatch and H2B–HaloTag were treated with C10852L (1 µM) and JF549 Haloalkane (100 nM). Probe activation was assessed following 2-hour treatment with DMSO (vehicle), C10852L (1 µM), and/or JF549 Haloalkane (100 nM). Hoechst 33342 was used as a nuclear counterstain. Fluorescence emission at 657-688 nm was collected as the readout for "switch-on" activation of the C10852L probe (red channel). Hoechst 33342 emission was detected at 437-467 nm (blue channel), eGFP emission at 499-523 nm (green channel), and JF549 emission at 576-624 nm (orange channel), enabling selective visualisation of probe engagement with GFP–BromoCatch and H2B–HaloTag. Experimental controls included: (i) vehicle-treated cells, (ii) competition assays in which cells were pre-incubated for 30 minutes with a saturating concentration of ET-JQ1-OMe (25 µM) prior to probe treatment to block BromoCatch engagement and confirm target-dependent activation, and (iii) mock-transfected cells treated with each probe to assess non-specific background fluorescence. Image panels are representative images of three technical replicates per condition per independent experiment. A total of N=2 independent biological repeats were performed. All microscopy images were processed in FIJI v1.54.

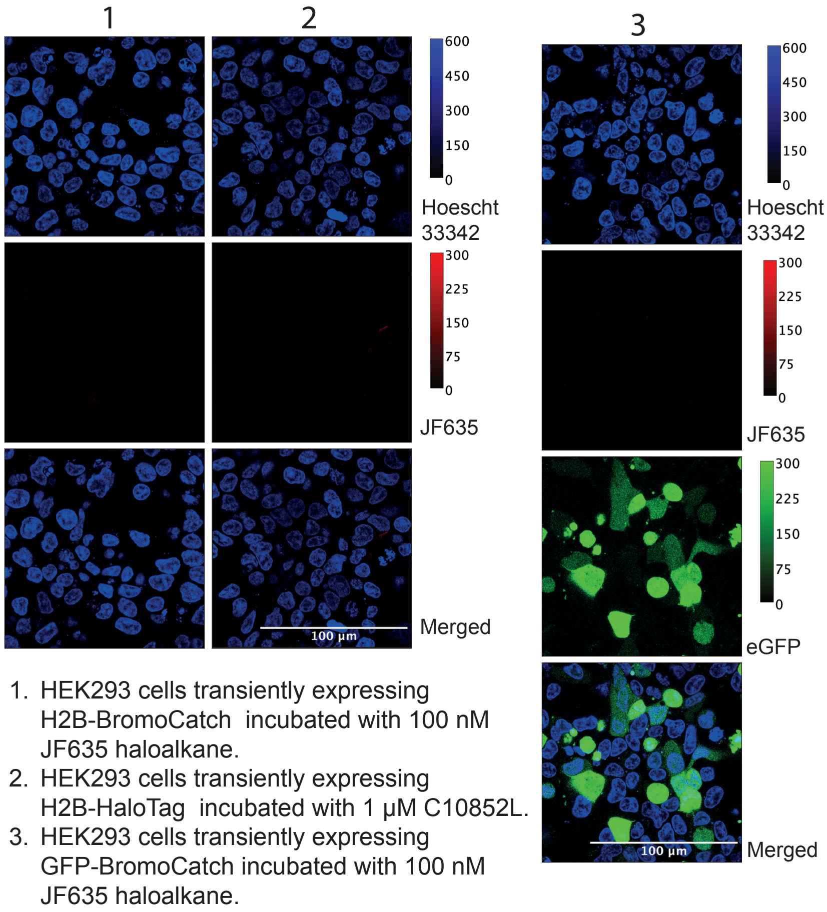

Supplementary Figure 23. BromoCatch and HaloTag probe specificity. Transiently transfected HEK293 cells expressing only the alternative tag were treated with 1 µM C10852L (H2B-HaloTag) or 100 nM JF635 Haloalkane (H2B-/GFP-BromoCatch) for 2 hours, counterstained with Hoechst 33342, and imaged as z-stacks. Single z-slice images are shown for clarity. Two technical images were captured per condition for each of the N = 2 independent experiments. No far red signal was detected in the JF635 channel, confirming chemical independence of BromoCatch and HaloTag labelling.

Supplementary Table 1. **Reversible and covalent ligand docking with both E438C and M442C mutants of Brd4-BD2.** Parentheses indicate the corresponding cysteine-containing mutations in Brd2-BD2.

			$Brd4BD2^{L387A,M442C}$				$Brd4BD2^{L387A,E438C}$			
"B&H" residue			L387A (L383V)				L387A (L383V)			
"Trap" residue			M442C (M438C)				E438C (D434C)			
Docking type			Reversible		Covalent		Reversible		Covalent	
			Glide score (kcal/mol)	RMSD (Å)	Cov score (kcal/mol)	RMSD (Å)	Glide score (kcal/mol)	RMSD (Å)	Cov score (kcal/mol)	RMSD (Å)
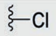	ET-JQ1	*para*	-7.38	0	N/A	N/A	-8.12	0	N/A	N/A
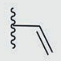	MR100	*para*	-7.38	0.03	N/R	N/A	-7.85	0.15	N/R	N/A
	MR108	*meta*	-7.76	0.14	N/R	N/A	-7.8	0.04	N/R	N/A
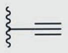	1	*para*	-7.41	0.04	-5.54	1.592	-8.14	0.04	-7.546	0.09
	2	*meta*	-7.94	0.1	-7.4	0.299	-7.55	0.74	-7.006	0.39
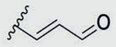	3	*para*	-7.40	0.7	-4.79	3.428	-7.78	0.17	-7.552	0.13
	4	*meta*	-6.03	2.87	-5.91	3.316	-6.16	2.97	-7.19	0.21
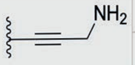	MR101	*para*	-8.29	0.73	-7.97	0.421	-8	0.04	-7.36	0.09
	MR109	*meta*	-6.72	2.93	-7.01	1.687	-6.59	3.02	-7.18	0.15
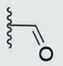	MR70	*para*	-7.52	0.04	-4.31	3.139	-7.86	0.75	-7.45	0.15
	MR115	*meta*	-7.77	0.2	-7.28	0.473	-7.68	0.32	-7.32	0.2
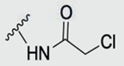	MR112	*para*	-7.50	0.08	-5.82	0.791	-7.77	0.04	-7.17	0.09
	MR118	*meta*	-7.78	0.09	-7.68	0.119	-7.62	0.06	-7.23	0.16
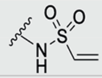	MR121	*para*	-7.31	0.1	-7.53	0.148	-7.35	0.08	-7.14	0.08
	MR117	*meta*	-7.05	0.06	-7.39	0.147	-7.48	0.07	-7.18	0.12
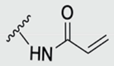	MR116	*para*	-5.00	3.09	-6.68	0.468	-6.1	3.03	-7.29	0.09
	MR119	*meta*	-8.27	0.34	-8.2	0.355	-7.8	0.34	-6.67	1.2
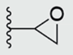	MR104	*para*	-6.31	2.92	-4.14	1.60	-7.37	3.41	-7.4	1.52
	MR111	*meta*	-7.99	0.09	-7.65	0.414	-7.59	0.08	-7.31	0.21

Supplementary Table 2. Summary of intact MS screening results to assess covalency of the protein-ligand system. Values are % modification of the protein in the presence of ligand based on the UV absorbance and the mass increase. Measurements are representative of two independent experiments. n.d = not detected.

Brd4-BD2 protein	ET-JQ1-OMe	MR100	MR108	MR101	MR109	MR70	MR115	MR104	MR111	MR112	MR118	MR121	MR117	MR116	MR119
WT	n.d.	n.d.	n.d.	n.d.	n.d.	n.d.	n.d.	5.3	n.d.	n.d.	n.d.	n.d.	7.4	n.d.	n.d.
L387A	n.d.	n.d.	n.d.	n.d.	n.d.	n.d.	n.d.	n.d.	n.d.	n.d.	n.d.	n.d.	8.6	n.d.	n.d.
L387A, M442C	n.d.	n.d.	n.d.	n.d.	n.d.	n.d.	n.d.	n.d.	n.d.	n.d.	n.d.	100	21.8	49.3	25
L387A, E438C	n.d.	n.d.	n.d.	n.d.	n.d.	n.d.	22	100	100	100	100	100	100	100	100

Supplementary Table 3. Summary of DSF screening results to assess thermal stabilisation of the protein-ligand system. The ΔT_m (°C) were calculated in reference to the corresponding apo proteins. The melting temperatures for the apo proteins were: Brd4-BD2 (apo) Tm = 53 °C; Brd4-BD2^{L387A} (apo) Tm = 51°C; Brd4-BD2^{L387A,E438C} (apo) Tm = 49.3 °C; Brd4-BD2^{L387A,M442C} (apo) Tm = 51°C. Data are average from N=3 technical replicates.

Brd4-BD2 protein	ET-JQ1-OMe	MR100	MR108	MR101	MR109	MR70	MR115	MR104	MR111	MR112	MR118	MR121	MR117	MR116	MR119
WT	5.0	5.0	3.7	2.7	3.0	4.0	2.0	3.0	2.3	6.0	5.0	2.0	3.0	4.7	6.0
L387A	10.0	11.0	10.0	7.7	9.0	9.0	8.7	9.0	8.0	12.3	12.0	6.3	10.0	9.3	12.3
L387A, M442C	7.3	9.0	9.3	5.7	7.0	6.3	6.0	7.0	6.7	15.0	9.0	21.0	7.0	12.3	9.3
L387A, E438C	11.6	12.0	11.3	8.3	11	12.7	8.0	28.3	24.3	23.7	19.3	25.3	21.7	26.0	21.0

Supplementary Table 4. GSH stability assay.

	R^2	$T_{1/2}$ (min)	Remaining (T=1440 min)	Remaining (NGSH*=1440 min)	Glutathione conjugation (a.u.c. at 1440 min)
**MR112 (1 µM)**	0.9997	20.4	0.0%	89.6%	0
**MR112 (10 µM)**	0.9998	21.2	0.0%	92.9%	33040
**MR116 (1 µM)**	0.9997	20.6	0.0%	94.8%	238938
**MR116 (10 µM)**	0.9986	21.3	0.0%	96.9%	3994627
**MR117 (1 µM)**	1.0000	5.2	0.0%	96.1%	409383
**MR117 (10 µM)**	1.0000	5.1	0.0%	93.7%	8713253
**MR118 (1 µM)**	0.9993	23.0	0.0%	92.6%	0
**MR118 (10 µM)**	1.0000	24.1	0.0%	89.0%	15490
**MR119 (1 µM)**	0.9983	47.9	0.0%	97.0%	34103
**MR119 (10 µM)**	0.9993	48.0	0.0%	89.6%	377886
**MR121 (1 µM)**	1.0000	5.0	0.0%	97.0%	298650
**MR121 (10 µM)**	1.0000	5.1	0.0%	92.8%	6781276
**Afatinib**	0.9964	17.0	0.4%	99.3%	208578
**Ibrutinib**	0.9838	353.3	5.3%	91.0%	996320
R^2 is the correlation coefficient of the linear regression for the determination of kinetic constant (see raw data worksheet)					
$T_{1/2}$ is half life. A.u.c. is area under the curve					
* NGSH is abbreviation for "no glutathione". Buffer is added in place of glutathione in the NGSH sample during the 24 hour-incubation					

Supplementary Table 5. NanoBRET target engagement binding affinities. Tabulated IC_{50} values obtained from data curve fitting of compounds displacing 1 µM ET-JQ1-PEG$_3$-BODIPY (MR141) from NanoLuc-tagged Brd4-BD2^{L387A,E438C} in HEK293 cells. Data is mean pIC_{50} ± s.e.m. and IC_{50} in nM from N=3 independent repeats using "log(inhibitor) vs. response (three parameters)" in Graphpad Prism Version 10.2.3.

Compound	IC_{50} (nM)	pIC_{50}	s.e.m. (pIC_{50})
**ET-JQ1-OMe**	83.8	7.1	0.08
**MR70**	46.3	7.3	0.17
**MR104**	32.3	7.5	0.10
**MR111**	81.6	7.1	0.18
**MR112**	15.9	7.8	0.12
**MR115**	200.1	6.7	0.22
**MR116**	31.1	7.5	0.12
**MR117**	183.8	6.7	0.20
**MR118**	8.0	8.1	0.18
**MR119**	110.7	7.0	0.12
**MR121**	318.1	6.5	0.22

Supplementary Table 6. $T_{1/2}$ and D_{MAX} values for MZ1, AGB1 and MR170 derived from live cell kinetic assay. $T_{1/2}$ and D_{Max} values extracted from live cell kinetic degradation assay in an endogenous HiBiT-BromoCatch-Brd4 HEK293 cell line. $T_{1/2}$ and D_{max} values calculated as mean from two independent biological experiments using "one phase decay' model from Graphpad Prism Version 10.

	MZ1 (1 µM)	AGB1 (1 µM)	MR170 (2.5 µM)
**$T_{1/2}$ (minutes)**	28.9	29.2	81.5
**D_{MAX}**	89.3	86.3	61.7

Supplementary Table 7. DC_{50} values for MZ1, AGB1 and C10852S measured in lytic cell degradation assay. DC_{50} values extracted from HiBiT lytic degradation assay in endogenous HiBiT-BromoCatch-Brd4, HiBiT-Brd4, HiBiT-Brd3 and HiBiT-Brd2 HEK293 cell lines. DC_{50} values calculated as mean of N=3 independent biological experiments using log(inhibitor) vs. response (three parameters) using Graphpad Prism Version 10.2.3. Degradation curves for AGB1 against endogenous Brd2/3/4 exhibited <25% maximal reduction in protein level at all concentrations, consistent with AGB1 selectivity for BromoTag/Catch over the endogenous non-mutant BET proteins. No BET protein degradation was observed with C10852S. n.d. - not determined.

DC_{50} (nM)	MZ1	AGB1	C10852S
**BromoCatch**	151	21.2	n.d.
**Brd4**	85.9	n.d.	n.d.
**Brd3**	432	n.d.	n.d.
**Brd2**	240.3	n.d.	n.d.

Supplementary Table 8. Mean Fluorescence Intensity (MFI) values from independent replicates corresponding to Figure 10A (main text) and Supplementary Figure 17. MFI values were quantified from individual images for each condition in the comparative live-cell confocal imaging experiment above. Summed Z-stack projections were produce of both channels. The Hoechst 33342 counterstain nuclear channel was applied to the JF635 'switch-on' channel to measure average fluorescence intensity in the JF635 channel using a nuclear ROI from the hoescht 33342 channel. Background fluorescence was measured separately and subtracted. For each biological replicate, the mean MFI was calculated from two technical replicates, and these values were averaged to produce the MFI for that individual replicate subsequently used to determine the overall mean ± s.e.m. N=3 independent replicates. arb.units = arbitrary units.

	H2B-BromoCatch						H2B-HaloTag	
MFI (arb.units)	C10852S 1 µM Mock	C10852S 1 µM	C10852L 0.5 µM Mock	C10852L 0.5 µM	C10852S 1 µM 25 µM ET-JQ1-OMe	C10852L 0.5 µM 25 µM ET-JQ1-OMe	JF635 Haloalkane 100 nM Mock	JF635 Haloalkane 100 nM
N1	2.6	1497.1	18.9	3001.4	44.6	11.1	7.0	6820.4
N2	6.5	1179.9	19.7	3515.7	87.6	58.5	6.4	5308.5
N3	2.8	1421.2	11.9	3189.6	13.1	16.7	0.8	4172.4
Mean	4.0	1366.1	16.8	3235.5	48.4	28.8	4.7	5433.7
STDEV	2.2	165.7	4.3	260.2	37.4	25.9	3.4	1328.4
SEM	1.3	95.6	2.5	150.2	21.6	14.9	2.0	767.0

Supplementary Table 9. Extracted Pearson's r values from N=2 independent biological replicates of orthogonal multiplexing BromoCatch/HaloTag corresponding to Figure 10C (main text) and Supplementary Figure 22. Pearson's r coefficients extracted from colocalsation analysis performed on corresponding images of dual transfected HEK293 treated with either C10852L 1 μM + JF549 Haloalkane 100 nM or JF549 Haloalkane 100 nM alone (Figure 10C, Supplementary Figure 20). Mean produced from N=2 biological repeats. All images were processed in FIJI v1.54.

Pearson's r value	JF549 Haloalkane 100 nM + C10852L 1 μM	JF549 Haloalkane 100 nM
N1	0.19	0.21
N2	0.19	0.24
Mean	0.19	0.22

Supplementary Table 10. List of buffers used for *in vitro* and *in cellulo* experiments.

Buffer	Composition
Activity buffer	25 mM HEPES, 100 mM NaCl, 1 mM TCEP
Kinetic assays buffer	25 mM HEPES, 100 mM NaCl
Loading buffer	4×LDS sample buffer, 2% DTT
MES running buffer	50 mM MES, 50 mM Tris Base, 0.1% SDS, 1 mM EDTA, pH 7.3
MOPS running buffer	50 mM MOPS, 50 mM Tris Base, 0.1% SDS, 1 mM EDTA, pH 7.7, 0.01-0.09% *N*,*N*-dimethylformamide
Transfer buffer	10% v/v 10x Transfer buffer, 20% v/v ethanol, 80% v/v ddH_2O
TBS-T buffer	TBS, 0.1% Tween
RIPA Buffer	1% Triton X-100, 0.1% SDS, and 1:200 Protease inhibitor cocktail

Supplementary Table 11. Protein expression plasmids.

<table>
<tr><th>Protein</th><th>Vector</th><th>Tag / Cleavage Site</th><th>Cloning</th><th>Antibiotic</th><th>Restriction Primers</th><th>Mutation primers</th></tr>
<tr><td>Brd4-BD2 L387A+438C (351-460)</td><td rowspan="5">pRSF-DUET</td><td rowspan="4">His-TEV</td><td rowspan="6">BamHI-EcoRI</td><td rowspan="6">KAN</td><td>Olg13/Olg14</td><td>Olg-80/Olg-81/Olg-84/Olg-85</td></tr>
<tr><td>Brd4-BD2 L387A+M442C (351-460)</td><td>Olg13/Olg14</td><td>Olg-80/Olg-81/Olg-86/Olg-87</td></tr>
<tr><td>Brd4-BD2 (333-460)</td><td>Olg132/Olg14</td><td>Olg132/Olg14</td></tr>
<tr><td>Brd4-BD2 L387A (333-460)</td><td>Olg132/Olg14</td><td>Olg80/Olg81</td></tr>
<tr><td>Brd4-BD2 L387A,E438C (333-460)</td><td>His-SUMO</td><td>Olg132/Olg14</td><td>Olg132/Olg14</td></tr>
<tr><td>Brd2-BD2 L383A + D434C (351-460)</td><td>DUET</td><td>His-MBP-TEV</td><td>Olg229/Olg230</td><td>Olg232/Olg233/Olg234/Olg235</td></tr>
</table>

Supplementary Table 12. Primers for DNA constructs.

Name	Construct	DNA sequence (5' to 3')	Direction
Olg-13	Brd4-BD2 (351-C)	taagcaGGATCCTCGGAGCAGCTCAAGTGC TGCAGC	Forward
Olg-132	Brd4-BD2 (333-C)	taagcaGGATCCAAGGACGTGCCCGACTCT CAGCAG	Forward
Olg-14	Brd4-BD2 (459-N)	tgcttaGAATTCTTAGTCCGGCATCTTGGCA AAGCGCATTTCG	Reverse
Olg-80	Brd4D2 (L387A)	GACGTGGAGGCACTGGGCGCACACGACT ACTGTGACATCAT	MUT-forward
Olg-81	Brd4-BD2 (L387A)	ATGATGTCACAGTAGTCGTGTGCGCCCA GTGCCTCCACGTC	MUT-reverse
Olg-84	Brd4-BD2 (E438C)	GTACAACCCTCCTGACCATTGCGTGGTG GCCATGGCCCGCAAG	MUT-forward
Olg-85	Brd4-BD2 (E438C)	CTTGCGGGCCATGGCCACCACGCAATGG TCAGGAGGGTTGTAC	MUT-reverse
Olg-86	Brd4-BD2 (M442C)	GACCATGAGGTGGTGGCCTGCGCCCGCA AGCTCCAGGATG	MUT-forward
Olg-87	Brd4-BD2 (M442C)	CATCCTGGAGCTTGCGGGCGCAGGCCAC CACCTCATGGTC	MUT-reverse
Olg-234	Brd2-BD2 (D434C)	GTATAACCCGCCGGACCATTGCGTCGTA GCAATGGCCCGCAAG	MUT-forward
Olg-235	Brd2-BD2 (D434C)	CTTGCGGGCCATTGCTACGACGCAATGG TCCGGCGGGTTATAC	MUT-reverse
Olg-229	Brd2-BD2 (348-C)	taagcaGGATCCGCAAACCCTGAACAACTT AAACATTGCAATGGTATTTTAAAAGAG	Forward
Olg-230	Brd2-BD2 (455-N)	tgcttaGAATTCTTAATCGGGCATCTTTGCA TAGCGGAACTCG	Reverse
Olg-232	Brd2-BD2 (L383A)	GTGGACGCCAGTGCCTTGGGAGCACACG ACTATCATGATATTATCAAG	MUT-forward
Olg-233	Brd2-BD2 (L383A)	CTTGATAATATCATGATAGTCGTGTGCTC CCAAGGCACTGGCGTCCAC	MUT-reverse
Cys1_FRW	**eGFP-IRES-HiBiT-** Brd4-BD2^{L387A,E438C}-Brd4 SDM	TCCTGACCATTGTGTGGTGGCCATG	Forward
Cys1-Rev	**eGFP-IRES-HiBiT-** Brd4-BD2^{L387A,E438C}-Brd4 SDM	GGGTTGTACTTATAGCAGTTG	Reverse

Supplementary Table 13. Protein sequences.

Protein	Residues	Sequence
Brd4-BD2	333-460	KDVPDSQQHPAPEKSSKVSEQLKCCSGILKEMFAKKHAA YAWPFYKPVDVEALG**L**HDYCDIIKHPMDMSTIKSKLEA REYRDAQEFGADVRLMFSNCYKYNPPDHEVVAMARKL QDVFEMRFAKMPDE
Brd4-BD2^{L387A}	333-460	KDVPDSQQHPAPEKSSKVSEQLKCCSGILKEMFAKKHAA YAWPFYKPVDVEALG**A**HDYCDIIKHPMDMSTIKSKLEA REYRDAQEFGADVRLMFSNCYKYNPPDHEVVAMARKL QDVFEMRFAKMPDE
Brd4-BD2^{L387A,E438C}	333-460	KDVPDSQQHPAPEKSSKVSEQLKCCSGILKEMFAKKHAA YAWPFYKPVDVEALG**A**HDYCDIIKHPMDMSTIKSKLEAR EYRDAQEFGADVRLMFSNCYKYNPPDH**C**VVAMARKLQ DVFEMRFAKMPDE
Brd4-BD2^{L387A,E438C}	351-459	SEQLKCCSGILKEMFAKKHAAYAWPFYKPVDVEALG**A**H DYCDIIKHPMDMSTIKSKLEAREYRDAQEFGADVRLMFS NCYKYNPPDH**C**VVAMARKLQDVFEMRFAKMPD
Brd4-BD2^{L387A,M442C}	351-459	SEQLKCCSGILKEMFAKKHAAYAWPFYKPVDVEALG**A**H DYCDIIKHPMDMSTIKSKLEAREYRDAQEFGADVRLMFS NCYKYNPPDHEVVA**C**ARKLQDVFEMRFAKMPD
Brd2-BD2^{L383A,D434C}	348-455	EQLKHCNGILKELLSKKHAAYAWPFYKPVDASALG**A**HD YHDIIKHPMDLSTVKRKMENRDYRDAQEFAADVRLMFS NCYKYNPPDH**C**VVAMARKLQDVFEFRYAKMPD

Supplementary Table 14. Plasmids for live cell work.

Plasmid	Residues	Amino Acid Sequence	Vector Backbone
NanoLuciferase-linker-Brd4-BD2^{L387A,E438C}	333-460	MVFTLEDFVGDWRQTAGYNLDQVLEQ<br>GGVSSLFQNLGVSVTPIQRIVLSGENGL<br>KIDIHVIIPYEGLSGDQMGQIEKIFKVVY<br>PVDDHHFKVILHYGTLVIDGVTPNMID<br>YFGRPYEGIAVFDGKKITVTGTLWNGN<br>KIIDERLINPDGSLLFRVTINGVTGWRLC<br>ERILAGSSGAIAKDVPDSQQHPAPEKSS<br>KVSEQLKCCSGILKEMFAKKHAAYAW<br>PFYKPVDVEALGAHDYCDIIKHPMDMS<br>TIKSKLEAREYRDAQEFGADVRLMFSN<br>CYKYNPPDHCVVAMARKLQDVFEMRF<br>AKMPDE*	pcDNA™3.1 (+) Mammalian Expression Vector
H2B-linker-Brd4-BD2^{L387A,E438C}	333-460	MPEPSKSAPAPKKGSKKAITKAQKKDG<br>KKRKRSRKESYSIYVYKVLKQVHPDTG<br>ISSKAMGIMNSFVNDIFERIAGEASRLA<br>HYNKRSTITSREIQTAVRLLLPGELAKH<br>AVSEGTKAVTKYTSSGAGAGAGAGAK<br>DVPDSQQHPAPEKSSKVSEQLKCCSGIL<br>KEMFAKKHAAYAWPFYKPVDVEALG<br>AHDYCDIIKHPMDMSTIKSKLEAREYR<br>DAQEFGADVRLMFSNCYKYNPPDHCV<br>VAMARKLQDVFEMRFAKMPDE	pcDNA™3.1 (+) Mammalian Expression Vector
H2B-linker-Brd4-BD2^{L387A,E438C}	351-460	MPEPSKSAPAPKKGSKKAITKAQKKDG<br>KKRKRSRKESYSIYVYKVLKQVHPDTGI<br>SSKAMGIMNSFVNDIFERIAGEASRLAH<br>YNKRSTITSREIQTAVRLLLPGELAKHA<br>VSEGTKAVTKYTSSGAGAGAGAGASEQ<br>LKCCSGILKEMFAKKHAAYAWPFYKPV<br>DVEALGAHDYCDIIKHPMDMSTIKSKLE<br>AREYRDAQEFGADVRLMFSNCYKYNPP<br>DHCVVAMARKLQDVFEMRFAKMPDE*	pcDNA™3.1 (+) Mammalian Expression Vector
Brd4-BD2^{L387A,E438C}-linker-GFP		MKDVPDSQQHPAPEKSSKVSEQLKCCS<br>GILKEMFAKKHAAYAWPFYKPVDVEAL<br>GAHDYCDIIKHPMDMSTIKSKLEAREYR<br>DAQEFGADVRLMFSNCYKYNPPDHCVV<br>AMARKLQDVFEMRFAKMPDEASDIGAP<br>AFKSVQTGEFTSKGEELFTGVVPILVELD<br>GDVNGHKFSVSGEGEGDATYGKLTLKF<br>ICTTGKLPVPWPTLVTTFSYGVQCFSRY<br>PDHMKRHDFFKSAMPEGYVQERTIFFK<br>DDGNYKTRAEVKFEGDTLVNRIELKGID<br>FKEDGNILGHKLEYNYNSHNVYIMADK<br>QKNGIKVNFKIRHNIEDGSVQLADHYQQ<br>NTPIGDGPVLLPDNHYLSTQSALSKDPN<br>EKRDHMVLLEFVTAAGITHGMDELYK*	pcDNA™3.1 (+) Mammalian Expression Vector
H2B-linker-HaloTag		MPEPSKSAPAPKKGSKKAITKAQKKDG<br>KKRKRSRKESYSIYVYKVLKQVHPDTGI<br>SSKAMGIMNSFVNDIFERIAGEASRLAH<br>YNKRSTITSREIQTAVRLLLPGELAKHA	pcDNA™3.1 (+) Mammalian Expression Vector

		VSEGTKAVTKYTSSGAGAGAGAGAAEI GTGFPFDPHYVEVLGERMHYVDVGPRD GTPVLFLHGNPTSSYVWRNIIPHVAPTH RCIAPDLIGMGKSDKPDLGYFFDDHVRF MDAFIEALGLEEVVLVIHDWGSALGFH WAKRNPERVKGIAFMEFIRPIPTWDEWP EFARETFQAFRTTDVGRKLIIDQNVFIEG TLPMGVVRPLTEVEMDHYREPFLNPVD REPLWRFPNELPIAGEPANIVALVEEYM DWLHQSPVPKLLFWGTPGVLIPPAEAAR LAKSLPNCKAVDIGPGLNLLQEDNPDLI GSEIARWLSTLEISG*	
**eGFP**-**IRES**-**HiBiT**-Brd4-BD2^{L387A,E438C}-Brd4	333-460	MSKGEELFTGVVPILVELDGDVNGHKFS VSGEGEGDATYGKLTLKFICTTGKLPVP WPTLVTTLTYGVQCFSRYPDHMKQHDF FKSAMPEGYVQERTIFFKDDGNYKTRA EVKFEGDTLVNRIELKGIDFKEDGNILG HKLEYNYNSHNVYIMADKQKNGIKVNF KIRHNIEDGSVQLADHYQQNTPIGDGPV LLPDNHYLSTQSALSKDPNEKRDHMVL LEFVTAAGITLGMDELYK*RSAPLPPPL TLLAEAAWNKAGVRLSICYFPPYCRLLA M*GPGNLALSS*RAFLGVFPLSPKECKV C*MS*RKQFLWKLLEDKQRL*RPFAGSG TPHLATGASAAKSHVYKIHLQRRHNPSA TL*VG*LWKESNGSPQAYSTRG*RMPRR YPIVWDLIWGLGAHALHVFSRG*KNV* APRTTGTWFSFEKHDDNMATTMVSGW RLFKKISGGGGGGKDVPDSQQHPAPEKS SKVSEQLKCCSGILKEMFAKKHAAYAW PFYKPVDVEALGAHDYCDIIKHPMDMS TIKSKLEAREYRDAQEFGADVRLMFSNC YKYNPPDHCVVAMARKLQDVFEMRFA KMPDEGAGAGAGAGA	pMA-RQ

Supplementary Table 15. gRNA sequences used for BromoCatch knock-in of Brd4. Red bases indicate PAM sequence.

Target	Sequence
BRD4$_{gRNA1}$	TGGGATCACTAGCATGTCTGCGG
BRD4$_{gRNA2}$	ACTAGCATGTCTGCGGAGAGCGG

Supplementary Table 16. Crystallographic data collection and refinement statistics.

	PDB 9QRK
**Data collection**	
Space group	$P2_12_12$
Cell dimensions	
a, *b*, *c* (Å)	32.05, 52.13, 71.64
α, β, γ (°)	90, 90, 90
Resolution (Å)	35.85 – 1.30 (1.32-1.30)*
R_{merge}	0.035 (0.096)
(I / I_{SD})	43.6 (20.7)
Completeness (%)	99.9 (99.2)
Redundancy	13.2 (13.2)
**Refinement**	
Resolution (Å)	35.85 – 1.30
Number of reflections/free	30269 (1503)
R_{work} / R_{free}	0.147 / 0.163
No. atoms	
Protein	1987
Ligand/ion	134
Water	161
B-factors	
Protein	11.37
Ligand/ion	11.51
Water	21.72
R.m.s. deviations	
Bond lengths (Å)	0.0144
Bond angles (°)	2.08

*Values in parentheses are for highest-resolution shell.

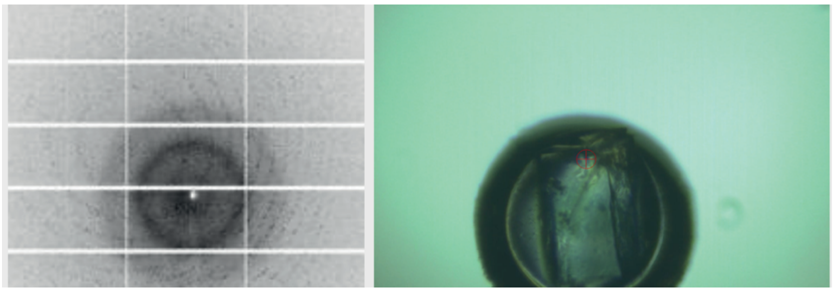

Supplementary methods

Extended in vitro kinetics – competition-based assay kinetic evaluation of MR116 and MR169

Kinetic data from microplate reader assays to evaluate the kinetics of the MR116 ligand and probe MR169 used a competition assay previously described in our group.[1] The BromoCatch covalent system should follow a two-step mechanism where the initial step is reversible (k_i) followed by a covalent step (k_{inact}).

$$P + I \overset{k_i}{\leftrightarrow} PI^* \xrightarrow{k_{inact}} PI \tag{10}$$

The first reversible equilibria is reached fast in our system. We observed that, at the time of the first kinetic read, that has a delay of 30 seconds from reaction initiation, the reversible binding equilibrium was reached and the changes we see in the polarisation measurements are mainly governed by the covalent step, as exemplified in the comparative experiment between the reversible and covalent mechanism in Figure 6 of the manuscript (BMG Labtech PHERAstar – firmware v1.33). The data was analysed using a simplified pseudo-first order model to fit the data to determine k_{inact}/K_I.

$$P + I \xrightarrow{k_{inact}/K_I} PI \tag{1}$$

To monitor the MR116 ligand binding kinetics we synthesised a sulfonated Cy5-labeled reversible tracer (MR133). The fluorescence polarization (mA) of a 2-fold serial dilution from a top concentration of 3 µM BromoCatch in 10 nM of the probe in activity buffer was measured to determine the K_L (BMG Labtech PHERAstar – firmware v1.33).

Protein titration and the curves were fitted using GraphPad Prism 9, using one-site fitting models to determine the tracer binding constant, K_L and decide the protein:tracer ratio to use in the competition assay. Samples were run in triplicate in 384 well plates, using a total volume per well of 15 µL. All conditions were run in triplicate (n = 3). The bound fraction of the probe is measure using the equation:

$$F_b = \frac{A_f - A}{A_f - A_b} \tag{2}$$

Where,
A - measured anisotropy.
A_f - free anisotropic value for the fluorescent probe
A_b – bound anisotropic value for the fluorescent probe

The time-dependent reduction in anisotropy was measured for a 2-fold serial dilution of compounds MR116 or MR169 in a mixture of 10 nM probe + 30-60 nM Brd4-BD2^{L387A,E438C} (Fb v/s time plot).

The k_{obs} from F_b versus time graph was obtained by fitting a one-phase exponential decay for each concentration curve. The k_{obs} obtained at each of compound concentration was plotted against the concentration of inhibitor using Michaelis–Menten model of GraphPad Prism 9, which provides the k_{inact} (maximum potential rate of inactivation, V_{max}) and the apparent K_I (K_m, concentration of MR116 at which $k_{obs} = k_{inact}/2$). The observed rate constant, k_{obs} is related to K_I and k_{inact} by the equation:

$$k_{obs} = \frac{k_{inact}[I]}{K_I + [I]} \tag{3}$$

Taking into account that this is a competition assay. The K_I calculated using the Michaelis Menten model yields $K_{I,app}$ and is converted into $K_{I,}$ using the following relationship.

$$K_I = \frac{K_{I,app}}{1 + [L]/K_L} \tag{4}$$

To determine the initial reversible binding affinity, the initial bound fraction of the probe at time = 0, Fb_o (the y-intercept in Fb v/s time plot) was plotted against the $\log_{10}$ of compound (MR116 or MR169) concentration, and the obtained IC_{50} is then converted to the estimated K_i.

Convert the IC_{50} to K_i using the equation below (see Source Data File):

$$IC_{50} = aK_I + b \tag{11}$$

$$a = \frac{(2[P]_{tot} - f_0(L)_0)(2 - f_0)}{2K_L f_0} - 1 \tag{12}$$

$$a = [P]_{tot} - \frac{K_L f_0}{2 - f_0} - \frac{f_0(L)_0}{2} \tag{13}$$

To calculate the k_{inact}/K_I with an orthogonal method, we used the Krippendorff equation,[2,3] looking at the IC_{50} decay over time for irreversible inhibitors to determine the values of k_{inact} and K_I. L is the ligand tracer concentration (10 nM) and K_L binding affinity (10 nM) (see Source Data File).

$$Y = \frac{K_I \cdot (\frac{[L]}{K_L} + 1)}{\dfrac{-k_{inact} \cdot \left(\dfrac{y}{y + K_I \cdot (\frac{[L]}{K_L} + 1)}\right) \cdot x}{-k_{inact} \cdot \left(\dfrac{y}{y + K_I \cdot (\frac{[L]}{K_L} + 1)}\right) \cdot x - 2 \cdot exp\left(-k_{inact} \cdot \left(\dfrac{y}{y + K_I \cdot (\frac{[L]}{K_L} + 1)}\right) \cdot x\right) + 2}} \tag{14}$$

Text format for Prism: Y=(((1+(S/Km))*KI)/(((kinact*(y/((KI*(1+(S/Km)))+y)))*x)/((2-((kinact*(y/((KI*(1+(S/Km)))+y)))*x))-(2*exp((((-1)*kinact)*(y/((KI*(1+(S/Km)))+y)))*x)))))

Chemistry

Unless otherwise stated, all reagents and solvents were purchased from commercial sources and used without further purification. Nuclear magnetic resonance spectra were recorded on a Bruker Ascend 500 MHz spectrometer or a Bruker Avance III HD spectrometer, operating at 500 MHz and 400 MHz for ^{1}H NMR, respectively, 100 MHz for ^{13}C NMR, and 376 MHz for ^{19}F NMR. ^{1}H NMR and ^{13}C NMR chemical shifts (δ) are reported in parts per million (ppm) and are referenced to residual protium in solvent and to the carbon resonances of the residual solvent peak respectively. DEPT and correlation spectra were run in conjunction to aid assignment. ^{19}F NMR chemical shifts are reported in ppm and are uncorrected. Coupling constants (*J*) are quoted in Hertz (Hz), and the following abbreviations were used to report multiplicity: s= singlet, d= doublet, dd= doublet of doublets, ddd= double doublet of doublets, t= triplet, dtt= double triplet of triplets, q= quartet, m= multiplet, br s= broad singlet. Liquid chromatography-mass spectrometry (LC-MS) was carried out on a Shimadzu HPLC/MS 2020 equipped with a Hypersil Gold column (1.9 µm particle size, 50 × 2.1 mm), photodiode array detector and ESI detector, or by using Agilent InfinityLab LC/MSD systems. Purification by flash column chromatography was carried out using Fisher Scientific silica gel 60Å (35-70 µm), or by using Biotage Selekt, Biotage Isolera, Grace Reveleris, Buchi Pure, or Teledyne Isco Combiflash systems. Preparative HPLC was performed on a Waters Preparative HPLC System with a Waters XBridge C18 column (100 mm x 19 mm; 5 µm particle size) and a gradient of 5–95% acetonitrile in water over 18 minutes, flow 25 mL/min, with 0.1% ammonia or 0.1% formic acid in the aqueous phase. Thin layer chromatography was performed on glass plates pre-coated with silica gel (Analtech, UNIPLATE™ 250 µm / UV254), with visualization being achieved using UV light (254 nm) and/or by staining with alkaline potassium permanganate dip. High-resolution mass spectral (HRMS) data were collected in the laboratories of the University of Bath Chemistry Department using an Agilent 6545 LC/Q-TOF system or at the University of Dundee on a Bruker MicroTOF II focus ESI mass spectrometer connected in parallel to a Dionex Ultimate 3000 RSLC system with a diode array detector and a Waters XBridge C18 column (50 mm × 2.1 mm, 3.5 µm particle size).

Methyl (*R*)-2-((*S*)-2,3,9-trimethyl-4-(4-vinylphenyl)-6*H*-thieno[3,2-*f*][1,2,4]triazolo[4,3-*a*][1,4]diazepin-6-yl)butanoate (MR100)

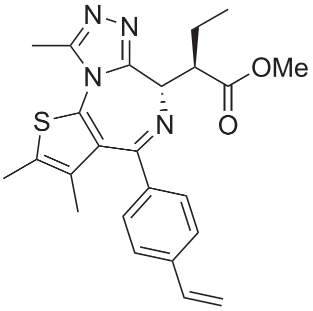

A solution of methyl (*R*)-2-((*S*)-4-(4-chlorophenyl)-2,3,9-trimethyl-6*H*-thieno[3,2-*f*][1,2,4]triazolo[4,3-*a*][1,4]diazepin-6-yl)butanoate ((+)-ET-JQ1-OMe bump, OMe) 40 mg, 0.09 mmol) , potassium vinyltrifluoroborate (36 mg, 0.27 mmol) , XPhos Pd G2 (21 mg, 0.03 mmol), *N,N*-diisopropylethylamine (0.08 mL, 0.45 mmol) in DMF (0.6 mL) and water (0.06 mL) was stirred at 100 °C for 24 hours. Upon cooling to ambient temperature, the mixture was filtered through diatomaceous earth, the filtrate was concentrated under reduced pressure and the residue was and redissolved in dichloromethane. The solution was washed with water (2 x 50 mL) and brine (20 x 50 mL), dried over anhydrous magnesium sulfate and concentrated under reduced pressure. Purification by flash column chromatography, eluting with 0-5% methanol/dichloromethane, afforded the title compound as a yellow solid (35 mg, quant.).

^{1}H NMR (500 MHz, $CDCl_3$) δ 7.32 – 7.24 (m, 4H), 6.64 (dd, *J* = 17.6, 10.9 Hz, 1H), 5.72 (d, *J* = 17.6, 1H), 5.24 (d, *J* = 10.9 Hz, 1H), 4.17 (d, *J* = 10.9 Hz, 1H), 3.93 (td, *J* = 10.8, 3.6 Hz, 1H), 3.79 (s, 3H), 2.60 (s, 3H), 2.34 (s, 3H), 2.16-2.07 (m, 1H), 1.64-1.55 (m, 4H), 0.96 (t, *J* = 7.4 Hz, 3H). ^{13}C NMR (126 MHz, $CDCl_3$) δ 175.4, 163.1, 154.4, 149.7, 143.5, 140.9, 136.8, 136.5, 132.1, 130.9, 130.8, 130.4, 129.8, 128.7, 128.7, 116.0, 59.4, 51.6, 49.7, 23.2, 14.5, 13.1, 11.9, 11.7. LC-MS (m/z, ES+): 435.0 $[M+H]^+$. HRMS (m/z) $[M+H]^+$ calculated for $C_{24}H_{26}O_2N_4S$, 435.18492; found 435.18550.

Methyl (*R*)-2-((*S*)-4-(4-formylphenyl)-2,3,9-trimethyl-6*H*-thieno[3,2-*f*][1,2,4]triazolo[4,3-*a*][1,4] diazepin-6-yl)butanoate (MR70)

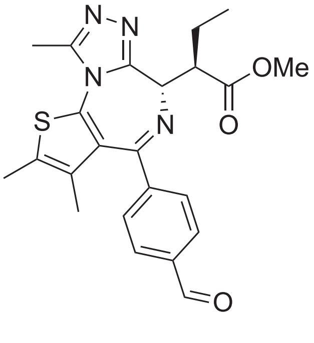

To a stirred solution of methyl (*R*)-2-((*S*)-2,3,9-trimethyl-4-(4-vinylphenyl)-6*H*-thieno[3,2-*f*][1,2,4]triazolo[4,3-*a*][1,4]diazepin-6-yl)butanoate (MR100, 20 mg, 0.05 mmol) and sodium periodate (30 mg, 0.14 mmol) in acetone (0.5 mL) and water (0.1 mL) was added osmium tetroxide (15 µL, 4%w/v solution in water). After stirring for 2 hours, the reaction mixture was diluted with EtOAc. The solution was washed with water, and the organics were dried over anhydrous magnesium sulfate and concentrated under reduced pressure. Purification by preparative reversed phase column chromatography, eluting with 5/95 to 95/5 MeCN/H_2O (0.1 % NH_4OH) afforded the title compound as a white solid (18 mg, quant).

^{1}H NMR (500 MHz, $CDCl_3$) δ 10.07 (s, 1H), 7.88 (d, *J* = 8.5 Hz, 2H), 7.57 (d, *J* = 8.1 Hz, 2H), 4.32 (d, *J* = 11.0 Hz, 1H), 4.05 (td, *J* = 10.7, 3.7 Hz, 1H), 3.90 (s, 3H), 2.71 (s, 3H), 2.45 (s, 3H), 2.26 – 2.18

(m, 1H), 1.77 – 1.66 (m, 4H), 1.06 (t, *J* = 7.4 Hz, 3H); LC-MS (m/z, ES+): 437.0 $[M+H]^+$. HRMS (m/z) $[M+H]^+$ calculated for $C_{23}H_{24}O_3N_4S$, 437.16419; found 437.16515.

Methyl (*R*)-2-((*S*)-4-(4-(3-aminoprop-1-yn-1-yl)phenyl)-2,3,9-trimethyl-6*H*-thieno[3,2-*f*][1,2,4] triazolo [4,3-*a*][1,4]diazepin-6-yl)butanoate (MR101)

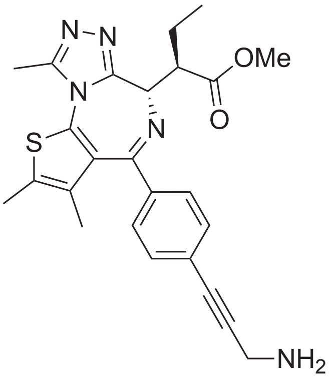

To a stirred solution of methyl (*R*)-2-((*S*)-4-(4-chlorophenyl)-2,3,9-trimethyl-6*H*-thieno[3,2-*f*][1,2,4]triazolo[4,3-*a*][1,4]diazepin-6-yl)butanoate (70 mg, 0.16 mmol) in THF (1.5 mL) was added XPhos Pd G2 (37. 3mg, 0,3 eq. 0.05 mmol), XPhos (23 mg, 0.05 mmol), cesium carbonate (258 mg, 0.79 mmol) and propargyl amine (0.10 mL, 1.58 mmol). The reaction was stirred for 3 h at 90 °C, then cooled to 60 °C and stirred overnight. Upon cooling to ambient temperature, the reaction mixture was suspended in dichloromethane and filtered through diatomaceous earth. The filtrate was washed with water, and the organic phase was dried over anhydrous magnesium sulfate and concentrated under reduced pressure. Purification by preparative reversed phase column chromatography, eluting with 5/95 to 95/5 MeCN/H_2O (0.1 % formic acid), afforded the title compound as a white solid (3 mg, 20%).

^1H NMR (500 MHz, $CDCl_3$) δ 7.36 (d, J= 8.2 Hz, 2H), 7.31 (d, J= 8.0 Hz, 2H), 4.23 (d, *J* = 10.9 Hz, 1H), 3.98 (td, *J* = 10.7, 3.6 Hz, 1H), 3.84 (s, 3H), 3.65 (s, 2H), 2.65 (s, 3H), 2.40 (s, 3H), 2.20 – 2.13 (m, 1H), 1.71 – 1.60 (m, 4H), 1.01 (t, *J* = 7.4 Hz, 3H); LC-MS (m/z, ES+): 462.1 $[M+H]^+$. HRMS (m/z): $[M+H]^+$ calculated for $C_{25}H_{27}O_2N_5S$, 462.19582 found 462.19632

Methyl 2-((6*S*)-2,3,9-trimethyl-4-(4-(oxiran-2-yl)phenyl)-6*H*-thieno[3,2-*f*][1,2,4]triazolo[4,3-*a*][1,4]diazepin-6-yl)butanoate (MR104)

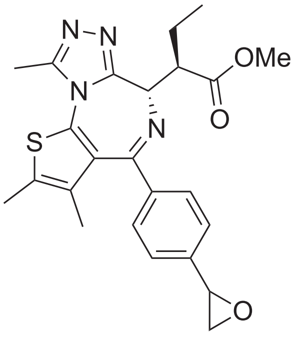

To a stirred solution of MR100 (30 mg, 0.05 mmol) in dichloromethane (1 mL) was added *m*-chloroperoxybenzoic acid (12 mg) and the reaction was stirred overnight. The reaction mixture was diluted with dichloromethane and then washed with water (2 x 50 mL) and brine (2 x 50 mL). The organic phase was dried over anhydrous magnesium sulfate and concentrated under reduced pressure. Purification by preparative reversed phase column chromatography, eluting with 5/95 to 95/5 MeCN/H_2O (0.1 % NH_4OH), afforded the title compound as a white solid (3 mg, 10%).

^1H NMR (500 MHz, $CDCl_3$) δ 7.40 – 7.31 (m, 2H), 7.23 (d, *J* = 7.6 Hz, 2H), 4.23 (d, *J* = 11.0 Hz, 1H), 3.98 (td, *J* = 10.9, 3.6 Hz, 1H), 3.86 – 3.85 (m, 1H), 3.84 (s, 3H), 3.16 – 3.13 (m, 1H), 2.74 (td, *J* = 5.8, 2.5 Hz, 1H), 2.66 (d, *J* = 2.9 Hz, 3H), 2.40 (s, 3H), 2.21 – 2.13 (m, 1H), 1.71 – 1.59 (m, 4H), 1.01 (t, *J* = 7.4 Hz, 3H); LC-MS (m/z, ES+): 451.0 $[M+H]^+$. HRMS (m/z): $[M+H]^+$ calculated for $C_{24}H_{26}N_4O_3S$, 451.17984; found 451.18087.

Methyl (*R*)-2-((*S*)-4-(4-aminophenyl)-2,3,9-trimethyl-6H-thieno[3,2-f][1,2,4]triazolo[4,3a][1,4] diazepin-6-yl)butanoate (Compound 7)

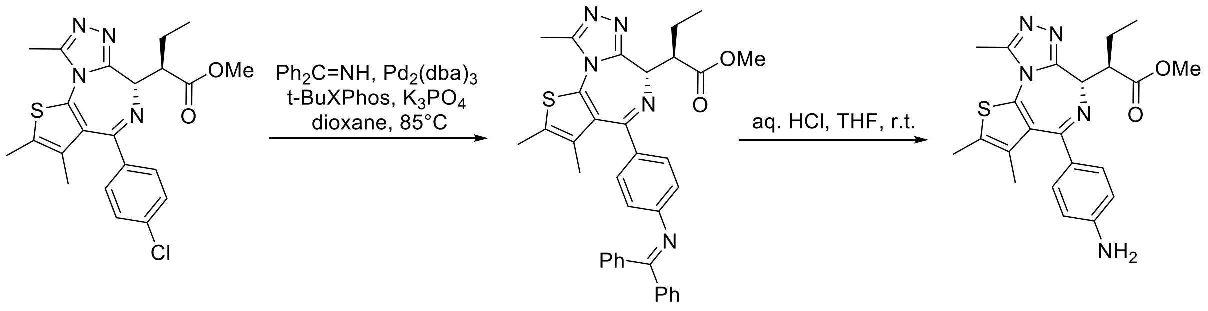

To a stirred solution of methyl (*R*)-2-((*S*)-4-(4-chlorophenyl)-2,3,9-trimethyl-6H-thieno[3,2-f][1,2,4]triazolo[4,3-a][1,4]diazepin-6-yl)butanoate (2.50 g, 5.64 mmol) in 1,4-dioxane (125 mL) was added benzophenone imine (1.22 g, 6.77 mmol) and potassium phosphate tribasic (2.99 g, 14.11 mmol), and argon (g) was bubbled through the stirring mixture for 30 minutes. After this time, *t*-BuXPhos (0.36 g, 0.85mmol) and $Pd_2(dba)_3$ (0.26 g, 0.28 mmol) were added, and after passage of argon (g) for a further 5 minutes, the reaction mixture was heated at 85 °C overnight. The reaction mixture was cooled to 55 °C and partially concentrated under reduced pressure, the subsequent residue being partitioned between water and ethyl acetate. The organic phase was separated, and the aqueous component was extracted with ethyl acetate. The combined organic extracts were washed with brine, dried over anhydrous magnesium sulfate and concentrated under reduced pressure to a brown oil. The crude imine was dissolved in THF (50 mL) and treated with 1M hydrochloric acid (50 mL), and the resulting solution was stirred at ambient temperature for 2 hours before being partially concentrated under reduced pressure. The aqueous residue was further diluted with water and washed with ethyl acetate. The aqueous phase was then adjusted to pH8-9 by slow addition of saturated aqueous $NaHCO_3$ solution and extracted with ethyl acetate (3 portions). These organic extracts were combined, dried over anhydrous magnesium sulfate and concentrated under reduced pressure to a pale-yellow foam. Purification by flash column chromatography, eluting with 1-2% 7M methanolic ammonia/dichloromethane, afforded a yellow foam. Lyophilisation from $MeCN/H_2O$ (1:2) afforded the title compound as a pale-yellow solid (1.20 g, 50%).

^1H NMR (400 MHz, $CDCl_3$) δ: 7.19 (d, *J*= 8.3 Hz, 2H), 6.58 (d, *J*= 8.8 Hz, 2H), 4.18 (d, *J*= 11.0 Hz, 1H), 3.97 (td, *J*= 10.7, 3.7 Hz, 1H), 3.83 (s, 3H), 2.64 (s, 3H), 2.40 (s, 3H), 2.22-2.11 (m, 1H), 1.74-1.72 (m, 3H), 1.68-1.59 (m, 1H), 1.01 (t, *J*= 7.4 Hz, 3H); LC-MS (m/z, ES+): 446.2 $[M+Na]^+$

Methyl (*R*)-2-((*S*)-4-(4-(2-chloroacetamido)phenyl)-2,3,9-trimethyl-6*H*-thieno[3,2-*f*][1,2,4]triazolo [4,3-*a*][1,4]diazepin-6-yl)butanoate (MR112)

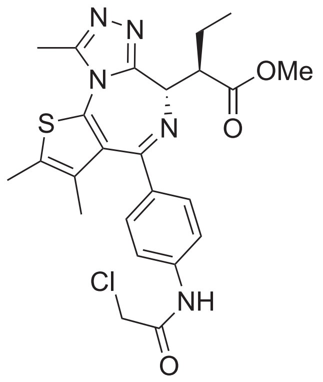

To a stirred solution of compound 7 (75 mg, 0.18 mmol) in dichloromethane (2 mL) at ambient temperature was added *N,N*-diisopropylethylamine (31 µL, 0.18 mmol), followed by the slow dropwise addition of 2-chloroacetyl chloride (043 µL, 0.53 mmol). After 2 hours, the reaction was quenched with a few drops of MeOH and concentrated under reduced pressure. The residue was partitioned between water and dichloromethane, and the organic phase was washed with water (2x50 mL) and brine (2x50 mL), dried over anhydrous magnesium sulfate and concentrated under reduced pressure. Purification by flash column chromatography, eluting with 0-5% methanol/dichloromethane, afforded the title compound as a white solid (60 mg, 67%).

^{1}H NMR (500 MHz, $CDCl_3$) δ 8.49 (s, 1H), 7.51 (d, *J* = 9.0 Hz, 2H), 7.31 (d, *J* = 8.6 Hz, 2H), 4.17 (d, *J* = 11.0 Hz, 1H), 4.12 (s, 2H), 3.91 (td, *J* = 10.7, 3.7 Hz, 1H), 3.78 (s, 3H), 2.60 (s, 3H), 2.34 (s, 3H), 2.13 – 2.05 (m, 1H), 1.65 – 1.53 (m, 4H), 0.95 (t, *J* = 7.4 Hz, 3H); ^{13}C NMR (126 MHz, $CDCl_3$) δ 175.4, 164.1, 163.4, 154.6, 149.8, 139.0, 134.5, 131.8, 131.1, 130.8, 130.8, 129.5, 119.4, 59.3, 51.6, 49.7, 43.0, 23.3, 14.5, 13.2, 11.8, 11.7; LC-MS (m/z, ES+): 500.1 $[M+H]^+$; HRMS (m/z) [M+H]+ calculated for $C_{24}H_{26}ClN_5O_3S$, 500.15176; found 500.15278.

Methyl (*R*)-2-((*S*)-2,3,9-trimethyl-4-(4-(vinylsulfonamido)phenyl)-6*H*-thieno[3,2-*f*][1,2,4]triazolo[4,3-*a*][1,4]diazepin-6-yl)butanoate (MR121)

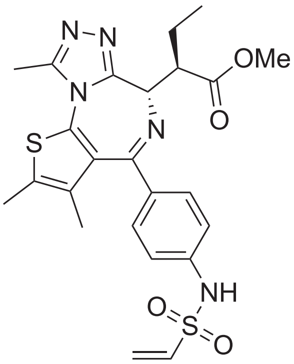

To a stirred solution of compound 7 (75 mg, 0.18 mmol) and pyridine (31 µL, 0.18 mmol) in dichloromethane (1.9 mL) at 0 °C was dropwise added vinyl sulfonyl chloride (17 µL, 0.18 mmol). The reaction mixture was stirred for 5 minutes before being quenched with a few drops of methanol and concentrated under reduced pressure. The residue was partitioned between dichloromethane and water, and the organic phase was washed with water (2x50 mL) and brine (2x50 mL), dried over anhydrous magnesium sulfate and concentrated under reduced pressure. Purification by flash column chromatography, eluting with 0-5% methanol/dichloromethane, afforded the title compound as a white crystalline solid (20 mg, 30%).

^{1}H NMR (500 MHz, $CDCl_3$) δ 7.45 (s, 1H), 7.35 (d, J = 8.2 Hz, 2H), 7.19 (d, J = 8.9 Hz, 2H), 6.53 (dd, J = 16.5, 9.9 Hz, 1H), 6.27 (d, J = 16.6 Hz, 1H), 5.95 (d, J = 9.9 Hz, 1H), 4.25 (d, J = 11.0 Hz, 1H), 3.99 (td, J = 10.7, 3.7 Hz, 1H), 3.86 (s, 3H), 2.69 (s, 3H), 2.43 (s, 3H), 2.24 – 2.12 (m, 1H), 1.74 – 1.61 (m, 4H), 1.04 (t, J = 7.4 Hz, 3H); ^{13}C NMR (126 MHz, $CDCl_3$) δ 175.4, 163.3, 154.5, 149.8, 138.8, 135.0, 134.4, 131.9, 131.0, 130.8, 130.7, 129.8, 128.7, 119.5, 76.8, 59.3, 51.6, 49.8, 23.2, 14.4, 13.2, 11.8, 11.7; LC-MS (m/z, ES+): 514.0 $[M+H]^+$; HRMS (m/z) $[M+H]^+$ calculated for $C_{24}H_{27}N_5O_4S_2$, 514.15772 found 514.15910.

(*R*)-2-((*S*)-4-(4-Acrylamidophenyl)-2,3,9-trimethyl-6*H*-thieno[3,2-*f*][1,2,4]triazolo[4,3-*a*][1,4]diazepin-6-yl)butanoate (MR116)

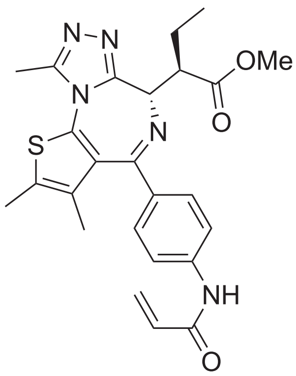

To a stirred solution of compound 7 (1 equiv., 75 mg, 0.18 mmol) and triethylamine (0.03 mL, 0.21 mmol) in dichloromethane (1.9 mL) at ambient temperature was dropwise added acryloyl chloride (0.021 mL, 0.27 mmol), and the reaction mixture was stirred for 30 minutes. The reaction mixture was diluted with dichloromethane and then washed with water (2 x 50 mL) and brine (2 x 50 mL), dried over anhydrous magnesium sulfate and concentrated under reduced pressure. Purification by flash column chromatography, eluting with 0-5% methanol/dichloromethane, afforded the title compound as a yellow solid (60 mg, 71 %).

^{1}H NMR (500 MHz, $CDCl_3$) δ 8.46 (s, 1H), 7.58 (d, *J* = 8.3 Hz, 2H), 7.26 (d, *J* = 8.8 Hz, 2H), 6.36 (dd, *J* = 16.9, 1.6 Hz, 1H), 6.27 (dd, *J* = 16.8, 9.9 Hz, 1H), 5.65 (dd, *J* = 9.9, 1.7 Hz, 1H), 4.16 (d, *J* = 10.9 Hz, 1H), 3.89 (td, *J* = 10.8, 3.7 Hz, 1H), 3.76 (s, 3H), 2.58 (s, 3H), 2.33 (s, 3H), 2.12 – 2.04 (m, 1H) , 1.58 (m, 4H), 0.93 (t, *J* = 7.4 Hz, 3H); ^{13}C NMR (126 MHz, $CDCl_3$) δ 175.4, 163.9, 163.6, 154.7, 149.8, 140.5, 133.6, 131.6, 131.3, 131.1, 131.0, 130.8, 129.4, 128.2, 119.4, 59.3, 51.6, 49.8, 23.3, 14.5, 13.2, 11.8, 11.7 ; LC-MS (m/z, ES+): 478.1 $[M+H]^+$. HRMS (m/z) $[M+H]^+$ calculated for $C_{25}H_{27}N_5O_3S$, 478.19074 found 478.19131

(2-Amino-4,5-dimethylthiophen-3-yl)(3-chlorophenyl)methanone

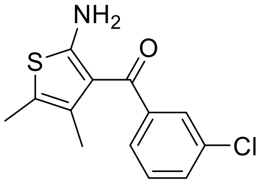

To a stirred suspension of 3-chlorobenzoylacetonitrile (40.00 g, 222.71 mmol) in ethanol (900 mL) was added methyl ethyl ketone (24.94 mL, 278.39 mmol), morpholine (3.90 mL, 44.54 mmol) and then sulfur (62.84 g, 244.98 mmol), and the resulting reaction mixture was stirred at 70 °C overnight. Upon cooling to ambient temperature, the reaction mixture was poured into brine (2500 mL) and extracted with ethyl acetate (3 x 1000 mL). The combined organic extracts were washed with brine (1000 mL), dried over anhydrous magnesium sulfate and concentrated under reduced pressure to an orange oil. Trituration with *tert*-butyl methyl ether overnight at ambient temperature afforded a yellow solid (16.91 g). Purification of the mother liquors by dry flash chromatography, eluting with 0-20% ethyl acetate/dichloromethane followed by trituration with *tert*-butyl methyl ether and petroleum ether (40:60) afforded a second crop of crude product (6.11 g). The combined material was converted to the oxalic acid salt by treatment with oxalic acid dihydrate (10.90 g, 86.6 mmol) in methanol (200 mL) and water (60 mL), and after concentration under reduced pressure, the salt was recrystallised from refluxing acetonitrile (330 mL) to give a crystalline solid (16.35 g). Treatment with 0.5M sodium hydroxide (aq.) (400 mL), dichloromethane (300mL) and *tert*-butyl methyl ether (400 mL) gave a biphasic mixture. The organic component was separated, and the aqueous phase extracted with *tert*-butyl methyl ether (2 x 200mL). The combined organic extracts were dried over anhydrous magnesium sulfate and concentrated under reduced pressure to afford the title compound as a green solid (13.59 g, 23%).

^{1}H NMR (400 MHz, $CDCl_3$): δ 7.52-7.32 (m, 4H), 2.13 (d, *J*= 0.7 Hz, 3H), 1.54 (d, *J*= 0.7 Hz, 3H); LC-MS (m/z, ES+): 266.1 $[M+H]^+$.

Methyl (*R*)-2-((*S*)-5-(3-chlorophenyl)-6,7-dimethyl-2-oxo-2,3-dihydro-1H-thieno[2,3-e][1,4]diazepin-3-yl)butanoate

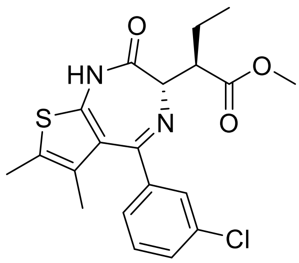

To a stirred suspension of (2-amino-4,5-dimethylthiophen-3-yl)(3-chlorophenyl)methanone (8.59 g, 32.31 mmol) in toluene (40 mL) at ambient temperature was added freshly dried and activated 4Å molecular sieves (30 g) followed by TFA (4.80 mL, 64.52 mmol). The resulting red solution was stirred for 5 minutes, after which time a solution of methyl (*R*)-2-((*S*)-2,5-dioxooxazolidin-4-yl)butanoate (6.50 g, 32.31 mmol) in toluene (20 mL) was added dropwise, and the reaction mixture was heated at 60 °C for 2.5 hours. Triethylamine (13.51 mL, 96.93 mmol) was added, and the reaction mixture was heated at 80 °C overnight. Upon cooling to ambient temperature, the reaction mixture was filtered, the filter cake being washed with dichloromethane, and the filtrate was concentrated under reduced pressure. The residue was partitioned between saturated aqueous sodium hydrogen carbonate solution and dichloromethane. The organic phase was separated, and the aqueous component was extracted with dichloromethane. The combined organic extracts were washed with brine, dried over anhydrous magnesium sulfate and concentrated under reduced pressure. Purification by flash column chromatography, eluting with 0-10% ethyl acetate/dichloromethane, and then a for a second time eluting with 2-10% ethyl acetate/cyclohexane, afforded the title compound as a yellow solid (5.20 g, 38%).

^{1}H NMR (400 MHz, $CDCl_3$): δ 8.63 (br s, 1H), 7.42-7.36 (m, 2H), 7.29-7.22 (m, 2H), 3.83 (s, 3H), 3.71-3.61 (m, 1H), 2.30 (d, *J*= 0.5 Hz, 3H), 1.96-1.85 (m, 1H), 1.65-1.53 (m, 5H), 1.02 (t, *J*= 7.4 Hz, 3H); LC-MS (m/z, ES+): 405.0 $[M+H]^+$.

Methyl (*R*)-2-((*S*)-4-(3-chlorophenyl)-2,3,9-trimethyl-6H-thieno[3,2-f][1,2,4]triazolo[4,3-a][1,4] diazepin-6-yl)butanoate (Compound 6)

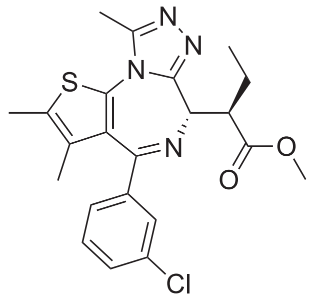

To a stirred solution of methyl (*R*)-2-((*S*)-5-(3-chlorophenyl)-6,7-dimethyl-2-oxo-2,3-dihydro-1H-thieno[2,3-e][1,4]diazepin-3-yl)butanoate (5.00 g, 12.35 mmol) in THF (60 mL) at -78 °C was dropwise added potassium *tert*-butoxide (24.70 mL, 24.70 mmol, 1M solution in THF), and stirring was maintained at this temperature for 30 minutes. Diethyl chlorophosphate (3.55 mL, 24.70 mmol) was added dropwise, and upon completion of the addition, the reaction mixture was allowed to warm to

ambient temperature over 90 minutes, with an additional portion of THF (8 mL) being added during this time. Acetohydrazide (2.74 g, 37.05 mmol) was added portion-wise, and stirring was maintained at ambient temperature for 1 hour. *n*-Butanol (90 mL) was added and the reaction mixture was heated at 90 °C overnight, before being re-cooled to ambient temperature and concentrated under reduced pressure. The residue was partitioned between saturated aqueous sodium hydrogen carbonate solution and dichloromethane. The organic phase was separated, and the aqueous component was extracted with dichloromethane. The combined organic extracts were washed with brine, dried over anhydrous magnesium sulfate and concentrated under reduced pressure. Purification by flash column chromatography, eluting with 5-75% ethyl acetate/heptane, followed by re-concentration from cyclohexane/*tert*-butyl methyl ether mixture and drying under high vacuum at 60 °C afforded the title compound as a beige solid (2.80 g, 51%).

^{1}H NMR (400 MHz, $CDCl_3$): δ 7.40-7.36 (m, 2H), 7.30-7.23 (m, 2H), 4.25 (d, *J*= 11.0 Hz, 1H), 3.99 (td, *J*= 10.7, 3.7 Hz, 1H), 3.87 (s, 3H), 2.68 (s, 3H), 2.42 (d, *J*= 0.6 Hz, 3H), 2.23-2.11 (m, 1H), 1.74-1.61 (m, 4H), 1.02 (t, *J*= 7.4 Hz, 3H); LC-MS (m/z, ES+): 443.2 $[M+H]^+$.

Methyl (*R*)-2-((*S*)-2,3,9-trimethyl-4-(3-vinylphenyl)-6*H*-thieno[3,2-*f*][1,2,4]triazolo[4,3-*a*][1,4] diazepin-6-yl)butanoate (MR108)

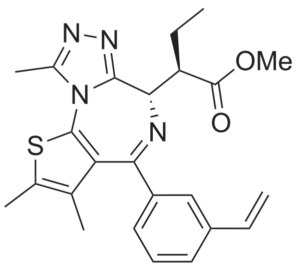

A solution of compound **6** (97 mg, 0.72 mmol), XPhos Pd G2 (57 mg, 0.07 mmol) and *N,N*-diisopropylethylamine (0.17 mL, 0.96 mmol) in DMF (2 mL) and water (0.5 mL) was heated at 100 °C for 24 hours. Upon cooling to ambient temperature, the mixture was filtered through diatomaceous earth, the filtrate was concentrated under reduced pressure and the residue was and redissolved in dichloromethane. The solution was washed with water (2 x 50 mL) and brine (20 x 50 mL), dried over anhydrous magnesium sulfate and concentrated under reduced pressure. Purification by flash column chromatography, eluting with 0-12% methanol/dichloromethane, afforded the title compound as a yellow solid (90 mg, 91 %).

^{1}H NMR (500 MHz, $CDCl_3$) δ 7.45 (d, *J* = 7.8 Hz, 1H), 7.42 (s, 1H), 7.28 (t, *J* = 7.6 Hz, 1H), 7.20 (d, *J* = 7.6 Hz, 1H), 6.68 (dd, *J* = 17.6, 10.9 Hz, 1H), 5.72 (dd, *J* = 17.7, 0.8 Hz, 1H), 5.26 (d, *J* = 10.8 Hz, 1H), 4.24 (d, *J* = 11.0 Hz, 1H), 4.01 (td, *J* = 10.8, 3.6 Hz, 1H), 3.87 (s, 3H), 2.67 (s, 3H), 2.40 (s, 3H), 2.22 – 2.14 (m, 1H), 1.71 – 1.61 (m, 4H), 1.03 (t, *J* = 7.4 Hz, 3H); ^{13}C NMR (126 MHz, $CDCl_3$) δ 175.6, 164.0, 154.5, 149.7, 138.4, 137.8, 136.3, 132.0, 131.2, 130.8, 130.5, 128.5, 128.1, 128.0, 126.3, 114.6, 59.4, 51.5, 49.7, 23.3, 14.4, 13.1, 11.9, 11.7; LC-MS (m/z, ES+): 435.3 $[M+H]^+$; HRMS (m/z) $[M+H]^+$ calculated for $C_{24}H_{26}N_4O_2S$, 435.18492; found 435.18543.

Methyl (*R*)-2-((*S*)-4-(3-(3-aminoprop-1-yn-1-yl)phenyl)-2,3,9-trimethyl-6*H*-thieno[3,2-*f*][1,2,4]triazolo[4,3-*a*][1,4]diazepin-6-yl)butanoate (MR109)

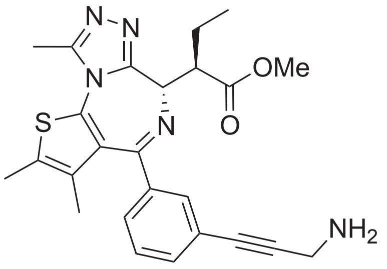

A stirred suspension of compound **6** (50 mg, 0.11 mmol), XPhos (16 mg, 0.03 mmol) , cesium carbonate (184 mg, 0.57 mmol), XPhos Pd G2 (27 mg, 0.03 mmol) and propargylamine (0.07 mL, 1.13 mmol) in THF (1.5mL) was heated at 100 °C for 1 hour, 90 °C for 2 hours, and then cooled to 60 °C and stirred overnight. Upon cooling to ambient temperature, the mixture was suspended in dichloromethane and filtered through diatomaceous earth. The filtrate was washed with water, dried over anhydrous magnesium sulfate and concentrated under reduced pressure. Purification by preparative reversed phase column chromatography, eluting with 5/95 to 95/5 MeCN/H_2O (0.1 % formic acid), afforded the title compound as a white solid (7 mg, 13%).

^{1}H NMR (500 MHz, $CDCl_3$) δ 7.36 (d, *J* = 8.2 Hz, 2H), 7.31 (d, *J* = 8.0 Hz, 2H), 4.23 (d, *J* = 10.9 Hz, 1H), 3.98 (td, *J* = 10.7, 3.6 Hz, 1H), 3.84 (s, 3H), 3.65 (s, 2H), 2.65 (s, 3H), 2.40 (s, 3H), 2.17 – 2.05 (m, 1H), 1.62 – 1.55 (m, 4H), 1.01 (t, *J* = 7.4 Hz, 3H); LC-MS (m/z, ES+): 462.1 $[M+H]^+$. HRMS (m/z) $[M+H]^+$ calculated for $C_{25}H_{27}N_5O_2S$, 462.19582; found 462.19629

Methyl (*R*)-2-((*S*)-4-(3-formylphenyl)-2,3,9-trimethyl-6*H*-thieno[3,2-*f*][1,2,4]triazolo[4,3-*a*][1,4] diazepin-6-yl)butanoate (MR115)

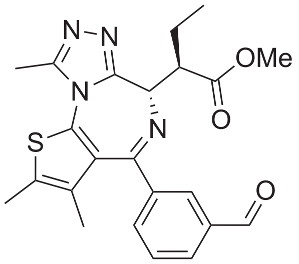

To a stirred solution of methyl MR108 (29 mg, 0.07 mmol) and sodium periodate (42 mg, 0.19 mmol) in acetone (0.5 mL) and water (0.1 mL) was added osmium tetroxide (21 µL, 4%w/v solution in water). After stirring for 2 hours, the reaction mixture was diluted with EtOAc. The solution was washed with water, and the organics were dried over anhydrous magnesium sulfate and concentrated under reduced pressure. Purification by preparative reversed phase column chromatography, eluting with 5/95 to 95/5 MeCN/H_2O (0.1 % NH_4OH) afforded the title compound as a white solid (28 mg, quant).

^{1}H NMR (500 MHz, $CDCl_3$) δ 10.00 (s, 1H), 7.92 (d, *J* = 7.6 Hz, 1H), 7.84 (s, 1H), 7.72 (d, *J* = 7.8 Hz, 1H), 7.54 (t, *J* = 7.7 Hz, 1H), 4.28 (d, *J* = 11.1 Hz, 1H), 4.01 (td, *J* = 10.7, 3.5 Hz, 1H), 3.89 (d, *J* = 1.6 Hz, 3H), 2.69 (s, 3H), 2.42 (s, 3H), 2.19 – 2.16 (m, 1H), 1.69 (dt, *J* = 9.9, 6.4 Hz, 1H), 1.65 (s, 3H), 1.03 (t, *J* = 7.3 Hz, 3H); ^{13}C NMR (126 MHz, $CDCl_3$) δ 191.7, 175.5, 163.1, 154.3, 149.9, 148.0, 139.1, 136.4, 134.2, 132.0, 131.1, 130.6, 130.1, 129.3, 129.2, 59.5, 51.7, 49.7, 23.3, 14.6, 13.2, 11.9, 11.6; LC-MS (m/z, ES+): 437.2 $[M+H]^+$. HRMS (m/z) $[M+H]^+$ calculated for $C_{23}H_{24}N_4O_3S$, 437.16419; found 437.16505

methyl (2*R*)-2-((6*S*)-2,3,9-trimethyl-4-(3-(oxiran-2-yl)phenyl)-6*H*-thieno[3,2-*f*][1,2,4]triazolo[4,3-*a*][1,4]diazepin-6-yl)butanoate (MR111)

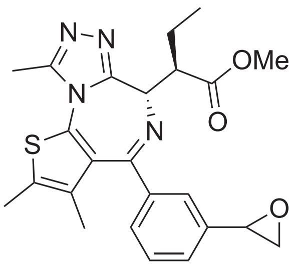

To a stirred solution of MR108 (30 mg, 0.07 mmol) in dichloromethane (1 mL) at ambient temperature was added *m*-chloroperoxybenzoic acid (1 eq., 12 mg, 0.07 mmol) and the reaction stirred overnight. The mixture was diluted with dichloromethane (50 mL), washed with water (2 x 50 mL) and brine (2 x 50 mL), and the organic phase was dried over anhydrous magnesium sulfate and concentrated under reduced pressure. Purification by preparative reversed phase column chromatography, eluting with 5/95 to 95/5 $MeCN/H_2O$ (0.1% NH_4OH), afforded the title compound as a white solid (3 mg, 7%). ^{1}H NMR (500 MHz, $CDCl_3$) δ 7.27 – 7.18 (m, 4H), 4.20 – 4.14 (m, 1H), 3.98 – 3.89 (m, 1H), 3.82 – 3.75 (m, 4H), 3.09 – 3.02 (m, 1H), 2.72 – 2.66 (m, 1H), 2.65 – 2.58 (m, 3H), 2.34 (s, 3H), 2.15 – 2.06 (m, 1H), 1.65 – 1.52 (m, 4H), 0.95 (t, *J* = 7.4 Hz, 3H) ; LC-MS (m/z, ES+): 451.2 $[M+H]^+$. HRMS (m/z) $[M+H]^+$ calculated for $C_{24}H_{26}N_4O_3S$, 451.17984; found 451.18083.

Methyl (*R*)-2-((*S*)-4-(3-aminophenyl)-2,3,9-trimethyl-6H-thieno[3,2-f][1,2,4]triazolo[4,3-a][1,4]diazepin-6-yl)butanoate (Compound 8)

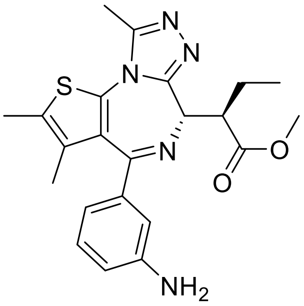

To a stirred solution of compound **6** (1.60 g, 3.61 mmol) in 1,4-dioxane (50 mL) was added benzophenone imine (0.79 g, 4.33 mmol) and potassium phosphate tribasic (1.92 g, 9.03 mmol), and the reaction mixture was degassed and back-filled with argon (g). *t*-BuXPhos (0.23 g, 0.54 mmol) and $Pd_2(dba)_3$ (0.17 g, 0.18 mmol) were added, and after further degassing with back-filling of argon (g), the reaction mixture was heated at 85 °C overnight. The reaction mixture was cooled to 55 °C and partially concentrated under reduced pressure, the subsequent residue being partitioned between water and ethyl acetate. The organic phase was separated, and the aqueous component was extracted with ethyl acetate. The combined organic extracts were washed with brine, dried over anhydrous magnesium sulfate and concentrated under reduced pressure to a brown oil. The crude imine was dissolved in THF (30 mL) and treated with 1M hydrochloric acid (30 mL), and the resulting solution was stirred at ambient temperature for 2 hours before being partially concentrated under reduced pressure. The aqueous residue was further diluted with water and washed with ethyl acetate. The aqueous phase was then adjusted to pH8-9 by slow addition of saturated aqueous sodium hydrogen carbonate solution and extracted with ethyl acetate (3 portions). These organic extracts were combined, dried over anhydrous magnesium sulfate and concentrated under reduced pressure. Purification by flash column chromatography, eluting with 0-2.5% 7M methanolic ammonia/dichloromethane, afforded a yellow foam. This process was repeated, followed by trituration with *tert*-butyl methyl ether/cyclohexane, affording the title compound as a yellow solid (0.76 g, 50%). ^{1}H NMR (400 MHz, $CDCl_3$): δ 7.10 (t, *J*= 7.8 Hz, 1H), 6.75-6.69 (m, 2H), 6.66 (d, *J*= 7.6 Hz, 1H), 4.22 (d, *J*= 11.0 Hz, 1H), 3.99 (td, *J*= 10.7, 3.7 Hz, 1H), 3.84 (s, 3H), 2.66 (s, 3H), 2.40 (s, 3H), 2.24-2.13 (m, 1H), 1.73-1.61 (m, 4H), 1.01 (t, *J*= 7.4 Hz, 3H); LC-MS (m/z, ES+): 446.2 $[M+Na]^+$.

methyl (*R*)-2-((*S*)-4-(3-(2-chloroacetamido)phenyl)-2,3,9-trimethyl-6*H*-thieno[3,2-*f*][1,2,4]triazolo[4,3-*a*][1,4]diazepin-6-yl)butanoate (MR118)

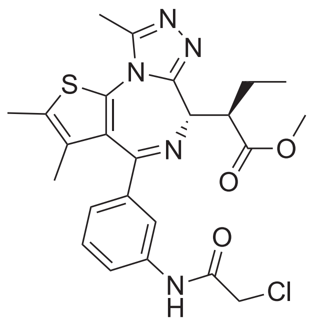

To a stirred solution of compound 8 (60 mg, 0.14 mmol) and *N*,*N*-diisopropylethylamine (250 μL, 0.14 mmol) in dichloromethane (1.4 mL) at ambient temperature was dropwise added 2-chloroacetyl chloride (430 μL, 0.53 mmol). The reaction mixture was stirred for 2 hours before being quenched with a few drops of methanol and then concentrated under reduced pressure. The residue was partitioned between dichloromethane and water, and the organic phase was separated. The organics were washed with water (2 x 50 mL) and brine (2 x 50 mL), dried over anhydrous magnesium sulfate, and concentrated under reduced pressure. Purification by flash column chromatography, eluting with 0-5% methanol/dichloromethane, afforded the title compound as a white solid (50 mg, 71%).

^{1}H NMR (500 MHz, $CDCl_3$) δ 8.46 (s, 1H), 7.66 (d, *J* = 9.5 Hz, 1H), 7.58 (s, 1H), 7.30 (t, *J* = 7.9 Hz, 1H), 7.11 (d, *J* = 7.7 Hz, 1H), 4.24 (d, *J* = 11.0 Hz, 1H), 4.17 (s, 2H), 3.98 (td, *J* = 10.7, 3.7 Hz, 1H), 3.84 (s, 3H), 2.66 (s, 3H), 2.41 (s, 3H), 2.16-2.10 (m, 1H), 1.73 – 1.59 (m, 4H), 1.00 (t, *J* = 7.4 Hz, 3H) ; ^{13}C NMR (126 MHz, $CDCl_3$) δ 175.5, 164.1, 163.9, 154.5, 149.9, 139.1, 137.2, 132.1, 131.2, 130.8, 130.63, 129.3, 125.5, 122.3, 120.0, 59.5, 51.8, 49.8, 43.0, 23.3, 14.6, 13.3, 12.0, 11.8. LC-MS (m/z, ES+): 500.4 $[M+H]^+$; HRMS (m/z) $[M+H]^+$ calculated for $C_{24}H_{26}ClN_5O_3S$ 500.15176 found 500.15287.

methyl (*R*)-2-((*S*)-2,3,9-trimethyl-4-(3-(vinylsulfonamido)phenyl)-6*H*-thieno[3,2-*f*][1,2,4]triazolo[4,3-*a*][1,4]diazepin-6-yl)butanoate (MR117)

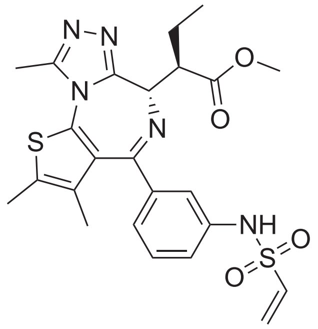

To a stirred solution of compound 8 (60 mg, 0.14 mmol) and pyridine (230 μL, 0.28 mmol) in dichloromethane (1.4 mL) at 0 °C was dropwise added vinyl sulfonyl chloride (120 μL, 0.12 mmol). The reaction mixture was stirred for 2 hours before being quenched with a few drops of methanol and then concentrated under reduced pressure. The residue was partitioned between dichloromethane and water, and the organic phase was separated. The organics were washed with water (2 x 50 mL) and brine (2 x 50 mL), dried over anhydrous magnesium sulphate, and concentrated under reduced pressure. Purification by flash column chromatography, eluting with 0-5% methanol/dichloromethane, afforded the title compound as a white crystalline solid (43 mg, 54%).

^{1}H NMR (500 MHz, $CDCl_3$) δ 7.43 (s, 1H), 7.31 – 7.20 (m, 2H), 7.08 (d, *J* = 7.1 Hz, 1H), 6.52 (dd, *J* = 16.5, 9.9 Hz, 1H), 6.25 (d, *J* = 16.6 Hz, 1H), 5.93 (d, *J* = 9.9 Hz, 1H), 5.29 (s, 1H), 4.25 (d, *J* = 11.0 Hz, 1H), 3.96 (td, *J* = 10.7, 3.7 Hz, 1H), 3.85 (s, 3H), 2.66 (s, 3H), 2.40 (s, 3H), 2.23 – 2.01 (m, 1H), 1.73 – 1.50 (m, 4H), 1.01 (t, *J* = 7.4 Hz, 3H); ^{13}C NMR (126 MHz, $CDCl_3$) δ 175.3, 163.6, 154.4, 149.9, 139.4, 136.9, 135.0, 132.0, 131.0, 130.8, 130.5, 129.4, 128.9, 125.2, 122.3, 120.1, 59.4, 51.8, 49.8, 23.2, 14.4, 13.1, 11.8, 11.7; LC-MS (m/z, ES+): 514.0 $[M+H]^+$; HRMS (m/z) $[M+H]^+$ calculated for $C_{24}H_{27}N_5O_4S_2$, 514.15772; found 514.15898.

methyl (*R*)-2-((*S*)-4-(3-acrylamidophenyl)-2,3,9-trimethyl-6*H*-thieno[3,2-*f*][1,2,4]triazolo[4,3-*a*][1,4]diazepin-6-yl)butanoate (MR119)

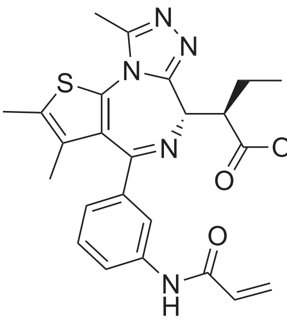

To a stirred solution of compound 8 (60 mg, 0.14 mmol) and triethylamine (240 µL, 0.20 mmol) in dichloromethane (1.4 mL) at ambient temperature was dropwise added acryloyl chloride (100 µL, 0.13 mmol). The reaction mixture was stirred for 2 hours before being quenched with a few drops of methanol and then concentrated under reduced pressure. The residue was partitioned between dichloromethane and water, and the organic phase was separated. The organics were washed with water (2 x 50 mL) and brine (2 x 50 mL), dried over anhydrous magnesium sulfate, and concentrated under reduced pressure. Purification by flash column chromatography, eluting with 0-5% methanol/dichloromethane, afforded the title compound as a yellow solid (40 mg, 59%). [1]

H NMR (500 MHz, $CDCl_3$) δ 7.70 – 7.63 (m, 2H), 7.51 (s, 1H), 7.22 (t, *J* = 7.9 Hz, 1H), 7.02 (d, *J* = 7.7 Hz, 1H), 6.34 (dd, *J* = 16.8, 1.3 Hz, 1H), 6.19 (dd, *J* = 16.9, 10.2 Hz, 1H), 5.68 (dd, *J* = 10.2, 1.3 Hz, 1H), 4.18 (d, *J* = 11.0 Hz, 1H), 3.91 (td, *J* = 10.7, 3.7 Hz, 1H), 3.77 (s, 3H), 2.59 (s, 3H), 2.34 (s, 3H), 2.17 – 2.05 (m, 1H), 1.64 – 1.56 (m, 4H), 0.94 (t, *J* = 7.4 Hz, 3H); ^{13}C NMR (126 MHz, $CDCl_3$) δ 175.3, 172.2, 164.0, 154.5, 149.8, 138.1, 131.9, 131.2, 130.7, 130.7, 129.1, 128.0, 124.8, 122.2, 119.7, 59.3, 51.7, 49.7, 23.2, 14.5, 13.2, 11.9, 11.7; LC-MS (m/z, ES+): 478.1 $[M+H]^+$. HRMS (m/z) $[M+H]^+$ calculated for $C_{25}H_{27}N_5O_3S$, 478.19074; found 478.19158.

methyl (*R*)-2-((*S*)-4-(4-((*tert*-butoxycarbonyl)amino)phenyl)-2,3,9-trimethyl-6*H*-thieno[3,2-*f*][1,2,4]triazolo[4,3-*a*][1,4]diazepin-6-yl)butanoate (MR126)

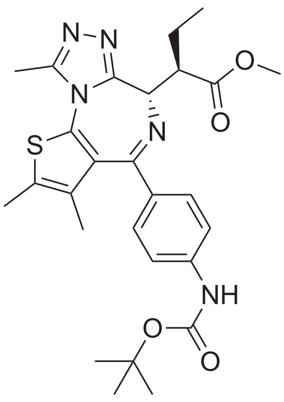

To a stirred solution of compound 7 (100 mg, 1 eq., 0.24 mmol) in methanol at ambient temperature (1.2mL) was added Boc anhydride (1.5 eq, 27 mg, 0.36 mmol) and TEA (2 eq, 66 µL, 0.47 mmol) and the reaction was stirred at 50 °C overnight. The reaction mixture was concentrated under reduced pressure and the residue was taken up in dichloromethane. The solution was washed with saturated aqueous sodium hydrogen carbonate solution (2x50 mL) and brine (2x50 mL), dried over anhydrous magnesium sulfate, and concentrated under reduced pressure. Purification by flash column chromatography, eluting with 0-5% methanol/dichloromethane, afforded the title compound as an off-white solid (90 mg, 73 %).

^{1}H NMR (500 MHz, $CDCl_3$) 7.37 (d, *J* = 8.6 Hz, 2H), 7.32 (d, *J* = 8.7 Hz, 2H), 6.81 (s, 1H), 4.22 (d, *J* = 10.9 Hz, 1H), 4.0 (td, *J* = 10.8, 3.7 Hz, 1H), 3.85 (s, 3H), 2.68 (s, 3H), 2.41 (s, 3H), 2.24 – 2.13 (m, 1H), 1.87 – 1.81 (m, 1H), 1.70 (s, 3H), 1.52 (s, 9H), 1.03 (t, *J* = 7.4 Hz, 3H); ^{13}C NMR (126 MHz, $CDCl_3$) δ 175.5, 163.5, 154.7, 152.3, 149.7, 140.6, 132.6, 131.8, 131.3, 131.0, 130.4, 129.5, 118.7, 80.97, 59.3, 51.5, 49.8, 28.3, 23.2, 14.5, 13.1, 11.9, 11.7; LC-MS (m/z, ES+): 524.5 $[M+H]^+$.

methyl (*R*)-2-((*S*)-4-(4-((*tert*-Butoxycarbonyl)amino)phenyl)-2,3,9-trimethyl-6H-thieno[3,2-f][1,2,4]triazolo[4,3-a][1,4]diazepin-6-yl)butanoic acid (MR137)

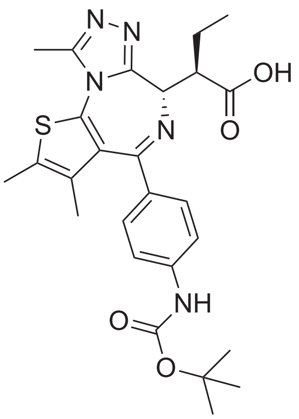

To a stirred solution of MR126 (2.15 g (uncorr.), 1.51 g (corr.), 2.87 mmol (corr.)) in THF (35 mL) and methanol (8.5 mL) was added lithium hydroxide (21.00 mL, 0.65M aqueous solution), and the resulting orange solution was heated at 45 °C for 16 hours. The volatiles were removed under reduced pressure, and the remaining aqueous was diluted with water (40 mL) and adjusted to pH 1 by addition of 2M hydrochloric acid (20 mL). The solution was extracted with dichloromethane (3 x 100 mL), and the combined organic extracts were dried over anhydrous magnesium sulfate and concentrated under reduced pressure. Purification by flash column chromatography, eluting with 0-10% methanol in dichloromethane, followed by a trituration with dichloromethane/*tert*-butyl methyl ether /heptane (1:2:2; 50 mL), afforded the title compound as a white solid (1.09 g (uncorr.), 0.87 g (corr.), 59% (corr.)).

^{1}H NMR (400 MHz, DMSO-d6) δ 12.41 (br. s, 1H), 9.58 (s, 1H), 7.48 (d, *J* = 8.9 Hz, 2H), 7.28 (d, *J* = 8.6 Hz, 2H), 4.06 (d, *J* = 10.7 Hz, 1H), 3.53 (td, *J* = 10.6, 3.5 Hz, 1H), 2.58 (s, 3H), 2.41 (d, *J* = 0.4 Hz, 3H), 2.01 – 1.90 (m, 1H), 1.63 (d, *J* = 0.4 Hz, 2H), 1.60 – 1.49 (m, 1H), 1.47 (s, 9H), 0.95 (t, *J* = 7.4 Hz, 3H)**;** LCMS purity = 92.6%; LC-MS (m/z, ES+): 510.30 $[M+H]^+$.

2-((1*E*,3*E*)-5-((*E*)-1-((*R*)-3-((*S*)-4-(4-chlorophenyl)-2,3,9-trimethyl-6*H*-thieno[3,2-*f*][1,2,4]triazolo[4,3-*a*][1,4]diazepin-6-yl)-4,18-dioxo-8,11,14-trioxa-5,17-diazatricosan-23-yl)-3,3-dimethyl-5-sulfoindolin-2-ylidene)penta-1,3-dien-1-yl)-1-ethyl-3,3-dimethyl-5-sulfo-3*H*-indol-1-ium (MR133)

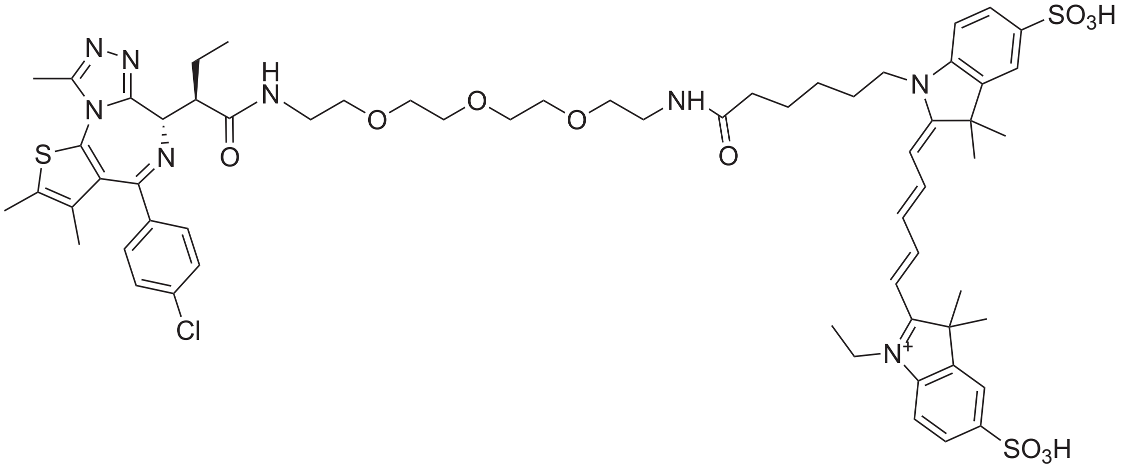

To a stirred solution of ET-JQ1-OH (1 eq, 0.02 mmol) in dichloromethane (1 mL) at ambient temperature was added a solution of Boc-amino-PEG3-amine (2, eq, 0.04 mmol), and the reaction mixture was stirred for 2 hours before being concentrated under reduced pressure. Purification by flash column chromatography, eluting with 0-20% methanol in dichloromethane, afforded *tert*-butyl ((*R*)-14-((*S*)-4-(4-chlorophenyl)-2,3,9-trimethyl-6*H*-thieno[3,2-*f*][1,2,4]triazolo[4,3-*a*][1,4]diazepin-6-yl)-13-oxo-3,6,9-trioxa-12-azahexadecyl)carbamate (10 mg, 0.0166 mmol) compound as a white powder. LC-MS (m/z, ES+): 703.3 $[M+H]^+$.

The resulting Boc-protected intermediate was dissolved in 20%TFA in DCM, after 1 hour, the solvent was evaporated and the resulting solid was dissolved in DCM and was dissolved in 20% TFA in DCM, after 1 hour, the solvent was evaporated to give the free amine. The crude amine was dissolved in DMF (2 mL) and DIPEA (3 eq, 0.06 mmol) was added. To this mixture, sulfoCy5-NHS (1 eq, 0.0166 mmol) was added annd the reaction was stirred for 2 hours at 40 C. Full conversion was observed after 2 hours. The solvent was evaporated and the crude purified by flash column chromatography, eluting with 0-5% methanol/dichloromethane, afforded the title compound as a blue solid (7 mg, 34 %).

^{1}H NMR (500 MHz, MeOD) δ 8.21 (td, *J* = 13.0, 4.2 Hz, 2H), 7.81 – 7.71 (m, 4H), 7.40 – 7.17 (m, 6H), 6.58 (t, *J* = 12.4 Hz, 1H), 6.24 (dd, *J* = 13.7, 9.9 Hz, 2H), 4.16 (d, *J* = 10.5 Hz, 1H), 4.04 (dt, *J* = 33.7, 7.2 Hz, 4H), 3.69 – 3.35 (m, 14H), 3.21 – 3.09 (m, 12H), 2.64 (s, 2H), 2.39 (s, 1H), 2.35 (s, 2H), 2.09 (t, *J* = 7.3 Hz, 2H), 1.91 – 1.80 (m, 2H), 1.71 (q, *J* = 7.6 Hz, 2H), 1.64 (d, *J* = 2.0 Hz, 9H), 1.59 (s, 2H), 1.43 (d, *J* = 7.0 Hz, 1H), 1.30 – 1.25 (m, 7H), 0.92 (t, *J* = 7.4 Hz, 3H). LC-MS (m/z, ES+): 621.9 $[M+H]^{+2}$. HRMS (m/z) $[M+H]^{+2}$ calculated for $C_{62}H_{78}ClN_8O_{11}S_3^+$, 621.7369; found 621.7367.

(2*R*)-*N*-[2-[2-[2-[2-[3-[2,2-difluoro-12-(1H-pyrrol-2-yl)-1,3λ5-diaza-2λ4-boratricyclo[7.3.0.03,7]dodeca-3,5,7,9,11-pentaen-4-yl]propanoylamino]ethoxy]ethoxy]ethoxy]ethyl]-2-[rac-(9*S*)-7-(4-chlorophenyl)-4,5,13-trimethyl-3-thia-1,8,11,12-tetrazatricyclo[8.3.0.02,6]trideca-2(6),4,7,10,12-pentaen-9-yl]butanamide (MR141)

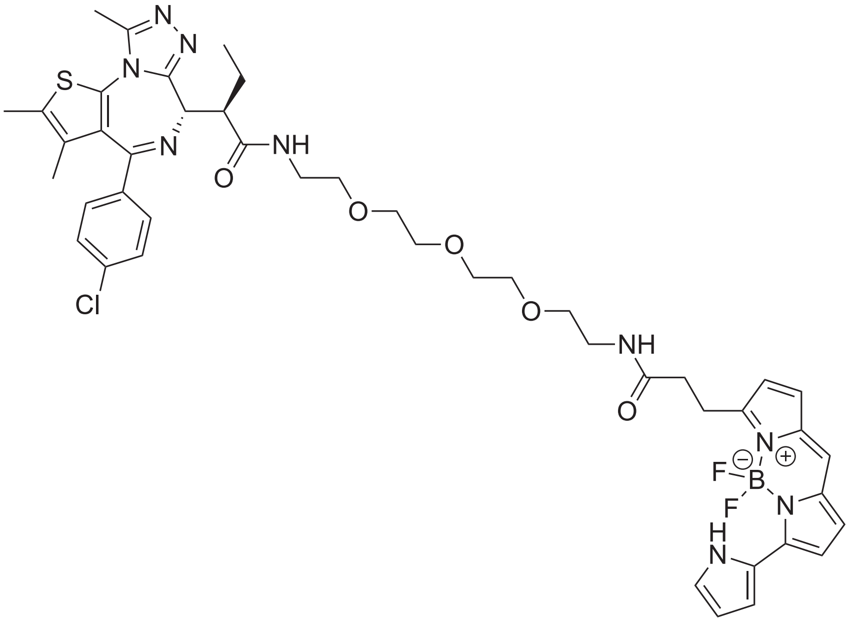

To a stirred solution of ET-JQ1-OH (0.02 mmol) in dichloromethane (1 mL) at ambient temperature was added a solution of Boc-amino-PEG_3-amine (0.04 mmol), and the reaction mixture was stirred for 2 hours before being concentrated under reduced pressure. Purification by flash column chromatography, eluting with 0-20% methanol in dichloromethane, afforded *tert*-butyl ((*R*)-14-((*S*)-4-(4-chlorophenyl)-2,3,9-trimethyl-6*H*-thieno[3,2-*f*][1,2,4]triazolo[4,3-*a*][1,4]diazepin-6-yl)-13-oxo-3,6,9-trioxa-12-azahexadecyl)carbamate (10 mg, 0.0166 mmol) compound as a white powder. LC-MS (m/z, ES+): 703.3 $[M+H]^+$.
The resulting boc protected amine (10 mg, 0.0166 mmol) was dissolved in 20% TFA in DCM, after 1 hour, the solvent was evaporated to give the free amine. The crude amine was dissolved in DMF (1 mL) and DIPEA (3 eq, 0.06) was added. To this mixture, BDP590-NHS (1 eq, 0.0166 mmol) was added and the reaction was stirred for 2 hours at 40 C. Full conversion was observed after 2 hours. The solvent was evaporated and the crude purified by flash column chromatography, eluting with 0-5% methanol/dichloromethane, afforded the title compound as a pink solid (5 mg, 66%). BDP590=BODIPY.

^{1}H NMR (500 MHz, $CDCl_3$) δ 10.33 (s, 1H), 7.30 – 7.24 (m, 2H), 7.24 – 7.19 (m, 2H), 7.10 (td, *J* = 2.7, 1.3 Hz, 1H), 6.95 (d, *J* = 4.6 Hz, 1H), 6.90 (s, 2H), 6.77 (dd, *J* = 13.2, 4.3 Hz, 3H), 6.55 (s, 1H), 6.29 (dt, *J* = 4.2, 2.4 Hz, 1H), 6.23 (d, *J* = 4.0 Hz, 1H), 5.23 (s, 2H), 4.17 (d, *J* = 10.1 Hz, 1H), 3.62 – 3.29 (m, 15H), 3.25 (t, *J* = 7.7 Hz, 2H), 2.56 (d, *J* = 7.2 Hz, 5H), 2.34 – 2.30 (m, 3H), 1.97 (ddd, *J* = 13.2, 7.5, 3.5 Hz, 1H), 1.68 – 1.55 (m, 4H), 0.95 (t, *J* = 7.4 Hz, 3H). LC-MS (m/z, ES+): 914.3 $[M+H]^+$. HRMS (m/z) $[M+Na]^+$ calculated for $C_{45}H_{51}BClF_2N_9O_5S$, 936.3383; found 936.3379.

tert-butyl (4-((6_S_)-6-((3_S_,18_R_)-3-((2_S_,4_R_)-4-hydroxy-2-((4-(4-methylthiazol-5-yl)benzyl)carbamoyl)pyrrolidine-1-carbonyl)-2,2-dimethyl-5,17-dioxo-7,10,13-trioxa-4,16-diazaicosan-18-yl)-2,3,9-trimethyl-6_H_-thieno[3,2-_f_][1,2,4]triazolo[4,3-_a_][1,4]diazepin-4-yl)phenyl)carbamate (MR162)

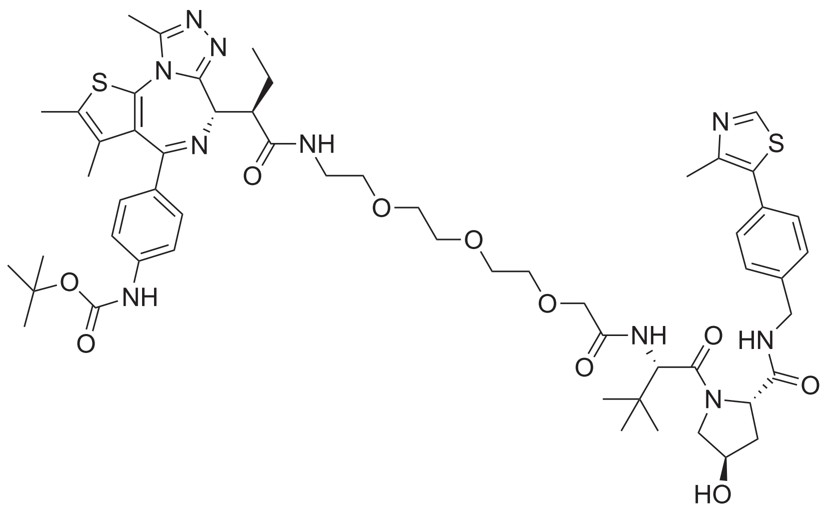

MR137 (10 mg, 20 µmol) was dissolved in anhydrous dichloromethane and _N_,_N_-diisopropylethylamine (14 µL, 80 µmol) was added, followed by HATU (7.5 mg, 20 µmol) and the resulting mixture was stirred for 5 min before adding 4-((2-(2-(2-aminoethoxy)ethoxy)ethyl)amino)-2-(2,6-dioxopiperidin-3-yl)isoindoline-1,3-dione (9.5 mg, 14 µmol). After stirring for 4 hours at ambient temperature, the reaction mixture was concentrated under reduced pressure. Purification by flash column chromatography, eluting with 0-10% methanol/dichloromethane, afforded the title compound as a white solid (15 mg, 76%).

^{1}H NMR (500 MHz, $CDCl_3$) δ 8.63 (s, 1H), 8.51 (d, _J_ = 4.4 Hz, 1H), 8.20 (d, _J_ = 8.3 Hz, 1H), 8.02 (s, 1H), 7.28 (s, 1H), 7.23 (t, _J_ = 8.8 Hz, 4H), 7.17 (d, _J_ = 4.1 Hz, 4H), 6.66 (s, 1H), 4.77 (t, _J_ = 8.2 Hz, 1H), 4.69 (d, _J_ = 9.5 Hz, 1H), 4.43 (s, 1H), 4.35 (dd, _J_ = 15.6, 7.0 Hz, 1H), 4.17 (d, _J_ = 10.2 Hz, 1H), 4.09 (d, _J_ = 10.9 Hz, 1H), 4.00 (s, 1H), 3.76 – 3.51 (m, 14H), 3.42 (s, 2H), 2.56 (s, 3H), 2.45 (s, 3H), 2.31 (s, 3H), 2.21 – 2.07 (m, 3H), 1.89 – 1.80 (m, 1H), 1.54 (s, 3H), 1.43 (s, 9H), 0.98 – 0.86 (m, 12H). LC-MS (m/z, ES+): 556.9 $[M+2H]^{2+}$

(2*S*,4*R*)-1-((2*S*,17*R*)-17-((6*S*)-4-(4-acrylamidophenyl)-2,3,9-trimethyl-6*H*-thieno[3,2-*f*][1,2,4]triazolo[4,3-*a*][1,4]diazepin-6-yl)-2-(*tert*-butyl)-4,16-dioxo-6,9,12-trioxa-3,15-diazanonadecanoyl)-4-hydroxy-*N*-(4-(4-methylthiazol-5-yl)benzyl)pyrrolidine-2-carboxamide (MR170)

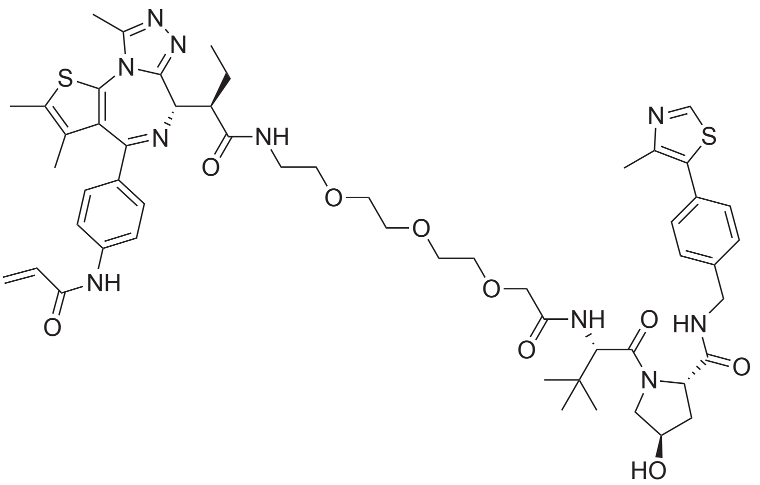

MR162 (15 mg, 14 µmol) was treated with a solution of 20% TFA in dichloromethane and then concentrated under reduced pressure. The residue was dissolved in dichloromethane (1 mL) and to this solution, *N*,*N*-diisopropylethylamine (4 µL, 22 µmol) was added followed by dropwise addition of a solution of acryloyl chloride (1 µL, 15 µmol) in dichloromethane (0.2 mL). After stirring for 30 minutes, the reaction was quenched by addition of a small amount of MeOH and then concentrated under reduced pressure. Purification by preparative reversed phase chromatography, using a gradient of 5/95 to 95/5 MeCN/H_2O (0.1% formic acid), afforded the title compound as a white solid (5 mg, 34% over two steps).

^{1}H NMR (500 MHz, $CDCl_3$) δ 8.60 (s, 1H), 7.90 (s, 1H), 7.77 (t, *J* = 5.5 Hz, 1H), 7.42 (d, *J* = 8.4 Hz, 2H), 7.29 (d, *J* = 9.3 Hz, 1H), 7.28 – 7.23 (m, 4H), 7.20 (d, *J* = 8.0 Hz, 2H), 6.35 (dd, *J* = 16.8, 1.3 Hz, 1H), 6.17 (dd, *J* = 16.9, 10.2 Hz, 1H), 5.69 (dd, *J* = 10.2, 1.3 Hz, 1H), 4.74 – 4.64 (m, 2H), 4.43 – 4.30 (m, 1H), 4.15 (d, *J* = 9.9 Hz, 1H), 4.06 – 3.97 (m, 3H), 3.87 – 3.79 (m, 1H), 3.67 – 3.47 (m, 16H), 3.47 – 3.39 (m, 1H), 2.56 (s, 3H), 2.44 (s, 3H), 2.33 – 2.29 (m, 3H), 2.27 – 2.18 (m, 1H), 2.11 – 2.01 (m, 1H), 1.90 – 1.70 (m, 1H), 1.62 – 1.52 (m, 4H), 0.97 – 0.88 (m, 12H); LC-MS (m/z, ES+): 1066.3 $[M+H]^+$**.** HRMS (m/z) $[M+H]^+$ calculated for $C_{54}H_{68}N_{10}O_9S_2$, 1065.46849; found 1065.46886.

***tert*-butyl (4-((6*S*)-6-((*R*)-4,21-dioxo-25-((3a*S*,4*S*,6a*R*)-2-oxohexahydro-1*H*-thieno[3,4-*d*]imidazol-4-yl)-8,11,14,17-tetraoxa-5,20-diazapentacosan-3-yl)-2,3,9-trimethyl-6*H*-thieno[3,2-*f*][1,2,4]triazolo[4,3-*a*][1,4]diazepin-4-yl)phenyl)carbamate (MR129)**

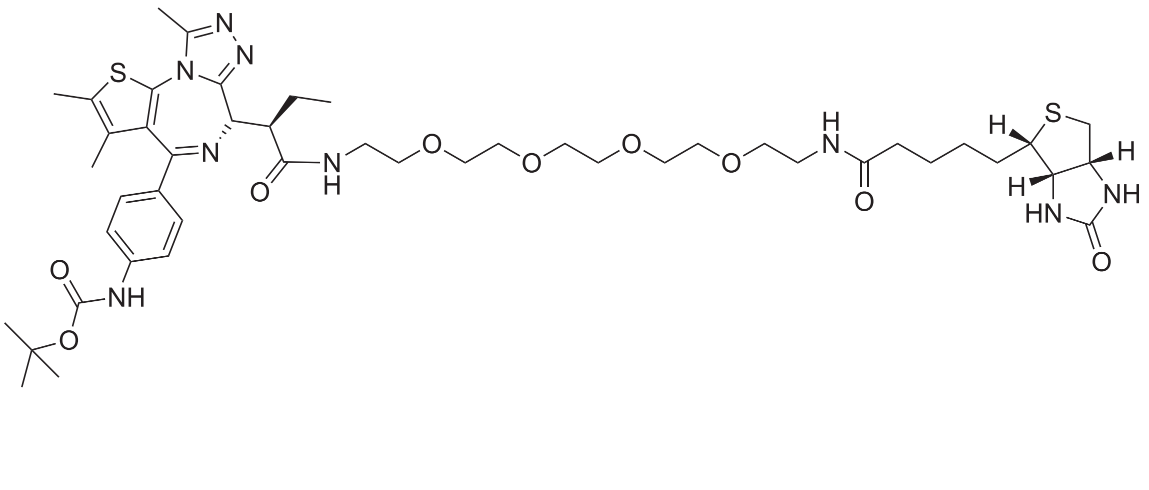

To a stirred solution of MR137 (20 mg, 40 µmol) and *N*,*N*-diisopropylethylamine (205 µL, 120 µmol) in DMF (1 mL) at ambient temperature was added HATU (18 mg, 50 µmol) and the resulting mixture was stirred for 5 min before adding *N*-(14-amino-3,6,9,12-tetraoxatetradecyl)-5-((3a*S*,4*S*,6a*R*)-2-oxohexahydro-1*H*-thieno[3,4-*d*]imidazol-4-yl)pentanamide (36 mg, 80 µmol). After stirring for 16 hours, the reaction mixture was concentrated under reduced pressure. Purification of the residue by flash column chromatography, eluting with 0-10% methanol/dichloromethane, afforded the title compound as a white solid (30 mg, 80%).

^{1}H NMR (500 MHz, $CDCl_3$) δ 8.55 (s, 1H), 7.57 (t, *J* = 5.4 Hz, 1H), 7.49 (s, 1H), 7.38 (d, *J* = 8.3 Hz, 2H), 7.31 (d, *J* = 8.4 Hz, 2H), 7.11 (t, *J* = 5.6 Hz, 1H), 6.20 (s, 1H), 5.57 (s, 1H), 4.35 (dd, *J* = 8.0, 4.9 Hz, 1H), 4.21 (d, *J* = 10.2 Hz, 1H), 4.16 (dd, *J* = 8.3, 4.6 Hz, 1H), 3.70 – 3.53 (m, 19H), 3.41 – 3.36 (m, 2H), 3.05 – 2.98 (m, 1H), 2.81 – 2.74 (m, 1H), 2.65 (s, 3H), 2.63 – 2.57 (m, 1H), 2.38 (s, 3H), 2.16 (t, *J* = 7.3 Hz, 2H), 2.03 – 1.94 (m, 1H), 1.65 (s, 4H), 1.62 – 1.52 (m, 3H), 1.48 (s, 9H), 1.39 – 1.29 (m, 2H), 1.01 (t, *J* = 7.4 Hz, 3H); LC-MS (m/z, ES+): 477.9 $[M+2H]^{2+}$.

***N*-((17*R*)-17-((6*S*)-4-(4-acrylamidophenyl)-2,3,9-trimethyl-6*H*-thieno[3,2-*f*][1,2,4]triazolo[4,3-*a*][1,4]diazepin-6-yl)-16-oxo-3,6,9,12-tetraoxa-15-azanonadecyl)-5-((3a*S*,4*S*,6a*R*)-2-oxohexahydro-1*H*-thieno[3,4-*d*]imidazol-4-yl)pentanamide (MR169)**

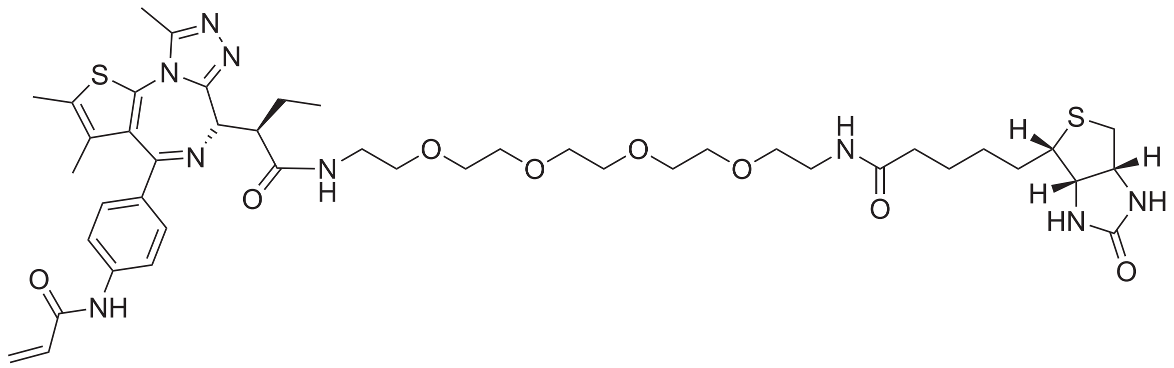

A solution of MR129 (10 mg, 10 µmol) in 20% TFA/dichloromethane (1 mL) was stirred for 30 minutes and then concentrated under reduce pressure. The residue was re-dissolved in dichloromethane (1 mL) and the solution was treated with *N,N*-diisopropylethylamine (10 µL, 50 µmol) followed by acryloyl chloride (20 µL, 20 µmol). After stirring for 2 hours, the reaction mixture was quenched by addition of a few drops of methanol and then concentrated under reduced pressure. Purification by preparative HPLC afforded the title compound as a white solid (3 mg, 28%).

^{1}H NMR (500 MHz, $CDCl_3$) δ 9.36 (s, 1H), 7.66 (s, 1H), 7.61 (d, *J* = 8.5 Hz, 2H), 7.28 (d, *J* = 8.4 Hz, 2H), 6.93 (s, 1H), 6.35 (d, *J* = 5.9 Hz, 2H), 5.85 (s, 1H), 5.63 (t, *J* = 5.9 Hz, 1H), 4.97 (s, 1H), 4.24 (d, *J* = 6.7 Hz, 1H), 4.15 (d, *J* = 10.2 Hz, 1H), 3.92 – 3.88 (m, 1H), 3.67 – 3.40 (m, 21H), 3.34 – 3.28 (m, 1H), 2.83 (q, *J* = 7.2 Hz, 1H), 2.74 – 2.66 (m, 1H), 2.60 – 2.57 (m, 3H), 2.57 – 2.50 (m, 1H), 2.32 (s, 3H), 2.07 – 2.00 (m, 2H), 1.59 (s, 3H), 1.50 – 1.42 (m, 1H), 1.38 – 1.30 (m, 2H), 1.24 – 1.15 (m, 4H), 0.95 (t, *J* = 7.4 Hz, 3H); m/z (ES+) : 908.5 $[M+H]^+$; HRMS (m/z) $[M+H]^+$ calculated for $C_{44}H_{61}N_9O_8S_2$, 908.41573; found; 908.41616.

***tert*-butyl (4-((6*S*)-2,3,9-trimethyl-6-((*R*)-14-oxo-4,7,10-trioxa-13-azaheptadec-1-yn-15-yl)-6*H*-thieno[3,2-*f*][1,2,4]triazolo[4,3-*a*][1,4]diazepin-4-yl)phenyl)carbamate (MR152)**

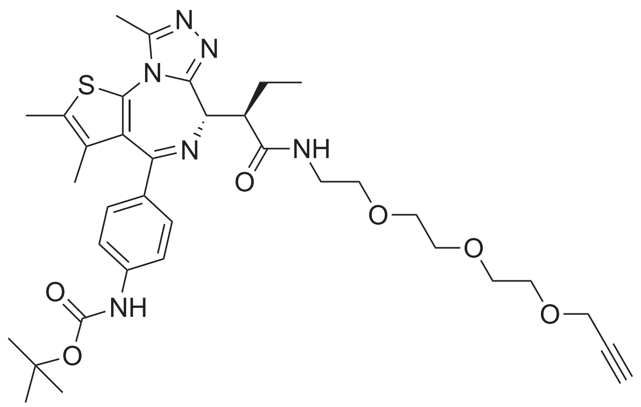

MR137 (17 mg, 0.033 mmol) was dissolved in DMF and *N*,*N*-diisopropylethylamine (23 µL, 0.13 mmol) was added, followed by HATU (7 mg, 33 mmol) and the resulting mixture was stirred for 5 min before adding 2-(2-(2-(prop-2-yn-1-yloxy)ethoxy)ethoxy)ethan-1-amine (8.1 mg, 43 µmol). After stirring for 1 hour at ambient temperature, the reaction mixture was concentrated under reduced pressure. Purification by flash column chromatography, eluting with 0-10% methanol/dichloromethane, afforded the title compound as a yellow solid (10 mg, 44%), which was used directly in the subsequent stage. LC-MS (m/z, ES+): 679.1 $[M+H]^+$.

(2*R*)-2-((6*S*)-4-(4-acrylamidophenyl)-2,3,9-trimethyl-6*H*-thieno[3,2-*f*][1,2,4]triazolo[4,3-*a*][1,4]diazepin-6-yl)-*N*-(2-(2-(2-(prop-2-yn-1-yloxy)ethoxy)ethoxy)ethyl)butanamide (MR155)

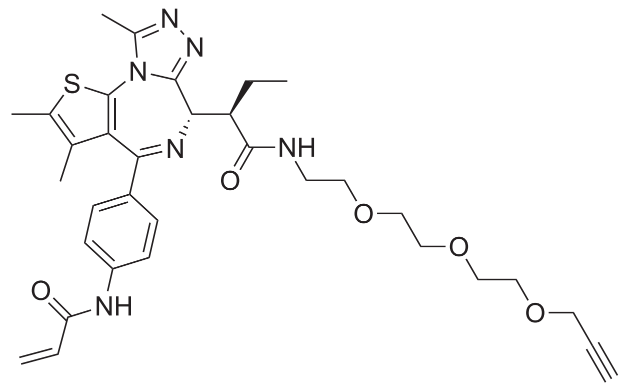

To a solution of MR152 (10 mg, 17 µmol) was added a solution of 20% TFA in dichloromethane (1 mL) and stirred for 30 min. Once Boc group removal was confirmed by LC-MS, the reaction mixture was concentrated under reduced pressure. A mixture of acrylic acid (2.4 mg, 33 µmol), HATU (12.7 mg, 33 µmol) and *N*,*N*-diisopropylethylamine (15 µL, 83 µmol) in dichloromethane (0.5 mL) was stirred for 5 minutes before adding a solution of the crude aniline (0.5 equiv., 8 mg, 17 µmol) in dichloromethane (0.5 mL). After stirring for 1 hour, the reaction mixture was concentrated under reduced pressure. Purification by preparative reversed phase HPLC, using a gradient of 5/95 to 95/5 MeCN/H_2O (0.1% formic acid), afforded the title compound as a white solid (3 mg, 15% over two steps).

^{1}H NMR (500 MHz, $CDCl_3$) δ 7.73 (s, 1H), 7.55 (d, *J* = 8.4 Hz, 2H), 7.33 (d, *J* = 8.8 Hz, 2H), 6.72 (s, 1H), 6.36 (d, *J* = 16.8 Hz, 1H), 6.20 (dd, *J* = 16.8, 10.3 Hz, 1H), 5.69 (d, *J* = 10.1 Hz, 1H), 4.19 – 4.08 (m, 3H), 3.74 – 3.47 (m, 12H), 3.48 – 3.35 (m, 1H), 2.58 (s, 3H), 2.40 – 2.35 (m, 1H), 2.32 (s, 3H), 2.03 – 1.94 (m, 1H), 1.70 – 1.61 (m, 1H), 1.60 (s, 3H), 0.96 (t, *J* = 7.3 Hz, 3H); LC-MS (m/z, ES+): 633.4 $[M+H]^+$. HRMS (m/z) $[M+H]^+$ calculated for $C_{33}H_{40}N_6O_5S$, 633.28536; found 633.28741.

5(6)-((2,2-dimethyl-4-oxo-3,8,11,14-tetraoxa-5-azahexadecan-16-yl)carbamoyl)-2-(6 (dimethylamino)-3-(dimethyliminio)-3*H*-xanthen-9-yl)benzoate (MR185)

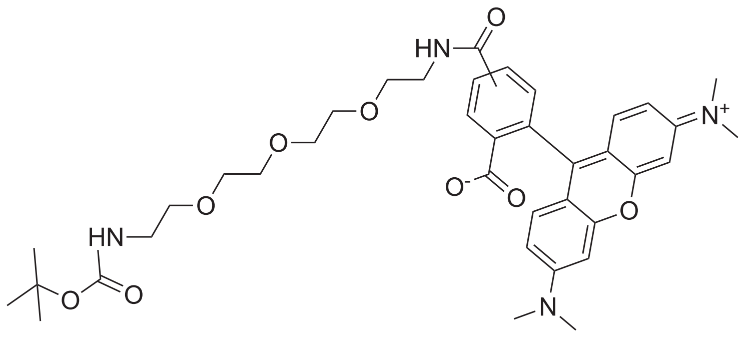

To a solution of 5(6)-TAMRA (23 mg, 50 µmol) in dichloromethane (1 mL), *N,N*-diisopropylethylamine (16 µL, 92 µmol) was added followed by HATU (24.4 mg, 25 µmol) and the reaction was stirred for 5 min before adding a mixture of Boc-amino-PEG$_3$-amine (23.4 mg, 46 µmol) and *N,N*-diisopropylethylamine (1.5 eq, 0.37 mL, 210 µmol). The mixture was stirred at room temperature for 1 h. The solvent was evaporated under reduced pressure and the product was purified by flash column chromatography, eluting with 0-5% methanol/ title compound as a pink solid (20 mg, 32 µmol, 61.9 % yield). LC-MS (m/z, ES+): 705.4 $[M+H]^+$.

5(6)-(((14*R*)-14-((6*S*)-4-(4-acrylamidophenyl)-2,3,9-trimethyl-6*H*-thieno[3,2-*f*][1,2,4]triazolo[4,3-*a*][1,4]diazepin-6-yl)-13-oxo-3,6,9-trioxa-12-azahexadecyl)carbamoyl)-2-(6-(dimethylamino)-3-(dimethyliminio)-3*H*-xanthen-9-yl)benzoate (MR202)

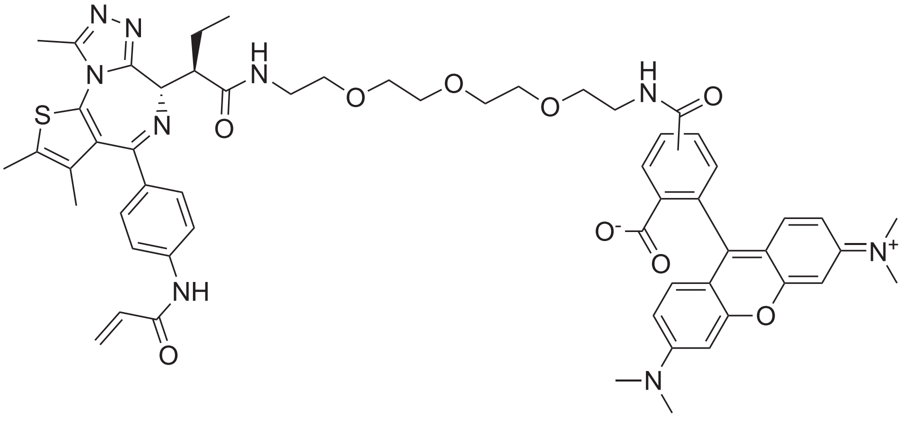

MR185 (11 mg, 18 µmol) was dissolved in 20% TFA in DCM and the reaction stirred for one hour. After one hour, the solvent was evaporated and to the crude amine, a solution of MR137 (10 mg, 180 µmol) and *N,N*-diisopropylethylamine (205 µL, 120 µmol) in DMF (1 mL) at ambient temperature was added, followed by HATU (12 mg, 180 µmol) and the resulting mixture. After stirring for 16 hours, the reaction mixture was concentrated under reduced pressure. The resulting Boc-aniline precursor (10 mg (uncorr.), 0.18 µmol (corr.)) was dissolved in a solution of 20% TFA in dichloromethane (2 mL) and the reaction mixture was stirred at room temperature for 1 hour. The solvent was evaporated, and the resulting crude resuspended in dichloromethane. The stirred solution was treated with triethylamine (0.90 mL) followed by the portion-wise addition of acryloyl chloride diluted in dichloromethane (440 µL of acryloyl chloride dissolved in 1 mL of dichloromethane, 0.6 mL added), and the reaction mixture was stirred at ambient temperature for 4 hours before being concentrated under reduced pressure. Purification by preparative reversed phase HPLC, using a gradient of 5/95 to 95/5 MeCN/H_2O (0.1% formic acid), afforded the title compound as a white solid (10 mg, 53% over two steps). The 6-isomer was the predominant species.

6-isomer ^{1}H NMR (500 MHz, DMSO) δ 10.25 (s, 1H), 8.72 – 8.66 (m, 1H), 8.30 – 8.25 (m, 1H), 8.08 (d, *J* = 7.9 Hz, 1H), 7.98 (d, *J* = 8.0 Hz, 1H), 7.61 (d, *J* = 8.3 Hz, 2H), 7.58 (s, 1H), 7.26 (d, *J* = 8.3 Hz, 2H), 6.48 – 6.39 (m, 7H), 6.35 (dd, *J* = 17.1, 9.9 Hz, 1H), 6.18 (d, *J* = 17.0 Hz, 1H), 5.69 (d, *J* = 10.7 Hz, 1H), 3.99 (d, *J* = 10.7 Hz, 1H), 3.49 – 3.36 (m, 16H), 2.87 (s, 12H), 2.51 (s, 3H), 2.36 – 2.32 (m, 3H), 1.80 – 1.77 (m, 1H), 1.56 (s, 3H), 1.39 – 1.31 (m, 1H), 0.81 (t, *J* = 7.3 Hz, 3H). 5-isomer ^{1}H NMR (400 MHz, DMSO) δ 10.33 (s, 1H), 8.89 (t, *J* = 5.5 Hz, 1H), 8.44 (d, *J* = 1.6 Hz, 1H), 8.38 (t, *J* = 5.7 Hz, 1H), 8.22 (dd, *J* = 8.1, 1.6 Hz, 1H), 7.68 (d, *J* = 9.0 Hz, 2H), 7.31 (dd, *J* = 15.0, 8.2 Hz, 3H), 6.54 – 6.43 (m, 7H), 6.41 (d, *J* = 10.1 Hz, 1H), 6.26 (dd, *J* = 17.0, 2.0 Hz, 1H), 5.76 (dd, *J* = 10.1, 2.0 Hz, 1H), 4.05 (d, *J* = 10.8 Hz, 1H), 3.61 – 3.43 (m, 16H), 2.94 (s, 12H), 2.57 (s, 3H), 2.40 (s, 3H), 1.93 – 1.79 (m, 1H), 1.62 (s, 3H), 1.48 – 1.35 (m, 1H), 0.89 (t, *J* = 7.4 Hz, 3H). LC-MS (m/z, ES+): 526.1 $[M+2H]^{+2}$, HRMS (m/z) $[M+H]^{+}$ calculated for $C_{57}H_{63}N_{9}O_{9}S$, 1050.45422; found 1050.45421.

4-((2-(2-(2-(2-Aminoethoxy)ethoxy)ethoxy)ethyl)carbamoyl)-2-(3-(3-fluoroazetidin-1-ium-1-ylidene)-7-(3-fluoroazetidin-1-yl)-5,5-dimethyl-3,5-dihydrodibenzo[b,e]silin-10-yl)benzoate

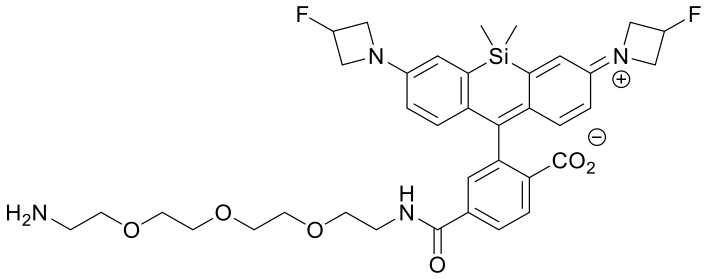

To a stirred solution of Amino-PEG$_3$-amine (450 mg, 290 μmol) in dichloromethane (3 mL) at ambient temperature was added a solution of Janelia Fluor® 635, NHS ester (200 mg (uncorr.), 184 mg (corr.), 290 μmol (corr.)) in dichloromethane (5 mL), and the reaction mixture was stirred for 2 hours before being concentrated under reduced pressure. Purification by flash column chromatography, eluting with 0-20% methanol in dichloromethane, afforded the title compound as a pale-yellow solid (175 mg (uncorr.), 166 mg (corr.), 81% (corr.)).

^{1}H NMR (400 MHz, DMSO-d6): **δ** 8.85 (t, *J* = 5.5 Hz, 1H), 8.08 (dd, *J* = 8.0, 1.3 Hz, 1H), 8.06 – 8.01 (m, 1H), 7.68 – 7.64 (m, 1H), 6.82 (d, *J* = 2.6 Hz, 2H), 6.66 (d, *J* = 8.7 Hz, 2H), 6.42 (dd, *J* = 8.8, 2.7 Hz, 2H), 5.47 (dtt, *J* = 57.3, 5.8, 3.0 Hz, 2H), 4.25 – 4.10 (m, 4H), 3.98 – 3.84 (m, 4H), 3.53 – 3.40 (m, 10H), 3.40 – 3.34 (m, 2H), 3.31 (t, *J* = 5.7 Hz, 2H), 2.61 (t, *J* = 5.7 Hz, 2H), 2.37 (br. s, *J* = 17.3 Hz, 2H), 0.63 (s, 3H), 0.52 (s, 3H); LCMS purity = 98.7%; LC-MS (m/z, ES+): 707.40 $[M+H]^{+}$.

4-(((*R*)-14-((*S*)-4-(4-Acrylamidophenyl)-2,3,9-trimethyl-6H-thieno[3,2-f][1,2,4]triazolo[4,3-a][1,4]diazepin-6-yl)-13-oxo-3,6,9-trioxa-12-azahexadecyl)carbamoyl)-2-(3-(3-fluoroazetidin-1-ium-1-ylidene)-7-(3-fluoroazetidin-1-yl)-5,5-dimethyl-3,5-dihydrodibenzo[b,e]silin-10-yl)benzoate (C10852S)

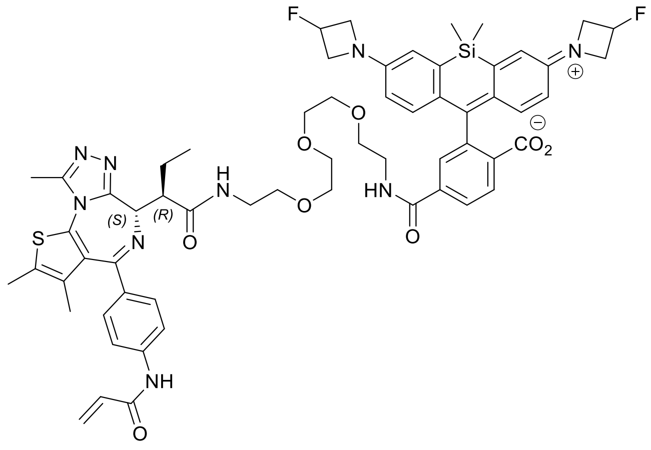

To a stirred solution of (2*R*)-2-[(9*S*)-7-[4-(tert-butoxycarbonylamino)phenyl]-4,5,13-trimethyl-3-thia-1,8,11,12-tetrazatricyclo[8.3.0.02,6]trideca-2(6),4,7,10,12-pentaen-9-yl]butanoic acid (MR137) (50 mg (uncorr.), 43 mg (corr.), 83.40 µmol (corr.)) in dichloromethane (3.0 mL) at ambient temperature was sequentially added bis(2,5-dioxopyrrolidin-1-yl) carbonate (43 mg, 167.86 µmol), *N,N*-dimethylpyridin-4-amine (2 mg, 16.37 µmol) and triethylamine (60 µL, 430.48 µmol) and the reaction mixture was stirred at ambient temperature for 45 minutes. After this time, a solution of 4-((2-(2-(2-(2-aminoethoxy)ethoxy)ethoxy)ethyl)carbamoyl)-2-(3-(3-fluoroazetidin-1-ium-1-ylidene)-7-(3-fluoroazetidin-1-yl)-5,5-dimethyl-3,5-dihydrodibenzo[b,e]silin-10-yl)benzoate (75 mg (uncorr.), 72 mg (corr.), 100.80 µmol (corr.)) in dichloromethane (2.0 mL) was added and the reaction mixture was stirred at ambient temperature for 20 hours before being concentrated under reduced pressure. Purification by flash column chromatography, eluting with 0-4% methanol in dichloromethane, afforded the Boc-protected precursor as a white solid (72 mg (uncorr.), 65 mg (corr.), 65% (corr.)). LCMS purity = 96.5%; LC-MS (m/z, ES+): 1198.30 $[M+H]^+$.

To a stirred solution of the Boc-precursor (72 mg (uncorr.), 54.07 µmol (corr.)) in dichloromethane (8 mL) was added TFA (0.40 mL) and the reaction mixture was stirred at 39 °C for 18 hours. After cooling to ambient temperature, the solution was treated with triethylamine (0.90 mL) followed by the portion-wise addition of acryloyl chloride diluted in dichloromethane (440 µL of acryloyl chloride dissolved in 1 mL of dichloromethane, 0.60 mL added), and the reaction mixture was stirred at ambient temperature for 4 hours before being concentrated under reduced pressure and reconcentrated from heptane. Purification by reversed phase chromatography, eluting with 10-70% acetonitrile (0.1% formic acid) in water (0.1% formic acid), was followed by conversion of the resulting formate salt to the free base by extractive isolation (dichloromethane and $NaHCO_3$ (aq.)) before lyophilisation from acetonitrile/water (1:1) afforded the title compound as an off-white solid (37 mg, 56%).

^{1}H NMR (400 MHz, DMSO-d6): δ 10.31 (s, 1H), 8.80 (t, *J* = 5.5 Hz, 1H), 8.35 (t, *J* = 5.6 Hz, 1H), 8.08 (dd, *J* = 8.1, 1.2 Hz, 1H), 8.02 (d, *J* = 8.1 Hz, 1H), 7.68 (d, *J* = 8.9 Hz, 2H), 7.66 – 7.66 (m, 1H), 7.33 (d, *J* = 8.5 Hz, 2H), 6.81 (d, *J* = 2.6 Hz, 2H), 6.66 (d, *J* = 8.7 Hz, 2H), 6.46 – 6.38 (m, 3H), 6.25 (dd, *J* = 17.0, 2.0 Hz, 1H), 5.75 (dd, *J* = 10.1, 2.0 Hz, 1H), 5.46 (dtt, *J* = 57.7, 5.7, 3.0 Hz, 2H), 4.23 – 4.10 (m, 4H), 4.06 (d, *J* = 10.8 Hz, 1H), 3.97 – 3.83 (m, 4H), 3.56 – 3.44 (m, 13H), 3.40 – 3.33 (m, 4H), 2.58 (s, 3H), 2.40 (s, 3H), 1.92 – 1.80 (m, 1H), 1.63 (s, 3H), 1.48 – 1.35 (m, 1H), 0.88 (t, *J* = 7.4 Hz, 3H), 0.63 (s, 3H), 0.51 (s, 3H); ^{13}C NMR (100 MHz, DMSO-d6): δ 172.88, 169.20, 164.88, 163.34, 162.36, 154.69, 154.60, 150.02, 150.01, 149.55, 140.99, 139.96, 135.75, 132.82, 132.18, 131.63, 130.73, 130.23, 129.95, 129.12, 128.28, 127.47, 127.36, 127.09, 125.54, 122.74, 118.62, 116.22, 113.46, 90.84, 83.48 (d, J_{CF} = 200.1 Hz), 69.72, 69.61, 69.51, 69.41, 68.64, 59.24 (d, J_{CF} = 23.4 Hz), 59.06, 49.34, 39.44, 38.49, 22.46, 14.06, 12.70, 11.59, 11.25, -0.10, -1.31; ^{19}F NMR (376 MHz, DMSO-

d6): δ -179.1; LCMS purity = 96.6%; LC-MS (m/z, ES+): 1152.30 $[M+H]^+$; HRMS (m/z) $[M+H]^+$ calculated for $C_{61}H_{67}F_2N_9O_8SSi$, 1152.4649; found 1152.4647.

(*R*)-2-((*S*)-4-(4-Acrylamidophenyl)-2,3,9-trimethyl-6H-thieno[3,2-f][1,2,4]triazolo[4,3-a][1,4]diazepin-6-yl)butanoic acid

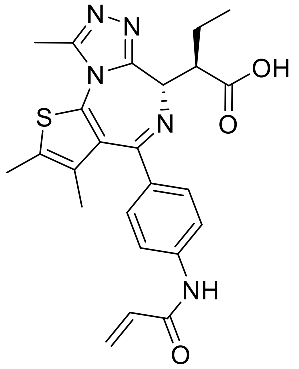

A solution of (*R*)-2-((*S*)-4-(4-((tert-butoxycarbonyl)amino)phenyl)-2,3,9-trimethyl-6H-thieno[3,2-f][1,2,4]triazolo[4,3-a][1,4]diazepin-6-yl)butanoic acid (393 mg (uncorr.), 365 mg (corr.), 0.717 mmol (corr.)) in methanol (3 mL) was treated with hydrogen chloride (21.0 mL, 84.0 mmol, 4 M solution in 1,4-dioxane), and the resulting solution was stirred at ambient temperature for 3 hours before being concentrated under reduced pressure. To the orange residue was added sodium acetate (300 mg, 3.66 mmol) and acetonitrile (15 mL), and the suspension was sonicated until a yellow solution with suspension was obtained. To the suspension was added acryloyl chloride (65 µL diluted with 435 µL acetonitrile, 0.800 mmol). The mixture was stirred at ambient temperature for 30 minutes before being concentrated under reduced pressure. The residue was treated with water and the pH was adjusted to pH 1 using 2M hydrochloric acid. The mixture was extracted with ethyl acetate (3x 100 mL) and the combined organic extracts were washed with brine (50 mL), dried over anhydrous magnesium sulfate and concentrated under reduced pressure. Purification by flash column chromatography, eluting with 2-15% MeOH in DCM, followed by stripping the desired fractions from TBME (10 mL), afforded the title compound as an off-white solid (151 mg (uncorr.), 136 mg (corr.), 41% (corr.)). ^{1}H NMR (400 MHz, DMSO-d6) δ 12.36 (br. s, 1H), 10.35 (s, 1H), 7.70 (d, *J* = 8.9 Hz, 2H), 7.36 (d, *J* = 8.7 Hz, 2H), 6.44 (dd, *J* = 17.0, 10.1 Hz, 1H), 6.27 (dd, *J* = 17.0, 2.0 Hz, 1H), 5.78 (dd, *J* = 10.1, 2.0 Hz, 1H), 4.09 (d, *J* = 10.7 Hz, 1H), 3.54 (td, *J* = 10.6, 3.5 Hz, 1H), 2.59 (s, 3H), 2.42 (s, 3H), 2.02 – 1.91 (m, 1H), 1.65 (s, 3H), 1.61 – 1.52 (m, 1H), 0.96 (t, *J* = 7.4 Hz, 3H); LC-MS (m/z, ES+): 464.1 $[M+H]^+$.

2,5-Dioxopyrrolidin-1-yl (*R*)-2-((*S*)-4-(4-acrylamidophenyl)-2,3,9-trimethyl-6H-thieno[3,2-f][1,2,4]triazolo[4,3-a][1,4]diazepin-6-yl)butanoate

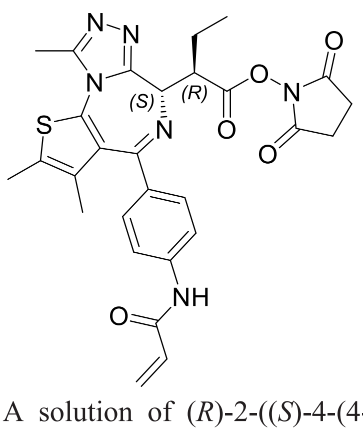

A solution of (*R*)-2-((*S*)-4-(4-acrylamidophenyl)-2,3,9-trimethyl-6H-thieno[3,2-f][1,2,4]triazolo[4,3-a][1,4]diazepin-6-yl)butanoic acid (150 mg (uncorr.), 135 mg (corr.), 0.291 mmol (corr.)) in DCM (10 mL) was treated with DMAP (7 mg, 57.3 µmol), triethylamine (0.20 mL, 1.43 mmol) and *N*,*N'*-disuccinimidyl carbonate (165 mg, 0.644 mmol). The reaction mixture was stirred at ambient temperature for 1 h before being diluted with DCM (80 mL). The organics were sequentially washed

with 1M hydrochloric acid (120 mL), saturated sodium hydrogen carbonate solution (2x 80 mL), dried over anhydrous magnesium sulfate and concentrated under reduced pressure. Purification by flash column chromatography, eluting with 10-50% acetone in DCM, and subsequent reconcentration from DCM/TBME/heptane (2:2:1; 10 mL) afforded the title compound as a white solid (145 mg (uncorr.), 136 mg (corr.), 84% (corr.)).

^{1}H NMR (400 MHz, DMSO-d6) δ 10.31 (s, 1H), 7.71 – 7.65 (m, *J* = 9.0 Hz, 2H), 7.54 – 7.47 (m, *J* = 8.0 Hz, 2H), 6.43 (dd, *J* = 17.0, 10.1 Hz, 1H), 6.27 (dd, *J* = 17.0, 2.0 Hz, 1H), 5.78 (dd, *J* = 10.1, 2.0 Hz, 1H), 4.31 (d, *J* = 11.0 Hz, 1H), 3.93 (td, *J* = 10.9, 3.5 Hz, 1H), 3.00 – 2.76 (m, 4H), 2.61 (s, 3H), 2.41 (s, 3H), 2.22 – 2.11 (m, 1H), 1.75 – 1.60 (m, 4H), 1.15 (t, *J* = 7.5 Hz, 3H); LC-MS (m/z, ES+): 561.1 $[M+H]^+$.

4-((2-Aminoethyl)carbamoyl)-2-(3-(3-fluoroazetidin-1-ium-1-ylidene)-7-(3-fluoroazetidin-1-yl)-5,5-dimethyl-3,5-dihydrodibenzo[b,e]silin-10-yl)benzoate

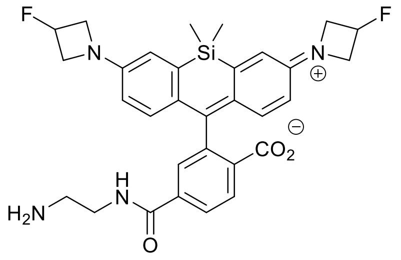

To a solution of ethane-1,2-diamine (0.50 mL, 7.48 mmol) in DCM (20 mL) was added a solution of 4-(2,5-dioxopyrrolidin-1-yl)oxycarbonyl-2-[3-(3-fluoroazetidin-1-ium-1-ylidene)-7-(3-fluoroazetidin-1-yl) 5,5-dimethyl-benzo[b][1]benzosilin-10-yl]benzoate (420 mg (uncorr.), 336mg (corr.), 0.534 mmol (corr.)) in DCM (10 mL). The RM was stirred at ambient temperature for 30 minutes before being purified directly by flash column chromatohgraphy, eluting with 2-20% MeOH/DCM, to afford the title compound as a light yellow solid (304 mg (uncorr.), 280 mg (corr.), 91% (corr.)).

^{1}H NMR (400 MHz, DMSO-d6) δ 8.70 – 8.60 (m, 1H), 8.08 (dd, *J* = 8.0, 1.3 Hz, 1H), 8.02 (dd, *J* = 8.0, 0.5 Hz, 1H), 7.70 – 7.65 (m, 1H), 6.82 (d, *J* = 2.6 Hz, 2H), 6.67 (d, *J* = 8.7 Hz, 2H), 6.42 (dd, *J* = 8.7, 2.7 Hz, 2H), 5.47 (dtt, *J* = 57.2, 5.7, 3.0 Hz, 2H), 4.24 – 4.10 (m, 4H), 3.98 – 3.83 (m, 4H), 3.25 – 3.17 (m, 2H), 2.63 (t, *J* = 6.5 Hz, 2H), 1.52 (br. s, 2H), 0.63 (s, 3H), 0.52 (s, 3H); LC-MS (m/z, ES+): 575.1 $[M+H]^+$.

4-((2-((*R*)-2-((*S*)-4-(4-Acrylamidophenyl)-2,3,9-trimethyl-6H-thieno[3,2-f][1,2,4]triazolo[4,3-a][1,4]diazepin-6-yl)butanamido)ethyl)carbamoyl)-2-(3-(3-fluoroazetidin-1-ium-1-ylidene)-7-(3-fluoroazetidin-1-yl)-5,5-dimethyl-3,5-dihydrodibenzo[b,e]silin-10-yl)benzoate (C10852L)

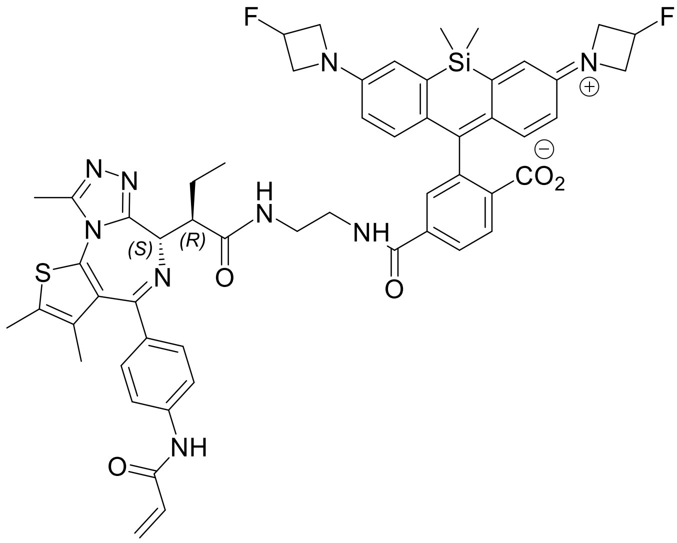

To a solution of (2,5-dioxopyrrolidin-1-yl) (2*R*)-2-[(9*S*)-4,5,13-trimethyl-7-[4-(prop-2-enoylamino)phenyl]-3-thia-1,8,11,12-tetrazatricyclo[8.3.0.02,6]trideca-2(6),4,7,10,12-pentaen-9-yl]butanoate (50 mg (uncorr.), 47 mg (corr.), 83.84 umol (corr.)) in DCM (0.5 mL) was added triethylamine (30 µL, 215 umol) and 4-(2-aminoethylcarbamoyl)-2-[3-(3-fluoroazetidin-1-ium-1-ylidene)-7-(3-fluoroazetidin-1-yl)-5,5-dimethyl-benzo[b][1]benzosilin-10-yl]benzoate (63 mg (uncorr.), 58.6 mg (corr.), 102 umol (corr.)) followed by DCM (1 mL). The reaction mixture was stirred at ambient temperature for 4 hours before being concentrated under a stream of nitrogen. Purification by reversed phase chromatography, eluting with 10-60% acetonitrile (0.1% formic acid) in water (0.1% formic acid), was followed by conversion of the resulting formate salt to the free base by treatment with saturated aqueous sodium hydrogen carbonate solution and extractive isolation with DCM. Lyophilisation from acetonitrile/water (1:1) afforded the title compound as a light-green solid (71 mg (uncorr.), 69.6 mg (corr.), 81% (corr).

^{1}H NMR (400 MHz, DMSO-d6) δ 10.30 (s, 1H), 8.86 (s, 1H), 8.54 – 8.45 (m, 1H), 8.09 (dd, J = 8.1, 1.2 Hz, 1H), 8.03 (d, J = 8.1 Hz, 1H), 7.73 – 7.70 (m, 1H), 7.66 (d, J = 8.9 Hz, 2H), 7.29 (d, J = 8.6 Hz, 2H), 6.80 (dd, J = 5.9, 2.7 Hz, 2H), 6.64 (d, J = 8.7 Hz, 1H), 6.61 (d, J = 8.7 Hz, 1H), 6.41 (dd, J = 17.0, 10.1 Hz, 1H), 6.35 (dd, J = 8.8, 2.6 Hz, 2H), 6.24 (dd, J = 17.0, 2.0 Hz, 1H), 5.76 (dd, J = 10.1, 2.0 Hz, 1H), 5.57 – 5.35 (m, 2H), 4.21 – 4.08 (m, 4H), 4.04 (d, J = 10.8 Hz, 1H), 3.96 – 3.81 (m, 4H), 3.53 – 3.36 (m, 5H), 2.58 (s, 3H), 2.39 (d, J = 0.5 Hz, 3H), 1.87 – 1.76 (m, 1H), 1.53 (d, J = 0.5 Hz, 3H), 1.42 – 1.30 (m, 1H), 0.81 (t, J = 7.4 Hz, 3H), 0.62 (s, 3H), 0.51 (s, 3H); ^{13}C NMR (101 MHz, DMSO-d6) δ 173.03, 168.65, 164.43, 162.86, 162.03, 154.08, 153.94, 149.56, 149.13, 140.55, 139.51, 135.49, 135.48, 132.34, 131.79, 131.78, 131.21, 130.27, 129.74, 129.49, 128.61, 127.73, 126.99, 126.98, 126.85, 125.15, 122.50, 118.20, 115.83, 115.80, 112.86, 112.83, 90.53, 83.02 (d, *J* = 200.6 Hz), 58.78 (d, *J* = 23.3 Hz), 58.57, 49.10, 39.75, 37.39, 21.89, 13.55, 12.24, 11.15, 10.80, -0.50, -1.92; ^{19}F NMR (377 MHz, DMSO-d6) δ -179.06, -179.08; LC-MS (m/z, ES+): 1020.4 $[M+H]^+$; HRMS (m/z) $[M+Na]^+$ calculated for $C_{55}H_{55}F_2N_9O_5SSi$ 1042.3682, found 1042.3675.

FULL GELS and Western Blots

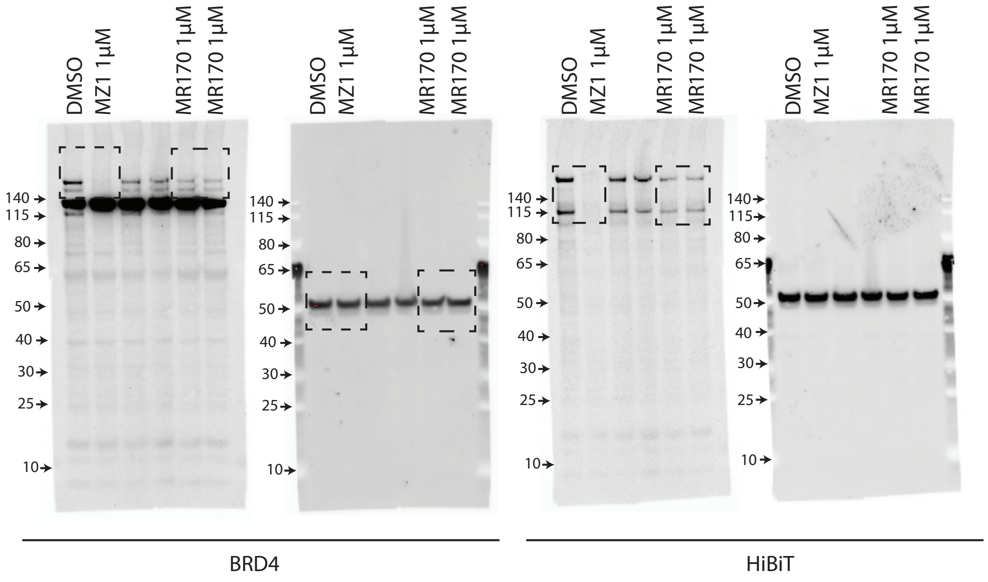

Supplementary Figure 25. Full gel western blot membrane from experiment shown in Supplementary Figure 10. A) Tubulin was imaged with α-tubulin antibody conjugated to Rhodamine (imaged in the ChemiDoc in the rhodamine channel). Brd4 was labelled with the Brd4 antibody conjugated to a IR800 dye and imaged in the ChemiDoc in the IR800 channel. MR170 lysate was loaded twice on the same gel as two technical replicates from one independent experiment.

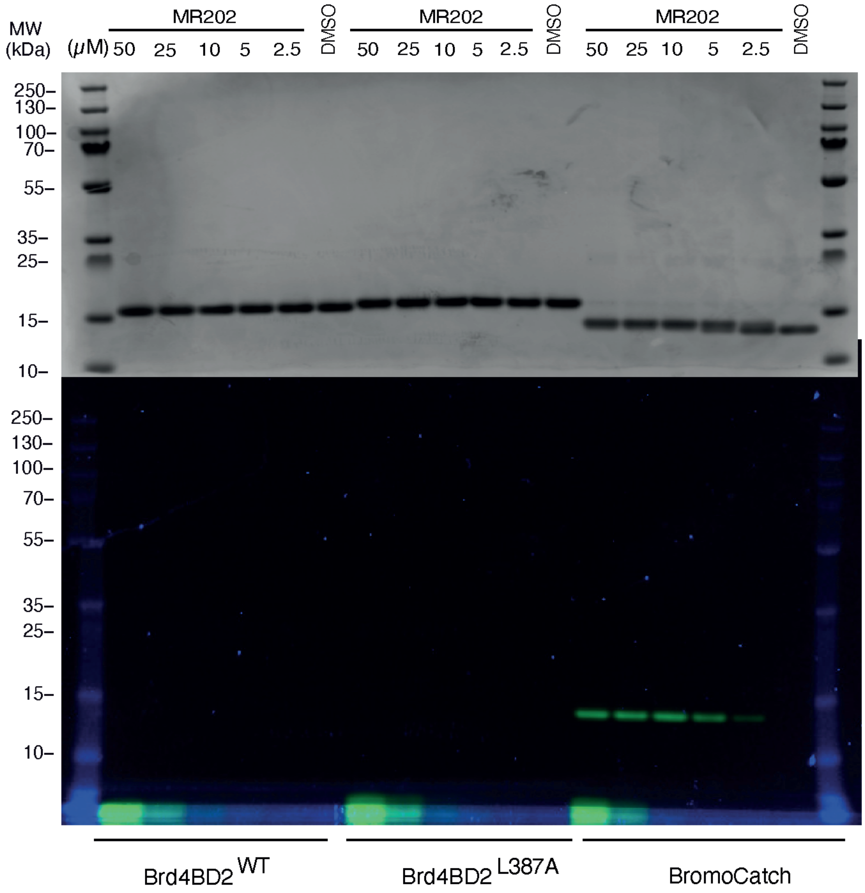

Supplementary Figure 26. Full length SDS gel of MR202 titration corresponding to Figure 6. Gel was imaged on a ChemiDoc in the Alexa546 channel. Data is representative of one independent experiment; the experiment was repeated twice with similar results.

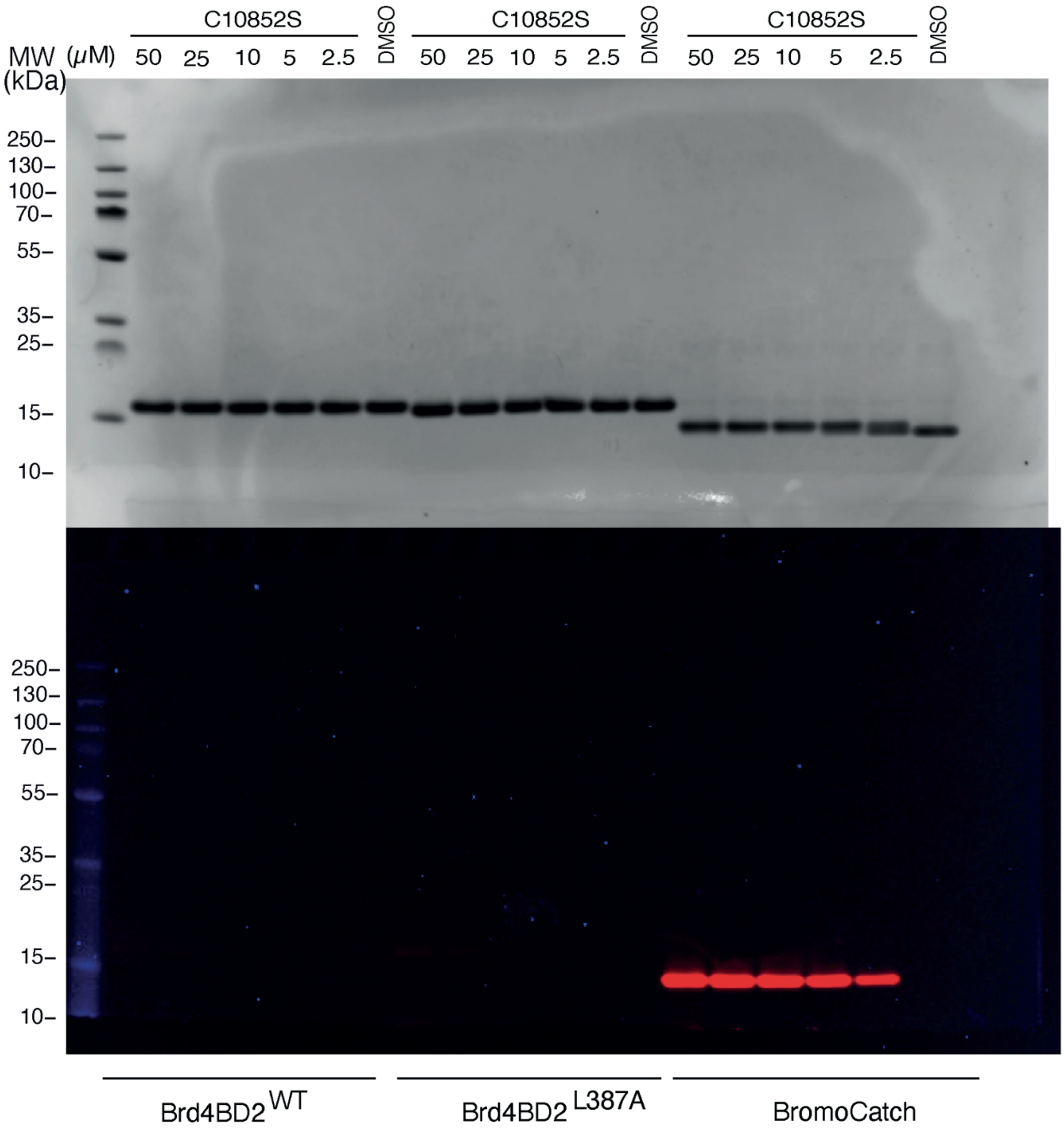

Supplementary Figure 27. Full length SDS gel of C18052S titration corresponding to Figure 7. Gel was imaged on a ChemiDoc in the Cy5 channel. Data is representative of one independent experiment; the experiment was repeated twice with similar results.

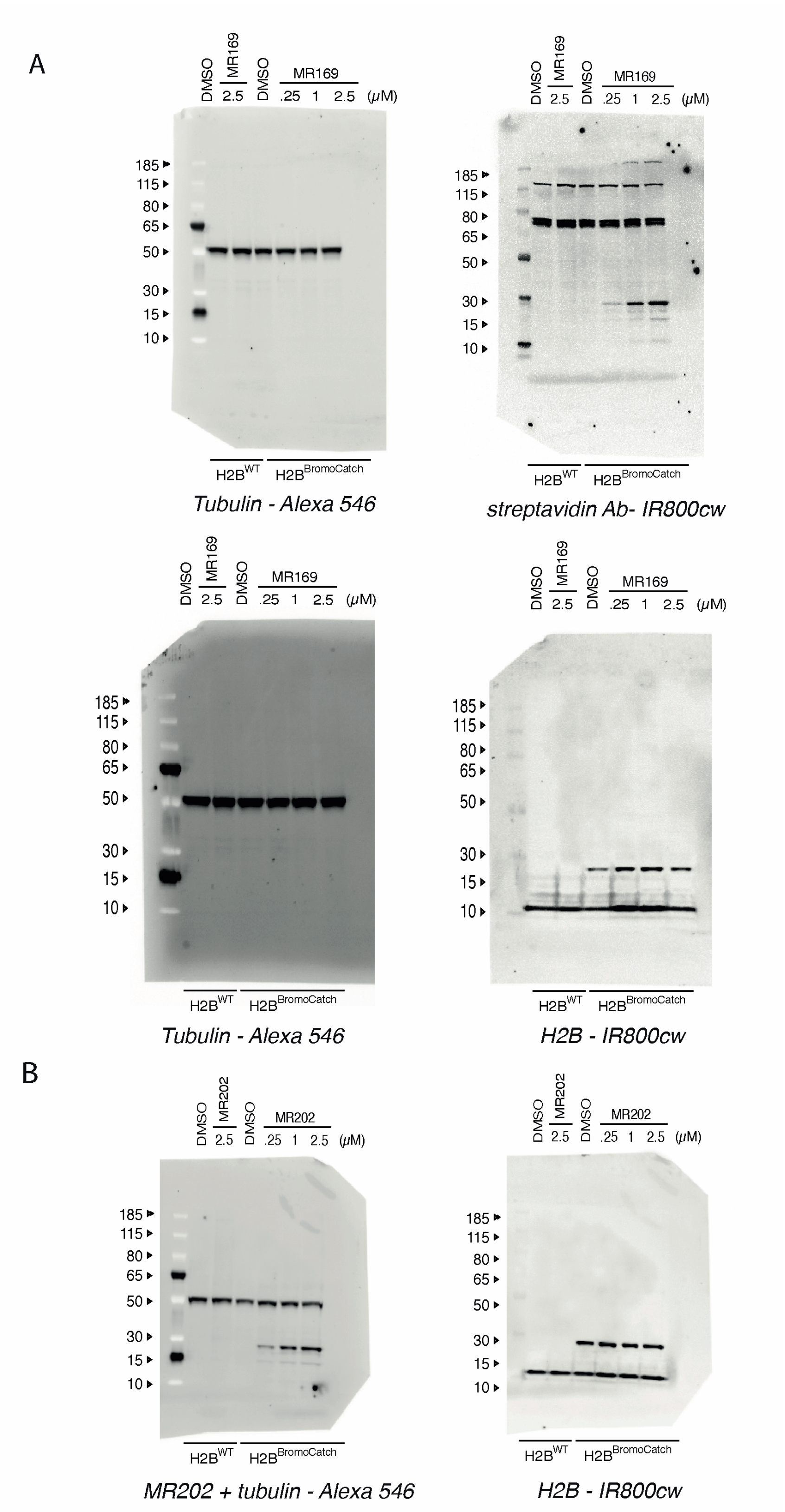

Supplementary Figure 28. Full gel western blot membrane from experiments shown in Figure 7 and 8. A) Tubulin was imaged with α-tubulin antibody conjugated to Rhodamine and imaged on ChemiDoc in the Alexa546 channel. Biotin was labelled with streptavidin antibody conjugated to a

IR800 dye and imaged on ChemiDoc in the IR800 channel. The biotin membrane also reveals endogenous biotinylated proteins that show both in WT and H2B-BromoCatch tranfected cells independently of probe treatment.

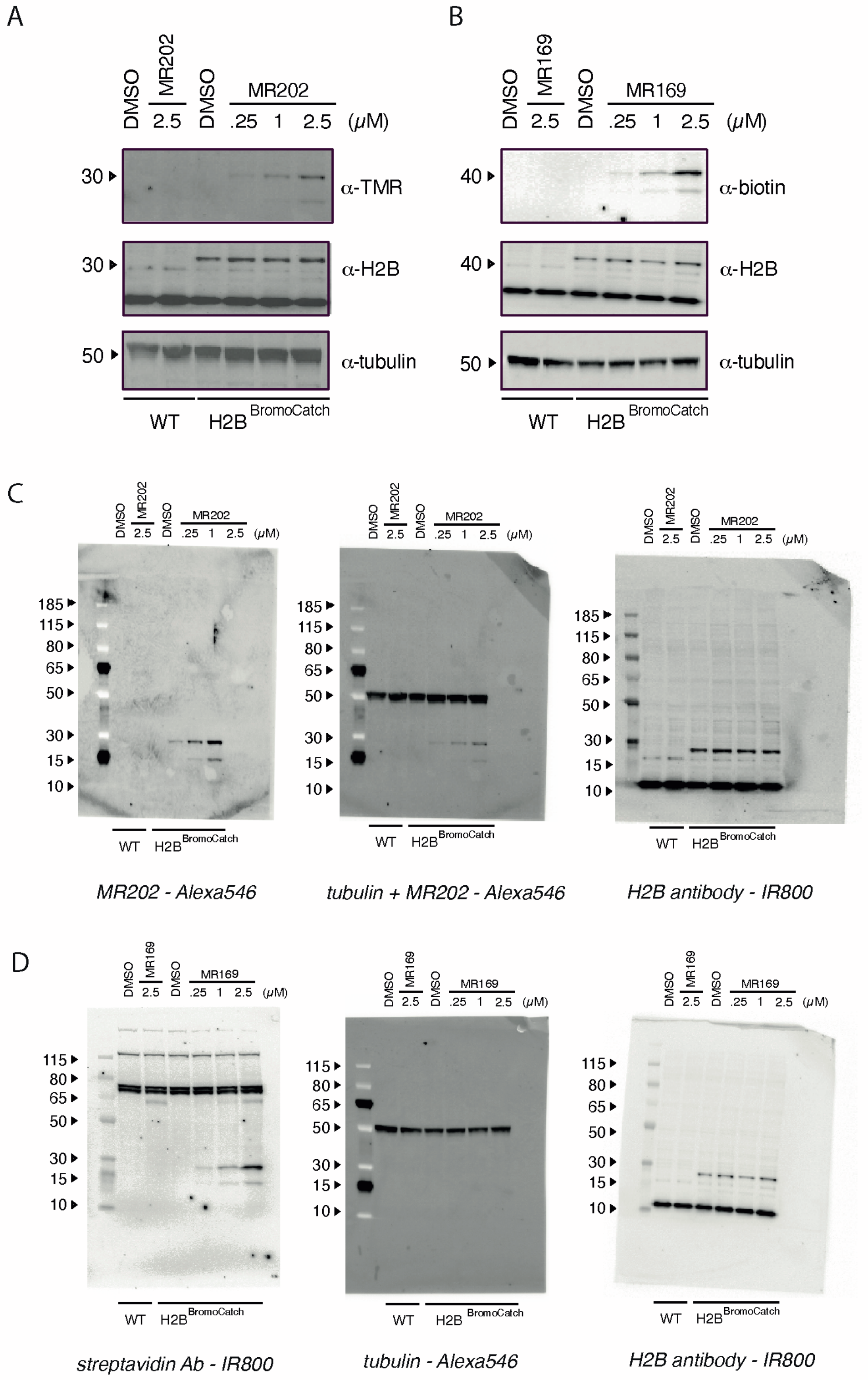

Supplementary Figure 29. Full gel western blot membranes from N=2 experiments. A) N=2 biological repeat of MR202 and B) MR169 validation. C) MR202 experiment full length membrane. Tubulin was imaged with α-tubulin antibody conjugated to Rhodamine, imaged on ChemiDoc in the Alexa546 channel. D) Biotin was labelled with streptavidin antibody conjugated to a IR800 dye and

imaged in the ChemiDoc in the IR800 channel. The biotin membrane also reveals endogenous biotinylated proteins that show both in WT and H2B-BromoCatch tranfected cells independently of probe treatment.

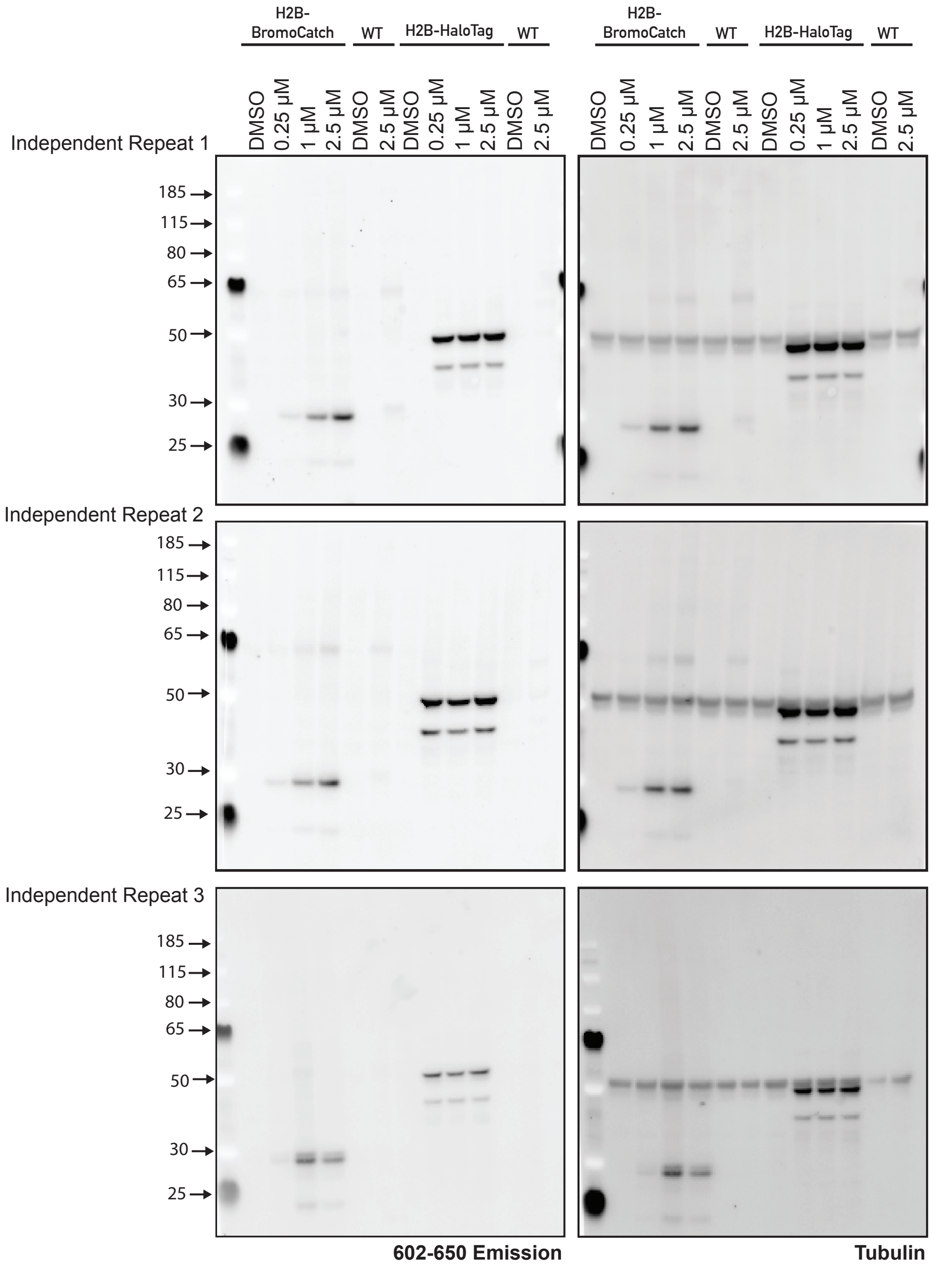

Supplementary Figure 30. Full gel western blot membranes from N=3 experiments. A) N=3 biological repeats of MR202 and HaloTag TMR immunoblotting competition shown in Supplementary Figure 15. Tubulin was imaged with α-tubulin antibody conjugated to Rhodamine, imaged on ChemiDoc in the Alexa546 channel.

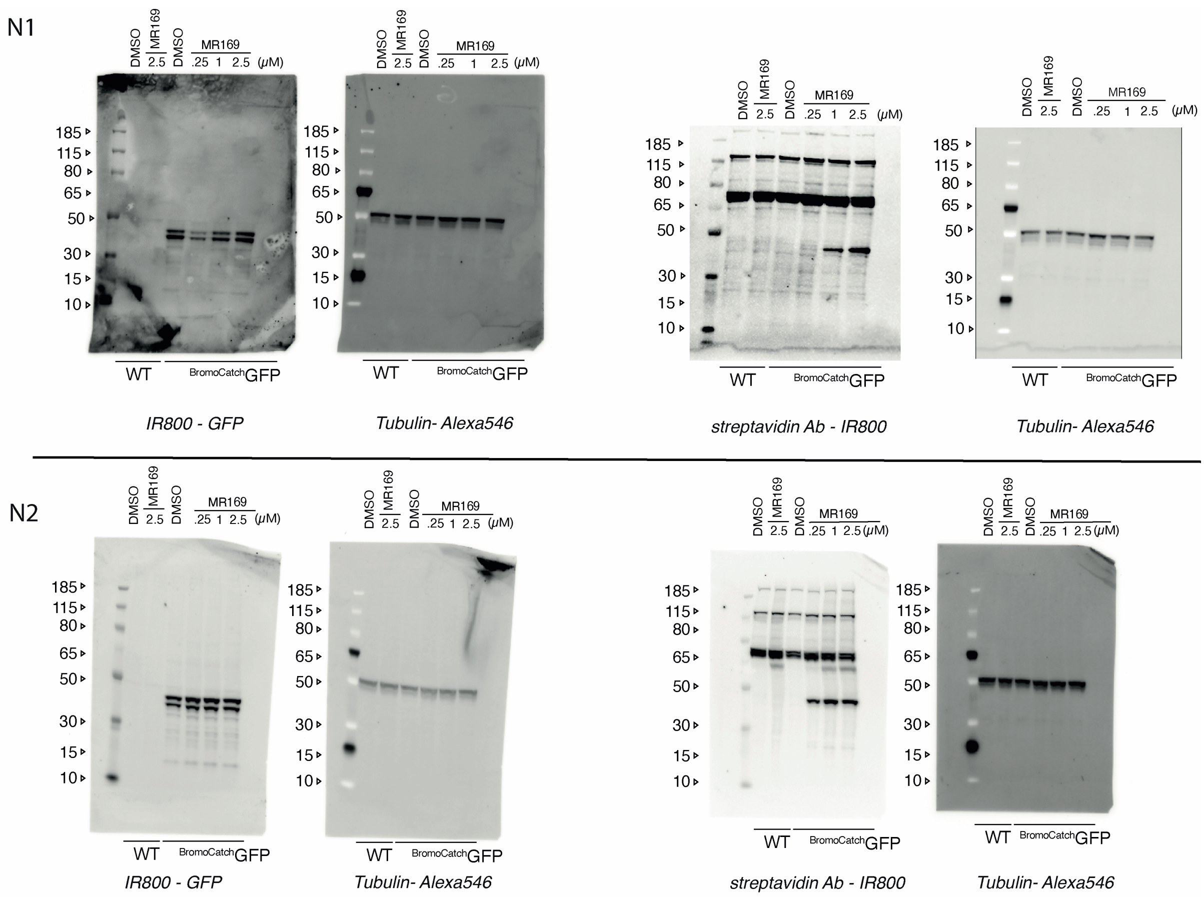

Supplementary Figure 31. Full membrane of two independent repeats of BromoCatch-GFP transfected cells with increasing concentrations of the MR169 probe. Tubulin was imaged with α-tubulin antibody conjugated to Rhodamine, imaged on ChemiDoc in the Alexa546 channel. GFP was labelled with a primary anti-GFP sheep antibody detected with a secondary IR800 dye conjugated antibody and imaged on ChemiDoc in the IR800 channel.

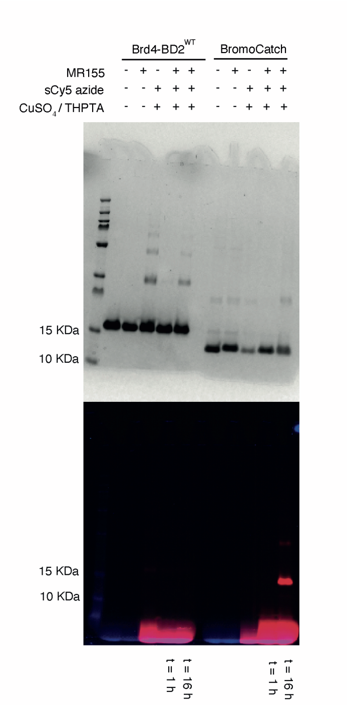

Supplementary Figure 32. Full membrane gel of the click experiment using MR155. Probe was imaged on ChemiDoc in the Cy5 channel. Data is representative of one independent experiment. The experiment was repeated twice with similar results.

HPLC-UV / LC-MS

MR155 – alkyne probe

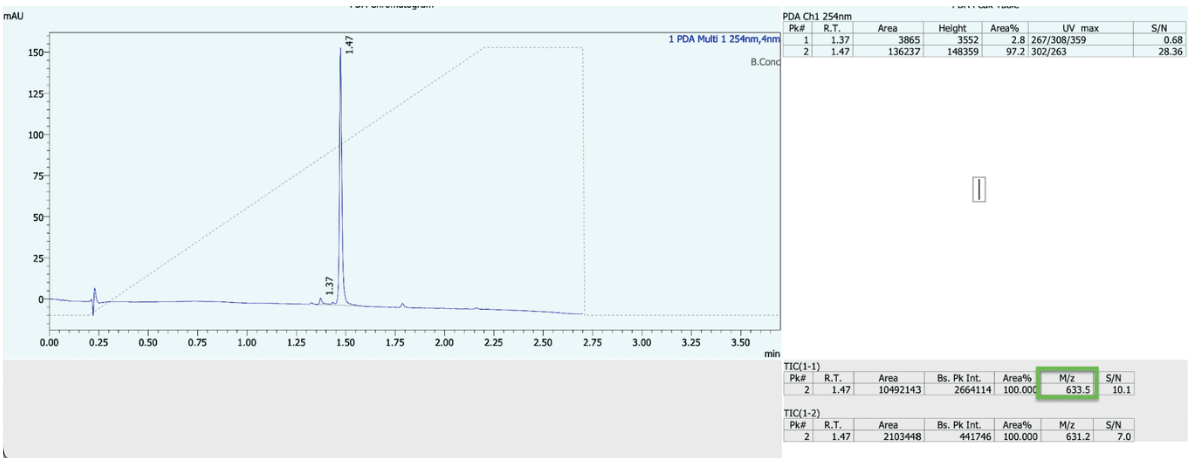

Supplementary Figure 33. HPLC-UV / LC-MS of MR155 (alkyne probe).

MR170 – VHL covalent PROTAC

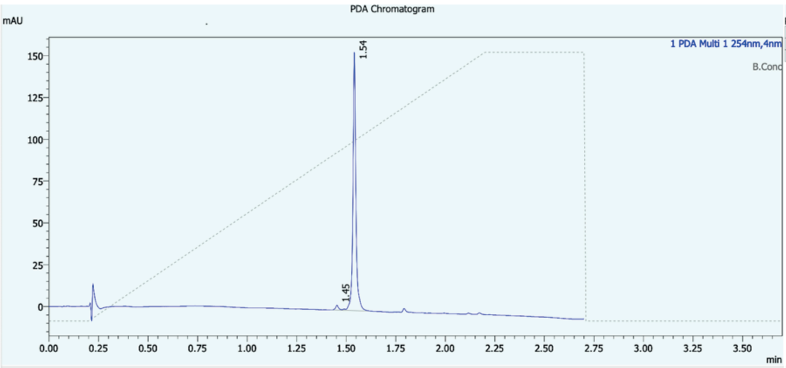

PDA Peak Table

PDA Ch1 254nm

Pk#	R.T.	Area	Height	Area%	UV max	S/N
1	1.45	3269	2772	2.0	268	0.42
2	1.54	162748	144254	98.0	267	21.86

TIC(1-1)

Pk#	R.T.	Area	Bs. Pk Int.	Area%	M/z	S/N
2	1.54	12690074	1316808	100.000	1065.7	18.8

TIC(1-2)

Pk#	R.T.	Area	Bs. Pk Int.	Area%	M/z	S/N
2	1.54	1349209	295332	100.000	1063.9	5.2

Supplementary Figure 34. HPLC-UV / LC-MS of MR170 (PROTAC).

MR202 – TMR probe

UV

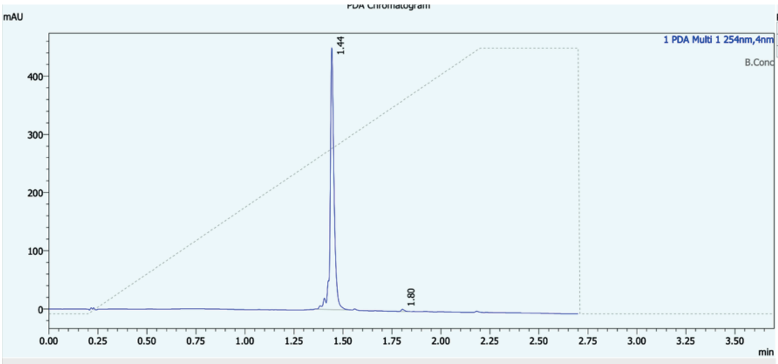

PDA Peak Table

PDA Ch1 254nm

Pk#	R.T.	Area	Height	Area%	UV max	S/N
1	1.44	620390	444275	99.3	556/256/285/195/352	194.52
2	1.80	4125	3635	0.7	259/552/630/660	1.59

MS

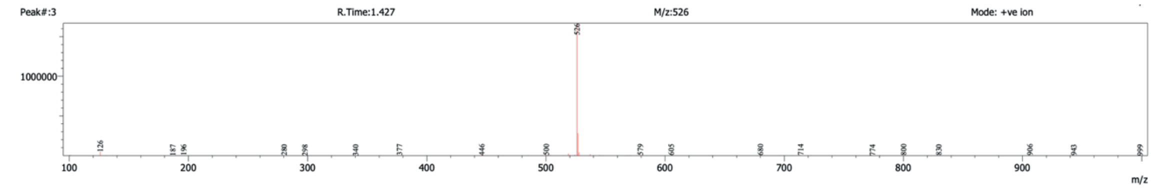

Supplementary Figure 35. HPLC-UV and MS spectra of MR202 (TMR probe).

MR169 – Biotin probe

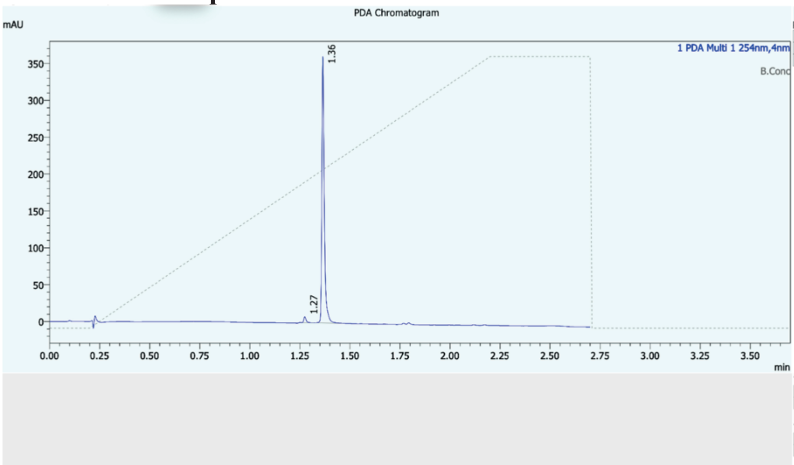

PDA Peak Table

PDA Ch1 254nm

Pk#	R.T.	Area	Height	Area%	UV max	S/N
1	1.27	6706	7961	2.1	267/307/357	1.51
2	1.36	311321	329828	97.9	302/263/231	62.70

TIC(1-1)

Pk#	R.T.	Area	Bs. Pk Int.	Area%	M/z	S/N
2	1.37	28410666	3774974	100.000	908.5	14.3

TIC(1-2)

Pk#	R.T.	Area	Bs. Pk Int.	Area%	M/z	S/N
2	1.36	5157416	714826	100.000	906.5	14.4

Supplementary Figure 36. HPLC-UV / LC-MS of MR169 (biotin probe).

C10852S – Janelia Fluor® 635 probe

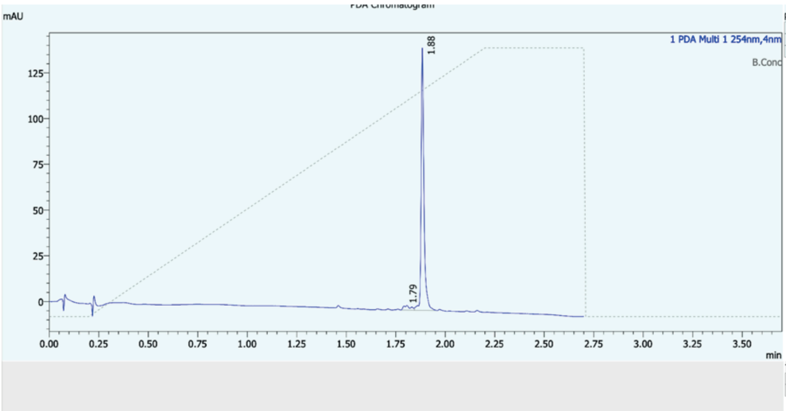

PDA Ch1 254nm

Pk#	R.T.	Area	Height	Area%	UV max	S/N
1	1.79	6136	2119	4.1	258/345	0.60
2	1.88	143051	136651	95.9	267	38.53

TIC(1-1)

Pk#	R.T.	Area	Bs. Pk Int.	Area%	M/z	S/N
2	1.88	2842961	1418341	100.000	577.2	4.4

Supplementary Figure 37. HPLC-UV / LC-MS of C10852S (Janelia Fluor® 635 probe).

C10852L – Janelia Fluor® 635 probe

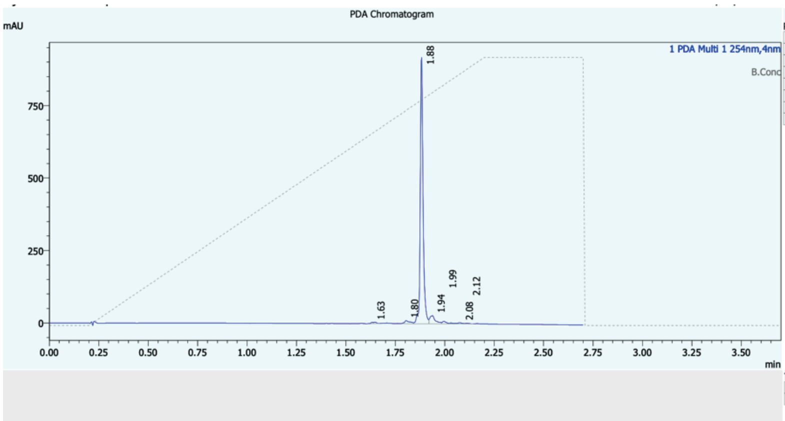

PDA Peak Table

PDA Ch1 254nm

Pk#	R.T.	Area	Height	Area%	UV max	S/N
1	1.63	7176	3949	0.7	266	0.89
2	1.80	20490	10045	2.0	266	2.26
3	1.88	906058	901935	89.9	198/266/223	202.68
4	1.94	52417	27148	5.2	265	6.10
5	1.99	11356	8294	1.1	264	1.86
6	2.08	8694	2885	0.9	268	0.65
7	2.12	2168	1710	0.2	275/371	0.38

TIC(1-1)

Pk#	R.T.	Area	Bs. Pk Int.	Area%	M/z	S/N
3	1.88	4259484	1211253	100.000	511.1	1.3

Supplementary Figure 38. HPLC-UV / LC-MS of C10852L (Janelia Fluor® 635 probe).

References

1. Ramachandran, S. *et al.* Structure-based design of a phosphotyrosine-masked covalent ligand targeting the E3 ligase SOCS2. *Nature Communications* **14**, 6345 (2023).
2. Krippendorff, B. F., Neuhaus, R., Lienau, P., Reichel, A. & Huisinga, W. Mechanism-based inhibition: deriving K(I) and k(inact) directly from time-dependent IC(50) values. *SLAS Discovery* **14**, 913–923 (2009).
3. Mader, L. K., Borean, J. E. and Keillor, J. W. A practical guide for the assay-dependent characterisation of irreversible inhibitors. *RSC Med Chem* **16**, 63–76 (2025).
4. Wilhelm, J. *et al.* Kinetic and structural characterization of the self-labeling protein tags HaloTag7, SNAP-tag, and CLIP-tag. *Biochemistry* **60**, 2560–2575 (2021).
5. Wohlwend, J. *et al.* Boltz-1: Democratizing biomolecular interaction modeling. *bioRxiv* 2024.11.19.624167 (2025) doi:10.1101/2024.11.19.624167.
6. Socolich, M. *et al.* Evolutionary information for specifying a protein fold. *Nature* **437**, 512–518 (2005).
7. Agirre, J. *et al.* The CCP4 suite: integrative software for macromolecular crystallography. *Acta Crystallogr D Struct Biol.* **79**, 449–461 (2023).
