# Supplementary material for: BromoCatch: a self-labelling tag platform for protein modification and live cell imaging
Source: Nat Commun. 2026 May 13;17:6406. doi: 10.1038/s41467-026-72539-w (PMC13376172; doi:10.1038/s41467-026-72539-w)
Supplement: Supplementary file 2 — Description of Additional Supplementary Files [file 41467_2026_72539_MOESM2_ESM.pdf]

**Description of Additional Supplementary Files**

File Name: Supplementary Data 1
Description: Raw data - INTACT LC-MS reports for figures 4, 7b, Sup. Fig. 2, Sup. Fig. 5.

File Name: Supplementary Data 2
Description: Raw data used for proteomics comparative analysis tables.
