# Supplementary material for: BromoCatch: a self-labelling tag platform for protein modification and live cell imaging
Source: Nat Commun. 2026 May 13;17:6406. doi: 10.1038/s41467-026-72539-w (PMC13376172; doi:10.1038/s41467-026-72539-w)

Single Injection Report

Agilent

**Data file:**	261124-10-75OVER20_PEPTIDES-C3_200M-30497_008.D		
**Sequence Name:**	mutant73	**Project Name:**	Walkup Submissions
**Sample name:**	MUT73_MR116_8_26NOV24	**Operator:**	Maria Rodriguez
**Instrument:**		**Injection date:**	2024-11-26 23:48:52-08:00
**Inj. volume:**		**Location:**	P1-D-09
**Acq. method:**	10-75OVER20_PEPTIDES-C3_200MZ.M	**Type:**	Sample
**Processing method:**	*Deconvolution Test 2.pmx	**Sample amount:**	
**Manually modified:**	Manual Integration		

Data Analysis Method: Deconvolution Test 2.pmx
Path: D:\CDSProjects\Walkup Submissions\Results\mutant73.rslt
Method parameters are filtered - only a subset is displayed

2 Method Parameters

2.11 MS Spectral Deconvolution Parameters

**Run automatic deconvolution:**	Yes	**Use RT window:**	No	**TIC peak type:**	All peaks
**TIC peak threshold:**	Top (n) peaks	**Top (n) peaks:**	2	**Positive adduct:**	+H
**Negative adduct:**	-H	**Use m/z range:**	No	**Low molecular weight:**	5000
**High molecular weight:**	25000	**Maximum charge:**	40	**Minimum peaks in set:**	3
**Show unmatched peaks:**	No	**MW agreement (0.01%):**	5	**Absolute noise threshold:**	1000
**Relative abundance threshold (%):**	10	**MW algorithm:**	Curve Fit	**MW algorithm threshold (%):**	40
**Envelope threshold (%):**	50				

Method Audit Trail

Method audit trail is not printed

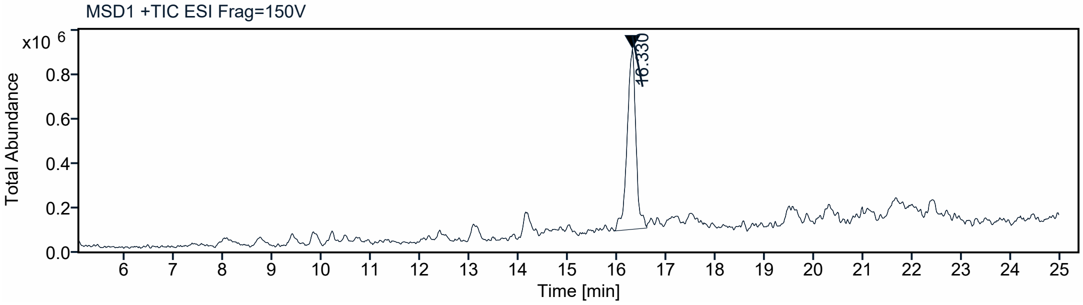

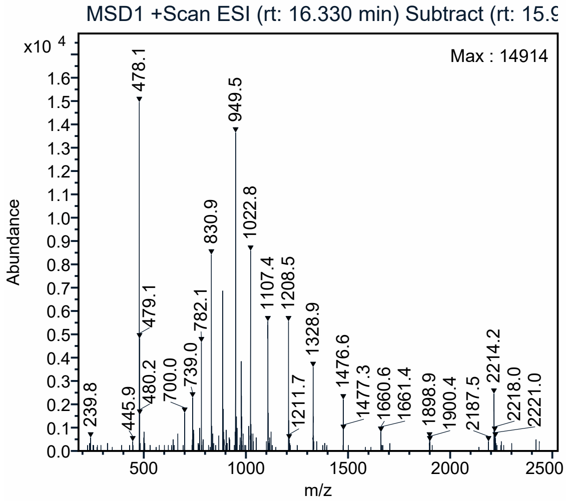

Deconvolution of peak at RT: 16.330

Signal: MSD1 +TIC ESI Frag=150V

Spectrum: MSD1 +Scan ESI (rt: 16.330 min) Subtract (rt: 15.955 min)

No spectra available!

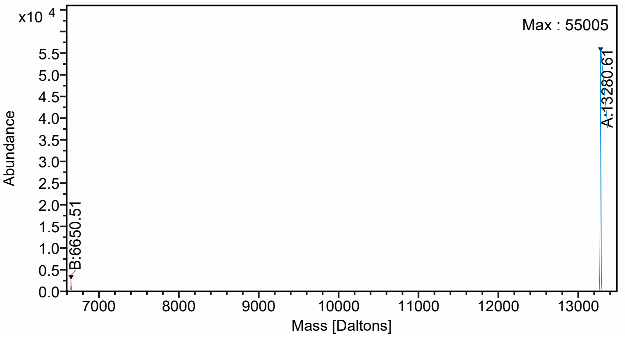

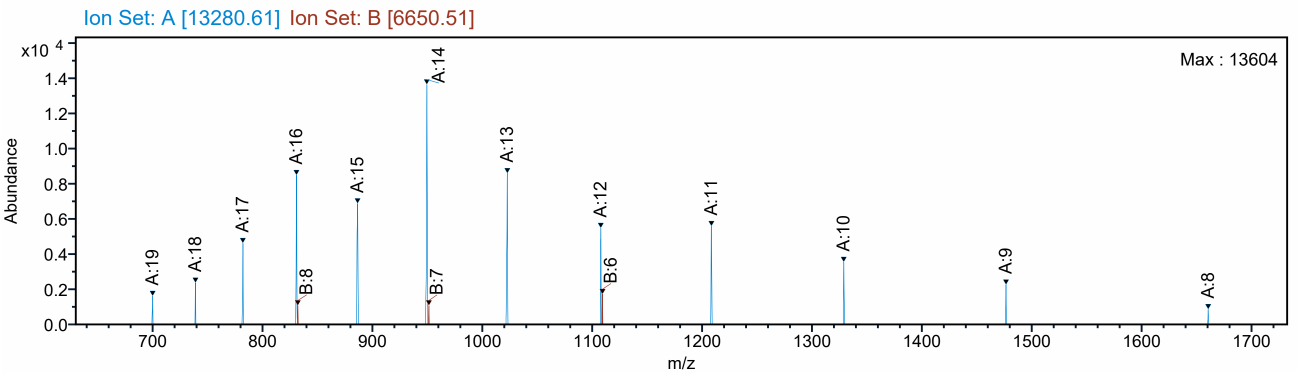

Component	Mass	Absolute Abundance	Relative Abundance (%)	Relative Quantitation (%)
A	13280.61	55005	100.00	95.79
B	6650.51	2419	4.40	4.21

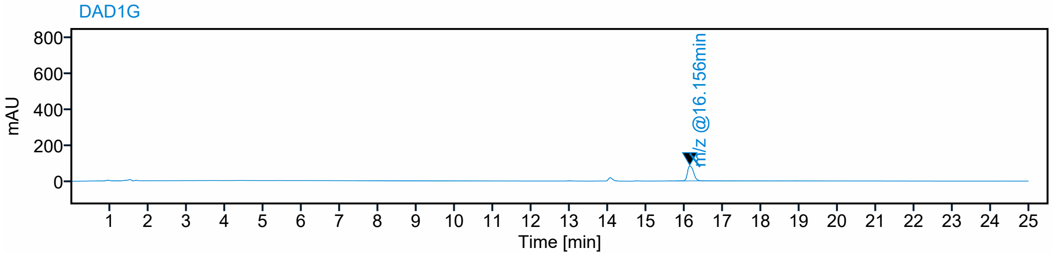

**Signal:**	DAD1C				
**Name**	**RT [min]**	**RF**	**Area**	**Peak Area Percent**	**Group**
	16.226		2183.0409	100.00	

**Signal:**	DAD1G				
**Name**	**RT [min]**	**RF**	**Area**	**Peak Area Percent**	**Group**
	16.156		903.2429	100.00	

**Signal:**	MSD1 +TIC ESI Frag=150V				
**Name**	**RT [min]**	**RF**	**Area**	**Peak Area Percent**	**Group**
	16.330		10003802.2699	100.00	

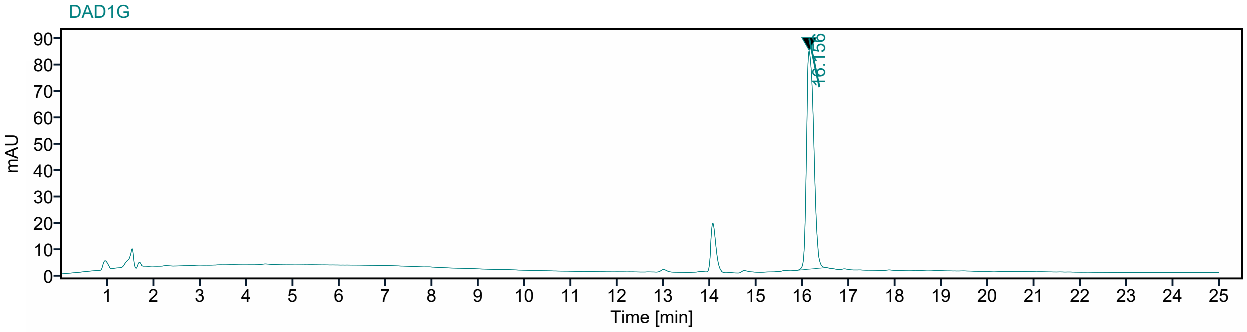

Supplement: Supplementary file 3 — Supplementary Data 1 [file 41467_2026_72539_MOESM3_ESM.zip › PUBLICATION INTACT MS/FIGURE 4A - MS SPECTRUM -DECONVOLUTION - BROMOCATCH MODIFIED.pdf]
