# Supplementary material for: BromoCatch: a self-labelling tag platform for protein modification and live cell imaging
Source: Nat Commun. 2026 May 13;17:6406. doi: 10.1038/s41467-026-72539-w (PMC13376172; doi:10.1038/s41467-026-72539-w)
Supplement: Supplementary file 3 — Supplementary Data 1 [file 41467_2026_72539_MOESM3_ESM.zip › PUBLICATION INTACT MS/Supplementary Figure 5 - 5 min modification.pdf]

Single Injection Report

Agilent

**Data file:**	MR116_5MIN_mutant73.dx		
**Sequence Name:**	SingleSample	**Project Name:**	Walkup Submissions
**Sample name:**	MR116_5MIN_mutant73	**Operator:**	admin (admin)
**Instrument:**	6135C LCMS	**Injection date:**	2025-04-07 10:45:30-08:00
**Inj. volume:**	5.000 µL	**Location:**	D1B-F2
**Acq. method:**	TFA_25min_10-95_C3_small.amx	**Type:**	Sample
**Processing method:**	*Deconv_8-50kDa_26min.pmx	**Sample amount:**	0.00
**Manually modified:**	Manual Integration		

Data Analysis Method: Deconv_8-50kDa_26min.pmx
Path: D:\CDSProjects\Walkup Submissions\Methods
Method parameters are filtered - only a subset is displayed

2 Method Parameters

2.11 MS Spectral Deconvolution Parameters

**Run automatic deconvolution:**	Yes	**Use RT window:**	No	**TIC peak type:**	All peaks
**TIC peak threshold:**	Top (n) peaks	**Top (n) peaks:**	3	**Positive adduct:**	+H
**Negative adduct:**	-H	**Use m/z range:**	No	**Low molecular weight:**	8000
**High molecular weight:**	50000	**Maximum charge:**	50	**Minimum peaks in set:**	3
**Show unmatched peaks:**	No	**MW agreement (0.01%):**	5	**Absolute noise threshold:**	1000
**Relative abundance threshold (%):**	15	**MW algorithm:**	Curve Fit	**MW algorithm threshold (%):**	40
**Envelope threshold (%):**	50				

Method Audit Trail

Method audit trail is not printed

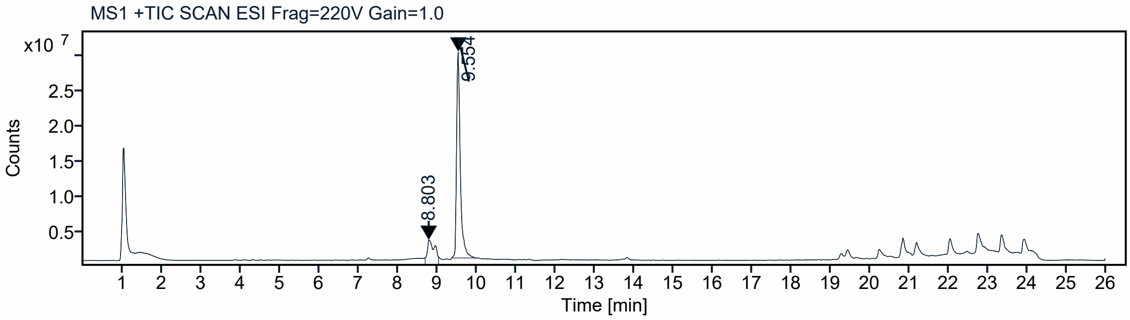

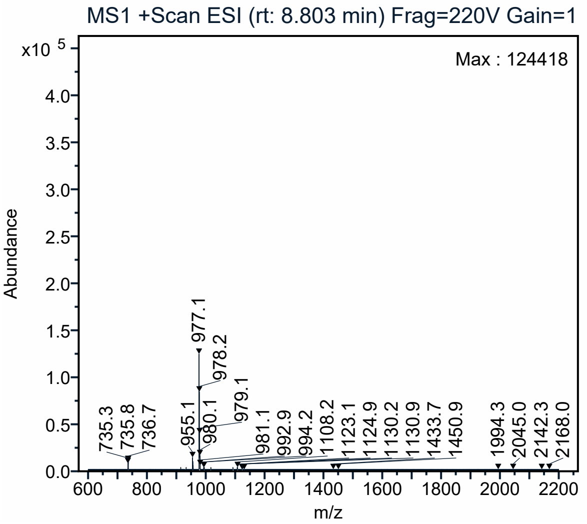

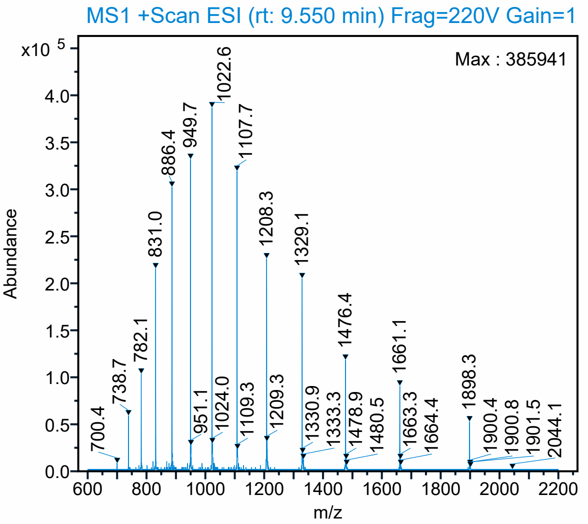

Deconvolution of peak at RT: 9.554

Signal: MS1 +TIC SCAN ESI Frag=220V Gain=1.0

Spectrum: MS1 +Scan ESI (rt: 9.550 min) Frag=220V Gain=1.0 Subtract (rt: 9.404 min)

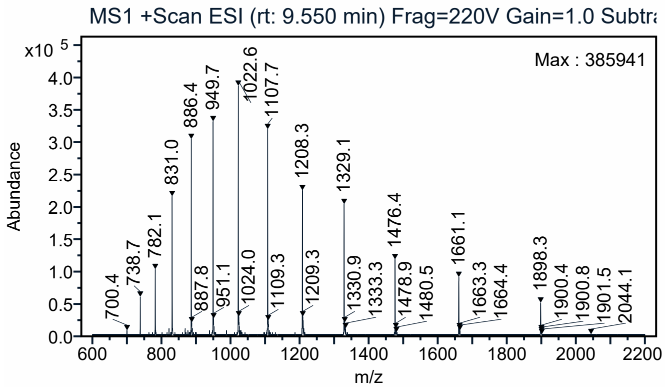

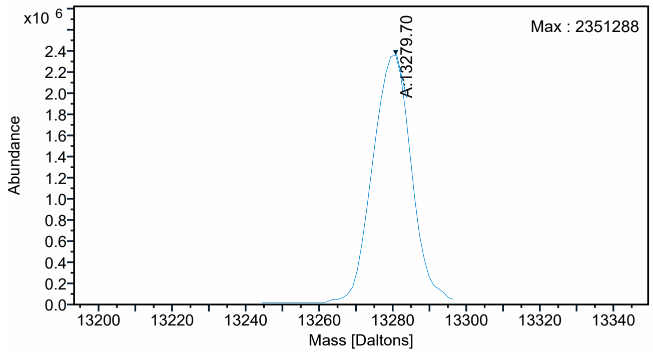

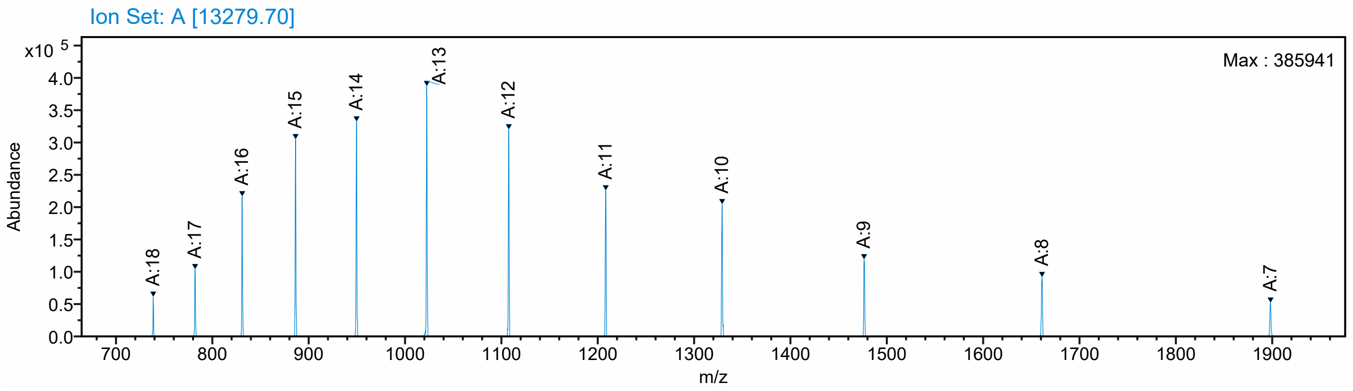

Component	Mass	Absolute Abundance	Relative Abundance (%)	Relative Quantitation (%)
A	13279.70	2351288	100.00	100.00

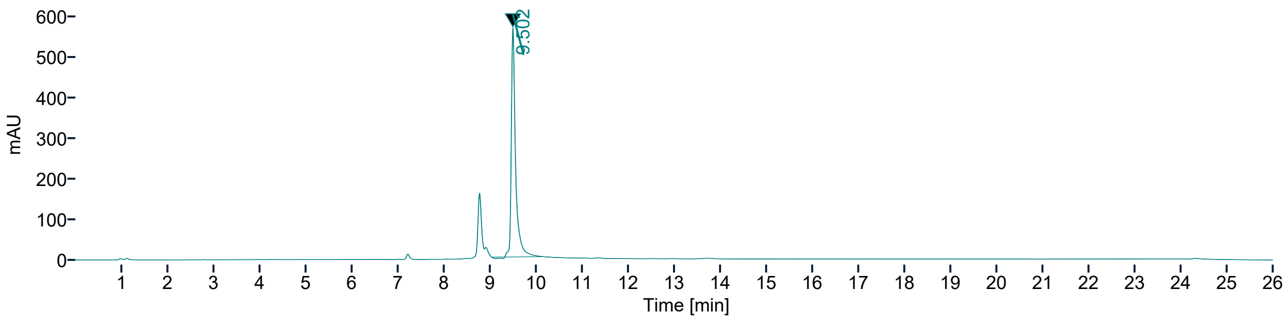

Signal: DAD1B,Sig=280.0,4.0 Ref=360.0,100.0

Name	RT [min]	RF	Area	Peak Area Percent	Group
	9.502		3655.5183	100.00	

Signal: MS1 +TIC SCAN ESI Frag=220V Gain=1.0

Name	RT [min]	RF	Area	Peak Area Percent	Group
	~~8.803~~		~~46840606.5757~~	~~20.03~~	no protein detected
	9.554		186996407.3329	79.97	modified protein
