# Supplementary material for: BromoCatch: a self-labelling tag platform for protein modification and live cell imaging
Source: Nat Commun. 2026 May 13;17:6406. doi: 10.1038/s41467-026-72539-w (PMC13376172; doi:10.1038/s41467-026-72539-w)

Single Injection Report

**Data file:**	061224-10-75OVER20_PEPTIDES-C3_200M-30604_001.D		
**Sequence Name:**	mutant73missing samples	**Project Name:**	
**Sample name:**	MUT73_DMSO_1_6DEC24	**Operator:**	
**Instrument:**		**Injection date:**	
**Inj. volume:**		**Location:**	
**Acq. method:**	10-75OVER20_PEPTIDES-C3_200MZ.M	**Type:**	
**Processing method:**	*Deconvolution Test 2.pmx	**Sample amount:**	
**Manually modified:**	Manual Integration		

Data Analysis Method: Deconvolution Test 2.pmx
Path: D:\CDSProjects\Walkup Submissions\Results\mutant73missing samples.rslt
Method parameters are filtered - only a subset is displayed

2 Method Parameters

2.11 MS Spectral Deconvolution Parameters

**Run automatic deconvolution:**	Yes	**Use RT window:**	No	**TIC peak type:**	All peaks
**TIC peak threshold:**	Top (n) peaks	**Top (n) peaks:**	2	**Positive adduct:**	+H
**Negative adduct:**	-H	**Use m/z range:**	No	**Low molecular weight:**	5000
**High molecular weight:**	25000	**Maximum charge:**	50	**Minimum peaks in set:**	3
**Show unmatched peaks:**	No	**MW agreement (0.01%):**	5	**Absolute noise threshold:**	1000
**Relative abundance threshold (%):**	5	**MW algorithm:**	Curve Fit	**MW algorithm threshold (%):**	40
**Envelope threshold (%):**	50				

Method Audit Trail

Method audit trail is not printed

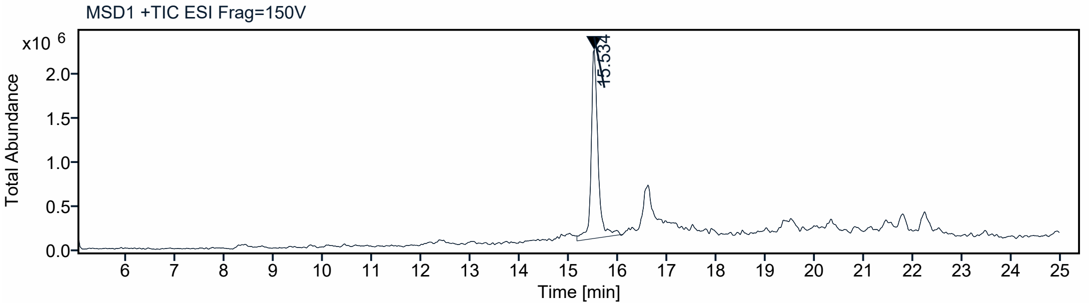

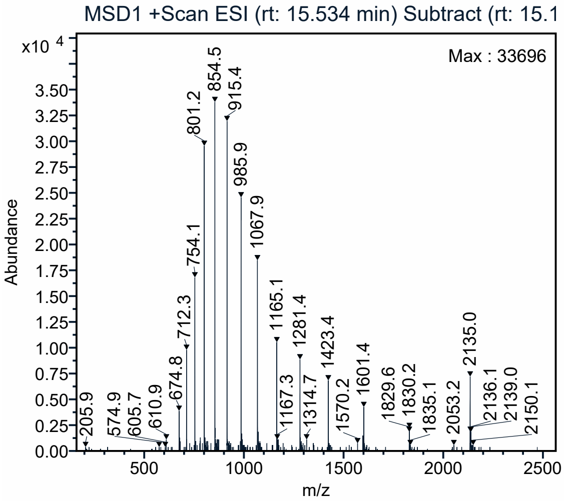

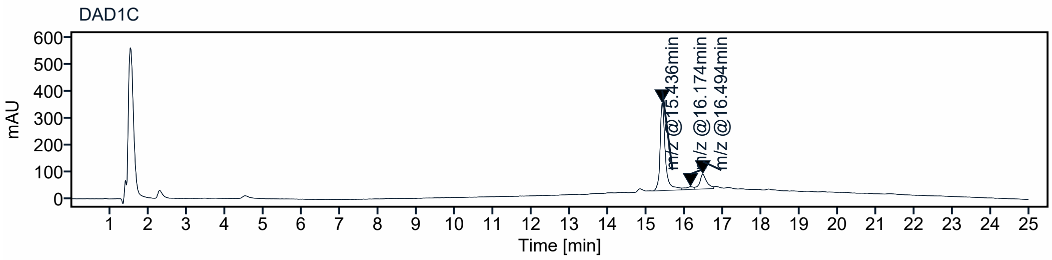

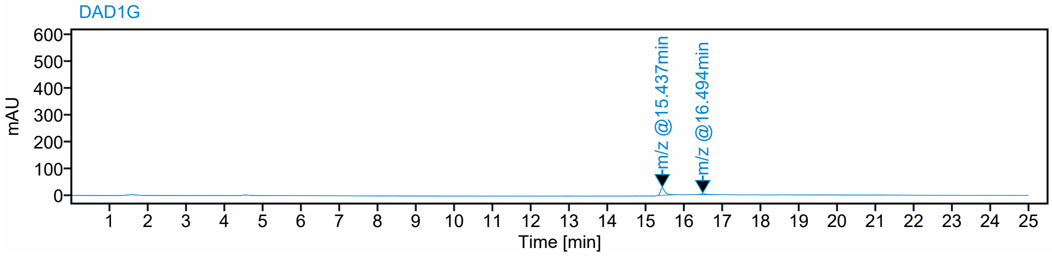

Signal: DAD1C

Name	RT [min]	RF	Area	Peak Area Percent	Group
	15.436		3075.9189	77.98	
	16.174		153.2557	3.89	
	16.494		715.2874	18.13	

Signal: DAD1G

Name	RT [min]	RF	Area	Peak Area Percent	Group
	15.437		228.9989	86.96	

Name	RT [min]	RF	Area	Peak Area Percent	Group
	16.494		34.3438	13.04	

Signal: MSD1 +TIC ESI Frag=150V

Name	RT [min]	RF	Area	Peak Area Percent	Group
	15.534		20593084.7648	100.00	

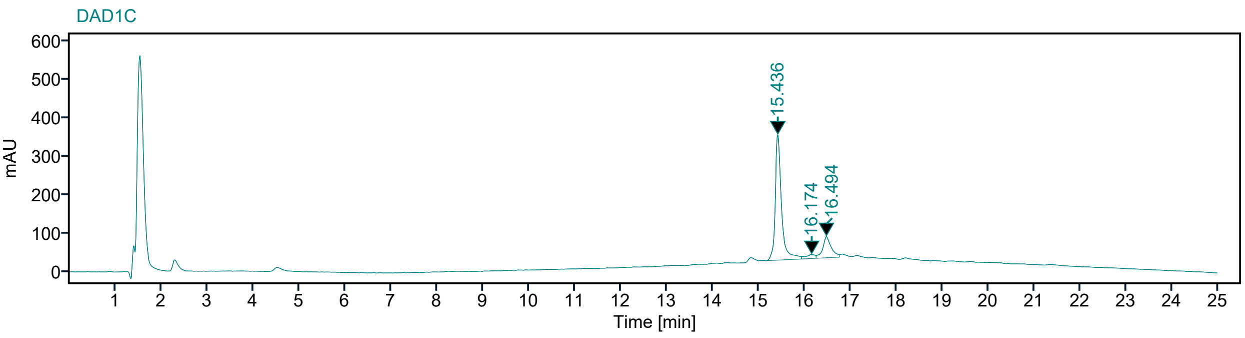

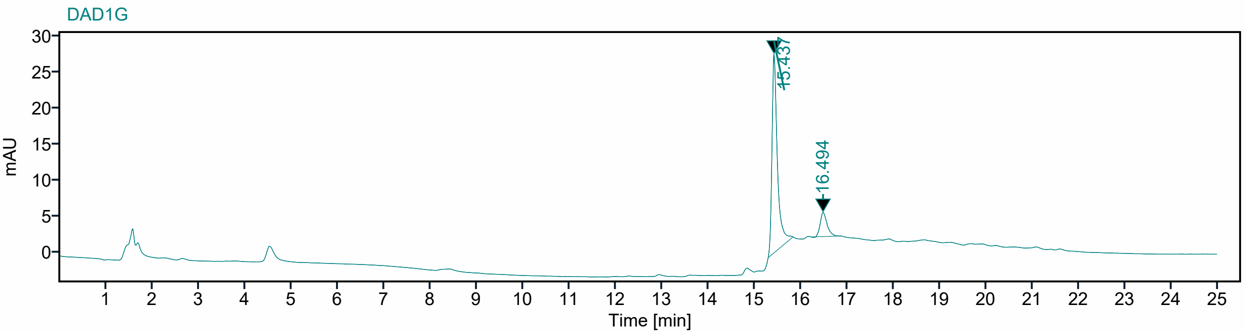

Supplement: Supplementary file 3 — Supplementary Data 1 [file 41467_2026_72539_MOESM3_ESM.zip › PUBLICATION INTACT MS/FIGURE 4A UV - MS SPECTRUM -DECONVOLUTION - BROMOCATCH UNMODIFIED .pdf]
