# Supplementary material for: BromoCatch: a self-labelling tag platform for protein modification and live cell imaging
Source: Nat Commun. 2026 May 13;17:6406. doi: 10.1038/s41467-026-72539-w (PMC13376172; doi:10.1038/s41467-026-72539-w)
Supplement: Supplementary file 3 — Supplementary Data 1 [file 41467_2026_72539_MOESM3_ESM.zip › PUBLICATION INTACT MS/FIGURE 4B - INTACT MS screens .pdf]

Single Injection Report

**Data file:**	090125-10-75OVER20_PEPTIDES-C3_200M-30666_001.D		
**Sequence Name:**	Chemstatioimports_MRR	**Project Name:**	Walkup Submissions
**Sample name:**	Brd4BD2_WT_dmso_9Jan25	**Operator:**	Maria Rodriguez
**Instrument:**		**Injection date:**	2025-01-09 18:12:04-08:00
**Inj. volume:**		**Location:**	P1-E-07
**Acq. method:**	10-75OVER20_PEPTIDES-C3_200MZ.M	**Type:**	Sample
**Processing method:**	*Deconvolution Test 2.pmx	**Sample amount:**	
**Manually modified:**	Manual Integration		

Data Analysis Method: **Deconvolution Test 2.pmx**
Path: **D:\CDSProjects\Walkup Submissions\Results\Chemstatioimports_MRR.rslt**
Method parameters are filtered - only a subset is displayed

2 Method Parameters

2.11 MS Spectral Deconvolution Parameters

**Run automatic deconvolution:**	Yes	**Use RT window:**	No	**TIC peak type:**	All peaks
**TIC peak threshold:**	Top (n) peaks	**Top (n) peaks:**	6	**Positive adduct:**	+H
**Negative adduct:**	-H	**Use m/z range:**	No	**Low molecular weight:**	4000
**High molecular weight:**	25000	**Maximum charge:**	40	**Minimum peaks in set:**	3
**Show unmatched peaks:**	No	**MW agreement (0.01%):**	5	**Absolute noise threshold:**	1000
**Relative abundance threshold (%):**	10	**MW algorithm:**	Curve Fit	**MW algorithm threshold (%):**	40
**Envelope threshold (%):**	50				

Method Audit Trail

Method audit trail is not printed

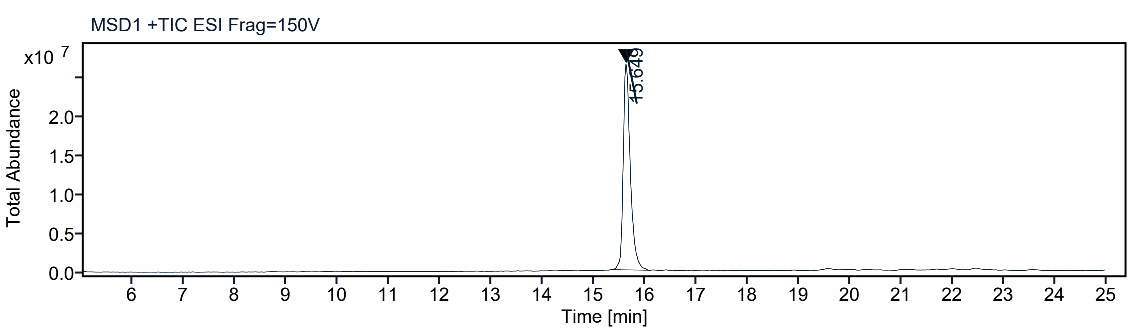

Single Injection Report

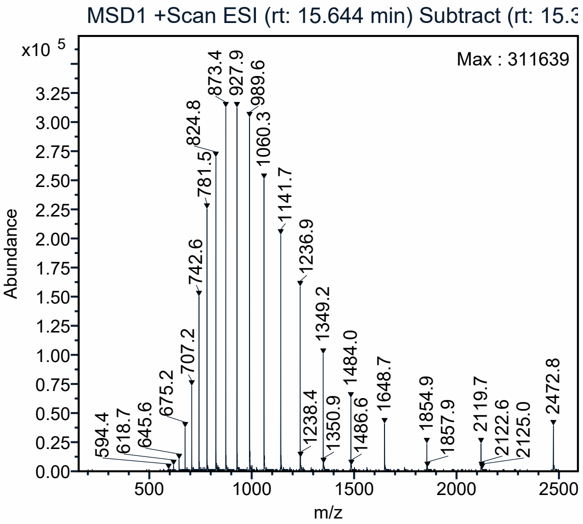

Deconvolution of peak at RT: 15.649

Signal: MSD1 +TIC ESI Frag=150V

Spectrum: MSD1 +Scan ESI (rt: 15.644 min) Subtract (rt: 15.378 min)

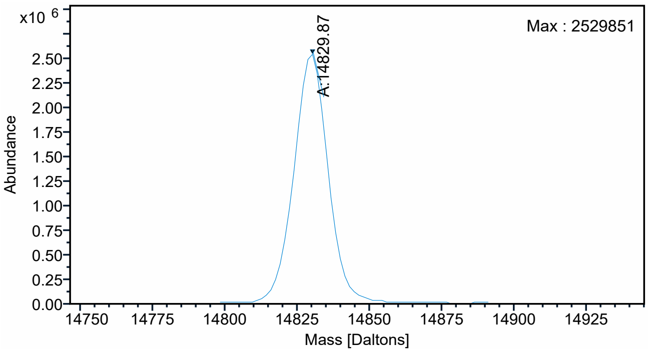

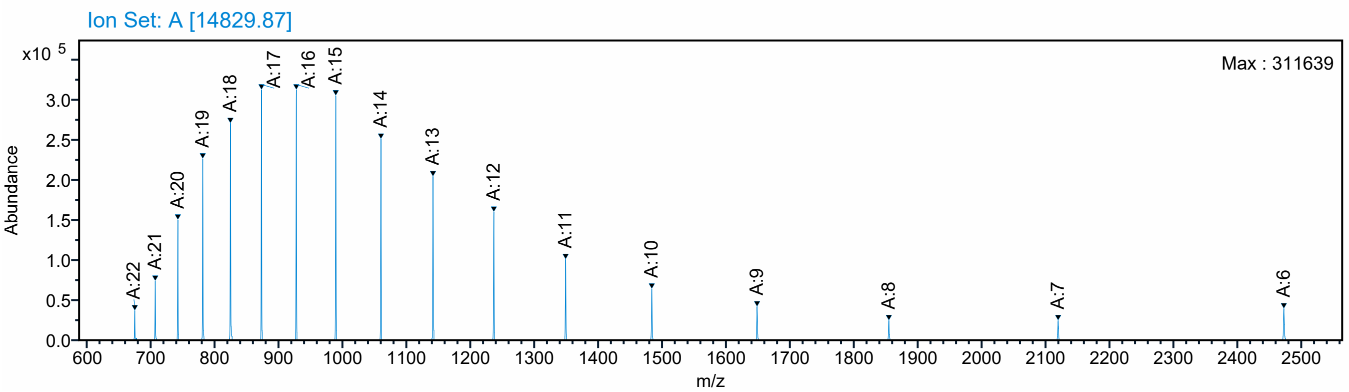

Component	Mass	Absolute Abundance	Relative Abundance (%)	Relative Quantitation (%)
A	14829.87	2529851	100.00	100.00

Single Injection Report

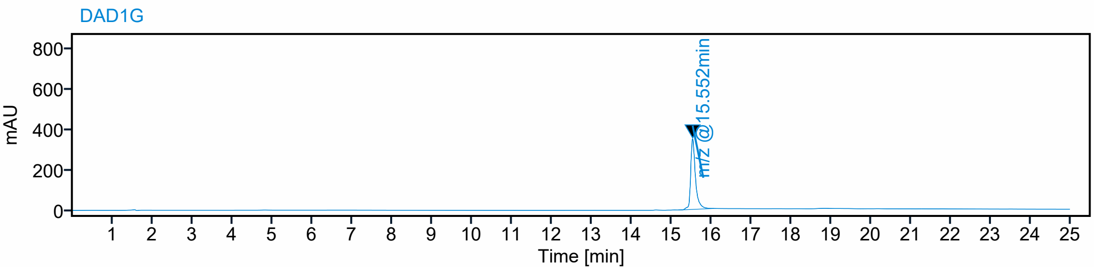

Signal: DAD1G

Name	RT [min]	RF	Area	Peak Area Percent	Group
	15.552		2917.4003	100.00	unmodified protein 100%

Signal: MSD1 +TIC ESI Frag=150V

Name	RT [min]	RF	Area	Peak Area Percent	Group
	15.649		255222230.2163	100.00	unmodified protein 100%

Single Injection Report

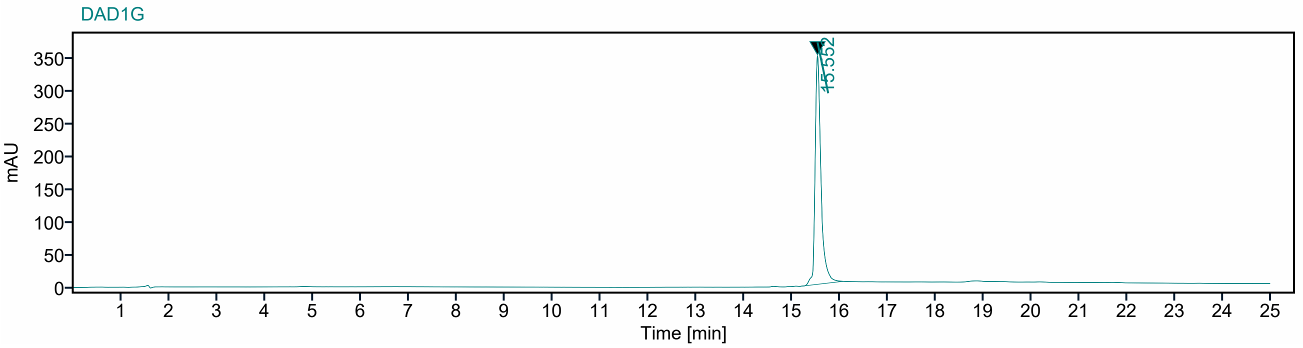

Single Injection Report

**Data file:**	090125-10-75OVER20_PEPTIDES-C3_200M-30667_002.D		
**Sequence Name:**	Chemstatioimports_MRR	**Project Name:**	Walkup Submissions
**Sample name:**	Brd4BD2_WT_etjq1_9Jan25	**Operator:**	Maria Rodriguez
**Instrument:**		**Injection date:**	2025-01-09 18:42:00-08:00
**Inj. volume:**		**Location:**	P1-E-08
**Acq. method:**	10-75OVER20_PEPTIDES-C3_200MZ.M	**Type:**	Sample
**Processing method:**	*Deconvolution Test 2.pmx	**Sample amount:**	
**Manually modified:**	Manual Integration		

Data Analysis Method: **Deconvolution Test 2.pmx**
Path: **D:\CDSProjects\Walkup Submissions\Results\Chemstatioimports_MRR.rslt**
Method parameters are filtered - only a subset is displayed

2 Method Parameters

2.11 MS Spectral Deconvolution Parameters

**Run automatic deconvolution:**	Yes	**Use RT window:**	No	**TIC peak type:**	All peaks
**TIC peak threshold:**	Top (n) peaks	**Top (n) peaks:**	6	**Positive adduct:**	+H
**Negative adduct:**	-H	**Use m/z range:**	No	**Low molecular weight:**	4000
**High molecular weight:**	25000	**Maximum charge:**	40	**Minimum peaks in set:**	3
**Show unmatched peaks:**	No	**MW agreement (0.01%):**	5	**Absolute noise threshold:**	1000
**Relative abundance threshold (%):**	10	**MW algorithm:**	Curve Fit	**MW algorithm threshold (%):**	40
**Envelope threshold (%):**	50				

Method Audit Trail

Method audit trail is not printed

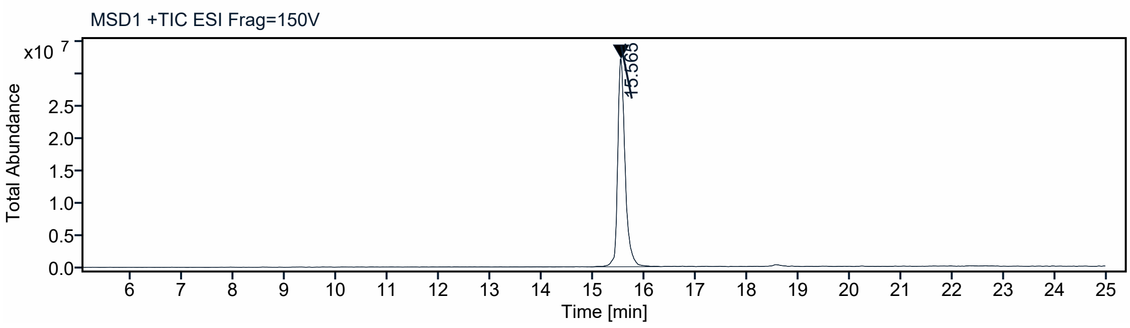

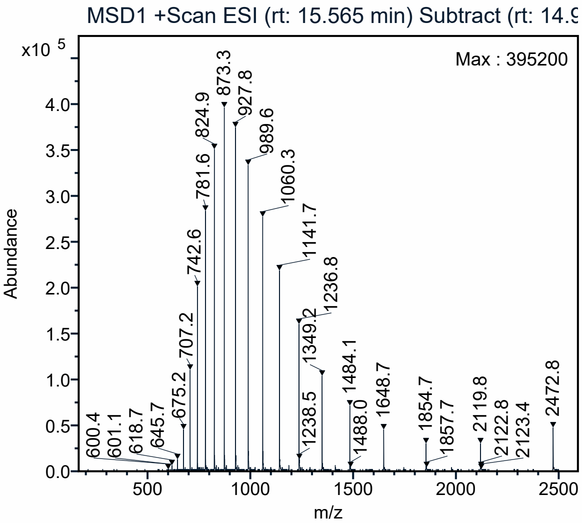

Deconvolution of peak at RT: 15.565

Signal: MSD1 +TIC ESI Frag=150V

Spectrum: MSD1 +Scan ESI (rt: 15.565 min) Subtract (rt: 14.988 min)

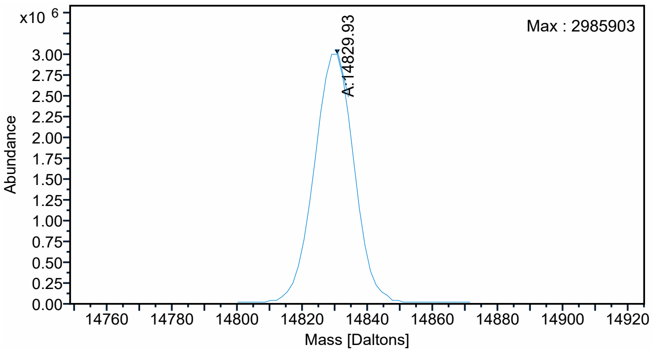

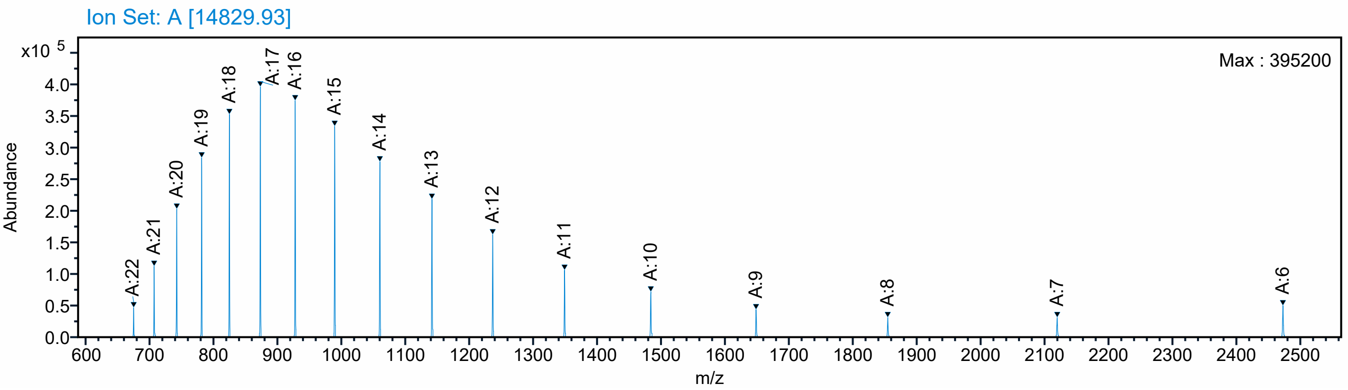

Component	Mass	Absolute Abundance	Relative Abundance (%)	Relative Quantitation (%)
A	14829.93	2985903	100.00	100.00

DAD1C

Time [min]

DAD1G

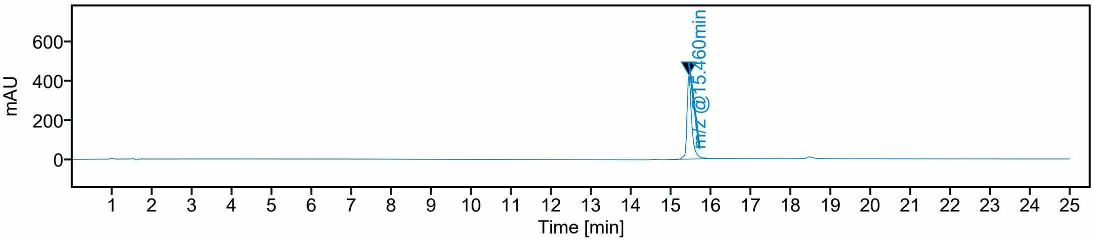

Signal: DAD1G

Name	RT [min]	RF	Area	Peak Area Percent	Group
	15.460		3500.3492	100.00	unmodified protein 100%

Signal: MSD1 +TIC ESI Frag=150V

Name	RT [min]	RF	Area	Peak Area Percent	Group
	15.565		321989617.9758	100.00	unmodified protein 100%

Single Injection Report

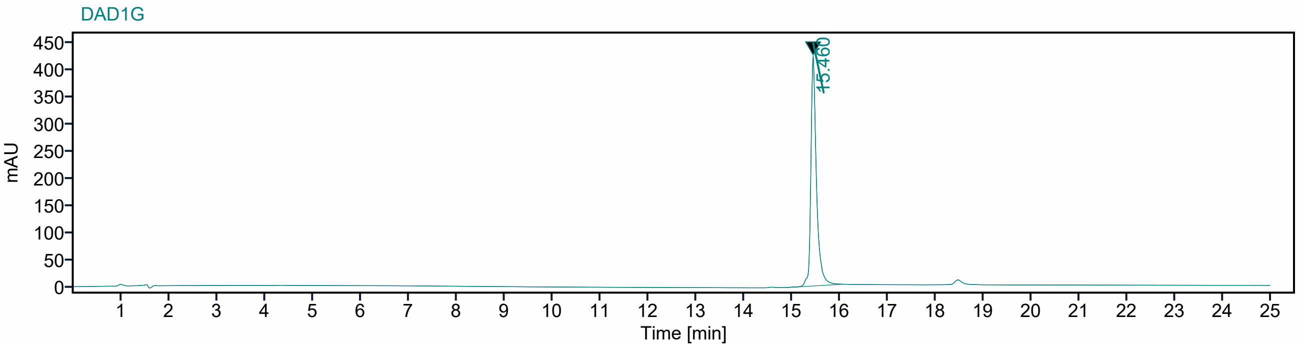

Single Injection Report

**Data file:**	090125-10-75OVER20_PEPTIDES-C3_200M-30668_003.D		
**Sequence Name:**	Chemstatioimports_MRR	**Project Name:**	Walkup Submissions
**Sample name:**	Brd4BD2_WT_mr100_9Jan25	**Operator:**	Maria Rodriguez
**Instrument:**		**Injection date:**	2025-01-09 19:11:50-08:00
**Inj. volume:**		**Location:**	P1-E-09
**Acq. method:**	10-75OVER20_PEPTIDES-C3_200MZ.M	**Type:**	Sample
**Processing method:**	*Deconvolution Test 2.pmx	**Sample amount:**	
**Manually modified:**	Manual Integration		

Data Analysis Method: Deconvolution Test 2.pmx
Path: D:\CDSProjects\Walkup Submissions\Results\Chemstatioimports_MRR.rslt
Method parameters are filtered - only a subset is displayed

2 Method Parameters

2.11 MS Spectral Deconvolution Parameters

**Run automatic deconvolution:**	Yes	**Use RT window:**	No	**TIC peak type:**	All peaks
**TIC peak threshold:**	Top (n) peaks	**Top (n) peaks:**	6	**Positive adduct:**	+H
**Negative adduct:**	-H	**Use m/z range:**	No	**Low molecular weight:**	4000
**High molecular weight:**	25000	**Maximum charge:**	40	**Minimum peaks in set:**	3
**Show unmatched peaks:**	No	**MW agreement (0.01%):**	5	**Absolute noise threshold:**	1000
**Relative abundance threshold (%):**	10	**MW algorithm:**	Curve Fit	**MW algorithm threshold (%):**	40
**Envelope threshold (%):**	50				

Method Audit Trail

Method audit trail is not printed

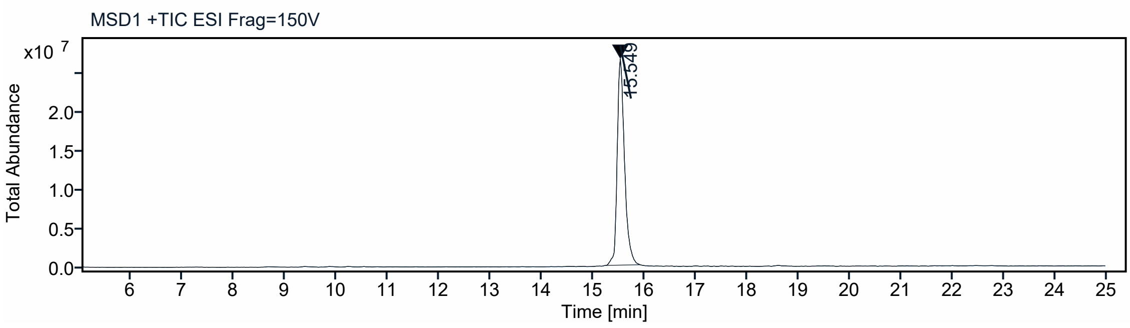

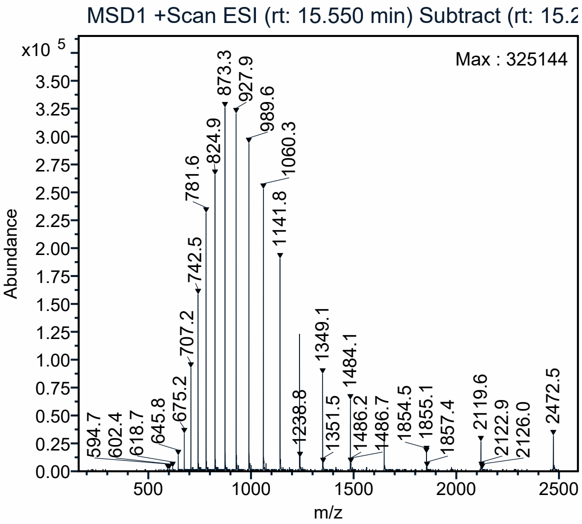

Deconvolution of peak at RT: 15.549

Signal: MSD1 +TIC ESI Frag=150V

Spectrum: MSD1 +Scan ESI (rt: 15.550 min) Subtract (rt: 15.284 min)

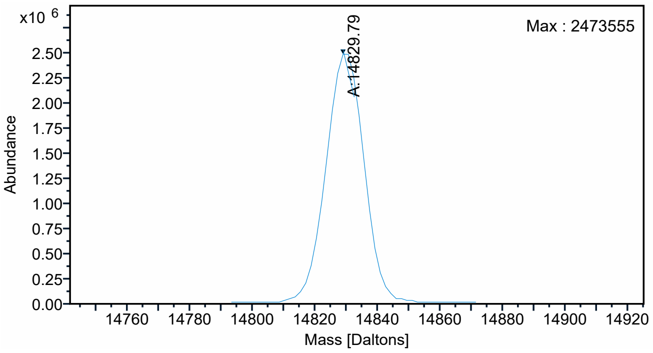

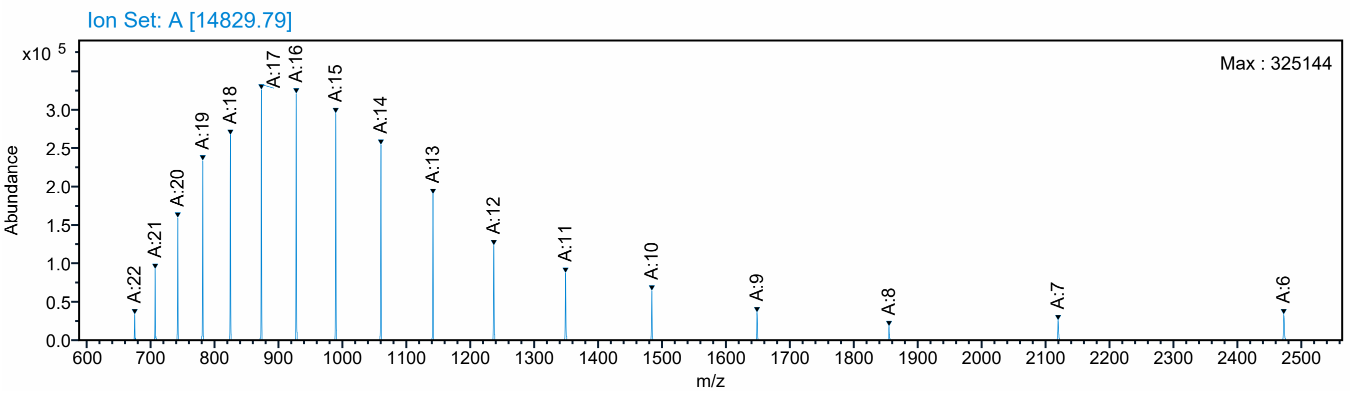

Component	Mass	Absolute Abundance	Relative Abundance (%)	Relative Quantitation (%)
A	14829.79	2473555	100.00	100.00

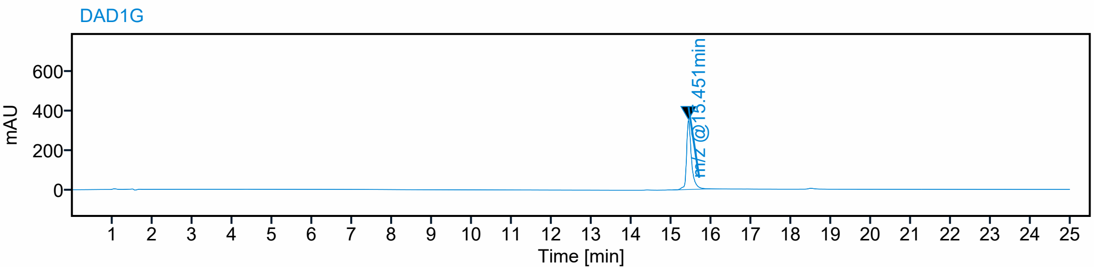

Signal: DAD1C

Name	RT [min]	RF	Area	Peak Area Percent	Group
	15.473		14559.7001	100.00	

unmodified protein 100%

Signal: MSD1 +TIC ESI Frag=150V

Name	RT [min]	RF	Area	Peak Area Percent	Group
	15.549		262097418.2737	100.00	

unmodified protein 100%

Single Injection Report

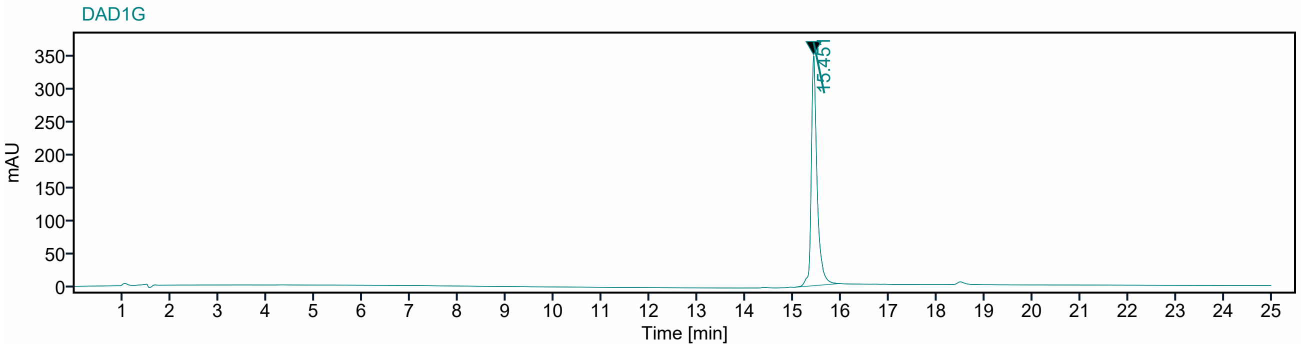

Single Injection Report

**Data file:**	090125-10-75OVER20_PEPTIDES-C3_200M-30669_004.D		
**Sequence Name:**	Chemstatioimports_MRR	**Project Name:**	Walkup Submissions
**Sample name:**	Brd4BD2_WT_mr101_9Jan25	**Operator:**	Maria Rodriguez
**Instrument:**		**Injection date:**	2025-01-09 19:41:39-08:00
**Inj. volume:**		**Location:**	P1-F-01
**Acq. method:**	10-75OVER20_PEPTIDES-C3_200MZ.M	**Type:**	Sample
**Processing method:**	*Deconvolution Test 2.pmx	**Sample amount:**	
**Manually modified:**	Manual Integration		

Data Analysis Method: **Deconvolution Test 2.pmx**
Path: **D:\CDSProjects\Walkup Submissions\Results\Chemstatioimports_MRR.rslt**
Method parameters are filtered - only a subset is displayed

2 Method Parameters

2.11 MS Spectral Deconvolution Parameters

**Run automatic deconvolution:**	Yes	**Use RT window:**	No	**TIC peak type:**	All peaks
**TIC peak threshold:**	Top (n) peaks	**Top (n) peaks:**	6	**Positive adduct:**	+H
**Negative adduct:**	-H	**Use m/z range:**	No	**Low molecular weight:**	4000
**High molecular weight:**	25000	**Maximum charge:**	40	**Minimum peaks in set:**	3
**Show unmatched peaks:**	No	**MW agreement (0.01%):**	5	**Absolute noise threshold:**	1000
**Relative abundance threshold (%):**	10	**MW algorithm:**	Curve Fit	**MW algorithm threshold (%):**	40
**Envelope threshold (%):**	50				

Method Audit Trail

Method audit trail is not printed

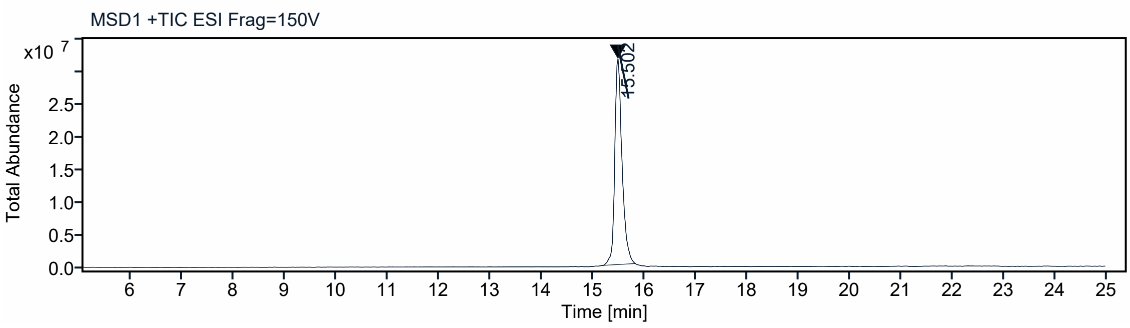

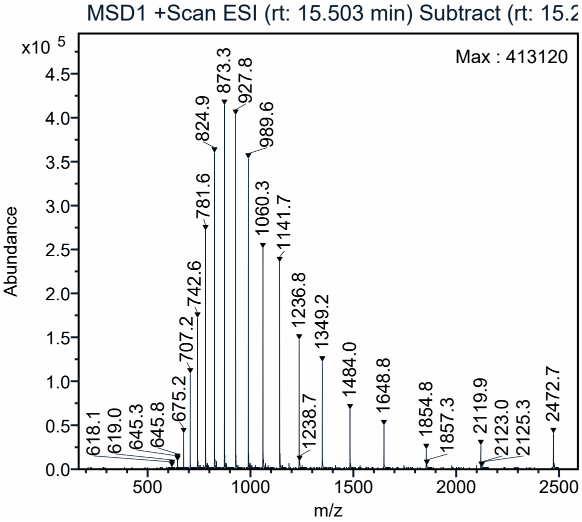

Deconvolution of peak at RT: 15.502

Signal: MSD1 +TIC ESI Frag=150V

Spectrum: MSD1 +Scan ESI (rt: 15.503 min) Subtract (rt: 15.207 min)

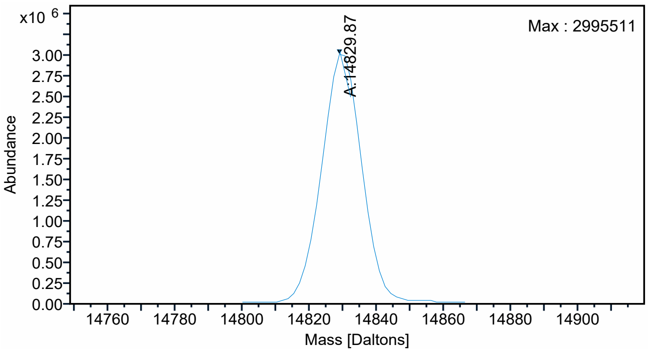

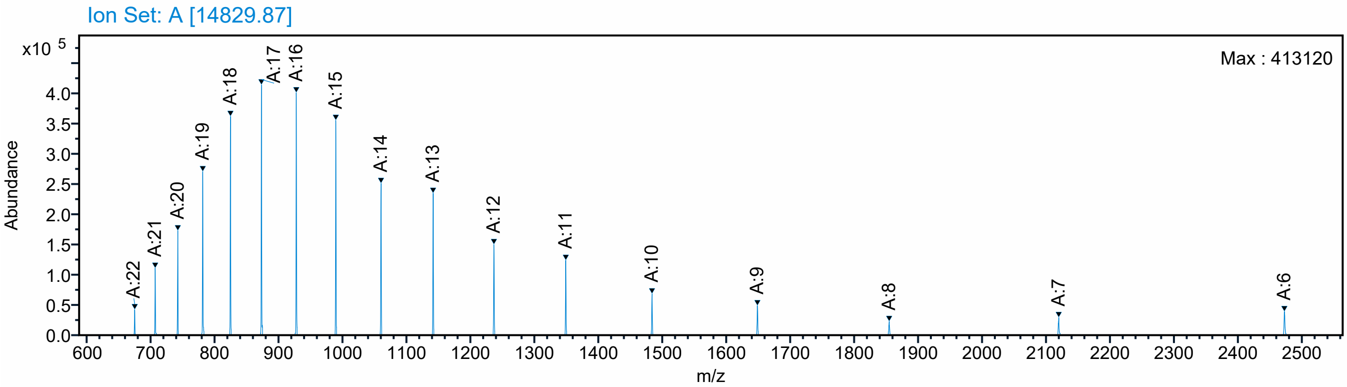

Component	Mass	Absolute Abundance	Relative Abundance (%)	Relative Quantitation (%)
A	14829.87	2995511	100.00	100.00

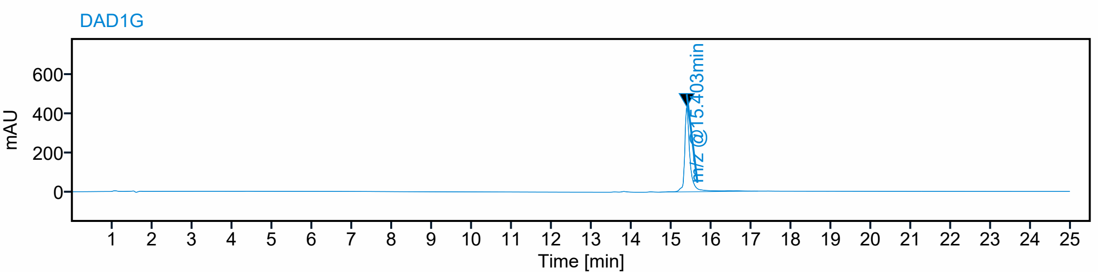

Signal: DAD1G

Name	RT [min]	RF	Area	Peak Area Percent	Group
	15.403		4008.5270	100.00	unmodified protein 100%

Signal: MSD1 +TIC ESI Frag=150V

Name	RT [min]	RF	Area	Peak Area Percent	Group
	15.502		309603219.8423	100.00	unmodified protein 100%

Single Injection Report

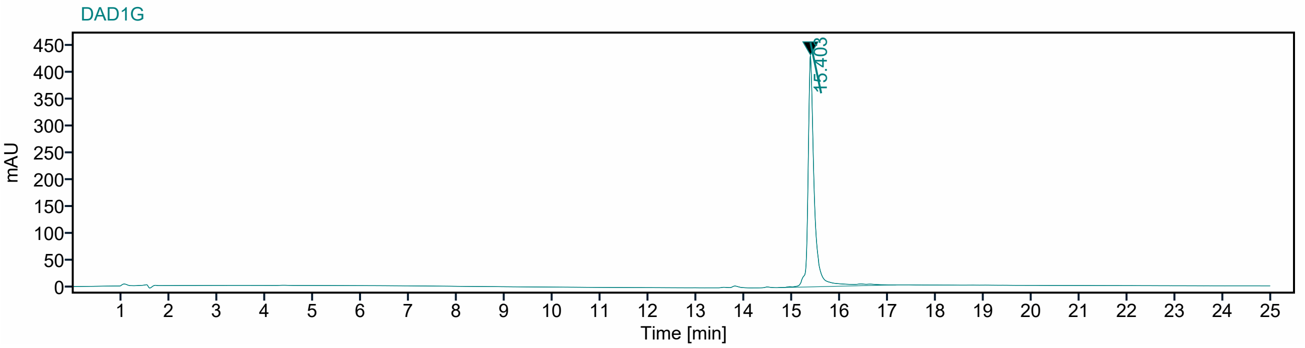

Single Injection Report

**Data file:**	090125-10-75OVER20_PEPTIDES-C3_200M-30670_005.D		
**Sequence Name:**	Chemstatioimports_MRR	**Project Name:**	Walkup Submissions
**Sample name:**	Brd4BD2_WT_mr70_9Jan25	**Operator:**	Maria Rodriguez
**Instrument:**		**Injection date:**	2025-01-09 20:11:30-08:00
**Inj. volume:**		**Location:**	P1-F-02
**Acq. method:**	10-75OVER20_PEPTIDES-C3_200MZ.M	**Type:**	Sample
**Processing method:**	*Deconvolution Test 2.pmx	**Sample amount:**	
**Manually modified:**	Manual Integration		

Data Analysis Method: Deconvolution Test 2.pmx
Path: D:\CDSProjects\Walkup Submissions\Results\Chemstatioimports_MRR.rslt
Method parameters are filtered - only a subset is displayed

2 Method Parameters

2.11 MS Spectral Deconvolution Parameters

**Run automatic deconvolution:**	Yes	**Use RT window:**	No	**TIC peak type:**	All peaks
**TIC peak threshold:**	Top (n) peaks	**Top (n) peaks:**	6	**Positive adduct:**	+H
**Negative adduct:**	-H	**Use m/z range:**	No	**Low molecular weight:**	4000
**High molecular weight:**	25000	**Maximum charge:**	40	**Minimum peaks in set:**	3
**Show unmatched peaks:**	No	**MW agreement (0.01%):**	5	**Absolute noise threshold:**	1000
**Relative abundance threshold (%):**	10	**MW algorithm:**	Curve Fit	**MW algorithm threshold (%):**	40
**Envelope threshold (%):**	50				

Method Audit Trail

Method audit trail is not printed

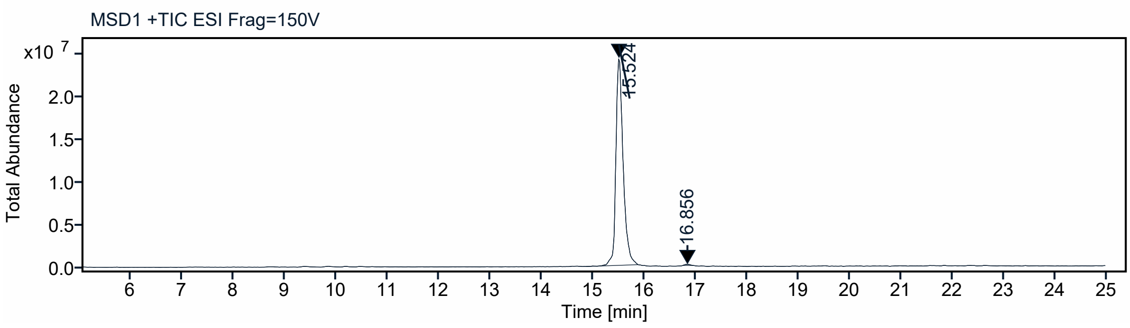

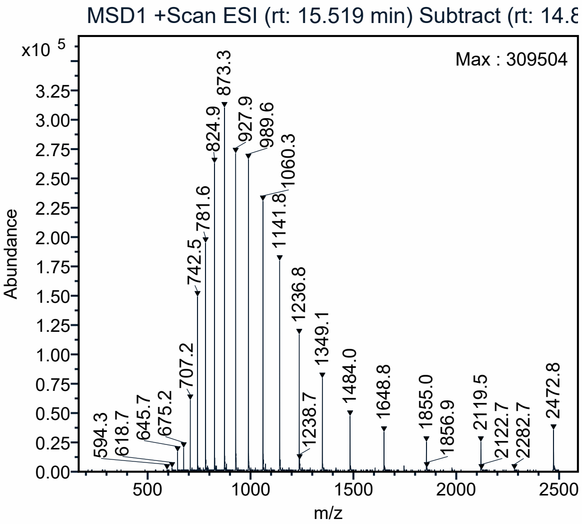

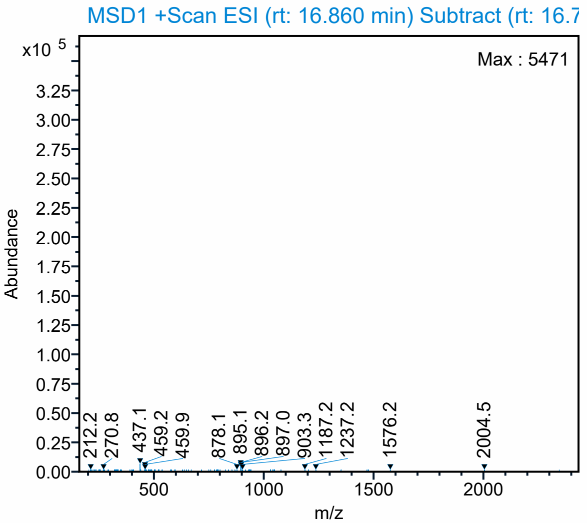

Deconvolution of peak at RT: 15.524

Signal: MSD1 +TIC ESI Frag=150V

Spectrum: MSD1 +Scan ESI (rt: 15.519 min) Subtract (rt: 14.894 min)

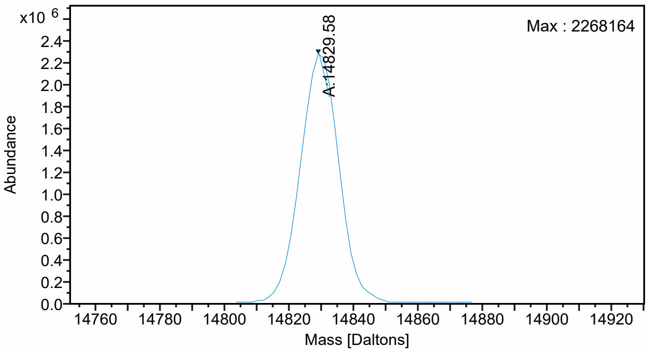

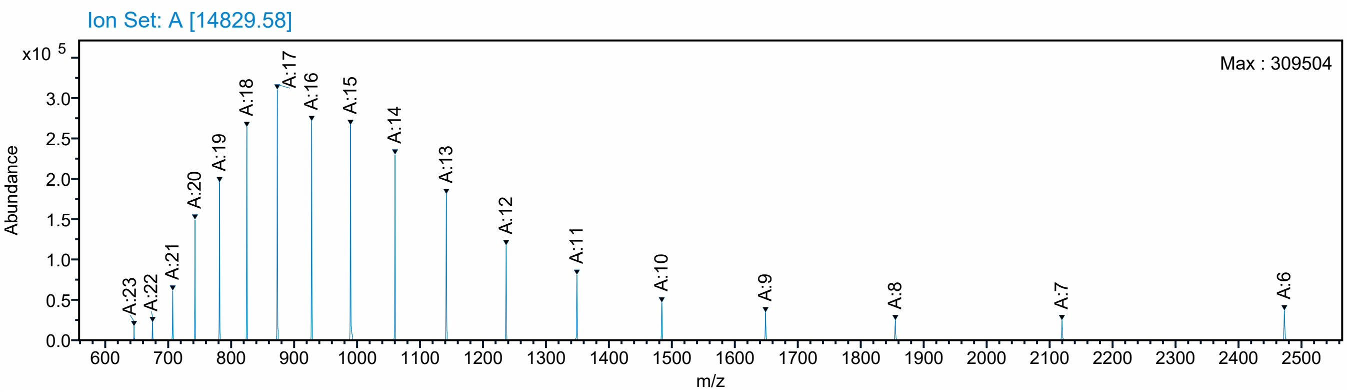

Component	Mass	Absolute Abundance	Relative Abundance (%)	Relative Quantitation (%)
A	14829.58	2268164	100.00	100.00

Single Injection Report

1C

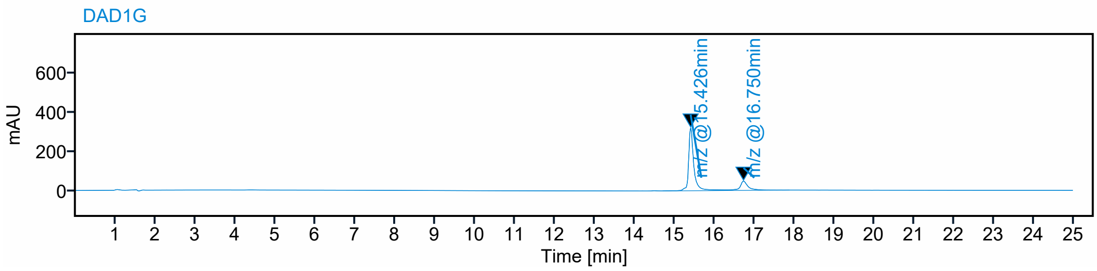

Signal: DAD1G

Name	RT [min]	RF	Area	Peak Area Percent	Group
	15.426		2915.8193	80.70	unmodified protein 100%
	~~16.750~~		~~697.2440~~	~~19.30~~	no protein detected

Signal: MSD1 +TIC ESI Frag=150V

Name	RT [min]	RF	Area	Peak Area Percent	Group
	15.524		235627627.1777	99.60	unmodified protein 100%
	~~16.856~~		~~943160.3688~~	~~0.40~~	no protein detected

Single Injection Report

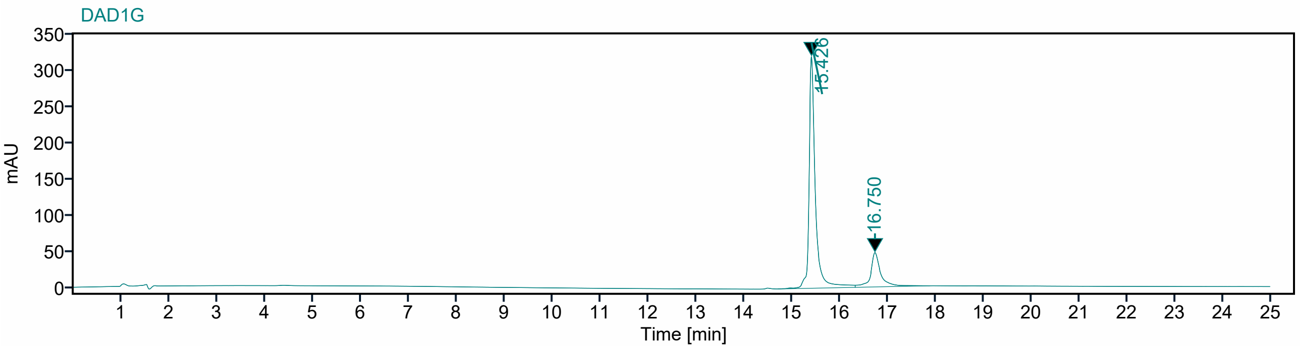

Single Injection Report

**Data file:**	090125-10-75OVER20_PEPTIDES-C3_200M-30671_006.D		
**Sequence Name:**	Chemstatioimports_MRR	**Project Name:**	Walkup Submissions
**Sample name:**	Brd4BD2_WT_mr112_9Jan25	**Operator:**	Maria Rodriguez
**Instrument:**		**Injection date:**	2025-01-09 20:41:20-08:00
**Inj. volume:**		**Location:**	P1-F-03
**Acq. method:**	10-75OVER20_PEPTIDES-C3_200MZ.M	**Type:**	Sample
**Processing method:**	*Deconvolution Test 2.pmx	**Sample amount:**	
**Manually modified:**	Manual Integration		

Data Analysis Method: **Deconvolution Test 2.pmx**
Path: **D:\CDSProjects\Walkup Submissions\Results\Chemstatioimports_MRR.rslt**
Method parameters are filtered - only a subset is displayed

2 Method Parameters

2.11 MS Spectral Deconvolution Parameters

**Run automatic deconvolution:**	Yes	**Use RT window:**	No	**TIC peak type:**	All peaks
**TIC peak threshold:**	Top (n) peaks	**Top (n) peaks:**	6	**Positive adduct:**	+H
**Negative adduct:**	-H	**Use m/z range:**	No	**Low molecular weight:**	4000
**High molecular weight:**	25000	**Maximum charge:**	40	**Minimum peaks in set:**	3
**Show unmatched peaks:**	No	**MW agreement (0.01%):**	5	**Absolute noise threshold:**	1000
**Relative abundance threshold (%):**	10	**MW algorithm:**	Curve Fit	**MW algorithm threshold (%):**	40
**Envelope threshold (%):**	50				

Method Audit Trail

Method audit trail is not printed

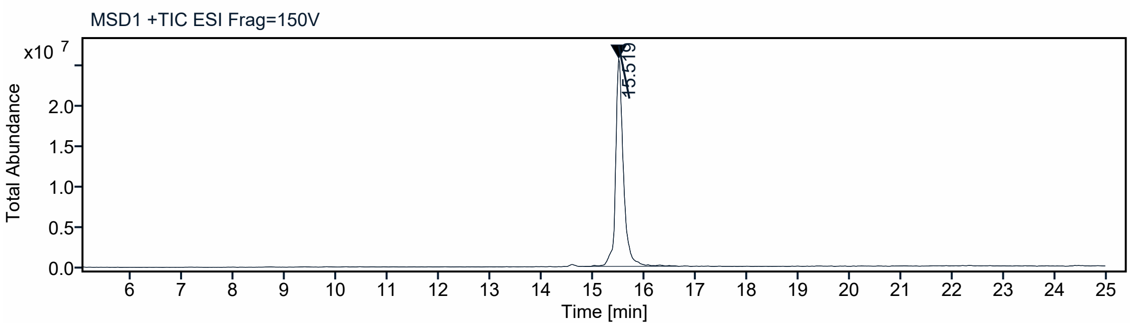

Single Injection Report

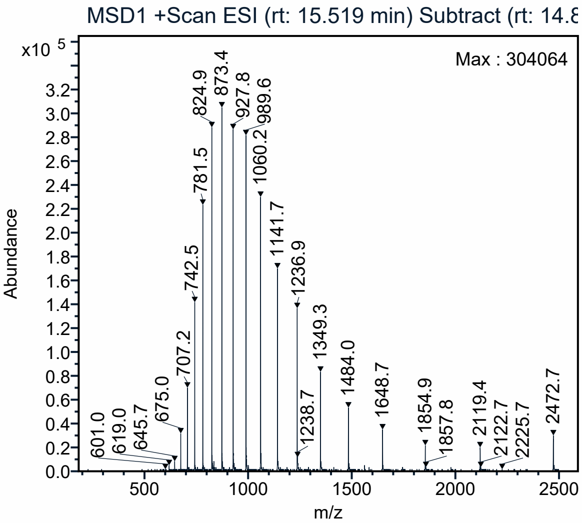

Deconvolution of peak at RT: 15.519

Signal: MSD1 +TIC ESI Frag=150V

Spectrum: MSD1 +Scan ESI (rt: 15.519 min) Subtract (rt: 14.863 min)

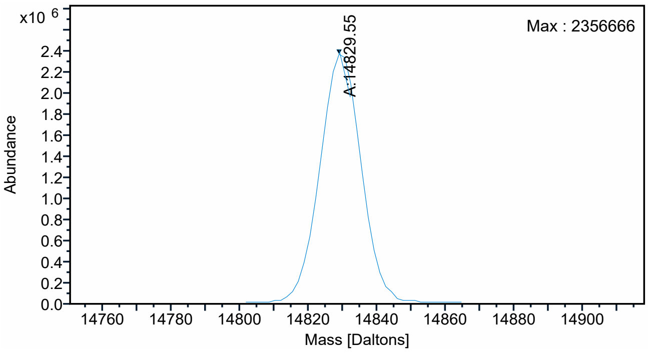

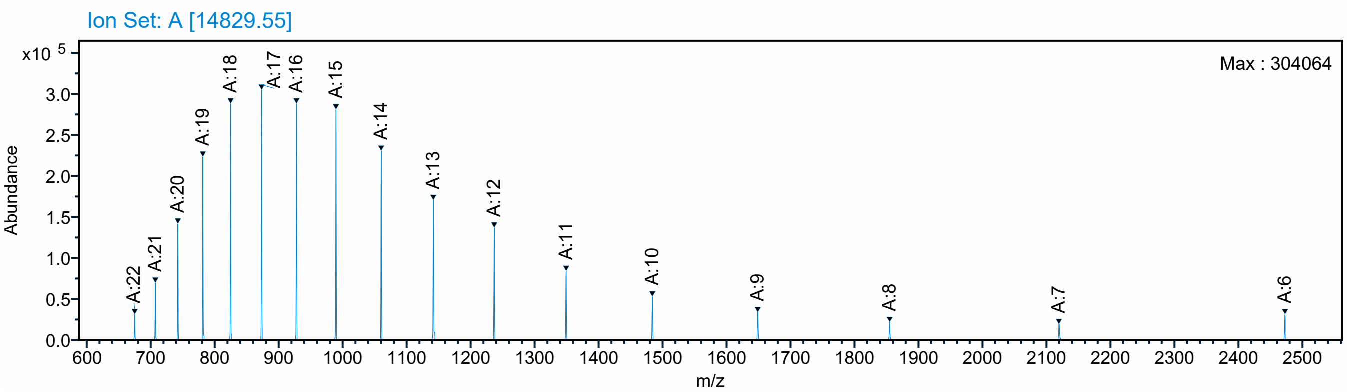

Component	Mass	Absolute Abundance	Relative Abundance (%)	Relative Quantitation (%)
A	14829.55	2356666	100.00	100.00

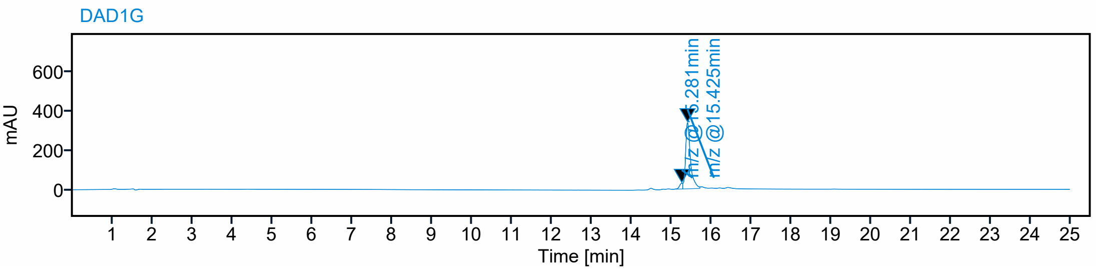

Signal: DAD1G

Name	RT [min]	RF	Area	Peak Area Percent	Group
	~~15.281~~		~~153.1356~~	~~5.10~~	no protein detected
	15.425		2849.0907	94.90	unmodified protein 100%

Signal: MSD1 +TIC ESI Frag=150V

Name	RT [min]	RF	Area	Peak Area Percent	Group
	15.519		270421975.9984	100.00	unmodified protein 100%

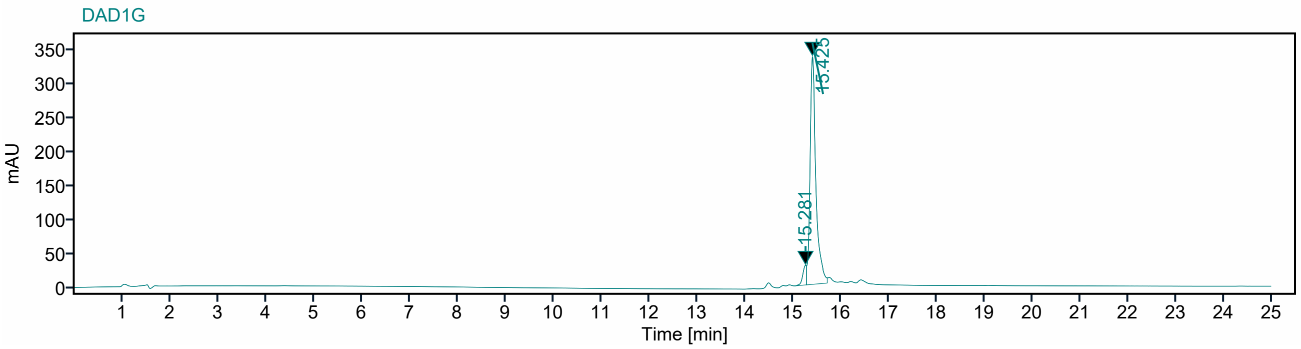
DAD1G
mAU
350
300
250
200
150
100
50
0
15.425
15.281
1
2
3
4
5
6
7
8
9
10
11
12
13
14
15
16
17
18
19
20
21
22
23
24
25
Time [min]

Single Injection Report

**Data file:**	090125-10-75OVER20_PEPTIDES-C3_200M-30672_007.D		
**Sequence Name:**	Chemstatioimports_MRR	**Project Name:**	Walkup Submissions
**Sample name:**	Brd4BD2_WT_mr121_9Jan25	**Operator:**	Maria Rodriguez
**Instrument:**		**Injection date:**	2025-01-09 21:11:10-08:00
**Inj. volume:**		**Location:**	P1-F-04
**Acq. method:**	10-75OVER20_PEPTIDES-C3_200MZ.M	**Type:**	Sample
**Processing method:**	*Deconvolution Test 2.pmx	**Sample amount:**	
**Manually modified:**	Manual Integration		

Data Analysis Method: **Deconvolution Test 2.pmx**
Path: **D:\CDSProjects\Walkup Submissions\Results\Chemstatioimports_MRR.rslt**
Method parameters are filtered - only a subset is displayed

2 Method Parameters

2.11 MS Spectral Deconvolution Parameters

**Run automatic deconvolution:**	Yes	**Use RT window:**	No	**TIC peak type:**	All peaks
**TIC peak threshold:**	Top (n) peaks	**Top (n) peaks:**	6	**Positive adduct:**	+H
**Negative adduct:**	-H	**Use m/z range:**	No	**Low molecular weight:**	4000
**High molecular weight:**	25000	**Maximum charge:**	40	**Minimum peaks in set:**	3
**Show unmatched peaks:**	No	**MW agreement (0.01%):**	5	**Absolute noise threshold:**	1000
**Relative abundance threshold (%):**	10	**MW algorithm:**	Curve Fit	**MW algorithm threshold (%):**	40
**Envelope threshold (%):**	50				

Method Audit Trail

Method audit trail is not printed

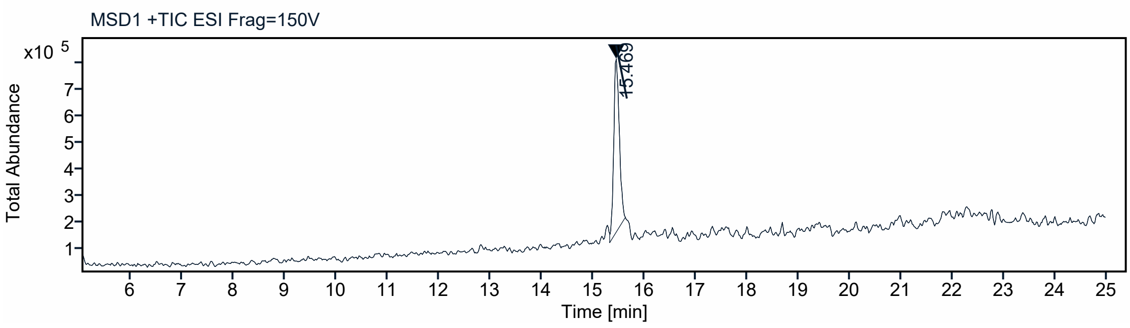

Single Injection Report

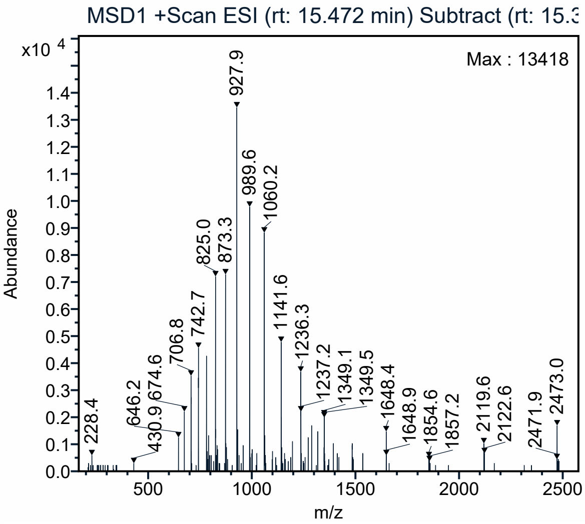

Deconvolution of peak at RT: 15.469

Signal: MSD1 +TIC ESI Frag=150V

Spectrum: MSD1 +Scan ESI (rt: 15.472 min) Subtract (rt: 15.347 min)

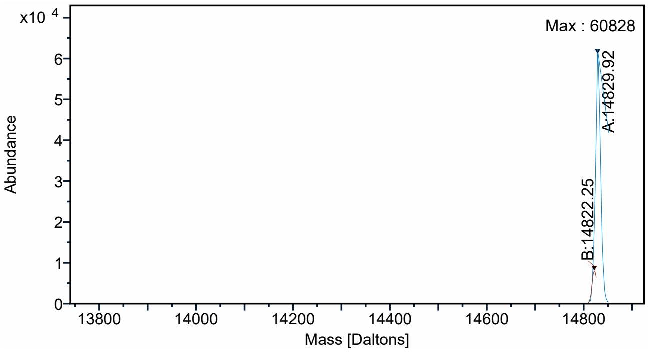

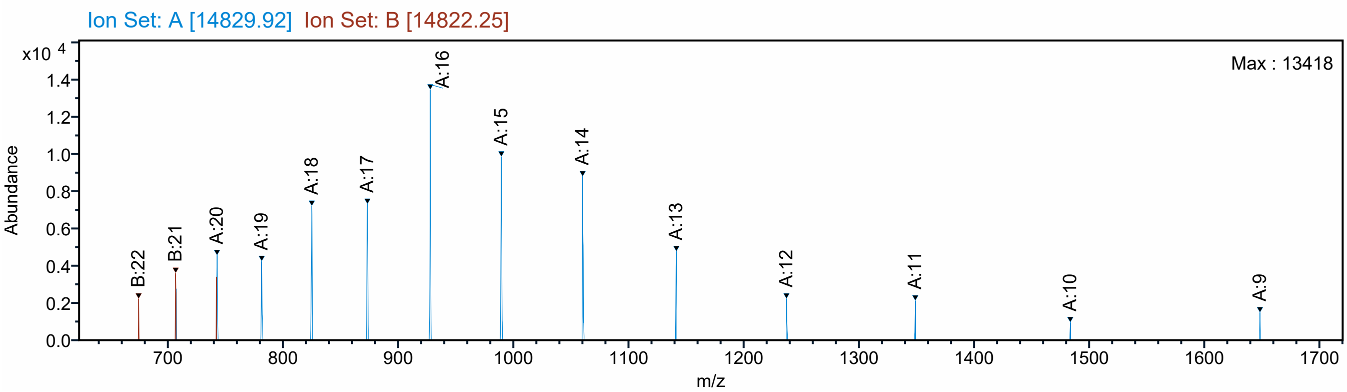

Component	Mass	Absolute Abundance	Relative Abundance (%)	Relative Quantitation (%)
A	14829.92	60828	100.00	88.42
B	14822.25	7968	13.10	11.58

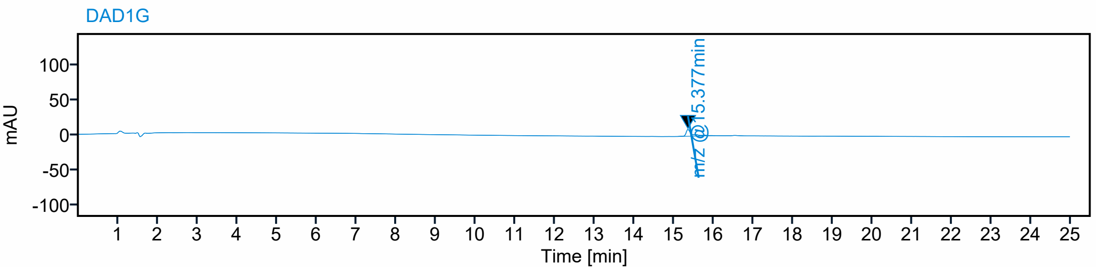

Signal: DAD1G

Name	RT [min]	RF	Area	Peak Area Percent	Group
	15.377		102.8639	100.00	unmodified protein 100%

Signal: MSD1 +TIC ESI Frag=150V

Name	RT [min]	RF	Area	Peak Area Percent	Group
	15.469		4938292.5188	100.00	unmodified protein 100%

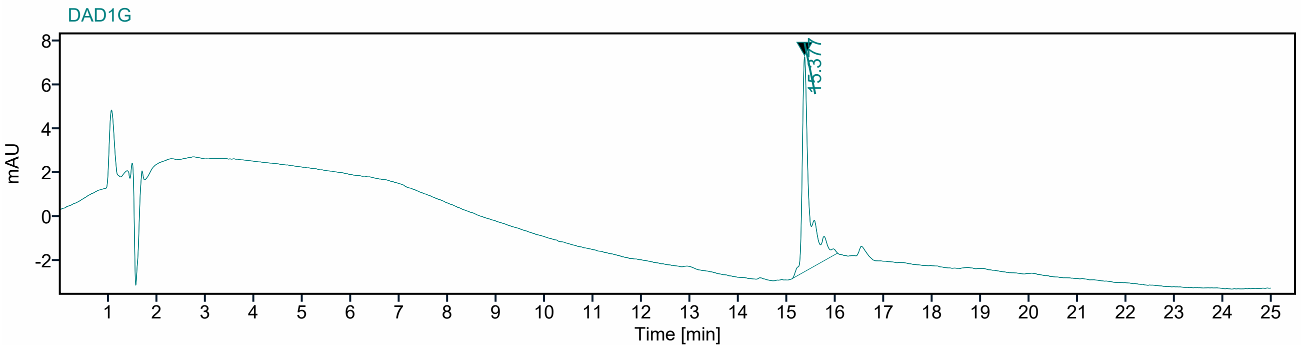
DAD1G
8
6
4
2
0
-2
mAU
15.377
1 2 3 4 5 6 7 8 9 10 11 12 13 14 15 16 17 18 19 20 21 22 23 24 25
Time [min]

Single Injection Report

**Data file:**	090125-10-75OVER20_PEPTIDES-C3_200M-30673_008.D		
**Sequence Name:**	Chemstatioimports_MRR	**Project Name:**	Walkup Submissions
**Sample name:**	Brd4BD2_WT_mr116_9Jan25	**Operator:**	Maria Rodriguez
**Instrument:**		**Injection date:**	2025-01-09 21:41:01-08:00
**Inj. volume:**		**Location:**	P1-F-05
**Acq. method:**	10-75OVER20_PEPTIDES-C3_200MZ.M	**Type:**	Sample
**Processing method:**	*Deconvolution Test 2.pmx	**Sample amount:**	
**Manually modified:**	Manual Integration		

Data Analysis Method: **Deconvolution Test 2.pmx**
Path: **D:\CDSProjects\Walkup Submissions\Results\Chemstatioimports_MRR.rslt**
Method parameters are filtered - only a subset is displayed

2 Method Parameters

2.11 MS Spectral Deconvolution Parameters

**Run automatic deconvolution:**	Yes	**Use RT window:**	No	**TIC peak type:**	All peaks
**TIC peak threshold:**	Top (n) peaks	**Top (n) peaks:**	6	**Positive adduct:**	+H
**Negative adduct:**	-H	**Use m/z range:**	No	**Low molecular weight:**	4000
**High molecular weight:**	25000	**Maximum charge:**	40	**Minimum peaks in set:**	3
**Show unmatched peaks:**	No	**MW agreement (0.01%):**	5	**Absolute noise threshold:**	1000
**Relative abundance threshold (%):**	10	**MW algorithm:**	Curve Fit	**MW algorithm threshold (%):**	40
**Envelope threshold (%):**	50				

Method Audit Trail

Method audit trail is not printed

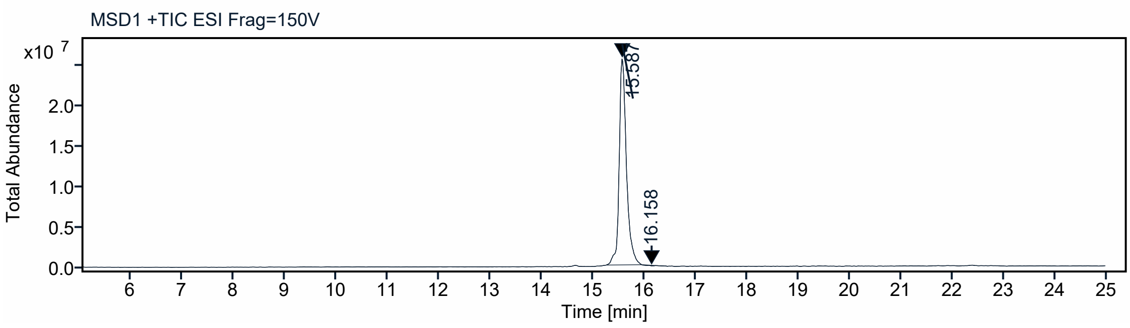

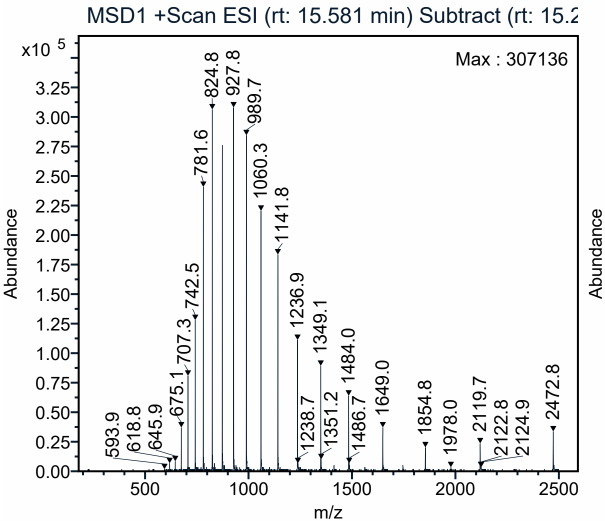

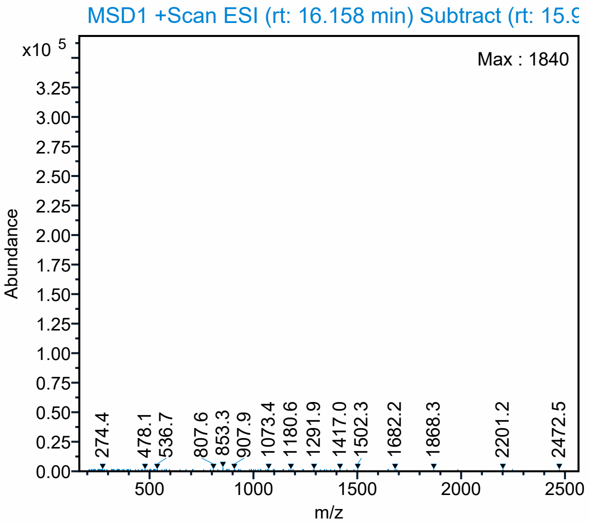

Deconvolution of peak at RT: 15.587

Signal: MSD1 +TIC ESI Frag=150V

Spectrum: MSD1 +Scan ESI (rt: 15.581 min) Subtract (rt: 15.285 min)

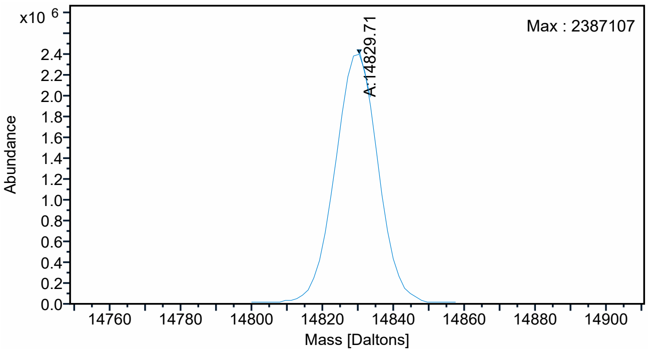

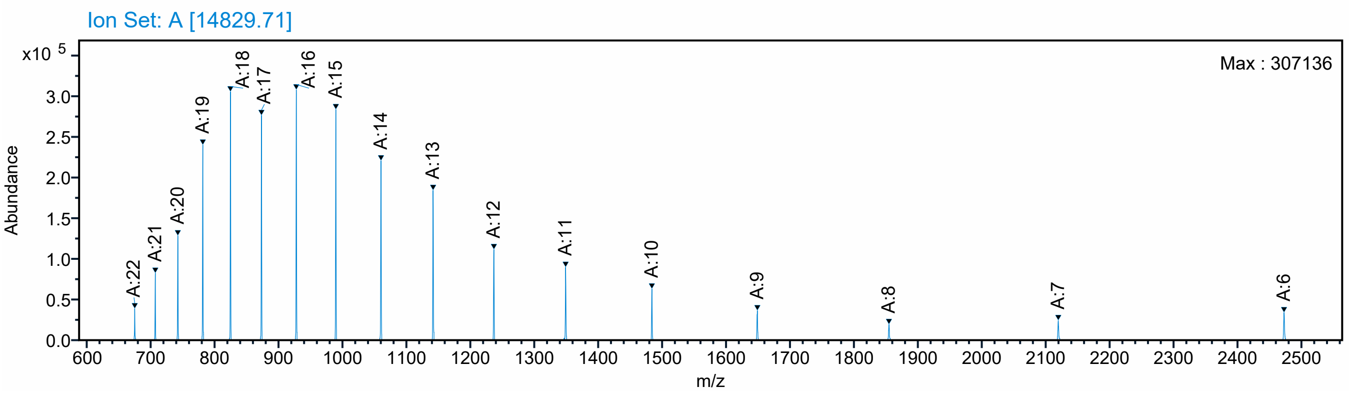

Component	Mass	Absolute Abundance	Relative Abundance (%)	Relative Quantitation (%)
A	14829.71	2387107	100.00	100.00

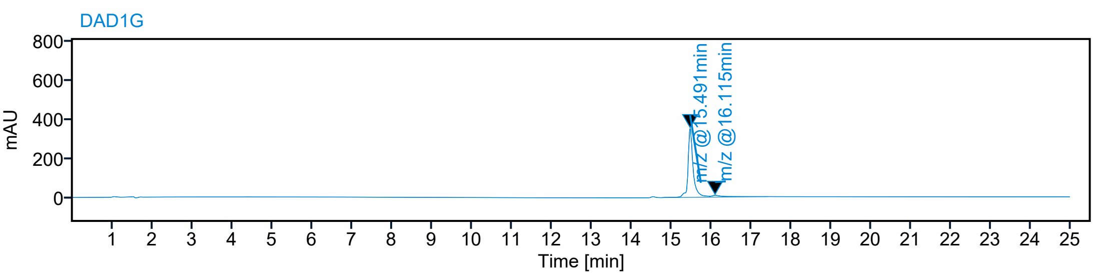

Signal: DAD1G

Name	RT [min]	RF	Area	Peak Area Percent	Group
	15.491		3122.2409	93.34	unmodified protein 100%
	~~16.115~~		~~222.7700~~	~~6.66~~	no protein detected in MS

Signal: MSD1 +TIC ESI Frag=150V

Name	RT [min]	RF	Area	Peak Area Percent	Group
	15.587		249218444.7878	99.70	unmodified protein 100%
	~~16.158~~		~~761955.9731~~	~~0.30~~	no protein detected in MS

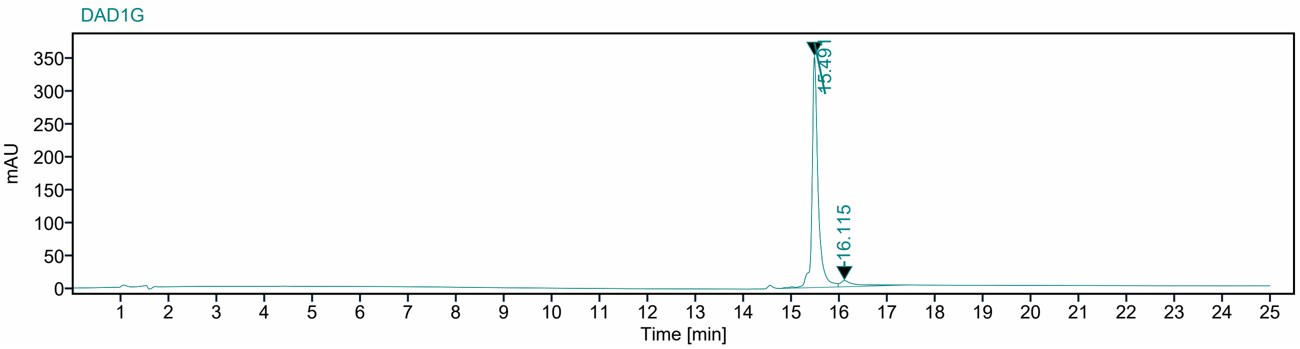
DAD1G
mAU
350
300
250
200
150
100
50
0
15.491
16.115
1
2
3
4
5
6
7
8
9
10
11
12
13
14
15
16
17
18
19
20
21
22
23
24
25
Time [min]

Single Injection Report

**Data file:**	090125-10-75OVER20_PEPTIDES-C3_200M-30674_009.D		
**Sequence Name:**	Chemstatioimports_MRR	**Project Name:**	Walkup Submissions
**Sample name:**	Brd4BD2_WT_mr108_9Jan25	**Operator:**	Maria Rodriguez
**Instrument:**		**Injection date:**	2025-01-09 22:10:51-08:00
**Inj. volume:**		**Location:**	P1-F-06
**Acq. method:**	10-75OVER20_PEPTIDES-C3_200MZ.M	**Type:**	Sample
**Processing method:**	*Deconvolution Test 2.pmx	**Sample amount:**	
**Manually modified:**	Manual Integration		

Data Analysis Method: **Deconvolution Test 2.pmx**
Path: **D:\CDSProjects\Walkup Submissions\Results\Chemstatioimports_MRR.rslt**
Method parameters are filtered - only a subset is displayed

2 Method Parameters

2.11 MS Spectral Deconvolution Parameters

**Run automatic deconvolution:**	Yes	**Use RT window:**	No	**TIC peak type:**	All peaks
**TIC peak threshold:**	Top (n) peaks	**Top (n) peaks:**	6	**Positive adduct:**	+H
**Negative adduct:**	-H	**Use m/z range:**	No	**Low molecular weight:**	4000
**High molecular weight:**	25000	**Maximum charge:**	40	**Minimum peaks in set:**	3
**Show unmatched peaks:**	No	**MW agreement (0.01%):**	5	**Absolute noise threshold:**	1000
**Relative abundance threshold (%):**	10	**MW algorithm:**	Curve Fit	**MW algorithm threshold (%):**	40
**Envelope threshold (%):**	50				

Method Audit Trail

Method audit trail is not printed

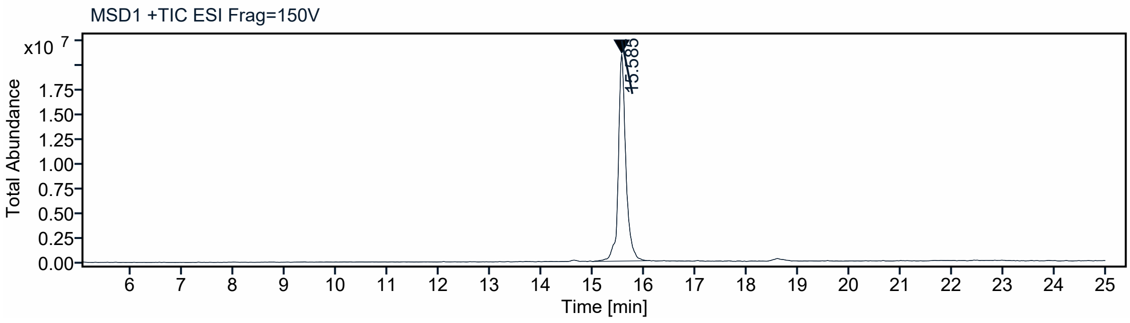

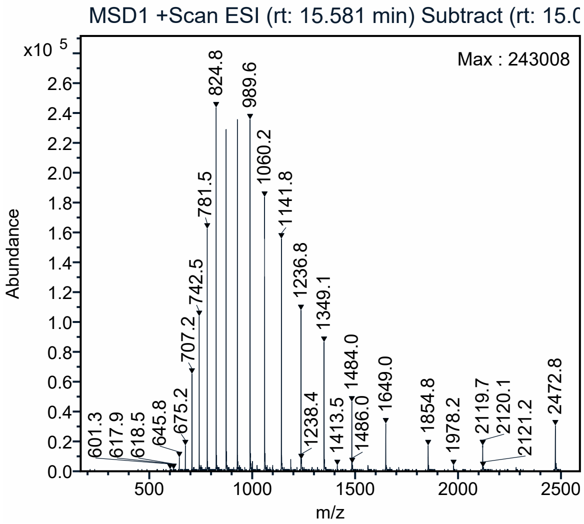

Deconvolution of peak at RT: 15.585

Signal: MSD1 +TIC ESI Frag=150V

Spectrum: MSD1 +Scan ESI (rt: 15.581 min) Subtract (rt: 15.035 min)

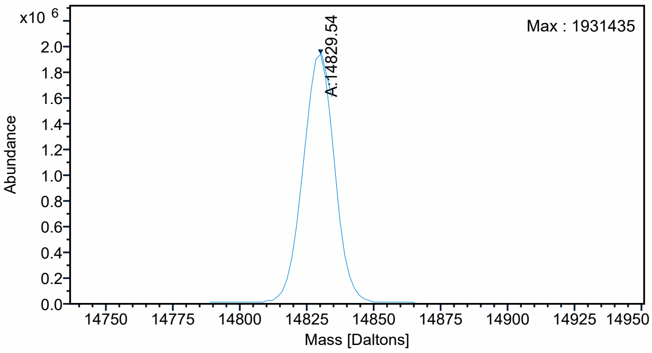

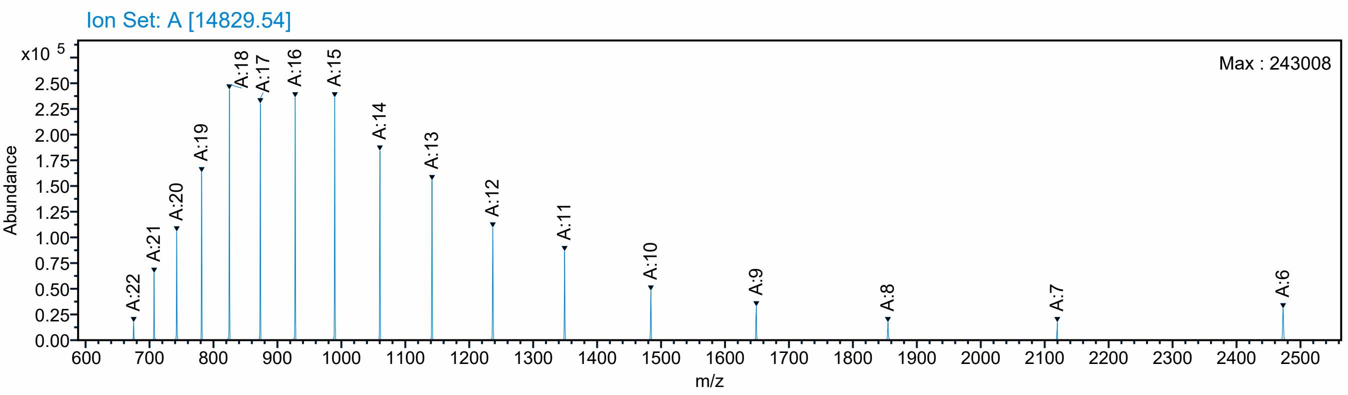

Component	Mass	Absolute Abundance	Relative Abundance (%)	Relative Quantitation (%)
A	14829.54	1931435	100.00	100.00

Single Injection Report

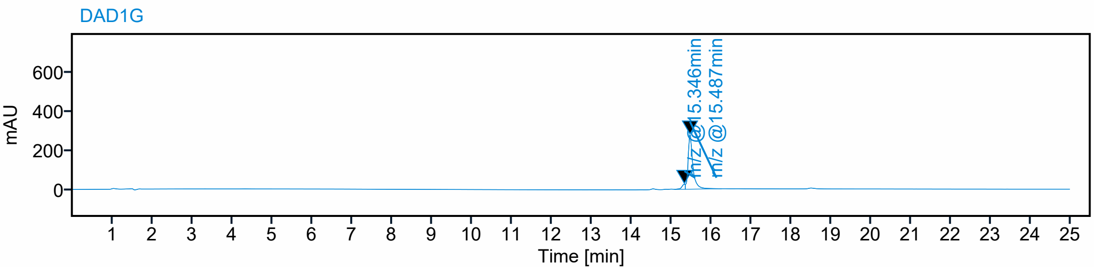

Signal: DAD1G

Name	RT [min]	RF	Area	Peak Area Percent	
no protein detected	~~15.346~~		~~151.0598~~	~~6.09~~	
	15.487		2330.1589	93.91	unmodified protein 100%

Signal: MSD1 +TIC ESI Frag=150V

Name	RT [min]	RF	Area	Peak Area Percent	Group
	15.585		213022123.1680	100.00	unmodified protein 100%

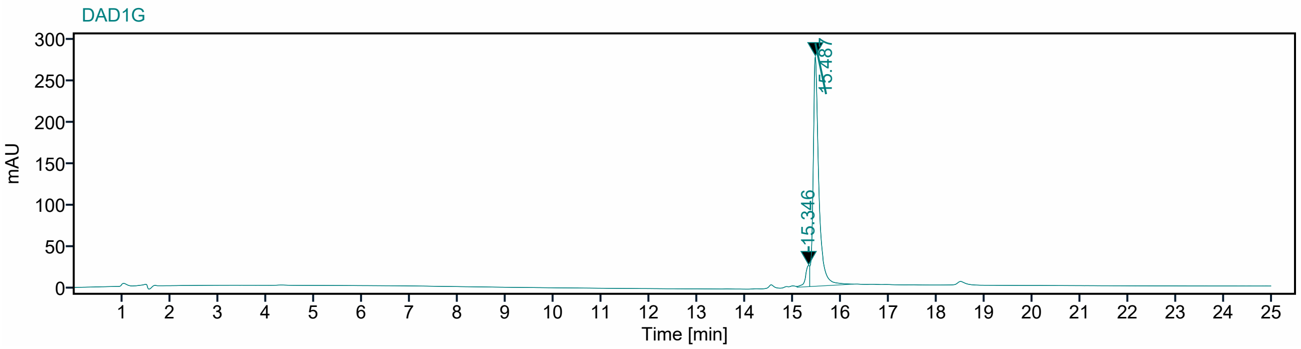
DAD1G
300
250
200
150
100
50
0
mAU
15.487
15.346
1
2
3
4
5
6
7
8
9
10
11
12
13
14
15
16
17
18
19
20
21
22
23
24
25
Time [min]

Single Injection Report

**Data file:**	090125-10-75OVER20_PEPTIDES-C3_200M-30675_010.D		
**Sequence Name:**	Chemstatioimports_MRR	**Project Name:**	Walkup Submissions
**Sample name:**	Brd4BD2_WT_mr109_9Jan25	**Operator:**	Maria Rodriguez
**Instrument:**		**Injection date:**	2025-01-09 22:40:42-08:00
**Inj. volume:**		**Location:**	P1-F-07
**Acq. method:**	10-75OVER20_PEPTIDES-C3_200MZ.M	**Type:**	Sample
**Processing method:**	*Deconvolution Test 2.pmx	**Sample amount:**	
**Manually modified:**	Manual Integration		

Data Analysis Method: Deconvolution Test 2.pmx
Path: D:\CDSProjects\Walkup Submissions\Results\Chemstatioimports_MRR.rslt
Method parameters are filtered - only a subset is displayed

2 Method Parameters

2.11 MS Spectral Deconvolution Parameters

**Run automatic deconvolution:**	Yes	**Use RT window:**	No	**TIC peak type:**	All peaks
**TIC peak threshold:**	Top (n) peaks	**Top (n) peaks:**	6	**Positive adduct:**	+H
**Negative adduct:**	-H	**Use m/z range:**	No	**Low molecular weight:**	4000
**High molecular weight:**	25000	**Maximum charge:**	40	**Minimum peaks in set:**	3
**Show unmatched peaks:**	No	**MW agreement (0.01%):**	5	**Absolute noise threshold:**	1000
**Relative abundance threshold (%):**	10	**MW algorithm:**	Curve Fit	**MW algorithm threshold (%):**	40
**Envelope threshold (%):**	50				

Method Audit Trail

Method audit trail is not printed

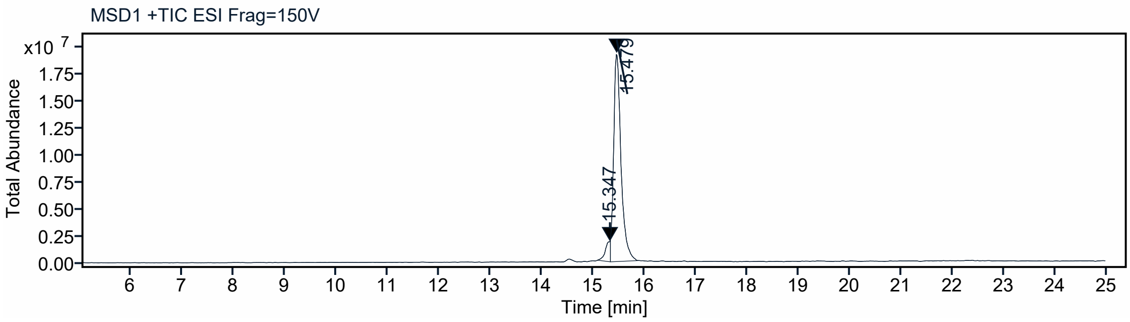

Single Injection Report

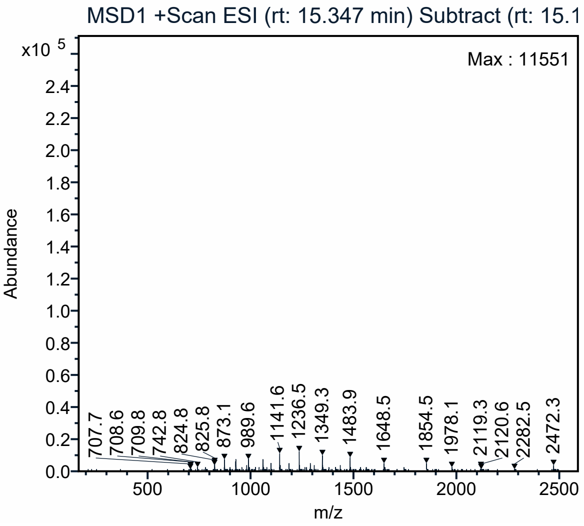

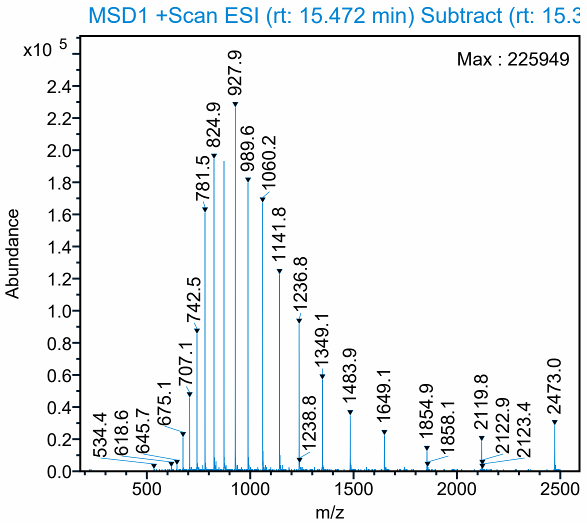

Deconvolution of peak at RT: 15.347

Signal: MSD1 +TIC ESI Frag=150V

Spectrum: MSD1 +Scan ESI (rt: 15.347 min) Subtract (rt: 15.113 min)

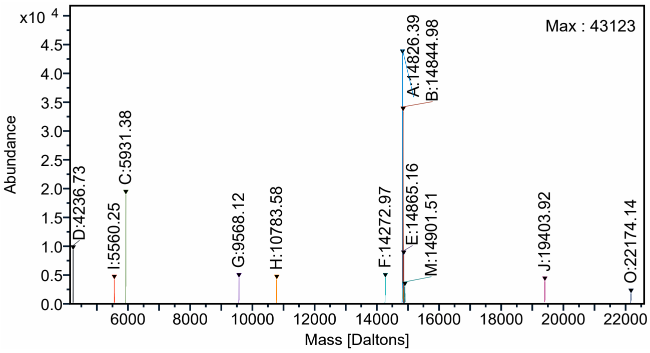

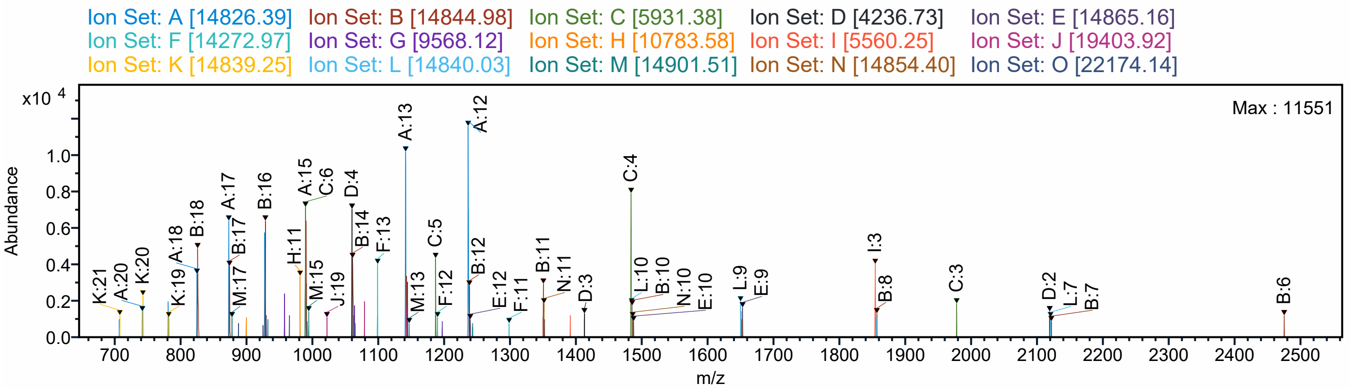

Component	Mass	Absolute Abundance	Relative Abundance (%)	Relative Quantitation (%)
A	14826.39	43123	100.00	29.34
B	14844.98	33329	77.29	22.67
C	5931.38	18901	43.83	12.86

D	4236.73	9039	20.96	6.15
E	14865.16	8388	19.45	5.71
F	14272.97	4481	10.39	3.05
G	9568.12	4402	10.21	2.99
H	10783.58	4187	9.71	2.85
I	5560.25	4042	9.37	2.75
J	19403.92	3853	8.93	2.62
K	14839.25	3659	8.49	2.49
L	14840.03	3010	6.98	2.05
M	14901.51	2721	6.31	1.85
N	14854.40	2309	5.35	1.57
O	22174.14	1553	3.60	1.06

Deconvolution of peak at RT: 15.479

Signal: MSD1 +TIC ESI Frag=150V

Spectrum: MSD1 +Scan ESI (rt: 15.472 min) Subtract (rt: 15.363 min)

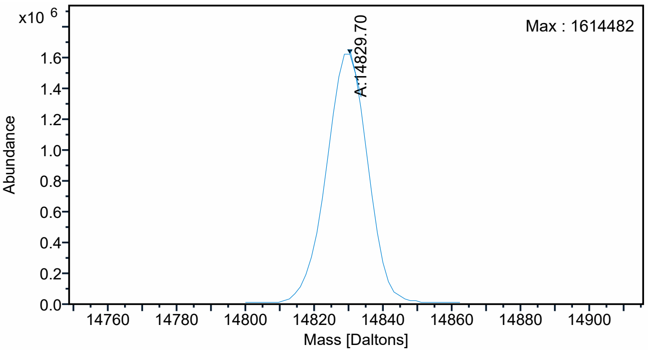

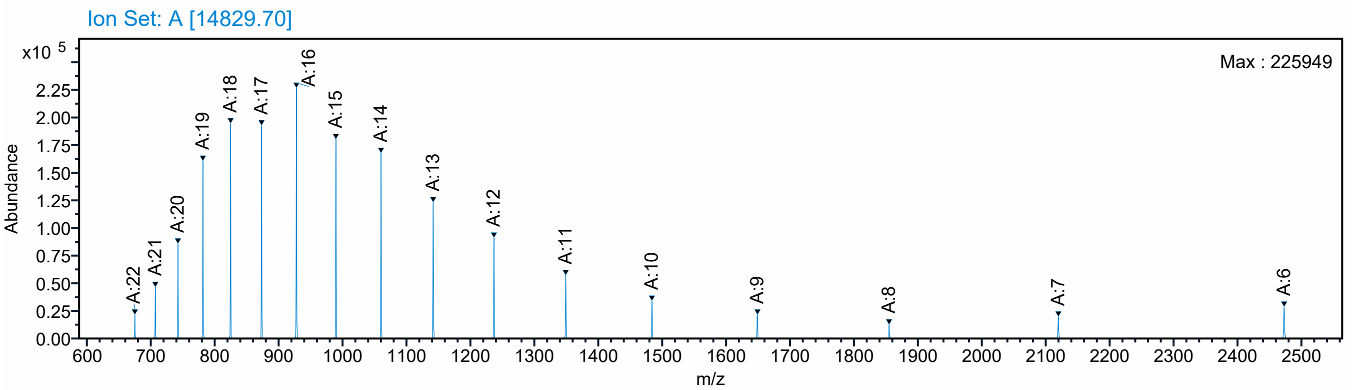

Component	Mass	Absolute Abundance	Relative Abundance (%)	Relative Quantitation (%)
A	14829.70	1614482	100.00	100.00

Single Injection Report

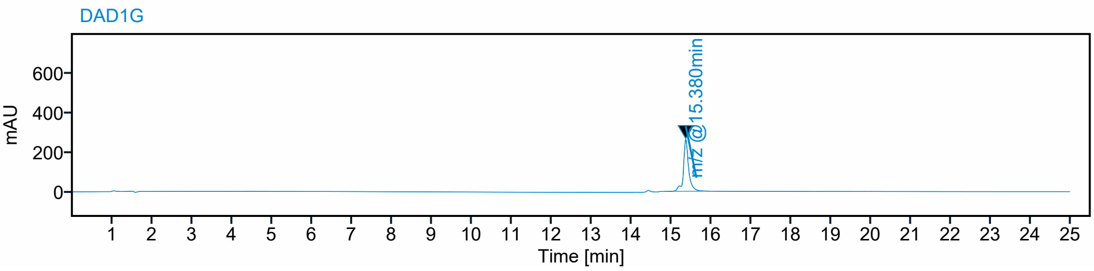

Signal: DAD1G

Name	RT [min]	RF	Area	Peak Area Percent	Group
	15.380		2340.9346	100.00	

unmodified protein 100%

Signal: MSD1 +TIC ESI Frag=150V

Name	RT [min]	RF	Area	Peak Area Percent	Group
	15.347		12659603.6263	6.22	
	15.479		190739158.8885	93.78	

unmodified protein 100%

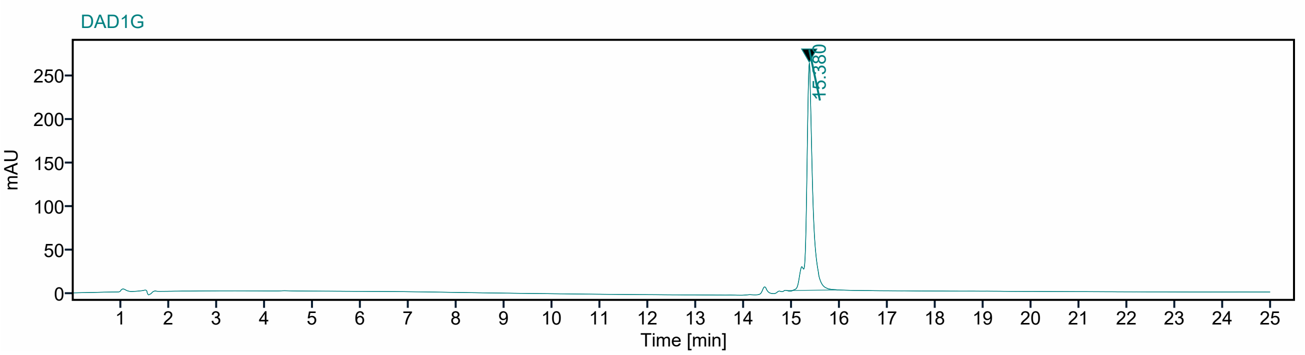
DAD1G
250
200
150
100
50
0
mAU
15.380
1
2
3
4
5
6
7
8
9
10
11
12
13
14
15
16
17
18
19
20
21
22
23
24
25
Time [min]

Single Injection Report

**Data file:**	090125-10-75OVER20_PEPTIDES-C3_200M-30676_011.D		
**Sequence Name:**	Chemstatioimports_MRR	**Project Name:**	Walkup Submissions
**Sample name:**	Brd4BD2_WT_mr115_9Jan25	**Operator:**	Maria Rodriguez
**Instrument:**		**Injection date:**	2025-01-09 23:10:32-08:00
**Inj. volume:**		**Location:**	P1-F-08
**Acq. method:**	10-75OVER20_PEPTIDES-C3_200MZ.M	**Type:**	Sample
**Processing method:**	*Deconvolution Test 2.pmx	**Sample amount:**	
**Manually modified:**	Manual Integration		

Data Analysis Method: Deconvolution Test 2.pmx
Path: D:\CDSProjects\Walkup Submissions\Results\Chemstatioimports_MRR.rslt
Method parameters are filtered - only a subset is displayed

2 Method Parameters

2.11 MS Spectral Deconvolution Parameters

**Run automatic deconvolution:**	Yes	**Use RT window:**	No	**TIC peak type:**	All peaks
**TIC peak threshold:**	Top (n) peaks	**Top (n) peaks:**	6	**Positive adduct:**	+H
**Negative adduct:**	-H	**Use m/z range:**	No	**Low molecular weight:**	4000
**High molecular weight:**	25000	**Maximum charge:**	40	**Minimum peaks in set:**	3
**Show unmatched peaks:**	No	**MW agreement (0.01%):**	5	**Absolute noise threshold:**	1000
**Relative abundance threshold (%):**	10	**MW algorithm:**	Curve Fit	**MW algorithm threshold (%):**	40
**Envelope threshold (%):**	50				

Method Audit Trail

Method audit trail is not printed

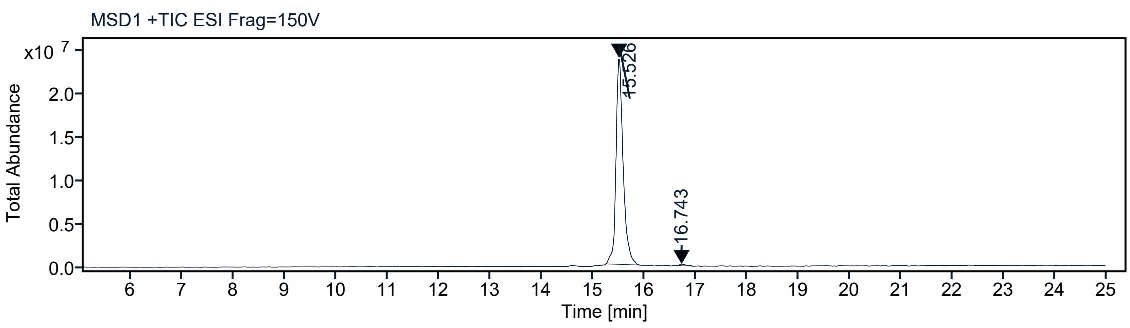

Single Injection Report

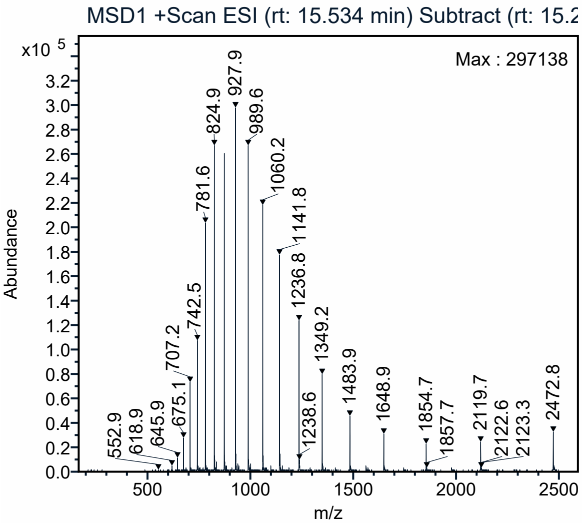

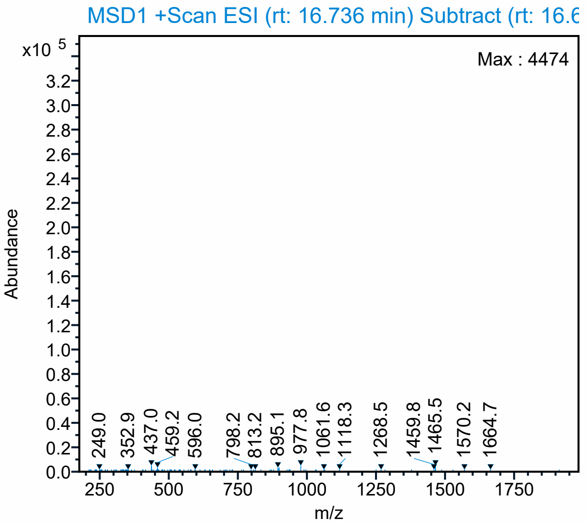

Deconvolution of peak at RT: 15.526

Signal: MSD1 +TIC ESI Frag=150V

Spectrum: MSD1 +Scan ESI (rt: 15.534 min) Subtract (rt: 15.285 min)

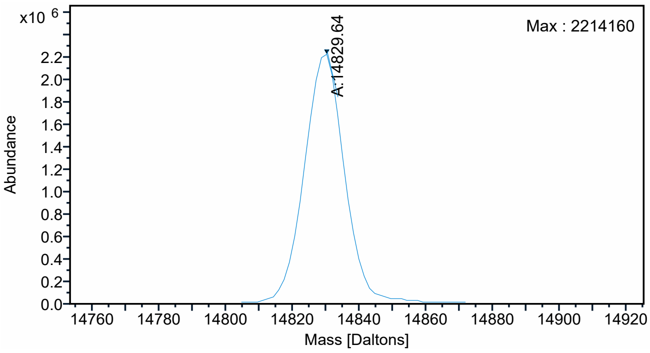

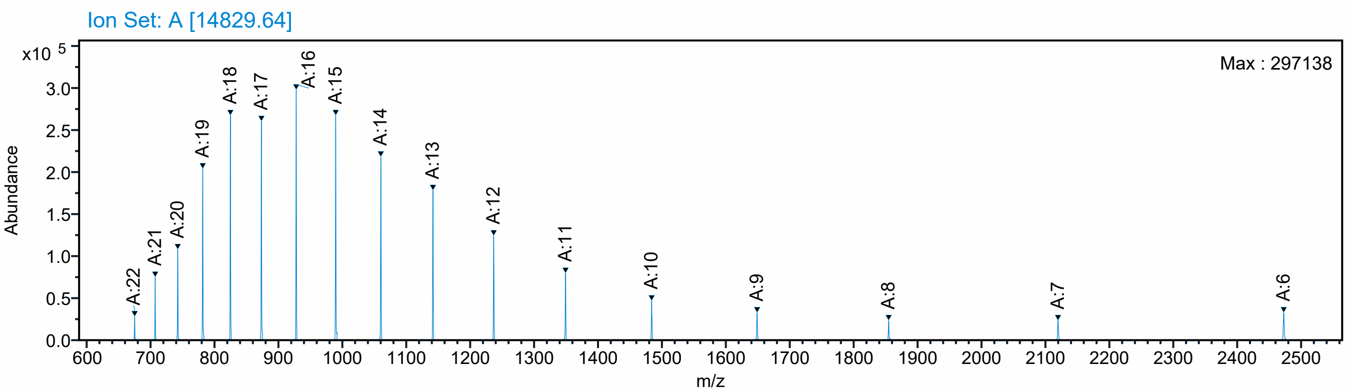

Component	Mass	Absolute Abundance	Relative Abundance (%)	Relative Quantitation (%)
A	14829.64	2214160	100.00	100.00

Single Injection Report

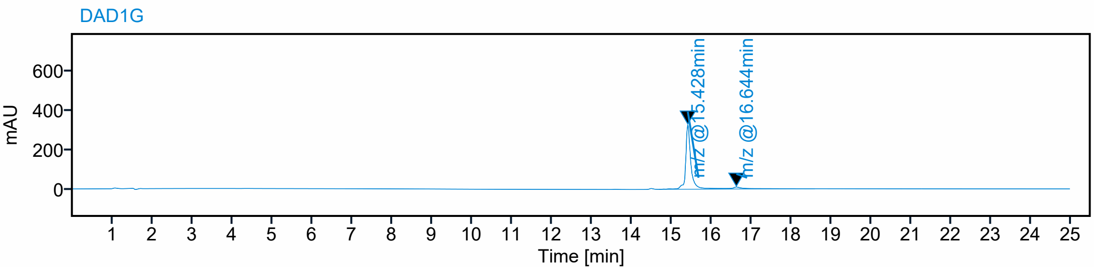

Signal: DAD1G

Name	RT [min]	RF	Area	Peak Area Percent	Group
	15.428		2979.0890	91.57	
	~~16.644~~		~~274.0832~~	~~8.43~~	

Signal: MSD1 +TIC ESI Frag=150V

Name	RT [min]	RF	Area	Peak Area Percent	Group
	15.526		228231699.2469	99.27	
	~~16.743~~		~~1688781.5187~~	~~0.73~~	

Single Injection Report

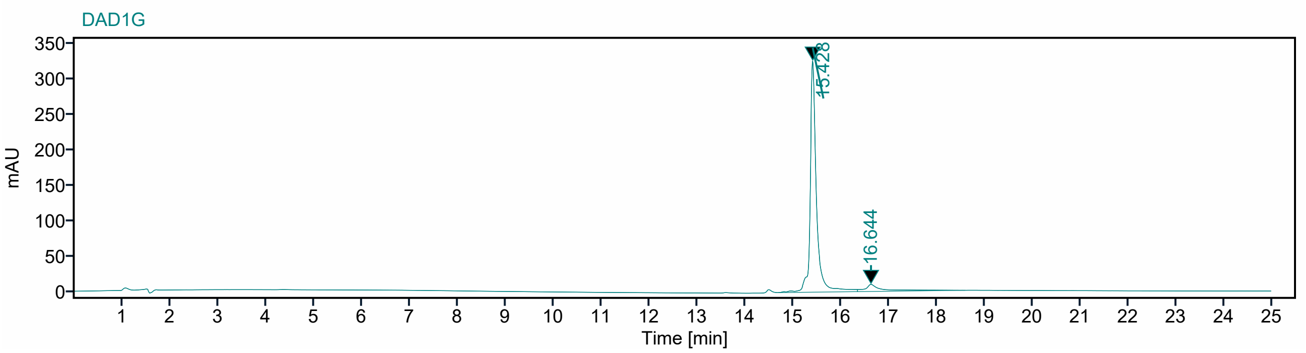

Single Injection Report

**Data file:**	090125-10-75OVER20_PEPTIDES-C3_200M-30677_012.D		
**Sequence Name:**	Chemstatioimports_MRR	**Project Name:**	Walkup Submissions
**Sample name:**	Brd4BD2_WT_mr111_9Jan25	**Operator:**	Maria Rodriguez
**Instrument:**		**Injection date:**	2025-01-09 23:40:23-08:00
**Inj. volume:**		**Location:**	P1-F-09
**Acq. method:**	10-75OVER20_PEPTIDES-C3_200MZ.M	**Type:**	Sample
**Processing method:**	*Deconvolution Test 2.pmx	**Sample amount:**	
**Manually modified:**	Manual Integration		

Data Analysis Method: **Deconvolution Test 2.pmx**
Path: **D:\CDSProjects\Walkup Submissions\Results\Chemstatioimports_MRR.rslt**
Method parameters are filtered - only a subset is displayed

2 Method Parameters

2.11 MS Spectral Deconvolution Parameters

**Run automatic deconvolution:**	Yes	**Use RT window:**	No	**TIC peak type:**	All peaks
**TIC peak threshold:**	Top (n) peaks	**Top (n) peaks:**	6	**Positive adduct:**	+H
**Negative adduct:**	-H	**Use m/z range:**	No	**Low molecular weight:**	4000
**High molecular weight:**	25000	**Maximum charge:**	40	**Minimum peaks in set:**	3
**Show unmatched peaks:**	No	**MW agreement (0.01%):**	5	**Absolute noise threshold:**	1000
**Relative abundance threshold (%):**	10	**MW algorithm:**	Curve Fit	**MW algorithm threshold (%):**	40
**Envelope threshold (%):**	50				

Method Audit Trail

Method audit trail is not printed

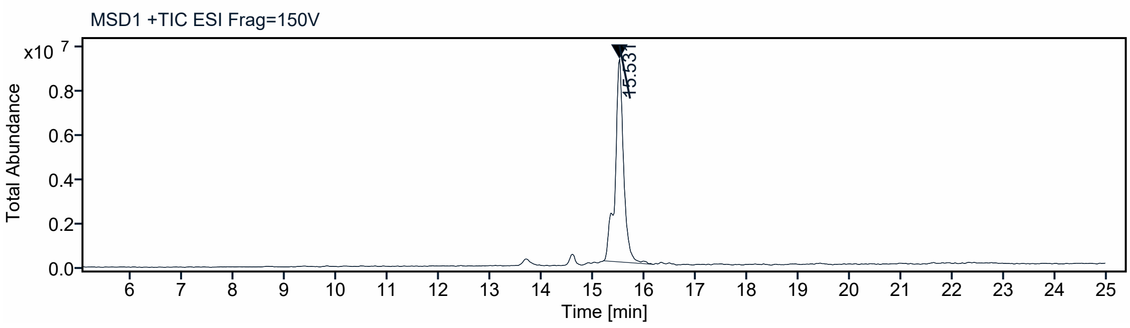

Single Injection Report

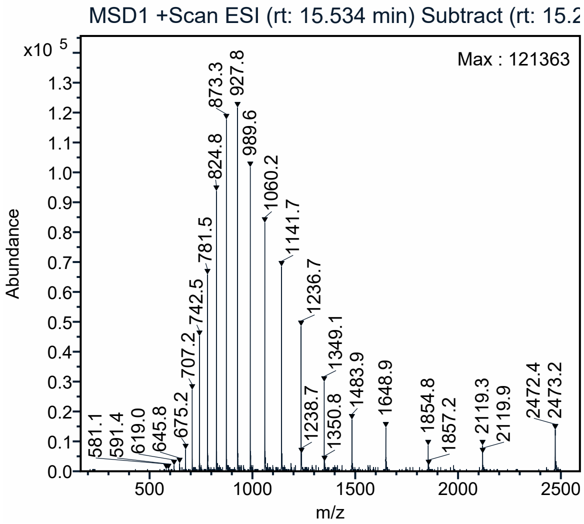

Deconvolution of peak at RT: 15.531

Signal: MSD1 +TIC ESI Frag=150V

Spectrum: MSD1 +Scan ESI (rt: 15.534 min) Subtract (rt: 15.222 min)

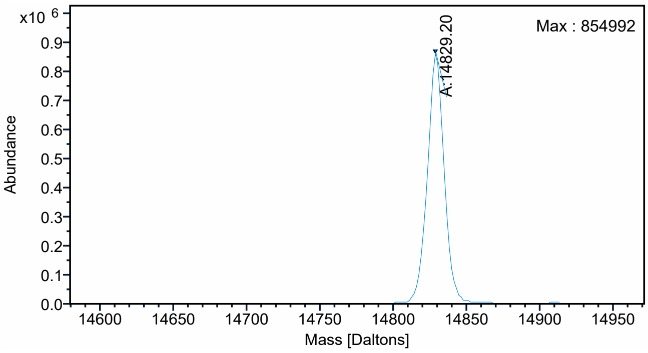

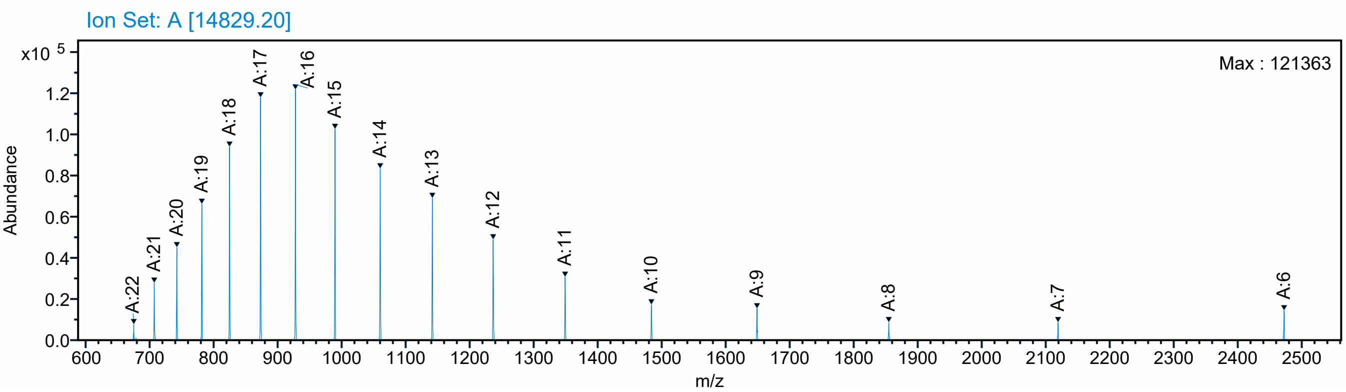

Component	Mass	Absolute Abundance	Relative Abundance (%)	Relative Quantitation (%)
A	14829.20	854992	100.00	100.00

Single Injection Report

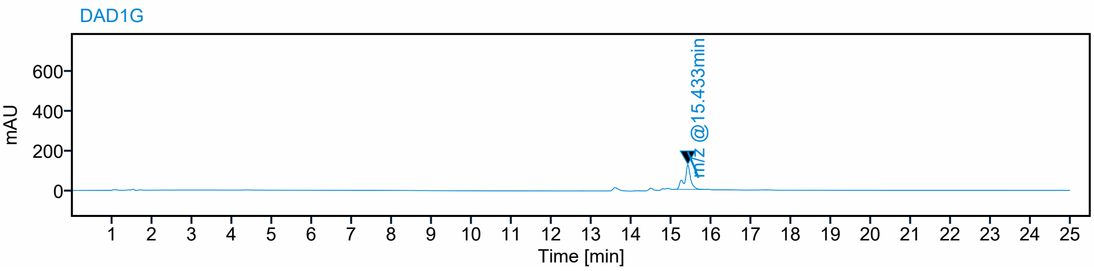

Signal: DAD1G

Name	RT [min]	RF	Area	Peak Area Percent	Group
	15.433		1310.2571	100.00	

Signal: MSD1 +TIC ESI Frag=150V

Name	RT [min]	RF	Area	Peak Area Percent	Group
	15.531		105313545.6303	100.00	

Single Injection Report

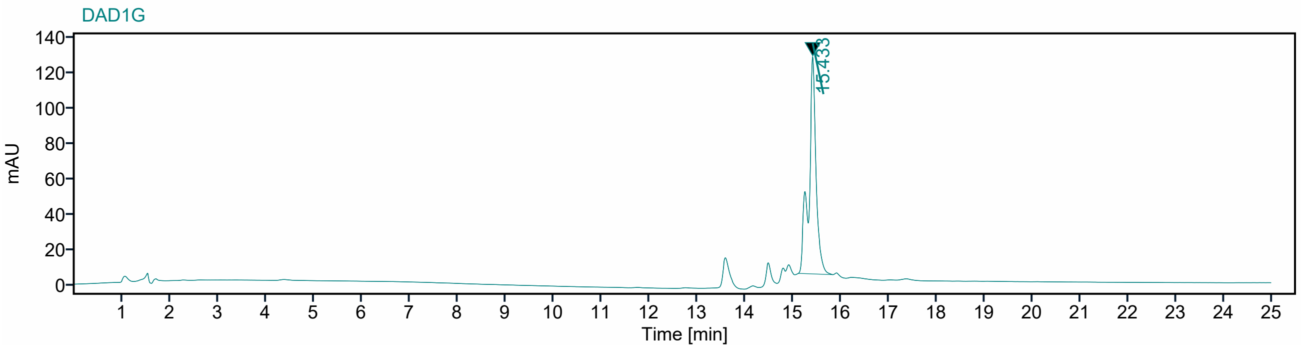

Single Injection Report

**Data file:**	090125-10-75OVER20_PEPTIDES-C3_200M-30678_013.D		
**Sequence Name:**	Chemstatioimports_MRR	**Project Name:**	Walkup Submissions
**Sample name:**	Brd4BD2_WT_mr118_9Jan25	**Operator:**	Maria Rodriguez
**Instrument:**		**Injection date:**	2025-01-10 00:11:00-08:00
**Inj. volume:**		**Location:**	P2-A-01
**Acq. method:**	10-75OVER20_PEPTIDES-C3_200MZ.M	**Type:**	Sample
**Processing method:**	*Deconvolution Test 2.pmx	**Sample amount:**	
**Manually modified:**	Manual Integration		

Data Analysis Method: Deconvolution Test 2.pmx
Path: D:\CDSProjects\Walkup Submissions\Results\Chemstatioimports_MRR.rslt
Method parameters are filtered - only a subset is displayed

2 Method Parameters

2.11 MS Spectral Deconvolution Parameters

**Run automatic deconvolution:**	Yes	**Use RT window:**	No	**TIC peak type:**	All peaks
**TIC peak threshold:**	Top (n) peaks	**Top (n) peaks:**	6	**Positive adduct:**	+H
**Negative adduct:**	-H	**Use m/z range:**	No	**Low molecular weight:**	4000
**High molecular weight:**	25000	**Maximum charge:**	40	**Minimum peaks in set:**	3
**Show unmatched peaks:**	No	**MW agreement (0.01%):**	5	**Absolute noise threshold:**	1000
**Relative abundance threshold (%):**	10	**MW algorithm:**	Curve Fit	**MW algorithm threshold (%):**	40
**Envelope threshold (%):**	50				

Method Audit Trail

Method audit trail is not printed

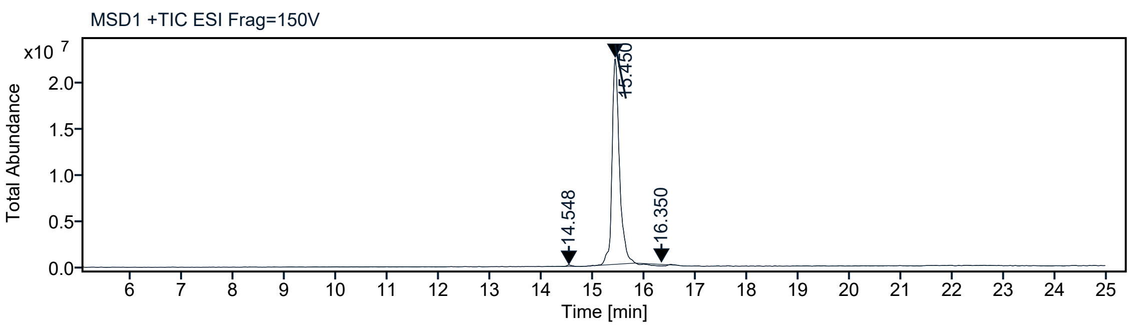

Single Injection Report

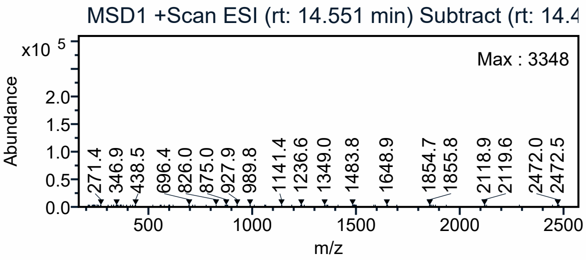

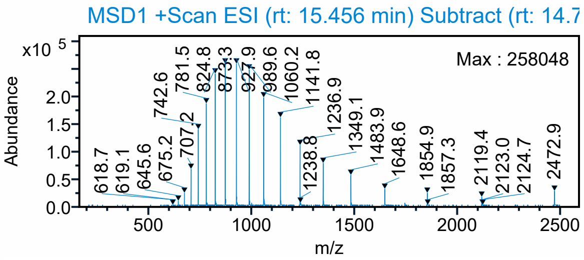

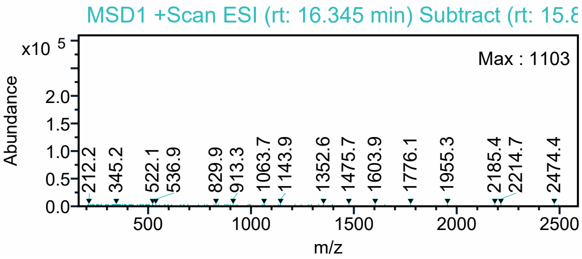

Deconvolution of peak at RT: 14.548

Signal: MSD1 +TIC ESI Frag=150V

Spectrum: MSD1 +Scan ESI (rt: 14.551 min) Subtract (rt: 14.411 min)

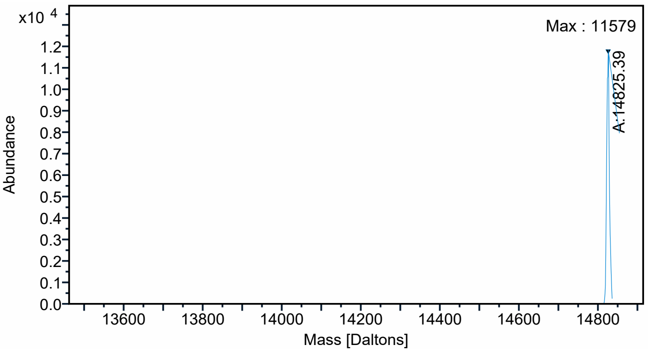

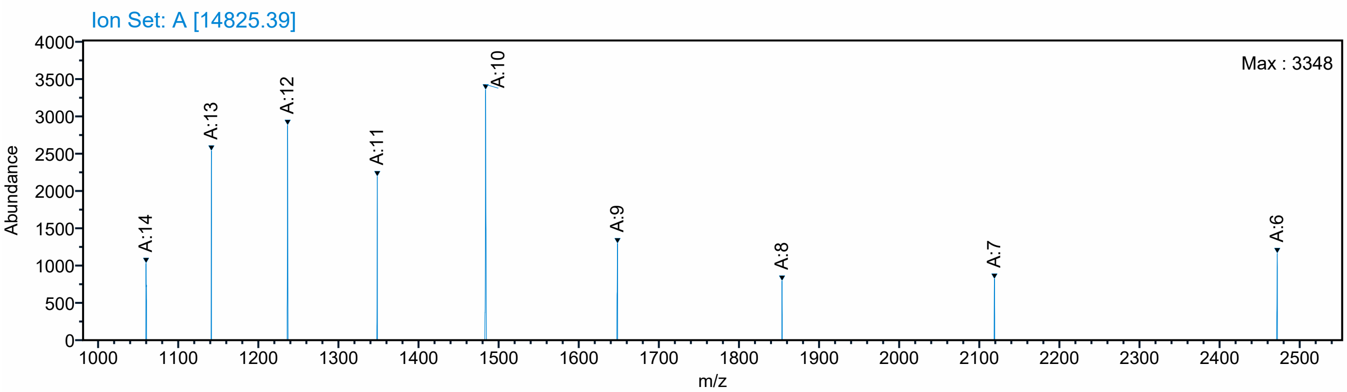

Component	Mass	Absolute Abundance	Relative Abundance (%)	Relative Quantitation (%)
A	14825.39	11579	100.00	100.00

Single Injection Report

Deconvolution of peak at RT: 15.450

Signal: MSD1 +TIC ESI Frag=150V

Spectrum: MSD1 +Scan ESI (rt: 15.456 min) Subtract (rt: 14.770 min)

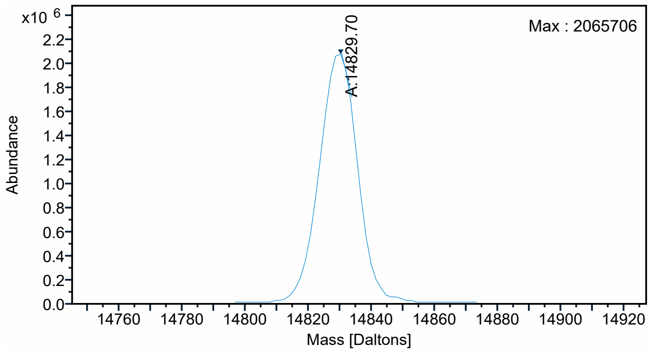

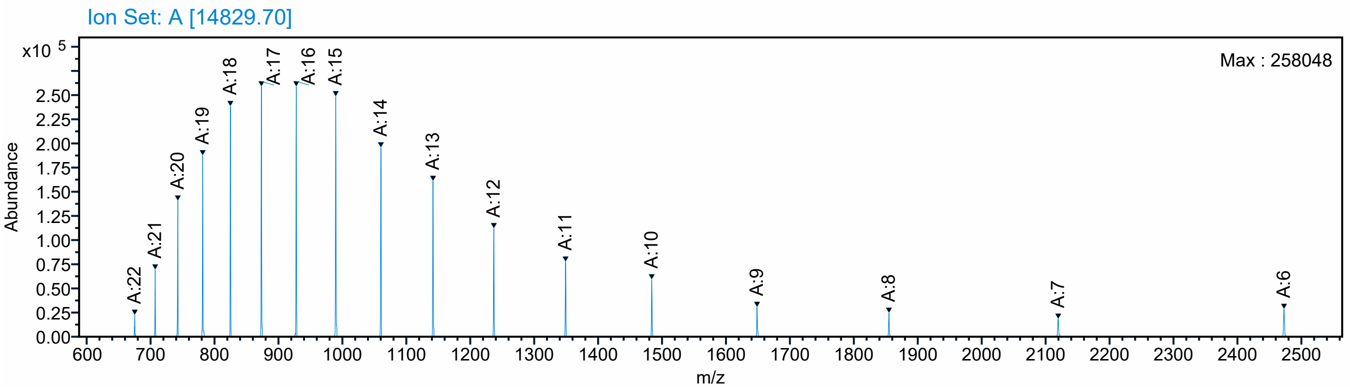

Component	Mass	Absolute Abundance	Relative Abundance (%)	Relative Quantitation (%)
A	14829.70	2065706	100.00	100.00

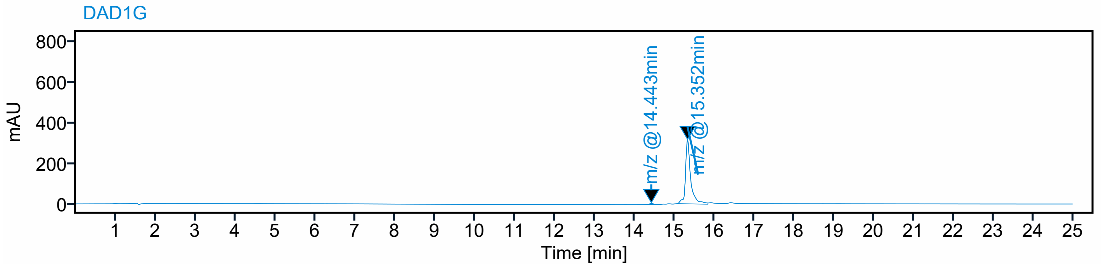

Single Injection Report

Signal: DAD1G

Name	RT [min]	RF	Area	Peak Area Percent	Group
	~~14.443~~		~~32.1432~~	~~1.12~~	
	15.352		2848.2125	98.88	

Signal: MSD1 +TIC ESI Frag=150V

Name	RT [min]	RF	Area	Peak Area Percent	Group
	~~14.548~~		~~1008953.2706~~	~~0.44~~	
	15.450		223858436.6474	97.57	
	~~16.350~~		~~4576006.9256~~	~~1.99~~	

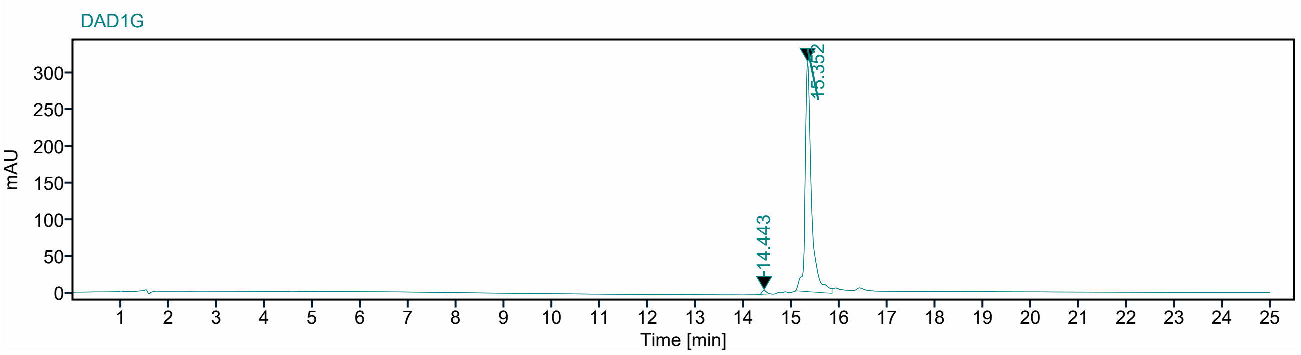

Single Injection Report

**Data file:**	090125-10-75OVER20_PEPTIDES-C3_200M-30679_014.D		
**Sequence Name:**	Chemstatioimports_MRR	**Project Name:**	Walkup Submissions
**Sample name:**	Brd4BD2_WT_mr117_9Jan25	**Operator:**	Maria Rodriguez
**Instrument:**		**Injection date:**	2025-01-10 00:40:51-08:00
**Inj. volume:**		**Location:**	P2-A-02
**Acq. method:**	10-75OVER20_PEPTIDES-C3_200MZ.M	**Type:**	Sample
**Processing method:**	*Deconvolution Test 2.pmx	**Sample amount:**	
**Manually modified:**	Manual Integration		

Data Analysis Method: Deconvolution Test 2.pmx
Path: D:\CDSProjects\Walkup Submissions\Results\Chemstatioimports_MRR.rslt
Method parameters are filtered - only a subset is displayed

2 Method Parameters

2.11 MS Spectral Deconvolution Parameters

**Run automatic deconvolution:**	Yes	**Use RT window:**	No	**TIC peak type:**	All peaks
**TIC peak threshold:**	Top (n) peaks	**Top (n) peaks:**	6	**Positive adduct:**	+H
**Negative adduct:**	-H	**Use m/z range:**	No	**Low molecular weight:**	4000
**High molecular weight:**	25000	**Maximum charge:**	40	**Minimum peaks in set:**	3
**Show unmatched peaks:**	No	**MW agreement (0.01%):**	5	**Absolute noise threshold:**	1000
**Relative abundance threshold (%):**	10	**MW algorithm:**	Curve Fit	**MW algorithm threshold (%):**	40
**Envelope threshold (%):**	50				

Method Audit Trail

Method audit trail is not printed

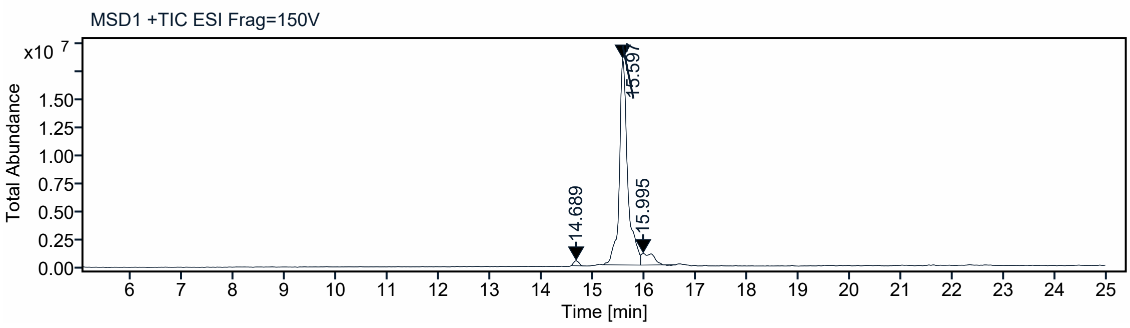

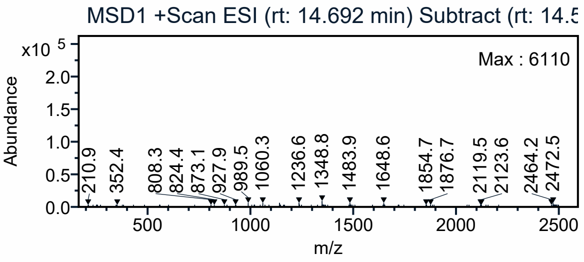

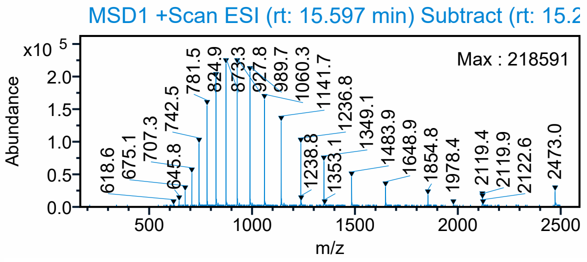

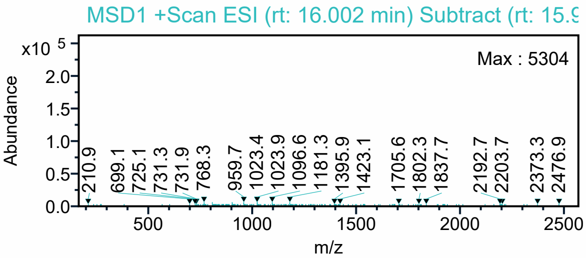

Deconvolution of peak at RT: 14.689

Signal: MSD1 +TIC ESI Frag=150V

Spectrum: MSD1 +Scan ESI (rt: 14.692 min) Subtract (rt: 14.598 min)

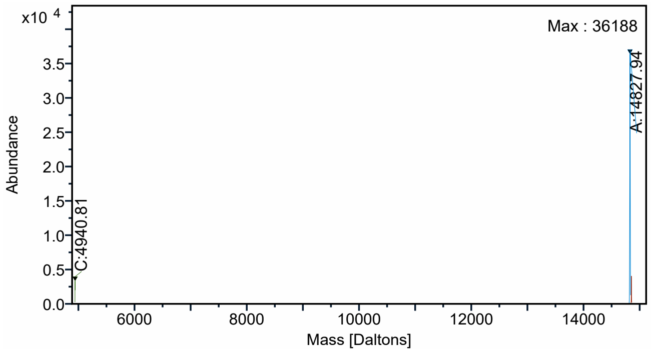

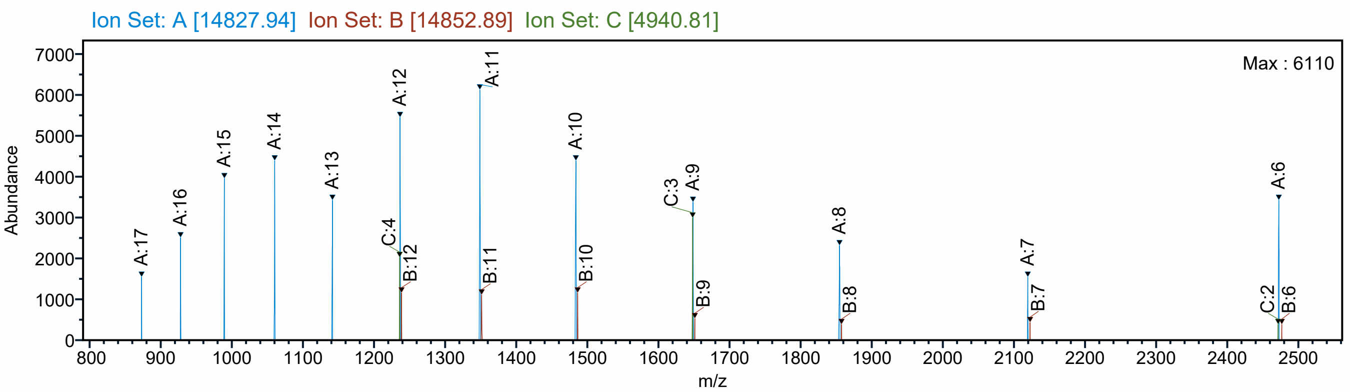

Component	Mass	Absolute Abundance	Relative Abundance (%)	Relative Quantitation (%)
A	14827.94	36188	100.00	83.61
B	14852.89	3876	10.71	8.96
C	4940.81	3218	8.89	7.43

Single Injection Report

Deconvolution of peak at RT: 15.597

Signal: MSD1 +TIC ESI Frag=150V

Spectrum: MSD1 +Scan ESI (rt: 15.597 min) Subtract (rt: 15.206 min)

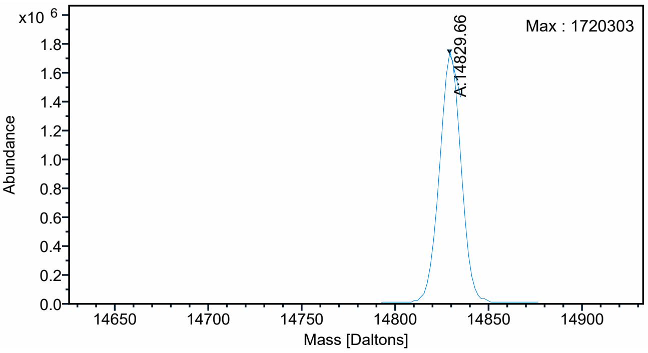

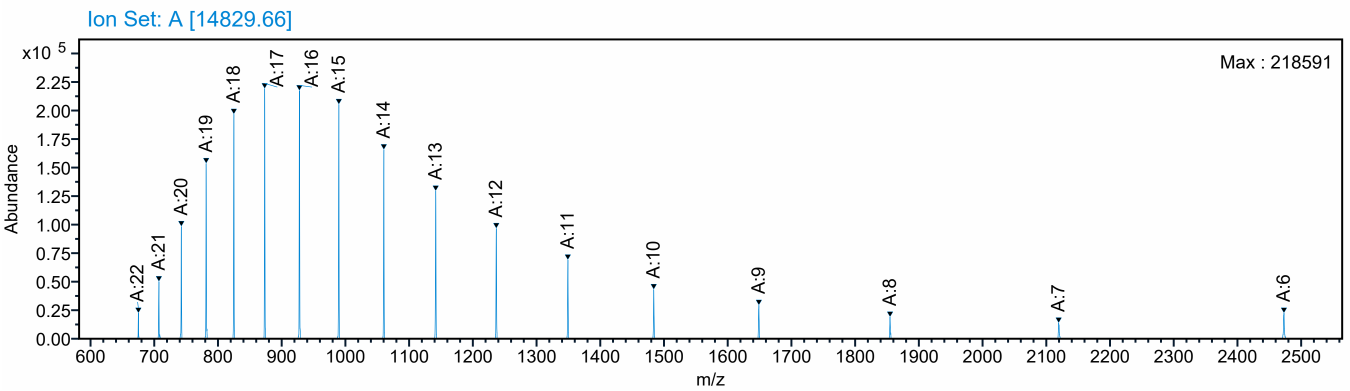

Component	Mass	Absolute Abundance	Relative Abundance (%)	Relative Quantitation (%)
A	14829.66	1720303	100.00	100.00

Deconvolution of peak at RT: 15.995

Signal: MSD1 +TIC ESI Frag=150V

Spectrum: MSD1 +Scan ESI (rt: 16.002 min) Subtract (rt: 15.955 min)

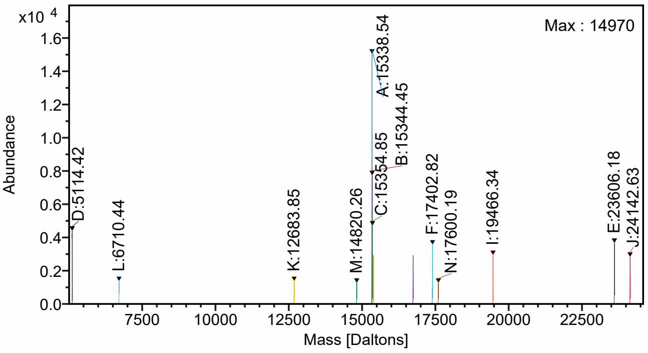

Single Injection Report

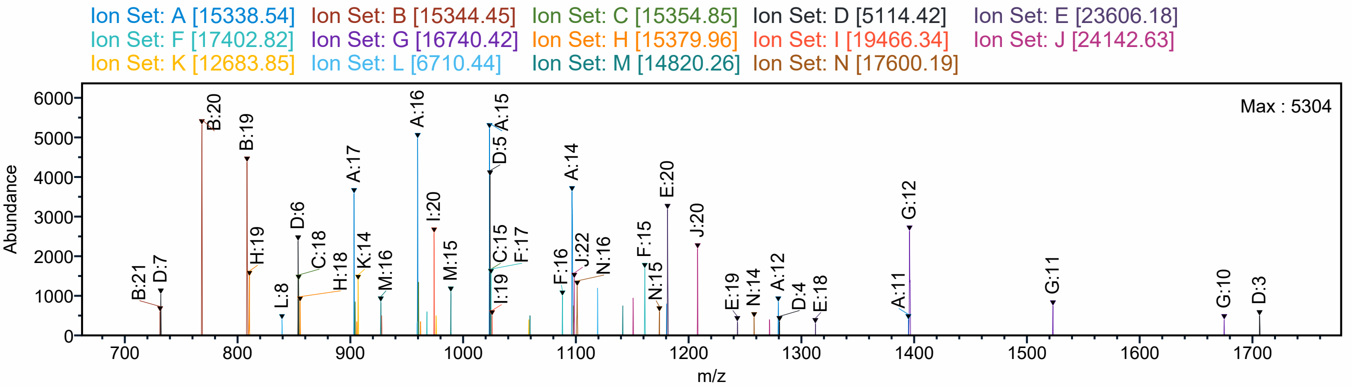

Component	Mass	Absolute Abundance	Relative Abundance (%)	Relative Quantitation (%)
A	15338.54	14970	100.00	27.09
B	15344.45	7653	51.12	13.85
C	15354.85	4656	31.10	8.43
D	5114.42	4367	29.17	7.90
E	23606.18	3621	24.19	6.55
F	17402.82	3504	23.41	6.34
G	16740.42	2917	19.49	5.28
H	15379.96	2869	19.16	5.19
I	19466.34	2843	18.99	5.14
J	24142.63	2731	18.24	4.94
K	12683.85	1344	8.98	2.43
L	6710.44	1338	8.94	2.42
M	14820.26	1234	8.24	2.23
N	17600.19	1217	8.13	2.20

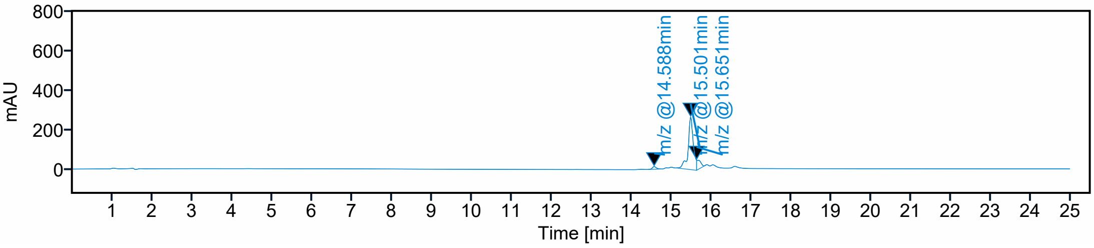

Signal: DAD1G

Name	RT [min]	RF	Area	Peak Area Percent	Group
	~~14.588~~		~~92.3981~~	~~3.28~~	no protein detected
	15.501		2393.6762	85.06	unmodified protein
	15.651		328.1223	11.66	modified protein (relative abundancy of modified ion in peak is 50%)

Signal: MSD1 +TIC ESI Frag=150V

Name	RT [min]	RF	Area	Peak Area Percent	Group
	~~14.689~~		~~3098171.0977~~	~~1.37~~	no protein detected
	15.597		206623743.1579	91.26	unmodified protein
	15.995		16700373.4224	7.38	modified protein (relative abundancy of modified ion in peak is 50%)

Single Injection Report

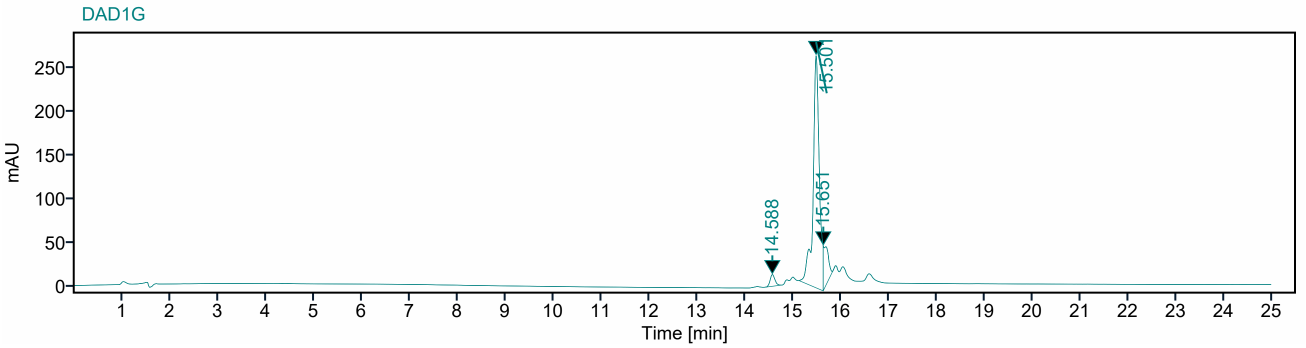

Single Injection Report

**Data file:**	090125-10-75OVER20_PEPTIDES-C3_200M-30680_015.D		
**Sequence Name:**	Chemstatioimports_MRR	**Project Name:**	Walkup Submissions
**Sample name:**	Brd4BD2_WT_mr119_9Jan25	**Operator:**	Maria Rodriguez
**Instrument:**		**Injection date:**	2025-01-10 01:10:43-08:00
**Inj. volume:**		**Location:**	P2-A-03
**Acq. method:**	10-75OVER20_PEPTIDES-C3_200MZ.M	**Type:**	Sample
**Processing method:**	*Deconvolution Test 2.pmx	**Sample amount:**	
**Manually modified:**	Manual Integration		

Data Analysis Method: **Deconvolution Test 2.pmx**
Path: **D:\CDSProjects\Walkup Submissions\Results\Chemstatioimports_MRR.rslt**
Method parameters are filtered - only a subset is displayed

2 Method Parameters

2.11 MS Spectral Deconvolution Parameters

**Run automatic deconvolution:**	Yes	**Use RT window:**	No	**TIC peak type:**	All peaks
**TIC peak threshold:**	Top (n) peaks	**Top (n) peaks:**	6	**Positive adduct:**	+H
**Negative adduct:**	-H	**Use m/z range:**	No	**Low molecular weight:**	4000
**High molecular weight:**	25000	**Maximum charge:**	40	**Minimum peaks in set:**	3
**Show unmatched peaks:**	No	**MW agreement (0.01%):**	5	**Absolute noise threshold:**	1000
**Relative abundance threshold (%):**	10	**MW algorithm:**	Curve Fit	**MW algorithm threshold (%):**	40
**Envelope threshold (%):**	50				

Method Audit Trail

Method audit trail is not printed

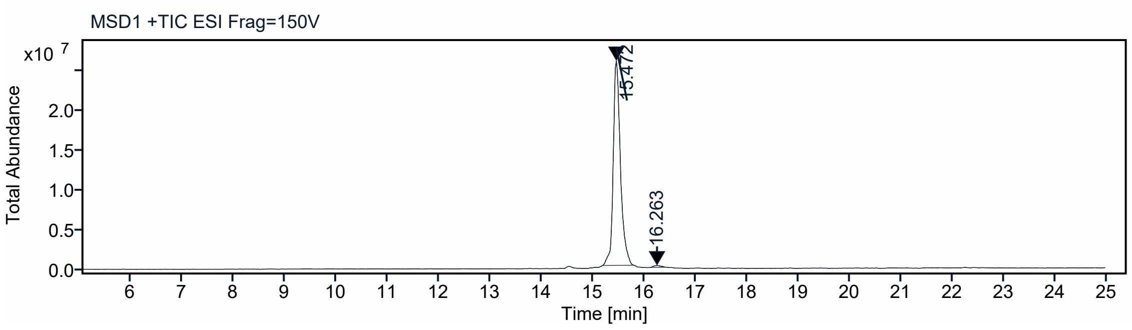

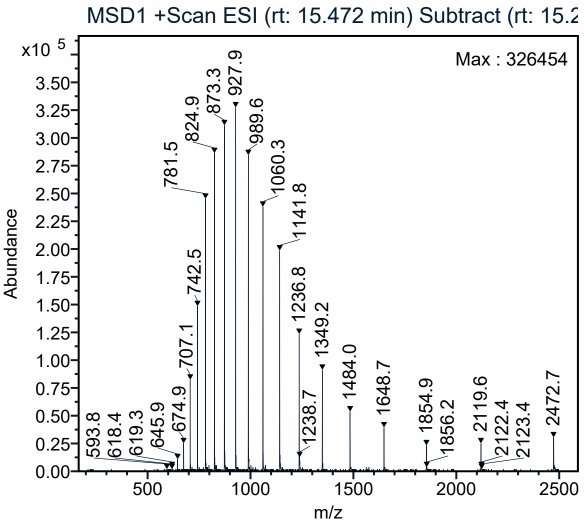

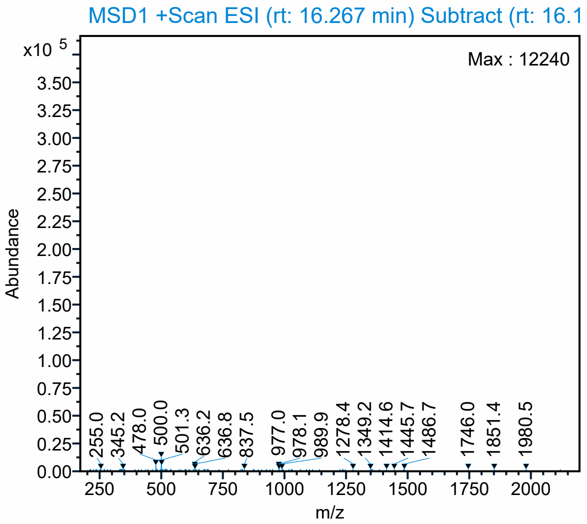

Deconvolution of peak at RT: 15.472

Signal: MSD1 +TIC ESI Frag=150V

Spectrum: MSD1 +Scan ESI (rt: 15.472 min) Subtract (rt: 15.206 min)

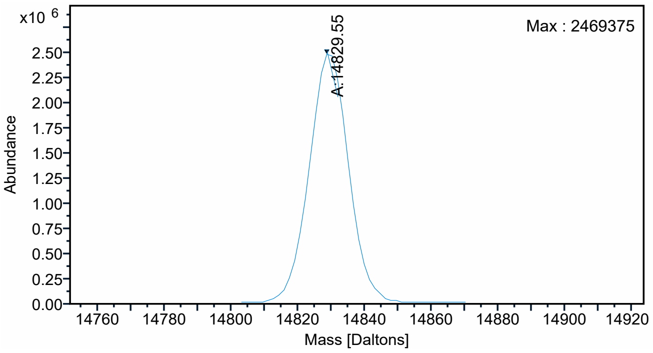

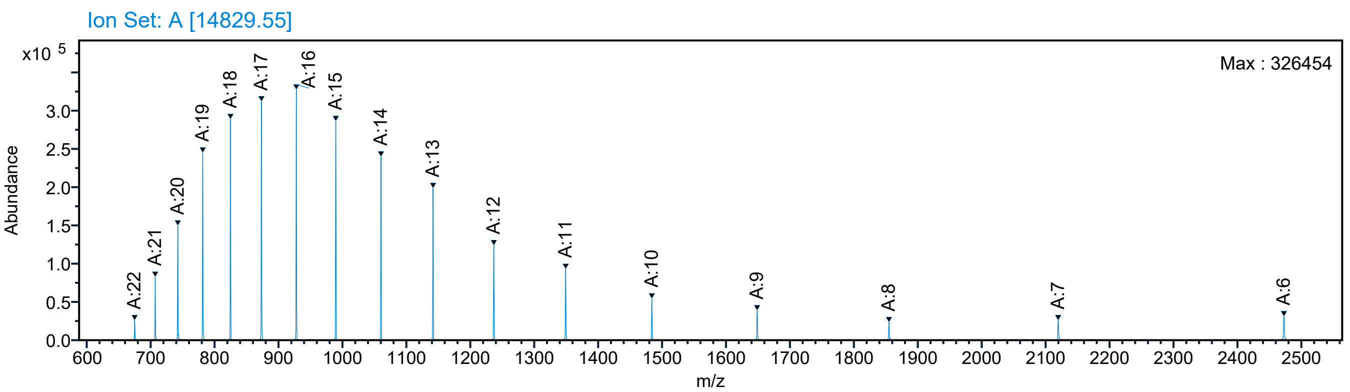

Component	Mass	Absolute Abundance	Relative Abundance (%)	Relative Quantitation (%)
A	14829.55	2469375	100.00	100.00

Single Injection Report

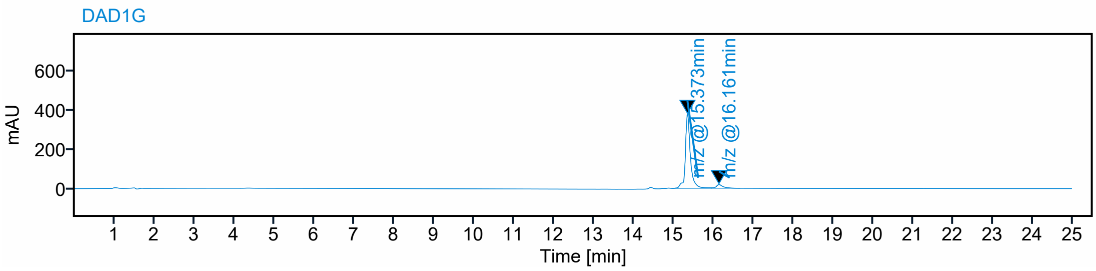

Signal: DAD1G

Name	RT [min]	RF	Area	Peak Area Percent	Group
	15.373		3278.2807	93.64	unmodified protein
no protein detected	~~16.161~~		~~222.7017~~	~~6.36~~	

Signal: MSD1 +TIC ESI Frag=150V

Name	RT [min]	RF	Area	Peak Area Percent	Group
	15.472		250090555.9303	99.12	
no protein detected	~~16.263~~		~~2220306.0341~~	~~0.88~~	

unmodified protein

Single Injection Report

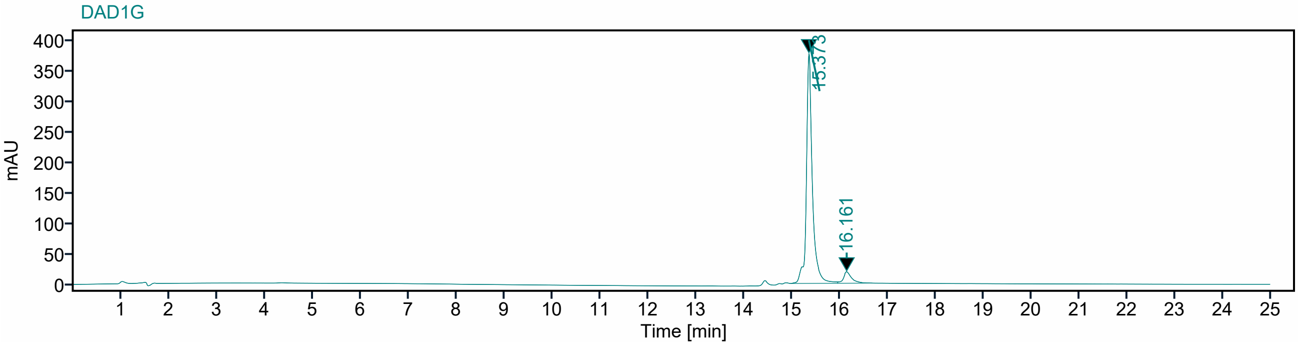

**Data file:**	090125-10-75OVER20_PEPTIDES-C3_200M-30681_016.D		
**Sequence Name:**	Chemstatioimports_MRR	**Project Name:**	Walkup Submissions
**Sample name:**	Brd4BD2_WT_mr104_9Jan25	**Operator:**	Maria Rodriguez
**Instrument:**		**Injection date:**	2025-01-10 01:40:34-08:00
**Inj. volume:**		**Location:**	P2-A-04
**Acq. method:**	10-75OVER20_PEPTIDES-C3_200MZ.M	**Type:**	Sample
**Processing method:**	*Deconvolution Test 2.pmx	**Sample amount:**	
**Manually modified:**	Manual Integration		

Data Analysis Method: **Deconvolution Test 2.pmx**
Path: **D:\CDSProjects\Walkup Submissions\Results\Chemstatioimports_MRR.rslt**
Method parameters are filtered - only a subset is displayed

2 Method Parameters

2.11 MS Spectral Deconvolution Parameters

**Run automatic deconvolution:**	Yes	**Use RT window:**	No	**TIC peak type:**	All peaks
**TIC peak threshold:**	Top (n) peaks	**Top (n) peaks:**	6	**Positive adduct:**	+H
**Negative adduct:**	-H	**Use m/z range:**	No	**Low molecular weight:**	4000
**High molecular weight:**	25000	**Maximum charge:**	40	**Minimum peaks in set:**	3
**Show unmatched peaks:**	No	**MW agreement (0.01%):**	5	**Absolute noise threshold:**	1000
**Relative abundance threshold (%):**	10	**MW algorithm:**	Curve Fit	**MW algorithm threshold (%):**	40
**Envelope threshold (%):**	50				

Method Audit Trail

Method audit trail is not printed

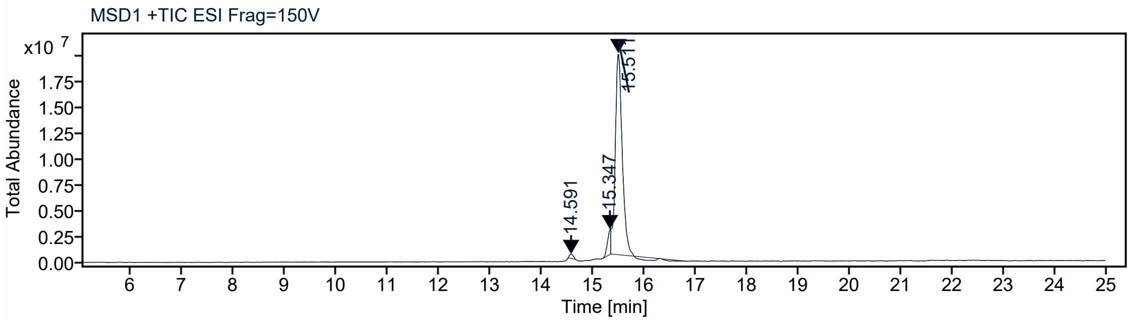

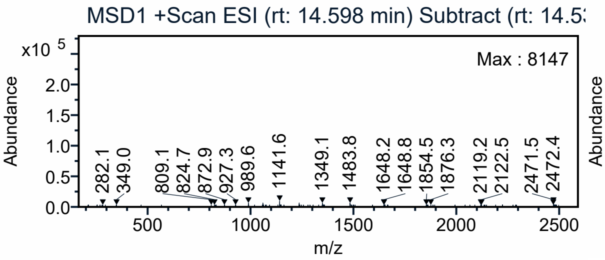

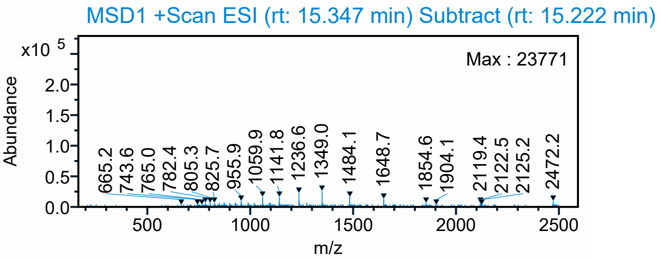

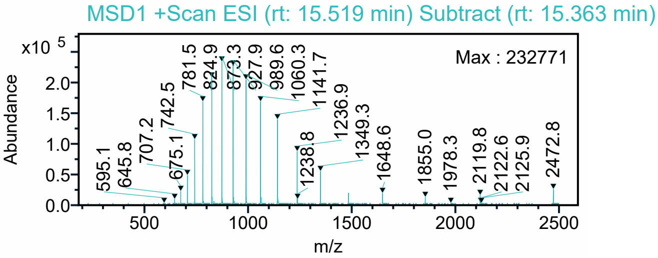

Deconvolution of peak at RT: 14.591

Signal: MSD1 +TIC ESI Frag=150V

Spectrum: MSD1 +Scan ESI (rt: 14.598 min) Subtract (rt: 14.536 min)

No spectra available!

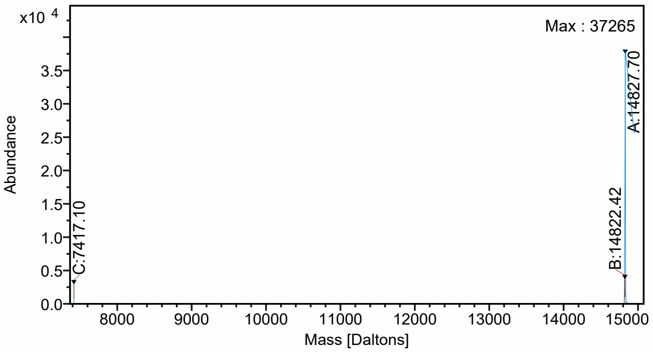

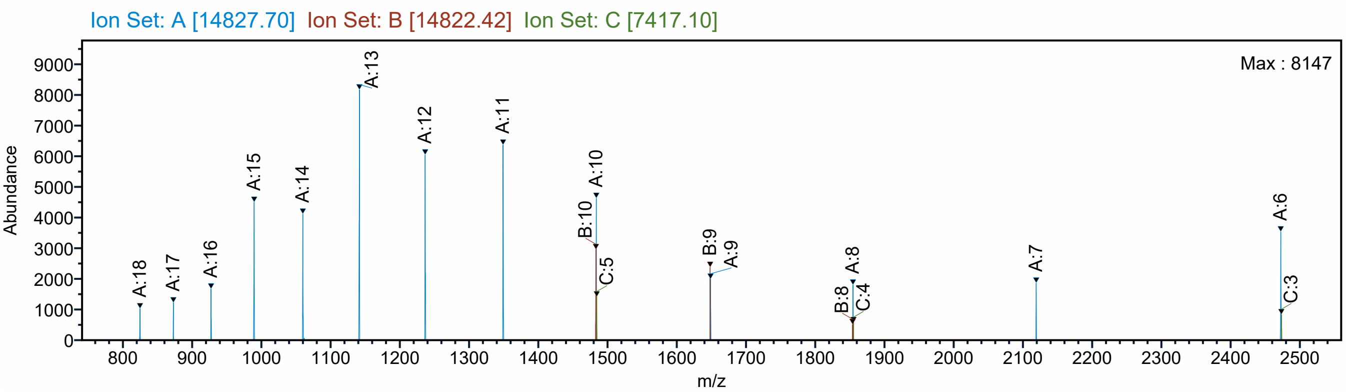

Component	Mass	Absolute Abundance	Relative Abundance (%)	Relative Quantitation (%)
A	14827.70	37265	100.00	86.08
B	14822.42	3422	9.18	7.90
C	7417.10	2603	6.99	6.01

Deconvolution of peak at RT: 15.347

Signal: MSD1 +TIC ESI Frag=150V

Spectrum: MSD1 +Scan ESI (rt: 15.347 min) Subtract (rt: 15.222 min)

No spectra available!

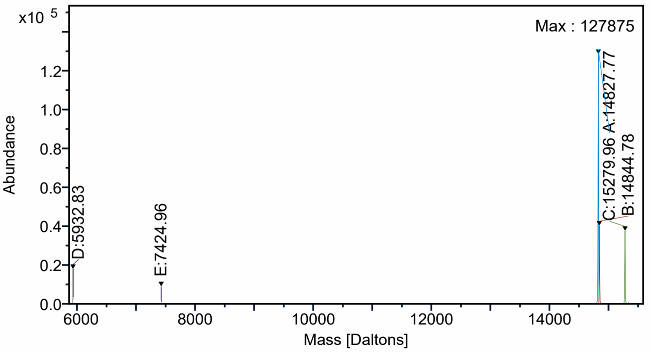

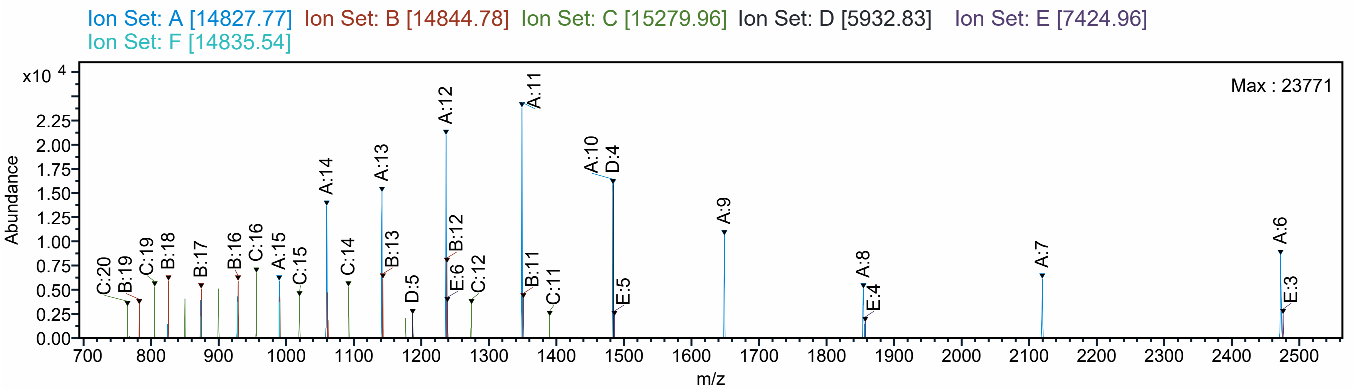

Component	Mass	Absolute Abundance	Relative Abundance (%)	Relative Quantitation (%)
A	14827.77	127875	100.00	53.56
B	14844.78	40074	31.34	16.79
C	15279.96	36603	28.62	15.33
D	5932.83	17268	13.50	7.23
E	7424.96	8563	6.70	3.59
F	14835.54	8359	6.54	3.50

Deconvolution of peak at RT: 15.511

Signal: MSD1 +TIC ESI Frag=150V

Spectrum: MSD1 +Scan ESI (rt: 15.519 min) Subtract (rt: 15.363 min)

No spectra available!

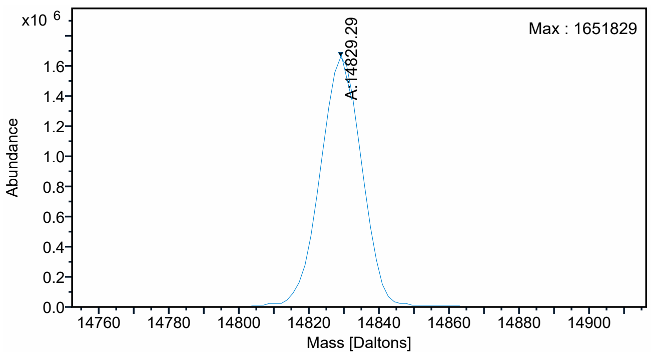

Ion Set: A [14829.29]

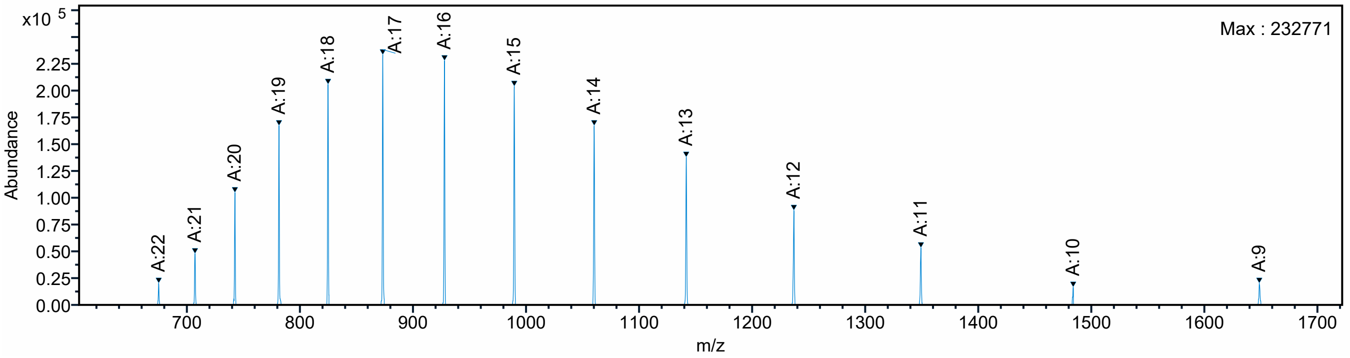

Component	Mass	Absolute Abundance	Relative Abundance (%)	Relative Quantitation (%)
A	14829.29	1651829	100.00	100.00

DAD1G

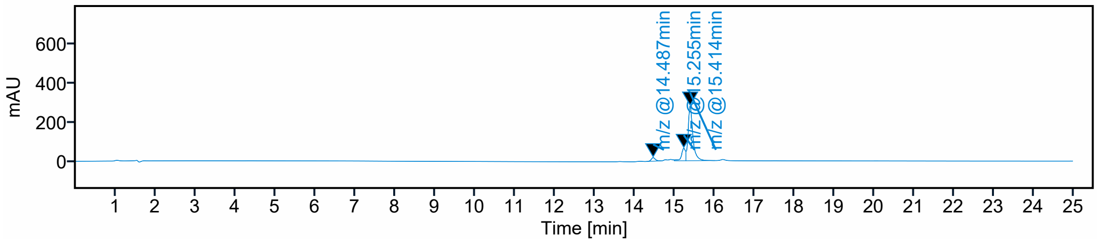

Single Injection Report

Signal: DAD1G

Name	RT [min]	RF	Area	Peak Area Percent	Group
	14.487		129.4738	4.50	
	15.255		443.1623	15.39	modified protein <15% (relative abundance in MS peak 15%)
	15.414		2306.9833	80.11	unmodified protein

Signal: MSD1 +TIC ESI Frag=150V

Name	RT [min]	RF	Area	Peak Area Percent	Group
	14.591		2196380.6238	1.17	
	15.347		9995929.7537	5.33	modified protein <5% (relative abundance in MS peak 15%)
	15.511		175463390.9477	93.50	unmodified protein

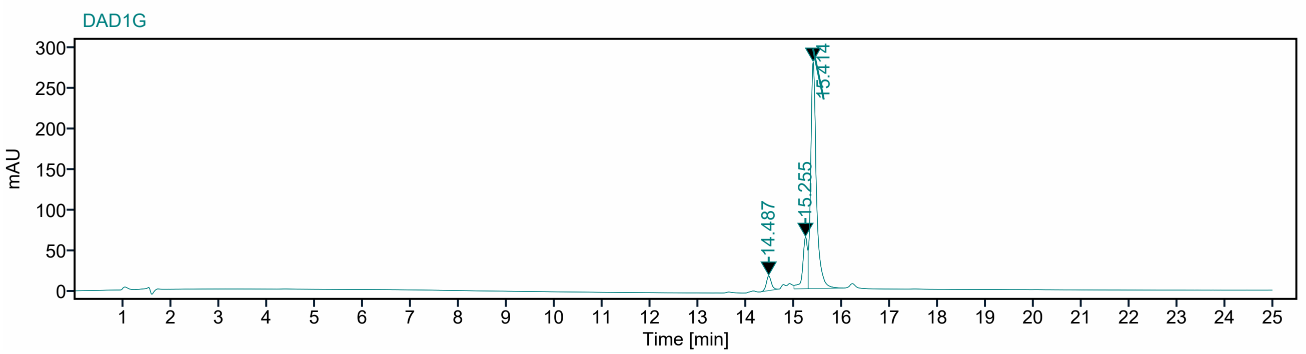

Data File D:\EZXDATA\MARIARODRIGUEZ\12-24\061224-10-75OVER20_PEPTIDES-C3_200M-30604.D
Sample Name: MUT73_DMSO_1_6DEC24

===

```
Acq. Operator   : Maria Rodriguez
Acq. Instrument : INSTRUMENT 1                    Location : P1-A-01
Injection Date  : 12/6/2024 4:50:04 PM                 Inj :    1
                                               Inj Volume : 10.000 µl
Acq. Method     : C:\CHEM32\1\METHODS\10-75OVER20_PEPTIDE
Last changed    : 12/6/2024 4:49:03 PM by Maria Rodriguez
                  (modified after loading)
Analysis Method : C:\CHEM32\1\METHODS\10-75OVER20_PEPTIDES-5UL-C3.M
Last changed    : 12/19/2024 5:40:28 PM by Liam Hales
                  (modified after loading)
Sample Info     : Easy-Access Method: '10-75over20-C3(200+m/Z)'

Additional Info : Peak(s) manually integrated
```

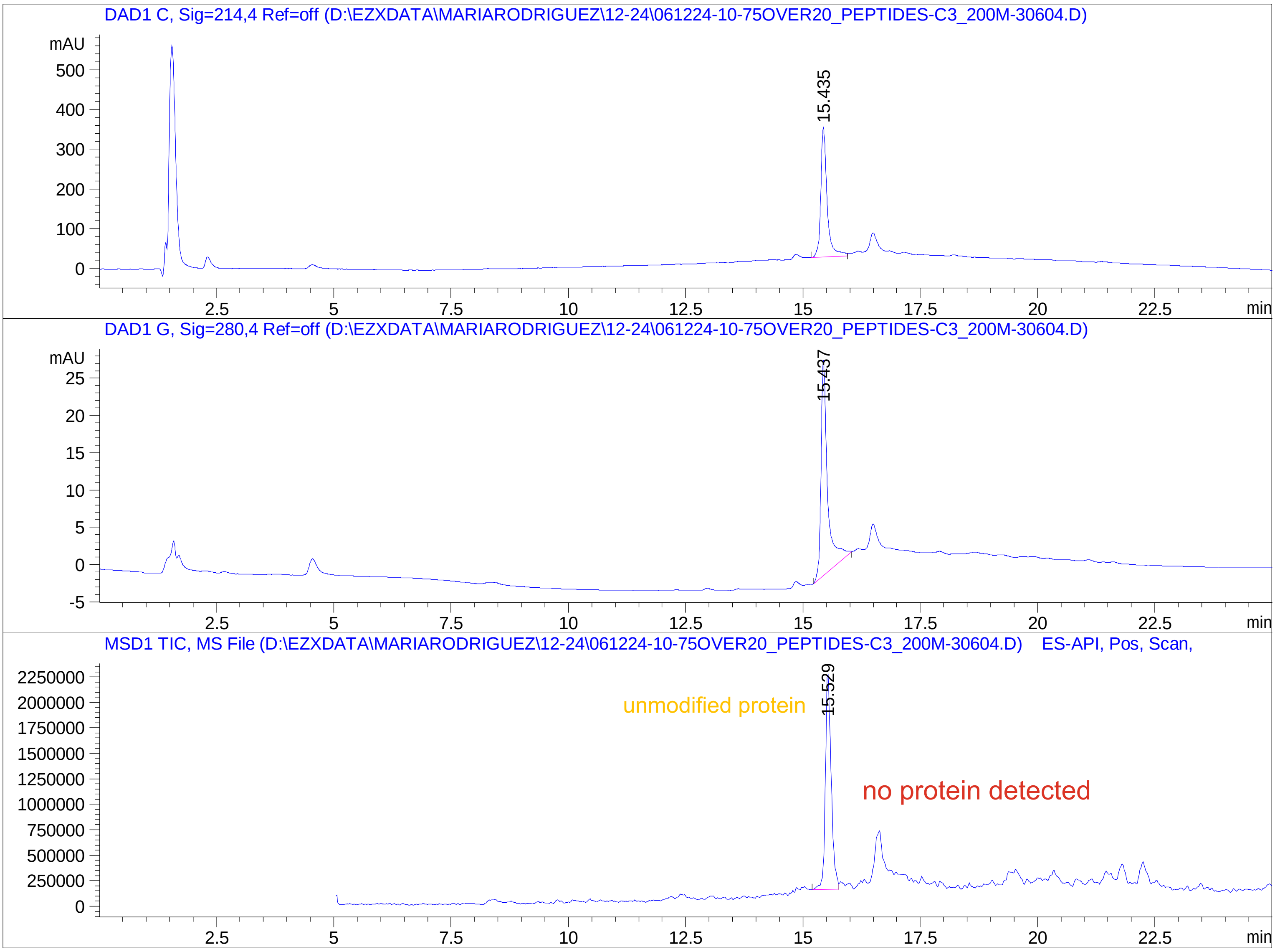

===

Area Percent Report

===

```
Sorted By             :      Signal
Multiplier            :      1.0000
Dilution              :      1.0000
Sample Amount:               :      15.00000  [ng/ul]   (not used in calc.)
Use Multiplier & Dilution Factor with ISTDs
```

Signal 1: DAD1 C, Sig=214,4 Ref=off

Peak #	RetTime [min]	Type	Width [min]	Area [mAU*s]	Height [mAU]	Area %
1	15.435	BV	0.1402	3076.54980	325.57642	100.0000
Totals :				3076.54980	325.57642	

Signal 2: DAD1 G, Sig=280,4 Ref=off

Peak #	RetTime [min]	Type	Width [min]	Area [mAU*s]	Height [mAU]	Area %
1	15.437	BB	0.1415	280.73364	28.84967	100.0000
Totals :				280.73364	28.84967	

Signal 3: MSD1 TIC, MS File

Peak #	RetTime [min]	Type	Width [min]	Area	Height	Area %
1	15.529	BV	0.1346	1.84930e7	2.11771e6	100.0000
Totals :				1.84930e7	2.11771e6	

===

*** End of Report ***

```
Easy-Access Method: '10-75over20-C3(200+m/Z)'
=====================================================================
Acq. Operator   : Maria Rodriguez
Acq. Instrument : INSTRUMENT 1                    Location : P1-A-01
Injection Date  : 12/6/2024 4:50:04 PM                 Inj :   1
                                                Inj Volume : 10.000 µl
Acq. Method     : C:\CHEM32\1\METHODS\10-75OVER20_PEPTIDE
Last changed    : 12/6/2024 4:49:03 PM by Maria Rodriguez
                  (modified after loading)
Analysis Method : C:\CHEM32\1\METHODS\10-75OVER20_PEPTIDES-5UL-C3.M
Last changed    : 12/19/2024 5:40:00 PM by Liam Hales
                  (modified after loading)
Sample Info     : Easy-Access Method: '10-75over20-C3(200+m/Z)'

Additional Info : Peak(s) manually integrated

=====================================================================
                      Deconvolution Parameters
=====================================================================

Adduct Ion(Positive): +H, 1.0079 Da
Adduct Ion(Negative):  , 0.0000 Da
Low MW:               9000
DeconvStartChgMaximum Charge:       50
Minimum Peaks in Set: 3
Retain Residual:      No
Ion PWHH:             0.6 Da
MW Agreement:         0.05 %
Noise Cutoff:         1000 counts
Abundance Cutoff:     10 %
MW Assign:            Curve fit
MW Assign Cutoff:     40 %
Envelope Cutoff:      50 %
```

Deconvolution of Spectrum # 1 @ 15.175 - 16.221 min

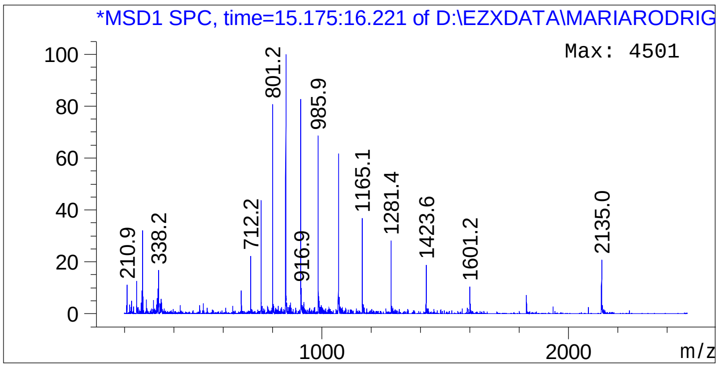

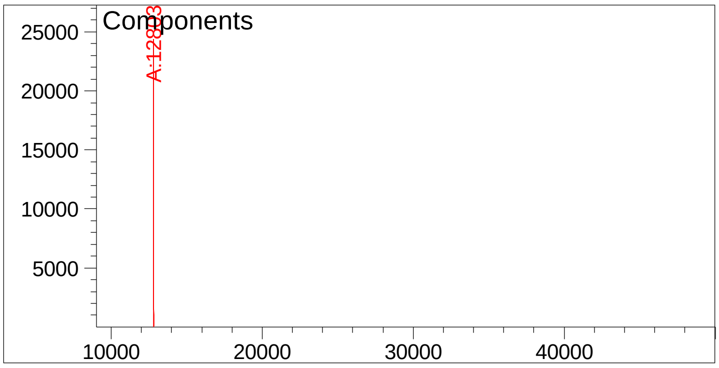

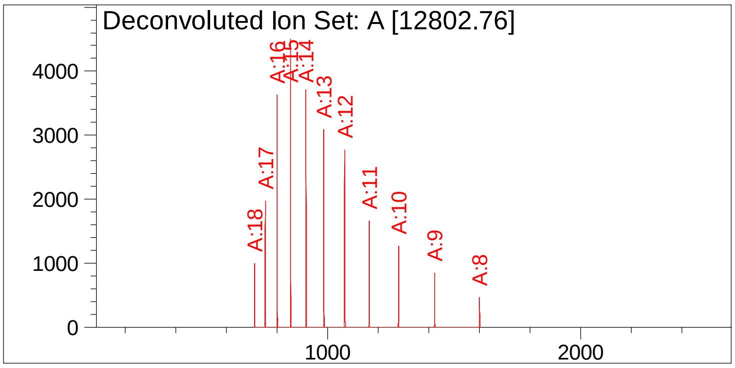

Component	Molecular Weight	Absolute Abundance	Relative Abundance
A	12802.76	24375	100.00

*** End of Report ***

Data File D:\EZXDATA\MARIARODRIGUEZ\11-24\261124-10-75OVER20_PEPTIDES-C3_200M-30493.D
Sample Name: MUT73_MR70_4_26NOV24

===

Acq. Operator   : Maria Rodriguez
Acq. Instrument : INSTRUMENT 1   Location : P1-D-05
Injection Date : 11/26/2024 9:49:04 PM   Inj :   1
                                            Inj Volume : 10.000 µl
Acq. Method   : C:\CHEM32\1\METHODS\10-75OVER20_PEPTIDE
Last changed   : 11/26/2024 9:48:08 PM by Maria Rodriguez
                  (modified after loading)
Analysis Method : C:\CHEM32\1\METHODS\10-75OVER20_PEPTIDES-5UL-C3.M
Last changed   : 12/19/2024 5:59:55 PM by Liam Hales
                  (modified after loading)
Sample Info   : Easy-Access Method: '10-75over20-C3(200+m/Z)'

Additional Info : Peak(s) manually integrated

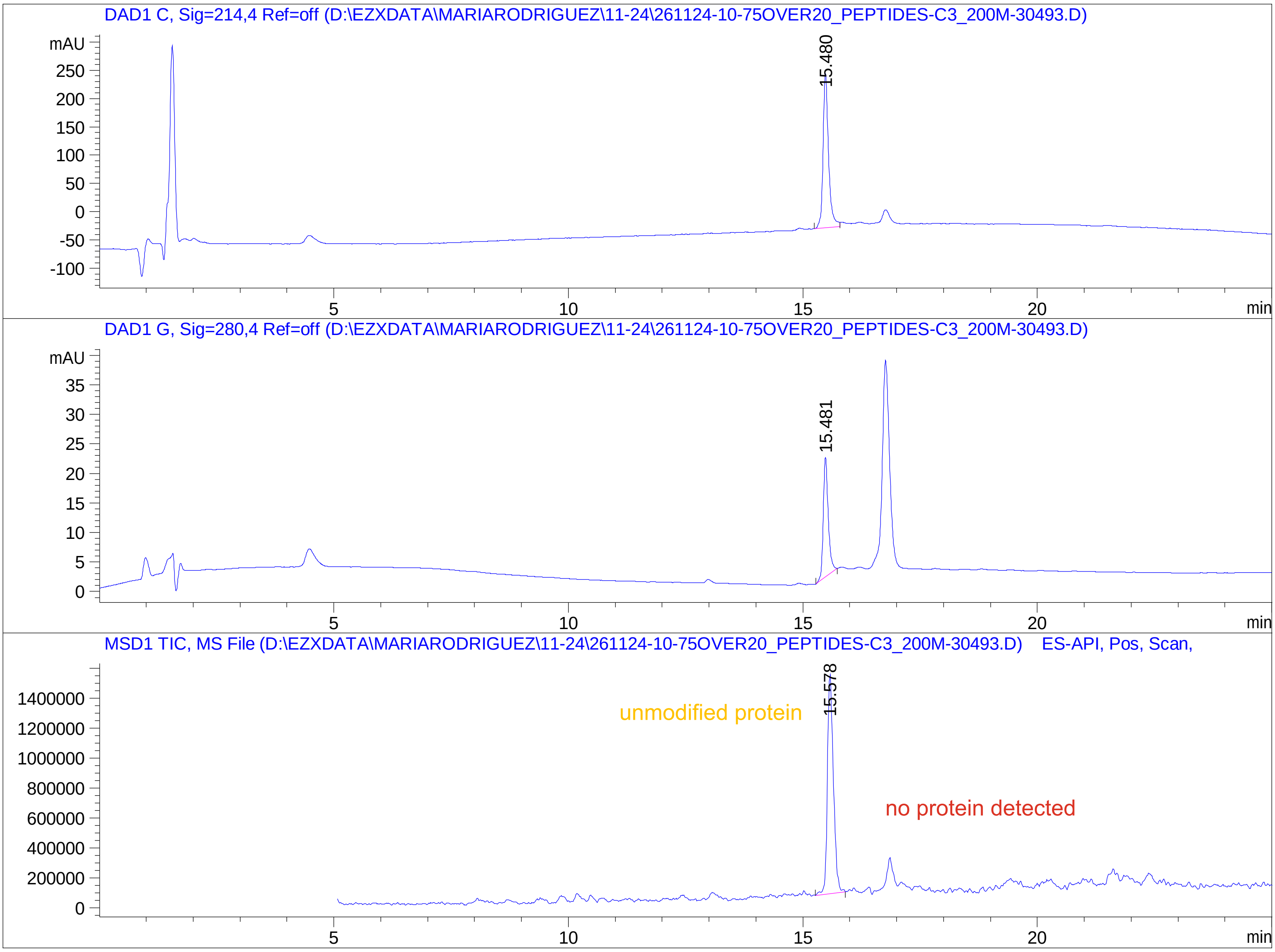

===

Area Percent Report

===

Sorted By   :   Signal
Multiplier   :   1.0000
Dilution   :   1.0000
Sample Amount: :   15.00000 [ng/ul]   (not used in calc.)
Use Multiplier & Dilution Factor with ISTDs

Signal 1: DAD1 C, Sig=214,4 Ref=off

Peak #	RetTime [min]	Type	Width [min]	Area [mAU*s]	Height [mAU]	Area %
1	15.480	BV	0.1206	2214.23926	272.96194	100.0000
Totals :				2214.23926	272.96194	

Signal 2: DAD1 G, Sig=280,4 Ref=off

Peak #	RetTime [min]	Type	Width [min]	Area [mAU*s]	Height [mAU]	Area %
1	15.481	BB	0.1097	148.41934	20.20031	100.0000
Totals :				148.41934	20.20031	

Signal 3: MSD1 TIC, MS File

Peak #	RetTime [min]	Type	Width [min]	Area	Height	Area %
1	15.578	BB	0.1310	1.29047e7	1.46229e6	100.0000
Totals :				1.29047e7	1.46229e6	

==

*** End of Report ***

```
Easy-Access Method: '10-75over20-C3(200+m/Z)'
=====================================================================
Acq. Operator   : Maria Rodriguez
Acq. Instrument : INSTRUMENT 1                    Location : P1-D-05
Injection Date  : 11/26/2024 9:49:04 PM                Inj :   1
                                                Inj Volume : 10.000 µl
Acq. Method     : C:\CHEM32\1\METHODS\10-75OVER20_PEPTIDE
Last changed    : 11/26/2024 9:48:08 PM by Maria Rodriguez
                  (modified after loading)
Analysis Method : C:\CHEM32\1\METHODS\10-75OVER20_PEPTIDES-5UL-C3.M
Last changed    : 12/19/2024 5:59:53 PM by Liam Hales
                  (modified after loading)
Sample Info     : Easy-Access Method: '10-75over20-C3(200+m/Z)'

Additional Info : Peak(s) manually integrated

=====================================================================
                      Deconvolution Parameters
=====================================================================

Adduct Ion(Positive): +H, 1.0079 Da
Adduct Ion(Negative):  , 0.0000 Da
Low MW:               9000
DeconvStartChgMaximum Charge:       50
Minimum Peaks in Set: 3
Retain Residual:      No
Ion PWHH:             0.6 Da
MW Agreement:         0.05 %
Noise Cutoff:         1000 counts
Abundance Cutoff:     10 %
MW Assign:            Curve fit
MW Assign Cutoff:     40 %
Envelope Cutoff:      50 %
```

Deconvolution of Spectrum # 1 @ 14.754 - 16.002 min

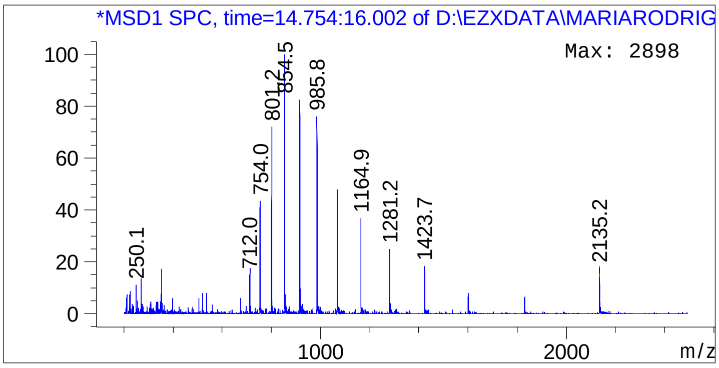

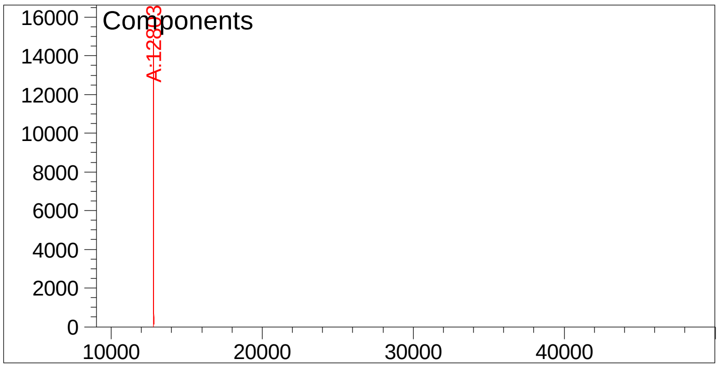

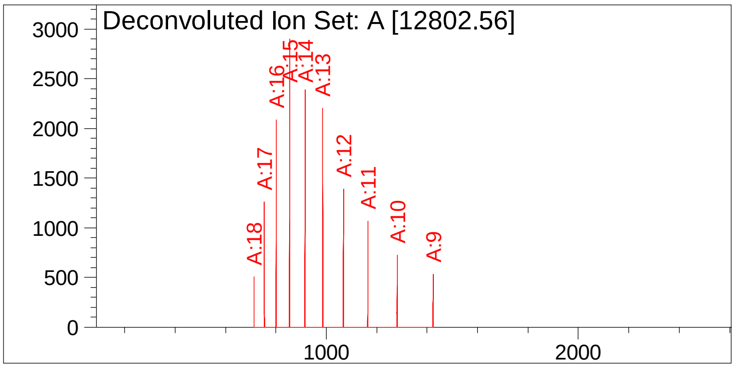

Component	Molecular Weight	Absolute Abundance	Relative Abundance
A	12802.56	14860	100.00

*** End of Report ***

```
Easy-Access Method: '10-75over20-C3(200+m/Z)'
=====================================================================
Acq. Operator   : Maria Rodriguez
Acq. Instrument : INSTRUMENT 1                    Location : P1-D-06
Injection Date  : 11/26/2024 10:19:01 PM               Inj :   1
                                                Inj Volume : 10.000 µl
Acq. Method     : C:\CHEM32\1\METHODS\10-75OVER20_PEPTIDE
Last changed    : 11/26/2024 10:18:05 PM by Maria Rodriguez
                  (modified after loading)
Analysis Method : C:\CHEM32\1\METHODS\10-75OVER20_PEPTIDES-5UL-C3.M
Last changed    : 12/19/2024 6:00:48 PM by Liam Hales
                  (modified after loading)
Sample Info     : Easy-Access Method: '10-75over20-C3(200+m/Z)'

Additional Info : Peak(s) manually integrated

=====================================================================
                      Deconvolution Parameters
=====================================================================

Adduct Ion(Positive): +H, 1.0079 Da
Adduct Ion(Negative):  , 0.0000 Da
Low MW:               9000
DeconvStartChgMaximum Charge:       50
Minimum Peaks in Set: 3
Retain Residual:      No
Ion PWHH:             0.6 Da
MW Agreement:         0.05 %
Noise Cutoff:         1000 counts
Abundance Cutoff:     10 %
MW Assign:            Curve fit
MW Assign Cutoff:     40 %
Envelope Cutoff:      50 %
```

Spectrum Deconvolution
Data File D:\EZXDATA\MARIARODRIGUEZ\11-24\261124-10-75OVER20_PEPTIDES-C3_200M-30494.D
Sample Name: MUT73_MR104_5_26NOV24

Deconvolution of Spectrum # 1 @ 15.893 - 17.640 min

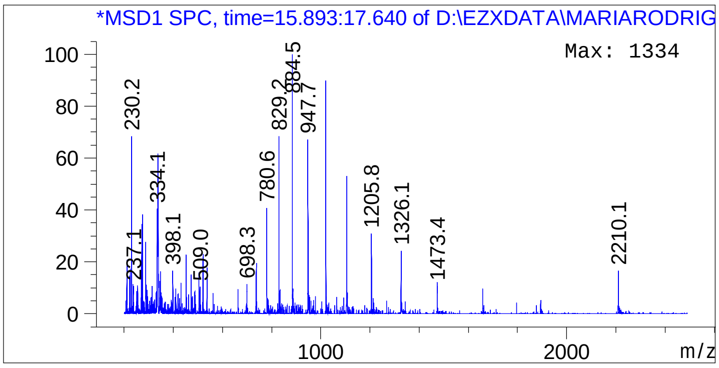

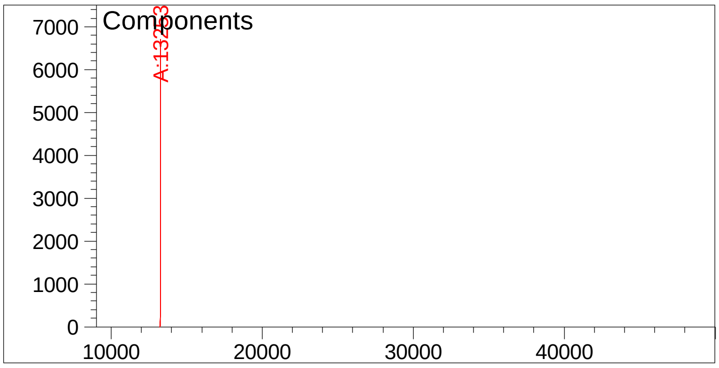

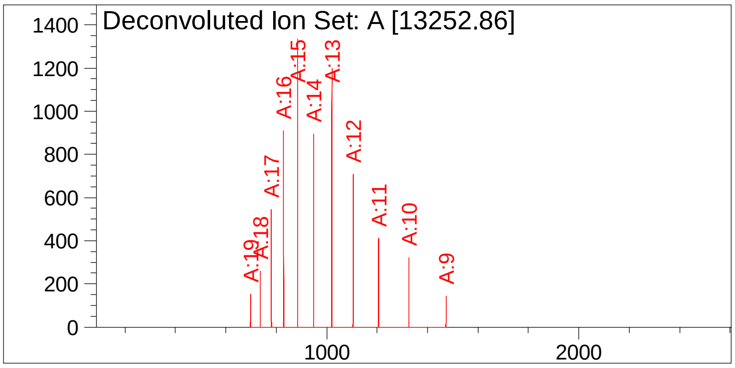

Component	Molecular Weight	Absolute Abundance	Relative Abundance
A	13252.86	6709	100.00

*** End of Report ***

===

Acq. Operator : Maria Rodriguez
Acq. Instrument : INSTRUMENT 1 Location : P1-D-06
Injection Date : 11/26/2024 10:19:01 PM Inj : 1
Inj Volume : 10.000 µl
Acq. Method : C:\CHEM32\1\METHODS\10-75OVER20_PEPTIDE
Last changed : 11/26/2024 10:18:05 PM by Maria Rodriguez
(modified after loading)
Analysis Method : C:\CHEM32\1\METHODS\10-75OVER20_PEPTIDES-5UL-C3.M
Last changed : 12/19/2024 6:00:50 PM by Liam Hales
(modified after loading)
Sample Info : Easy-Access Method: '10-75over20-C3(200+m/Z)'

Additional Info : Peak(s) manually integrated

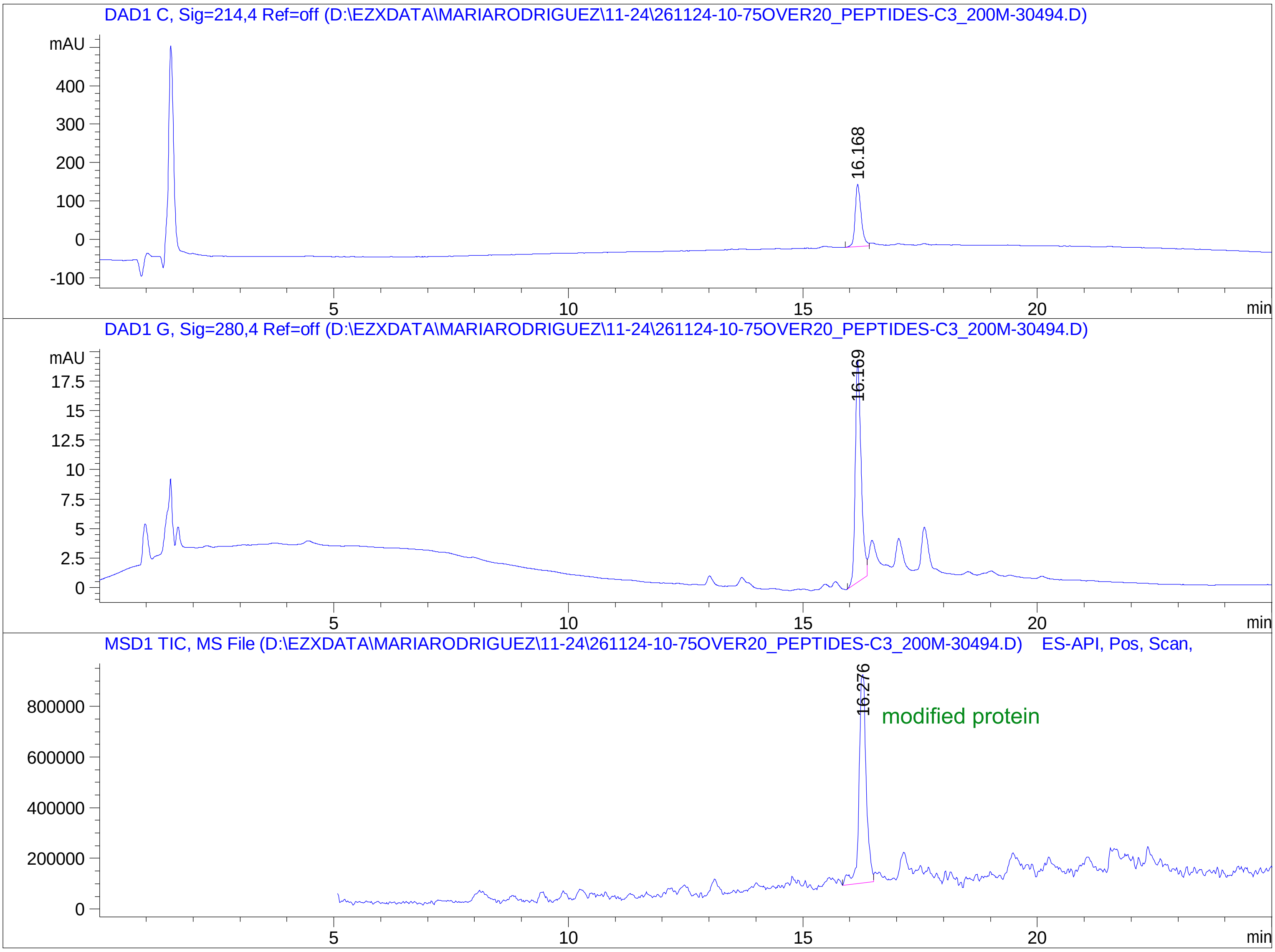

===
Area Percent Report
===

Sorted By : Signal
Multiplier : 1.0000
Dilution : 1.0000
Sample Amount: : 15.00000 [ng/ul] (not used in calc.)
Use Multiplier & Dilution Factor with ISTDs

Signal 1: DAD1 C, Sig=214,4 Ref=off

Peak #	RetTime [min]	Type	Width [min]	Area [mAU*s]	Height [mAU]	Area %
1	16.168	BV	0.1358	1447.08423	162.57765	100.0000
Totals :				1447.08423	162.57765	

Signal 2: DAD1 G, Sig=280,4 Ref=off

Peak #	RetTime [min]	Type	Width [min]	Area [mAU*s]	Height [mAU]	Area %
1	16.169	BV	0.1313	163.51889	18.83063	100.0000
Totals :				163.51889	18.83063	

Signal 3: MSD1 TIC, MS File

Peak #	RetTime [min]	Type	Width [min]	Area	Height	Area %
1	16.276	BV	0.1566	8.76225e6	8.25419e5	100.0000
Totals :				8.76225e6	8.25419e5	

===

*** End of Report ***

Data File D:\EZXDATA\MARIARODRIGUEZ\11-24\261124-10-75OVER20_PEPTIDES-C3_200M-30501.D
Sample Name: MUT73_MR111_12_26NOV24

===
```
Acq. Operator   : Maria Rodriguez
Acq. Instrument : INSTRUMENT 1                    Location : P1-E-04
Injection Date  : 11/27/2024 1:48:40 AM                Inj :    1
                                                Inj Volume : 10.000 µl
Acq. Method     : C:\CHEM32\1\METHODS\10-75OVER20_PEPTIDE
Last changed    : 11/27/2024 1:47:44 AM by Maria Rodriguez
                  (modified after loading)
Analysis Method : C:\CHEM32\1\METHODS\10-75OVER20_PEPTIDES-5UL-C3.M
Last changed    : 12/19/2024 5:54:08 PM by Liam Hales
                  (modified after loading)
Sample Info     : Easy-Access Method: '10-75over20-C3(200+m/Z)'

Additional Info : Peak(s) manually integrated
```

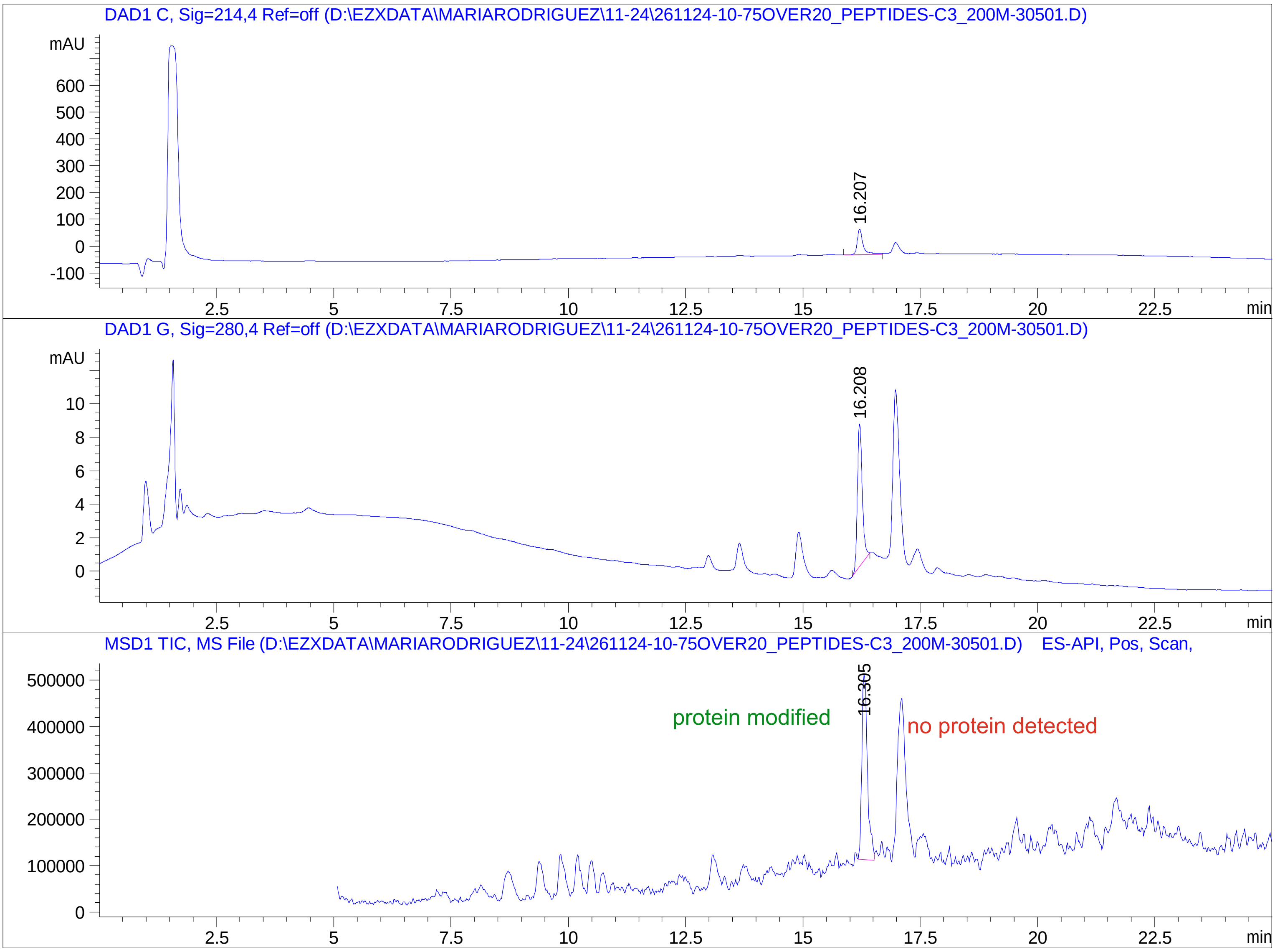

===
Area Percent Report
===

```
Sorted By             :      Signal
Multiplier            :      1.0000
Dilution              :      1.0000
Sample Amount:               :      15.00000  [ng/ul]   (not used in calc.)
Use Multiplier & Dilution Factor with ISTDs
```

Signal 1: DAD1 C, Sig=214,4 Ref=off

Peak #	RetTime [min]	Type	Width [min]	Area [mAU*s]	Height [mAU]	Area %
1	16.207	BV	0.1307	857.40656	95.53775	100.0000

Totals : 857.40656 95.53775

Signal 2: DAD1 G, Sig=280,4 Ref=off

Peak #	RetTime [min]	Type	Width [min]	Area [mAU*s]	Height [mAU]	Area %
1	16.208	BB	0.1090	60.83061	8.55526	100.0000

Totals : 60.83061 8.55526

Signal 3: MSD1 TIC, MS File

Peak #	RetTime [min]	Type	Width [min]	Area	Height	Area %
1	16.305	BV	0.1289	3.30249e6	4.00963e5	100.0000

Totals : 3.30249e6 4.00963e5

*** End of Report ***

```
Easy-Access Method: '10-75over20-C3(200+m/Z)'
=====================================================================
Acq. Operator   : Maria Rodriguez
Acq. Instrument : INSTRUMENT 1                     Location : P1-E-04
Injection Date  : 11/27/2024 1:48:40 AM                 Inj :    1
                                                Inj Volume : 10.000 µl
Acq. Method     : C:\CHEM32\1\METHODS\10-75OVER20_PEPTIDE
Last changed    : 11/27/2024 1:47:44 AM by Maria Rodriguez
                  (modified after loading)
Analysis Method : C:\CHEM32\1\METHODS\10-75OVER20_PEPTIDES-5UL-C3.M
Last changed    : 12/19/2024 5:54:08 PM by Liam Hales
                  (modified after loading)
Sample Info     : Easy-Access Method: '10-75over20-C3(200+m/Z)'

Additional Info : Peak(s) manually integrated

=====================================================================
                      Deconvolution Parameters
=====================================================================

Adduct Ion(Positive): +H, 1.0079 Da
Adduct Ion(Negative):  , 0.0000 Da
Low MW:               9000
DeconvStartChgMaximum Charge:       50
Minimum Peaks in Set: 3
Retain Residual:      No
Ion PWHH:             0.6 Da
MW Agreement:         0.05 %
Noise Cutoff:         1000 counts
Abundance Cutoff:     10 %
MW Assign:            Curve fit
MW Assign Cutoff:     40 %
Envelope Cutoff:      50 %
```

Deconvolution of Spectrum # 1 @ 16.096 - 16.782 min

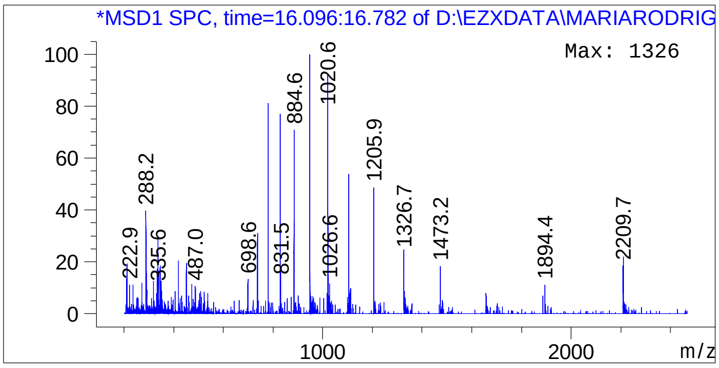

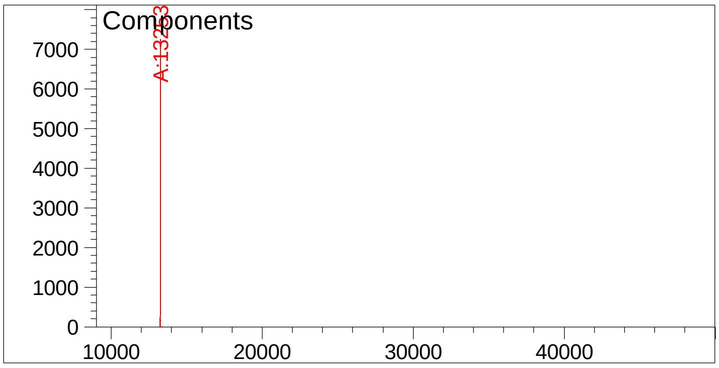

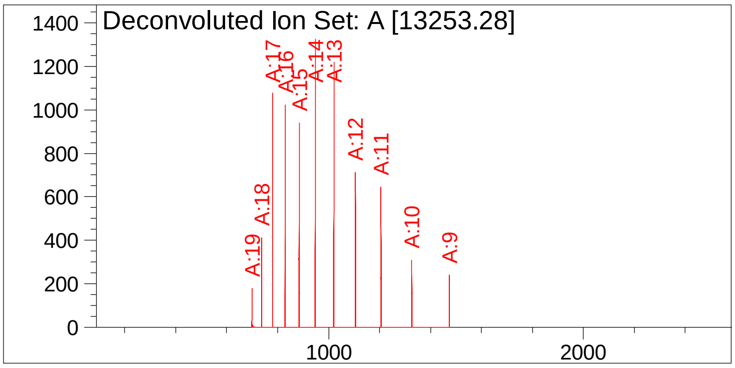

Component	Molecular Weight	Absolute Abundance	Relative Abundance
A	13253.28	7254	100.00

*** End of Report ***

Data File D:\EZXDATA\MARIARODRIGUEZ\11-24\261124-10-75OVER20_PEPTIDES-C3_200M-30495.D
Sample Name: MUT73_MR112_6_26NOV24

===

Acq. Operator : Maria Rodriguez
Acq. Instrument : INSTRUMENT 1 Location : P1-D-07
Injection Date : 11/26/2024 10:48:57 PM Inj : 1
Inj Volume : 10.000 µl
Acq. Method : C:\CHEM32\1\METHODS\10-75OVER20_PEPTIDE
Last changed : 11/26/2024 10:48:02 PM by Maria Rodriguez
(modified after loading)
Analysis Method : C:\CHEM32\1\METHODS\10-75OVER20_PEPTIDES-5UL-C3.M
Last changed : 12/19/2024 6:02:52 PM by Liam Hales
(modified after loading)
Sample Info : Easy-Access Method: '10-75over20-C3(200+m/Z)'

Additional Info : Peak(s) manually integrated

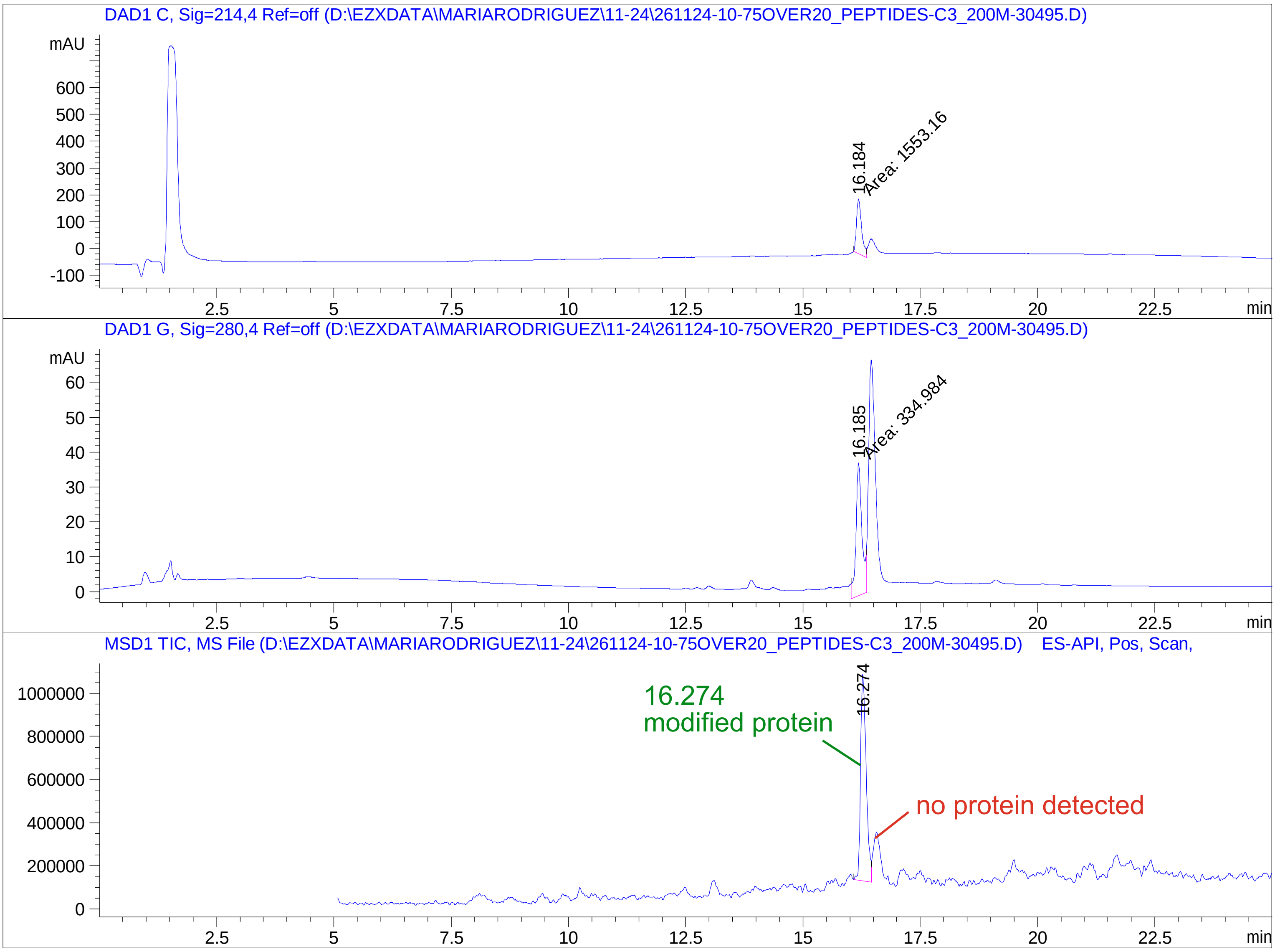

===
Area Percent Report
===

Sorted By : Signal
Multiplier : 1.0000
Dilution : 1.0000
Sample Amount: : 15.00000 [ng/ul] (not used in calc.)
Use Multiplier & Dilution Factor with ISTDs

Signal 1: DAD1 C, Sig=214,4 Ref=off

Peak #	RetTime [min]	Type	Width [min]	Area [mAU*s]	Height [mAU]	Area %
1	16.184	MM	0.1276	1553.16296	202.88480	100.0000

Totals : 1553.16296 202.88480

Signal 2: DAD1 G, Sig=280,4 Ref=off

Peak #	RetTime [min]	Type	Width [min]	Area [mAU*s]	Height [mAU]	Area %
1	16.185	MM	0.1469	334.98407	37.99491	100.0000

Totals : 334.98407 37.99491

Signal 3: MSD1 TIC, MS File

Peak #	RetTime [min]	Type	Width [min]	Area	Height	Area %
1	16.274	BV	0.1195	7.97152e6	9.67377e5	100.0000

Totals : 7.97152e6 9.67377e5

===

*** End of Report ***

```
Easy-Access Method: '10-75over20-C3(200+m/Z)'
=====================================================================
Acq. Operator   : Maria Rodriguez
Acq. Instrument : INSTRUMENT 1                     Location : P1-D-07
Injection Date  : 11/26/2024 10:48:57 PM                Inj :   1
                                                 Inj Volume : 10.000 µl
Acq. Method     : C:\CHEM32\1\METHODS\10-75OVER20_PEPTIDE
Last changed    : 11/26/2024 10:48:02 PM by Maria Rodriguez
                  (modified after loading)
Analysis Method : C:\CHEM32\1\METHODS\10-75OVER20_PEPTIDES-5UL-C3.M
Last changed    : 12/19/2024 6:02:50 PM by Liam Hales
                  (modified after loading)
Sample Info     : Easy-Access Method: '10-75over20-C3(200+m/Z)'

Additional Info : Peak(s) manually integrated

=====================================================================
                      Deconvolution Parameters
=====================================================================

Adduct Ion(Positive): +H, 1.0079 Da
Adduct Ion(Negative):  , 0.0000 Da
Low MW:               9000
DeconvStartChgMaximum Charge:       50
Minimum Peaks in Set: 3
Retain Residual:      No
Ion PWHH:             0.6 Da
MW Agreement:         0.05 %
Noise Cutoff:         1000 counts
Abundance Cutoff:     10 %
MW Assign:            Curve fit
MW Assign Cutoff:     40 %
Envelope Cutoff:      50 %
```

Spectrum Deconvolution
Data File D:\EZXDATA\MARIARODRIGUEZ\11-24\261124-10-75OVER20_PEPTIDES-C3_200M-30495.D
Sample Name: MUT73_MR112_6_26NOV24

Deconvolution of Spectrum # 1 @ 16.127 - 16.829 min

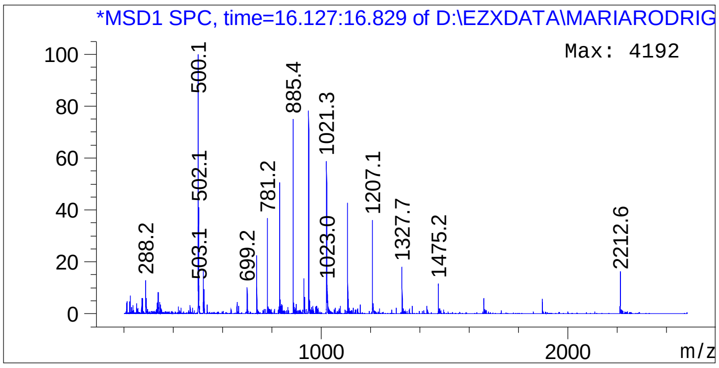

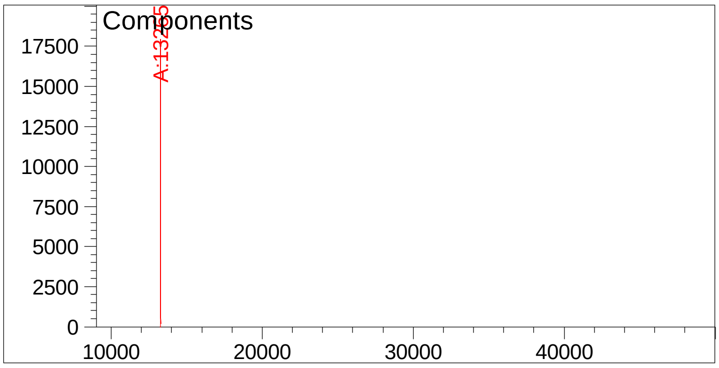

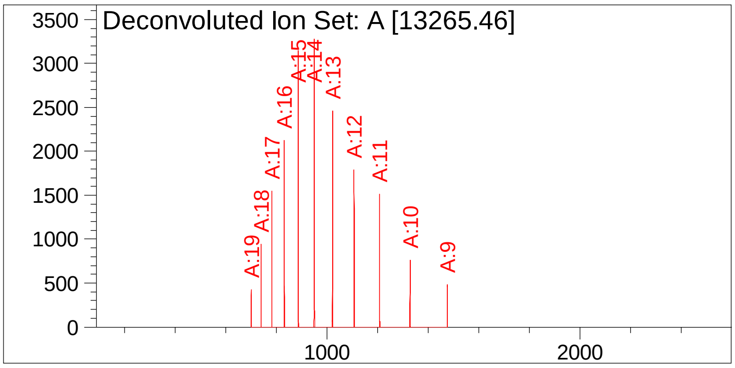

Component	Molecular Weight	Absolute Abundance	Relative Abundance
A	13265.46	17943	100.00

*** End of Report ***

===

Acq. Operator : Maria Rodriguez
Acq. Instrument : INSTRUMENT 1 Location : P1-E-03
Injection Date : 11/27/2024 1:18:44 AM Inj : 1
Inj Volume : 10.000 µl
Acq. Method : C:\CHEM32\1\METHODS\10-75OVER20_PEPTIDE
Last changed : 11/27/2024 1:17:48 AM by Maria Rodriguez
(modified after loading)
Analysis Method : C:\CHEM32\1\METHODS\10-75OVER20_PEPTIDES-5UL-C3.M
Last changed : 12/19/2024 5:56:44 PM by Liam Hales
(modified after loading)
Sample Info : Easy-Access Method: '10-75over20-C3(200+m/Z)'

Additional Info : Peak(s) manually integrated

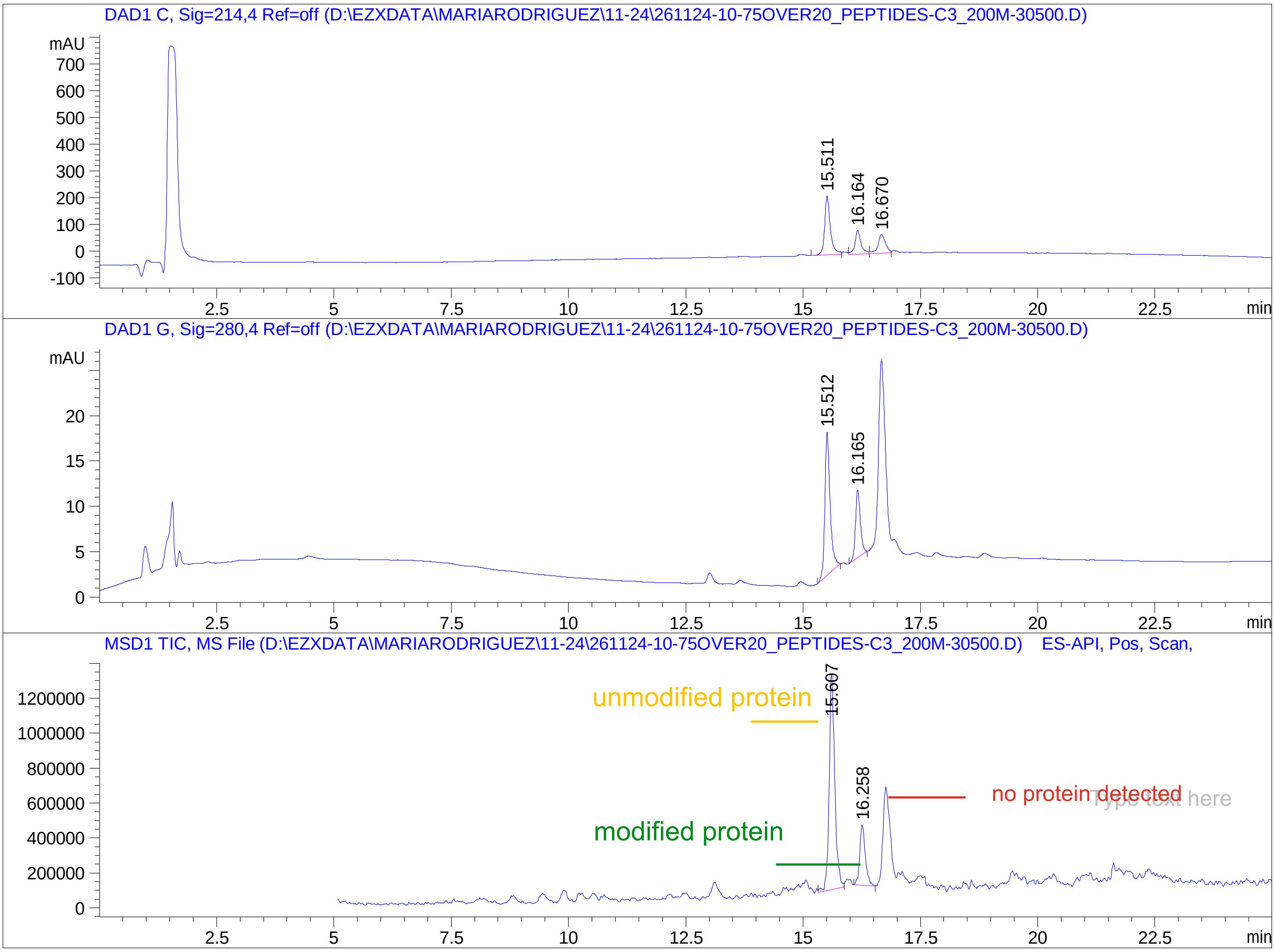

===

Area Percent Report

===

Sorted By : Signal
Multiplier : 1.0000
Dilution : 1.0000
Sample Amount: : 15.00000 [ng/ul] (not used in calc.)
Use Multiplier & Dilution Factor with ISTDs

Signal 1: DAD1 C, Sig=214,4 Ref=off

Peak #	RetTime [min]	Type	Width [min]	Area [mAU*s]	Height [mAU]	Area %
1	15.511	BV	0.1257	1892.53772	221.50458	54.0428
2	16.164	VV	0.1329	809.90894	88.40625	23.1275
3	16.670	VV	0.1729	799.47650	70.15894	22.8296

Totals : 3501.92316 380.06976

Signal 2: DAD1 G, Sig=280,4 Ref=off

Peak #	RetTime [min]	Type	Width [min]	Area [mAU*s]	Height [mAU]	Area %
1	15.512	BB	0.1146	122.89776	15.82265	69.3776
2	16.165	BB	0.1086	54.24543	7.47848	30.6224

Totals : 177.14319 23.30112

Signal 3: MSD1 TIC, MS File

Peak #	RetTime [min]	Type	Width [min]	Area	Height	Area %
1	15.607	VV	0.1302	1.03343e7	1.23746e6	77.9571
2	16.258	BB	0.1305	2.92210e6	3.48869e5	22.0429

Totals : 1.32564e7 1.58633e6

*** End of Report ***

```
Easy-Access Method: '10-75over20-C3(200+m/Z)'
=====================================================================
Acq. Operator   : Maria Rodriguez
Acq. Instrument : INSTRUMENT 1                    Location : P1-E-03
Injection Date  : 11/27/2024 1:18:44 AM                Inj :   1
                                               Inj Volume : 10.000 µl
Acq. Method     : C:\CHEM32\1\METHODS\10-75OVER20_PEPTIDE
Last changed    : 11/27/2024 1:17:48 AM by Maria Rodriguez
                  (modified after loading)
Analysis Method : C:\CHEM32\1\METHODS\10-75OVER20_PEPTIDES-5UL-C3.M
Last changed    : 12/19/2024 5:56:21 PM by Liam Hales
                  (modified after loading)
Sample Info     : Easy-Access Method: '10-75over20-C3(200+m/Z)'


=====================================================================
                      Deconvolution Parameters
=====================================================================

Adduct Ion(Positive): +H, 1.0079 Da
Adduct Ion(Negative):  , 0.0000 Da
Low MW:               9000
DeconvStartChgMaximum Charge:       50
Minimum Peaks in Set: 3
Retain Residual:      No
Ion PWHH:             0.6 Da
MW Agreement:         0.05 %
Noise Cutoff:         1000 counts
Abundance Cutoff:     10 %
MW Assign:            Curve fit
MW Assign Cutoff:     40 %
Envelope Cutoff:      50 %
```

Deconvolution of Spectrum #   1 @ 15.097 - 16.049 min

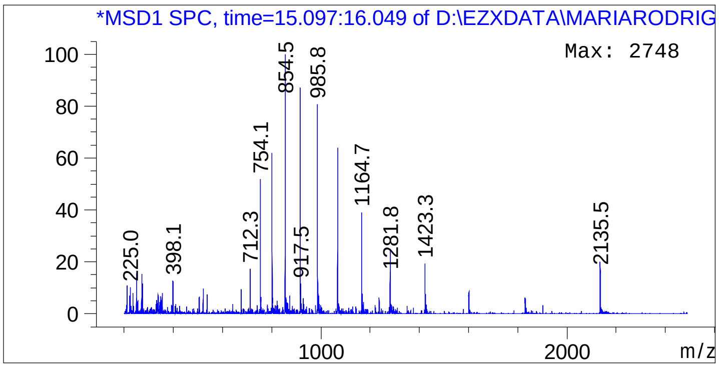

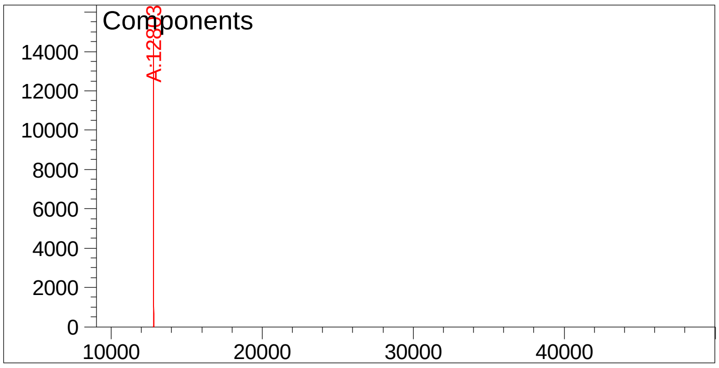

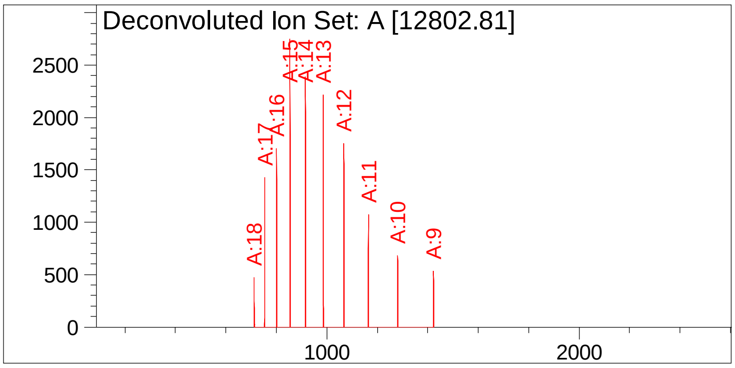

Component	Molecular Weight	Absolute Abundance	Relative Abundance
A	12802.81	14623	100.00

*** End of Report ***

```
Easy-Access Method: '10-75over20-C3(200+m/Z)'
=====================================================================
Acq. Operator   : Maria Rodriguez
Acq. Instrument : INSTRUMENT 1                    Location : P1-E-03
Injection Date  : 11/27/2024 1:18:44 AM                Inj :   1
                                               Inj Volume : 10.000 µl
Acq. Method     : C:\CHEM32\1\METHODS\10-75OVER20_PEPTIDE
Last changed    : 11/27/2024 1:17:48 AM by Maria Rodriguez
                  (modified after loading)
Analysis Method : C:\CHEM32\1\METHODS\10-75OVER20_PEPTIDES-5UL-C3.M
Last changed    : 12/19/2024 5:56:42 PM by Liam Hales
                  (modified after loading)
Sample Info     : Easy-Access Method: '10-75over20-C3(200+m/Z)'


=====================================================================
                      Deconvolution Parameters
=====================================================================

Adduct Ion(Positive): +H, 1.0079 Da
Adduct Ion(Negative):  , 0.0000 Da
Low MW:               9000
DeconvStartChgMaximum Charge:       50
Minimum Peaks in Set: 3
Retain Residual:      No
Ion PWHH:             0.6 Da
MW Agreement:         0.05 %
Noise Cutoff:         1000 counts
Abundance Cutoff:     10 %
MW Assign:            Curve fit
MW Assign Cutoff:     40 %
Envelope Cutoff:      50 %
```

Deconvolution of Spectrum # 1 @ 15.909 - 16.611 min

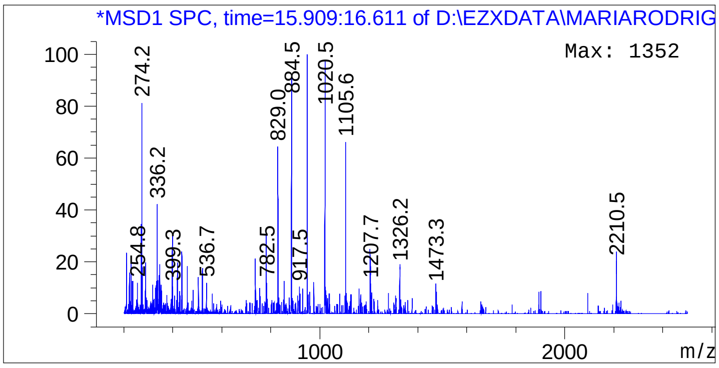

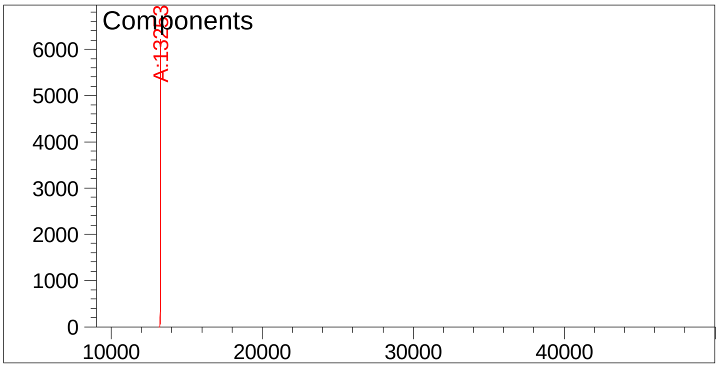

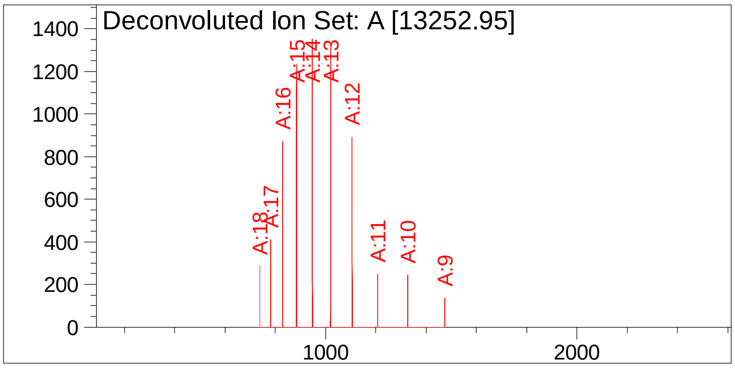

Component	Molecular Weight	Absolute Abundance	Relative Abundance
A	13252.95	6217	100.00

*** End of Report ***

Data File D:\EZXDATA\MARIARODRIGUEZ\11-24\261124-10-75OVER20_PEPTIDES-C3_200M-30497.D
Sample Name: MUT73_MR116_8_26NOV24

===
Acq. Operator : Maria Rodriguez
Acq. Instrument : INSTRUMENT 1 Location : P1-D-09
Injection Date : 11/26/2024 11:48:52 PM Inj : 1
Inj Volume : 10.000 µl
Acq. Method : C:\CHEM32\1\METHODS\10-75OVER20_PEPTIDE
Last changed : 11/26/2024 11:47:56 PM by Maria Rodriguez
(modified after loading)
Analysis Method : C:\CHEM32\1\METHODS\10-75OVER20_PEPTIDES-5UL-C3.M
Last changed : 12/19/2024 5:56:44 PM by Liam Hales
(modified after loading)
Sample Info : Easy-Access Method: '10-75over20-C3(200+m/Z)'

Additional Info : Peak(s) manually integrated

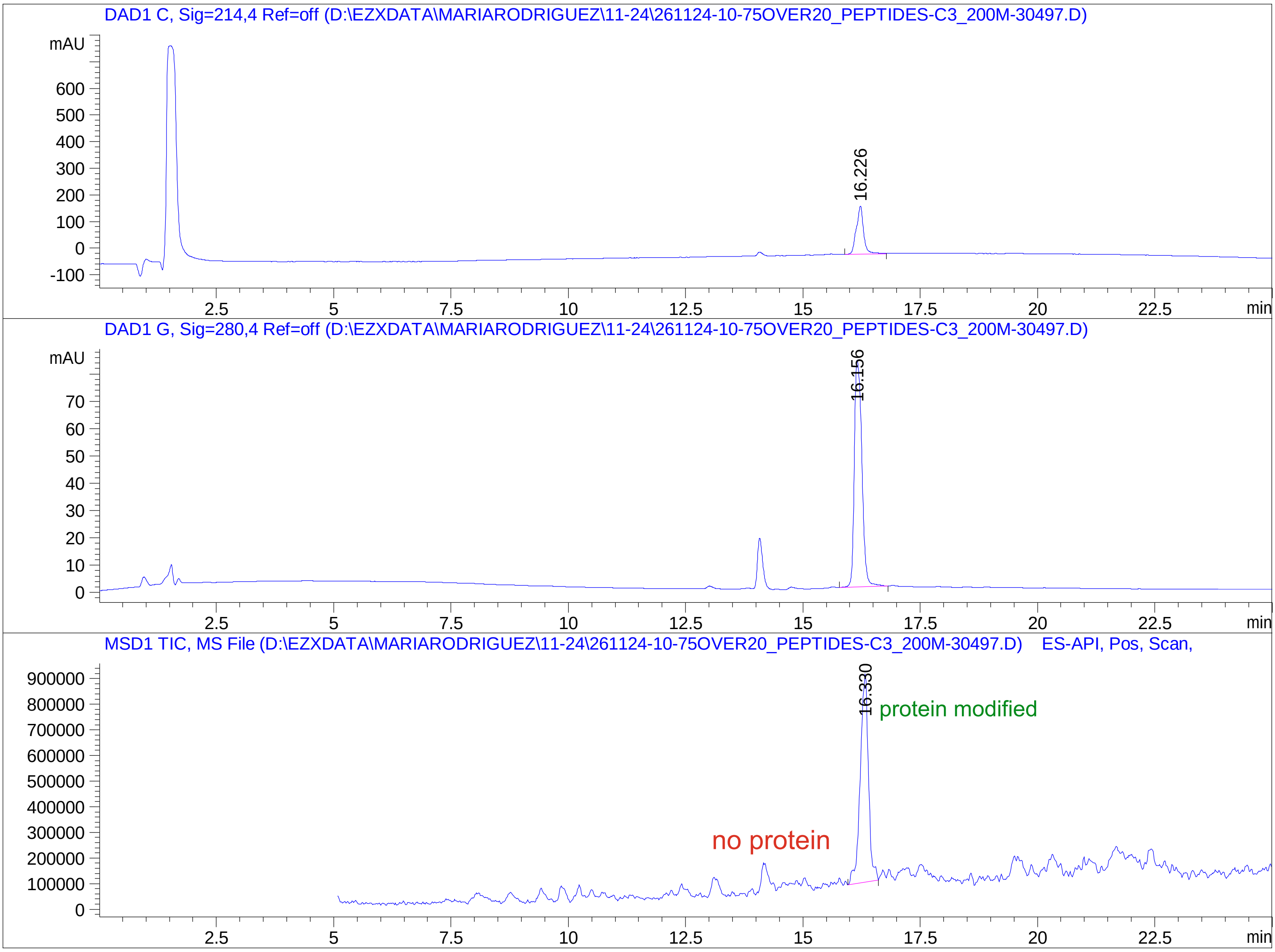

===
Area Percent Report
===

Sorted By : Signal
Multiplier : 1.0000
Dilution : 1.0000
Sample Amount: : 15.00000 [ng/ul] (not used in calc.)
Use Multiplier & Dilution Factor with ISTDs

Signal 1: DAD1 C, Sig=214,4 Ref=off

Peak #	RetTime [min]	Type	Width [min]	Area [mAU*s]	Height [mAU]	Area %
1	16.226	BV	0.1499	1950.40002	180.80363	100.0000

Totals : 1950.40002 180.80363

Signal 2: DAD1 G, Sig=280,4 Ref=off

Peak #	RetTime [min]	Type	Width [min]	Area [mAU*s]	Height [mAU]	Area %
1	16.156	BB	0.1786	929.64935	83.05582	100.0000

Totals : 929.64935 83.05582

Signal 3: MSD1 TIC, MS File

Peak #	RetTime [min]	Type	Width [min]	Area	Height	Area %
1	16.330	BB	0.1749	9.85215e6	8.08898e5	100.0000

Totals : 9.85215e6 8.08898e5

*** End of Report ***

```
Easy-Access Method: '10-75over20-C3(200+m/Z)'
=====================================================================
Acq. Operator   : Maria Rodriguez
Acq. Instrument : INSTRUMENT 1                    Location : P1-D-09
Injection Date  : 11/26/2024 11:48:52 PM               Inj :   1
                                                Inj Volume : 10.000 µl
Acq. Method     : C:\CHEM32\1\METHODS\10-75OVER20_PEPTIDE
Last changed    : 11/26/2024 11:47:56 PM by Maria Rodriguez
                  (modified after loading)
Analysis Method : C:\CHEM32\1\METHODS\10-75OVER20_PEPTIDES-5UL-C3.M
Last changed    : 12/19/2024 5:56:44 PM by Liam Hales
                  (modified after loading)
Sample Info     : Easy-Access Method: '10-75over20-C3(200+m/Z)'

Additional Info : Peak(s) manually integrated

=====================================================================
                      Deconvolution Parameters
=====================================================================

Adduct Ion(Positive): +H, 1.0079 Da
Adduct Ion(Negative):  , 0.0000 Da
Low MW:               9000
DeconvStartChgMaximum Charge:       50
Minimum Peaks in Set: 3
Retain Residual:      No
Ion PWHH:             0.6 Da
MW Agreement:         0.05 %
Noise Cutoff:         1000 counts
Abundance Cutoff:     10 %
MW Assign:            Curve fit
MW Assign Cutoff:     40 %
Envelope Cutoff:      50 %
```

Spectrum Deconvolution
Data File D:\EZXDATA\MARIARODRIGUEZ\11-24\261124-10-75OVER20_PEPTIDES-C3_200M-30497.D
Sample Name: MUT73_MR116_8_26NOV24

Deconvolution of Spectrum # 1 @ 15.877 - 17.094 min

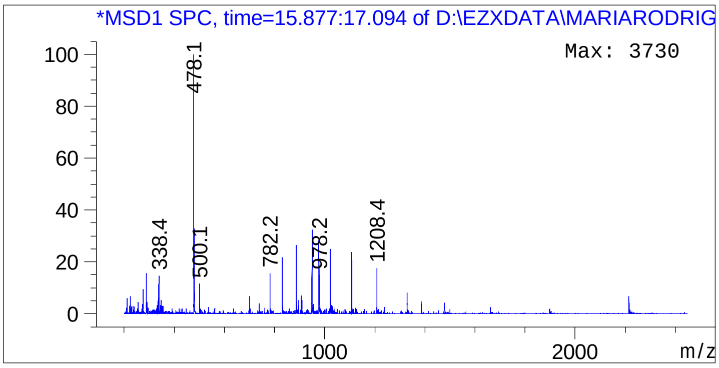

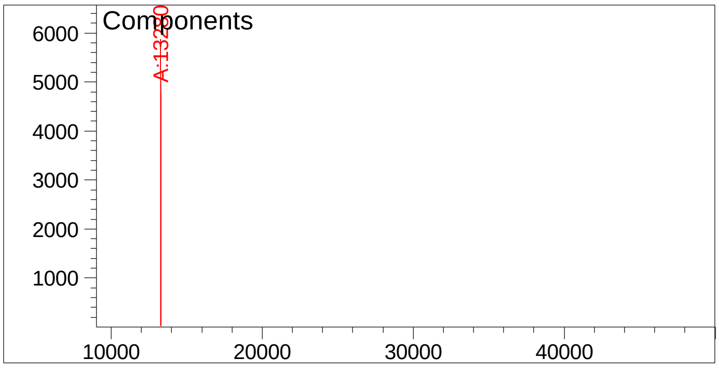

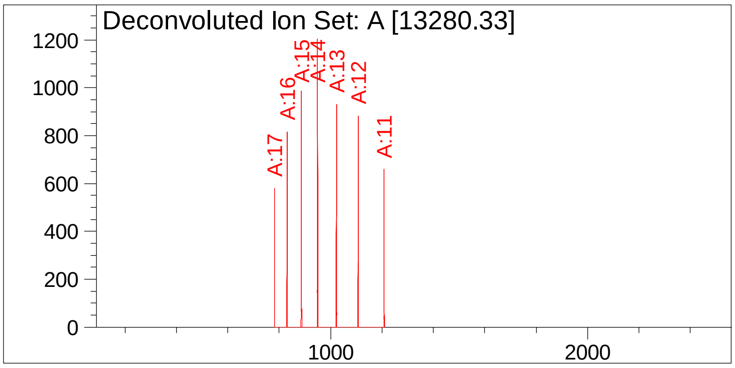

Component	Molecular Weight	Absolute Abundance	Relative Abundance
A	13280.33	5872	100.00

*** End of Report ***

===

Acq. Operator   : Maria Rodriguez
Acq. Instrument : INSTRUMENT 1   Location : P1-A-08
Injection Date : 12/6/2024 9:19:38 PM   Inj :   1
                                              Inj Volume : 10.000 µl
Acq. Method   : C:\CHEM32\1\METHODS\10-75OVER20_PEPTIDE
Last changed   : 12/6/2024 9:18:40 PM by Maria Rodriguez
                  (modified after loading)
Analysis Method : C:\CHEM32\1\METHODS\10-75OVER20_PEPTIDES-5UL-C3.M
Last changed   : 12/19/2024 5:49:25 PM by Liam Hales
                  (modified after loading)
Sample Info   : Easy-Access Method: '10-75over20-C3(200+m/Z)'

Additional Info : Peak(s) manually integrated

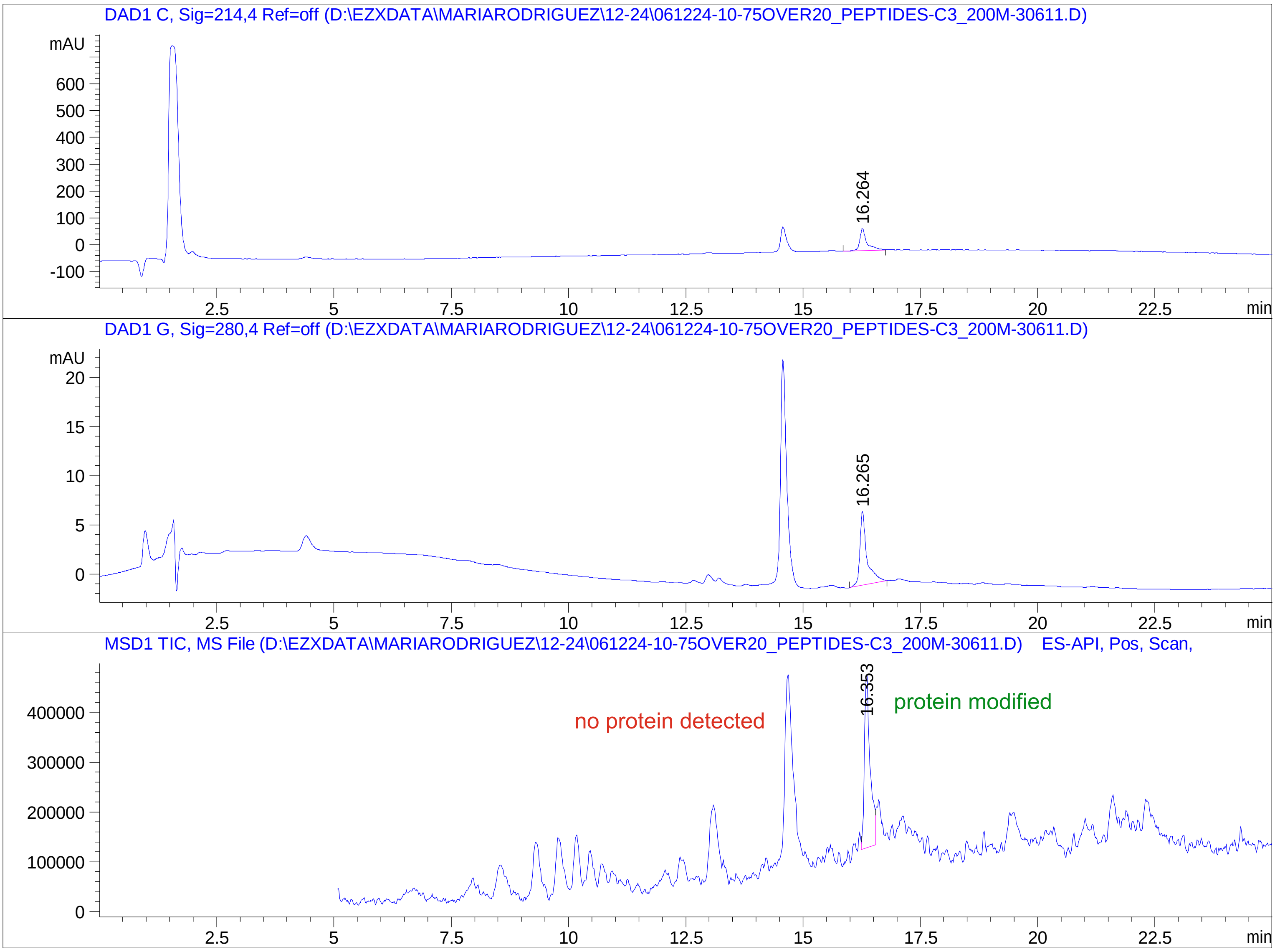

===

Area Percent Report

===

Sorted By   :   Signal
Multiplier   :   1.0000
Dilution   :   1.0000
Sample Amount: :   15.00000   [ng/ul]   (not used in calc.)
Use Multiplier & Dilution Factor with ISTDs

Signal 1: DAD1 C, Sig=214,4 Ref=off

Peak #	RetTime [min]	Type	Width [min]	Area [mAU*s]	Height [mAU]	Area %
1	16.264	BV	0.1530	900.17413	82.71322	100.0000

Totals : 900.17413 82.71322

Signal 2: DAD1 G, Sig=280,4 Ref=off

Peak #	RetTime [min]	Type	Width [min]	Area [mAU*s]	Height [mAU]	Area %
1	16.265	BB	0.1500	78.27454	7.48734	100.0000

Totals : 78.27454 7.48734

Signal 3: MSD1 TIC, MS File

Peak #	RetTime [min]	Type	Width [min]	Area	Height	Area %
1	16.353	VV	0.1212	2.93612e6	3.50417e5	100.0000

Totals : 2.93612e6 3.50417e5

==
*** End of Report ***

Spectrum Deconvolution
Data File D:\EZXDATA\MARIARODRIGUEZ\12-24\061224-10-75OVER20_PEPTIDES-C3_200M-30611.D
Sample Name: MUT73_MR117_14_6Dec2

```
    Easy-Access Method: '10-75over20-C3(200+m/Z)'
    =====================================================================
    Acq. Operator   : Maria Rodriguez
    Acq. Instrument : INSTRUMENT 1                    Location : P1-A-08
    Injection Date  : 12/6/2024 9:19:38 PM                 Inj :   1
                                                    Inj Volume : 10.000 µl
    Acq. Method     : C:\CHEM32\1\METHODS\10-75OVER20_PEPTIDE
    Last changed    : 12/6/2024 9:18:40 PM by Maria Rodriguez
                      (modified after loading)
    Analysis Method : C:\CHEM32\1\METHODS\10-75OVER20_PEPTIDES-5UL-C3.M
    Last changed    : 12/19/2024 5:49:23 PM by Liam Hales
                      (modified after loading)
    Sample Info     : Easy-Access Method: '10-75over20-C3(200+m/Z)'

    Additional Info : Peak(s) manually integrated

    =====================================================================
                          Deconvolution Parameters
    =====================================================================

    Adduct Ion(Positive): +H, 1.0079 Da
    Adduct Ion(Negative):  , 0.0000 Da
    Low MW:               9000
    DeconvStartChgMaximum Charge:       50
    Minimum Peaks in Set: 3
    Retain Residual:      No
    Ion PWHH:             0.6 Da
    MW Agreement:         0.05 %
    Noise Cutoff:         1000 counts
    Abundance Cutoff:     10 %
    MW Assign:            Curve fit
    MW Assign Cutoff:     40 %
    Envelope Cutoff:      50 %
```

Spectrum Deconvolution
Data File D:\EZXDATA\MARIARODRIGUEZ\12-24\061224-10-75OVER20_PEPTIDES-C3_200M-30611.D
Sample Name: MUT73_MR117_14_6Dec2

Deconvolution of Spectrum # 1 @ 16.314 - 16.751 min

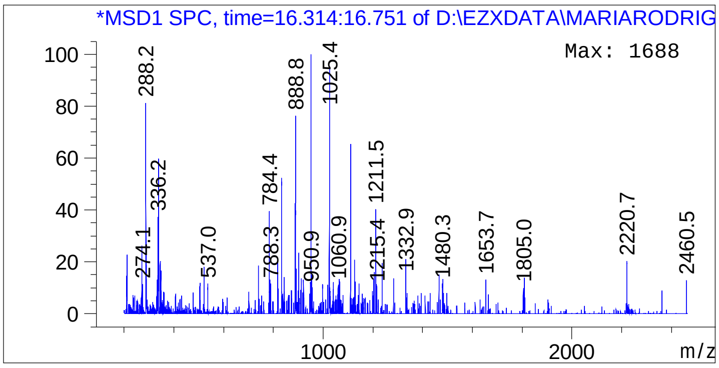

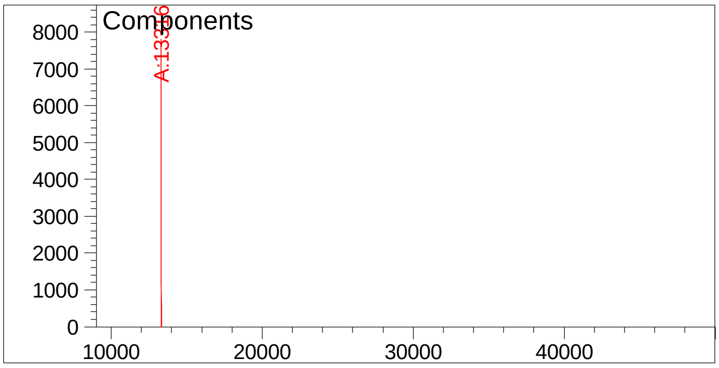

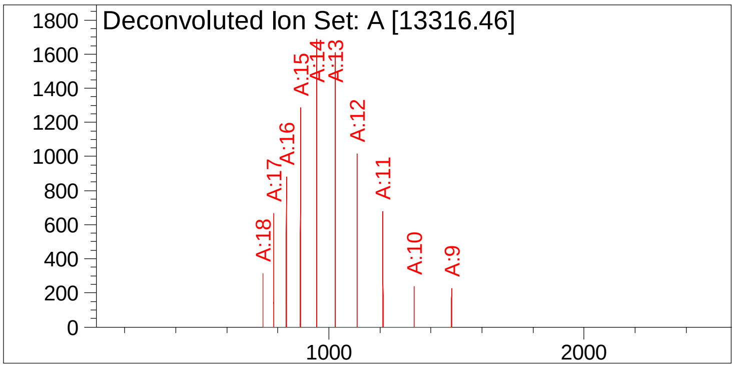

Component	Molecular Weight	Absolute Abundance	Relative Abundance
A	13316.46	7810	100.00

*** End of Report ***

Data File D:\EZXDATA\MARIARODRIGUEZ\11-24\261124-10-75OVER20_PEPTIDES-C3_200M-30502.D
Sample Name: MUT73_MR118_13_26NOV24

===

```
Acq. Operator   : Maria Rodriguez
Acq. Instrument : INSTRUMENT 1                    Location : P1-E-05
Injection Date  : 11/27/2024 2:18:36 AM                Inj :   1
                                               Inj Volume : 10.000 µl
Acq. Method     : C:\CHEM32\1\METHODS\10-75OVER20_PEPTIDE
Last changed    : 11/27/2024 2:17:41 AM by Maria Rodriguez
                  (modified after loading)
Analysis Method : C:\CHEM32\1\METHODS\10-75OVER20_PEPTIDES-5UL-C3.M
Last changed    : 12/19/2024 5:53:04 PM by Liam Hales
                  (modified after loading)
Sample Info     : Easy-Access Method: '10-75over20-C3(200+m/Z)'

Additional Info : Peak(s) manually integrated
```

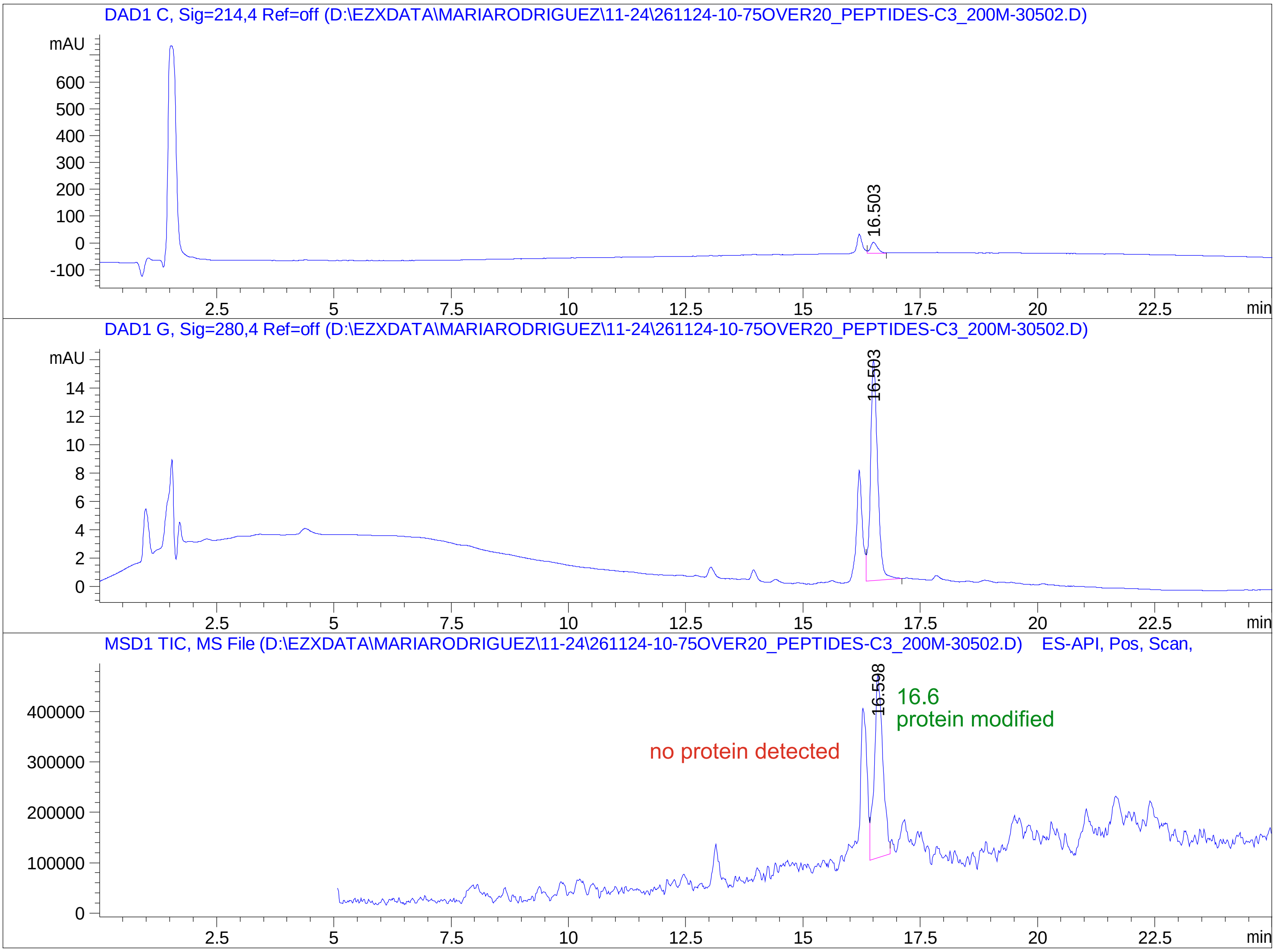

===

Area Percent Report

===

```
Sorted By             :      Signal
Multiplier            :      1.0000
Dilution              :      1.0000
Sample Amount:               :      15.00000   [ng/ul]   (not used in calc.)
Use Multiplier & Dilution Factor with ISTDs
```

Signal 1: DAD1 C, Sig=214,4 Ref=off

Peak #	RetTime [min]	Type	Width [min]	Area [mAU*s]	Height [mAU]	Area %
1	16.503	VV	0.1628	438.02594	41.60106	100.0000

Totals : 438.02594 41.60106

Signal 2: DAD1 G, Sig=280,4 Ref=off

Peak #	RetTime [min]	Type	Width [min]	Area [mAU*s]	Height [mAU]	Area %
1	16.503	VB	0.1590	163.94727	15.55462	100.0000

Totals : 163.94727 15.55462

Signal 3: MSD1 TIC, MS File

Peak #	RetTime [min]	Type	Width [min]	Area	Height	Area %
1	16.598	VV	0.1755	4.59937e6	3.63913e5	100.0000

Totals : 4.59937e6 3.63913e5

==
*** End of Report ***

```
Easy-Access Method: '10-75over20-C3(200+m/Z)'
=====================================================================
Acq. Operator   : Maria Rodriguez
Acq. Instrument : INSTRUMENT 1                    Location : P1-E-05
Injection Date  : 11/27/2024 2:18:36 AM                Inj :   1
                                                Inj Volume : 10.000 µl
Acq. Method     : C:\CHEM32\1\METHODS\10-75OVER20_PEPTIDE
Last changed    : 11/27/2024 2:17:41 AM by Maria Rodriguez
                  (modified after loading)
Analysis Method : C:\CHEM32\1\METHODS\10-75OVER20_PEPTIDES-5UL-C3.M
Last changed    : 12/19/2024 5:53:01 PM by Liam Hales
                  (modified after loading)
Sample Info     : Easy-Access Method: '10-75over20-C3(200+m/Z)'

Additional Info : Peak(s) manually integrated

=====================================================================
                      Deconvolution Parameters
=====================================================================

Adduct Ion(Positive): +H, 1.0079 Da
Adduct Ion(Negative):  , 0.0000 Da
Low MW:               9000
DeconvStartChgMaximum Charge:       50
Minimum Peaks in Set: 3
Retain Residual:      No
Ion PWHH:             0.6 Da
MW Agreement:         0.05 %
Noise Cutoff:         1000 counts
Abundance Cutoff:     10 %
MW Assign:            Curve fit
MW Assign Cutoff:     40 %
Envelope Cutoff:      50 %
```

Deconvolution of Spectrum # 1 @ 16.096 - 16.704 min

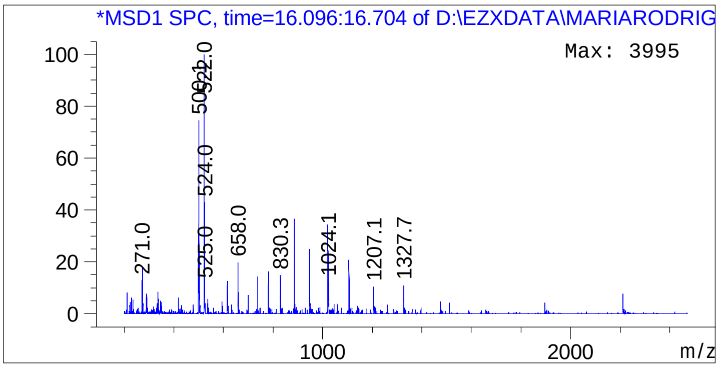

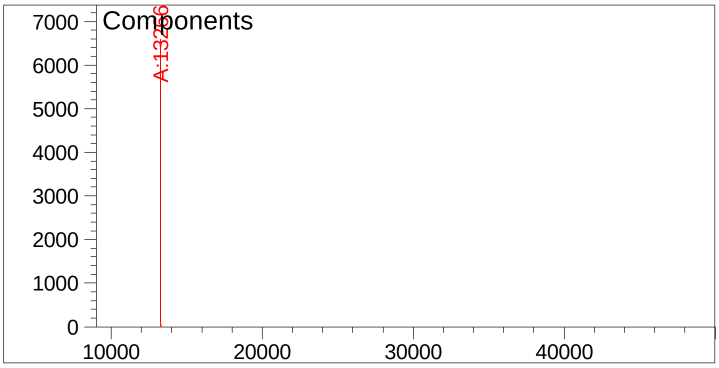

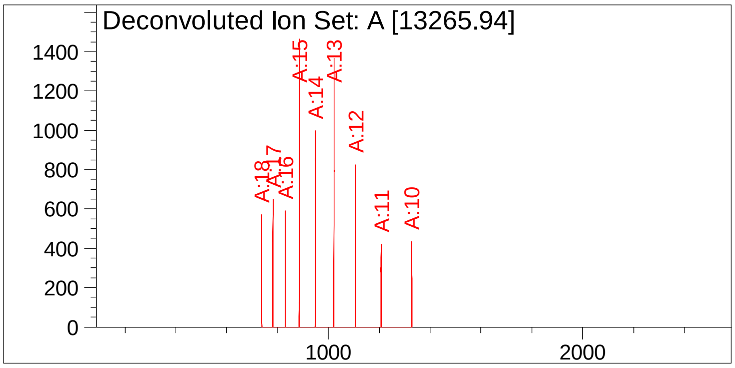

Component	Molecular Weight	Absolute Abundance	Relative Abundance
A	13265.94	6591	100.00

*** End of Report ***

===

```
Acq. Operator   : Maria Rodriguez
Acq. Instrument : INSTRUMENT 1                     Location : P1-E-07
Injection Date  : 11/27/2024 3:18:30 AM                 Inj :   1
                                                 Inj Volume : 10.000 µl
Acq. Method     : C:\CHEM32\1\METHODS\10-75OVER20_PEPTIDE
Last changed    : 11/27/2024 3:17:34 AM by Maria Rodriguez
                  (modified after loading)
Analysis Method : C:\CHEM32\1\METHODS\10-75OVER20_PEPTIDES-5UL-C3.M
Last changed    : 12/19/2024 5:51:14 PM by Liam Hales
                  (modified after loading)
Sample Info     : Easy-Access Method: '10-75over20-C3(200+m/Z)'

Additional Info : Peak(s) manually integrated
```

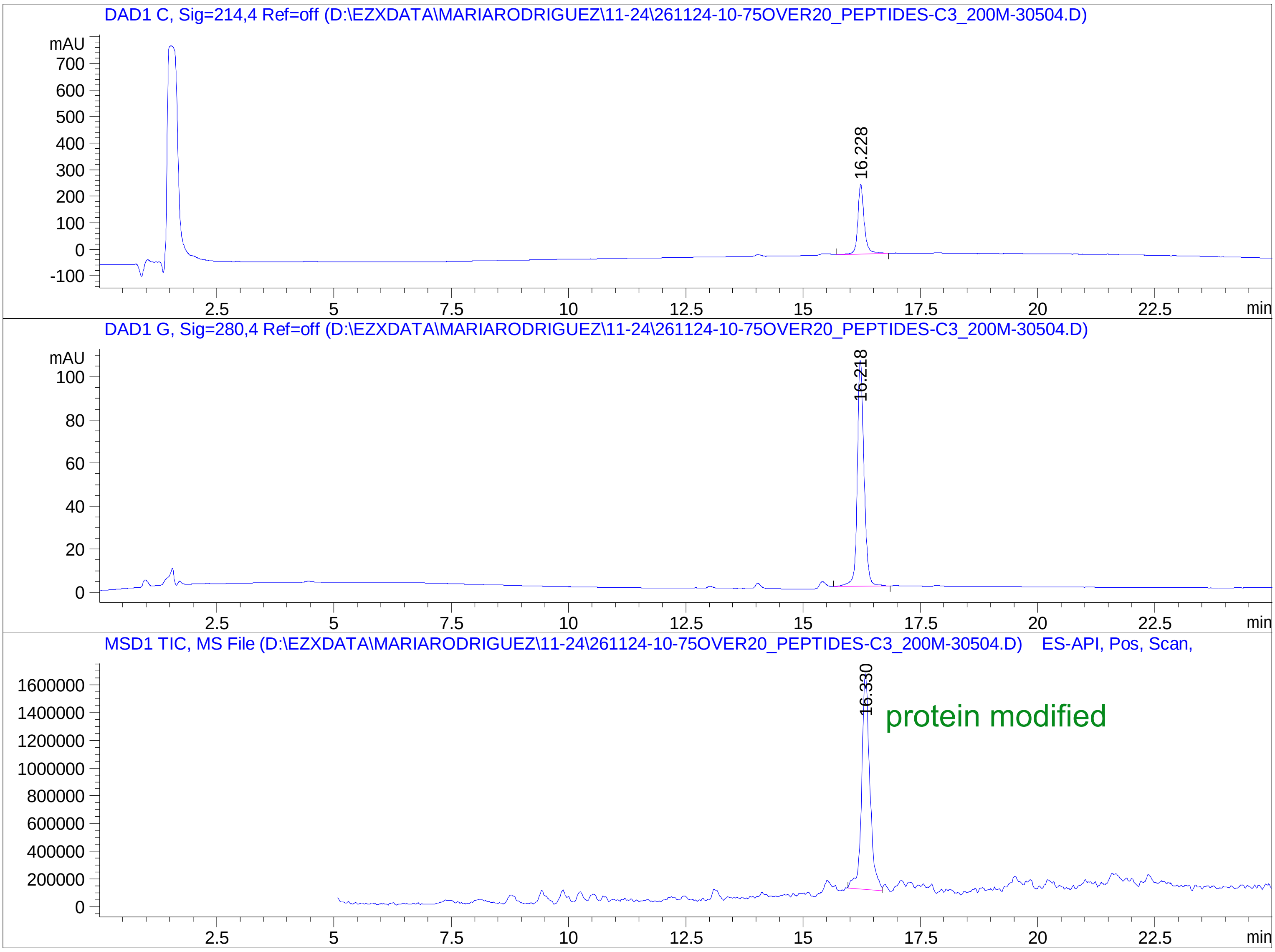

===

Area Percent Report

===

```
Sorted By             :      Signal
Multiplier            :      1.0000
Dilution              :      1.0000
Sample Amount:               :      15.00000  [ng/ul]   (not used in calc.)
Use Multiplier & Dilution Factor with ISTDs
```

Signal 1: DAD1 C, Sig=214,4 Ref=off

Peak #	RetTime [min]	Type	Width [min]	Area [mAU*s]	Height [mAU]	Area %
1	16.228	VV	0.1419	2519.33081	262.60269	100.0000

Totals : 2519.33081 262.60269

Signal 2: DAD1 G, Sig=280,4 Ref=off

Peak #	RetTime [min]	Type	Width [min]	Area [mAU*s]	Height [mAU]	Area %
1	16.218	BB	0.1492	1036.96008	105.00607	100.0000

Totals : 1036.96008 105.00607

Signal 3: MSD1 TIC, MS File

Peak #	RetTime [min]	Type	Width [min]	Area	Height	Area %
1	16.330	BV	0.1790	1.87525e7	1.54508e6	100.0000

Totals : 1.87525e7 1.54508e6

*** End of Report ***

```
Easy-Access Method: '10-75over20-C3(200+m/Z)'
=====================================================================
Acq. Operator   : Maria Rodriguez
Acq. Instrument : INSTRUMENT 1                    Location : P1-E-07
Injection Date  : 11/27/2024 3:18:30 AM                Inj :   1
                                               Inj Volume : 10.000 µl
Acq. Method     : C:\CHEM32\1\METHODS\10-75OVER20_PEPTIDE
Last changed    : 11/27/2024 3:17:34 AM by Maria Rodriguez
                  (modified after loading)
Analysis Method : C:\CHEM32\1\METHODS\10-75OVER20_PEPTIDES-5UL-C3.M
Last changed    : 12/19/2024 5:51:11 PM by Liam Hales
                  (modified after loading)
Sample Info     : Easy-Access Method: '10-75over20-C3(200+m/Z)'

Additional Info : Peak(s) manually integrated

=====================================================================
                      Deconvolution Parameters
=====================================================================

Adduct Ion(Positive): +H, 1.0079 Da
Adduct Ion(Negative):  , 0.0000 Da
Low MW:               9000
DeconvStartChgMaximum Charge:       50
Minimum Peaks in Set: 3
Retain Residual:      No
Ion PWHH:             0.6 Da
MW Agreement:         0.05 %
Noise Cutoff:         1000 counts
Abundance Cutoff:     10 %
MW Assign:            Curve fit
MW Assign Cutoff:     40 %
Envelope Cutoff:      50 %
```

Spectrum Deconvolution
Data File D:\EZXDATA\MARIARODRIGUEZ\11-24\261124-10-75OVER20_PEPTIDES-C3_200M-30504.D
Sample Name: MUT73_MR119_15_26NOV24

Deconvolution of Spectrum #   1 @ 15.612 - 17.266 min

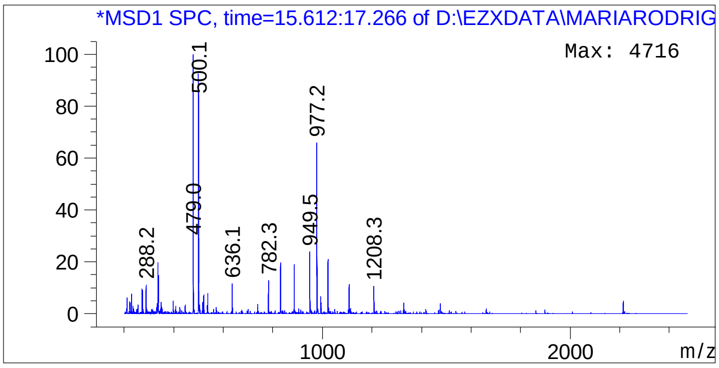

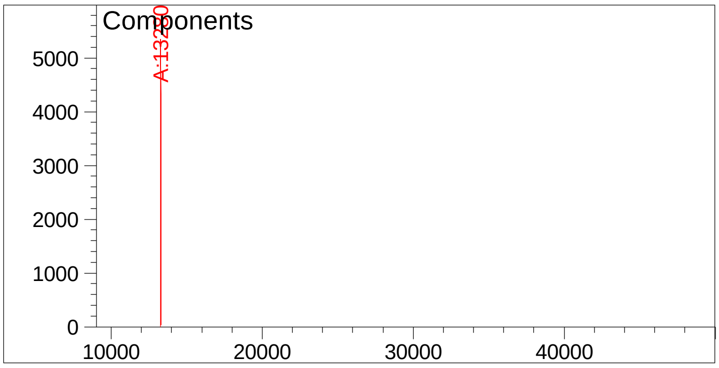

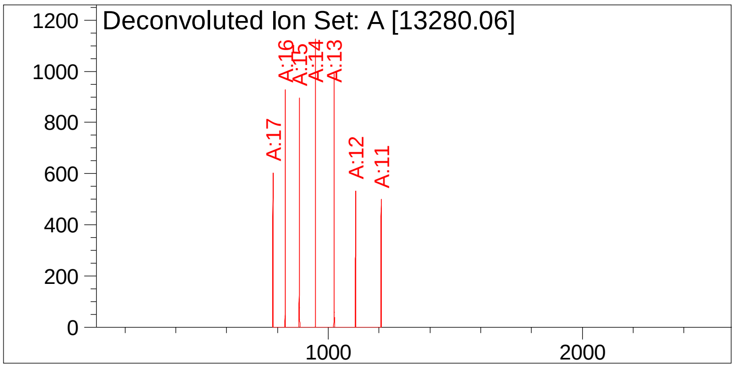

Component	Molecular Weight	Absolute Abundance	Relative Abundance
A	13280.06	5349	100.00

*** End of Report ***

Data File D:\EZXDATA\MARIARODRIGUEZ\12-24\061224-10-75OVER20_PEPTIDES-C3_200M-30610.D
Sample Name: MUT73_MR121_7_6Dec24

===

Acq. Operator : Maria Rodriguez
Acq. Instrument : INSTRUMENT 1 Location : P1-A-07
Injection Date : 12/6/2024 8:49:40 PM Inj : 1
Inj Volume : 10.000 µl
Acq. Method : C:\CHEM32\1\METHODS\10-75OVER20_PEPTIDE
Last changed : 12/6/2024 8:48:42 PM by Maria Rodriguez
(modified after loading)
Analysis Method : C:\CHEM32\1\METHODS\10-75OVER20_PEPTIDES-5UL-C3.M
Last changed : 12/19/2024 5:47:40 PM by Liam Hales
(modified after loading)
Sample Info : Easy-Access Method: '10-75over20-C3(200+m/Z)'

Additional Info : Peak(s) manually integrated

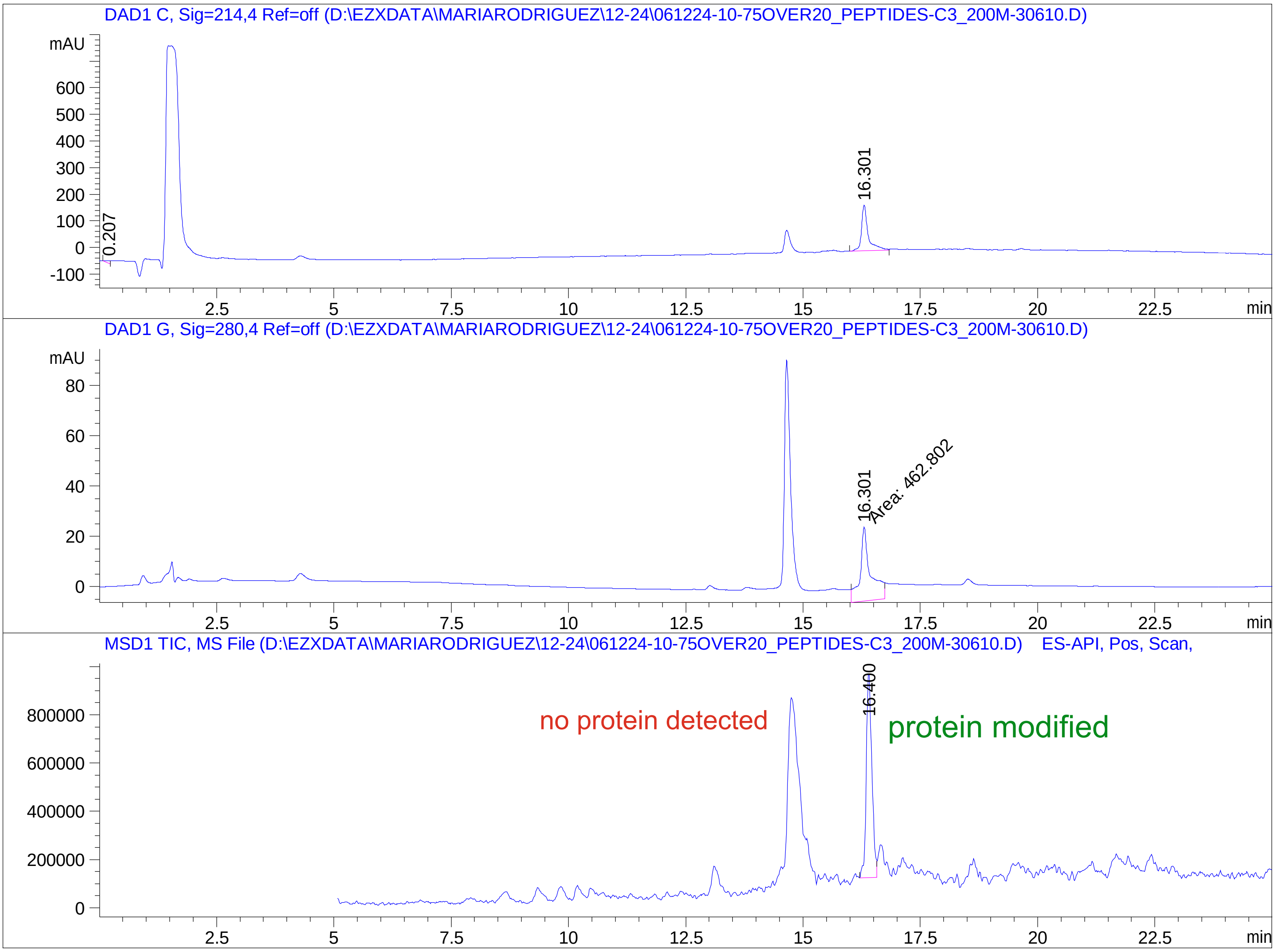

===
Area Percent Report
===

Sorted By : Signal
Multiplier : 1.0000
Dilution : 1.0000
Sample Amount: : 15.00000 [ng/ul] (not used in calc.)
Use Multiplier & Dilution Factor with ISTDs

Signal 1: DAD1 C, Sig=214,4 Ref=off

Peak #	RetTime [min]	Type	Width [min]	Area [mAU*s]	Height [mAU]	Area %
1	0.207	BV	0.0783	64.63530	11.38142	3.6673
2	16.301	BV	0.1439	1697.85669	170.90038	96.3327
Totals :				1762.49199	182.28180	

Signal 2: DAD1 G, Sig=280,4 Ref=off

Peak #	RetTime [min]	Type	Width [min]	Area [mAU*s]	Height [mAU]	Area %
1	16.301	MM	0.2626	462.80154	29.36919	100.0000
Totals :				462.80154	29.36919	

Signal 3: MSD1 TIC, MS File

Peak #	RetTime [min]	Type	Width [min]	Area	Height	Area %
1	16.400	BV	0.1242	7.01378e6	8.50936e5	100.0000
Totals :				7.01378e6	8.50936e5	

===

*** End of Report ***

Spectrum Deconvolution
Data File D:\EZXDATA\MARIARODRIGUEZ\12-24\061224-10-75OVER20_PEPTIDES-C3_200M-30610.D
Sample Name: MUT73_MR121_7_6Dec24

```
Easy-Access Method: '10-75over20-C3(200+m/Z)'
=====================================================================
Acq. Operator   : Maria Rodriguez
Acq. Instrument : INSTRUMENT 1                     Location : P1-A-07
Injection Date  : 12/6/2024 8:49:40 PM                  Inj :   1
                                                Inj Volume : 10.000 µl
Acq. Method     : C:\CHEM32\1\METHODS\10-75OVER20_PEPTIDE
Last changed    : 12/6/2024 8:48:42 PM by Maria Rodriguez
                  (modified after loading)
Analysis Method : C:\CHEM32\1\METHODS\10-75OVER20_PEPTIDES-5UL-C3.M
Last changed    : 12/19/2024 5:47:38 PM by Liam Hales
                  (modified after loading)
Sample Info     : Easy-Access Method: '10-75over20-C3(200+m/Z)'

Additional Info : Peak(s) manually integrated

=====================================================================
                      Deconvolution Parameters
=====================================================================

Adduct Ion(Positive): +H, 1.0079 Da
Adduct Ion(Negative):  , 0.0000 Da
Low MW:               9000
DeconvStartChgMaximum Charge:       50
Minimum Peaks in Set: 3
Retain Residual:      No
Ion PWHH:             0.6 Da
MW Agreement:         0.05 %
Noise Cutoff:         1000 counts
Abundance Cutoff:     10 %
MW Assign:            Curve fit
MW Assign Cutoff:     40 %
Envelope Cutoff:      50 %
```

Spectrum Deconvolution
Data File D:\EZXDATA\MARIARODRIGUEZ\12-24\061224-10-75OVER20_PEPTIDES-C3_200M-30610.D
Sample Name: MUT73_MR121_7_6Dec24

Deconvolution of Spectrum # 1 @ 15.955 - 17.079 min

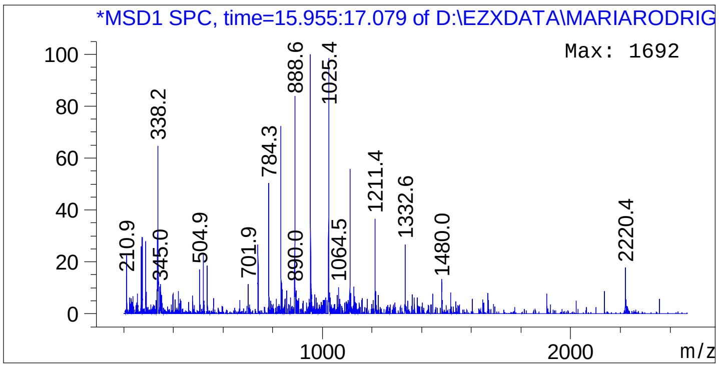

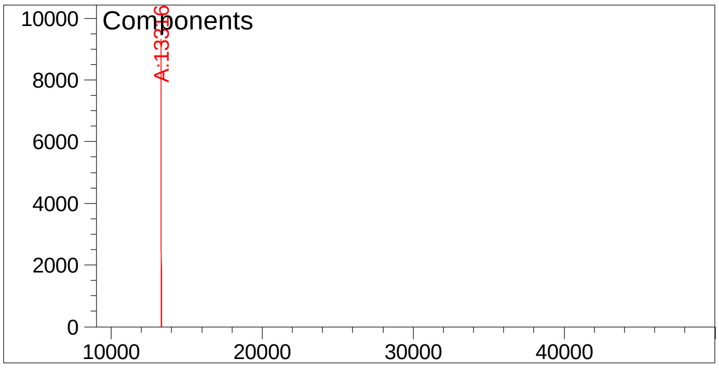

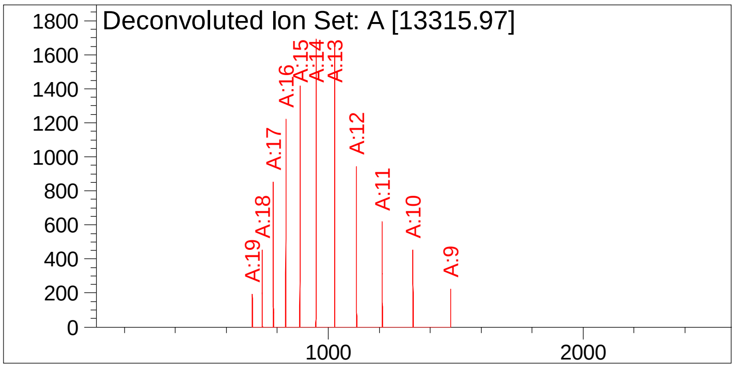

Component	Molecular Weight	Absolute Abundance	Relative Abundance
A	13315.97	9318	100.00

*** End of Report ***

Single Injection Report

**Data file:**	100125-10-75OVER20_PEPTIDES-C3_200M-30685_017.D		
**Sequence Name:**	Chemstatioimports_MRR	**Project Name:**	Walkup Submissions
**Sample name:**	BromoTag_DMSO_10Jan25	**Operator:**	Maria Rodriguez
**Instrument:**		**Injection date:**	2025-01-10 17:33:50-08:00
**Inj. volume:**		**Location:**	P2-A-08
**Acq. method:**	10-75OVER20_PEPTIDES-C3_200MZ.M	**Type:**	Sample
**Processing method:**	*Deconvolution Test 2.pmx	**Sample amount:**	
**Manually modified:**	None		

Data Analysis Method: **Deconvolution Test 2.pmx**
Path: **D:\CDSProjects\Walkup Submissions\Results\Chemstatioimports_MRR.rslt**
Method parameters are filtered - only a subset is displayed

2 Method Parameters

2.11 MS Spectral Deconvolution Parameters

**Run automatic deconvolution:**	Yes	**Use RT window:**	No	**TIC peak type:**	All peaks
**TIC peak threshold:**	Top (n) peaks	**Top (n) peaks:**	6	**Positive adduct:**	+H
**Negative adduct:**	-H	**Use m/z range:**	No	**Low molecular weight:**	4000
**High molecular weight:**	25000	**Maximum charge:**	40	**Minimum peaks in set:**	3
**Show unmatched peaks:**	No	**MW agreement (0.01%):**	5	**Absolute noise threshold:**	1000
**Relative abundance threshold (%):**	10	**MW algorithm:**	Curve Fit	**MW algorithm threshold (%):**	40
**Envelope threshold (%):**	50				

Method Audit Trail

Method audit trail is not printed

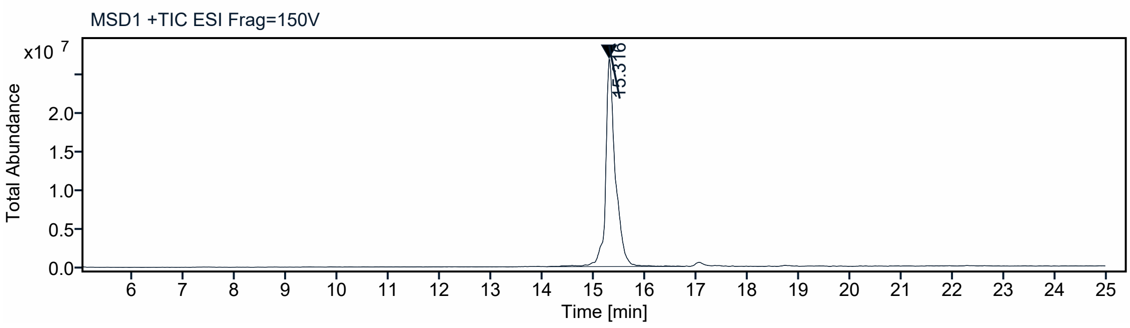

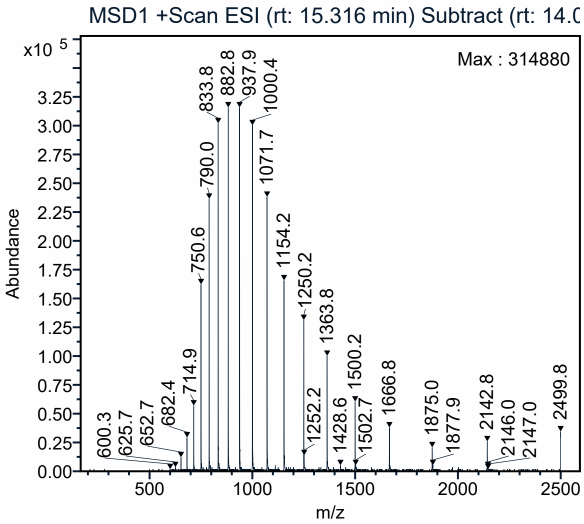

Deconvolution of peak at RT: 15.316

Signal: MSD1 +TIC ESI Frag=150V

Spectrum: MSD1 +Scan ESI (rt: 15.316 min) Subtract (rt: 14.099 min)

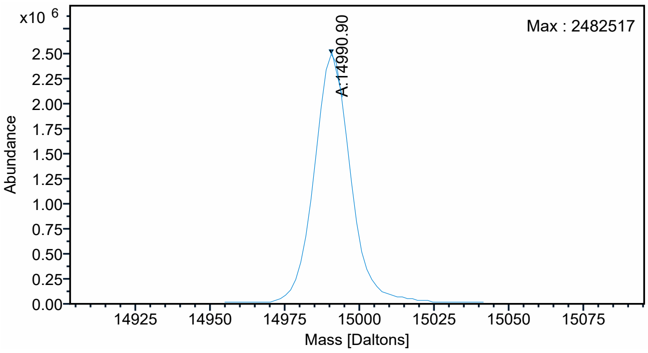

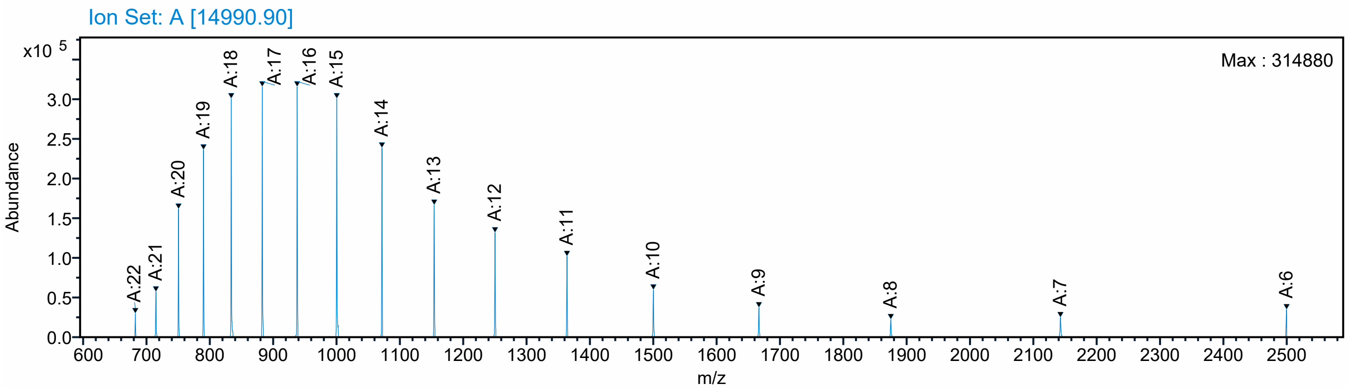

Component	Mass	Absolute Abundance	Relative Abundance (%)	Relative Quantitation (%)
A	14990.90	2482517	100.00	100.00

Time [min]

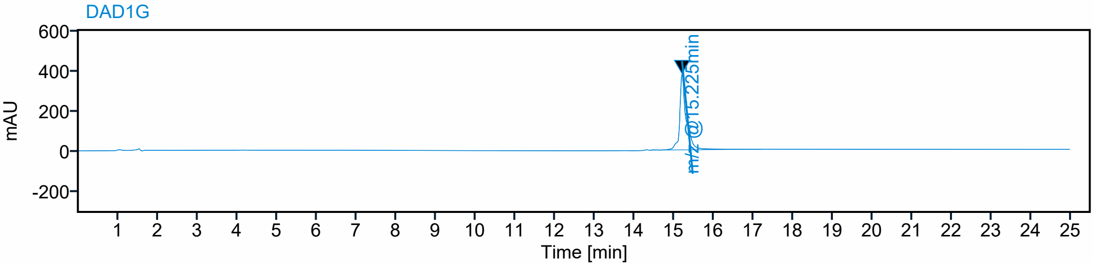

Signal: DAD1G

Name	RT [min]	RF	Area	Peak Area Percent	Group
	15.225		4451.4894	100.00	unmodified protein - 100%

Signal: MSD1 +TIC ESI Frag=150V

Name	RT [min]	RF	Area	Peak Area Percent	Group
	15.316		355667162.6850	100.00	unmodified protein - 100%

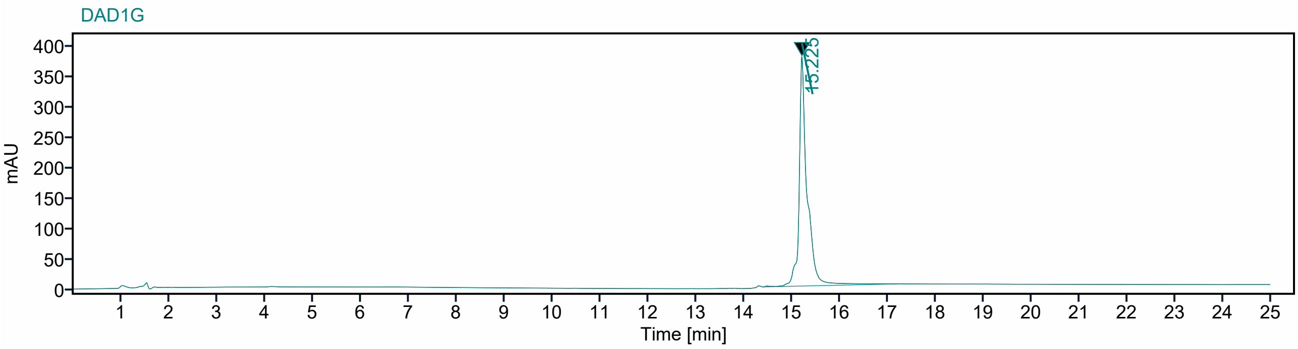
DAD1G
400
350
300
250
200
150
100
50
0
mAU
15.225
1
2
3
4
5
6
7
8
9
10
11
12
13
14
15
16
17
18
19
20
21
22
23
24
25
Time [min]

Single Injection Report

**Data file:**	100125-10-75OVER20_PEPTIDES-C3_200M-30686_018.D		
**Sequence Name:**	Chemstatioimports_MRR	**Project Name:**	Walkup Submissions
**Sample name:**	BromoTag_etjq1_10Jan25	**Operator:**	Maria Rodriguez
**Instrument:**		**Injection date:**	2025-01-10 18:03:42-08:00
**Inj. volume:**		**Location:**	P2-A-09
**Acq. method:**	10-75OVER20_PEPTIDES-C3_200MZ.M	**Type:**	Sample
**Processing method:**	*Deconvolution Test 2.pmx	**Sample amount:**	
**Manually modified:**	None		

Data Analysis Method: Deconvolution Test 2.pmx
Path: D:\CDSProjects\Walkup Submissions\Results\Chemstatioimports_MRR.rslt
Method parameters are filtered - only a subset is displayed

2 Method Parameters

2.11 MS Spectral Deconvolution Parameters

**Run automatic deconvolution:**	Yes	**Use RT window:**	No	**TIC peak type:**	All peaks
**TIC peak threshold:**	Top (n) peaks	**Top (n) peaks:**	6	**Positive adduct:**	+H
**Negative adduct:**	-H	**Use m/z range:**	No	**Low molecular weight:**	4000
**High molecular weight:**	25000	**Maximum charge:**	40	**Minimum peaks in set:**	3
**Show unmatched peaks:**	No	**MW agreement (0.01%):**	5	**Absolute noise threshold:**	1000
**Relative abundance threshold (%):**	10	**MW algorithm:**	Curve Fit	**MW algorithm threshold (%):**	40
**Envelope threshold (%):**	50				

Method Audit Trail

Method audit trail is not printed

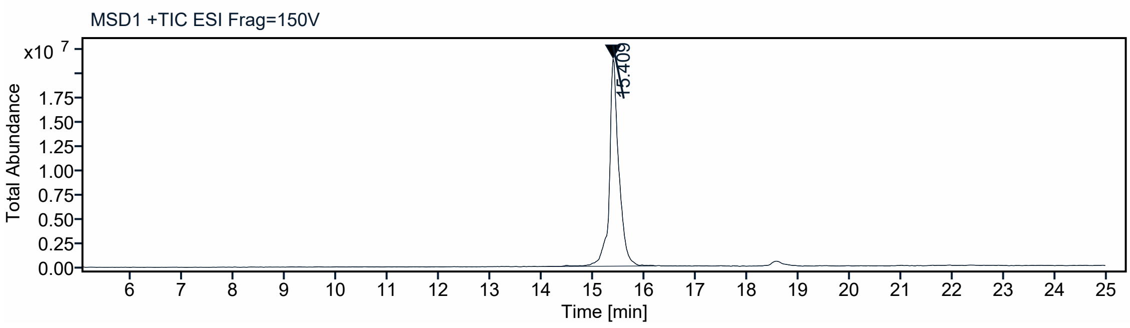

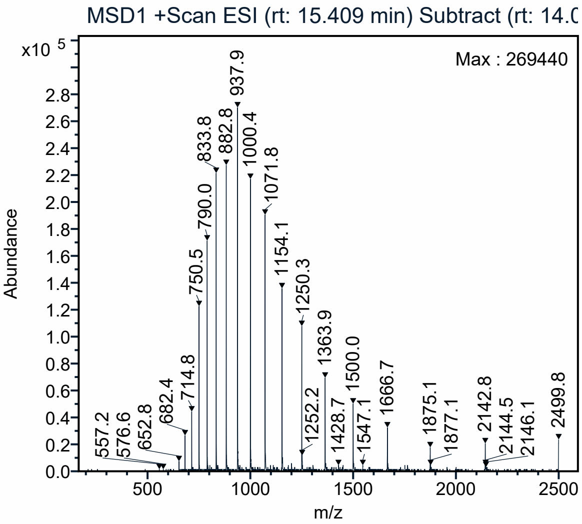

Deconvolution of peak at RT: 15.409

Signal: MSD1 +TIC ESI Frag=150V

Spectrum: MSD1 +Scan ESI (rt: 15.409 min) Subtract (rt: 14.083 min)

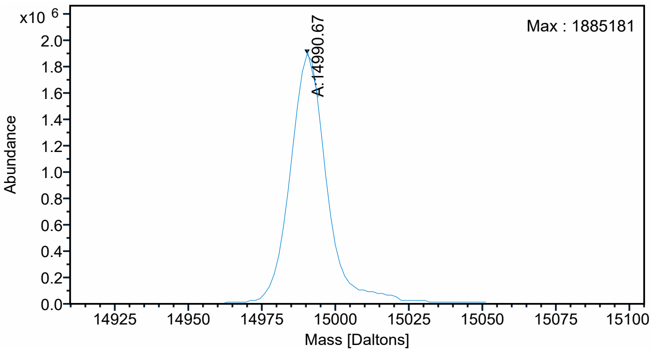

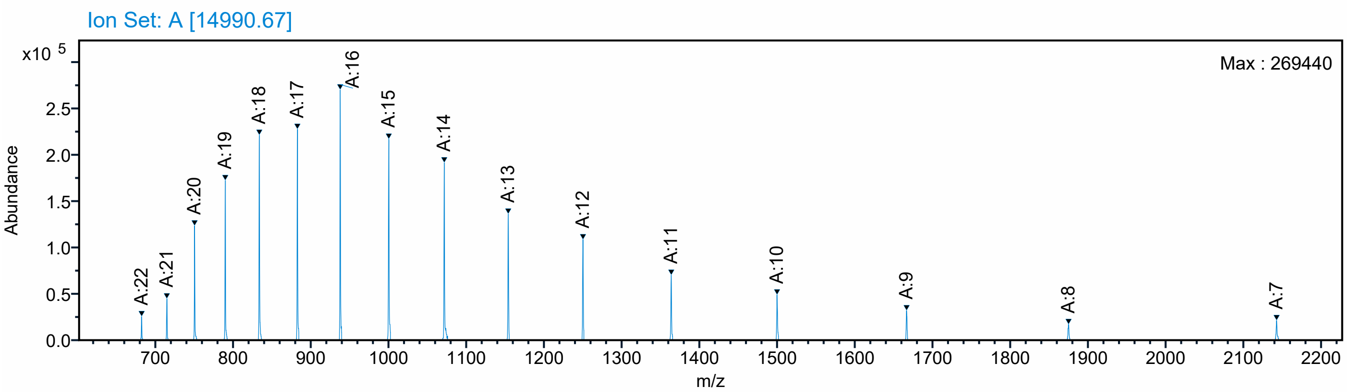

Component	Mass	Absolute Abundance	Relative Abundance (%)	Relative Quantitation (%)
A	14990.67	1885181	100.00	100.00

Time [min]

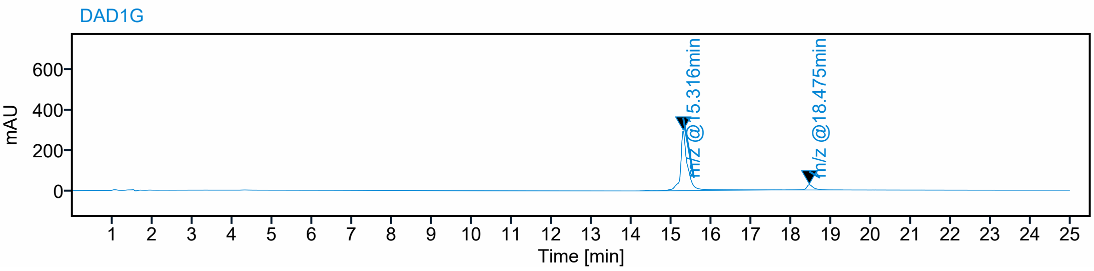

Signal: DAD1G

Name	RT [min]	RF	Area	Peak Area Percent	Group
	15.316		3736.5397	92.02	UNMODIFIED PROTEIN - 100%
	~~18.475~~		~~323.9702~~	~~7.98~~	no protein detected in MS

Signal: MSD1 +TIC ESI Frag=150V

Name	RT [min]	RF	Area	Peak Area Percent	Group
	15.409		274968182.8215	100.00	UNMODIFIED PROTEIN - 100%

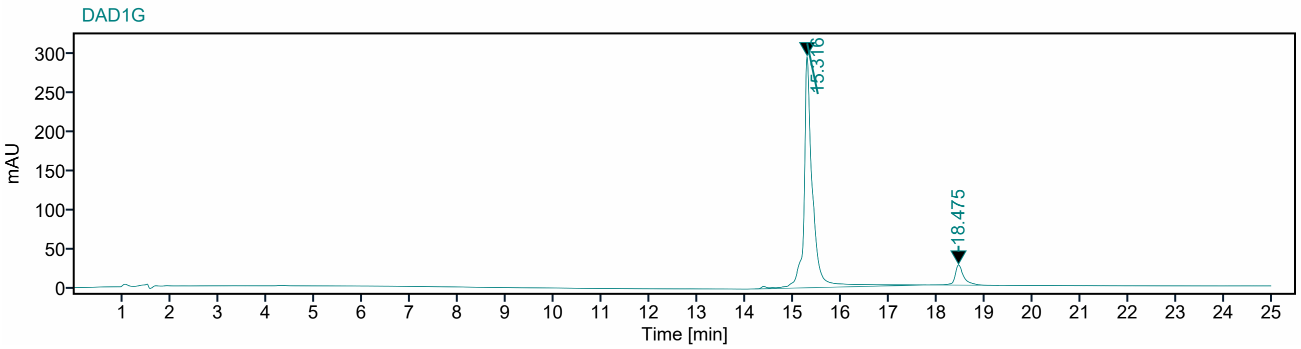
DAD1G
mAU
300
250
200
150
100
50
0
15.316
18.475
1
2
3
4
5
6
7
8
9
10
11
12
13
14
15
16
17
18
19
20
21
22
23
24
25
Time [min]

**Data file:**	100125-10-75OVER20_PEPTIDES-C3_200M-30687_019.D		
**Sequence Name:**	Chemstatioimports_MRR	**Project Name:**	Walkup Submissions
**Sample name:**	BromoTag_mr100_10Jan25	**Operator:**	Maria Rodriguez
**Instrument:**		**Injection date:**	2025-01-10 18:33:34-08:00
**Inj. volume:**		**Location:**	P2-B-01
**Acq. method:**	10-75OVER20_PEPTIDES-C3_200MZ.M	**Type:**	Sample
**Processing method:**	*Deconvolution Test 2.pmx	**Sample amount:**	
**Manually modified:**	None		

Data Analysis Method: Deconvolution Test 2.pmx
Path: D:\CDSProjects\Walkup Submissions\Results\Chemstatioimports_MRR.rslt
Method parameters are filtered - only a subset is displayed

2 Method Parameters

2.11 MS Spectral Deconvolution Parameters

**Run automatic deconvolution:**	Yes	**Use RT window:**	No	**TIC peak type:**	All peaks
**TIC peak threshold:**	Top (n) peaks	**Top (n) peaks:**	6	**Positive adduct:**	+H
**Negative adduct:**	-H	**Use m/z range:**	No	**Low molecular weight:**	4000
**High molecular weight:**	25000	**Maximum charge:**	40	**Minimum peaks in set:**	3
**Show unmatched peaks:**	No	**MW agreement (0.01%):**	5	**Absolute noise threshold:**	1000
**Relative abundance threshold (%):**	10	**MW algorithm:**	Curve Fit	**MW algorithm threshold (%):**	40
**Envelope threshold (%):**	50				

Method Audit Trail

Method audit trail is not printed

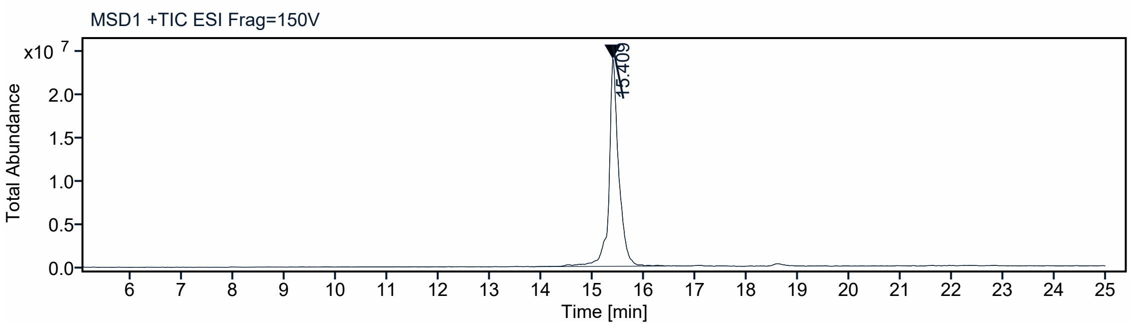

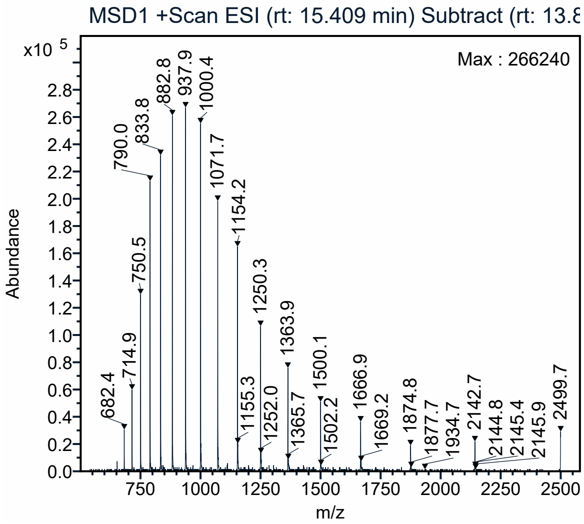

Deconvolution of peak at RT: 15.409

Signal: MSD1 +TIC ESI Frag=150V

Spectrum: MSD1 +Scan ESI (rt: 15.409 min) Subtract (rt: 13.802 min)

No spectra available!

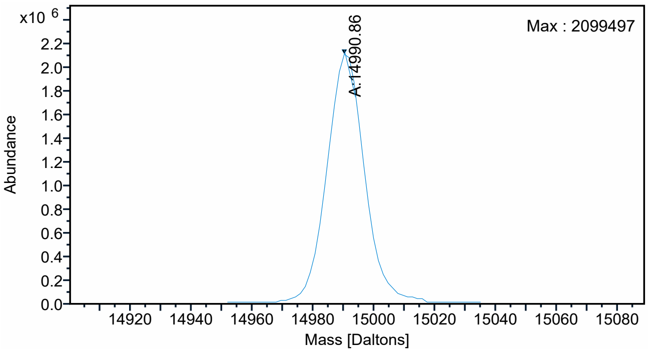

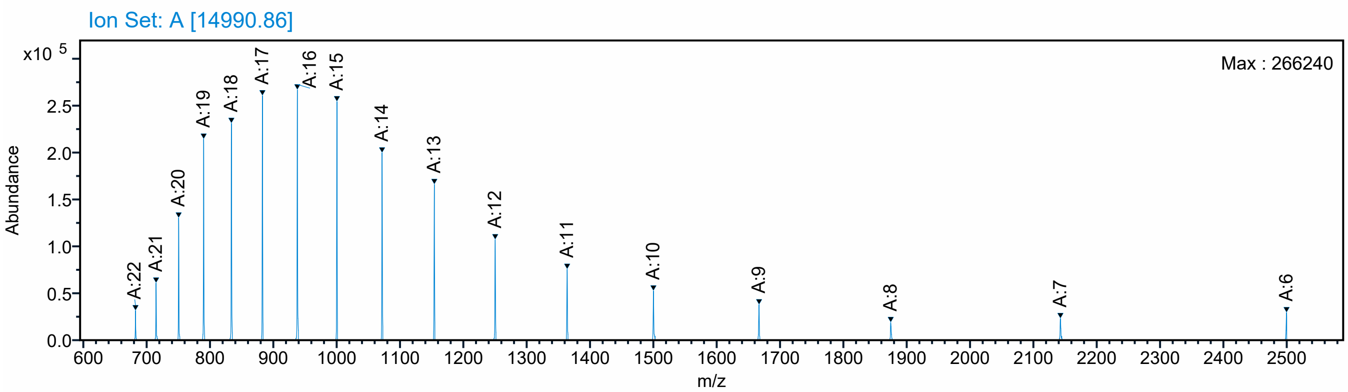

Component	Mass	Absolute Abundance	Relative Abundance (%)	Relative Quantitation (%)
A	14990.86	2099497	100.00	100.00

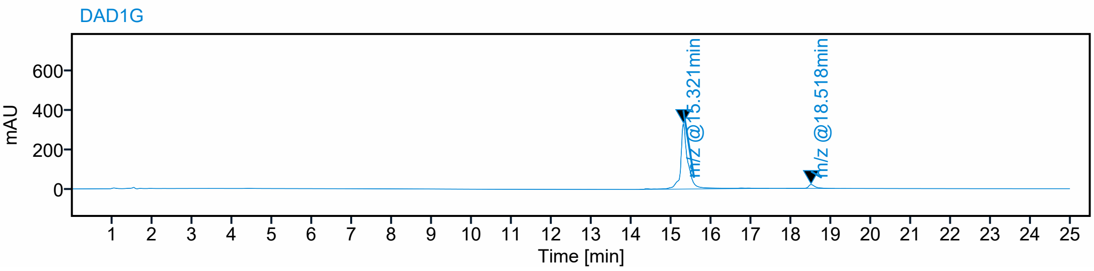

Signal: DAD1G

Name	RT [min]	RF	Area	Peak Area Percent	Group
	15.321		4234.0514	94.64	UNMODIFIED PROTEIN - 100%
	~~18.518~~		~~239.8899~~	~~5.36~~	no protein detected

Signal: MSD1 +TIC ESI Frag=150V

Name	RT [min]	RF	Area	Peak Area Percent	Group
	15.409		318823636.5257	100.00	UNMODIFIED PROTEIN - 100%

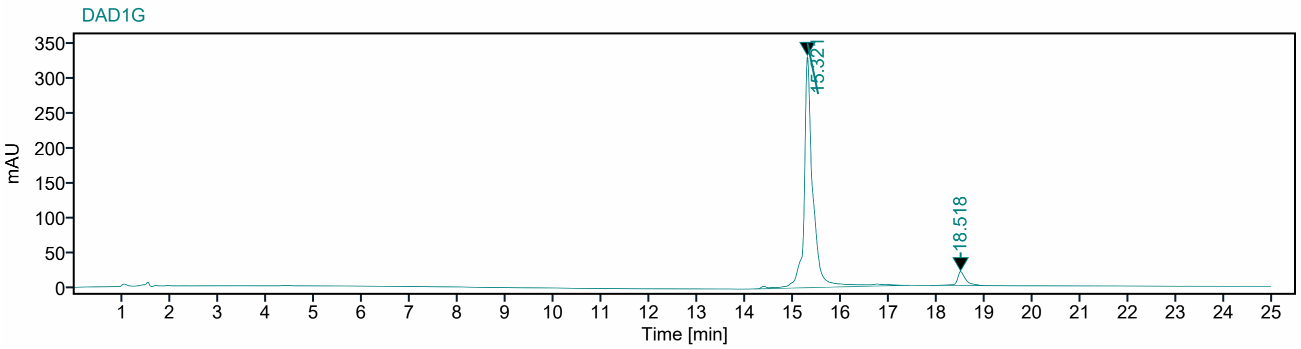
DAD1G
mAU
350
300
250
200
150
100
50
0
15.321
18.518
1 2 3 4 5 6 7 8 9 10 11 12 13 14 15 16 17 18 19 20 21 22 23 24 25
Time [min]

Single Injection Report

Agilent

**Data file:**	100125-10-75OVER20_PEPTIDES-C3_200M-30688_020.D		
**Sequence Name:**	Chemstatioimports_MRR	**Project Name:**	Walkup Submissions
**Sample name:**	BromoTag_mr101_10Jan25	**Operator:**	Maria Rodriguez
**Instrument:**		**Injection date:**	2025-01-10 19:03:24-08:00
**Inj. volume:**		**Location:**	P2-B-02
**Acq. method:**	10-75OVER20_PEPTIDES-C3_200MZ.M	**Type:**	Sample
**Processing method:**	*Deconvolution Test 2.pmx	**Sample amount:**	
**Manually modified:**	None		

Data Analysis Method: Deconvolution Test 2.pmx
Path: D:\CDSProjects\Walkup Submissions\Results\Chemstatioimports_MRR.rslt
Method parameters are filtered - only a subset is displayed

2 Method Parameters

2.11 MS Spectral Deconvolution Parameters

**Run automatic deconvolution:**	Yes	**Use RT window:**	No	**TIC peak type:**	All peaks
**TIC peak threshold:**	Top (n) peaks	**Top (n) peaks:**	6	**Positive adduct:**	+H
**Negative adduct:**	-H	**Use m/z range:**	No	**Low molecular weight:**	4000
**High molecular weight:**	25000	**Maximum charge:**	40	**Minimum peaks in set:**	3
**Show unmatched peaks:**	No	**MW agreement (0.01%):**	5	**Absolute noise threshold:**	1000
**Relative abundance threshold (%):**	10	**MW algorithm:**	Curve Fit	**MW algorithm threshold (%):**	40
**Envelope threshold (%):**	50				

Method Audit Trail

Method audit trail is not printed

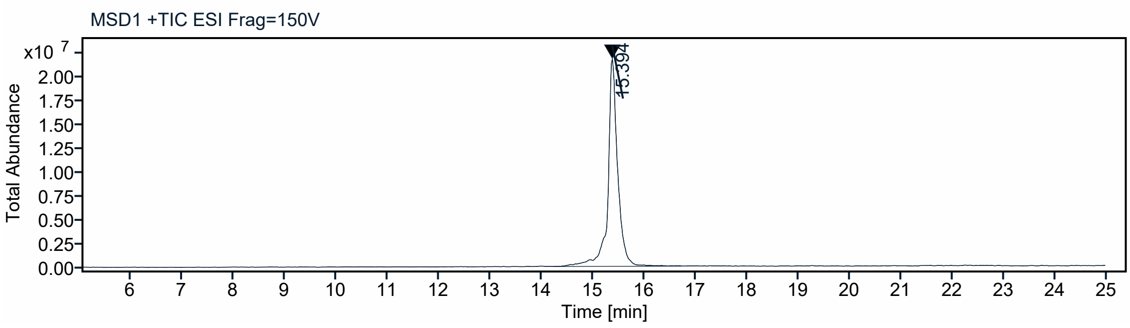

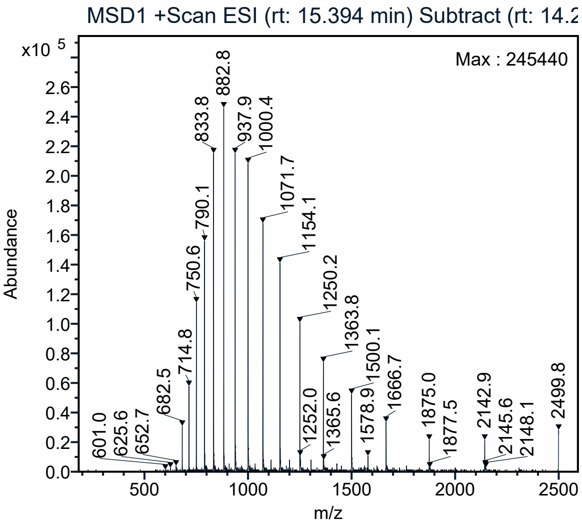

Deconvolution of peak at RT: 15.394

Signal: MSD1 +TIC ESI Frag=150V

Spectrum: MSD1 +Scan ESI (rt: 15.394 min) Subtract (rt: 14.239 min)

No spectra available!

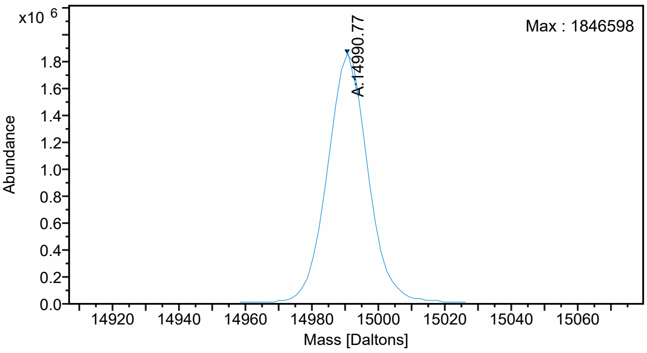

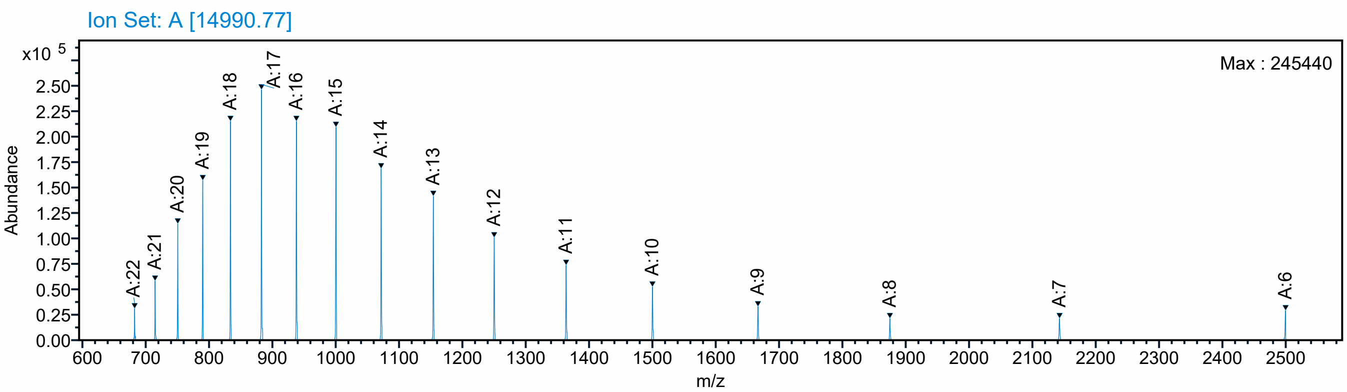

Component	Mass	Absolute Abundance	Relative Abundance (%)	Relative Quantitation (%)
A	14990.77	1846598	100.00	100.00

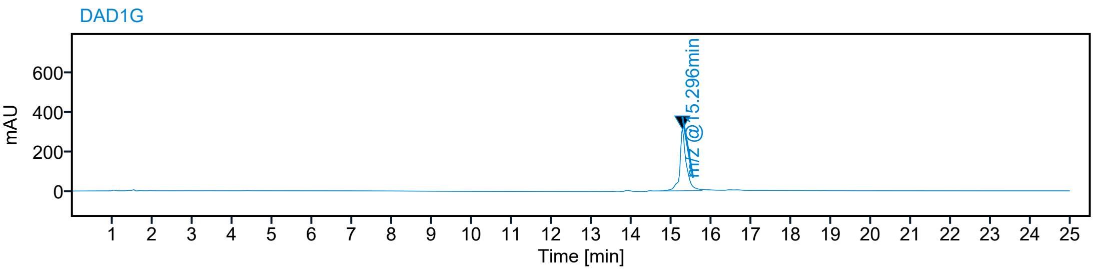

Signal: DAD1G

Name	RT [min]	RF	Area	Peak Area Percent	Group
	15.296		3476.6229	100.00	UNMODIFIED PROTEIN - 100%

Signal: MSD1 +TIC ESI Frag=150V

Name	RT [min]	RF	Area	Peak Area Percent	Group
	15.394		289793894.3885	100.00	UNMODIFIED PROTEIN - 100%

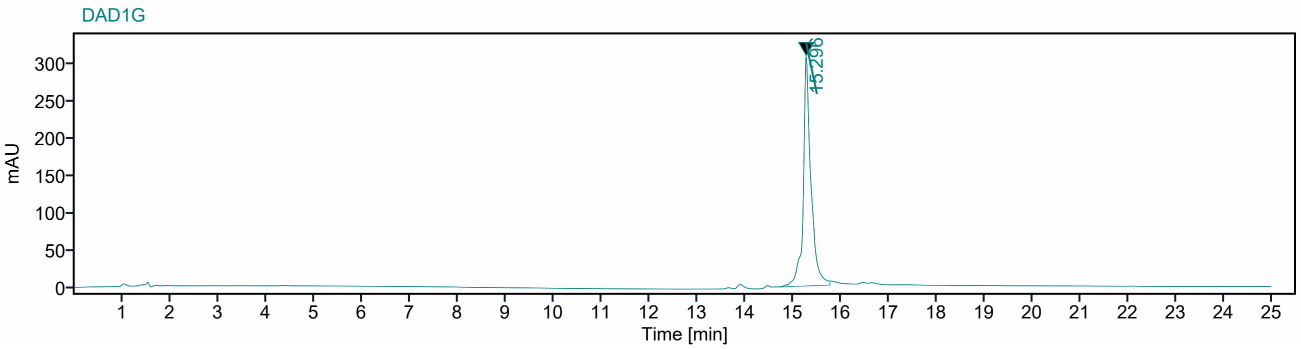
DAD1G
mAU
300
250
200
150
100
50
0
15.296
1
2
3
4
5
6
7
8
9
10
11
12
13
14
15
16
17
18
19
20
21
22
23
24
25
Time [min]

**Data file:**	100125-10-75OVER20_PEPTIDES-C3_200M-30689_021.D		
**Sequence Name:**	Chemstatioimports_MRR	**Project Name:**	Walkup Submissions
**Sample name:**	BromoTag_MR70_10Jan25	**Operator:**	Maria Rodriguez
**Instrument:**		**Injection date:**	2025-01-10 19:33:17-08:00
**Inj. volume:**		**Location:**	P2-B-03
**Acq. method:**	10-75OVER20_PEPTIDES-C3_200MZ.M	**Type:**	Sample
**Processing method:**	*Deconvolution Test 2.pmx	**Sample amount:**	
**Manually modified:**	None		

Data Analysis Method: **Deconvolution Test 2.pmx**
Path: **D:\CDSProjects\Walkup Submissions\Results\Chemstatioimports_MRR.rslt**
Method parameters are filtered - only a subset is displayed

2 Method Parameters

2.11 MS Spectral Deconvolution Parameters

**Run automatic deconvolution:**	Yes	**Use RT window:**	No	**TIC peak type:**	All peaks
**TIC peak threshold:**	Top (n) peaks	**Top (n) peaks:**	6	**Positive adduct:**	+H
**Negative adduct:**	-H	**Use m/z range:**	No	**Low molecular weight:**	4000
**High molecular weight:**	25000	**Maximum charge:**	40	**Minimum peaks in set:**	3
**Show unmatched peaks:**	No	**MW agreement (0.01%):**	5	**Absolute noise threshold:**	1000
**Relative abundance threshold (%):**	10	**MW algorithm:**	Curve Fit	**MW algorithm threshold (%):**	40
**Envelope threshold (%):**	50				

Method Audit Trail

Method audit trail is not printed

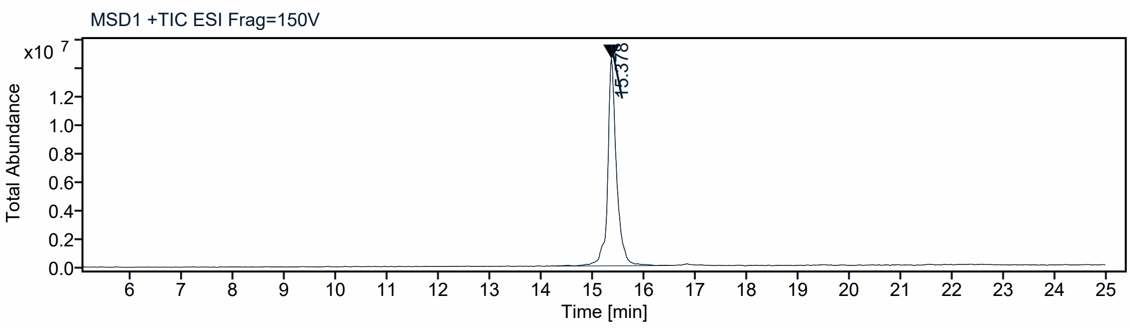

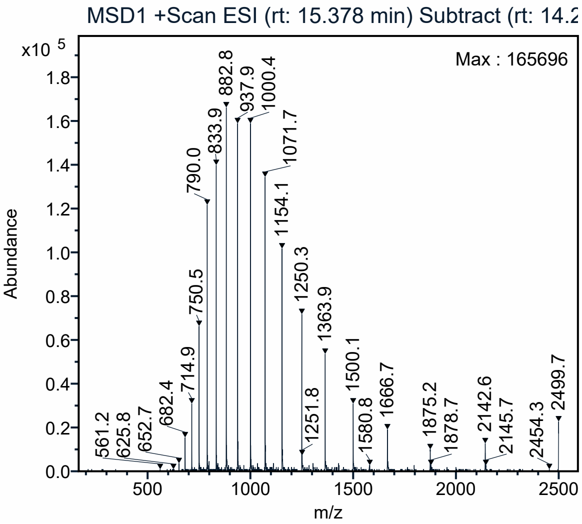

Deconvolution of peak at RT: 15.378

Signal: MSD1 +TIC ESI Frag=150V

Spectrum: MSD1 +Scan ESI (rt: 15.378 min) Subtract (rt: 14.286 min)

No spectra available!

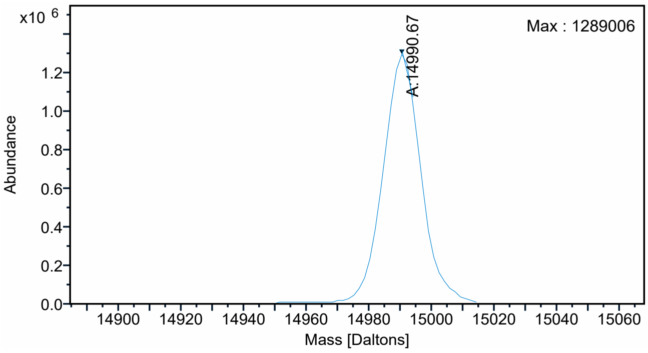

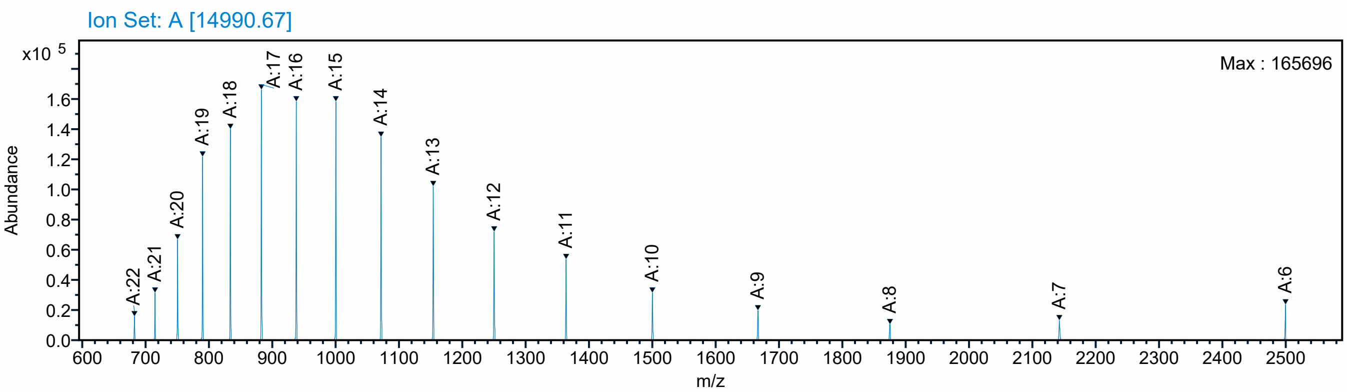

Component	Mass	Absolute Abundance	Relative Abundance (%)	Relative Quantitation (%)
A	14990.67	1289006	100.00	100.00

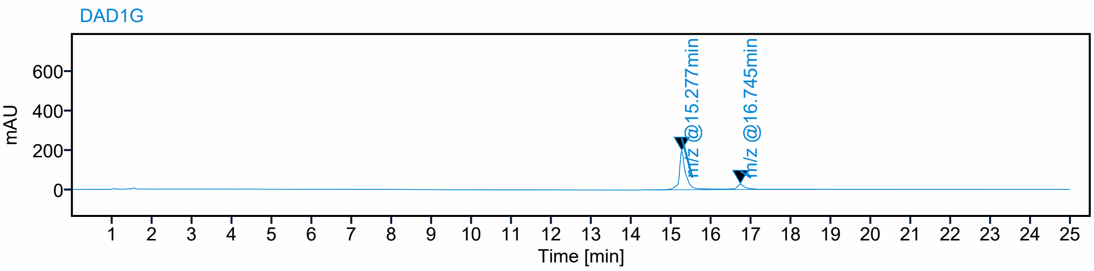

Signal: DAD1G

Name	RT [min]	RF	Area	Peak Area Percent	Group
	15.277		2258.4715	82.53	unmodified protein - 100%
	16.745		478.1853	17.47	no protein detected ir

Signal: MSD1 +TIC ESI Frag=150V

Name	RT [min]	RF	Area	Peak Area Percent	Group
	15.378		175138635.6670	100.00	unmodified protein

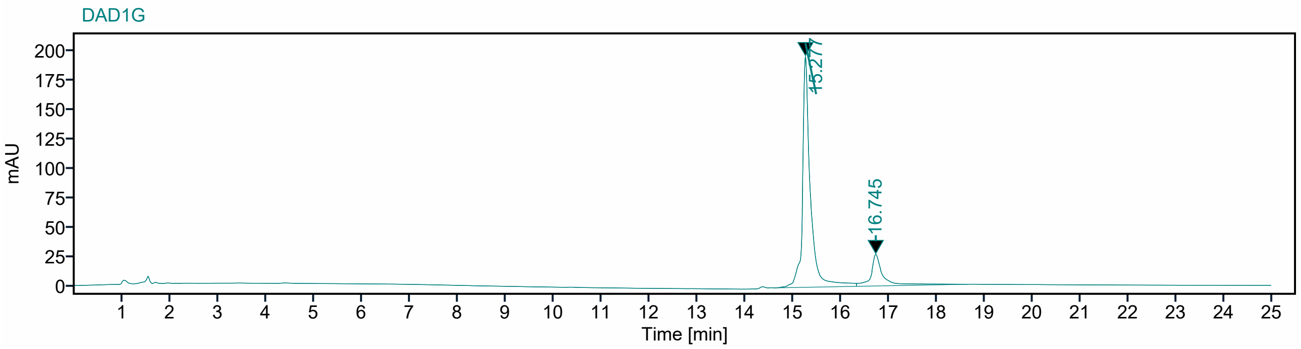
DAD1G
mAU
200
175
150
125
100
75
50
25
0
15.277
16.745
1
2
3
4
5
6
7
8
9
10
11
12
13
14
15
16
17
18
19
20
21
22
23
24
25
Time [min]

Single Injection Report

**Data file:**	100125-10-75OVER20_PEPTIDES-C3_200M-30690_022.D		
**Sequence Name:**	Chemstatioimports_MRR	**Project Name:**	Walkup Submissions
**Sample name:**	BromoTag_MR104_10Jan25	**Operator:**	Maria Rodriguez
**Instrument:**		**Injection date:**	2025-01-10 20:03:06-08:00
**Inj. volume:**		**Location:**	P2-B-04
**Acq. method:**	10-75OVER20_PEPTIDES-C3_200MZ.M	**Type:**	Sample
**Processing method:**	*Deconvolution Test 2.pmx	**Sample amount:**	
**Manually modified:**	Manual Integration		

Data Analysis Method: **Deconvolution Test 2.pmx**
Path: **D:\CDSProjects\Walkup Submissions\Results\Chemstatioimports_MRR.rslt**
Method parameters are filtered - only a subset is displayed

2 Method Parameters

2.11 MS Spectral Deconvolution Parameters

**Run automatic deconvolution:**	Yes	**Use RT window:**	No	**TIC peak type:**	All peaks
**TIC peak threshold:**	Top (n) peaks	**Top (n) peaks:**	6	**Positive adduct:**	+H
**Negative adduct:**	-H	**Use m/z range:**	No	**Low molecular weight:**	4000
**High molecular weight:**	25000	**Maximum charge:**	40	**Minimum peaks in set:**	3
**Show unmatched peaks:**	No	**MW agreement (0.01%):**	5	**Absolute noise threshold:**	1000
**Relative abundance threshold (%):**	10	**MW algorithm:**	Curve Fit	**MW algorithm threshold (%):**	40
**Envelope threshold (%):**	50				

Method Audit Trail

Method audit trail is not printed

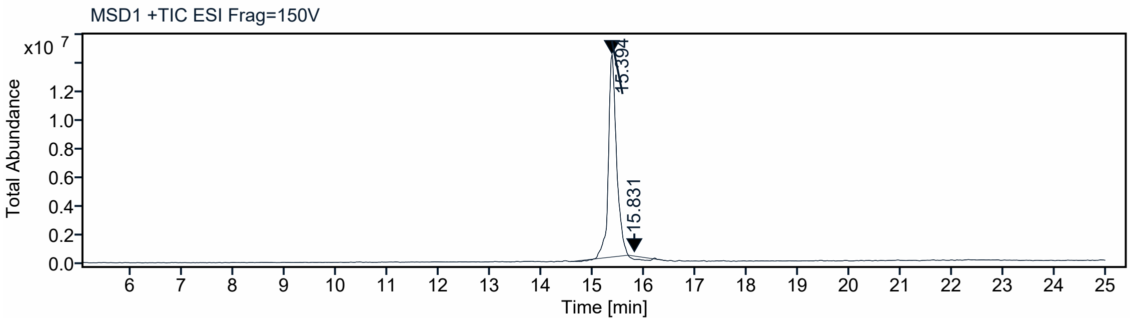

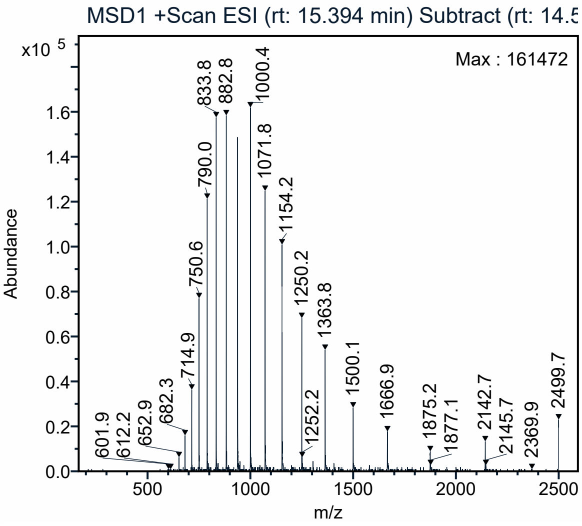

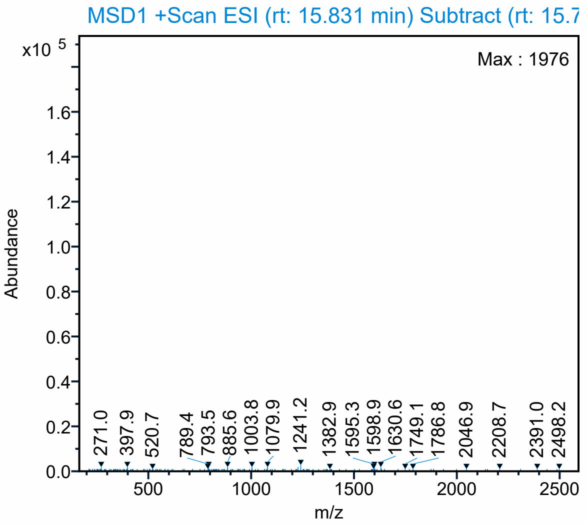

Deconvolution of peak at RT: 15.394

Signal: MSD1 +TIC ESI Frag=150V

Spectrum: MSD1 +Scan ESI (rt: 15.394 min) Subtract (rt: 14.583 min)

No spectra available!

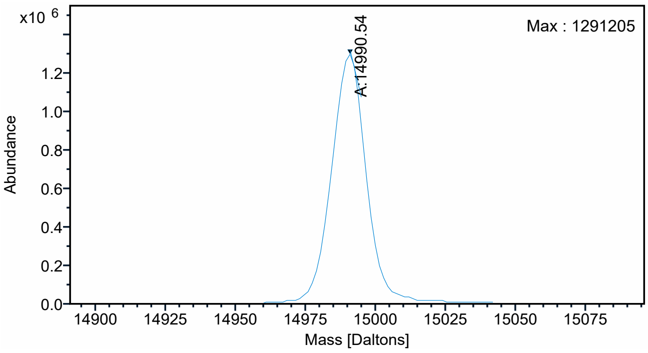

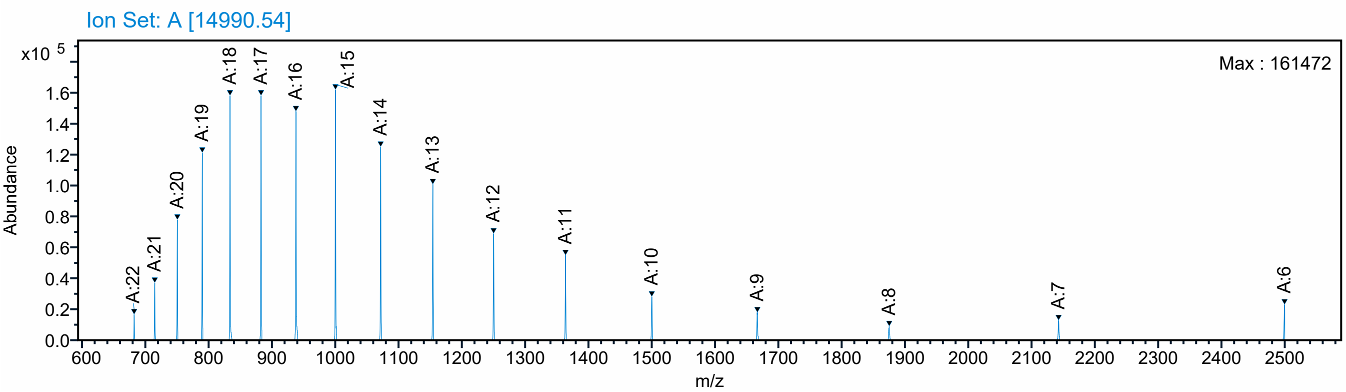

Component	Mass	Absolute Abundance	Relative Abundance (%)	Relative Quantitation (%)
A	14990.54	1291205	100.00	100.00

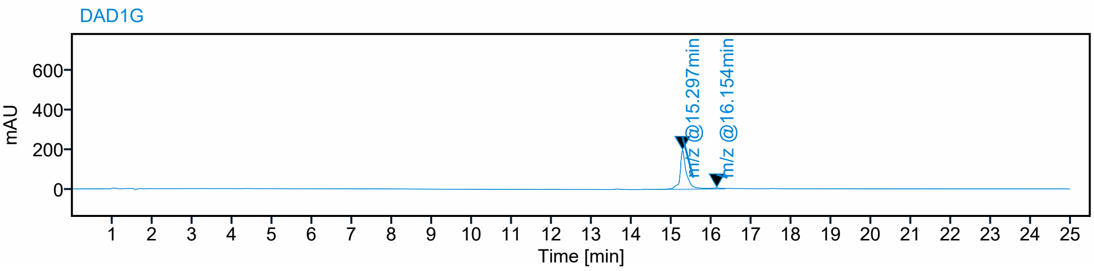

Signal: DAD1G

Name	RT [min]	RF	Area	Peak Area Percent	Group
	15.297		2176.9506	96.14	umodified protein 100%
	~~16.154~~		~~87.4237~~	~~3.86~~	no protein detected in MS

Signal: MSD1 +TIC ESI Frag=150V

Name	RT [min]	RF	Area	Peak Area Percent	Group
	15.394		154943168.5338	97.07	unmodified protein 100%
	~~15.831~~		~~4678012.4384~~	~~2.93~~	no protein detected in MS

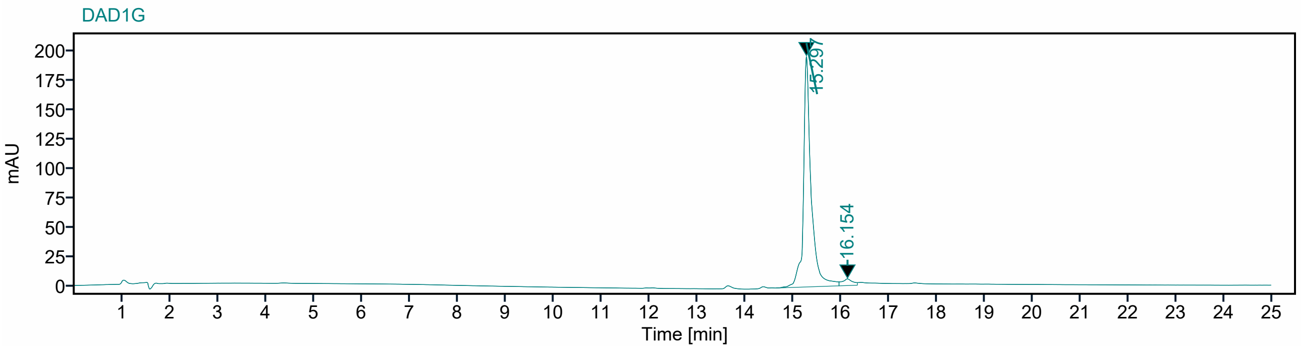
DAD1G
mAU
200
175
150
125
100
75
50
25
0
15.297
16.154
1 2 3 4 5 6 7 8 9 10 11 12 13 14 15 16 17 18 19 20 21 22 23 24 25
Time [min]

**Data file:**	100125-10-75OVER20_PEPTIDES-C3_200M-30691_023.D		
**Sequence Name:**	Chemstatioimports_MRR	**Project Name:**	Walkup Submissions
**Sample name:**	BromoTag_MR112_10Jan25	**Operator:**	Maria Rodriguez
**Instrument:**		**Injection date:**	2025-01-10 20:32:59-08:00
**Inj. volume:**		**Location:**	P2-B-05
**Acq. method:**	10-75OVER20_PEPTIDES-C3_200MZ.M	**Type:**	Sample
**Processing method:**	*Deconvolution Test 2.pmx	**Sample amount:**	
**Manually modified:**	Manual Integration		

Data Analysis Method: **Deconvolution Test 2.pmx**
Path: **D:\CDSProjects\Walkup Submissions\Results\Chemstatioimports_MRR.rslt**
Method parameters are filtered - only a subset is displayed

2 Method Parameters

2.11 MS Spectral Deconvolution Parameters

**Run automatic deconvolution:**	Yes	**Use RT window:**	No	**TIC peak type:**	All peaks
**TIC peak threshold:**	Top (n) peaks	**Top (n) peaks:**	6	**Positive adduct:**	+H
**Negative adduct:**	-H	**Use m/z range:**	No	**Low molecular weight:**	4000
**High molecular weight:**	25000	**Maximum charge:**	40	**Minimum peaks in set:**	3
**Show unmatched peaks:**	No	**MW agreement (0.01%):**	5	**Absolute noise threshold:**	1000
**Relative abundance threshold (%):**	10	**MW algorithm:**	Curve Fit	**MW algorithm threshold (%):**	40
**Envelope threshold (%):**	50				

Method Audit Trail

Method audit trail is not printed

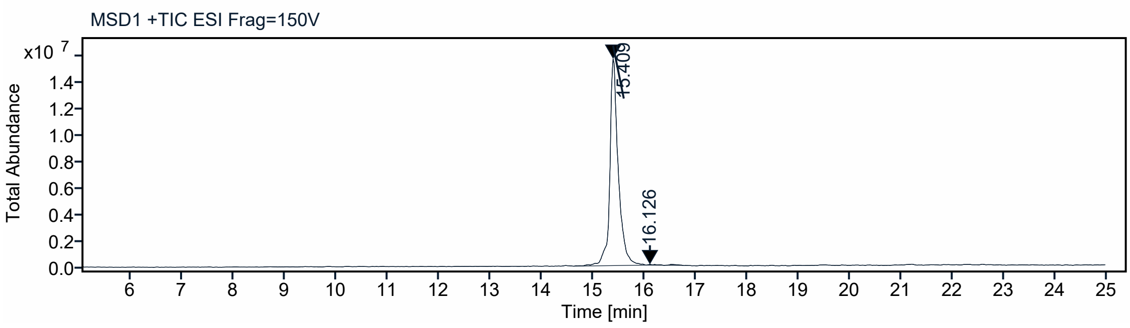

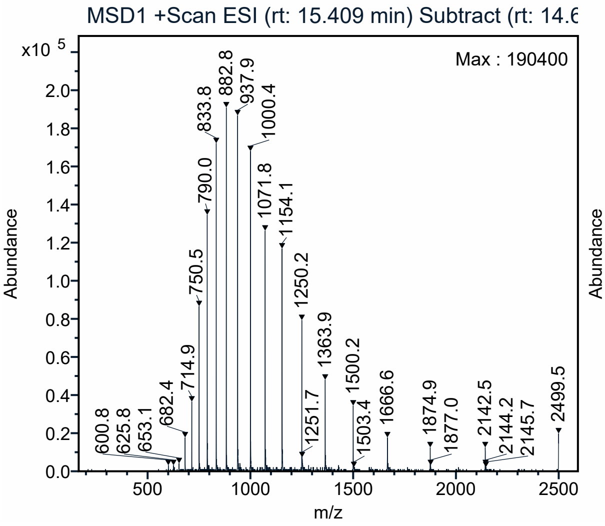

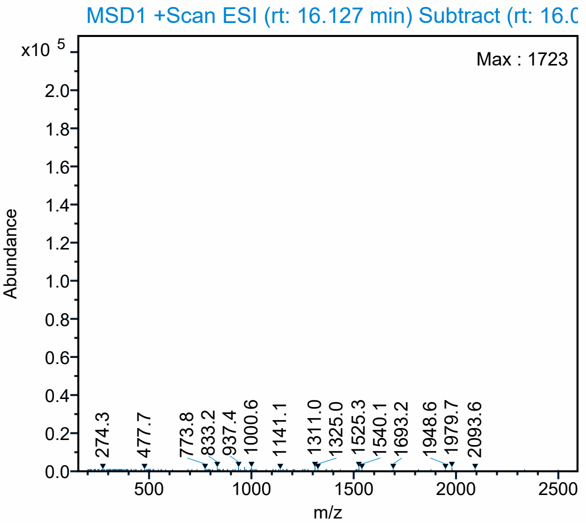

Deconvolution of peak at RT: 15.409

Signal: MSD1 +TIC ESI Frag=150V

Spectrum: MSD1 +Scan ESI (rt: 15.409 min) Subtract (rt: 14.645 min)

No spectra available!

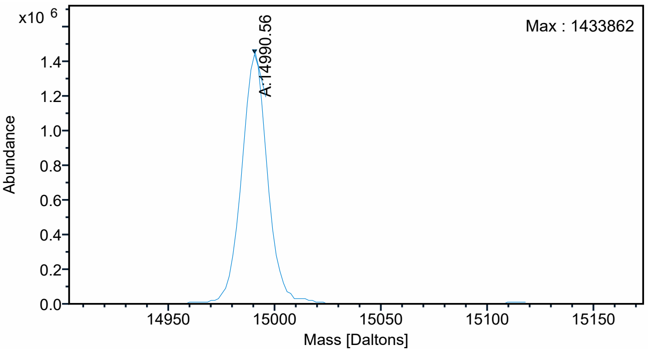

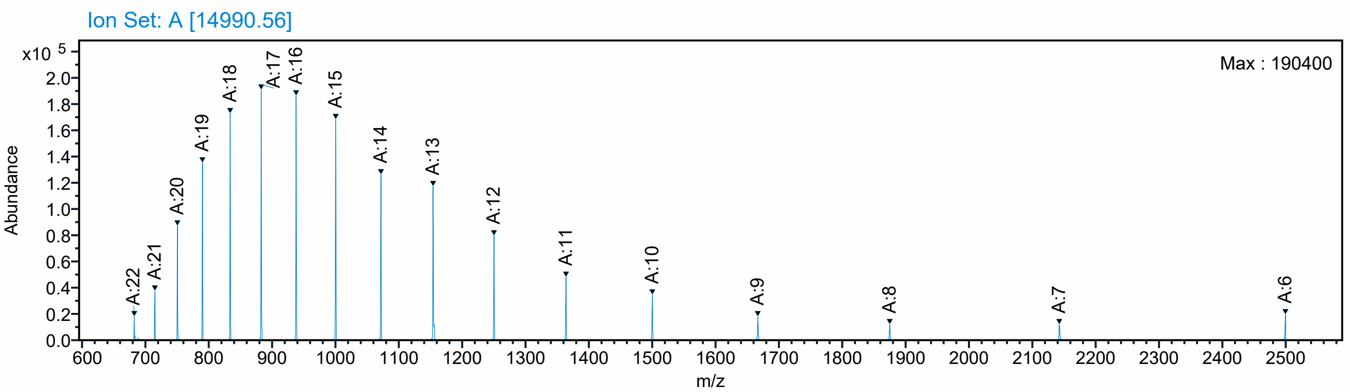

Component	Mass	Absolute Abundance	Relative Abundance (%)	Relative Quantitation (%)
A	14990.56	1433862	100.00	100.00

Deconvolution of peak at RT: 16.126

Signal: MSD1 +TIC ESI Frag=150V

Spectrum: MSD1 +Scan ESI (rt: 16.127 min) Subtract (rt: 16.049 min)

No spectra available!

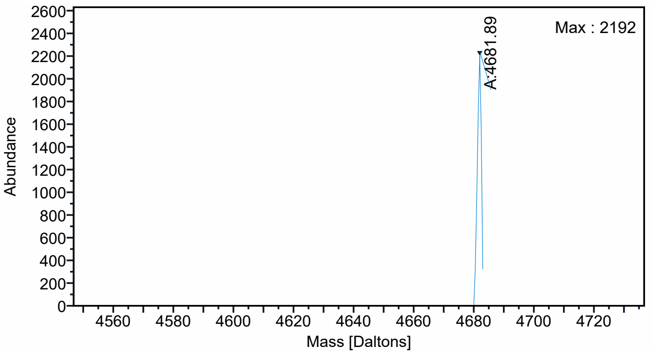

Ion Set: A [4681.89]

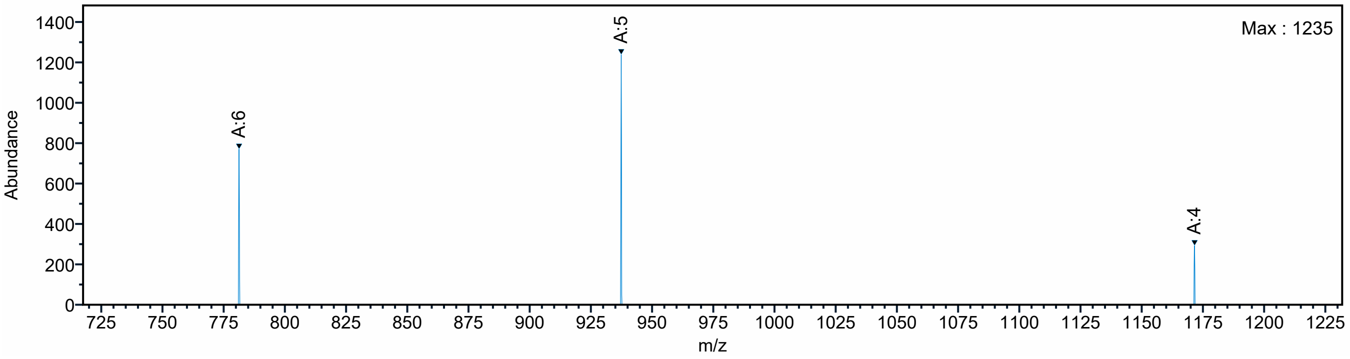

Component	Mass	Absolute Abundance	Relative Abundance (%)	Relative Quantitation (%)
A	4681.89	2192	100.00	100.00

DAD1G

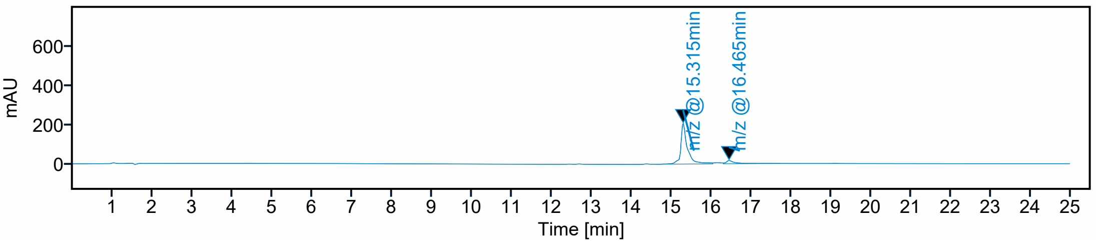

Signal: DAD1G

Name	RT [min]	RF	Area	Peak Area Percent	Group
	15.315		2370.2259	89.33	unmodified protein - 100%
	~~16.465~~		~~283.2147~~	~~10.67~~	no protein detected

Signal: MSD1 +TIC ESI Frag=150V

Name	RT [min]	RF	Area	Peak Area Percent	Group
	15.409		179769843.1830	99.28	unmodified protein - 100%
	~~16.126~~		~~1306140.0607~~	~~0.72~~	no protein detected in MS

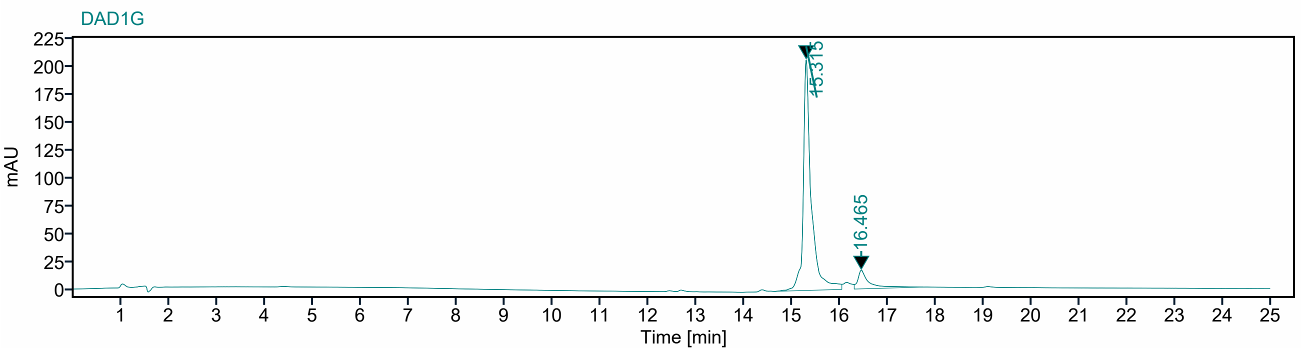

Single Injection Report

**Data file:**	100125-10-75OVER20_PEPTIDES-C3_200M-30692_024.D		
**Sequence Name:**	Chemstatioimports_MRR	**Project Name:**	Walkup Submissions
**Sample name:**	BromoTag_MR121_10Jan25	**Operator:**	Maria Rodriguez
**Instrument:**		**Injection date:**	2025-01-10 21:02:51-08:00
**Inj. volume:**		**Location:**	P2-B-06
**Acq. method:**	10-75OVER20_PEPTIDES-C3_200MZ.M	**Type:**	Sample
**Processing method:**	*Deconvolution Test 2.pmx	**Sample amount:**	
**Manually modified:**	Manual Integration		

Data Analysis Method: **Deconvolution Test 2.pmx**
Path: **D:\CDSProjects\Walkup Submissions\Results\Chemstatioimports_MRR.rslt**
Method parameters are filtered - only a subset is displayed

2 Method Parameters

2.11 MS Spectral Deconvolution Parameters

**Run automatic deconvolution:**	Yes	**Use RT window:**	No	**TIC peak type:**	All peaks
**TIC peak threshold:**	Top (n) peaks	**Top (n) peaks:**	6	**Positive adduct:**	+H
**Negative adduct:**	-H	**Use m/z range:**	No	**Low molecular weight:**	4000
**High molecular weight:**	25000	**Maximum charge:**	40	**Minimum peaks in set:**	3
**Show unmatched peaks:**	No	**MW agreement (0.01%):**	5	**Absolute noise threshold:**	1000
**Relative abundance threshold (%):**	10	**MW algorithm:**	Curve Fit	**MW algorithm threshold (%):**	40
**Envelope threshold (%):**	50				

Method Audit Trail

Method audit trail is not printed

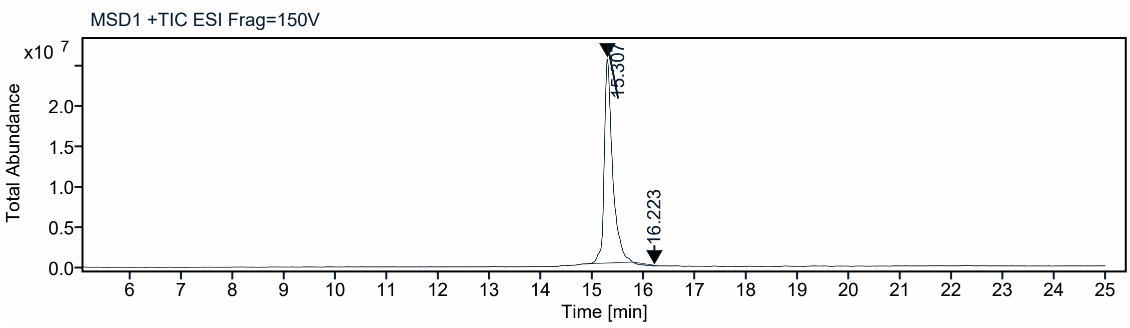

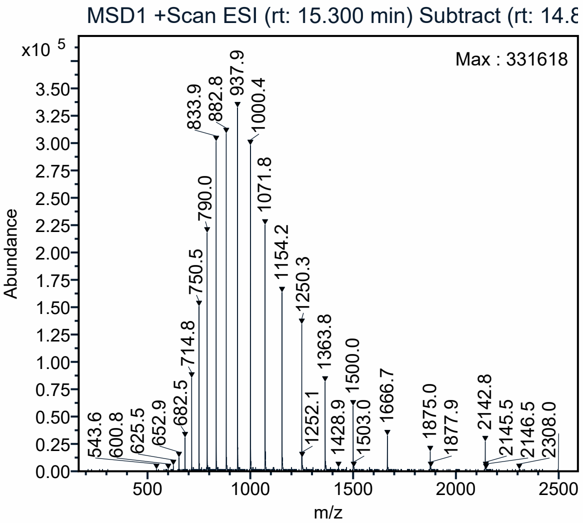

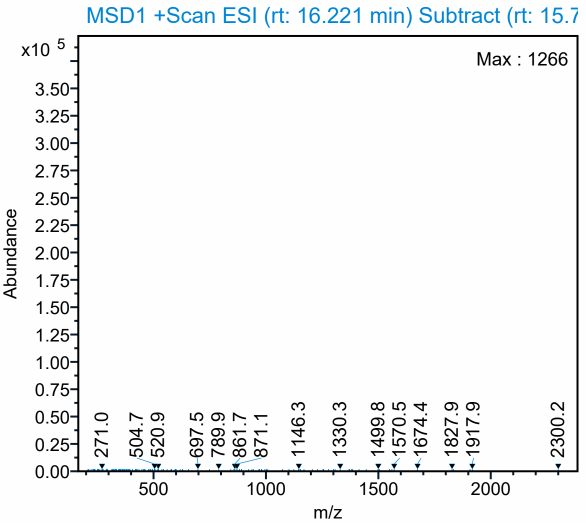

Deconvolution of peak at RT: 15.307

Signal: MSD1 +TIC ESI Frag=150V

Spectrum: MSD1 +Scan ESI (rt: 15.300 min) Subtract (rt: 14.895 min)

No spectra available!

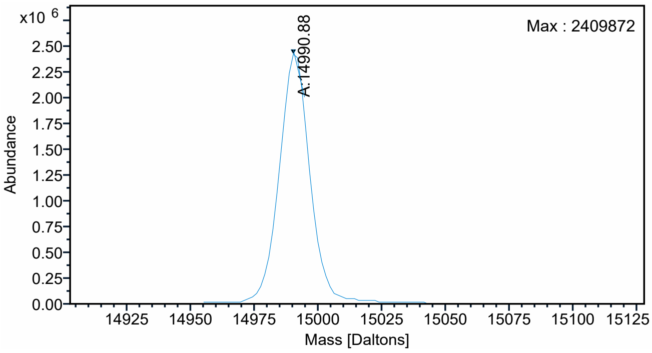

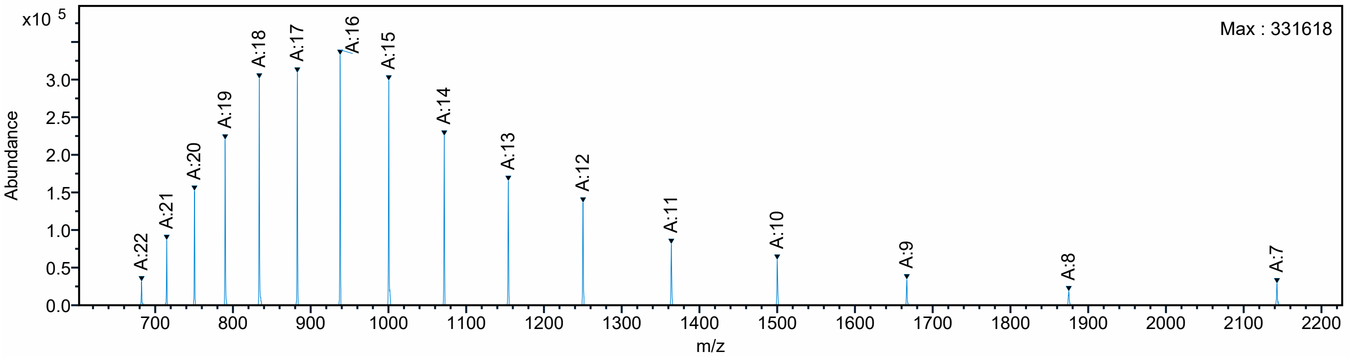

Component	Mass	Absolute Abundance	Relative Abundance (%)	Relative Quantitation (%)
A	14990.88	2409872	100.00	100.00

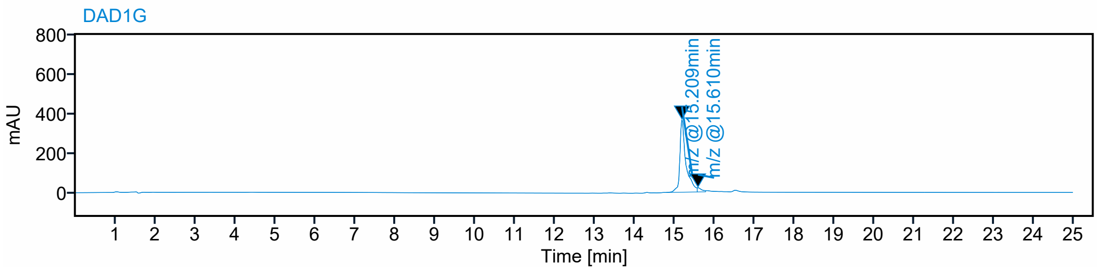

Signal: DAD1G

Name	RT [min]	RF	Area	Peak Area Percent	Group	
	15.209		3898.0856	95.97		unmodified protein - 100%
	~~15.610~~		~~163.5941~~	~~4.03~~		no protein detected

Signal: MSD1 +TIC ESI Frag=150V

Name	RT [min]	RF	Area	Peak Area Percent	Group	
	15.307		285932907.2752	98.72		unmodified protein - 100%
	~~16.223~~		~~3714062.3216~~	~~1.28~~		no protein detected in MS

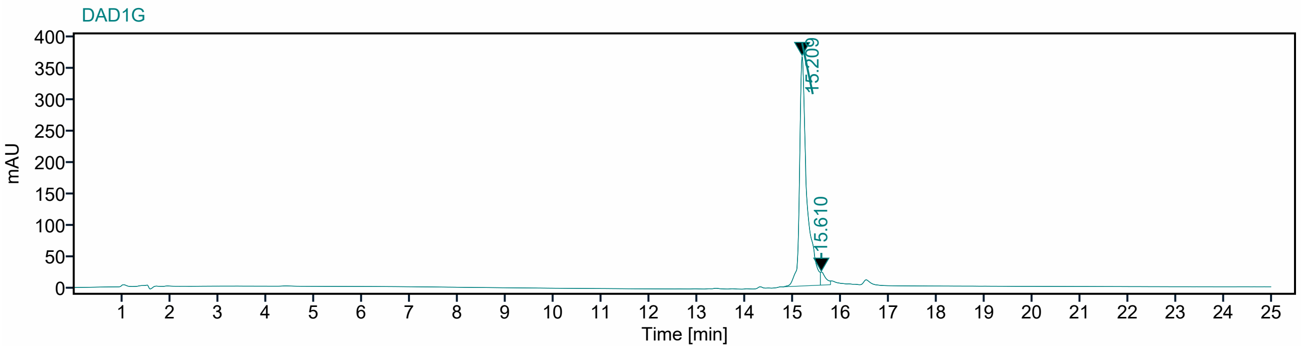
DAD1G
mAU
400
350
300
250
200
150
100
50
0
15.209
15.610
1
2
3
4
5
6
7
8
9
10
11
12
13
14
15
16
17
18
19
20
21
22
23
24
25
Time [min]

Single Injection Report

**Data file:**	100125-10-75OVER20_PEPTIDES-C3_200M-30693_025.D		
**Sequence Name:**	Chemstatioimports_MRR	**Project Name:**	Walkup Submissions
**Sample name:**	BromoTag_MR116_10Jan25	**Operator:**	Maria Rodriguez
**Instrument:**		**Injection date:**	2025-01-10 21:32:43-08:00
**Inj. volume:**		**Location:**	P2-B-07
**Acq. method:**	10-75OVER20_PEPTIDES-C3_200MZ.M	**Type:**	Sample
**Processing method:**	*Deconvolution Test 2.pmx	**Sample amount:**	
**Manually modified:**	Manual Integration		

Data Analysis Method: **Deconvolution Test 2.pmx**
Path: **D:\CDSProjects\Walkup Submissions\Results\Chemstatioimports_MRR.rslt**
Method parameters are filtered - only a subset is displayed

2 Method Parameters

2.11 MS Spectral Deconvolution Parameters

**Run automatic deconvolution:**	Yes	**Use RT window:**	No	**TIC peak type:**	All peaks
**TIC peak threshold:**	Top (n) peaks	**Top (n) peaks:**	6	**Positive adduct:**	+H
**Negative adduct:**	-H	**Use m/z range:**	No	**Low molecular weight:**	4000
**High molecular weight:**	25000	**Maximum charge:**	40	**Minimum peaks in set:**	3
**Show unmatched peaks:**	No	**MW agreement (0.01%):**	5	**Absolute noise threshold:**	1000
**Relative abundance threshold (%):**	10	**MW algorithm:**	Curve Fit	**MW algorithm threshold (%):**	40
**Envelope threshold (%):**	50				

Method Audit Trail

Method audit trail is not printed

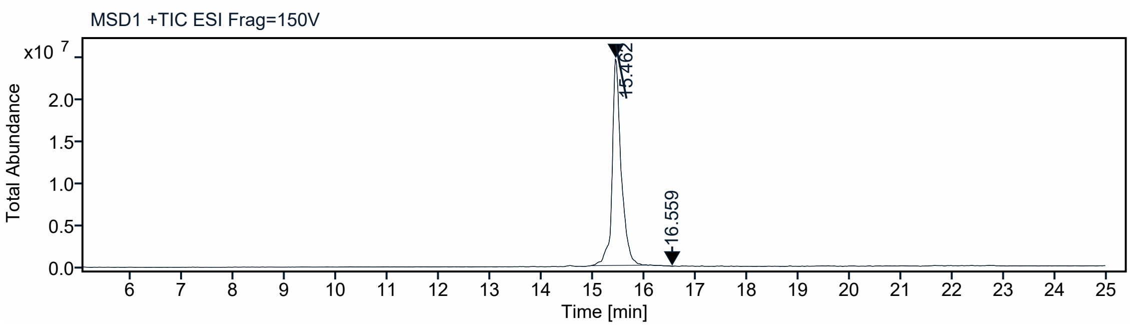

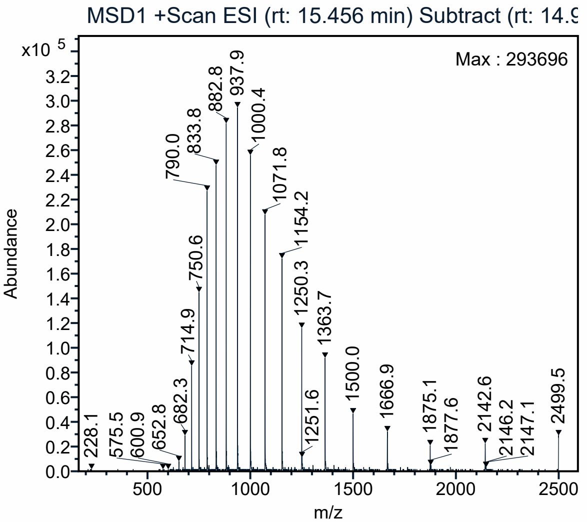

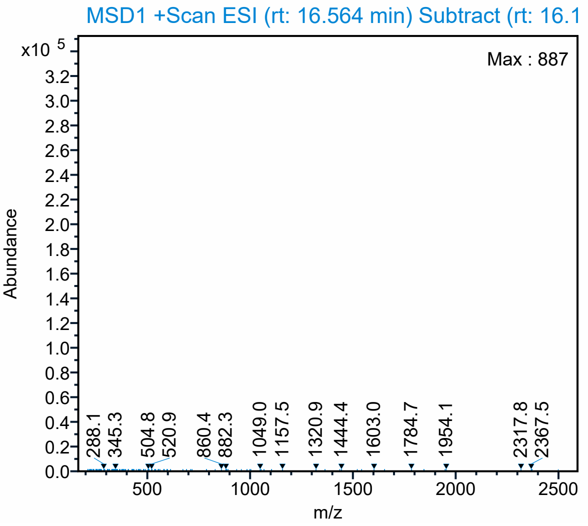

Deconvolution of peak at RT: 15.462

Signal: MSD1 +TIC ESI Frag=150V

Spectrum: MSD1 +Scan ESI (rt: 15.456 min) Subtract (rt: 14.973 min)

No spectra available!

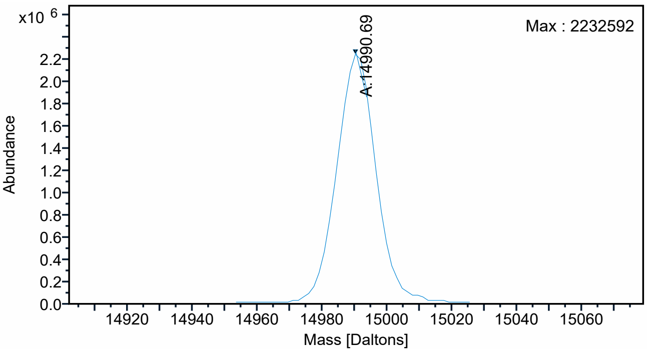

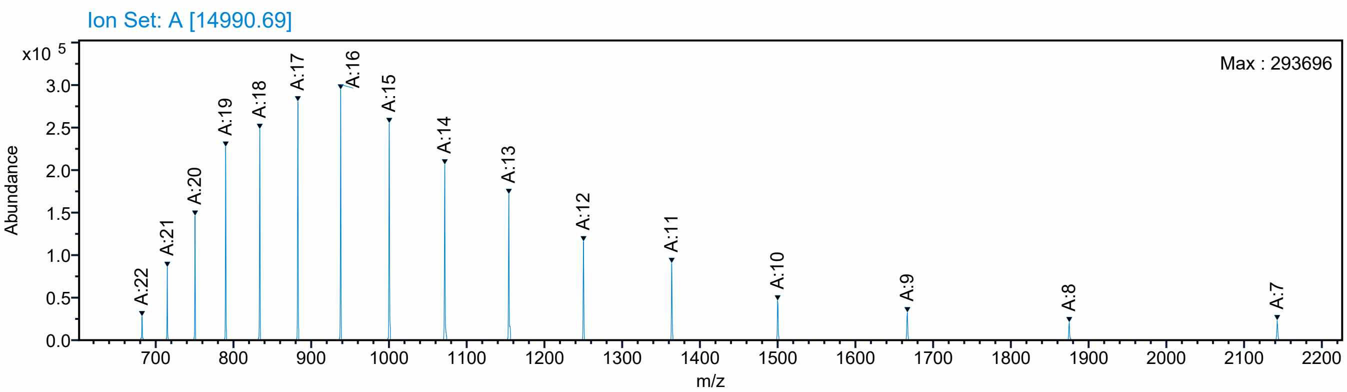

Component	Mass	Absolute Abundance	Relative Abundance (%)	Relative Quantitation (%)
A	14990.69	2232592	100.00	100.00

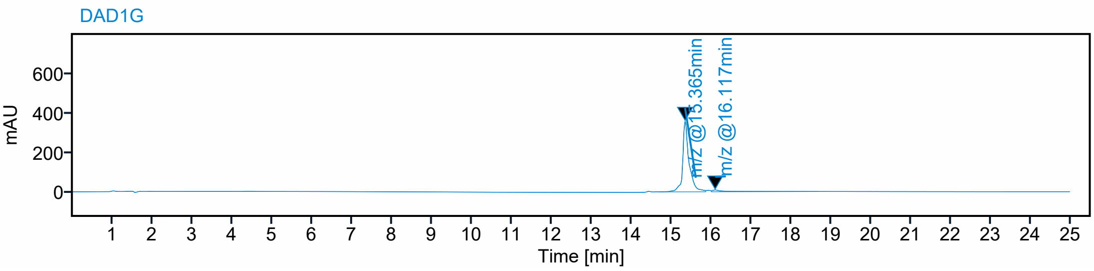

Signal: DAD1G

Name	RT [min]	RF	Area	Peak Area Percent	Group
	15.365		3948.7647	93.54	UNMODIFIED PROTEIN - 100%
	~~16.117~~		~~272.6483~~	~~6.46~~	no protein detected in MS

Signal: MSD1 +TIC ESI Frag=150V

Name	RT [min]	RF	Area	Peak Area Percent	Group
	15.462		300121387.1369	99.87	UNMODIFIED PROTEIN - 100%
	~~16.559~~		~~376969.7095~~	~~0.13~~	no protein detected in MS

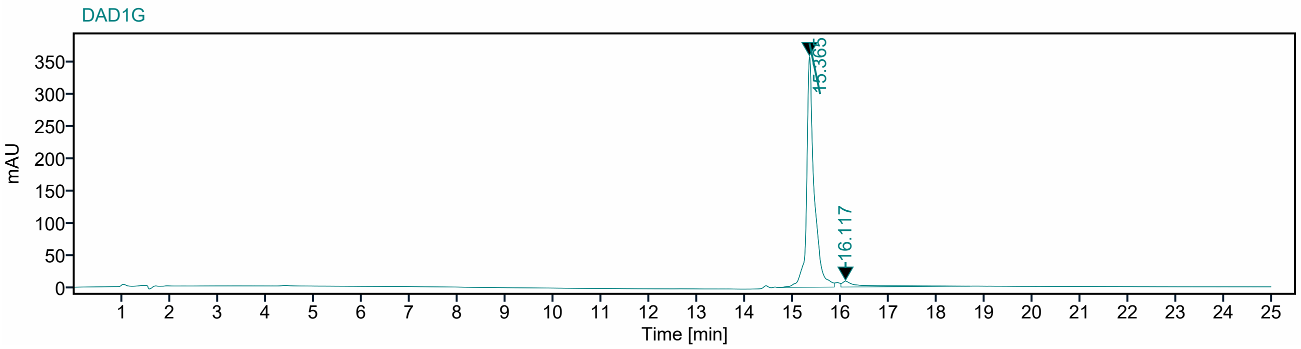
DAD1G
mAU
350
300
250
200
150
100
50
0
15.365
16.117
1 2 3 4 5 6 7 8 9 10 11 12 13 14 15 16 17 18 19 20 21 22 23 24 25
Time [min]

Single Injection Report

**Data file:**	100125-10-75OVER20_PEPTIDES-C3_200M-30694_026.D		
**Sequence Name:**	Chemstatioimports_MRR	**Project Name:**	Walkup Submissions
**Sample name:**	BromoTag_MR108_10Jan25	**Operator:**	Maria Rodriguez
**Instrument:**		**Injection date:**	2025-01-10 22:02:34-08:00
**Inj. volume:**		**Location:**	P2-B-08
**Acq. method:**	10-75OVER20_PEPTIDES-C3_200MZ.M	**Type:**	Sample
**Processing method:**	*Deconvolution Test 2.pmx	**Sample amount:**	
**Manually modified:**	Manual Integration		

Data Analysis Method: **Deconvolution Test 2.pmx**
Path: **D:\CDSProjects\Walkup Submissions\Results\Chemstatioimports_MRR.rslt**
Method parameters are filtered - only a subset is displayed

2 Method Parameters

2.11 MS Spectral Deconvolution Parameters

**Run automatic deconvolution:**	Yes	**Use RT window:**	No	**TIC peak type:**	All peaks
**TIC peak threshold:**	Top (n) peaks	**Top (n) peaks:**	6	**Positive adduct:**	+H
**Negative adduct:**	-H	**Use m/z range:**	No	**Low molecular weight:**	4000
**High molecular weight:**	25000	**Maximum charge:**	40	**Minimum peaks in set:**	3
**Show unmatched peaks:**	No	**MW agreement (0.01%):**	5	**Absolute noise threshold:**	1000
**Relative abundance threshold (%):**	10	**MW algorithm:**	Curve Fit	**MW algorithm threshold (%):**	40
**Envelope threshold (%):**	50				

Method Audit Trail

Method audit trail is not printed

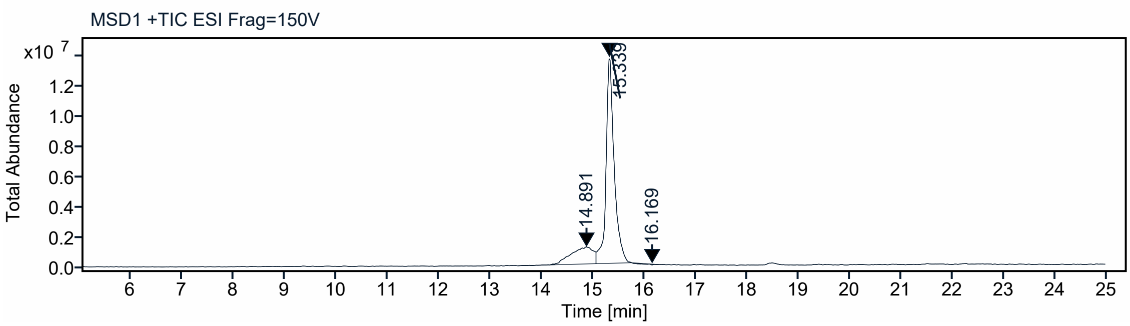

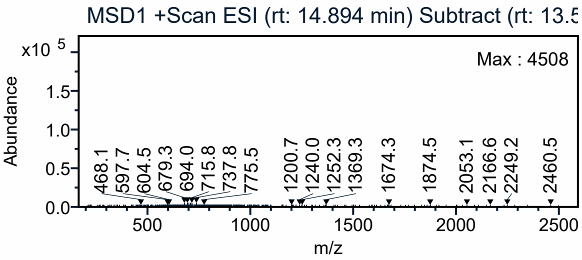
MSD1 +Scan ESI (rt: 14.894 min) Subtract (rt: 13.5
x10 5
Max : 4508
Abundance
m/z
468.1
597.7
604.5
679.3
694.0
715.8
737.8
775.5
1200.7
1240.0
1252.3
1369.3
1674.3
1874.5
2053.1
2166.6
2249.2
2460.5

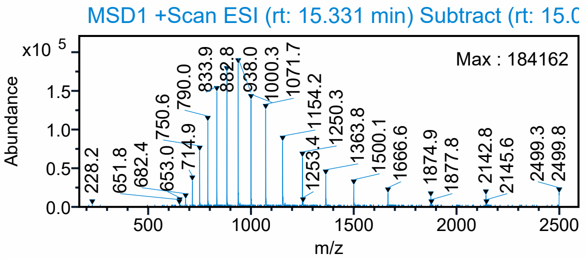
MSD1 +Scan ESI (rt: 15.331 min) Subtract (rt: 15.0
x10 5
Max : 184162
Abundance
m/z
228.2
651.8
682.4
653.0
750.6
714.9
790.0
833.9
882.8
938.0
1000.3
1071.7
1154.2
1253.4
1250.3
1363.8
1500.1
1666.6
1874.9
1877.8
2142.8
2145.6
2499.3
2499.8

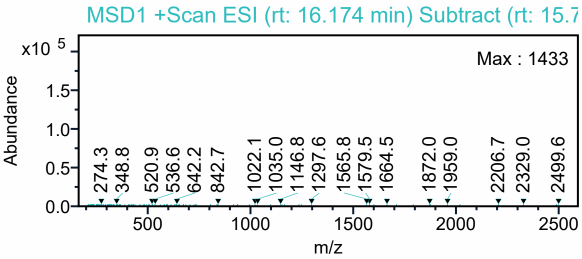
MSD1 +Scan ESI (rt: 16.174 min) Subtract (rt: 15.7
x10 5
Max : 1433
Abundance
m/z
274.3
348.8
520.9
536.6
642.2
842.7
1022.1
1035.0
1146.8
1297.6
1565.8
1579.5
1664.5
1872.0
1959.0
2206.7
2329.0
2499.6

Deconvolution of peak at RT: 15.339

Signal: MSD1 +TIC ESI Frag=150V

Spectrum: MSD1 +Scan ESI (rt: 15.331 min) Subtract (rt: 15.082 min)

No spectra available!

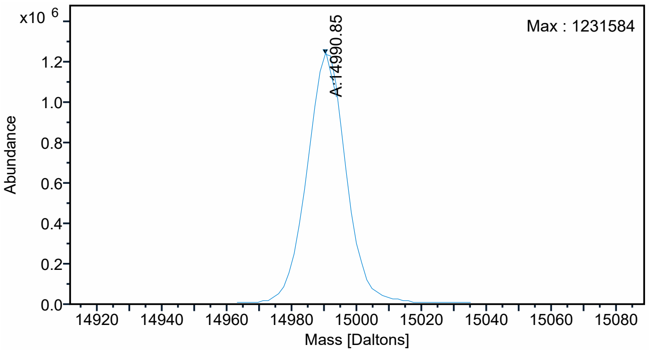

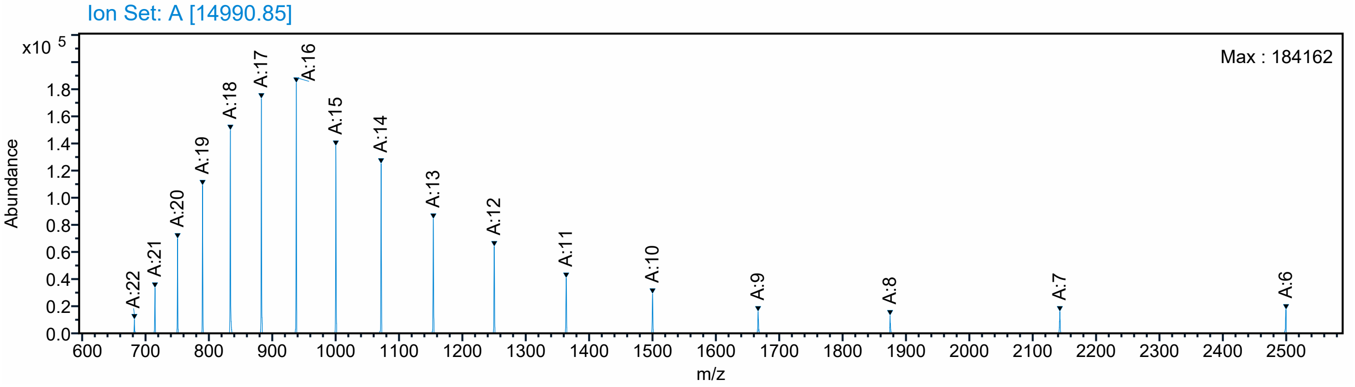

Component	Mass	Absolute Abundance	Relative Abundance (%)	Relative Quantitation (%)
A	14990.85	1231584	100.00	100.00

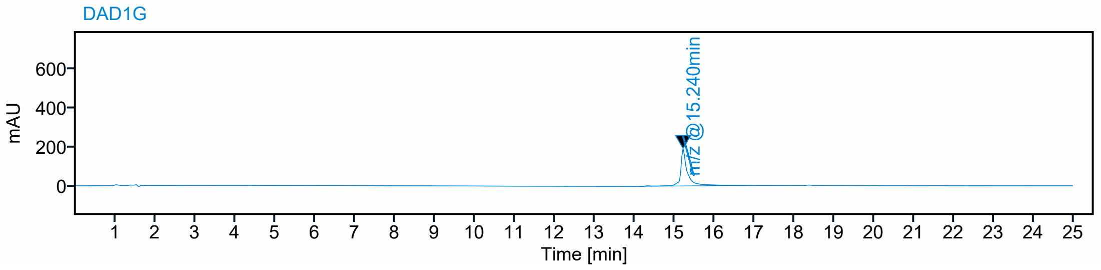

Signal: DAD1G

Name	RT [min]	RF	Area	Peak Area Percent	Group
	15.240		2257.6145	100.00	UNMODIFIED PROTEIN - 100%

Single Injection Report

Signal: MSD1 +TIC ESI Frag=150V

Name	RT [min]	RF	Area	Peak Area Percent	Group
	14.891		34217846.0153	18.52	no protein
	15.339		149575712.9619	80.95	unmodified protein - 100%
	16.169		987520.0906	0.53	no protein

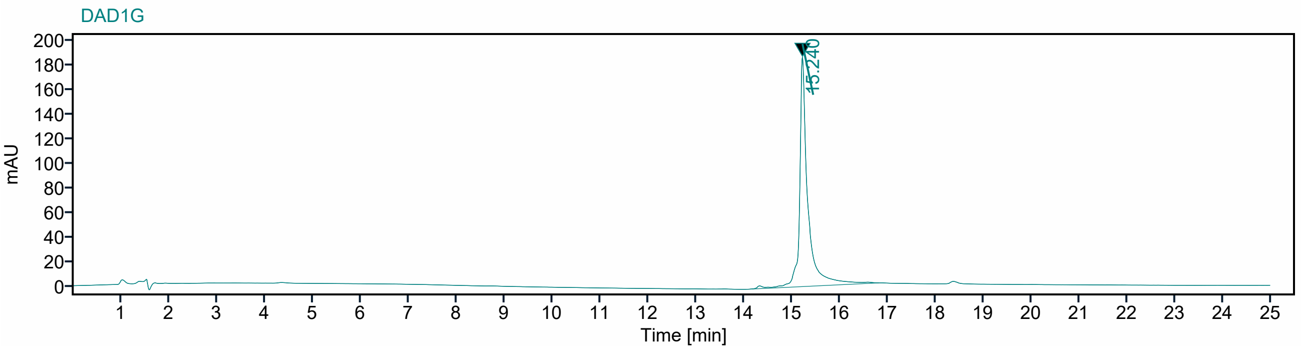

Single Injection Report

**Data file:**	100125-10-75OVER20_PEPTIDES-C3_200M-30695_027.D		
**Sequence Name:**	Chemstatioimports_MRR	**Project Name:**	Walkup Submissions
**Sample name:**	BromoTag_MR109_10Jan25	**Operator:**	Maria Rodriguez
**Instrument:**		**Injection date:**	2025-01-10 22:32:24-08:00
**Inj. volume:**		**Location:**	P2-B-09
**Acq. method:**	10-75OVER20_PEPTIDES-C3_200MZ.M	**Type:**	Sample
**Processing method:**	*Deconvolution Test 2.pmx	**Sample amount:**	
**Manually modified:**	None		

Data Analysis Method: Deconvolution Test 2.pmx
Path: D:\CDSProjects\Walkup Submissions\Results\Chemstatioimports_MRR.rslt
Method parameters are filtered - only a subset is displayed

2 Method Parameters

2.11 MS Spectral Deconvolution Parameters

**Run automatic deconvolution:**	Yes	**Use RT window:**	No	**TIC peak type:**	All peaks
**TIC peak threshold:**	Top (n) peaks	**Top (n) peaks:**	6	**Positive adduct:**	+H
**Negative adduct:**	-H	**Use m/z range:**	No	**Low molecular weight:**	4000
**High molecular weight:**	25000	**Maximum charge:**	40	**Minimum peaks in set:**	3
**Show unmatched peaks:**	No	**MW agreement (0.01%):**	5	**Absolute noise threshold:**	1000
**Relative abundance threshold (%):**	10	**MW algorithm:**	Curve Fit	**MW algorithm threshold (%):**	40
**Envelope threshold (%):**	50				

Method Audit Trail

Method audit trail is not printed

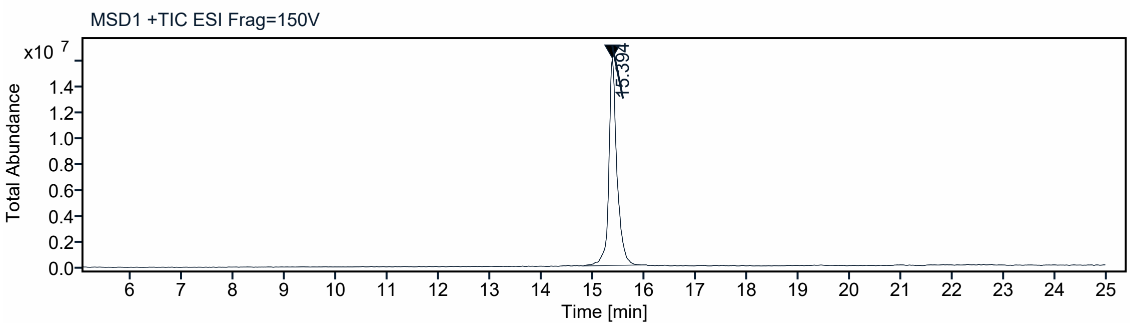

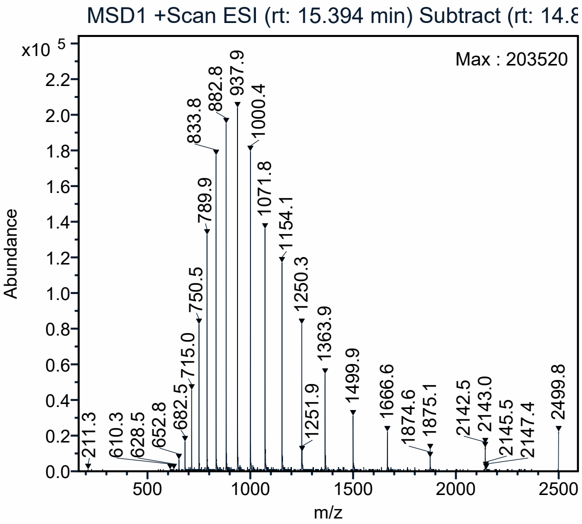

Deconvolution of peak at RT: 15.394

Signal: MSD1 +TIC ESI Frag=150V

Spectrum: MSD1 +Scan ESI (rt: 15.394 min) Subtract (rt: 14.817 min)

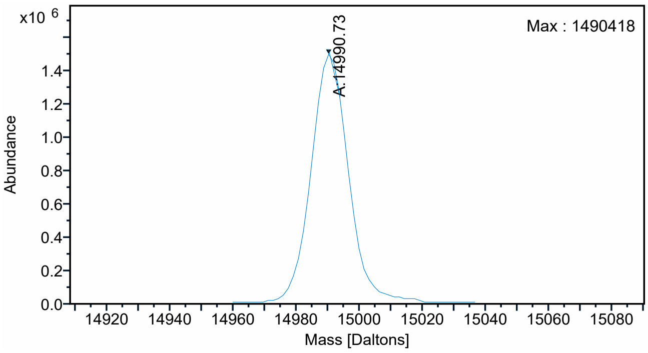

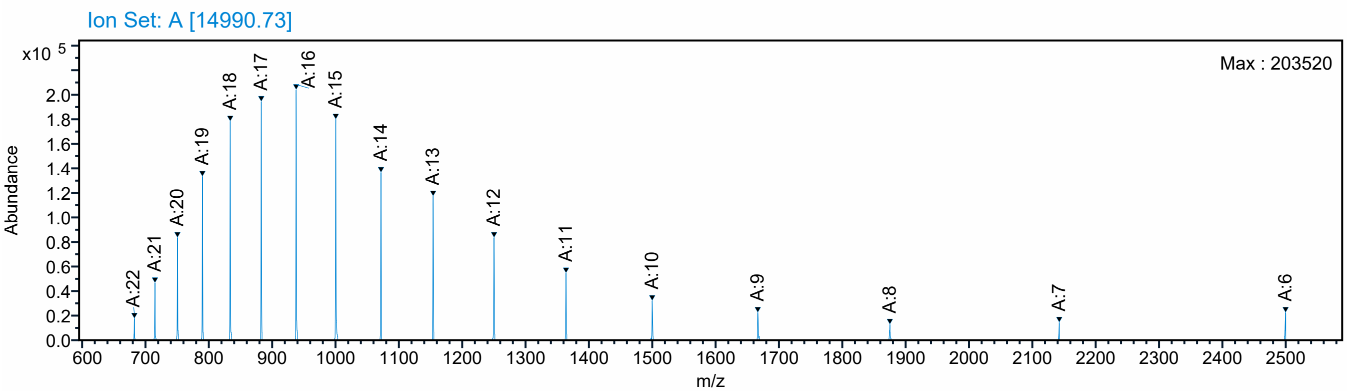

Component	Mass	Absolute Abundance	Relative Abundance (%)	Relative Quantitation (%)
A	14990.73	1490418	100.00	100.00

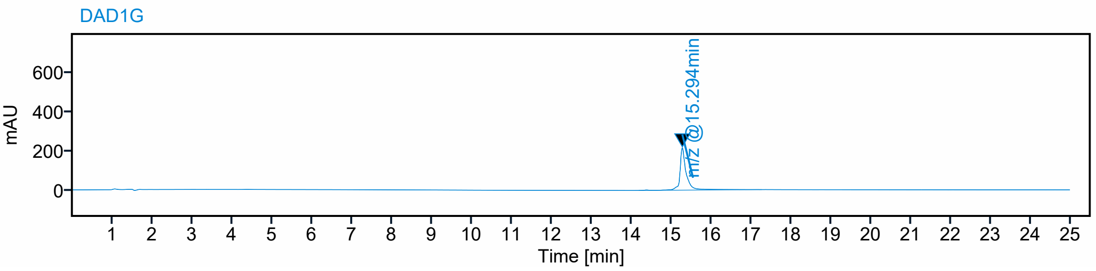

Signal: DAD1G

Name	RT [min]	RF	Area	Peak Area Percent	Group
	15.294		2400.9192	100.00	

Signal: MSD1 +TIC ESI Frag=150V

Name	RT [min]	RF	Area	Peak Area Percent	Group
	15.394		176023830.4585	100.00	

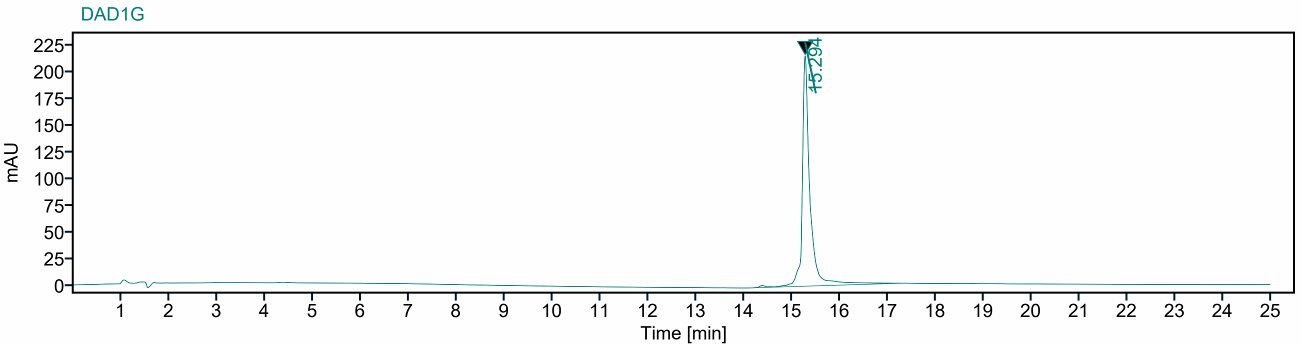
DAD1G
mAU
225
200
175
150
125
100
75
50
25
0
15.294
1 2 3 4 5 6 7 8 9 10 11 12 13 14 15 16 17 18 19 20 21 22 23 24 25
Time [min]

**Data file:**	100125-10-75OVER20_PEPTIDES-C3_200M-30696_028.D		
**Sequence Name:**	Chemstatioimports_MRR	**Project Name:**	Walkup Submissions
**Sample name:**	BromoTag_MR111_10Jan25	**Operator:**	Maria Rodriguez
**Instrument:**		**Injection date:**	2025-01-10 23:02:16-08:00
**Inj. volume:**		**Location:**	P2-C-01
**Acq. method:**	10-75OVER20_PEPTIDES-C3_200MZ.M	**Type:**	Sample
**Processing method:**	*Deconvolution Test 2.pmx	**Sample amount:**	
**Manually modified:**	Manual Integration		

Data Analysis Method: **Deconvolution Test 2.pmx**
Path: **D:\CDSProjects\Walkup Submissions\Results\Chemstatioimports_MRR.rslt**
Method parameters are filtered - only a subset is displayed

2 Method Parameters

2.11 MS Spectral Deconvolution Parameters

**Run automatic deconvolution:**	Yes	**Use RT window:**	No	**TIC peak type:**	All peaks
**TIC peak threshold:**	Top (n) peaks	**Top (n) peaks:**	6	**Positive adduct:**	+H
**Negative adduct:**	-H	**Use m/z range:**	No	**Low molecular weight:**	4000
**High molecular weight:**	25000	**Maximum charge:**	40	**Minimum peaks in set:**	3
**Show unmatched peaks:**	No	**MW agreement (0.01%):**	5	**Absolute noise threshold:**	1000
**Relative abundance threshold (%):**	10	**MW algorithm:**	Curve Fit	**MW algorithm threshold (%):**	40
**Envelope threshold (%):**	50				

Method Audit Trail

Method audit trail is not printed

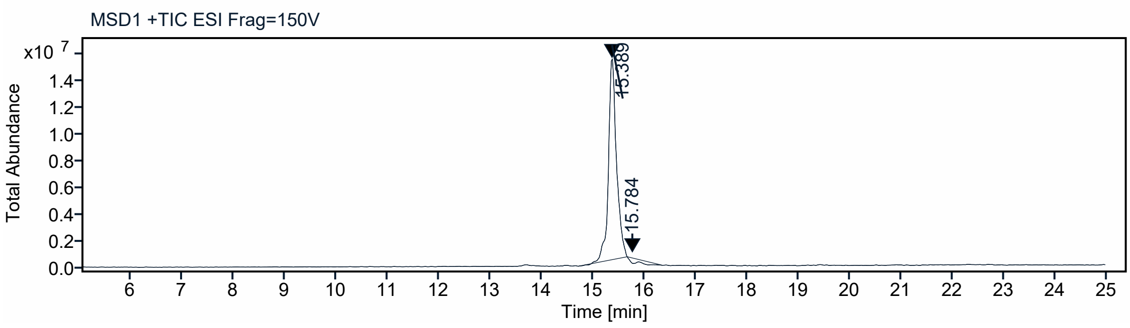

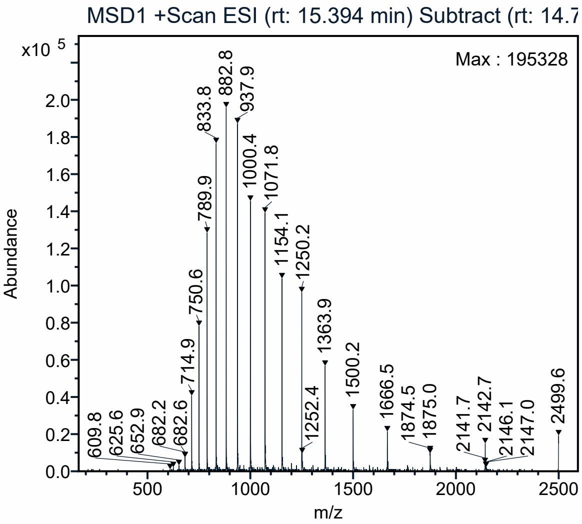

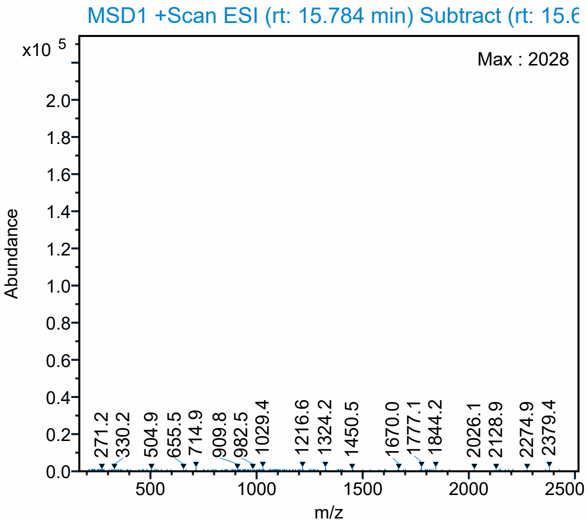

Deconvolution of peak at RT: 15.389

Signal: MSD1 +TIC ESI Frag=150V

Spectrum: MSD1 +Scan ESI (rt: 15.394 min) Subtract (rt: 14.739 min)

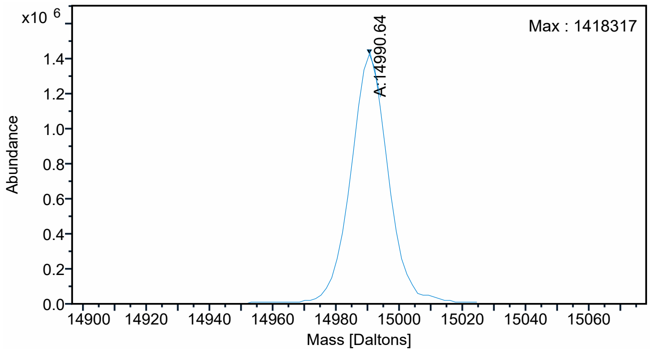

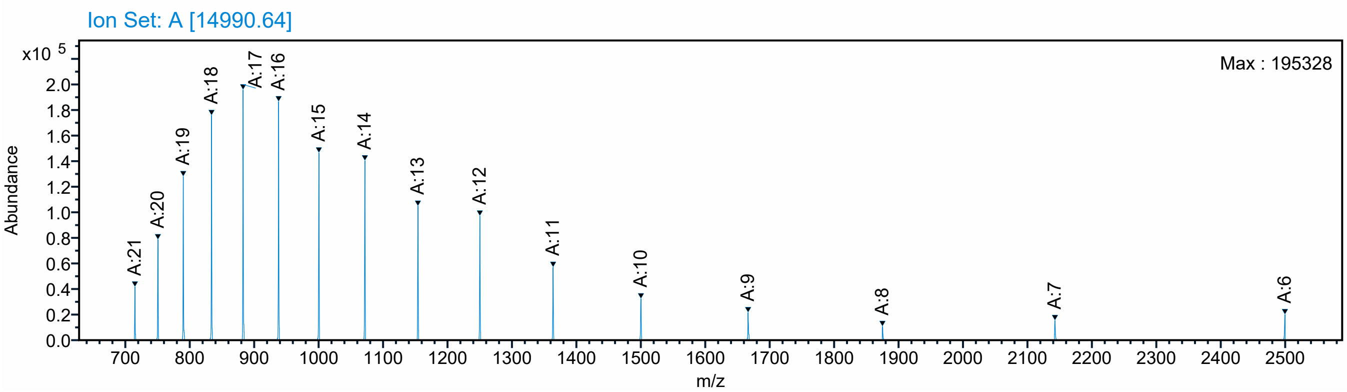

Component	Mass	Absolute Abundance	Relative Abundance (%)	Relative Quantitation (%)
A	14990.64	1418317	100.00	100.00

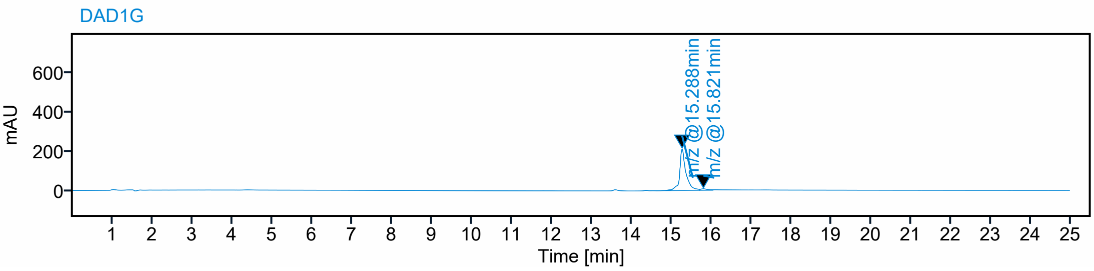

Signal: DAD1G

Name	RT [min]	RF	Area	Peak Area Percent	Group
	15.288		2314.8260	95.67	unmodified protein - 100%
	~~15.821~~		~~104.7998~~	~~4.33~~	no protein detected

Signal: MSD1 +TIC ESI Frag=150V

Name	RT [min]	RF	Area	Peak Area Percent	Group
	15.389		164341623.5468	95.59	unmodified protein - 100%
	~~15.784~~		~~7586950.6516~~	~~4.41~~	no protein detected

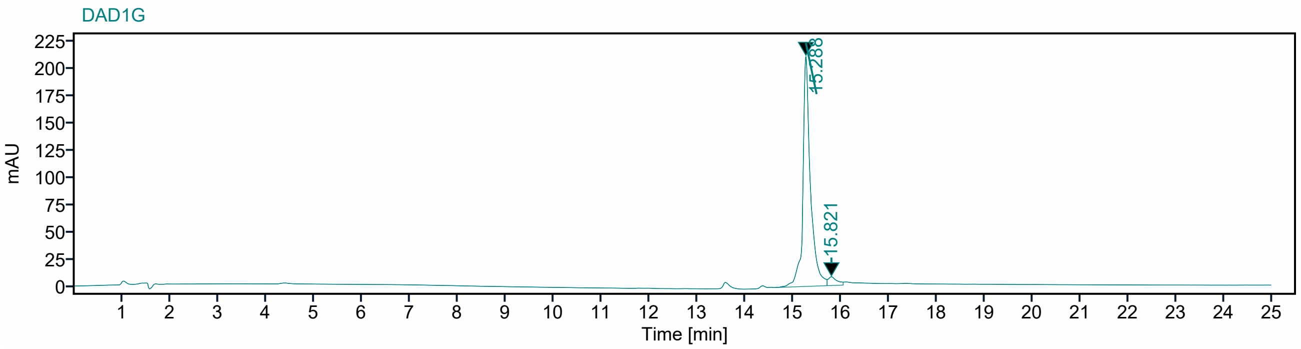
DAD1G
225
200
175
150
125
100
75
50
25
0
mAU
15.288
15.821
1 2 3 4 5 6 7 8 9 10 11 12 13 14 15 16 17 18 19 20 21 22 23 24 25
Time [min]

Single Injection Report

**Data file:**	100125-10-75OVER20_PEPTIDES-C3_200M-30697_029.D		
**Sequence Name:**	Chemstatioimports_MRR	**Project Name:**	Walkup Submissions
**Sample name:**	BromoTag_MR115_10Jan25	**Operator:**	Maria Rodriguez
**Instrument:**		**Injection date:**	2025-01-10 23:32:06-08:00
**Inj. volume:**		**Location:**	P2-C-02
**Acq. method:**	10-75OVER20_PEPTIDES-C3_200MZ.M	**Type:**	Sample
**Processing method:**	*Deconvolution Test 2.pmx	**Sample amount:**	
**Manually modified:**	Manual Integration		

Data Analysis Method: **Deconvolution Test 2.pmx**
Path: **D:\CDSProjects\Walkup Submissions\Results\Chemstatioimports_MRR.rslt**
Method parameters are filtered - only a subset is displayed

2 Method Parameters

2.11 MS Spectral Deconvolution Parameters

**Run automatic deconvolution:**	Yes	**Use RT window:**	No	**TIC peak type:**	All peaks
**TIC peak threshold:**	Top (n) peaks	**Top (n) peaks:**	6	**Positive adduct:**	+H
**Negative adduct:**	-H	**Use m/z range:**	No	**Low molecular weight:**	4000
**High molecular weight:**	25000	**Maximum charge:**	40	**Minimum peaks in set:**	3
**Show unmatched peaks:**	No	**MW agreement (0.01%):**	5	**Absolute noise threshold:**	1000
**Relative abundance threshold (%):**	10	**MW algorithm:**	Curve Fit	**MW algorithm threshold (%):**	40
**Envelope threshold (%):**	50				

Method Audit Trail

Method audit trail is not printed

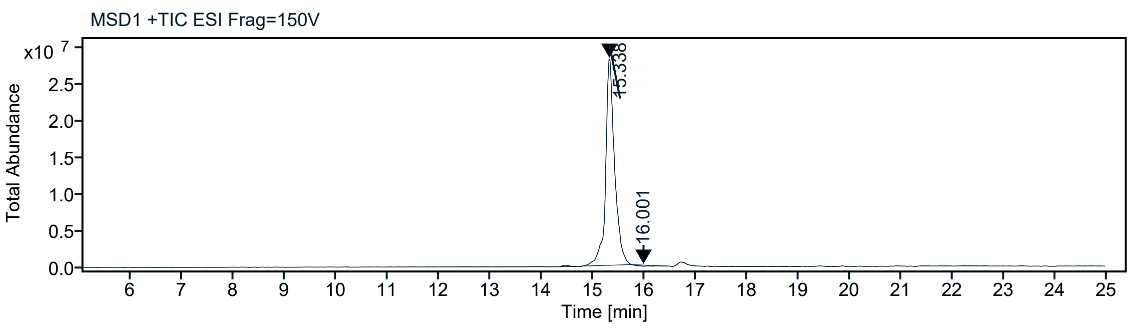

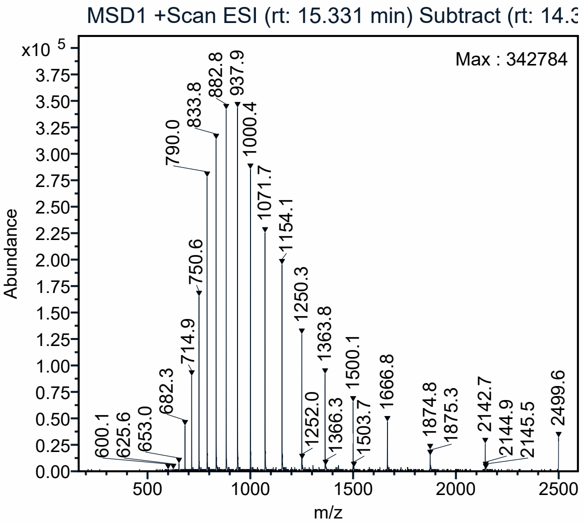

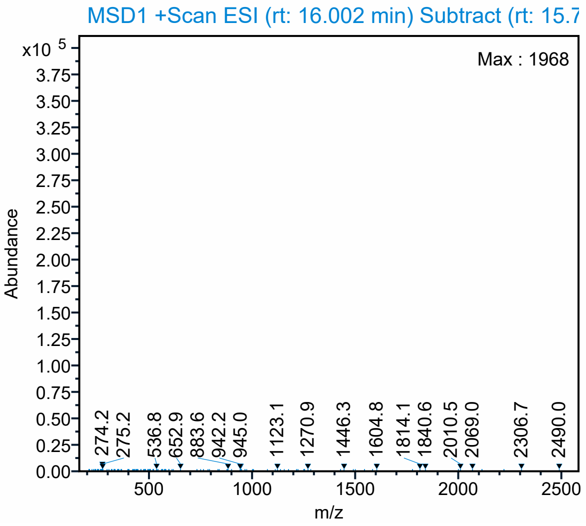

Deconvolution of peak at RT: 15.338

Signal: MSD1 +TIC ESI Frag=150V

Spectrum: MSD1 +Scan ESI (rt: 15.331 min) Subtract (rt: 14.364 min)

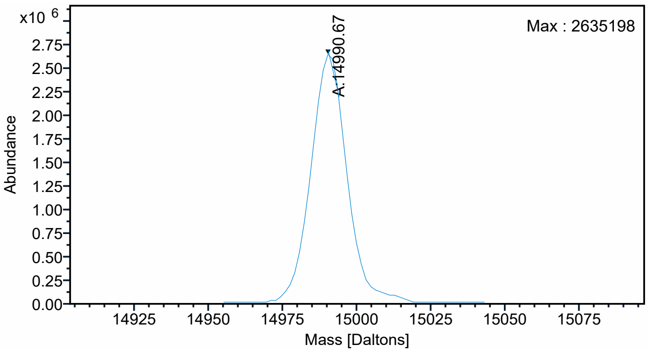

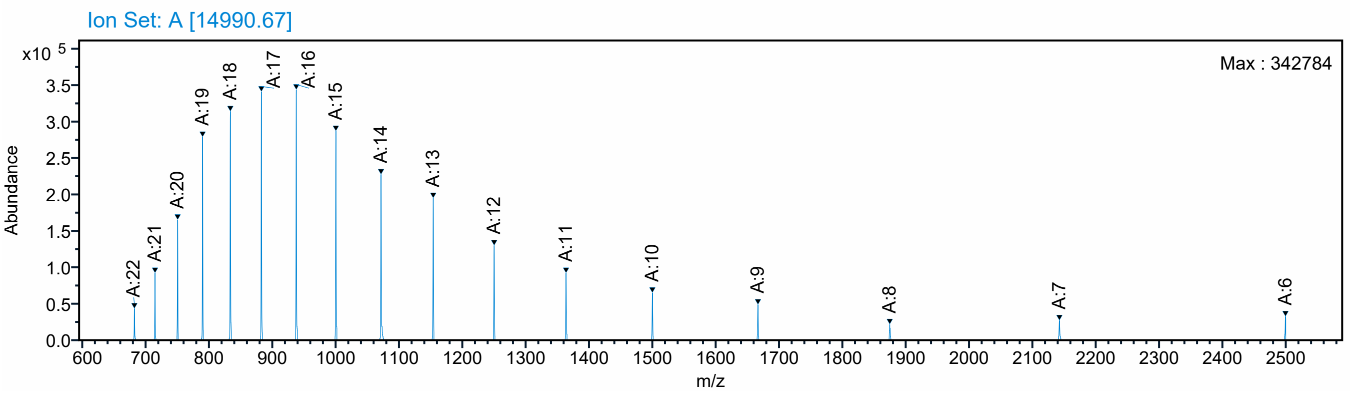

Component	Mass	Absolute Abundance	Relative Abundance (%)	Relative Quantitation (%)
A	14990.67	2635198	100.00	100.00

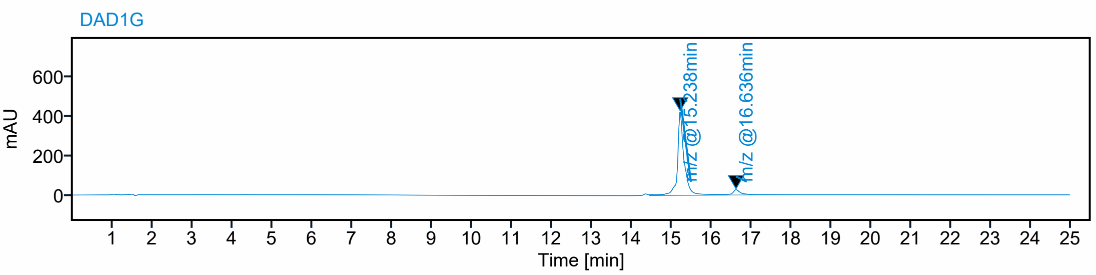

Signal: DAD1G

Name	RT [min]	RF	Area	Peak Area Percent	Group
	15.238		4916.5956	91.61	
	~~16.636~~		~~450.2794~~	~~8.39~~	

Signal: MSD1 +TIC ESI Frag=150V

Name	RT [min]	RF	Area	Peak Area Percent	Group
	15.338		343910473.9652	99.02	
	~~16.001~~		~~3392576.5380~~	~~0.98~~	

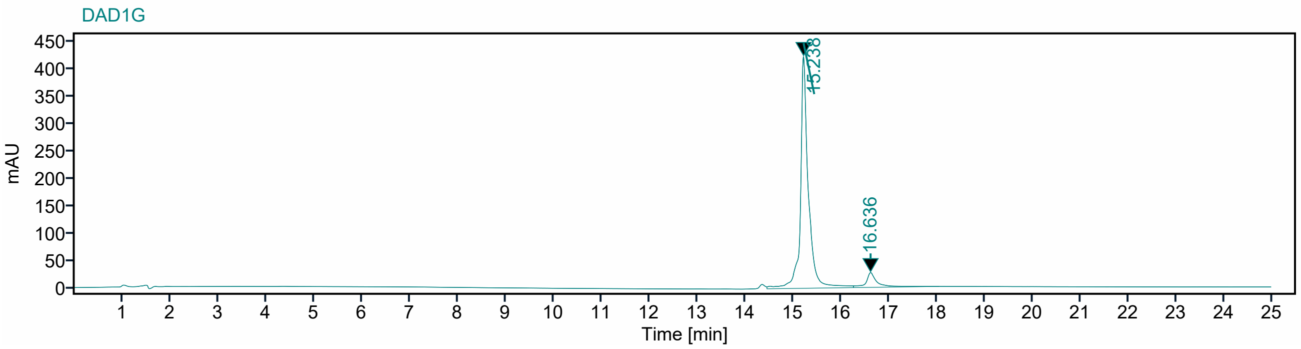
DAD1G
450
400
350
300
250
200
150
100
50
0
mAU
15.238
16.636
1
2
3
4
5
6
7
8
9
10
11
12
13
14
15
16
17
18
19
20
21
22
23
24
25
Time [min]

**Data file:**	100125-10-75OVER20_PEPTIDES-C3_200M-30698_030.D		
**Sequence Name:**	Chemstatioimports_MRR	**Project Name:**	Walkup Submissions
**Sample name:**	BromoTag_MR118_10Jan25	**Operator:**	Maria Rodriguez
**Instrument:**		**Injection date:**	2025-01-11 00:01:56-08:00
**Inj. volume:**		**Location:**	P2-C-03
**Acq. method:**	10-75OVER20_PEPTIDES-C3_200MZ.M	**Type:**	Sample
**Processing method:**	*Deconvolution Test 2.pmx	**Sample amount:**	
**Manually modified:**	None		

Data Analysis Method: **Deconvolution Test 2.pmx**
Path: **D:\CDSProjects\Walkup Submissions\Results\Chemstatioimports_MRR.rslt**
Method parameters are filtered - only a subset is displayed

2 Method Parameters

2.11 MS Spectral Deconvolution Parameters

**Run automatic deconvolution:**	Yes	**Use RT window:**	No	**TIC peak type:**	All peaks
**TIC peak threshold:**	Top (n) peaks	**Top (n) peaks:**	6	**Positive adduct:**	+H
**Negative adduct:**	-H	**Use m/z range:**	No	**Low molecular weight:**	4000
**High molecular weight:**	25000	**Maximum charge:**	40	**Minimum peaks in set:**	3
**Show unmatched peaks:**	No	**MW agreement (0.01%):**	5	**Absolute noise threshold:**	1000
**Relative abundance threshold (%):**	10	**MW algorithm:**	Curve Fit	**MW algorithm threshold (%):**	40
**Envelope threshold (%):**	50				

Method Audit Trail

Method audit trail is not printed

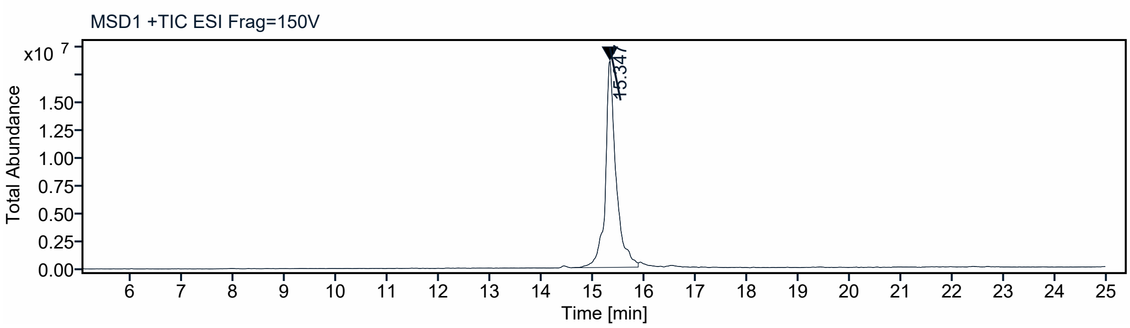

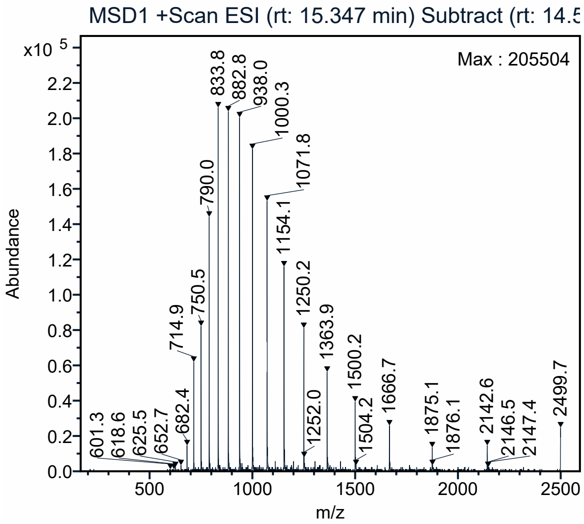

Deconvolution of peak at RT: 15.347

Signal: MSD1 +TIC ESI Frag=150V

Spectrum: MSD1 +Scan ESI (rt: 15.347 min) Subtract (rt: 14.598 min)

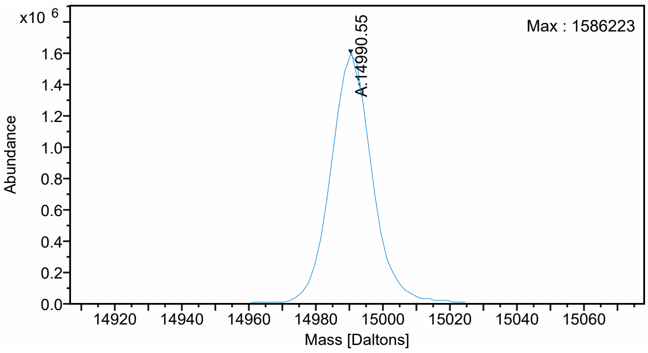

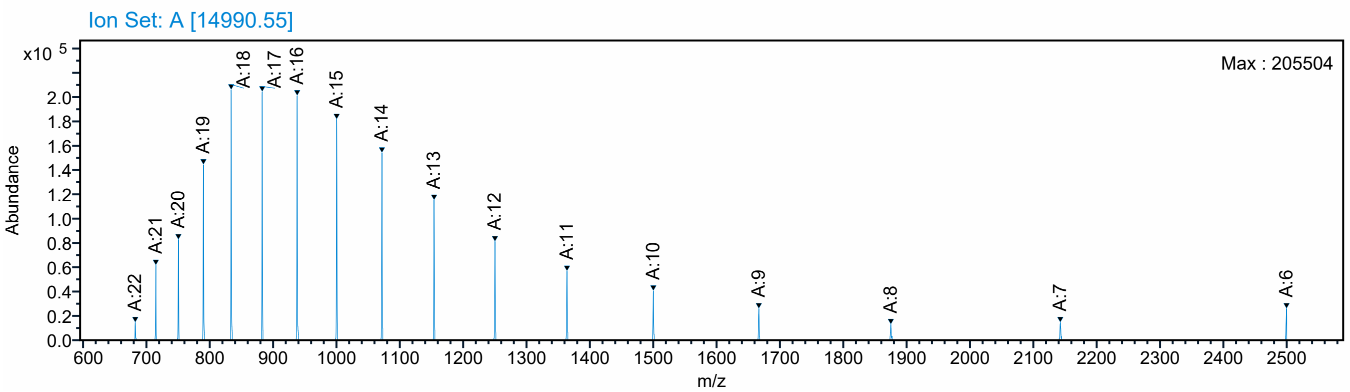

Component	Mass	Absolute Abundance	Relative Abundance (%)	Relative Quantitation (%)
A	14990.55	1586223	100.00	100.00

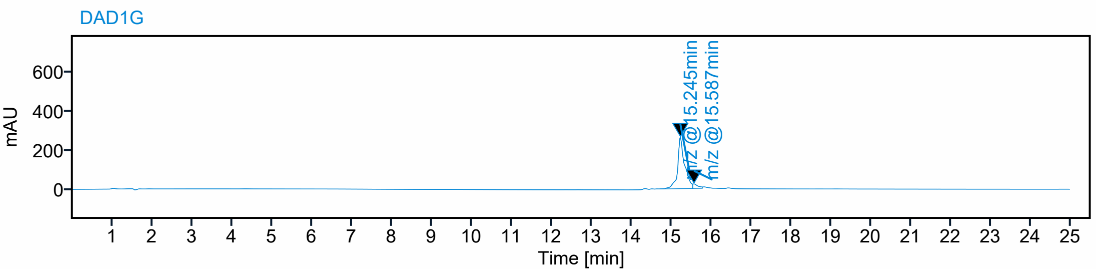

Signal: DAD1G

Name	RT [min]	RF	Area	Peak Area Percent	Group
	15.245		3191.0516	93.84	unmodified protein - 100%
	~~15.587~~		~~209.4297~~	~~6.16~~	

Signal: MSD1 +TIC ESI Frag=150V

Name	RT [min]	RF	Area	Peak Area Percent	Group
	15.347		266277679.5852	100.00	

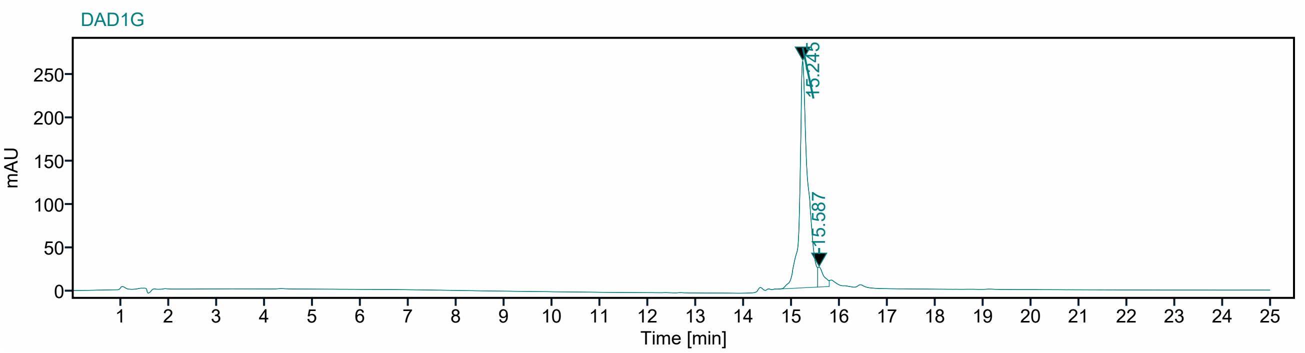
DAD1G
mAU
250
200
150
100
50
0
15.245
15.587
1
2
3
4
5
6
7
8
9
10
11
12
13
14
15
16
17
18
19
20
21
22
23
24
25
Time [min]

Single Injection Report

**Data file:**	100125-10-75OVER20_PEPTIDES-C3_200M-30699_031.D		
**Sequence Name:**	Chemstatioimports_MRR	**Project Name:**	Walkup Submissions
**Sample name:**	BromoTag_MR117_10Jan25	**Operator:**	Maria Rodriguez
**Instrument:**		**Injection date:**	2025-01-11 00:31:48-08:00
**Inj. volume:**		**Location:**	P2-C-04
**Acq. method:**	10-75OVER20_PEPTIDES-C3_200MZ.M	**Type:**	Sample
**Processing method:**	*Deconvolution Test 2.pmx	**Sample amount:**	
**Manually modified:**	Manual Integration		

Data Analysis Method: Deconvolution Test 2.pmx
Path: D:\CDSProjects\Walkup Submissions\Results\Chemstatioimports_MRR.rslt
Method parameters are filtered - only a subset is displayed

2 Method Parameters

2.11 MS Spectral Deconvolution Parameters

**Run automatic deconvolution:**	Yes	**Use RT window:**	No	**TIC peak type:**	All peaks
**TIC peak threshold:**	Top (n) peaks	**Top (n) peaks:**	6	**Positive adduct:**	+H
**Negative adduct:**	-H	**Use m/z range:**	No	**Low molecular weight:**	4000
**High molecular weight:**	25000	**Maximum charge:**	40	**Minimum peaks in set:**	3
**Show unmatched peaks:**	No	**MW agreement (0.01%):**	5	**Absolute noise threshold:**	1000
**Relative abundance threshold (%):**	10	**MW algorithm:**	Curve Fit	**MW algorithm threshold (%):**	40
**Envelope threshold (%):**	50				

Method Audit Trail

Method audit trail is not printed

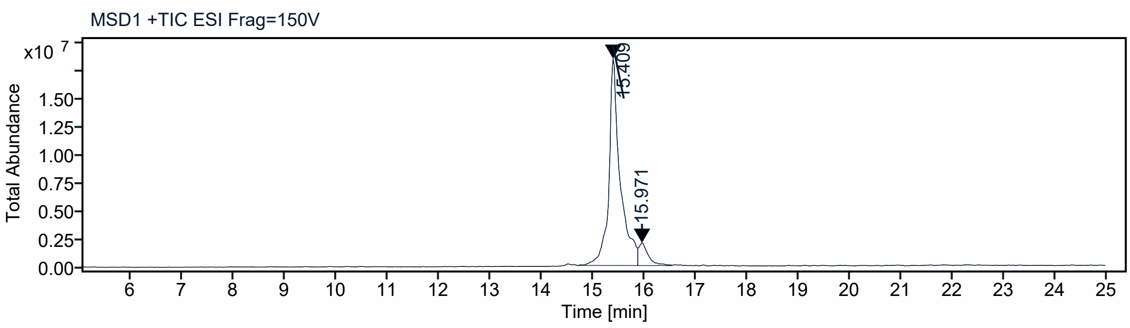

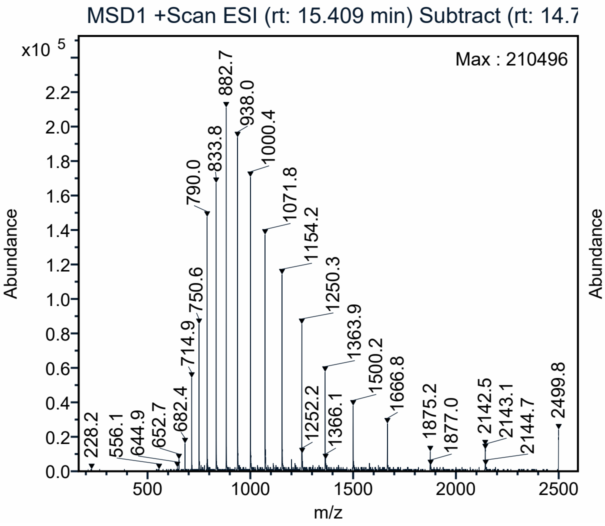

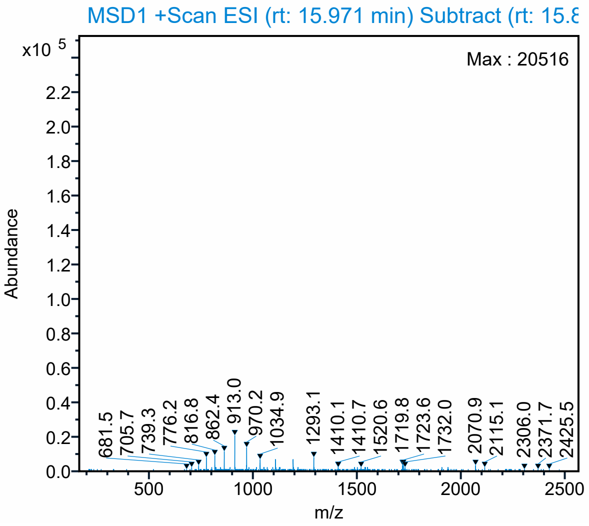

Deconvolution of peak at RT: 15.409

Signal: MSD1 +TIC ESI Frag=150V

Spectrum: MSD1 +Scan ESI (rt: 15.409 min) Subtract (rt: 14.723 min)

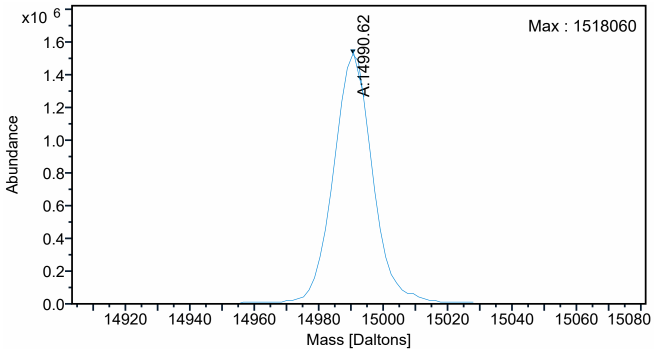

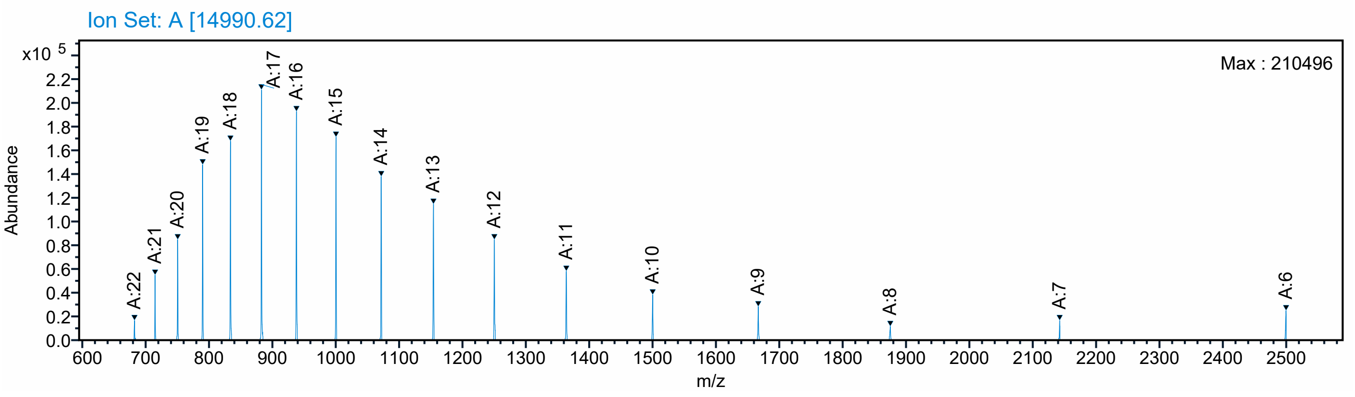

Component	Mass	Absolute Abundance	Relative Abundance (%)	Relative Quantitation (%)
A	14990.62	1518060	100.00	100.00

Single Injection Report

Deconvolution of peak at RT: 15.971

Signal: MSD1 +TIC ESI Frag=150V

Spectrum: MSD1 +Scan ESI (rt: 15.971 min) Subtract (rt: 15.893 min)

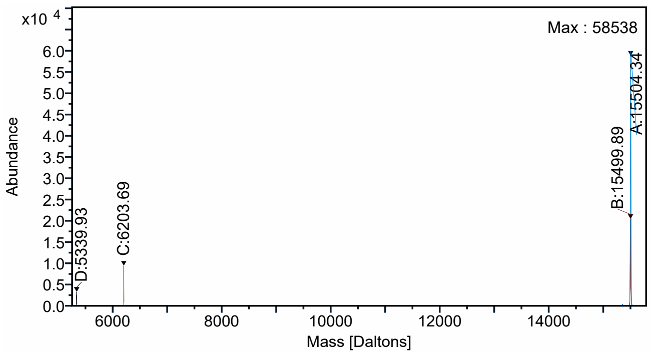

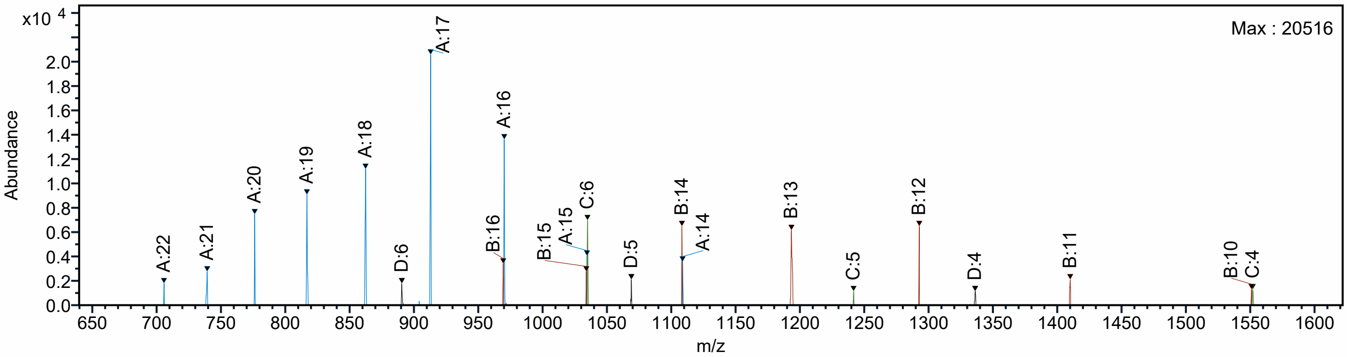

Component	Mass	Absolute Abundance	Relative Abundance (%)	Relative Quantitation (%)
A	15504.34	58538	100.00	64.39
B	15499.89	20100	34.34	22.11
C	6203.69	9095	15.54	10.00
D	5339.93	3173	5.42	3.49

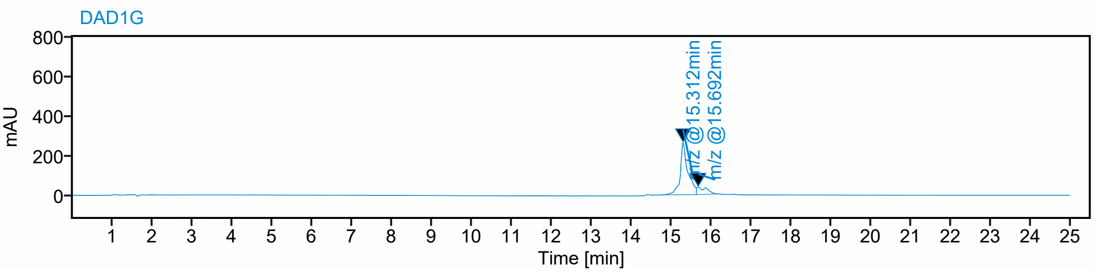

Signal: DAD1G

Name	RT [min]	RF	Area	Peak Area Percent	Group
	15.312		3494.5166	84.34	unmodified protein - 90%
	15.692		648.7887	15.66	double peak uv (MS peak used for % modif)

Signal: MSD1 +TIC ESI Frag=150V

Name	RT [min]	RF	Area	Peak Area Percent	Group
	15.409		280751938.9210	91.39	unmodified protein - 90%
	15.971		26444425.8094	8.61	modified protein 10%

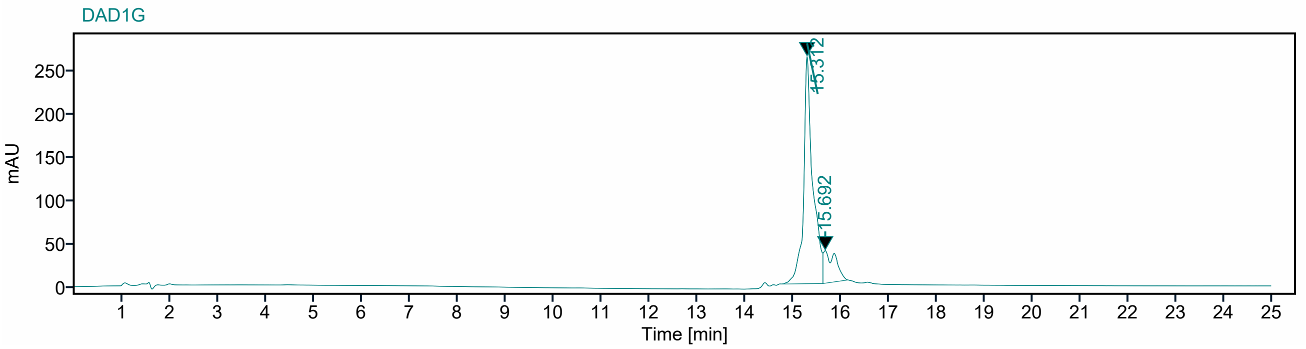
DAD1G
mAU
250
200
150
100
50
0
15.312
15.692
1 2 3 4 5 6 7 8 9 10 11 12 13 14 15 16 17 18 19 20 21 22 23 24 25
Time [min]

Single Injection Report

**Data file:**	100125-10-75OVER20_PEPTIDES-C3_200M-30700_032.D		
**Sequence Name:**	Chemstatioimports_MRR	**Project Name:**	Walkup Submissions
**Sample name:**	BromoTag_MR119_10Jan25	**Operator:**	Maria Rodriguez
**Instrument:**		**Injection date:**	2025-01-11 01:01:39-08:00
**Inj. volume:**		**Location:**	P2-C-05
**Acq. method:**	10-75OVER20_PEPTIDES-C3_200MZ.M	**Type:**	Sample
**Processing method:**	*Deconvolution Test 2.pmx	**Sample amount:**	
**Manually modified:**	Manual Integration		

Data Analysis Method: **Deconvolution Test 2.pmx**
Path: **D:\CDSProjects\Walkup Submissions\Results\Chemstatioimports_MRR.rslt**
Method parameters are filtered - only a subset is displayed

2 Method Parameters

2.11 MS Spectral Deconvolution Parameters

**Run automatic deconvolution:**	Yes	**Use RT window:**	No	**TIC peak type:**	All peaks
**TIC peak threshold:**	Top (n) peaks	**Top (n) peaks:**	6	**Positive adduct:**	+H
**Negative adduct:**	-H	**Use m/z range:**	No	**Low molecular weight:**	4000
**High molecular weight:**	25000	**Maximum charge:**	40	**Minimum peaks in set:**	3
**Show unmatched peaks:**	No	**MW agreement (0.01%):**	5	**Absolute noise threshold:**	1000
**Relative abundance threshold (%):**	10	**MW algorithm:**	Curve Fit	**MW algorithm threshold (%):**	40
**Envelope threshold (%):**	50				

Method Audit Trail

Method audit trail is not printed

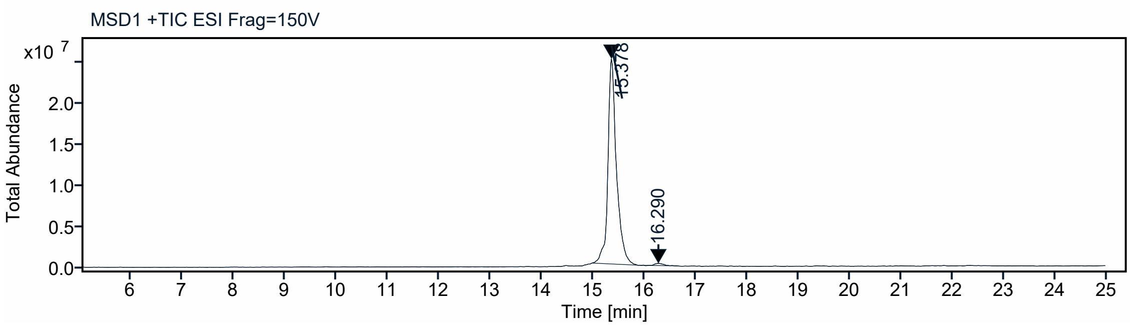

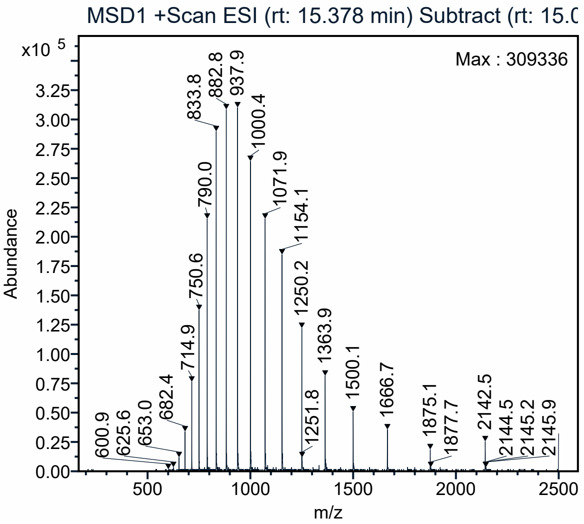

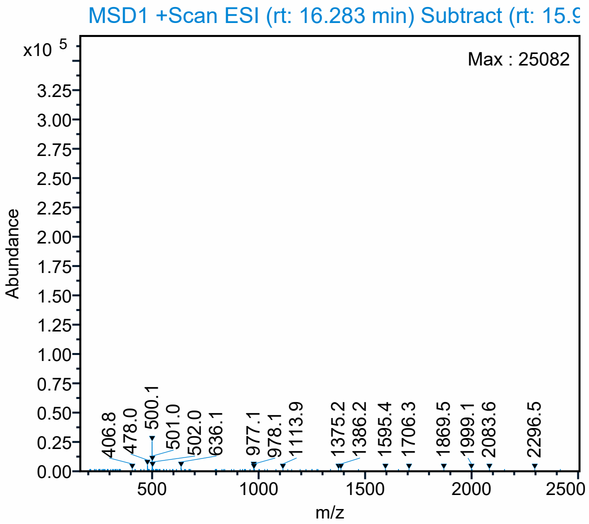

Deconvolution of peak at RT: 15.378

Signal: MSD1 +TIC ESI Frag=150V

Spectrum: MSD1 +Scan ESI (rt: 15.378 min) Subtract (rt: 15.004 min)

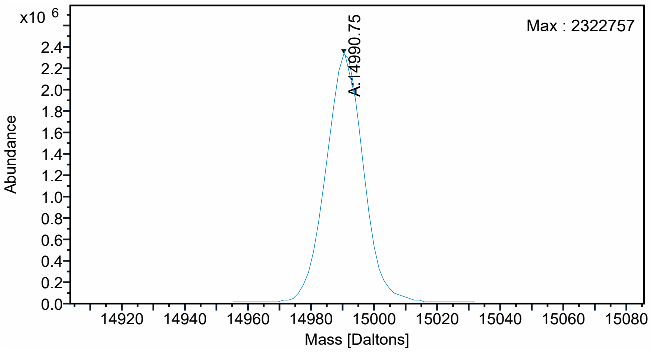

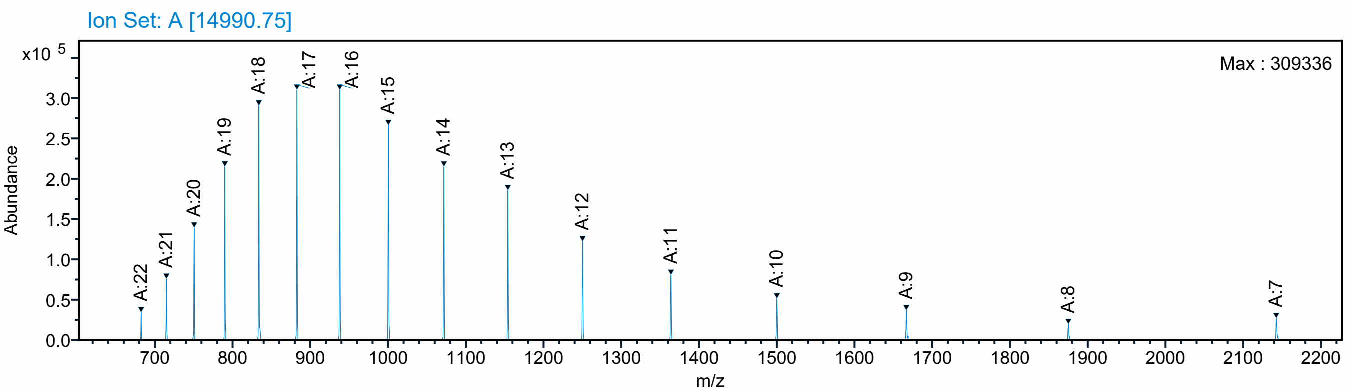

Component	Mass	Absolute Abundance	Relative Abundance (%)	Relative Quantitation (%)
A	14990.75	2322757	100.00	100.00

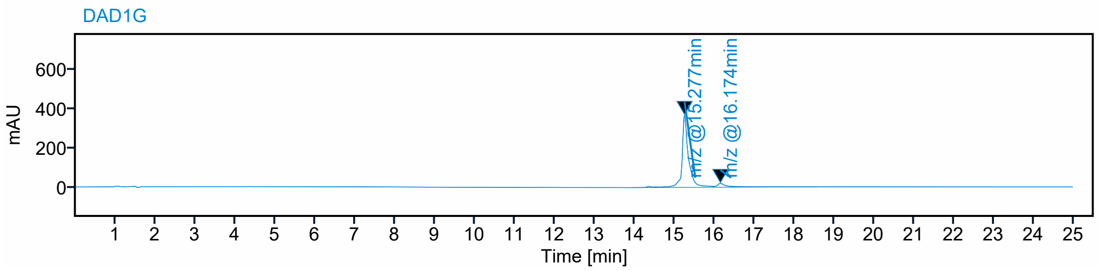

Signal: DAD1G

Name	RT [min]	RF	Area	Peak Area Percent	Group
	15.277		4149.9341	90.18	unmodified protein - 100%
	~~16.174~~		~~451.8384~~	~~9.82~~	no protein detected

Signal: MSD1 +TIC ESI Frag=150V

Name	RT [min]	RF	Area	Peak Area Percent	Group
	15.378		289114943.3952	98.93	unmodified protein - 100%
	~~16.290~~		~~3129969.9724~~	~~1.07~~	no protein detected

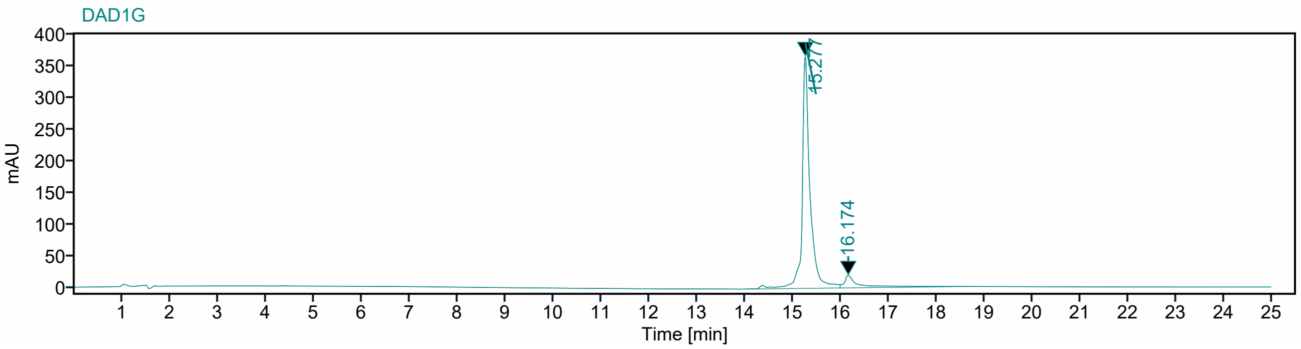
DAD1G
mAU
400
350
300
250
200
150
100
50
0
15.277
16.174
1
2
3
4
5
6
7
8
9
10
11
12
13
14
15
16
17
18
19
20
21
22
23
24
25
Time [min]

Data File D:\EZXDATA\MARIARODRIGUEZ\12-24\121224-10-75OVER20_PEPTIDES-C3-30636.D
Sample Name: Brd4BD2_74_dmso_12dec24

===

```
Acq. Operator   : Maria Rodriguez
Acq. Instrument : INSTRUMENT 1                    Location : P1-B-04
Injection Date  : 12/12/2024 5:36:49 PM                Inj :    1
                                                Inj Volume : 10.000 µl
Acq. Method     : C:\CHEM32\1\METHODS\10-75OVER20_PEPTIDE
Last changed    : 12/12/2024 5:35:48 PM by Maria Rodriguez
                  (modified after loading)
Analysis Method : C:\CHEM32\1\METHODS\10-75OVER20_PEPTIDES-5UL-C3.M
Last changed    : 12/19/2024 5:14:30 PM by Liam Hales
                  (modified after loading)
Sample Info     : Easy-Access Method: '10-75over20_C3'

Additional Info : Peak(s) manually integrated
```

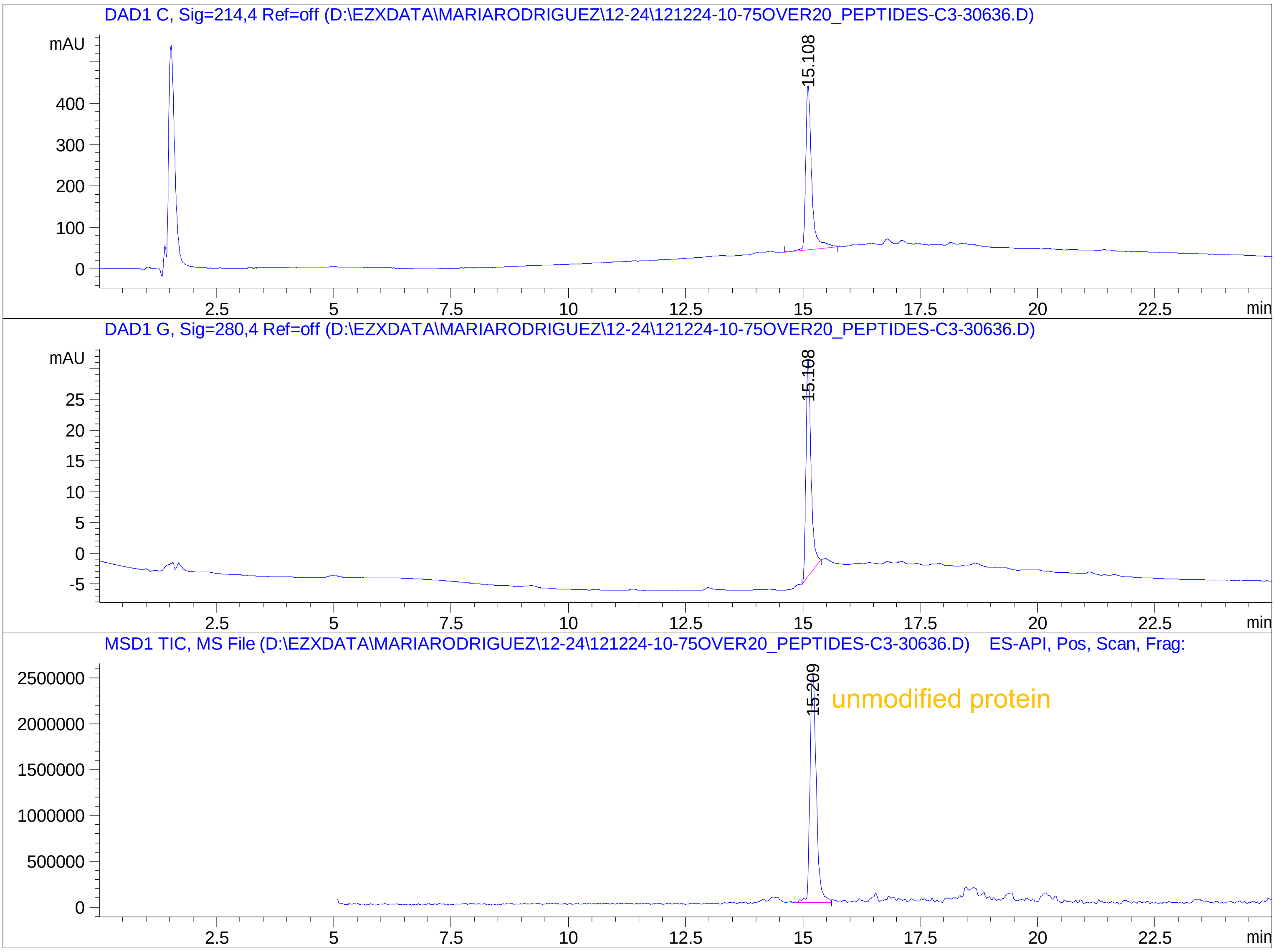

===

Area Percent Report

===

```
Sorted By             :      Signal
Multiplier            :      1.0000
Dilution              :      1.0000
Sample Amount:               :      15.00000  [ng/ul]   (not used in calc.)
Use Multiplier & Dilution Factor with ISTDs
```

Signal 1: DAD1 C, Sig=214,4 Ref=off

Peak #	RetTime [min]	Type	Width [min]	Area [mAU*s]	Height [mAU]	Area %
1	15.108	BV	0.1353	3517.32910	397.31027	100.0000
Totals :				3517.32910	397.31027	

Signal 2: DAD1 G, Sig=280,4 Ref=off

Peak #	RetTime [min]	Type	Width [min]	Area [mAU*s]	Height [mAU]	Area %
1	15.108	BB	0.1158	265.31793	35.27115	100.0000
Totals :				265.31793	35.27115	

Signal 3: MSD1 TIC, MS File

Peak #	RetTime [min]	Type	Width [min]	Area	Height	Area %
1	15.209	BV	0.1471	2.35054e7	2.48875e6	100.0000
Totals :				2.35054e7	2.48875e6	

===

*** End of Report ***

Spectrum Deconvolution
Data File D:\EZXDATA\MARIARODRIGUEZ\12-24\121224-10-75OVER20_PEPTIDES-C3-30636.D
Sample Name: Brd4BD2_74_dmso_12dec24

```
    Easy-Access Method: '10-75over20_C3'
    =====================================================================
    Acq. Operator   : Maria Rodriguez
    Acq. Instrument : INSTRUMENT 1                    Location : P1-B-04
    Injection Date  : 12/12/2024 5:36:49 PM                Inj :   1
                                                    Inj Volume : 10.000 µl
    Acq. Method     : C:\CHEM32\1\METHODS\10-75OVER20_PEPTIDE
    Last changed    : 12/12/2024 5:35:48 PM by Maria Rodriguez
                      (modified after loading)
    Analysis Method : C:\CHEM32\1\METHODS\10-75OVER20_PEPTIDES-5UL-C3.M
    Last changed    : 12/19/2024 5:14:28 PM by Liam Hales
                      (modified after loading)
    Sample Info     : Easy-Access Method: '10-75over20_C3'


    =====================================================================
                          Deconvolution Parameters
    =====================================================================

    Adduct Ion(Positive): +H, 1.0079 Da
    Adduct Ion(Negative):  , 0.0000 Da
    Low MW:               500
    DeconvStartChgMaximum Charge:       50
    Minimum Peaks in Set: 3
    Retain Residual:      No
    Ion PWHH:             0.6 Da
    MW Agreement:         0.05 %
    Noise Cutoff:         1000 counts
    Abundance Cutoff:     10 %
    MW Assign:            Curve fit
    MW Assign Cutoff:     40 %
    Envelope Cutoff:      50 %
```

Spectrum Deconvolution
Data File D:\EZXDATA\MARIARODRIGUEZ\12-24\121224-10-75OVER20_PEPTIDES-C3-30636.D
Sample Name: Brd4BD2_74_dmso_12dec24

Deconvolution of Spectrum # 1 @ 14.885 - 15.706 min

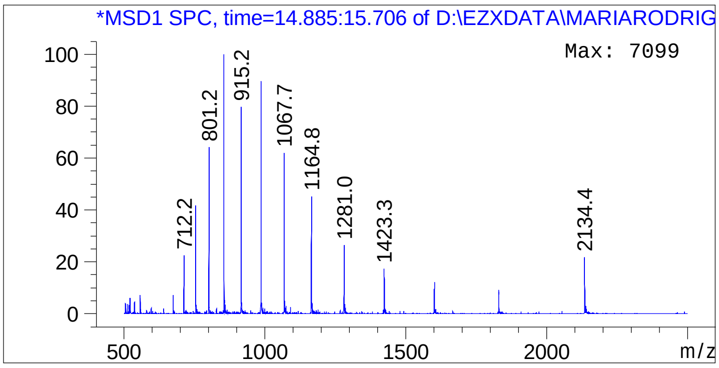

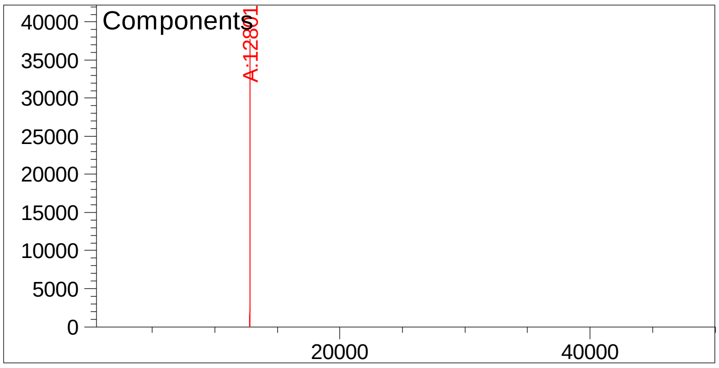

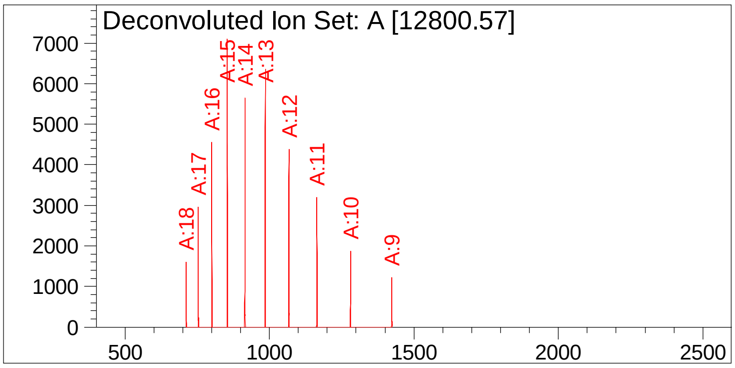

Component	Molecular Weight	Absolute Abundance	Relative Abundance
A	12800.57	37734	100.00

*** End of Report ***

===

Acq. Operator : Maria Rodriguez
Acq. Instrument : INSTRUMENT 1 Location : P1-B-05
Injection Date : 12/12/2024 6:06:46 PM Inj : 1
Inj Volume : 10.000 µl
Acq. Method : C:\CHEM32\1\METHODS\10-75OVER20_PEPTIDE
Last changed : 12/12/2024 6:05:50 PM by Maria Rodriguez
(modified after loading)
Analysis Method : C:\CHEM32\1\METHODS\10-75OVER20_PEPTIDES-5UL-C3.M
Last changed : 12/19/2024 5:14:30 PM by Liam Hales
(modified after loading)
Sample Info : Easy-Access Method: '10-75over20_C3'

Additional Info : Peak(s) manually integrated

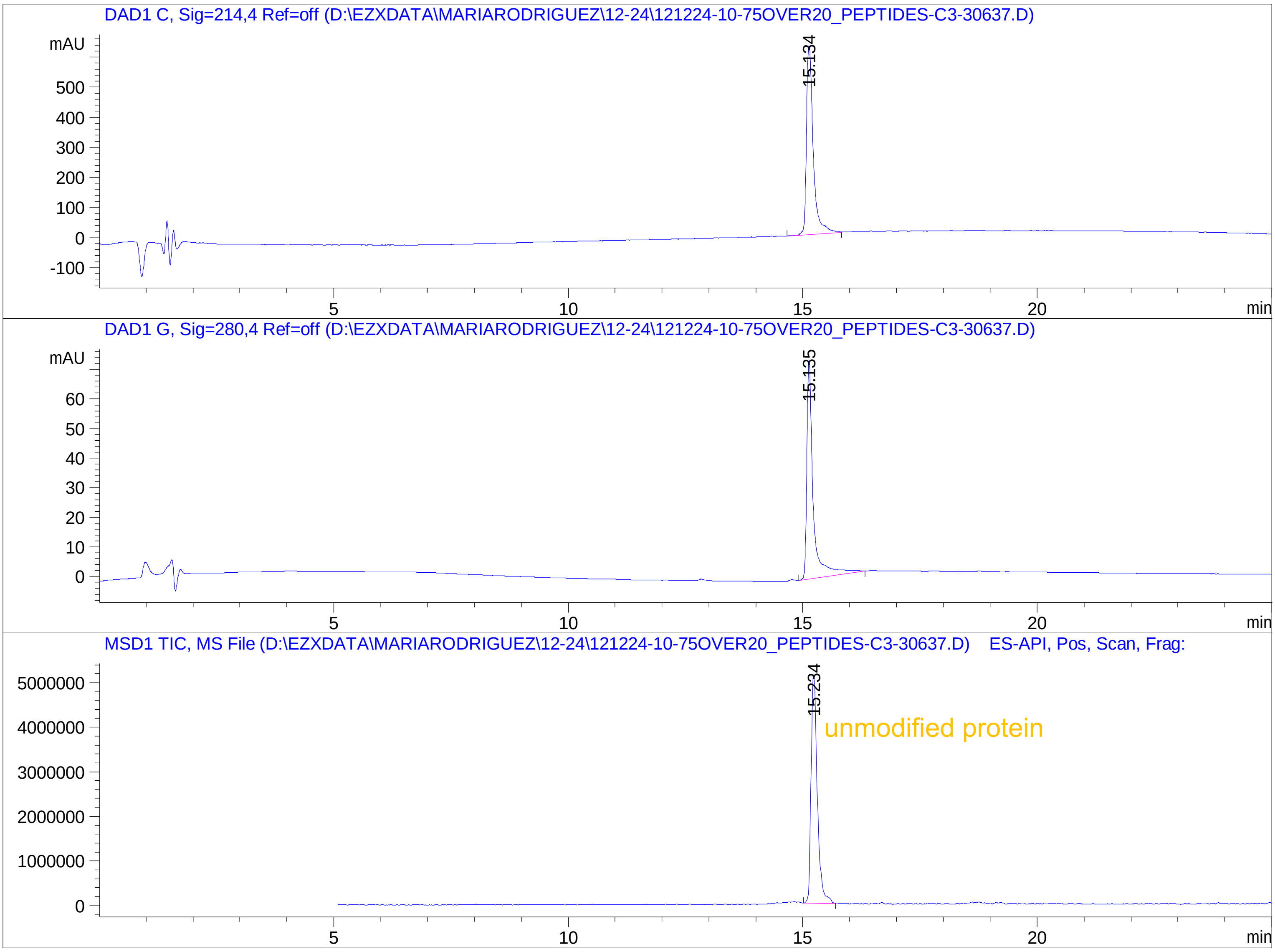

===

Area Percent Report

===

Sorted By : Signal
Multiplier : 1.0000
Dilution : 1.0000
Sample Amount: : 15.00000 [ng/ul] (not used in calc.)
Use Multiplier & Dilution Factor with ISTDs

Signal 1: DAD1 C, Sig=214,4 Ref=off

Peak #	RetTime [min]	Type	Width [min]	Area [mAU*s]	Height [mAU]	Area %
1	15.134	BV	0.1556	6325.35010	627.66064	100.0000

Totals : 6325.35010 627.66064

Signal 2: DAD1 G, Sig=280,4 Ref=off

Peak #	RetTime [min]	Type	Width [min]	Area [mAU*s]	Height [mAU]	Area %
1	15.135	BB	0.1385	701.99249	74.13409	100.0000

Totals : 701.99249 74.13409

Signal 3: MSD1 TIC, MS File

Peak #	RetTime [min]	Type	Width [min]	Area	Height	Area %
1	15.234	BV	0.1406	4.77170e7	5.16528e6	100.0000

Totals : 4.77170e7 5.16528e6

*** End of Report ***

```
Easy-Access Method: '10-75over20_C3'
=====================================================================
Acq. Operator   : Maria Rodriguez
Acq. Instrument : INSTRUMENT 1                    Location : P1-B-05
Injection Date  : 12/12/2024 6:06:46 PM                Inj :   1
                                              Inj Volume : 10.000 µl
Acq. Method     : C:\CHEM32\1\METHODS\10-75OVER20_PEPTIDE
Last changed    : 12/12/2024 6:05:50 PM by Maria Rodriguez
                  (modified after loading)
Analysis Method : C:\CHEM32\1\METHODS\10-75OVER20_PEPTIDES-5UL-C3.M
Last changed    : 12/19/2024 5:16:43 PM by Liam Hales
                  (modified after loading)
Sample Info     : Easy-Access Method: '10-75over20_C3'

Additional Info : Peak(s) manually integrated

=====================================================================
                      Deconvolution Parameters
=====================================================================

Adduct Ion(Positive): +H, 1.0079 Da
Adduct Ion(Negative):  , 0.0000 Da
Low MW:               500
DeconvStartChgMaximum Charge:       50
Minimum Peaks in Set: 3
Retain Residual:      No
Ion PWHH:             0.6 Da
MW Agreement:         0.05 %
Noise Cutoff:         1000 counts
Abundance Cutoff:     10 %
MW Assign:            Curve fit
MW Assign Cutoff:     40 %
Envelope Cutoff:      50 %
```

Data File D:\EZXDATA\MARIARODRIGUEZ\12-24\121224-10-75OVER20_PEPTIDES-C3-30637.D
Sample Name: Brd4BD2_74_ETJQ1_12dec24

Deconvolution of Spectrum # 1 @ 14.324 - 16.896 min

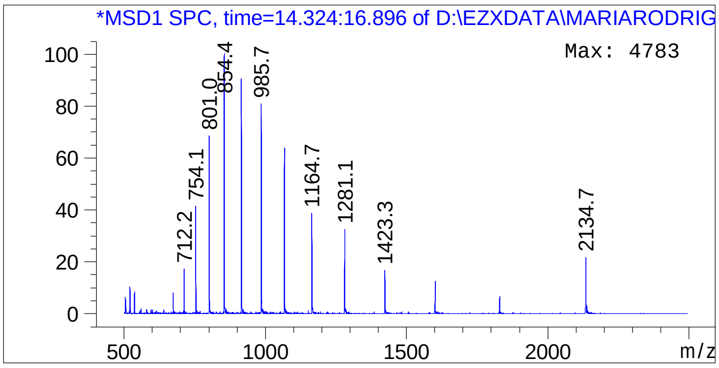

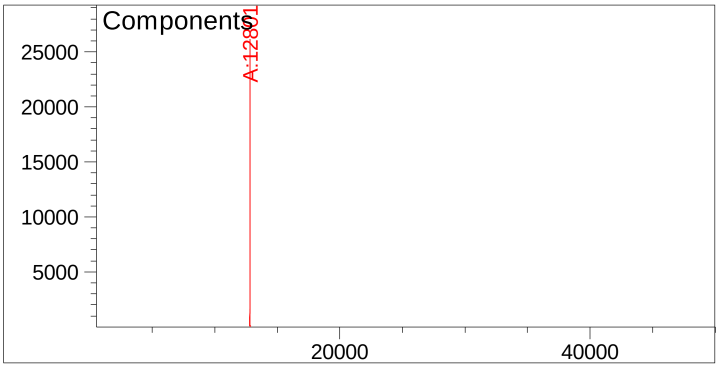

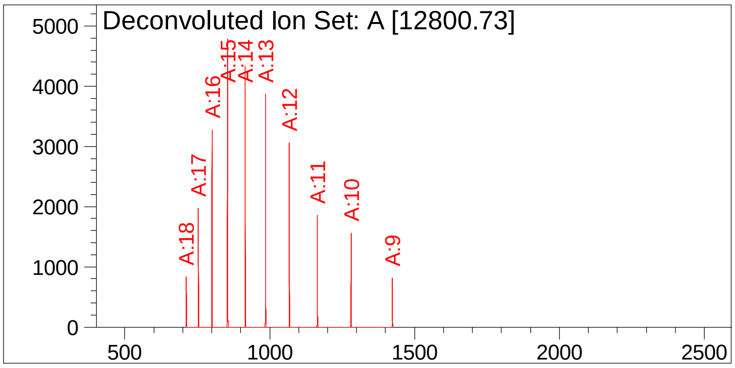

Component	Molecular Weight	Absolute Abundance	Relative Abundance
A	12800.73	26152	100.00

*** End of Report ***

Data File D:\EZXDATA\MARIARODRIGUEZ\12-24\121224-10-75OVER20_PEPTIDES-C3-30638.D
Sample Name: Brd4BD2_74_100_12dec24

===

Acq. Operator : Maria Rodriguez
Acq. Instrument : INSTRUMENT 1 Location : P1-B-06
Injection Date : 12/12/2024 6:36:45 PM Inj : 1
Inj Volume : 10.000 µl
Acq. Method : C:\CHEM32\1\METHODS\10-75OVER20_PEPTIDE
Last changed : 12/12/2024 6:35:48 PM by Maria Rodriguez
(modified after loading)
Analysis Method : C:\CHEM32\1\METHODS\10-75OVER20_PEPTIDES-5UL-C3.M
Last changed : 12/19/2024 5:16:45 PM by Liam Hales
(modified after loading)
Sample Info : Easy-Access Method: '10-75over20_C3'

Additional Info : Peak(s) manually integrated

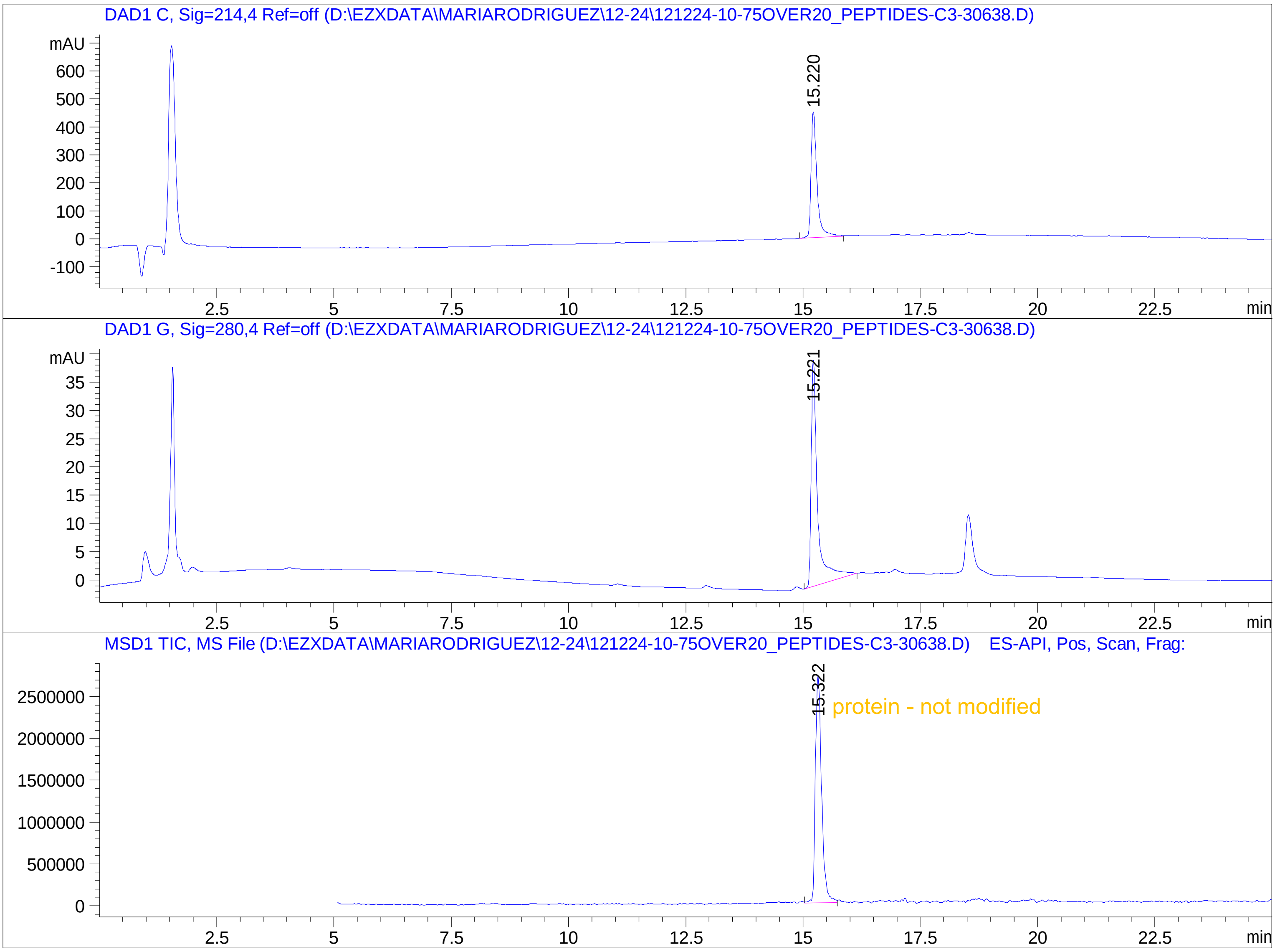

===
Area Percent Report
===

Sorted By : Signal
Multiplier : 1.0000
Dilution : 1.0000
Sample Amount: : 15.00000 [ng/ul] (not used in calc.)
Use Multiplier & Dilution Factor with ISTDs

Signal 1: DAD1 C, Sig=214,4 Ref=off

Peak #	RetTime [min]	Type	Width [min]	Area [mAU*s]	Height [mAU]	Area %
1	15.220	BV	0.1345	4022.61108	449.15967	100.0000

Totals : 4022.61108 449.15967

Signal 2: DAD1 G, Sig=280,4 Ref=off

Peak #	RetTime [min]	Type	Width [min]	Area [mAU*s]	Height [mAU]	Area %
1	15.221	BB	0.1385	378.32370	39.94930	100.0000

Totals : 378.32370 39.94930

Signal 3: MSD1 TIC, MS File

Peak #	RetTime [min]	Type	Width [min]	Area	Height	Area %
1	15.322	VV	0.1393	2.49082e7	2.72888e6	100.0000

Totals : 2.49082e7 2.72888e6

==

*** End of Report ***

Spectrum Deconvolution
Data File D:\EZXDATA\MARIARODRIGUEZ\12-24\121224-10-75OVER20_PEPTIDES-C3-30638.D
Sample Name: Brd4BD2_74_100_12dec24

```
Easy-Access Method: '10-75over20_C3'
=====================================================================
Acq. Operator   : Maria Rodriguez
Acq. Instrument : INSTRUMENT 1                     Location : P1-B-06
Injection Date  : 12/12/2024 6:36:45 PM                 Inj :   1
                                                 Inj Volume : 10.000 µl
Acq. Method     : C:\CHEM32\1\METHODS\10-75OVER20_PEPTIDE
Last changed    : 12/12/2024 6:35:48 PM by Maria Rodriguez
                  (modified after loading)
Analysis Method : C:\CHEM32\1\METHODS\10-75OVER20_PEPTIDES-5UL-C3.M
Last changed    : 12/19/2024 5:16:45 PM by Liam Hales
                  (modified after loading)
Sample Info     : Easy-Access Method: '10-75over20_C3'

Additional Info : Peak(s) manually integrated

=====================================================================
                      Deconvolution Parameters
=====================================================================

Adduct Ion(Positive): +H, 1.0079 Da
Adduct Ion(Negative):  , 0.0000 Da
Low MW:               500
DeconvStartChgMaximum Charge:       50
Minimum Peaks in Set: 3
Retain Residual:      No
Ion PWHH:             0.6 Da
MW Agreement:         0.05 %
Noise Cutoff:         1000 counts
Abundance Cutoff:     10 %
MW Assign:            Curve fit
MW Assign Cutoff:     40 %
Envelope Cutoff:      50 %
```

Deconvolution of Spectrum #   1 @ 14.995 - 16.075 min

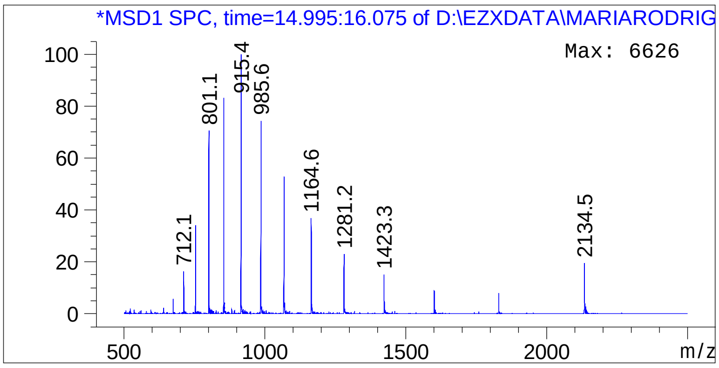

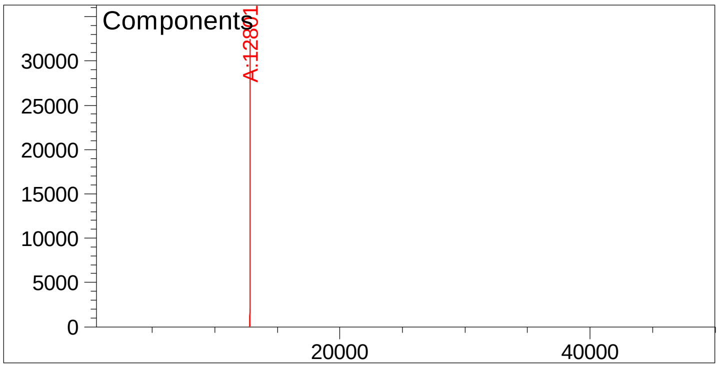

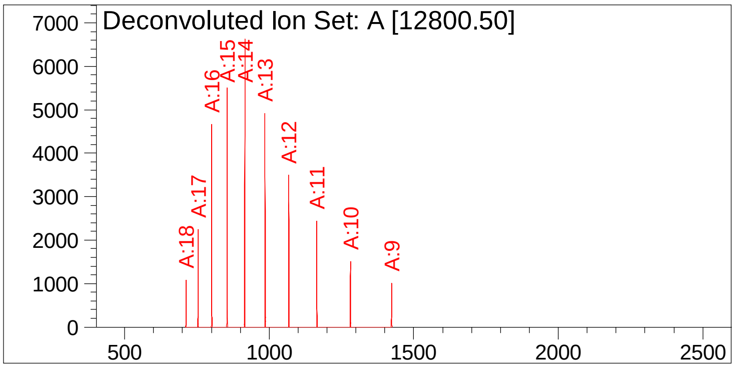

Component	Molecular Weight	Absolute Abundance	Relative Abundance
A	12800.50	32455	100.00

*** End of Report ***

Data File D:\EZXDATA\MARIARODRIGUEZ\12-24\121224-10-75OVER20_PEPTIDES-C3-30639.D
Sample Name: Brd4BD2_74_101_12dec24

===

Acq. Operator : Maria Rodriguez
Acq. Instrument : INSTRUMENT 1 Location : P1-B-07
Injection Date : 12/12/2024 7:06:43 PM Inj : 1
Inj Volume : 10.000 µl
Acq. Method : C:\CHEM32\1\METHODS\10-75OVER20_PEPTIDE
Last changed : 12/12/2024 7:05:45 PM by Maria Rodriguez
(modified after loading)
Analysis Method : C:\CHEM32\1\METHODS\10-75OVER20_PEPTIDES-5UL-C3.M
Last changed : 12/19/2024 5:18:00 PM by Liam Hales
(modified after loading)
Sample Info : Easy-Access Method: '10-75over20_C3'

Additional Info : Peak(s) manually integrated

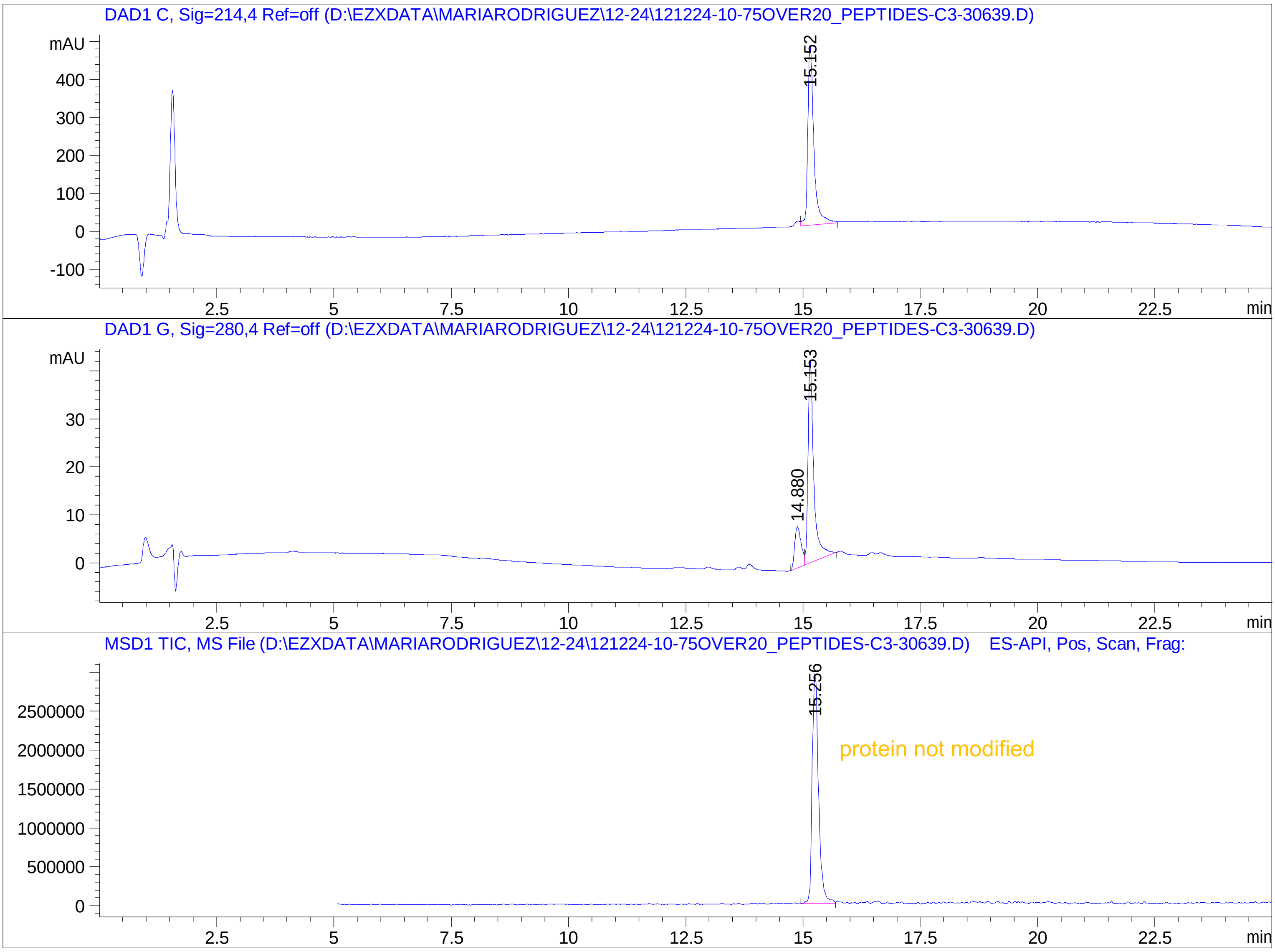

===

Area Percent Report

===

Sorted By : Signal
Multiplier : 1.0000
Dilution : 1.0000
Sample Amount: : 15.00000 [ng/ul] (not used in calc.)
Use Multiplier & Dilution Factor with ISTDs

Signal 1: DAD1 C, Sig=214,4 Ref=off

Peak #	RetTime [min]	Type	Width [min]	Area [mAU*s]	Height [mAU]	Area %
1	15.152	VV	0.1379	4293.90186	473.02881	100.0000
Totals :				4293.90186	473.02881	

Signal 2: DAD1 G, Sig=280,4 Ref=off

Peak #	RetTime [min]	Type	Width [min]	Area [mAU*s]	Height [mAU]	Area %
1	14.880	BV	0.1457	82.10262	8.57201	18.5177
2	15.153	VB	0.1256	361.27194	42.30935	81.4823
Totals :				443.37457	50.88135	

Signal 3: MSD1 TIC, MS File

Peak #	RetTime [min]	Type	Width [min]	Area	Height	Area %
1	15.256	BV	0.1421	2.75804e7	2.94422e6	100.0000
Totals :				2.75804e7	2.94422e6	

===

*** End of Report ***

Spectrum Deconvolution
Data File D:\EZXDATA\MARIARODRIGUEZ\12-24\121224-10-75OVER20_PEPTIDES-C3-30639.D
Sample Name: Brd4BD2_74_101_12dec24

```
    Easy-Access Method: '10-75over20_C3'
    =====================================================================
    Acq. Operator   : Maria Rodriguez
    Acq. Instrument : INSTRUMENT 1                    Location : P1-B-07
    Injection Date  : 12/12/2024 7:06:43 PM                Inj :   1
                                                   Inj Volume : 10.000 µl
    Acq. Method     : C:\CHEM32\1\METHODS\10-75OVER20_PEPTIDE
    Last changed    : 12/12/2024 7:05:45 PM by Maria Rodriguez
                      (modified after loading)
    Analysis Method : C:\CHEM32\1\METHODS\10-75OVER20_PEPTIDES-5UL-C3.M
    Last changed    : 12/19/2024 5:17:58 PM by Liam Hales
                      (modified after loading)
    Sample Info     : Easy-Access Method: '10-75over20_C3'

    Additional Info : Peak(s) manually integrated

    =====================================================================
                          Deconvolution Parameters
    =====================================================================

    Adduct Ion(Positive): +H, 1.0079 Da
    Adduct Ion(Negative):  , 0.0000 Da
    Low MW:               500
    DeconvStartChgMaximum Charge:       50
    Minimum Peaks in Set: 3
    Retain Residual:      No
    Ion PWHH:             0.6 Da
    MW Agreement:         0.05 %
    Noise Cutoff:         1000 counts
    Abundance Cutoff:     10 %
    MW Assign:            Curve fit
    MW Assign Cutoff:     40 %
    Envelope Cutoff:      50 %
```

Spectrum Deconvolution
Data File D:\EZXDATA\MARIARODRIGUEZ\12-24\121224-10-75OVER20_PEPTIDES-C3-30639.D
Sample Name: Brd4BD2_74_101_12dec24

Deconvolution of Spectrum #   1 @ 14.830 - 16.431 min

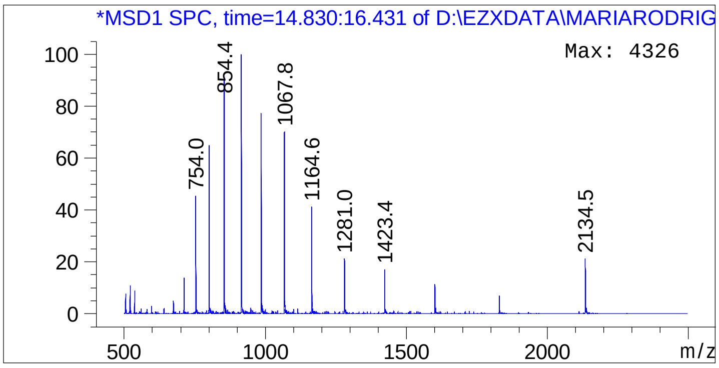

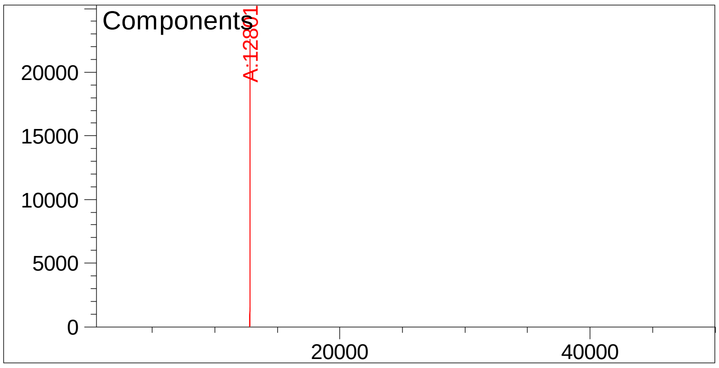

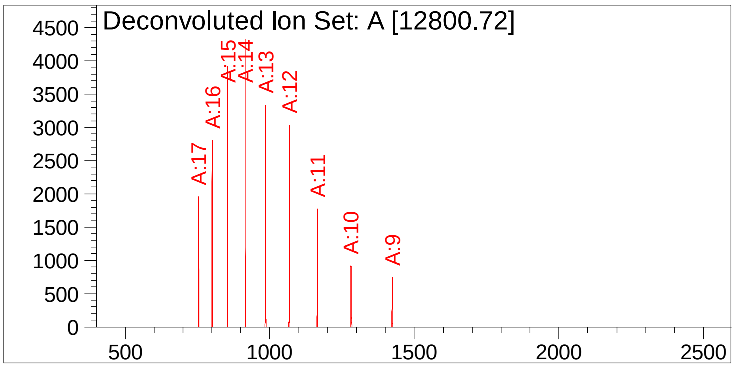

Component	Molecular Weight	Absolute Abundance	Relative Abundance
A	12800.72	22593	100.00

*** End of Report ***

Data File D:\EZXDATA\MARIARODRIGUEZ\12-24\121224-10-75OVER20_PEPTIDES-C3-30645.D
Sample Name: Brd4BD2_74_108_12dec24

===

Acq. Operator : Maria Rodriguez
Acq. Instrument : INSTRUMENT 1 Location : P1-C-04
Injection Date : 12/12/2024 10:06:24 PM Inj : 1
Inj Volume : 10.000 µl
Acq. Method : C:\CHEM32\1\METHODS\10-75OVER20_PEPTIDE
Last changed : 12/12/2024 10:05:29 PM by Maria Rodriguez
(modified after loading)
Analysis Method : C:\CHEM32\1\METHODS\10-75OVER20_PEPTIDES-5UL-C3.M
Last changed : 12/19/2024 5:27:44 PM by Liam Hales
(modified after loading)
Sample Info : Easy-Access Method: '10-75over20_C3'

Additional Info : Peak(s) manually integrated

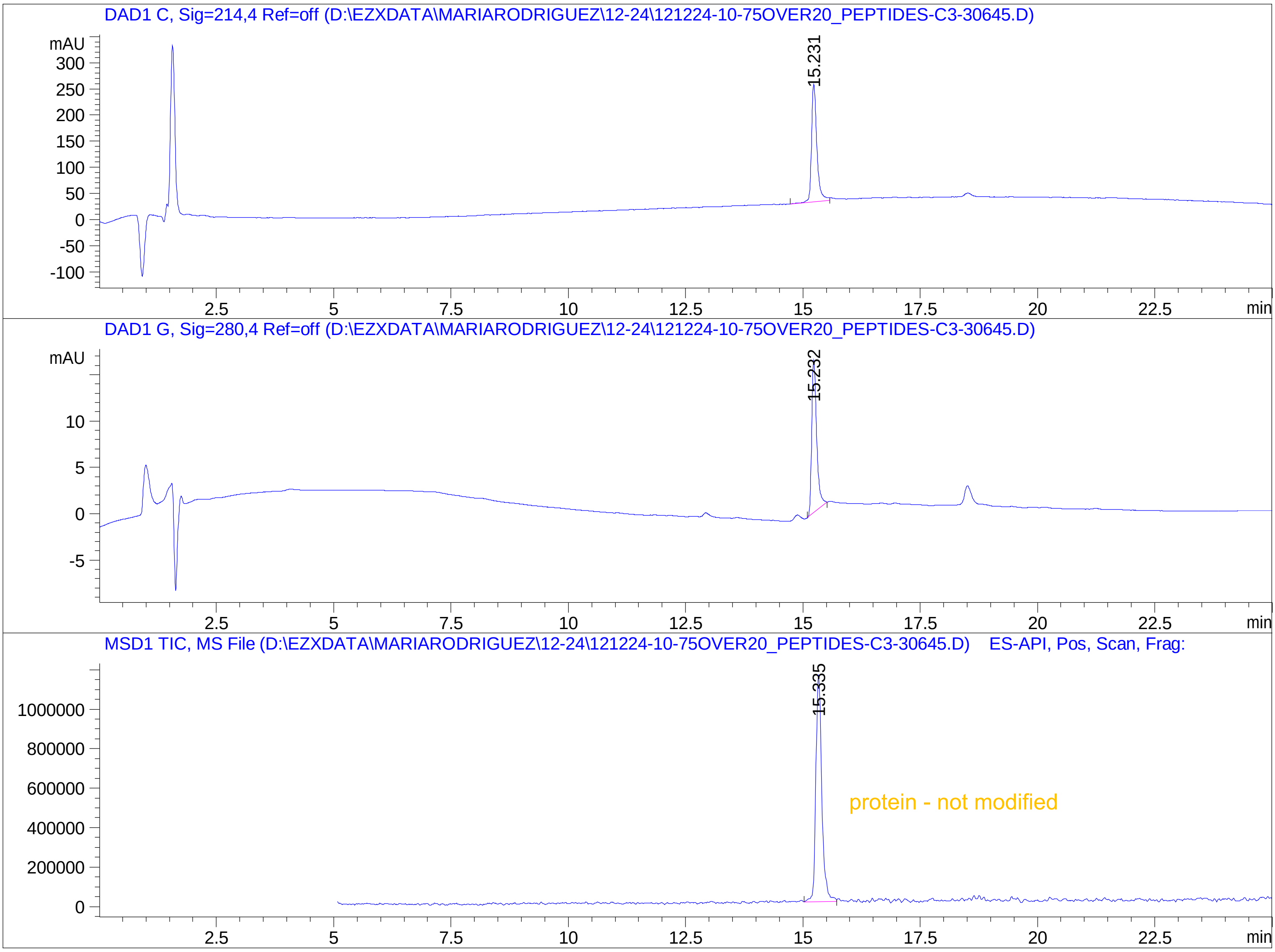

===
Area Percent Report
===

Sorted By : Signal
Multiplier : 1.0000
Dilution : 1.0000
Sample Amount: : 15.00000 [ng/ul] (not used in calc.)
Use Multiplier & Dilution Factor with ISTDs

Signal 1: DAD1 C, Sig=214,4 Ref=off

Peak #	RetTime [min]	Type	Width [min]	Area [mAU*s]	Height [mAU]	Area %
1	15.231	BV	0.1208	1790.31897	225.13939	100.0000
Totals :				1790.31897	225.13939	

Signal 2: DAD1 G, Sig=280,4 Ref=off

Peak #	RetTime [min]	Type	Width [min]	Area [mAU*s]	Height [mAU]	Area %
1	15.232	BB	0.1119	121.21861	16.45733	100.0000
Totals :				121.21861	16.45733	

Signal 3: MSD1 TIC, MS File

Peak #	RetTime [min]	Type	Width [min]	Area	Height	Area %
1	15.335	BV	0.1361	1.01853e7	1.15019e6	100.0000
Totals :				1.01853e7	1.15019e6	

===

*** End of Report ***

Spectrum Deconvolution
Data File D:\EZXDATA\MARIARODRIGUEZ\12-24\121224-10-75OVER20_PEPTIDES-C3-30645.D
Sample Name: Brd4BD2_74_108_12dec24

```
    Easy-Access Method: '10-75over20_C3'
    =====================================================================
    Acq. Operator   : Maria Rodriguez
    Acq. Instrument : INSTRUMENT 1                    Location : P1-C-04
    Injection Date  : 12/12/2024 10:06:24 PM               Inj :   1
                                                   Inj Volume : 10.000 µl
    Acq. Method     : C:\CHEM32\1\METHODS\10-75OVER20_PEPTIDE
    Last changed    : 12/12/2024 10:05:29 PM by Maria Rodriguez
                      (modified after loading)
    Analysis Method : C:\CHEM32\1\METHODS\10-75OVER20_PEPTIDES-5UL-C3.M
    Last changed    : 12/19/2024 5:27:43 PM by Liam Hales
                      (modified after loading)
    Sample Info     : Easy-Access Method: '10-75over20_C3'

    Additional Info : Peak(s) manually integrated

    =====================================================================
                          Deconvolution Parameters
    =====================================================================

    Adduct Ion(Positive): +H, 1.0079 Da
    Adduct Ion(Negative):  , 0.0000 Da
    Low MW:               5000
    DeconvStartChgMaximum Charge:       50
    Minimum Peaks in Set: 3
    Retain Residual:      No
    Ion PWHH:             0.6 Da
    MW Agreement:         0.05 %
    Noise Cutoff:         1000 counts
    Abundance Cutoff:     10 %
    MW Assign:            Curve fit
    MW Assign Cutoff:     40 %
    Envelope Cutoff:      50 %
```

Spectrum Deconvolution
Data File D:\EZXDATA\MARIARODRIGUEZ\12-24\121224-10-75OVER20_PEPTIDES-C3-30645.D
Sample Name: Brd4BD2_74_108_12dec24

Deconvolution of Spectrum # 1 @ 15.104 - 15.706 min

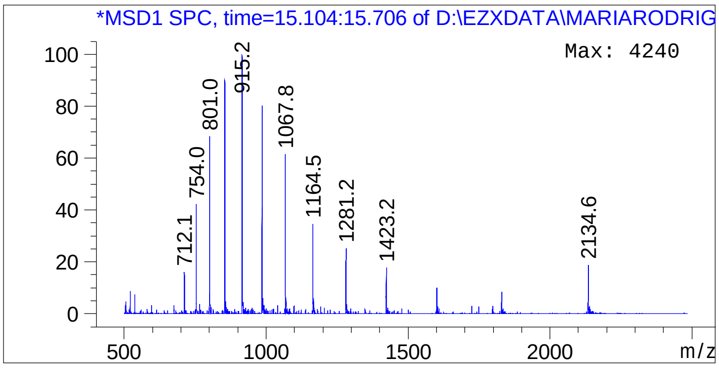

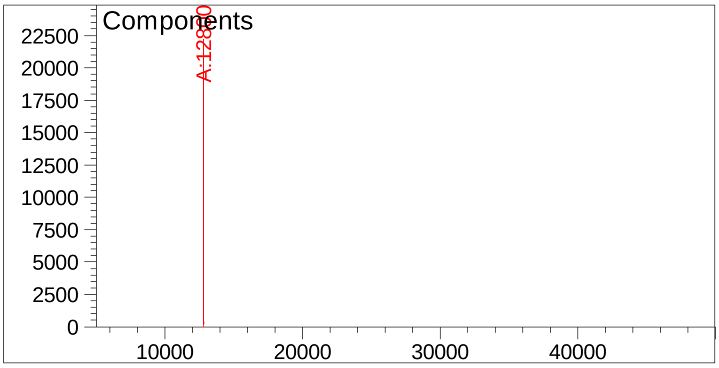

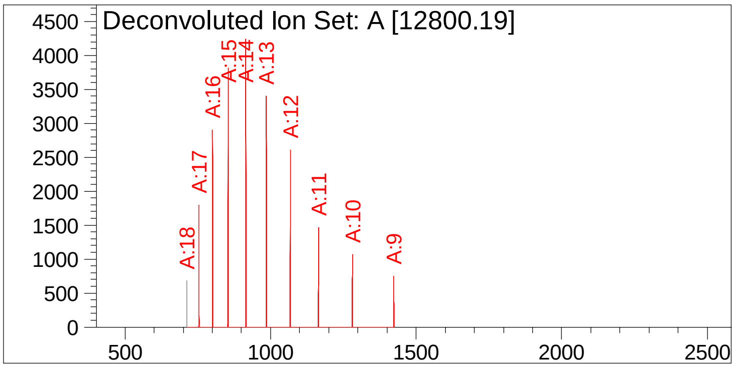

Component	Molecular Weight	Absolute Abundance	Relative Abundance
A	12800.19	22210	100.00

*** End of Report ***

Data File D:\EZXDATA\MARIARODRIGUEZ\12-24\121224-10-75OVER20_PEPTIDES-C3-30646.D
Sample Name: Brd4BD2_74_109_12dec24

===
Acq. Operator   : Maria Rodriguez
Acq. Instrument : INSTRUMENT 1   Location : P1-C-05
Injection Date : 12/12/2024 10:36:22 PM   Inj :   1
                                                Inj Volume : 10.000 µl
Acq. Method   : C:\CHEM32\1\METHODS\10-75OVER20_PEPTIDE
Last changed   : 12/12/2024 10:35:26 PM by Maria Rodriguez
                  (modified after loading)
Analysis Method : C:\CHEM32\1\METHODS\10-75OVER20_PEPTIDES-5UL-C3.M
Last changed   : 12/19/2024 5:27:44 PM by Liam Hales
                  (modified after loading)
Sample Info   : Easy-Access Method: '10-75over20_C3'

Additional Info : Peak(s) manually integrated

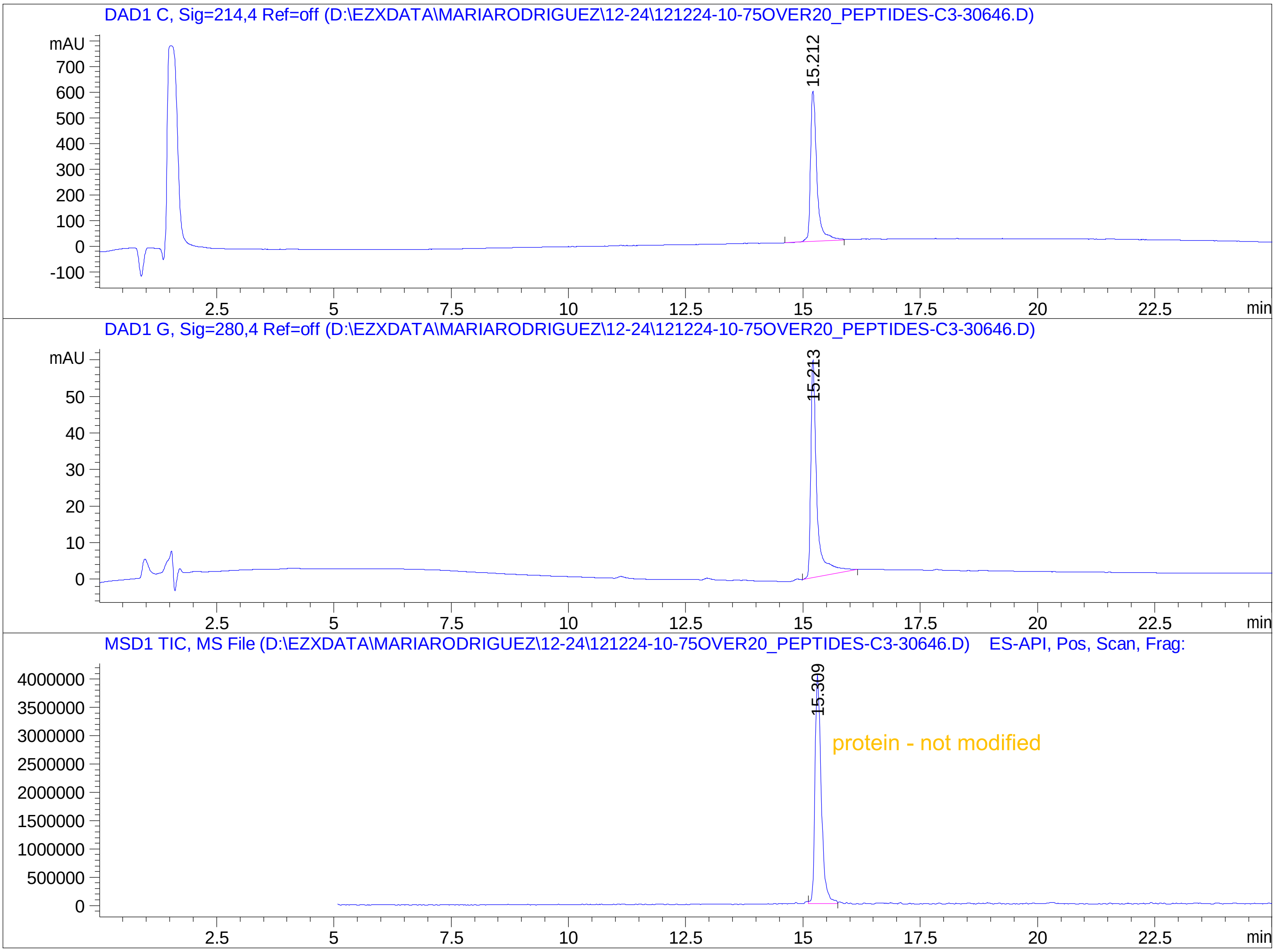

===
Area Percent Report
===

Sorted By   :   Signal
Multiplier   :   1.0000
Dilution   :   1.0000
Sample Amount: :   15.00000 [ng/ul]   (not used in calc.)
Use Multiplier & Dilution Factor with ISTDs

Signal 1: DAD1 C, Sig=214,4 Ref=off

Peak #	RetTime [min]	Type	Width [min]	Area [mAU*s]	Height [mAU]	Area %
1	15.212	BV	0.1451	5572.85498	585.11584	100.0000

Totals : 5572.85498 585.11584

Signal 2: DAD1 G, Sig=280,4 Ref=off

Peak #	RetTime [min]	Type	Width [min]	Area [mAU*s]	Height [mAU]	Area %
1	15.213	BB	0.1319	540.37598	59.55358	100.0000

Totals : 540.37598 59.55358

Signal 3: MSD1 TIC, MS File

Peak #	RetTime [min]	Type	Width [min]	Area	Height	Area %
1	15.309	VV	0.1405	3.73319e7	4.04583e6	100.0000

Totals : 3.73319e7 4.04583e6

===

*** End of Report ***

Spectrum Deconvolution
Data File D:\EZXDATA\MARIARODRIGUEZ\12-24\121224-10-75OVER20_PEPTIDES-C3-30646.D
Sample Name: Brd4BD2_74_109_12dec24

```
    Easy-Access Method: '10-75over20_C3'
    =====================================================================
    Acq. Operator   : Maria Rodriguez
    Acq. Instrument : INSTRUMENT 1                     Location : P1-C-05
    Injection Date  : 12/12/2024 10:36:22 PM                Inj :   1
                                                   Inj Volume : 10.000 µl
    Acq. Method     : C:\CHEM32\1\METHODS\10-75OVER20_PEPTIDE
    Last changed    : 12/12/2024 10:35:26 PM by Maria Rodriguez
                      (modified after loading)
    Analysis Method : C:\CHEM32\1\METHODS\10-75OVER20_PEPTIDES-5UL-C3.M
    Last changed    : 12/19/2024 5:27:44 PM by Liam Hales
                      (modified after loading)
    Sample Info     : Easy-Access Method: '10-75over20_C3'

    Additional Info : Peak(s) manually integrated

    =====================================================================
                          Deconvolution Parameters
    =====================================================================

    Adduct Ion(Positive): +H, 1.0079 Da
    Adduct Ion(Negative):  , 0.0000 Da
    Low MW:               5000
    DeconvStartChgMaximum Charge:       50
    Minimum Peaks in Set: 3
    Retain Residual:      No
    Ion PWHH:             0.6 Da
    MW Agreement:         0.05 %
    Noise Cutoff:         1000 counts
    Abundance Cutoff:     10 %
    MW Assign:            Curve fit
    MW Assign Cutoff:     40 %
    Envelope Cutoff:      50 %
```

Data File D:\EZXDATA\MARIARODRIGUEZ\12-24\121224-10-75OVER20_PEPTIDES-C3-30646.D
Sample Name: Brd4BD2_74_109_12dec24

Deconvolution of Spectrum # 1 @ 15.008 - 16.048 min

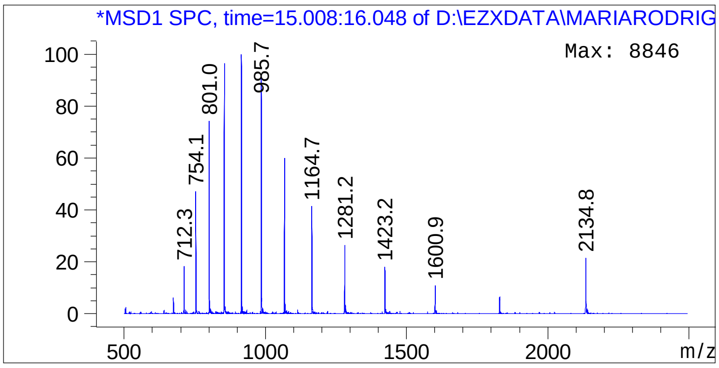

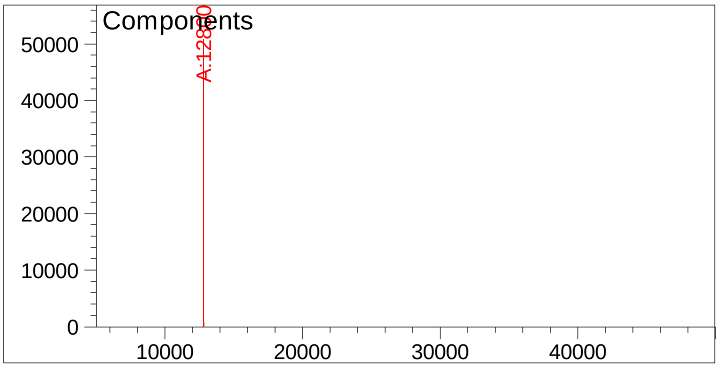

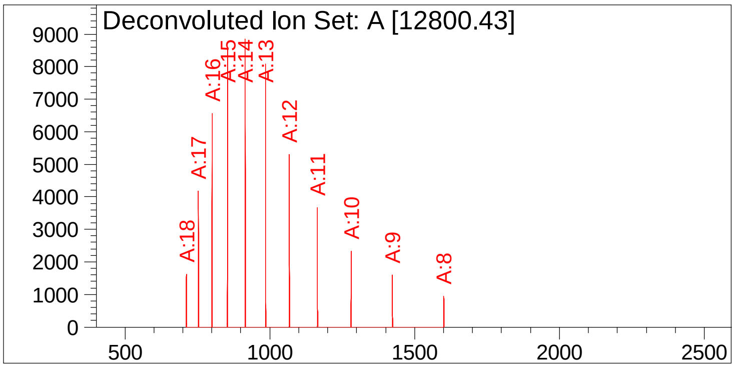

Component	Molecular Weight	Absolute Abundance	Relative Abundance
A	12800.43	50826	100.00

*** End of Report ***

Data File D:\EZXDATA\MARIARODRIGUEZ\12-24\121224-10-75OVER20_PEPTIDES-C3-30647.D
Sample Name: Brd4BD2_74_115_12dec24

===

Acq. Operator : Maria Rodriguez
Acq. Instrument : INSTRUMENT 1 Location : P1-C-06
Injection Date : 12/12/2024 11:06:19 PM Inj : 1
Inj Volume : 10.000 µl
Acq. Method : C:\CHEM32\1\METHODS\10-75OVER20_PEPTIDE
Last changed : 12/12/2024 11:05:24 PM by Maria Rodriguez
(modified after loading)
Analysis Method : C:\CHEM32\1\METHODS\10-75OVER20_PEPTIDES-5UL-C3.M
Last changed : 12/19/2024 5:29:24 PM by Liam Hales
(modified after loading)
Sample Info : Easy-Access Method: '10-75over20_C3'

Additional Info : Peak(s) manually integrated

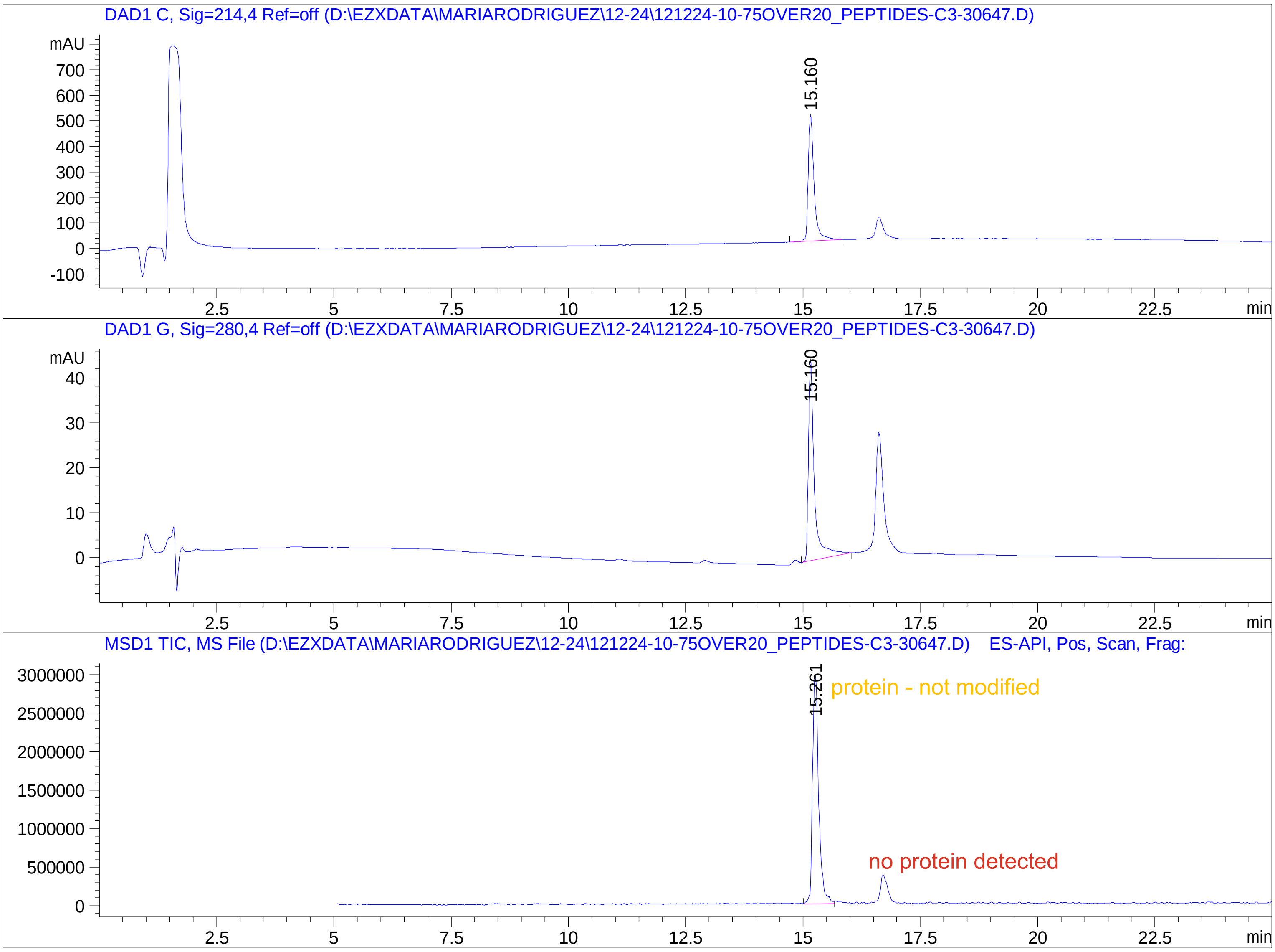

===

Area Percent Report

===

Sorted By : Signal
Multiplier : 1.0000
Dilution : 1.0000
Sample Amount: : 15.00000 [ng/ul] (not used in calc.)
Use Multiplier & Dilution Factor with ISTDs

Signal 1: DAD1 C, Sig=214,4 Ref=off

Peak #	RetTime [min]	Type	Width [min]	Area [mAU*s]	Height [mAU]	Area %
1	15.160	BB	0.1351	4356.68408	493.04822	100.0000

Totals : 4356.68408 493.04822

Signal 2: DAD1 G, Sig=280,4 Ref=off

Peak #	RetTime [min]	Type	Width [min]	Area [mAU*s]	Height [mAU]	Area %
1	15.160	BB	0.1296	397.47556	44.77067	100.0000

Totals : 397.47556 44.77067

Signal 3: MSD1 TIC, MS File

Peak #	RetTime [min]	Type	Width [min]	Area	Height	Area %
1	15.261	BV	0.1433	2.72606e7	2.99056e6	100.0000

Totals : 2.72606e7 2.99056e6

*** End of Report ***

Spectrum Deconvolution
Data File D:\EZXDATA\MARIARODRIGUEZ\12-24\121224-10-75OVER20_PEPTIDES-C3-30647.D
Sample Name: Brd4BD2_74_115_12dec24

```
Easy-Access Method: '10-75over20_C3'
=====================================================================
Acq. Operator   : Maria Rodriguez
Acq. Instrument : INSTRUMENT 1                     Location : P1-C-06
Injection Date  : 12/12/2024 11:06:19 PM                Inj :   1
                                                Inj Volume : 10.000 µl
Acq. Method     : C:\CHEM32\1\METHODS\10-75OVER20_PEPTIDE
Last changed    : 12/12/2024 11:05:24 PM by Maria Rodriguez
                  (modified after loading)
Analysis Method : C:\CHEM32\1\METHODS\10-75OVER20_PEPTIDES-5UL-C3.M
Last changed    : 12/19/2024 5:29:24 PM by Liam Hales
                  (modified after loading)
Sample Info     : Easy-Access Method: '10-75over20_C3'

Additional Info : Peak(s) manually integrated

=====================================================================
                      Deconvolution Parameters
=====================================================================

Adduct Ion(Positive): +H, 1.0079 Da
Adduct Ion(Negative):  , 0.0000 Da
Low MW:               5000
DeconvStartChgMaximum Charge:       50
Minimum Peaks in Set: 3
Retain Residual:      No
Ion PWHH:             0.6 Da
MW Agreement:         0.05 %
Noise Cutoff:         1000 counts
Abundance Cutoff:     10 %
MW Assign:            Curve fit
MW Assign Cutoff:     40 %
Envelope Cutoff:      50 %
```

Spectrum Deconvolution
Data File D:\EZXDATA\MARIARODRIGUEZ\12-24\121224-10-75OVER20_PEPTIDES-C3-30647.D
Sample Name: Brd4BD2_74_115_12dec24

Deconvolution of Spectrum # 1 @ 14.434 - 16.198 min

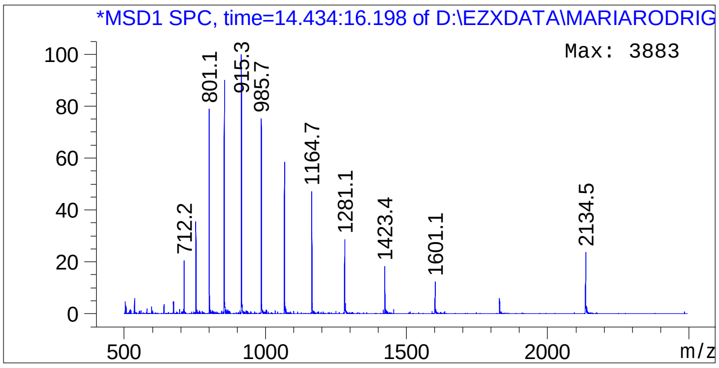

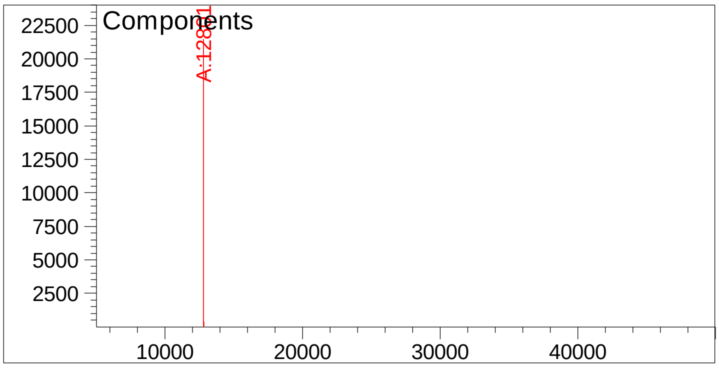

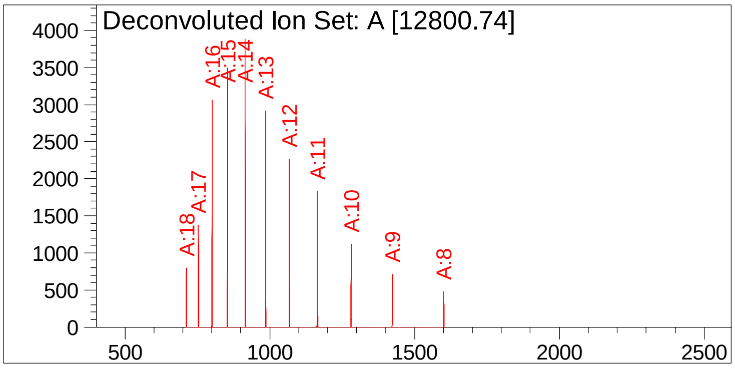

Component	Molecular Weight	Absolute Abundance	Relative Abundance
A	12800.74	21462	100.00

*** End of Report ***

Data File D:\EZXDATA\MARIARODRIGUEZ\12-24\121224-10-75OVER20_PEPTIDES-C3-30640.D
Sample Name: Brd4BD2_74_70_12dec24

===

Acq. Operator : Maria Rodriguez
Acq. Instrument : INSTRUMENT 1 Location : P1-B-08
Injection Date : 12/12/2024 7:36:41 PM Inj : 1
Inj Volume : 10.000 µl
Acq. Method : C:\CHEM32\1\METHODS\10-75OVER20_PEPTIDE
Last changed : 12/12/2024 7:35:44 PM by Maria Rodriguez
(modified after loading)
Analysis Method : C:\CHEM32\1\METHODS\10-75OVER20_PEPTIDES-5UL-C3.M
Last changed : 12/19/2024 5:19:48 PM by Liam Hales
(modified after loading)
Sample Info : Easy-Access Method: '10-75over20_C3'

Additional Info : Peak(s) manually integrated

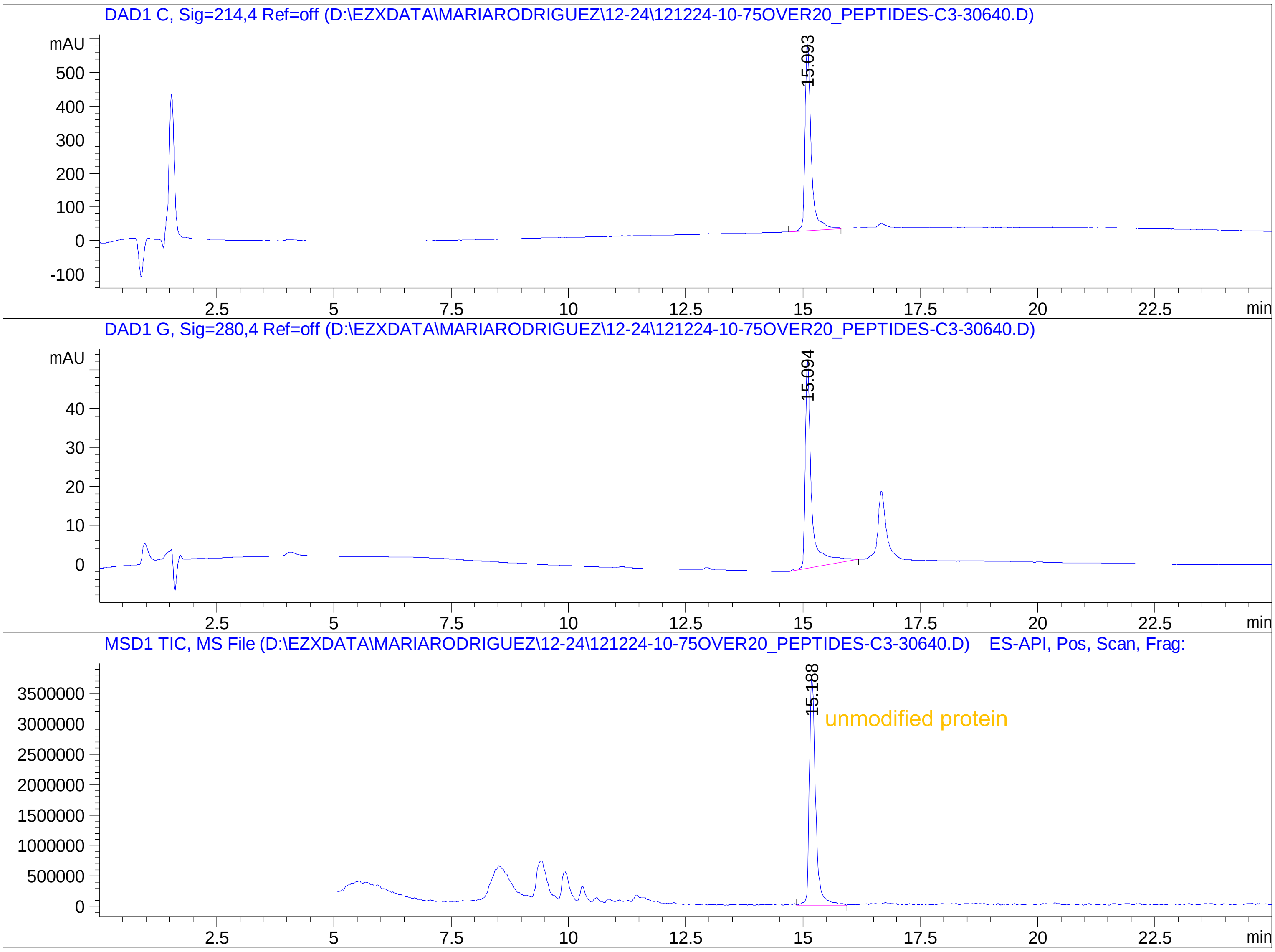

===
Area Percent Report
===

Sorted By : Signal
Multiplier : 1.0000
Dilution : 1.0000
Sample Amount: : 15.00000 [ng/ul] (not used in calc.)
Use Multiplier & Dilution Factor with ISTDs

Signal 1: DAD1 C, Sig=214,4 Ref=off

Peak #	RetTime [min]	Type	Width [min]	Area [mAU*s]	Height [mAU]	Area %
1	15.093	BV	0.1405	5126.12207	551.06586	100.0000
Totals :				5126.12207	551.06586	

Signal 2: DAD1 G, Sig=280,4 Ref=off

Peak #	RetTime [min]	Type	Width [min]	Area [mAU*s]	Height [mAU]	Area %
1	15.094	BB	0.1365	507.87088	53.64358	100.0000
Totals :				507.87088	53.64358	

Signal 3: MSD1 TIC, MS File

Peak #	RetTime [min]	Type	Width [min]	Area	Height	Area %
1	15.188	VB	0.1401	3.48362e7	3.79001e6	100.0000
Totals :				3.48362e7	3.79001e6	

*** End of Report ***

Spectrum Deconvolution
Data File D:\EZXDATA\MARIARODRIGUEZ\12-24\121224-10-75OVER20_PEPTIDES-C3-30640.D
Sample Name: Brd4BD2_74_70_12dec24

```
Easy-Access Method: '10-75over20_C3'
=====================================================================
Acq. Operator   : Maria Rodriguez
Acq. Instrument : INSTRUMENT 1                     Location : P1-B-08
Injection Date  : 12/12/2024 7:36:41 PM                 Inj :    1
                                                 Inj Volume : 10.000 µl
Acq. Method     : C:\CHEM32\1\METHODS\10-75OVER20_PEPTIDE
Last changed    : 12/12/2024 7:35:44 PM by Maria Rodriguez
                  (modified after loading)
Analysis Method : C:\CHEM32\1\METHODS\10-75OVER20_PEPTIDES-5UL-C3.M
Last changed    : 12/19/2024 5:19:08 PM by Liam Hales
                  (modified after loading)
Sample Info     : Easy-Access Method: '10-75over20_C3'

Additional Info : Peak(s) manually integrated

=====================================================================
                      Deconvolution Parameters
=====================================================================

Adduct Ion(Positive): +H, 1.0079 Da
Adduct Ion(Negative):  , 0.0000 Da
Low MW:               500
DeconvStartChgMaximum Charge:       50
Minimum Peaks in Set: 3
Retain Residual:      No
Ion PWHH:             0.6 Da
MW Agreement:         0.05 %
Noise Cutoff:         1000 counts
Abundance Cutoff:     10 %
MW Assign:            Curve fit
MW Assign Cutoff:     40 %
Envelope Cutoff:      50 %
```

Data File D:\EZXDATA\MARIARODRIGUEZ\12-24\121224-10-75OVER20_PEPTIDES-C3-30640.D
Sample Name: Brd4BD2_74_70_12dec24

Deconvolution of Spectrum #   1 @ 14.529 - 16.650 min

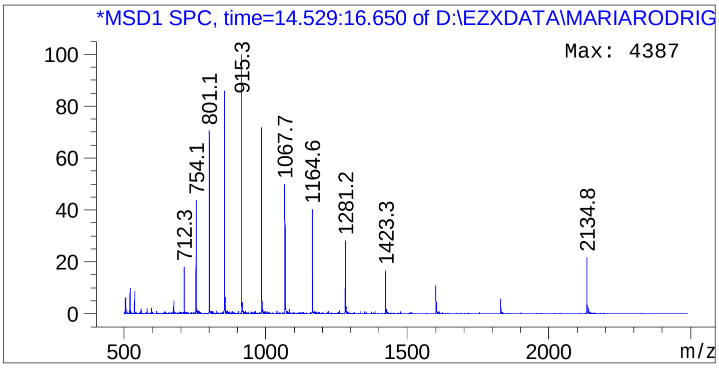

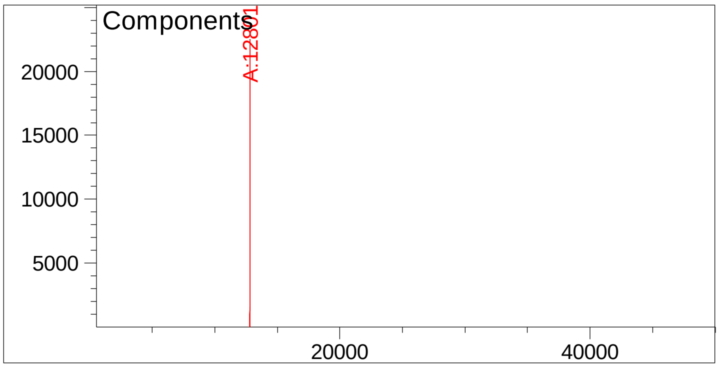

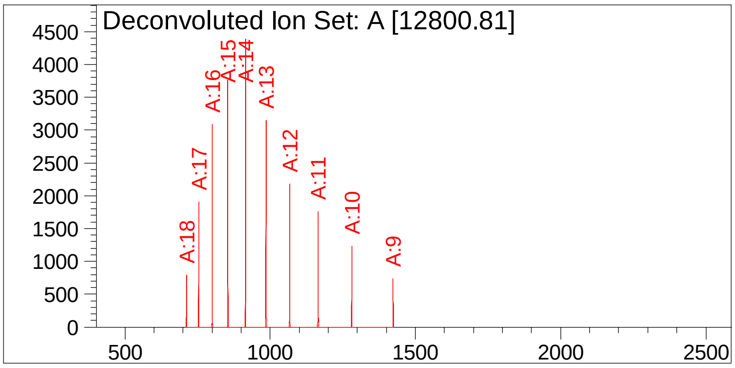

Component	Molecular Weight	Absolute Abundance	Relative Abundance
A	12800.81	22514	100.00

*** End of Report ***

Data File D:\EZXDATA\MARIARODRIGUEZ\12-24\121224-10-75OVER20_PEPTIDES-C3-30641.D
Sample Name: Brd4BD2_74_104_12dec24

===
Acq. Operator : Maria Rodriguez
Acq. Instrument : INSTRUMENT 1 Location : P1-B-09
Injection Date : 12/12/2024 8:06:37 PM Inj : 1
Inj Volume : 10.000 µl
Acq. Method : C:\CHEM32\1\METHODS\10-75OVER20_PEPTIDE
Last changed : 12/12/2024 8:05:41 PM by Maria Rodriguez
(modified after loading)
Analysis Method : C:\CHEM32\1\METHODS\10-75OVER20_PEPTIDES-5UL-C3.M
Last changed : 12/19/2024 5:21:15 PM by Liam Hales
(modified after loading)
Sample Info : Easy-Access Method: '10-75over20_C3'

Additional Info : Peak(s) manually integrated

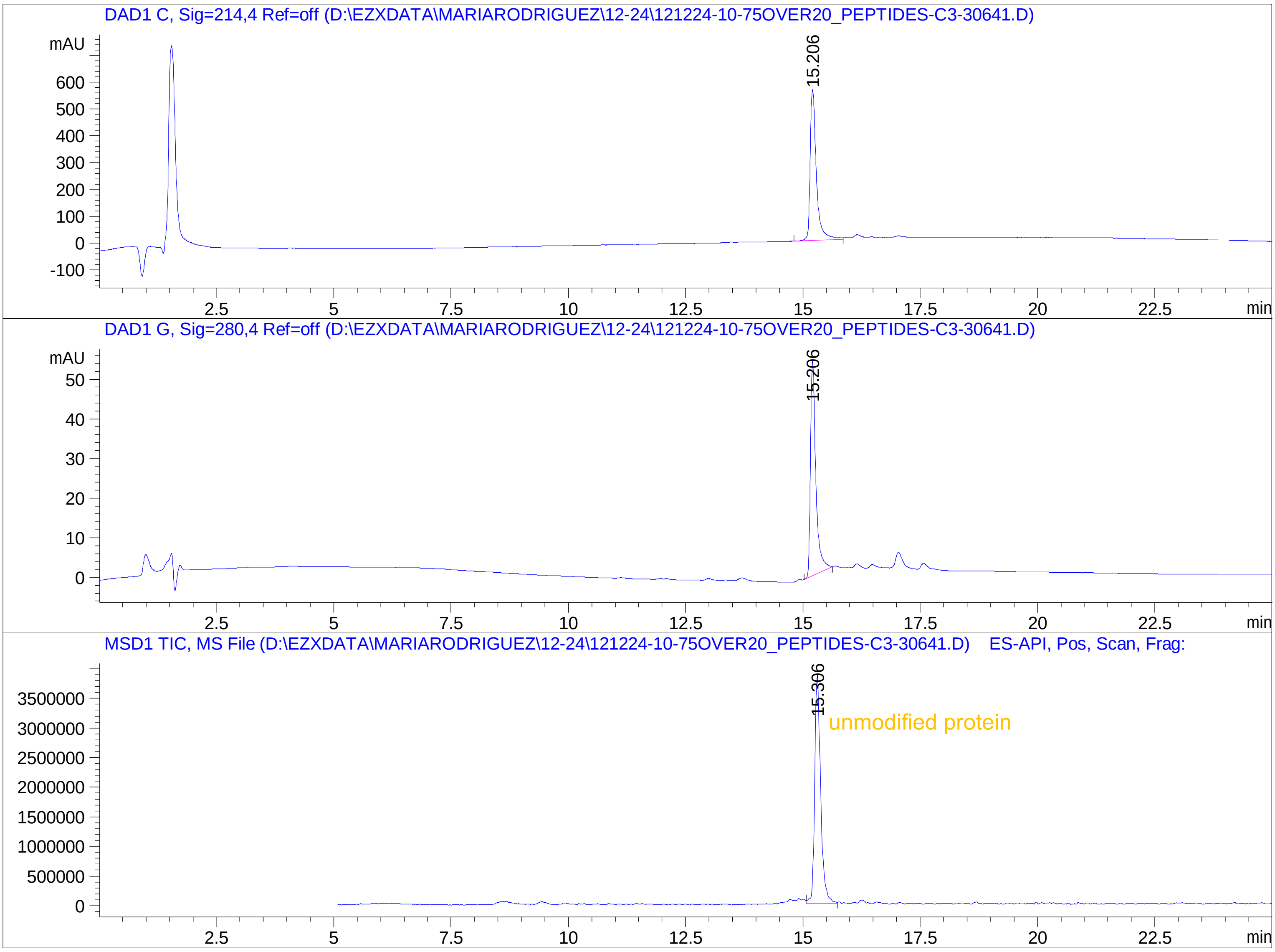

===
Area Percent Report
===

Sorted By : Signal
Multiplier : 1.0000
Dilution : 1.0000
Sample Amount: : 15.00000 [ng/ul] (not used in calc.)
Use Multiplier & Dilution Factor with ISTDs

Signal 1: DAD1 C, Sig=214,4 Ref=off

Peak #	RetTime [min]	Type	Width [min]	Area [mAU*s]	Height [mAU]	Area %
1	15.206	BV	0.1404	5228.37305	562.60925	100.0000
Totals :				5228.37305	562.60925	

Signal 2: DAD1 G, Sig=280,4 Ref=off

Peak #	RetTime [min]	Type	Width [min]	Area [mAU*s]	Height [mAU]	Area %
1	15.206	BB	0.1188	432.36984	54.35858	100.0000
Totals :				432.36984	54.35858	

Signal 3: MSD1 TIC, MS File

Peak #	RetTime [min]	Type	Width [min]	Area	Height	Area %
1	15.306	VV	0.1321	3.42826e7	3.87060e6	100.0000
Totals :				3.42826e7	3.87060e6	

==
*** End of Report ***

Spectrum Deconvolution
Data File D:\EZXDATA\MARIARODRIGUEZ\12-24\121224-10-75OVER20_PEPTIDES-C3-30641.D
Sample Name: Brd4BD2_74_104_12dec24

Easy-Access Method: '10-75over20_C3'
===
Acq. Operator : Maria Rodriguez
Acq. Instrument : INSTRUMENT 1 Location : P1-B-09
Injection Date : 12/12/2024 8:06:37 PM Inj : 1
Inj Volume : 10.000 µl
Acq. Method : C:\CHEM32\1\METHODS\10-75OVER20_PEPTIDE
Last changed : 12/12/2024 8:05:41 PM by Maria Rodriguez
(modified after loading)
Analysis Method : C:\CHEM32\1\METHODS\10-75OVER20_PEPTIDES-5UL-C3.M
Last changed : 12/19/2024 5:21:14 PM by Liam Hales
(modified after loading)
Sample Info : Easy-Access Method: '10-75over20_C3'

Additional Info : Peak(s) manually integrated

===
Deconvolution Parameters
===

Adduct Ion(Positive): +H, 1.0079 Da
Adduct Ion(Negative): , 0.0000 Da
Low MW: 5000
DeconvStartChgMaximum Charge: 50
Minimum Peaks in Set: 3
Retain Residual: No
Ion PWHH: 0.6 Da
MW Agreement: 0.05 %
Noise Cutoff: 1000 counts
Abundance Cutoff: 10 %
MW Assign: Curve fit
MW Assign Cutoff: 40 %
Envelope Cutoff: 50 %

Spectrum Deconvolution
Data File D:\EZXDATA\MARIARODRIGUEZ\12-24\121224-10-75OVER20_PEPTIDES-C3-30641.D
Sample Name: Brd4BD2_74_104_12dec24

Deconvolution of Spectrum # 1 @ 14.789 - 16.390 min

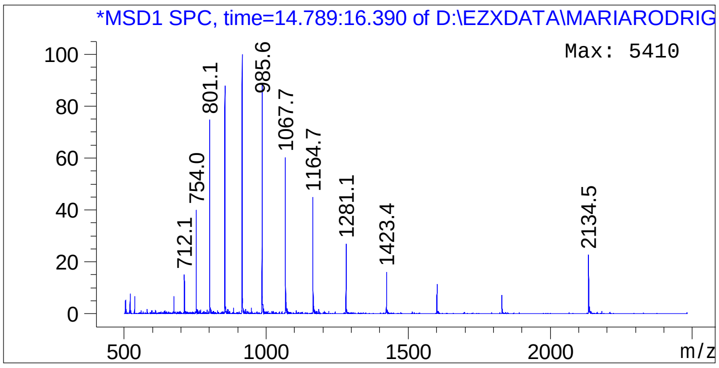

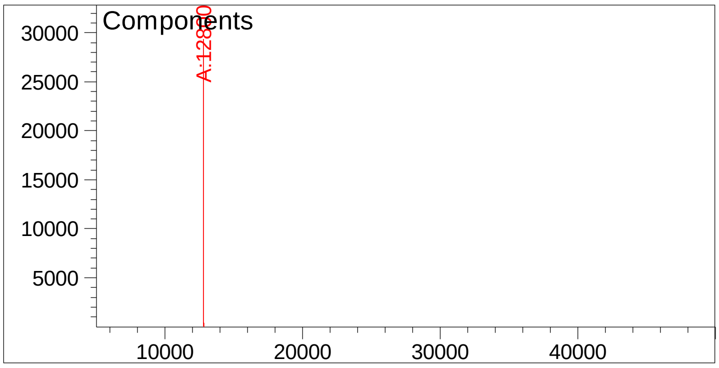

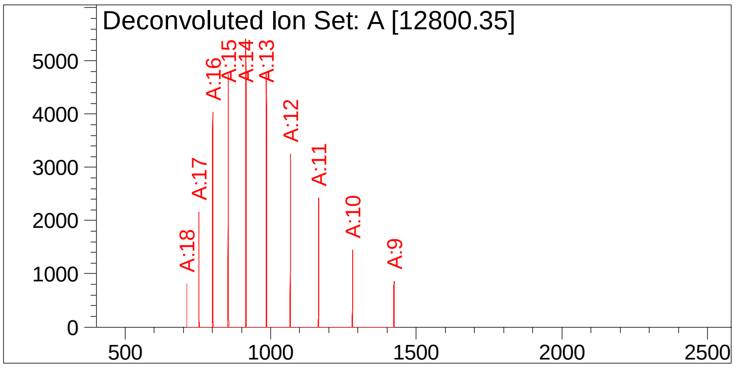

Component	Molecular Weight	Absolute Abundance	Relative Abundance
A	12800.35	29336	100.00

*** End of Report ***

Data File D:\EZXDATA\MARIARODRIGUEZ\12-24\121224-10-75OVER20_PEPTIDES-C3-30648.D
Sample Name: Brd4BD2_74_111_12dec24

===

Acq. Operator : Maria Rodriguez
Acq. Instrument : INSTRUMENT 1 Location : P1-C-07
Injection Date : 12/12/2024 11:36:19 PM Inj : 1
Inj Volume : 10.000 µl
Acq. Method : C:\CHEM32\1\METHODS\10-75OVER20_PEPTIDE
Last changed : 12/12/2024 11:35:21 PM by Maria Rodriguez
(modified after loading)
Analysis Method : C:\CHEM32\1\METHODS\10-75OVER20_PEPTIDES-5UL-C3.M
Last changed : 12/19/2024 5:31:01 PM by Liam Hales
(modified after loading)
Sample Info : Easy-Access Method: '10-75over20_C3'

Additional Info : Peak(s) manually integrated

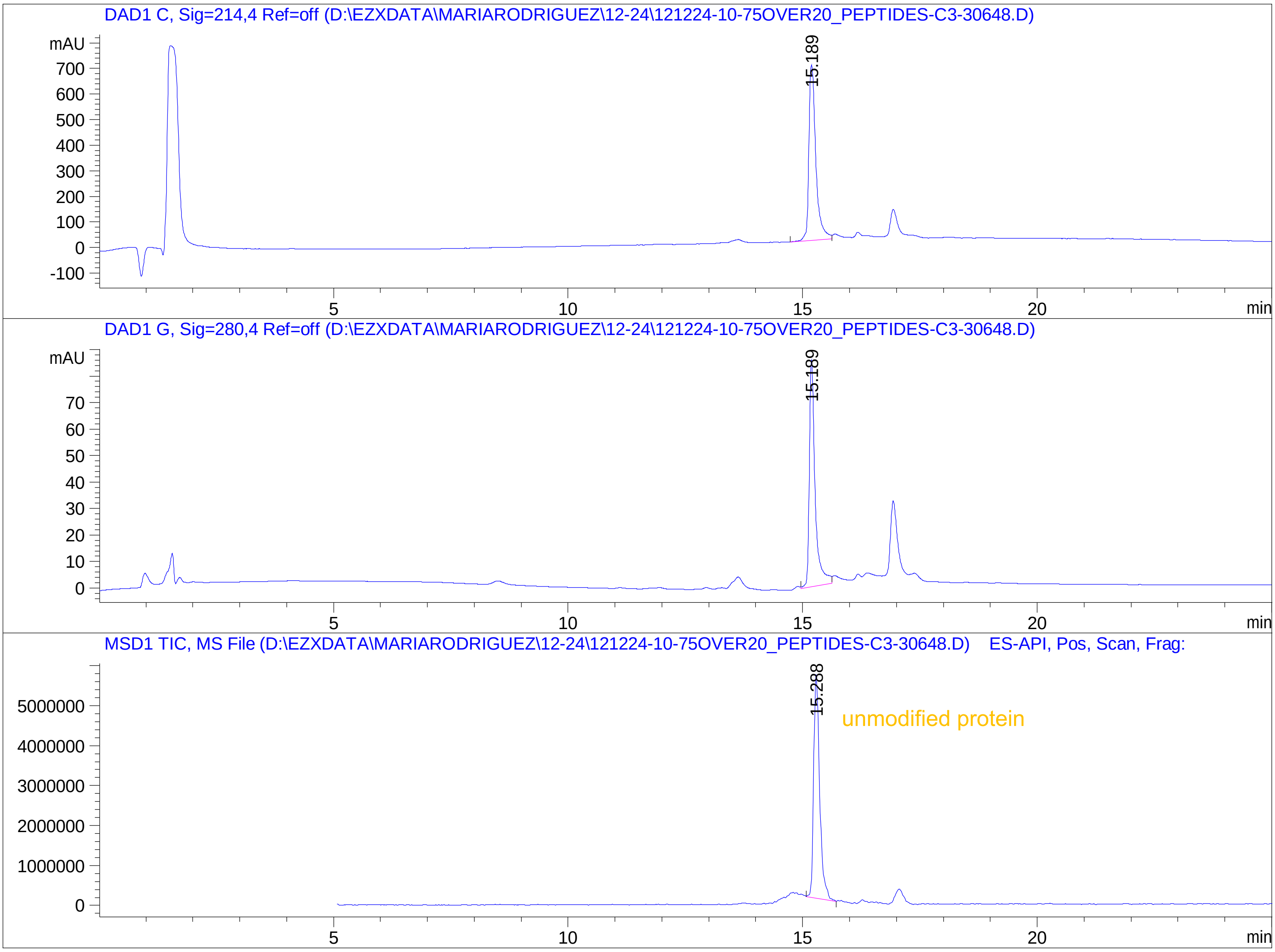

===

Area Percent Report

===

Sorted By : Signal
Multiplier : 1.0000
Dilution : 1.0000
Sample Amount: : 15.00000 [ng/ul] (not used in calc.)
Use Multiplier & Dilution Factor with ISTDs

Signal 1: DAD1 C, Sig=214,4 Ref=off

Peak #	RetTime [min]	Type	Width [min]	Area [mAU*s]	Height [mAU]	Area %
1	15.189	BV	0.1588	7091.92529	684.87842	100.0000
Totals :				7091.92529	684.87842	

Signal 2: DAD1 G, Sig=280,4 Ref=off

Peak #	RetTime [min]	Type	Width [min]	Area [mAU*s]	Height [mAU]	Area %
1	15.189	VV	0.1265	737.94794	85.65382	100.0000
Totals :				737.94794	85.65382	

Signal 3: MSD1 TIC, MS File

Peak #	RetTime [min]	Type	Width [min]	Area	Height	Area %
1	15.288	BB	0.1318	4.97074e7	5.62854e6	100.0000
Totals :				4.97074e7	5.62854e6	

===

*** End of Report ***

Spectrum Deconvolution
Data File D:\EZXDATA\MARIARODRIGUEZ\12-24\121224-10-75OVER20_PEPTIDES-C3-30648.D
Sample Name: Brd4BD2_74_111_12dec24

```
    Easy-Access Method: '10-75over20_C3'
    =====================================================================
    Acq. Operator   : Maria Rodriguez
    Acq. Instrument : INSTRUMENT 1                     Location : P1-C-07
    Injection Date  : 12/12/2024 11:36:19 PM                Inj :   1
                                                 Inj Volume : 10.000 µl
    Acq. Method     : C:\CHEM32\1\METHODS\10-75OVER20_PEPTIDE
    Last changed    : 12/12/2024 11:35:21 PM by Maria Rodriguez
                      (modified after loading)
    Analysis Method : C:\CHEM32\1\METHODS\10-75OVER20_PEPTIDES-5UL-C3.M
    Last changed    : 12/19/2024 5:31:00 PM by Liam Hales
                      (modified after loading)
    Sample Info     : Easy-Access Method: '10-75over20_C3'

    Additional Info : Peak(s) manually integrated

    =====================================================================
                          Deconvolution Parameters
    =====================================================================

    Adduct Ion(Positive): +H, 1.0079 Da
    Adduct Ion(Negative):  , 0.0000 Da
    Low MW:               5000
    DeconvStartChgMaximum Charge:       50
    Minimum Peaks in Set: 3
    Retain Residual:      No
    Ion PWHH:             0.6 Da
    MW Agreement:         0.05 %
    Noise Cutoff:         1000 counts
    Abundance Cutoff:     10 %
    MW Assign:            Curve fit
    MW Assign Cutoff:     40 %
    Envelope Cutoff:      50 %
```

Spectrum Deconvolution
Data File D:\EZXDATA\MARIARODRIGUEZ\12-24\121224-10-75OVER20_PEPTIDES-C3-30648.D
Sample Name: Brd4BD2_74_111_12dec24

Deconvolution of Spectrum # 1 @ 15.323 - 16.267 min

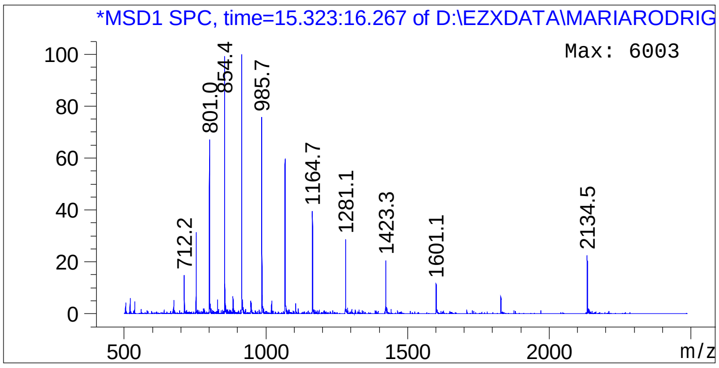

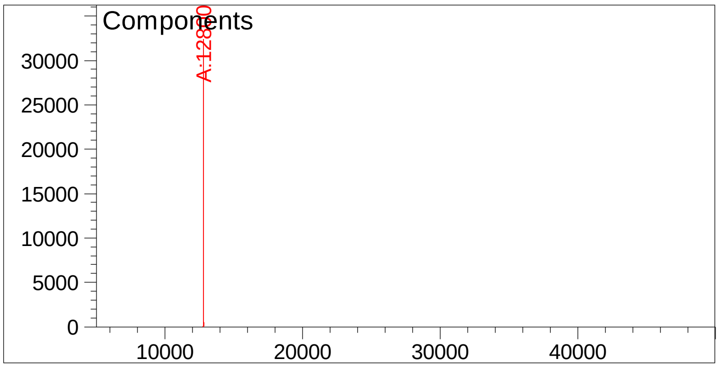

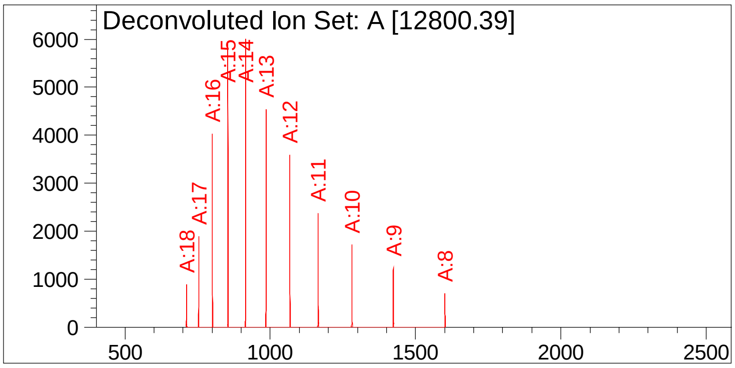

Component	Molecular Weight	Absolute Abundance	Relative Abundance
A	12800.39	32389	100.00

*** End of Report ***

===

```
Acq. Operator   : Maria Rodriguez
Acq. Instrument : INSTRUMENT 1                    Location : P1-C-08
Injection Date  : 12/13/2024 12:06:17 AM               Inj :   1
                                                Inj Volume : 10.000 µl
Acq. Method     : C:\CHEM32\1\METHODS\10-75OVER20_PEPTIDE
Last changed    : 12/13/2024 12:05:19 AM by Maria Rodriguez
                  (modified after loading)
Analysis Method : C:\CHEM32\1\METHODS\10-75OVER20_PEPTIDES-5UL-C3.M
Last changed    : 12/19/2024 5:32:51 PM by Liam Hales
                  (modified after loading)
Sample Info     : Easy-Access Method: '10-75over20_C3'

Additional Info : Peak(s) manually integrated
```

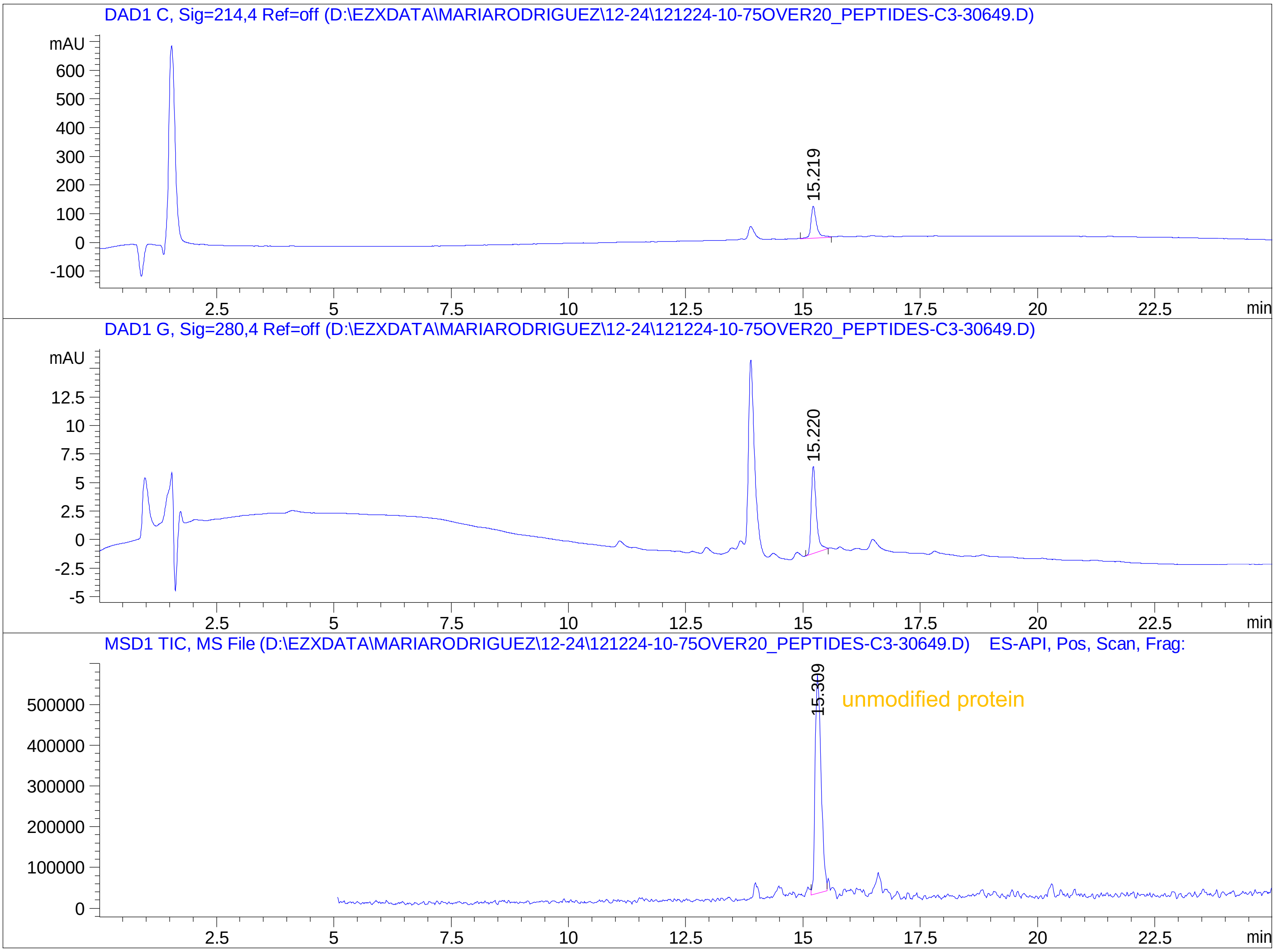

===

Area Percent Report

===

```
Sorted By             :      Signal
Multiplier            :      1.0000
Dilution              :      1.0000
Sample Amount:               :      15.00000  [ng/ul]   (not used in calc.)
Use Multiplier & Dilution Factor with ISTDs
```

Signal 1: DAD1 C, Sig=214,4 Ref=off

Peak #	RetTime [min]	Type	Width [min]	Area [mAU*s]	Height [mAU]	Area %
1	15.219	VV	0.1275	942.67596	110.50864	100.0000
Totals :				942.67596	110.50864	

Signal 2: DAD1 G, Sig=280,4 Ref=off

Peak #	RetTime [min]	Type	Width [min]	Area [mAU*s]	Height [mAU]	Area %
1	15.220	BB	0.1139	57.27344	7.60003	100.0000
Totals :				57.27344	7.60003	

Signal 3: MSD1 TIC, MS File

Peak #	RetTime [min]	Type	Width [min]	Area	Height	Area %
1	15.309	VV	0.1228	4.69824e6	5.36021e5	100.0000
Totals :				4.69824e6	5.36021e5	

*** End of Report ***

Spectrum Deconvolution
Data File D:\EZXDATA\MARIARODRIGUEZ\12-24\121224-10-75OVER20_PEPTIDES-C3-30649.D
Sample Name: Brd4BD2_74_118_12dec24

```
    Easy-Access Method: '10-75over20_C3'
    =====================================================================
    Acq. Operator   : Maria Rodriguez
    Acq. Instrument : INSTRUMENT 1                    Location : P1-C-08
    Injection Date  : 12/13/2024 12:06:17 AM                Inj :   1
                                                   Inj Volume : 10.000 µl
    Acq. Method     : C:\CHEM32\1\METHODS\10-75OVER20_PEPTIDE
    Last changed    : 12/13/2024 12:05:19 AM by Maria Rodriguez
                      (modified after loading)
    Analysis Method : C:\CHEM32\1\METHODS\10-75OVER20_PEPTIDES-5UL-C3.M
    Last changed    : 12/19/2024 5:32:51 PM by Liam Hales
                      (modified after loading)
    Sample Info     : Easy-Access Method: '10-75over20_C3'

    Additional Info : Peak(s) manually integrated

    =====================================================================
                          Deconvolution Parameters
    =====================================================================

    Adduct Ion(Positive): +H, 1.0079 Da
    Adduct Ion(Negative):  , 0.0000 Da
    Low MW:               5000
    DeconvStartChgMaximum Charge:       50
    Minimum Peaks in Set: 3
    Retain Residual:      No
    Ion PWHH:             0.6 Da
    MW Agreement:         0.05 %
    Noise Cutoff:         1000 counts
    Abundance Cutoff:     10 %
    MW Assign:            Curve fit
    MW Assign Cutoff:     40 %
    Envelope Cutoff:      50 %
```

Deconvolution of Spectrum # 1 @ 15.145 - 15.870 min

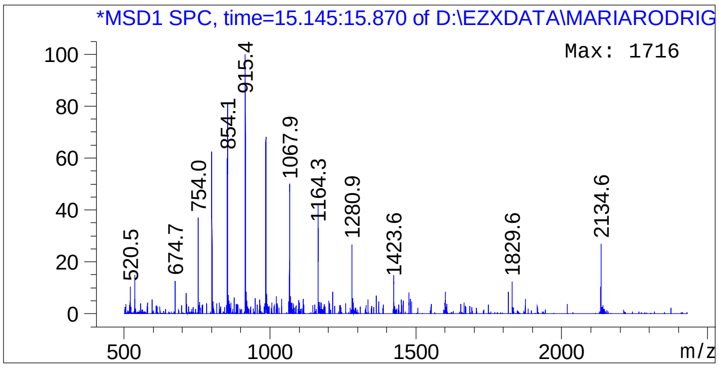

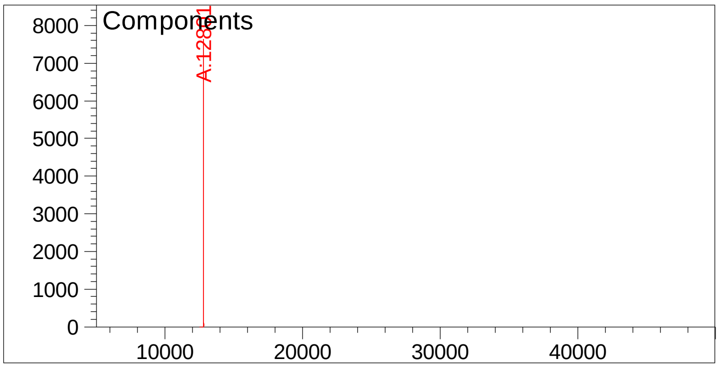

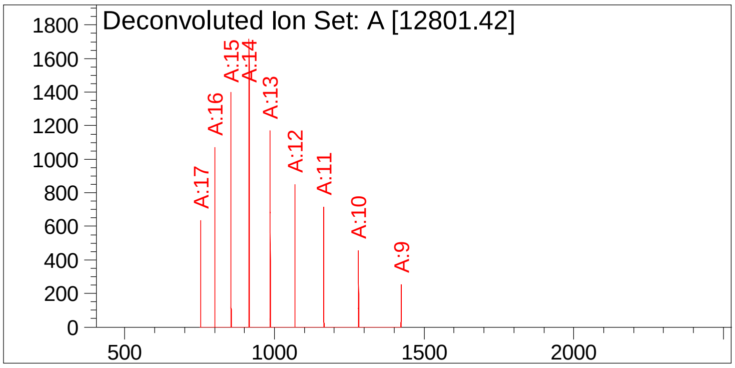

Component	Molecular Weight	Absolute Abundance	Relative Abundance
A	12801.42	7635	100.00

*** End of Report ***

Data File D:\EZXDATA\MARIARODRIGUEZ\12-24\121224-10-75OVER20_PEPTIDES-C3-30644.D
Sample Name: Brd4BD2_74_116_12dec24

===

Acq. Operator : Maria Rodriguez
Acq. Instrument : INSTRUMENT 1 Location : P1-C-03
Injection Date : 12/12/2024 9:36:27 PM Inj : 1
Inj Volume : 10.000 µl
Acq. Method : C:\CHEM32\1\METHODS\10-75OVER20_PEPTIDE
Last changed : 12/12/2024 9:35:31 PM by Maria Rodriguez
(modified after loading)
Analysis Method : C:\CHEM32\1\METHODS\10-75OVER20_PEPTIDES-5UL-C3.M
Last changed : 12/19/2024 5:25:47 PM by Liam Hales
(modified after loading)
Sample Info : Easy-Access Method: '10-75over20_C3'

Additional Info : Peak(s) manually integrated

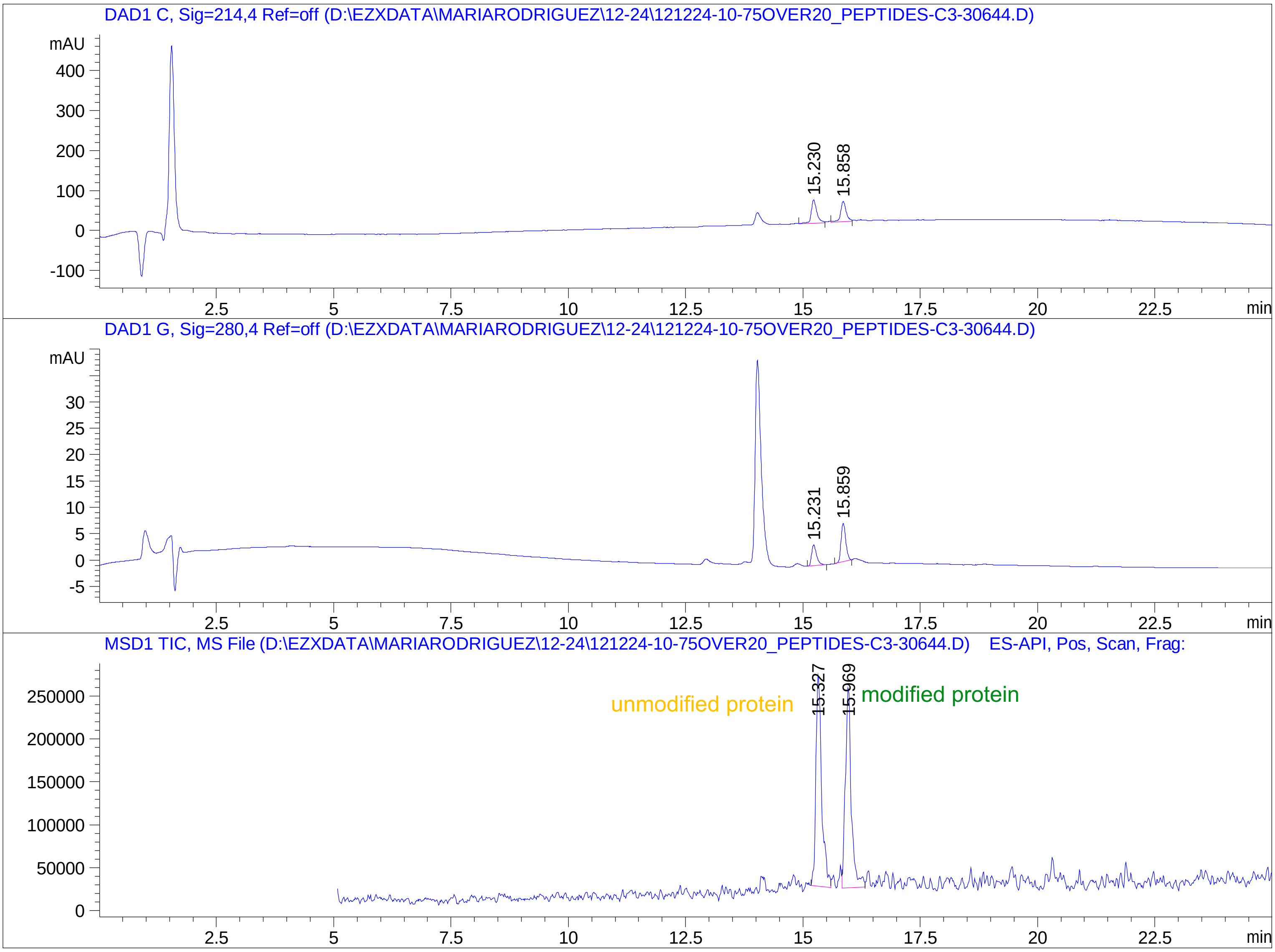

===

Area Percent Report

===

Sorted By : Signal
Multiplier : 1.0000
Dilution : 1.0000
Sample Amount: : 15.00000 [ng/ul] (not used in calc.)
Use Multiplier & Dilution Factor with ISTDs

Signal 1: DAD1 C, Sig=214,4 Ref=off

Peak #	RetTime [min]	Type	Width [min]	Area [mAU*s]	Height [mAU]	Area %
1	15.230	VV	0.1254	483.66458	57.92999	53.2867
2	15.858	VV	0.1231	423.99976	50.96665	46.7133
Totals :				907.66434	108.89664	

Signal 2: DAD1 G, Sig=280,4 Ref=off

Peak #	RetTime [min]	Type	Width [min]	Area [mAU*s]	Height [mAU]	Area %
1	15.231	BB	0.1145	29.20686	3.93748	36.5955
2	15.859	BB	0.1070	50.60306	7.28545	63.4045
Totals :				79.80992	11.22293	

Signal 3: MSD1 TIC, MS File

Peak #	RetTime [min]	Type	Width [min]	Area	Height	Area %
1	15.327	BV	0.1282	2.10570e6	2.47018e5	50.7346
2	15.969	VV	0.1271	2.04472e6	2.32885e5	49.2654
Totals :				4.15043e6	4.79903e5	

*** End of Report ***

Spectrum Deconvolution
Data File D:\EZXDATA\MARIARODRIGUEZ\12-24\121224-10-75OVER20_PEPTIDES-C3-30644.D
Sample Name: Brd4BD2_74_116_12dec24

Easy-Access Method: '10-75over20_C3'

===

```
Acq. Operator   : Maria Rodriguez
Acq. Instrument : INSTRUMENT 1                  Location : P1-C-03
Injection Date  : 12/12/2024 9:36:27 PM              Inj :   1
                                              Inj Volume : 10.000 µl
Acq. Method     : C:\CHEM32\1\METHODS\10-75OVER20_PEPTIDE
Last changed    : 12/12/2024 9:35:31 PM by Maria Rodriguez
                  (modified after loading)
Analysis Method : C:\CHEM32\1\METHODS\10-75OVER20_PEPTIDES-5UL-C3.M
Last changed    : 12/19/2024 5:25:47 PM by Liam Hales
                  (modified after loading)
Sample Info     : Easy-Access Method: '10-75over20_C3'

Additional Info : Peak(s) manually integrated
```

===

Deconvolution Parameters

===

```
Adduct Ion(Positive): +H, 1.0079 Da
Adduct Ion(Negative):  , 0.0000 Da
Low MW:               5000
DeconvStartChgMaximum Charge:       50
Minimum Peaks in Set: 3
Retain Residual:      No
Ion PWHH:             0.6 Da
MW Agreement:         0.05 %
Noise Cutoff:         1000 counts
Abundance Cutoff:     10 %
MW Assign:            Curve fit
MW Assign Cutoff:     40 %
Envelope Cutoff:      50 %
```

Spectrum Deconvolution
Data File D:\EZXDATA\MARIARODRIGUEZ\12-24\121224-10-75OVER20_PEPTIDES-C3-30644.D
Sample Name: Brd4BD2_74_116_12dec24

Deconvolution of Spectrum # 1 @ 15.227 - 15.665 min

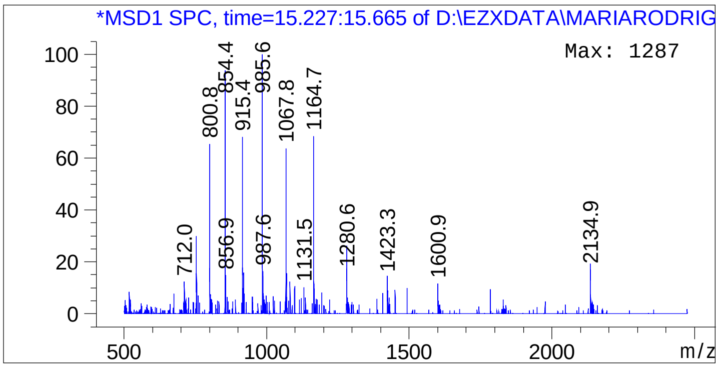

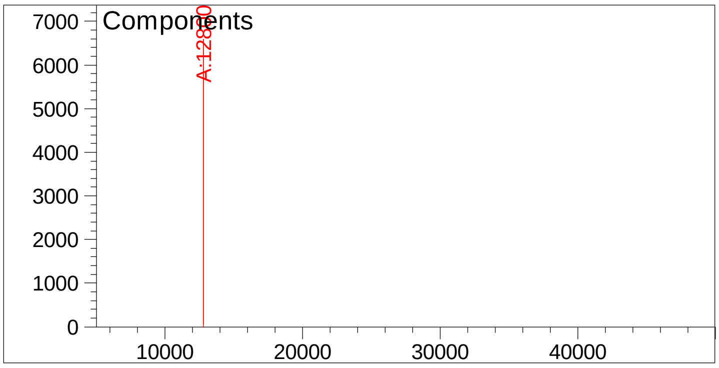

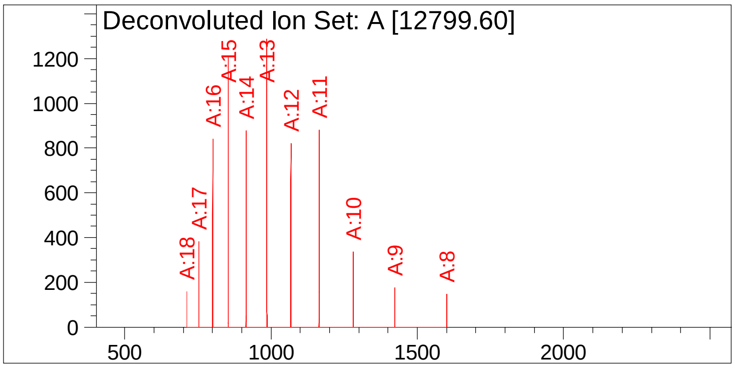

Component	Molecular Weight	Absolute Abundance	Relative Abundance
A	12799.60	6590	100.00

*** End of Report ***

Spectrum Deconvolution
Data File D:\EZXDATA\MARIARODRIGUEZ\12-24\121224-10-75OVER20_PEPTIDES-C3-30644.D
Sample Name: Brd4BD2_74_116_12dec24

```
Easy-Access Method: '10-75over20_C3'
=====================================================================
Acq. Operator   : Maria Rodriguez
Acq. Instrument : INSTRUMENT 1                    Location : P1-C-03
Injection Date  : 12/12/2024 9:36:27 PM                Inj :   1
                                              Inj Volume : 10.000 µl
Acq. Method     : C:\CHEM32\1\METHODS\10-75OVER20_PEPTIDE
Last changed    : 12/12/2024 9:35:31 PM by Maria Rodriguez
                  (modified after loading)
Analysis Method : C:\CHEM32\1\METHODS\10-75OVER20_PEPTIDES-5UL-C3.M
Last changed    : 12/19/2024 5:25:47 PM by Liam Hales
                  (modified after loading)
Sample Info     : Easy-Access Method: '10-75over20_C3'

Additional Info : Peak(s) manually integrated

=====================================================================
                      Deconvolution Parameters
=====================================================================

Adduct Ion(Positive): +H, 1.0079 Da
Adduct Ion(Negative):  , 0.0000 Da
Low MW:               5000
DeconvStartChgMaximum Charge:       50
Minimum Peaks in Set: 3
Retain Residual:      No
Ion PWHH:             0.6 Da
MW Agreement:         0.05 %
Noise Cutoff:         1000 counts
Abundance Cutoff:     10 %
MW Assign:            Curve fit
MW Assign Cutoff:     40 %
Envelope Cutoff:      50 %
```

Data File D:\EZXDATA\MARIARODRIGUEZ\12-24\121224-10-75OVER20_PEPTIDES-C3-30644.D
Sample Name: Brd4BD2_74_116_12dec24

Deconvolution of Spectrum # 1 @ 15.829 - 16.130 min

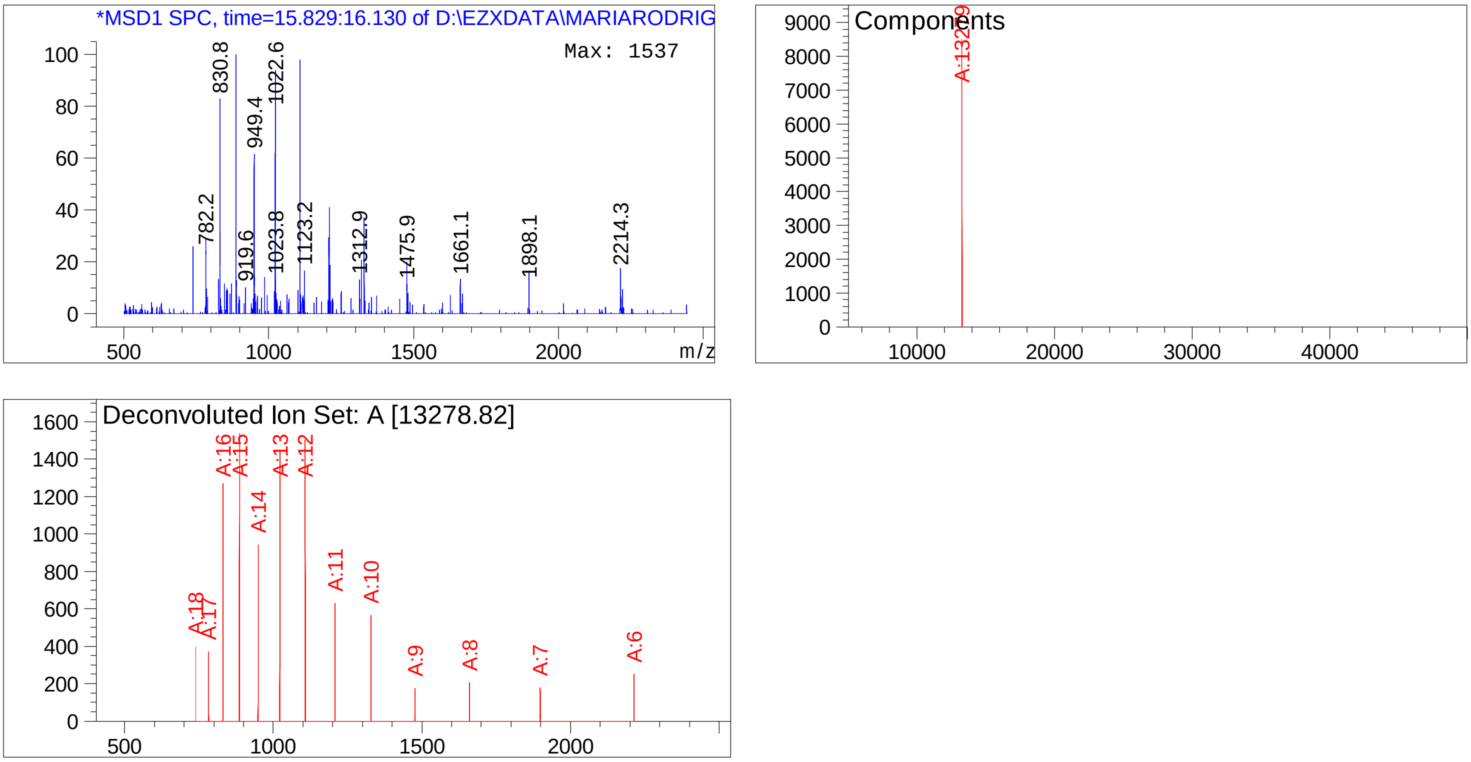

Component	Molecular Weight	Absolute Abundance	Relative Abundance
A	13278.82	8509	100.00

*** End of Report ***

Data File D:\EZXDATA\MARIAARODRIGUEZ\12-24\121224-10-75OVER20_PEPTIDES-C3-30643.D
Sample Name: Brd4BD2_74_121_12dec24

===

Acq. Operator : Maria Rodriguez
Acq. Instrument : INSTRUMENT 1 Location : P1-C-02
Injection Date : 12/12/2024 9:06:30 PM Inj : 1
Inj Volume : 10.000 µl
Acq. Method : C:\CHEM32\1\METHODS\10-75OVER20_PEPTIDE
Last changed : 12/12/2024 9:05:34 PM by Maria Rodriguez
(modified after loading)
Analysis Method : C:\CHEM32\1\METHODS\10-75OVER20_PEPTIDES-5UL-C3.M
Last changed : 12/19/2024 5:23:37 PM by Liam Hales
(modified after loading)
Sample Info : Easy-Access Method: '10-75over20_C3'

Additional Info : Peak(s) manually integrated

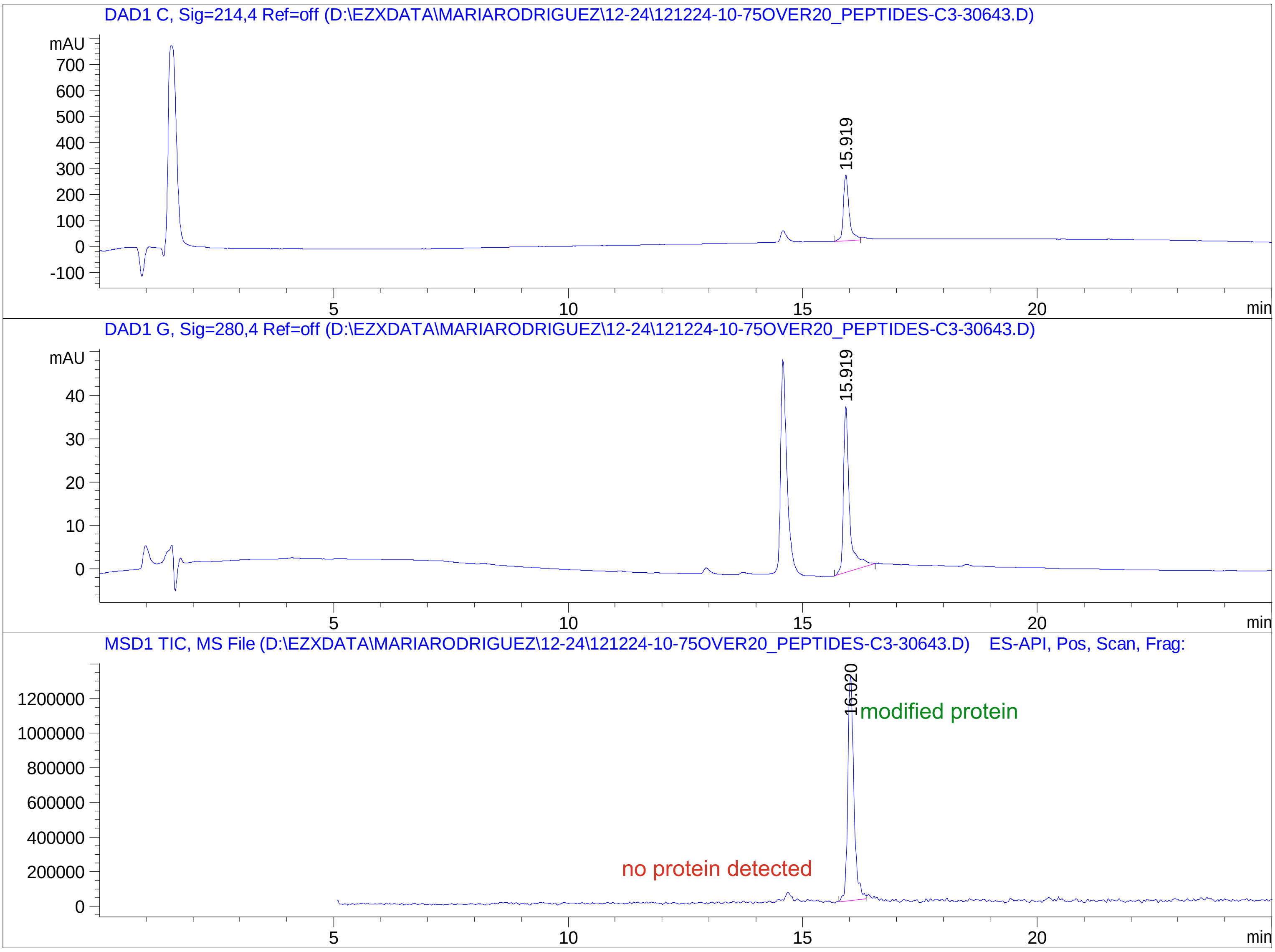

===

Area Percent Report

===

Sorted By : Signal
Multiplier : 1.0000
Dilution : 1.0000
Sample Amount: : 15.00000 [ng/ul] (not used in calc.)
Use Multiplier & Dilution Factor with ISTDs

Signal 1: DAD1 C, Sig=214,4 Ref=off

Peak #	RetTime [min]	Type	Width [min]	Area [mAU*s]	Height [mAU]	Area %
1	15.919	BV	0.1301	2163.42212	252.02696	100.0000
Totals :				2163.42212	252.02696	

Signal 2: DAD1 G, Sig=280,4 Ref=off

Peak #	RetTime [min]	Type	Width [min]	Area [mAU*s]	Height [mAU]	Area %
1	15.919	BB	0.1300	339.94354	38.13904	100.0000
Totals :				339.94354	38.13904	

Signal 3: MSD1 TIC, MS File

Peak #	RetTime [min]	Type	Width [min]	Area	Height	Area %
1	16.020	BV	0.1371	1.16696e7	1.30557e6	100.0000
Totals :				1.16696e7	1.30557e6	

===

*** End of Report ***

Spectrum Deconvolution
Data File D:\EZXDATA\MARIARODRIGUEZ\12-24\121224-10-75OVER20_PEPTIDES-C3-30643.D
Sample Name: Brd4BD2_74_121_12dec24

```
    Easy-Access Method: '10-75over20_C3'
    =====================================================================
    Acq. Operator   : Maria Rodriguez
    Acq. Instrument : INSTRUMENT 1                     Location : P1-C-02
    Injection Date  : 12/12/2024 9:06:30 PM                 Inj :   1
                                                     Inj Volume : 10.000 µl
    Acq. Method     : C:\CHEM32\1\METHODS\10-75OVER20_PEPTIDE
    Last changed    : 12/12/2024 9:05:34 PM by Maria Rodriguez
                      (modified after loading)
    Analysis Method : C:\CHEM32\1\METHODS\10-75OVER20_PEPTIDES-5UL-C3.M
    Last changed    : 12/19/2024 5:23:19 PM by Liam Hales
                      (modified after loading)
    Sample Info     : Easy-Access Method: '10-75over20_C3'

    Additional Info : Peak(s) manually integrated

    =====================================================================
                          Deconvolution Parameters
    =====================================================================

    Adduct Ion(Positive): +H, 1.0079 Da
    Adduct Ion(Negative):  , 0.0000 Da
    Low MW:               5000
    DeconvStartChgMaximum Charge:       50
    Minimum Peaks in Set: 3
    Retain Residual:      No
    Ion PWHH:             0.6 Da
    MW Agreement:         0.05 %
    Noise Cutoff:         1000 counts
    Abundance Cutoff:     10 %
    MW Assign:            Curve fit
    MW Assign Cutoff:     40 %
    Envelope Cutoff:      50 %
```

Spectrum Deconvolution
Data File D:\EZXDATA\MARIARODRIGUEZ\12-24\121224-10-75OVER20_PEPTIDES-C3-30643.D
Sample Name: Brd4BD2_74_121_12dec24

Deconvolution of Spectrum #   1 @ 15.788 - 16.445 min

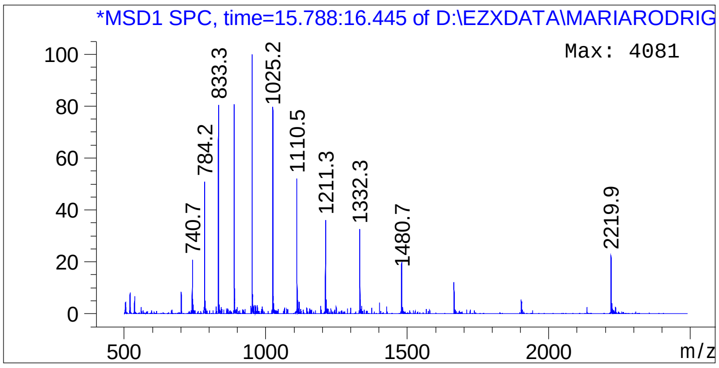

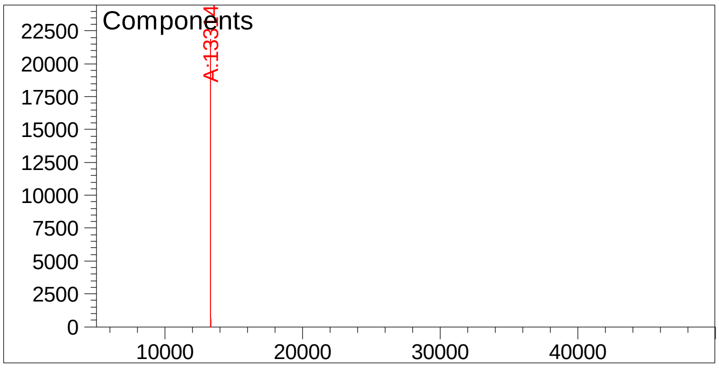

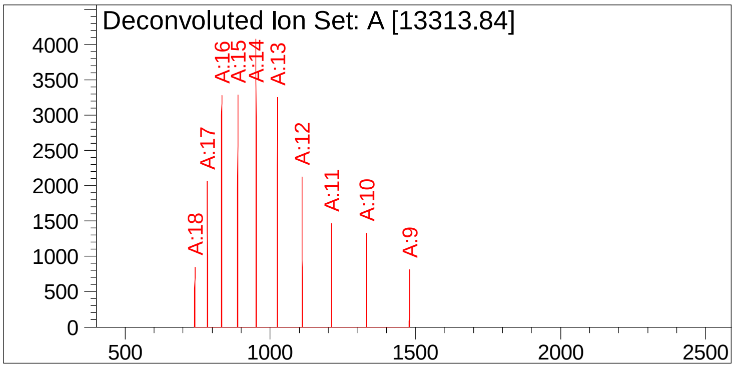

Component	Molecular Weight	Absolute Abundance	Relative Abundance
A	13313.84	21868	100.00

*** End of Report ***

Data File D:\EZXDATA\MARIARODRIGUEZ\12-24\121224-10-75OVER20_PEPTIDES-C3-30650.D
Sample Name: Brd4BD2_74_117_12dec24

===
Acq. Operator   : Maria Rodriguez
Acq. Instrument : INSTRUMENT 1   Location : P1-C-09
Injection Date : 12/13/2024 12:36:15 AM   Inj :   1
                                               Inj Volume : 10.000 µl
Acq. Method   : C:\CHEM32\1\METHODS\10-75OVER20_PEPTIDE
Last changed   : 12/13/2024 12:35:17 AM by Maria Rodriguez
                  (modified after loading)
Analysis Method : C:\CHEM32\1\METHODS\10-75OVER20_PEPTIDES-5UL-C3.M
Last changed   : 12/19/2024 5:35:55 PM by Liam Hales
                  (modified after loading)
Sample Info   : Easy-Access Method: '10-75over20_C3'

Additional Info : Peak(s) manually integrated

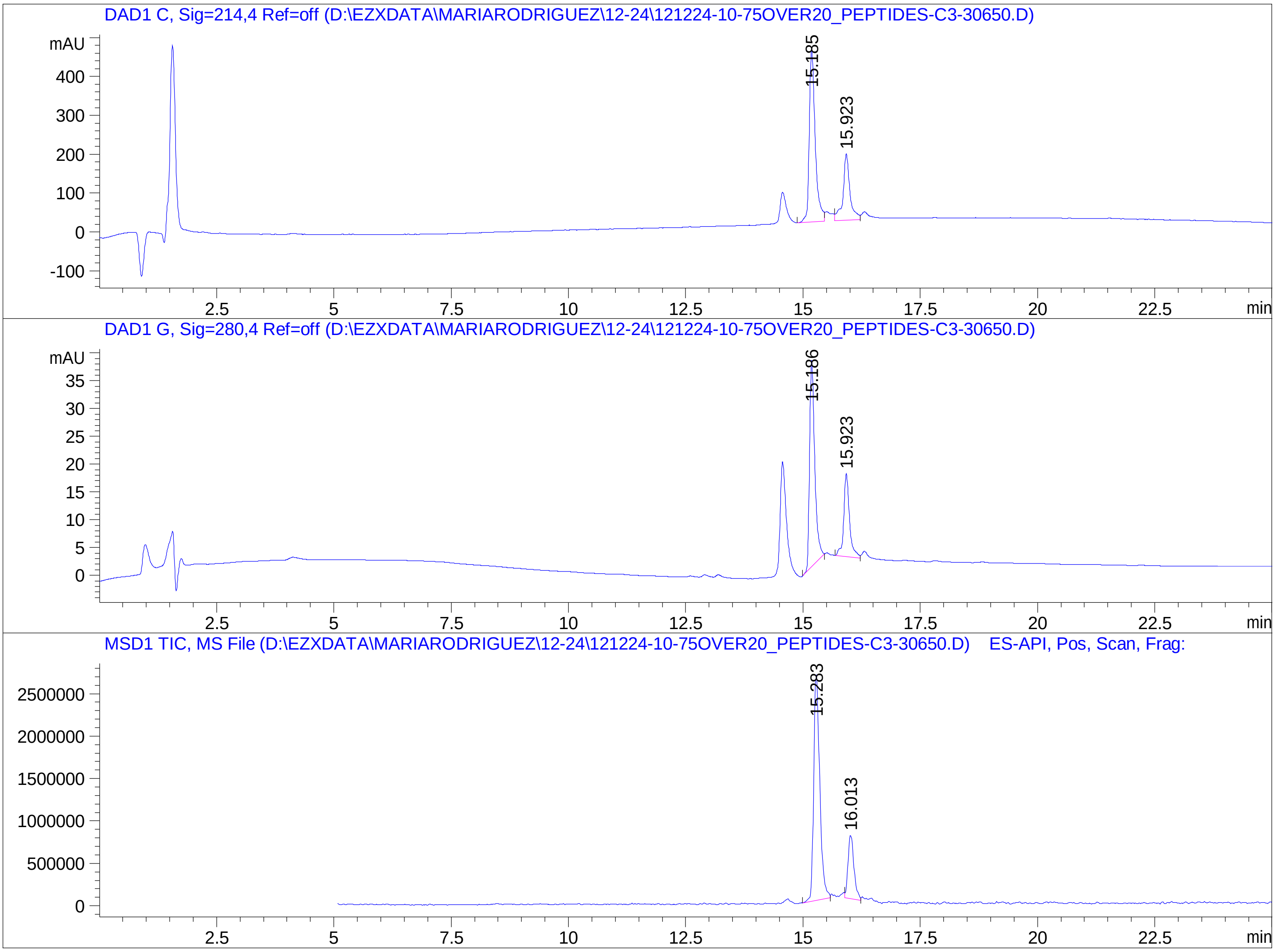

===
Area Percent Report
===

Sorted By   :   Signal
Multiplier   :   1.0000
Dilution   :   1.0000
Sample Amount: :   15.00000   [ng/ul]   (not used in calc.)
Use Multiplier & Dilution Factor with ISTDs

Signal 1: DAD1 C, Sig=214,4 Ref=off

Peak #	RetTime [min]	Type	Width [min]	Area [mAU*s]	Height [mAU]	Area %
1	15.185	BV	0.1332	3926.07056	443.90045	69.1646
2	15.923	VV	0.1472	1750.34875	171.31154	30.8354

Totals : 5676.41931 615.21199

Signal 2: DAD1 G, Sig=280,4 Ref=off

Peak #	RetTime [min]	Type	Width [min]	Area [mAU*s]	Height [mAU]	Area %
1	15.186	BB	0.1170	283.41089	37.13812	68.8641
2	15.923	BV	0.1280	128.14029	14.95491	31.1359

Totals : 411.55118 52.09303

Signal 3: MSD1 TIC, MS File

Peak #	RetTime [min]	Type	Width [min]	Area	Height	Area %
1	15.283	BV	0.1292	2.38333e7	2.65855e6	78.2120
2	16.013	VV	0.1366	6.63939e6	7.46768e5	21.7880

Totals : 3.04727e7 3.40532e6

==
*** End of Report ***

Spectrum Deconvolution
Data File D:\EZXDATA\MARIARODRIGUEZ\12-24\121224-10-75OVER20_PEPTIDES-C3-30650.D
Sample Name: Brd4BD2_74_117_12dec24

```
    Easy-Access Method: '10-75over20_C3'
    =====================================================================
    Acq. Operator   : Maria Rodriguez
    Acq. Instrument : INSTRUMENT 1                     Location : P1-C-09
    Injection Date  : 12/13/2024 12:36:15 AM                Inj :   1
                                                    Inj Volume : 10.000 µl
    Acq. Method     : C:\CHEM32\1\METHODS\10-75OVER20_PEPTIDE
    Last changed    : 12/13/2024 12:35:17 AM by Maria Rodriguez
                      (modified after loading)
    Analysis Method : C:\CHEM32\1\METHODS\10-75OVER20_PEPTIDES-5UL-C3.M
    Last changed    : 12/19/2024 5:35:54 PM by Liam Hales
                      (modified after loading)
    Sample Info     : Easy-Access Method: '10-75over20_C3'

    Additional Info : Peak(s) manually integrated

    =====================================================================
                          Deconvolution Parameters
    =====================================================================

    Adduct Ion(Positive): +H, 1.0079 Da
    Adduct Ion(Negative):  , 0.0000 Da
    Low MW:               9000
    DeconvStartChgMaximum Charge:       50
    Minimum Peaks in Set: 3
    Retain Residual:      No
    Ion PWHH:             0.6 Da
    MW Agreement:         0.05 %
    Noise Cutoff:         1000 counts
    Abundance Cutoff:     10 %
    MW Assign:            Curve fit
    MW Assign Cutoff:     40 %
    Envelope Cutoff:      50 %
```

Spectrum Deconvolution
Data File D:\EZXDATA\MARIARODRIGUEZ\12-24\121224-10-75OVER20_PEPTIDES-C3-30650.D
Sample Name: Brd4BD2_74_117_12dec24

Deconvolution of Spectrum # 1 @ 15.049 - 15.706 min

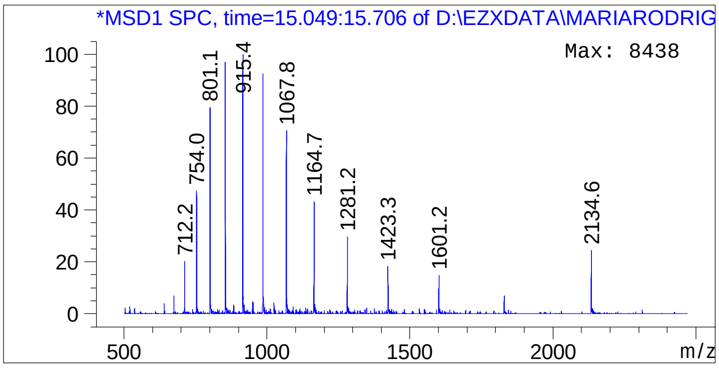

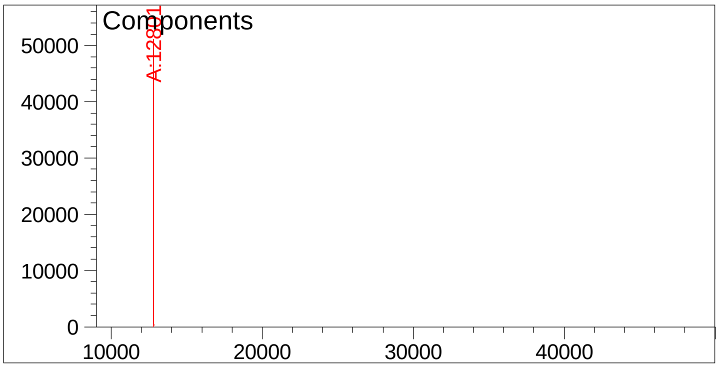

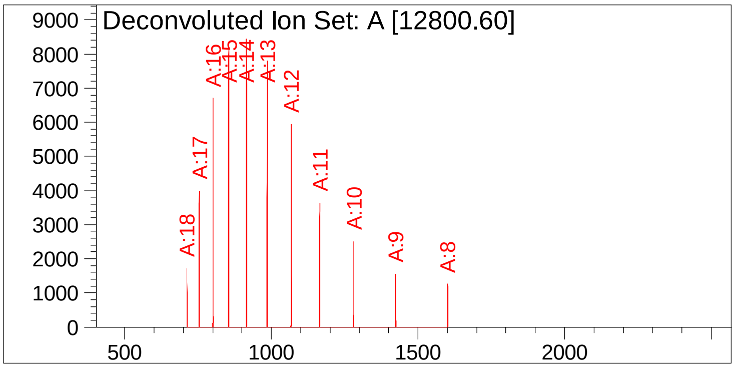

Component	Molecular Weight	Absolute Abundance	Relative Abundance
A	12800.60	51114	100.00

*** End of Report ***

Spectrum Deconvolution
Data File D:\EZXDATA\MARIARODRIGUEZ\12-24\121224-10-75OVER20_PEPTIDES-C3-30650.D
Sample Name: Brd4BD2_74_117_12dec24

```
    Easy-Access Method: '10-75over20_C3'
    =====================================================================
    Acq. Operator   : Maria Rodriguez
    Acq. Instrument : INSTRUMENT 1                    Location : P1-C-09
    Injection Date  : 12/13/2024 12:36:15 AM               Inj :   1
                                                    Inj Volume : 10.000 µl
    Acq. Method     : C:\CHEM32\1\METHODS\10-75OVER20_PEPTIDE
    Last changed    : 12/13/2024 12:35:17 AM by Maria Rodriguez
                      (modified after loading)
    Analysis Method : C:\CHEM32\1\METHODS\10-75OVER20_PEPTIDES-5UL-C3.M
    Last changed    : 12/19/2024 5:35:35 PM by Liam Hales
                      (modified after loading)
    Sample Info     : Easy-Access Method: '10-75over20_C3'

    Additional Info : Peak(s) manually integrated

    =====================================================================
                          Deconvolution Parameters
    =====================================================================

    Adduct Ion(Positive): +H, 1.0079 Da
    Adduct Ion(Negative):  , 0.0000 Da
    Low MW:               9000
    DeconvStartChgMaximum Charge:       50
    Minimum Peaks in Set: 3
    Retain Residual:      No
    Ion PWHH:             0.6 Da
    MW Agreement:         0.05 %
    Noise Cutoff:         1000 counts
    Abundance Cutoff:     10 %
    MW Assign:            Curve fit
    MW Assign Cutoff:     40 %
    Envelope Cutoff:      50 %
```

Deconvolution of Spectrum # 1 @ 15.829 - 16.541 min

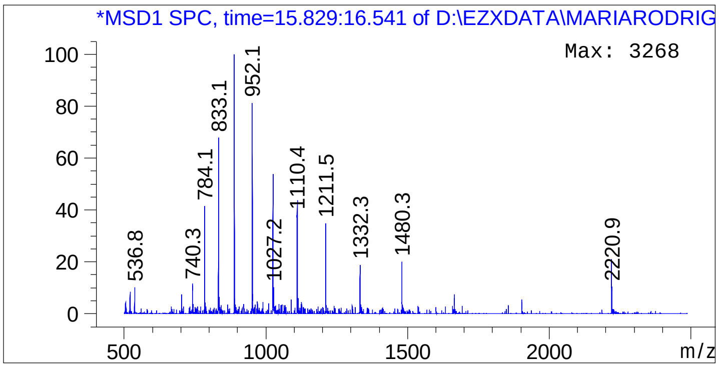

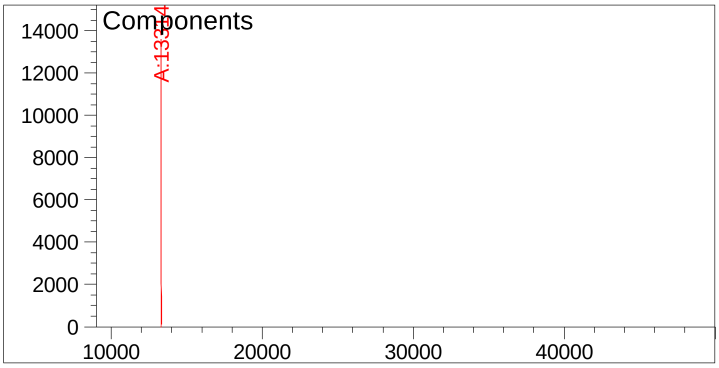

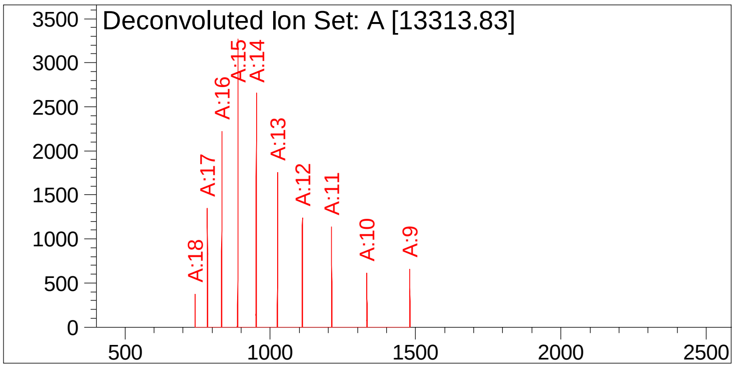

Component	Molecular Weight	Absolute Abundance	Relative Abundance
A	13313.83	13598	100.00

*** End of Report ***

Data File D:\EZXDATA\MARIARODRIGUEZ\12-24\121224-10-75OVER20_PEPTIDES-C3-30651.D
Sample Name: Brd4BD2_74_119_12dec24

===

Acq. Operator : Maria Rodriguez
Acq. Instrument : INSTRUMENT 1 Location : P1-D-01
Injection Date : 12/13/2024 1:06:11 AM Inj : 1
Inj Volume : 10.000 µl
Acq. Method : C:\CHEM32\1\METHODS\10-75OVER20_PEPTIDE
Last changed : 12/13/2024 1:05:16 AM by Maria Rodriguez
(modified after loading)
Analysis Method : C:\CHEM32\1\METHODS\10-75OVER20_PEPTIDES-5UL-C3.M
Last changed : 12/19/2024 5:37:37 PM by Liam Hales
(modified after loading)
Sample Info : Easy-Access Method: '10-75over20_C3'

Additional Info : Peak(s) manually integrated

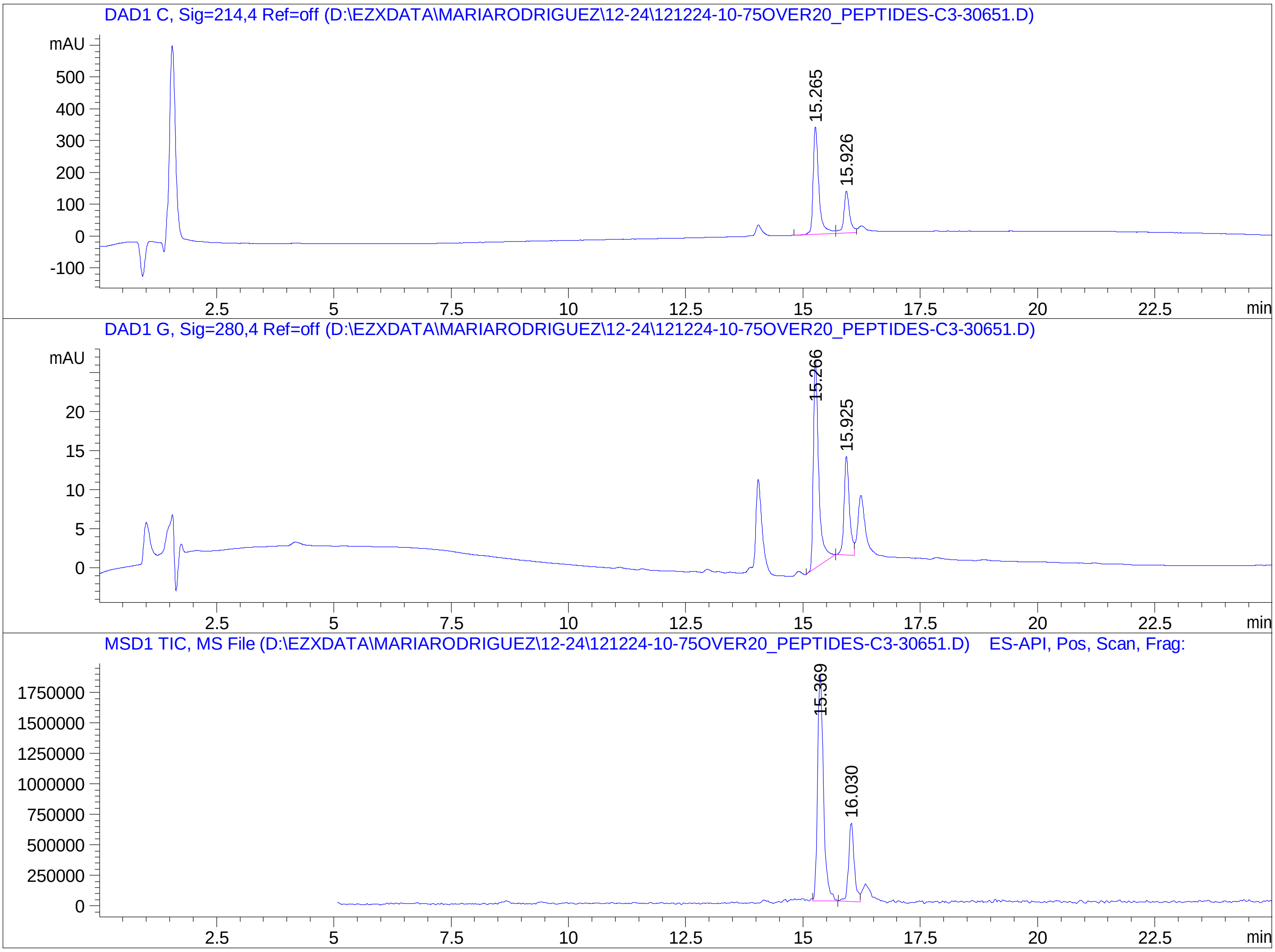

===

Area Percent Report

===

Sorted By : Signal
Multiplier : 1.0000
Dilution : 1.0000
Sample Amount: : 15.00000 [ng/ul] (not used in calc.)
Use Multiplier & Dilution Factor with ISTDs

Signal 1: DAD1 C, Sig=214,4 Ref=off

Peak #	RetTime [min]	Type	Width [min]	Area [mAU*s]	Height [mAU]	Area %
1	15.265	BV	0.1268	2860.91431	337.75491	71.6541
2	15.926	VV	0.1267	1131.75781	131.10789	28.3459
Totals :				3992.67212	468.86281	

Signal 2: DAD1 G, Sig=280,4 Ref=off

Peak #	RetTime [min]	Type	Width [min]	Area [mAU*s]	Height [mAU]	Area %
1	15.266	BB	0.1189	212.22273	26.64262	67.7713
2	15.925	BV	0.1191	100.92262	12.64079	32.2287
Totals :				313.14535	39.28342	

Signal 3: MSD1 TIC, MS File

Peak #	RetTime [min]	Type	Width [min]	Area	Height	Area %
1	15.369	VB	0.1351	1.63350e7	1.86305e6	75.0101
2	16.030	BV	0.1313	5.44208e6	6.44706e5	24.9899
Totals :				2.17771e7	2.50775e6	

===

*** End of Report ***

Spectrum Deconvolution
Data File D:\EZXDATA\MARIARODRIGUEZ\12-24\121224-10-75OVER20_PEPTIDES-C3-30651.D
Sample Name: Brd4BD2_74_119_12dec24

```
    Easy-Access Method: '10-75over20_C3'
    =====================================================================
    Acq. Operator   : Maria Rodriguez
    Acq. Instrument : INSTRUMENT 1                    Location : P1-D-01
    Injection Date  : 12/13/2024 1:06:11 AM                Inj :   1
                                                    Inj Volume : 10.000 µl
    Acq. Method     : C:\CHEM32\1\METHODS\10-75OVER20_PEPTIDE
    Last changed    : 12/13/2024 1:05:16 AM by Maria Rodriguez
                      (modified after loading)
    Analysis Method : C:\CHEM32\1\METHODS\10-75OVER20_PEPTIDES-5UL-C3.M
    Last changed    : 12/19/2024 5:37:17 PM by Liam Hales
                      (modified after loading)
    Sample Info     : Easy-Access Method: '10-75over20_C3'

    Additional Info : Peak(s) manually integrated

    =====================================================================
                          Deconvolution Parameters
    =====================================================================

    Adduct Ion(Positive): +H, 1.0079 Da
    Adduct Ion(Negative):  , 0.0000 Da
    Low MW:               9000
    DeconvStartChgMaximum Charge:       50
    Minimum Peaks in Set: 3
    Retain Residual:      No
    Ion PWHH:             0.6 Da
    MW Agreement:         0.05 %
    Noise Cutoff:         1000 counts
    Abundance Cutoff:     10 %
    MW Assign:            Curve fit
    MW Assign Cutoff:     40 %
    Envelope Cutoff:      50 %
```

Spectrum Deconvolution
Data File D:\EZXDATA\MARIARODRIGUEZ\12-24\121224-10-75OVER20_PEPTIDES-C3-30651.D
Sample Name: Brd4BD2_74_119_12dec24

Deconvolution of Spectrum # 1 @ 15.432 min

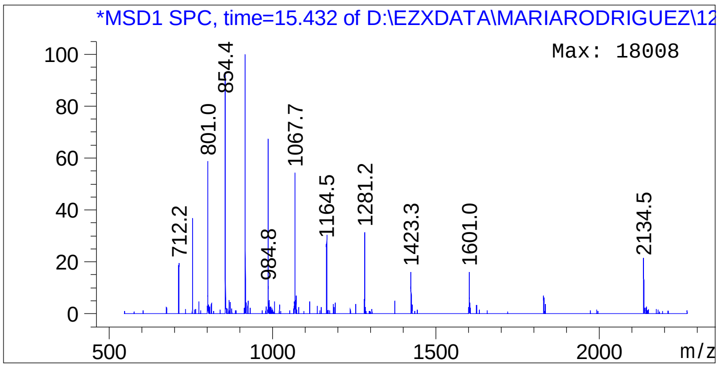

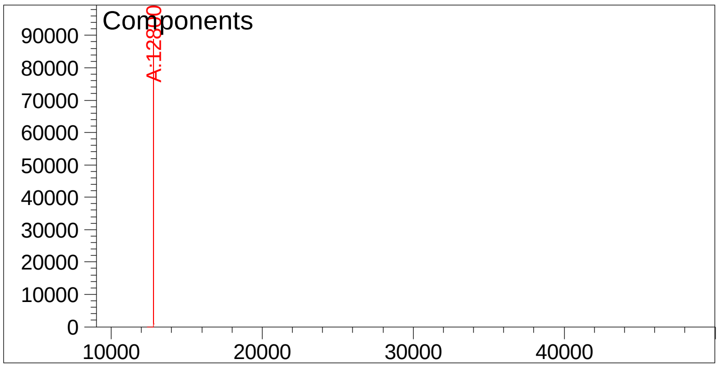

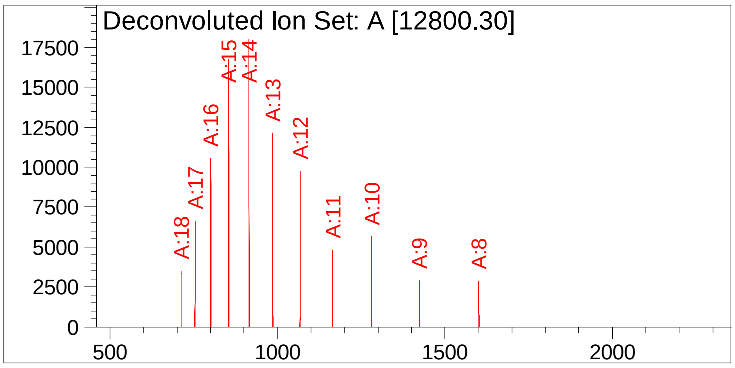

Component	Molecular Weight	Absolute Abundance	Relative Abundance
A	12800.30	88811	100.00

*** End of Report ***

Spectrum Deconvolution
Data File D:\EZXDATA\MARIARODRIGUEZ\12-24\121224-10-75OVER20_PEPTIDES-C3-30651.D
Sample Name: Brd4BD2_74_119_12dec24

Easy-Access Method: '10-75over20_C3'

===

```
Acq. Operator   : Maria Rodriguez
Acq. Instrument : INSTRUMENT 1                    Location : P1-D-01
Injection Date  : 12/13/2024 1:06:11 AM                Inj :   1
                                                Inj Volume : 10.000 µl
Acq. Method     : C:\CHEM32\1\METHODS\10-75OVER20_PEPTIDE
Last changed    : 12/13/2024 1:05:16 AM by Maria Rodriguez
                  (modified after loading)
Analysis Method : C:\CHEM32\1\METHODS\10-75OVER20_PEPTIDES-5UL-C3.M
Last changed    : 12/19/2024 5:37:35 PM by Liam Hales
                  (modified after loading)
Sample Info     : Easy-Access Method: '10-75over20_C3'

Additional Info : Peak(s) manually integrated
```

===

Deconvolution Parameters

===

```
Adduct Ion(Positive): +H, 1.0079 Da
Adduct Ion(Negative):  , 0.0000 Da
Low MW:               9000
DeconvStartChgMaximum Charge:       50
Minimum Peaks in Set: 3
Retain Residual:      No
Ion PWHH:             0.6 Da
MW Agreement:         0.05 %
Noise Cutoff:         1000 counts
Abundance Cutoff:     10 %
MW Assign:            Curve fit
MW Assign Cutoff:     40 %
Envelope Cutoff:      50 %
```

Spectrum Deconvolution
Data File D:\EZXDATA\MARIARODRIGUEZ\12-24\121224-10-75OVER20_PEPTIDES-C3-30651.D
Sample Name: Brd4BD2_74_119_12dec24

Deconvolution of Spectrum # 1 @ 15.911 - 16.869 min

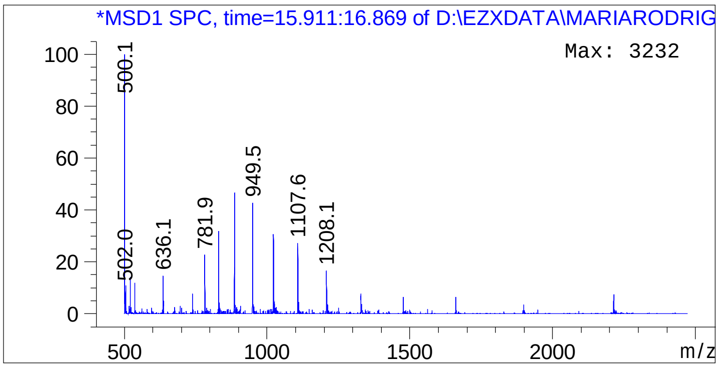

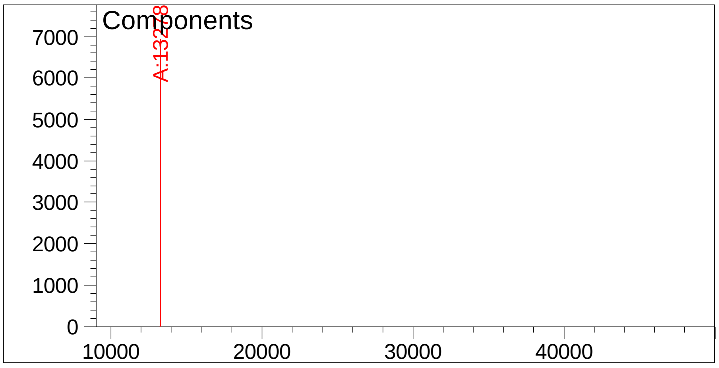

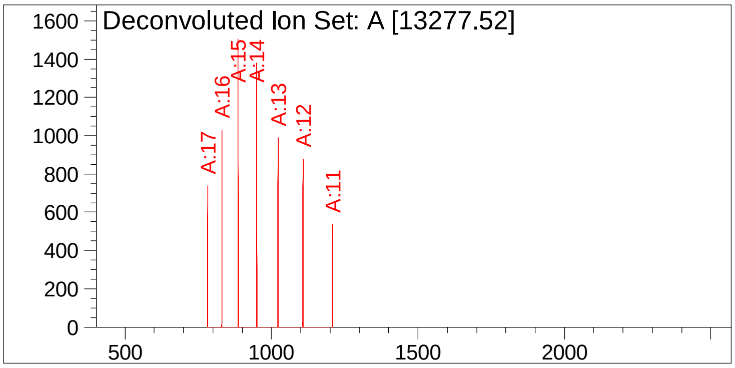

Component	Molecular Weight	Absolute Abundance	Relative Abundance
A	13277.52	6941	100.00

*** End of Report ***

===

Acq. Operator : Maria Rodriguez
Acq. Instrument : INSTRUMENT 1 Location : P1-C-01
Injection Date : 12/12/2024 8:36:33 PM Inj : 1
Inj Volume : 10.000 µl
Acq. Method : C:\CHEM32\1\METHODS\10-75OVER20_PEPTIDE
Last changed : 12/12/2024 8:35:37 PM by Maria Rodriguez
(modified after loading)
Analysis Method : C:\CHEM32\1\METHODS\10-75OVER20_PEPTIDES-5UL-C3.M
Last changed : 12/19/2024 5:21:15 PM by Liam Hales
(modified after loading)
Sample Info : Easy-Access Method: '10-75over20_C3'

Additional Info : Peak(s) manually integrated

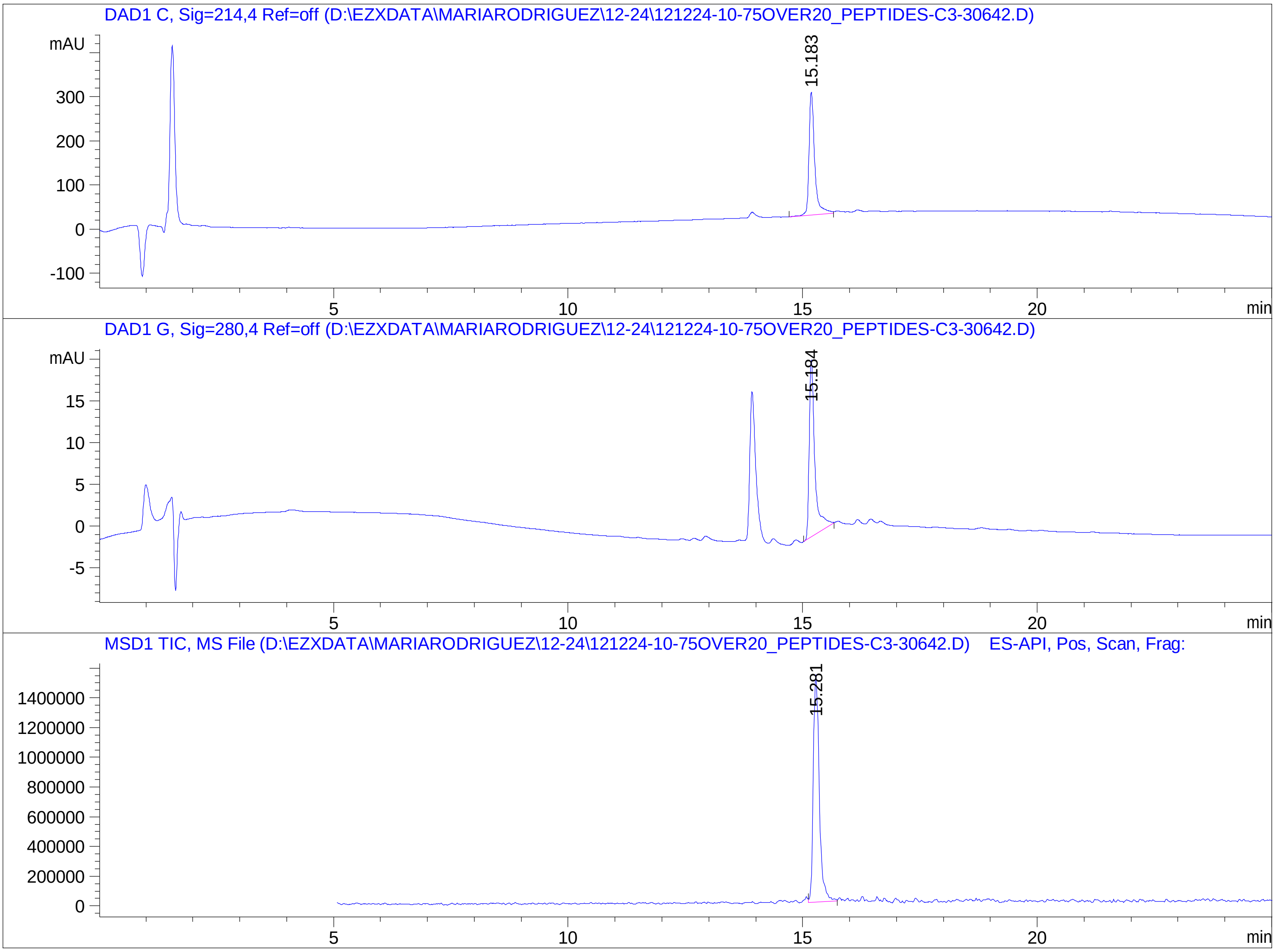

===

Area Percent Report

===

Sorted By : Signal
Multiplier : 1.0000
Dilution : 1.0000
Sample Amount: : 15.00000 [ng/ul] (not used in calc.)
Use Multiplier & Dilution Factor with ISTDs

Signal 1: DAD1 C, Sig=214,4 Ref=off

Peak #	RetTime [min]	Type	Width [min]	Area [mAU*s]	Height [mAU]	Area %
1	15.183	BV	0.1272	2364.49707	278.19254	100.0000
Totals :				2364.49707	278.19254	

Signal 2: DAD1 G, Sig=280,4 Ref=off

Peak #	RetTime [min]	Type	Width [min]	Area [mAU*s]	Height [mAU]	Area %
1	15.184	BB	0.1254	176.61708	21.15519	100.0000
Totals :				176.61708	21.15519	

Signal 3: MSD1 TIC, MS File

Peak #	RetTime [min]	Type	Width [min]	Area	Height	Area %
1	15.281	VV	0.1410	1.36432e7	1.52930e6	100.0000
Totals :				1.36432e7	1.52930e6	

===

*** End of Report ***

Easy-Access Method: '10-75over20_C3'

===

Acq. Operator : Maria Rodriguez
Acq. Instrument : INSTRUMENT 1 Location : P1-C-01
Injection Date : 12/12/2024 8:36:33 PM Inj : 1
Inj Volume : 10.000 µl
Acq. Method : C:\CHEM32\1\METHODS\10-75OVER20_PEPTIDE
Last changed : 12/12/2024 8:35:37 PM by Maria Rodriguez
(modified after loading)
Analysis Method : C:\CHEM32\1\METHODS\10-75OVER20_PEPTIDES-5UL-C3.M
Last changed : 12/19/2024 5:21:15 PM by Liam Hales
(modified after loading)
Sample Info : Easy-Access Method: '10-75over20_C3'

Additional Info : Peak(s) manually integrated

===

Deconvolution Parameters

===

Adduct Ion(Positive): +H, 1.0079 Da
Adduct Ion(Negative): , 0.0000 Da
Low MW: 5000
DeconvStartChgMaximum Charge: 50
Minimum Peaks in Set: 3
Retain Residual: No
Ion PWHH: 0.6 Da
MW Agreement: 0.05 %
Noise Cutoff: 1000 counts
Abundance Cutoff: 10 %
MW Assign: Curve fit
MW Assign Cutoff: 40 %
Envelope Cutoff: 50 %

Spectrum Deconvolution
Data File D:\EZXDATA\MARIARODRIGUEZ\12-24\121224-10-75OVER20_PEPTIDES-C3-30642.D
Sample Name: Brd4BD2_74_112_12dec24

Deconvolution of Spectrum #   1 @ 14.584 - 16.308 min

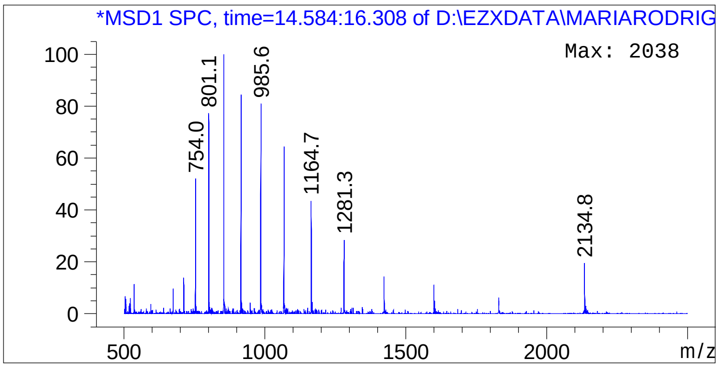

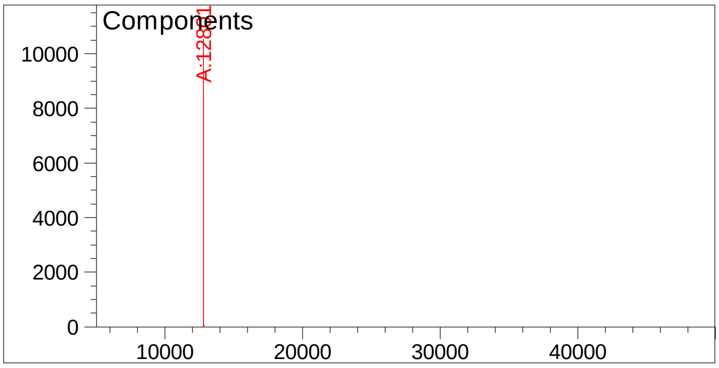

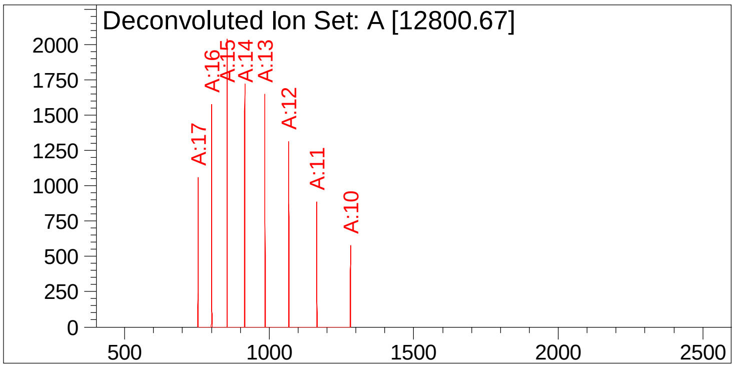

Component	Molecular Weight	Absolute Abundance	Relative Abundance
A	12800.67	10533	100.00

*** End of Report ***
