# Supplementary material for: BromoCatch: a self-labelling tag platform for protein modification and live cell imaging
Source: Nat Commun. 2026 May 13;17:6406. doi: 10.1038/s41467-026-72539-w (PMC13376172; doi:10.1038/s41467-026-72539-w)
Supplement: Supplementary file 3 — Supplementary Data 1 [file 41467_2026_72539_MOESM3_ESM.zip › PUBLICATION INTACT MS/Suplementary Figure 2 Protein Controls.pdf]

===

Acq. Operator   : Maria Rodriguez
Acq. Instrument : INSTRUMENT 1   Location : P2-A-04
Injection Date : 7/8/2024 6:43:59 PM   Inj :   1
                                            Inj Volume : 3.000 µl
Acq. Method   : C:\CHEM32\1\METHODS\10-75OVER20_PEPTIDE
Last changed   : 7/8/2024 6:43:07 PM by Maria Rodriguez
                  (modified after loading)
Analysis Method : C:\CHEM32\1\METHODS\10-75OVER20_PEPTIDES-C3.M
Last changed   : 10/10/2024 10:16:46 AM by Kevin Haubrich
                  (modified after loading)
Sample Info   : Easy-Access Method: '10-75over20_C3'

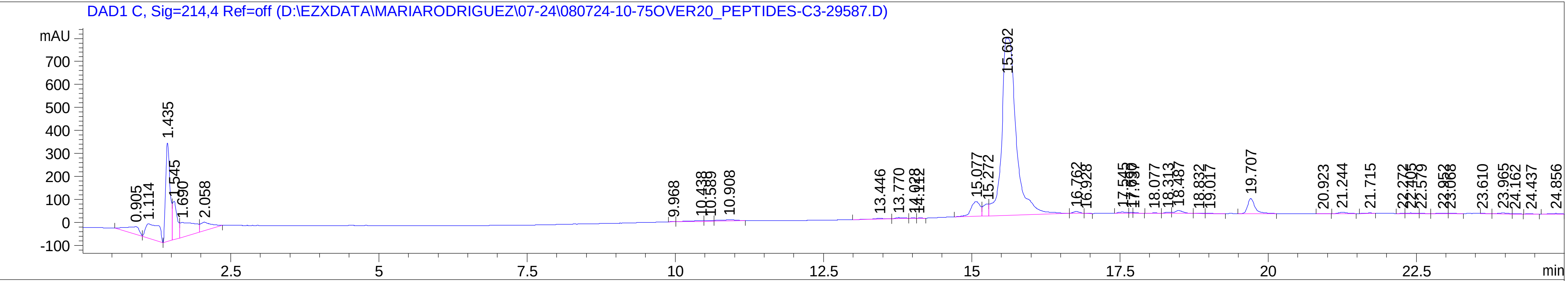

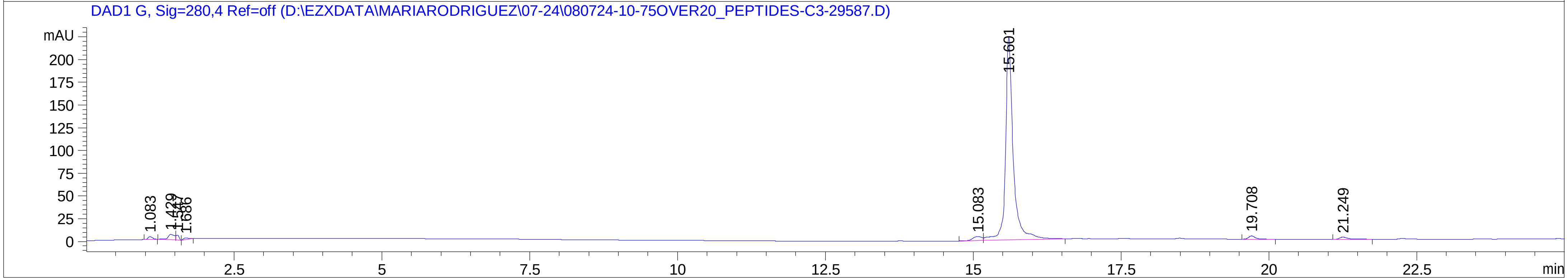

Data File D:\EZXDATA\MARIARODRIGUEZ\07-24\080724-10-75OVER20_PEPTIDES-C3-29587.D
Sample Name: MR_brd4BD2_L387A_E438Csh

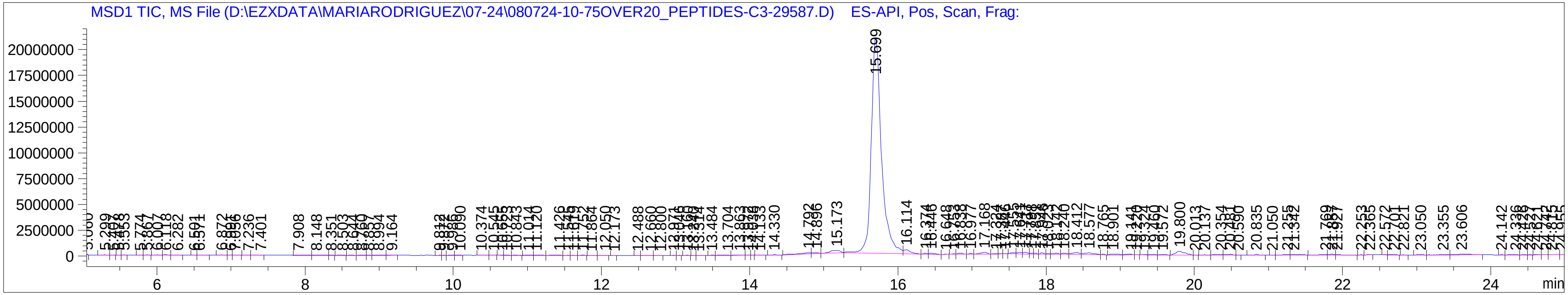

Area Percent Report

Sorted By	:	Signal
Multiplier	:	1.0000
Dilution	:	1.0000
Sample Amount:	:	15.00000 [ng/ul] (not used in calc.)

Use Multiplier & Dilution Factor with ISTDs

Signal 1: DAD1 C, Sig=214,4 Ref=off

Peak #	RetTime [min]	Type	Width [min]	Area [mAU*s]	Height [mAU]	Area %
1	0.905	BV	0.1946	492.27896	33.67065	2.2604
2	1.114	VB	0.2251	1069.33167	62.66356	4.9101
3	1.435	BV	0.0784	2223.06738	429.22382	10.2078
4	1.545	VV	0.0806	917.95801	165.78879	4.2150
5	1.690	VV	0.2042	1012.26758	62.76615	4.6481
6	2.058	VB	0.1714	474.99420	36.60890	2.1811
7	9.968	BV	0.0855	5.62383	1.10345	0.0258
8	10.438	VV	0.2100	62.44225	3.64329	0.2867
9	10.589	VV	0.1205	35.43103	3.73957	0.1627
10	10.908	VB	0.2696	111.96951	5.31993	0.5141

Peak #	RetTime [min]	Type	Width [min]	Area [mAU*s]	Height [mAU]	Area %
11	13.446	BB	0.1928	47.80000	3.11963	0.2195
12	13.770	BV	0.1451	50.39294	4.85678	0.2314
13	14.028	VV	0.0963	12.31786	1.70464	0.0566
14	14.112	VB	0.0673	8.26248	1.57307	0.0379
15	15.077	BV	0.1691	681.50226	63.53911	3.1293
16	15.272	VV	0.0952	323.22214	51.52110	1.4842
17	15.602	VB	0.2513	1.30181e4	769.89581	59.7759
18	16.762	BV	0.1129	63.08064	8.27938	0.2897
19	16.928	VB	0.0807	8.52912	1.40833	0.0392
20	17.545	BV	0.1407	47.93841	5.34234	0.2201
21	17.690	VV	0.0539	13.98002	3.40433	0.0642
22	17.737	VB	0.0727	18.64885	3.36293	0.0856
23	18.077	BB	0.1240	16.13069	1.68062	0.0741
24	18.313	BV	0.1043	32.42897	4.83347	0.1489
25	18.487	VV	0.1493	119.88888	12.13420	0.5505
26	18.832	VV	0.1215	11.65780	1.17725	0.0535
27	19.017	VB	0.0990	12.79818	1.75740	0.0588
28	19.707	VV	0.1305	579.07336	65.92276	2.6590
29	20.923	VB	0.1110	9.08302	1.13984	0.0417
30	21.244	BV	0.1461	65.28366	5.85298	0.2998
31	21.715	BV	0.1192	18.45162	2.00863	0.0847
32	22.272	BV	0.0669	7.10888	1.51137	0.0326
33	22.405	VV	0.1419	29.91452	2.81696	0.1374
34	22.579	VB	0.0906	11.42973	1.61366	0.0525
35	22.952	BV	0.1363	32.19998	2.92713	0.1479
36	23.068	VB	0.1012	18.23903	2.38435	0.0837
37	23.610	VB	0.0931	11.56634	1.62315	0.0531
38	23.965	BV	0.1468	49.04895	4.30981	0.2252
39	24.162	VB	0.0974	11.74826	1.49925	0.0539
40	24.437	BB	0.0917	9.85588	1.40747	0.0453
41	24.856	BBA	0.1605	33.12257	2.71249	0.1521
Totals :				2.17782e4	1841.84834	

Signal 2: DAD1 G, Sig=280,4 Ref=off

Peak #	RetTime [min]	Type	Width [min]	Area [mAU*s]	Height [mAU]	Area %
1	1.083	BB	0.0871	16.01343	2.96201	0.7190
2	1.429	BV	0.1061	46.13834	5.97675	2.0716
3	1.547	VB	0.0540	20.34844	5.64686	0.9137
4	1.686	BB	0.0951	12.13605	1.99216	0.5449
5	15.083	BV	0.1659	47.71276	4.56568	2.1423
6	15.601	VB	0.1302	2029.91492	222.91757	91.1444
7	19.708	BB	0.1235	29.53149	3.60929	1.3260
8	21.249	BB	0.1529	25.34645	2.44622	1.1381
Totals :				2227.14188	250.11654	

Signal 3: MSD1 TIC, MS File

Peak #	RetTime [min]	Type	Width [min]	Area	Height	Area %
1	5.060	BB	0.0146	4.16793e4	4.75340e4	0.0178
2	5.299	BV	0.0686	1.46338e5	3.27254e4	0.0624
3	5.407	VB	0.0394	4.53834e4	1.92022e4	0.0193
4	5.478	BB	0.0366	4.95697e4	2.25643e4	0.0211
5	5.553	BB	0.0404	4.66871e4	1.93639e4	0.0199
6	5.774	BV	0.0712	8.69036e4	2.00140e4	0.0370
7	5.867	VB	0.0517	8.79564e4	2.60626e4	0.0375
8	6.007	BV	0.0804	1.26553e5	2.30611e4	0.0539
9	6.118	VB	0.0595	1.08201e5	3.03134e4	0.0461
10	6.282	BB	0.0546	4.55508e4	1.14637e4	0.0194
11	6.501	BV	0.0359	3.18841e4	1.48149e4	0.0136
12	6.571	VB	0.0918	8.08164e4	1.18582e4	0.0344
13	6.872	BB	0.0659	1.39221e5	3.02925e4	0.0593
14	6.991	BV	0.0324	4.23413e4	2.04107e4	0.0180
15	7.056	VB	0.0526	8.64504e4	2.50599e4	0.0368

Peak #	RetTime [min]	Type	Width [min]	Area	Height	Area %
16	7.236	BB	0.0483	5.57820e4	1.80718e4	0.0238
17	7.401	BB	0.0653	1.02606e5	2.09703e4	0.0437
18	7.908	BV	0.1195	3.29650e5	3.72981e4	0.1405
19	8.148	VV	0.0923	2.40861e5	3.70357e4	0.1026
20	8.351	VB	0.0515	4.23764e4	1.37130e4	0.0181
21	8.503	BB	0.0694	9.08141e4	2.16564e4	0.0387
22	8.644	BB	0.0652	9.22529e4	2.03344e4	0.0393
23	8.760	BV	0.0780	1.39714e5	3.05397e4	0.0595
24	8.857	VV	0.0451	5.41427e4	2.00244e4	0.0231
25	8.994	VV	0.0856	1.65170e5	3.15819e4	0.0704
26	9.164	VB	0.0755	8.97065e4	1.66052e4	0.0382
27	9.812	BV	0.0434	5.96091e4	2.67915e4	0.0254
28	9.872	VV	0.0427	3.97749e4	1.55238e4	0.0170
29	9.986	VV	0.0663	6.37506e4	1.48950e4	0.0272
30	10.090	VB	0.0802	5.90017e4	1.36386e4	0.0251
31	10.374	BV	0.0900	1.32677e5	1.99278e4	0.0565
32	10.545	VB	0.0475	7.01252e4	2.08210e4	0.0299
33	10.655	BV	0.0493	5.75909e4	1.81511e4	0.0245
34	10.723	VB	0.0549	7.21880e4	2.18835e4	0.0308
35	10.843	BB	0.0405	6.89684e4	2.49289e4	0.0294
36	11.014	BV	0.0778	1.76508e5	3.14522e4	0.0752
37	11.120	VB	0.0875	1.23030e5	1.80961e4	0.0524
38	11.426	BB	0.0988	1.91900e5	2.71751e4	0.0818
39	11.545	BV	0.0467	3.80695e4	1.15309e4	0.0162
40	11.619	VV	0.0582	4.69333e4	1.34325e4	0.0200
41	11.752	VB	0.0549	9.30524e4	2.81877e4	0.0397
42	11.864	BB	0.0816	9.16577e4	1.75181e4	0.0391
43	12.050	BB	0.0886	1.03737e5	1.77712e4	0.0442
44	12.173	BB	0.0321	2.78835e4	1.36152e4	0.0119
45	12.488	BV	0.0447	5.11993e4	1.94273e4	0.0218
46	12.660	VB	0.0916	8.14333e4	1.41772e4	0.0347
47	12.800	BB	0.0615	4.41040e4	1.04555e4	0.0188
48	12.971	BV	0.0462	5.19060e4	2.02985e4	0.0221
49	13.046	VB	0.0371	3.27641e4	1.32413e4	0.0140
50	13.160	BB	0.0389	4.03261e4	1.53328e4	0.0172
51	13.240	BV	0.0360	1.90725e4	8015.17041	8.128e-3

Peak #	RetTime [min]	Type	Width [min]	Area	Height	Area %
52	13.314	VB	0.0536	3.99908e4	1.13048e4	0.0170
53	13.484	BV	0.0511	8.71904e4	2.37178e4	0.0372
54	13.704	VV	0.1239	1.83793e5	1.95788e4	0.0783
55	13.863	VV	0.0904	2.05040e5	3.42467e4	0.0874
56	13.972	VV	0.0401	6.37604e4	2.67941e4	0.0272
57	14.038	VB	0.0360	3.32378e4	1.77731e4	0.0142
58	14.133	BB	0.0657	9.01539e4	1.82890e4	0.0384
59	14.330	BB	0.0733	1.29895e5	2.87208e4	0.0554
60	14.792	BV	0.1404	1.26148e6	1.18723e5	0.5376
61	14.896	VV	0.1068	6.03734e5	9.42372e4	0.2573
62	15.173	VB	0.1426	2.31242e6	2.45693e5	0.9855
63	15.699	BV	0.1456	2.01601e8	2.08653e7	85.9142
64	16.114	VB	0.0930	2.65975e6	4.05205e5	1.1335
65	16.374	BV	0.0579	3.16806e5	9.23212e4	0.1350
66	16.446	VB	0.0767	6.42105e5	1.16316e5	0.2736
67	16.648	BB	0.0519	1.21229e5	3.57498e4	0.0517
68	16.753	BV	0.0500	1.51294e5	4.68052e4	0.0645
69	16.838	VB	0.0855	5.62805e5	1.07780e5	0.2398
70	16.977	BV	0.0519	2.55793e5	8.60408e4	0.1090
71	17.168	VB	0.0858	1.16649e6	1.96109e5	0.4971
72	17.324	BV	0.0562	1.60097e5	4.35982e4	0.0682
73	17.387	VV	0.0509	1.22866e5	4.02328e4	0.0524
74	17.446	VB	0.0520	1.13668e5	3.75887e4	0.0484
75	17.555	BV	0.0619	5.35847e5	1.25998e5	0.2284
76	17.641	VV	0.0655	7.04749e5	1.54630e5	0.3003
77	17.717	VV	0.0719	7.01538e5	1.62587e5	0.2990
78	17.798	VV	0.0459	2.41769e5	8.77883e4	0.1030
79	17.851	VB	0.0508	2.46610e5	8.76968e4	0.1051
80	17.946	BV	0.0502	4.00650e5	1.38300e5	0.1707
81	18.023	VV	0.0554	8.16153e4	2.44990e4	0.0348
82	18.142	VB	0.0695	3.35282e5	7.36184e4	0.1429
83	18.240	BV	0.0684	4.59399e5	1.11909e5	0.1958
84	18.412	VV	0.1009	1.23285e6	1.83434e5	0.5254
85	18.577	VV	0.1194	1.69530e6	1.92093e5	0.7225
86	18.765	VB	0.0461	2.09289e5	7.21761e4	0.0892
87	18.901	BV	0.1114	6.98844e5	8.96771e4	0.2978

Peak #	RetTime [min]	Type	Width [min]	Area	Height	Area %
88	19.141	VV	0.0842	4.35612e5	7.06788e4	0.1856
89	19.220	VB	0.0438	5.50573e4	2.03722e4	0.0235
90	19.324	BB	0.0502	7.41974e4	2.28704e4	0.0316
91	19.460	BV	0.0568	1.15941e5	3.34948e4	0.0494
92	19.572	VB	0.0820	2.01405e5	3.58438e4	0.0858
93	19.800	BV	0.1215	3.11041e6	3.59442e5	1.3255
94	20.013	VB	0.0475	6.95576e4	2.30694e4	0.0296
95	20.137	BB	0.0717	1.25450e5	2.30697e4	0.0535
96	20.354	BV	0.1245	3.58626e5	4.85328e4	0.1528
97	20.481	VB	0.0770	3.73016e5	6.72232e4	0.1590
98	20.590	BB	0.0332	3.53459e4	1.77688e4	0.0151
99	20.835	BB	0.1068	3.56021e5	4.21340e4	0.1517
100	21.050	BB	0.0331	2.81715e4	1.32138e4	0.0120
101	21.255	BV	0.0815	4.12295e5	7.07055e4	0.1757
102	21.342	VB	0.1409	5.23479e5	6.18999e4	0.2231
103	21.769	BV	0.0964	3.86961e5	5.42010e4	0.1649
104	21.857	VV	0.0702	3.75620e5	7.06868e4	0.1601
105	21.927	VB	0.0814	3.09744e5	4.93962e4	0.1320
106	22.253	BB	0.0486	7.81808e4	2.83221e4	0.0333
107	22.365	BB	0.0561	6.20148e4	1.65043e4	0.0264
108	22.572	BV	0.0418	3.45021e4	1.41368e4	0.0147
109	22.701	VB	0.0997	1.66135e5	2.58246e4	0.0708
110	22.821	BB	0.0444	7.95032e4	2.98438e4	0.0339
111	23.050	BV	0.0921	2.43063e5	3.38052e4	0.1036
112	23.355	VV	0.1508	2.61175e5	2.20071e4	0.1113
113	23.606	VB	0.1544	7.13278e5	6.21654e4	0.3040
114	24.142	BV	0.0484	5.84845e4	1.89141e4	0.0249
115	24.336	VV	0.0848	2.80708e5	4.51948e4	0.1196
116	24.426	VV	0.0670	6.65784e4	1.65739e4	0.0284
117	24.527	VB	0.0360	2.91339e4	1.43738e4	0.0124
118	24.621	BB	0.0642	8.35149e4	2.22825e4	0.0356
119	24.742	BV	0.0563	1.29261e5	3.78524e4	0.0551
120	24.815	VV	0.0808	1.64690e5	3.23748e4	0.0702
121	24.955	VBA	0.0379	2.24057e4	9841.48730	9.548e-3

Totals :				2.34653e8	2.68101e7	

===

*** End of Report ***

Spectrum Deconvolution
Data File D:\EZXDATA\MARIARODRIGUEZ\07-24\080724-10-75OVER20_PEPTIDES-C3_200M-29589.D
Sample Name: MR_Brd4BD2_L387A_M442C

```
    Easy-Access Method: '10-75over20-C3(200+m/Z)'
    =====================================================================
    Acq. Operator   : Maria Rodriguez
    Acq. Instrument : INSTRUMENT 1                    Location : P2-A-06
    Injection Date  : 7/8/2024 7:43:43 PM                  Inj :   1
                                                   Inj Volume : 3.000 µl
    Acq. Method     : C:\CHEM32\1\METHODS\10-75OVER20_PEPTIDE
    Last changed    : 7/8/2024 7:42:55 PM by Maria Rodriguez
                      (modified after loading)
    Analysis Method : C:\CHEM32\1\METHODS\10-75OVER20_PEPTIDES-C3.M
    Last changed    : 10/10/2024 10:16:44 AM by Kevin Haubrich
                      (modified after loading)
    Sample Info     : Easy-Access Method: '10-75over20-C3(200+m/Z)'


    =====================================================================
                          Deconvolution Parameters
    =====================================================================

    Adduct Ion(Positive): +H, 1.0079 Da
    Adduct Ion(Negative):  , 0.0000 Da
    Low MW:               10000
    DeconvStartChgMaximum Charge:       50
    Minimum Peaks in Set: 3
    Retain Residual:      No
    Ion PWHH:             0.6 Da
    MW Agreement:         0.05 %
    Noise Cutoff:         1000 counts
    Abundance Cutoff:     10 %
    MW Assign:            Curve fit
    MW Assign Cutoff:     40 %
    Envelope Cutoff:      50 %
```

Spectrum Deconvolution
Data File D:\EZXDATA\MARIARODRIGUEZ\07-24\080724-10-75OVER20_PEPTIDES-C3_200M-29589.D
Sample Name: MR_Brd4BD2_L387A_M442C

Deconvolution of Spectrum # 1 @ 15.394 min

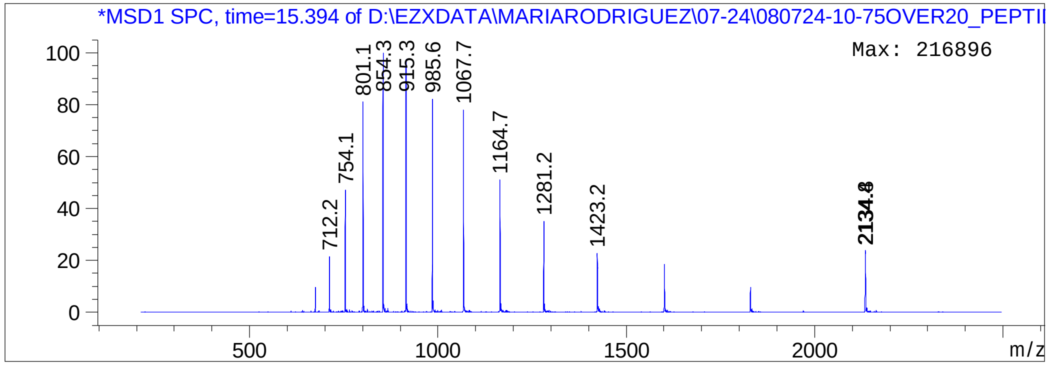

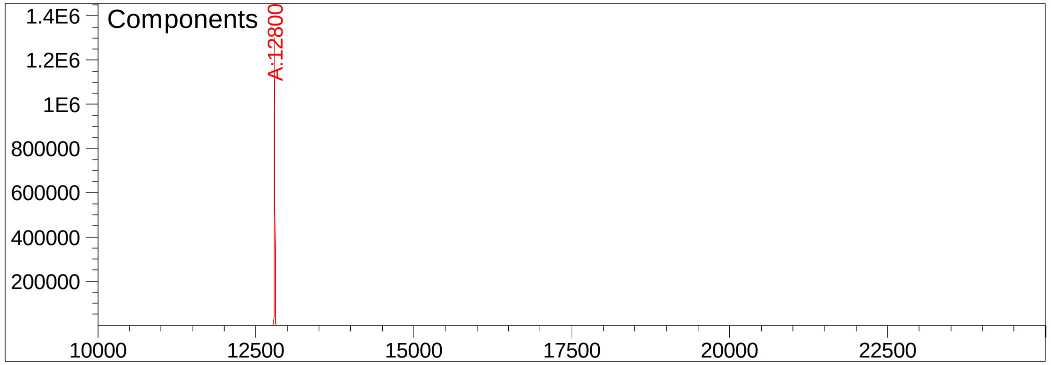

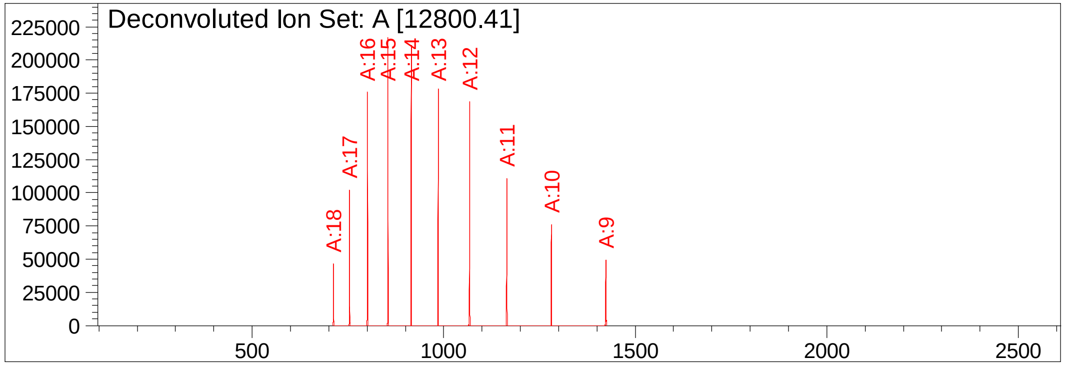

Component	Molecular Weight	Absolute Abundance	Relative Abundance
A	12800.41	1301086	100.00

*** End of Report ***

Data File D:\EZXDATA\MARIARODRIGUEZ\07-24\080724-10-75OVER20_PEPTIDES-C3_200M-29589.D
Sample Name: MR_Brd4BD2_L387A_M442C

===

Acq. Operator : Maria Rodriguez
Acq. Instrument : INSTRUMENT 1 Location : P2-A-06
Injection Date : 7/8/2024 7:43:43 PM Inj : 1
Inj Volume : 3.000 µl
Acq. Method : C:\CHEM32\1\METHODS\10-75OVER20_PEPTIDE
Last changed : 7/8/2024 7:42:55 PM by Maria Rodriguez
(modified after loading)
Analysis Method : C:\CHEM32\1\METHODS\10-75OVER20_PEPTIDES-C3.M
Last changed : 10/10/2024 10:16:46 AM by Kevin Haubrich
(modified after loading)
Sample Info : Easy-Access Method: '10-75over20-C3(200+m/Z)'

DAD1 C, Sig=214,4 Ref=off (D:\EZXDATA\MARIARODRIGUEZ\07-24\080724-10-75OVER20_PEPTIDES-C3_200M-29589.D)

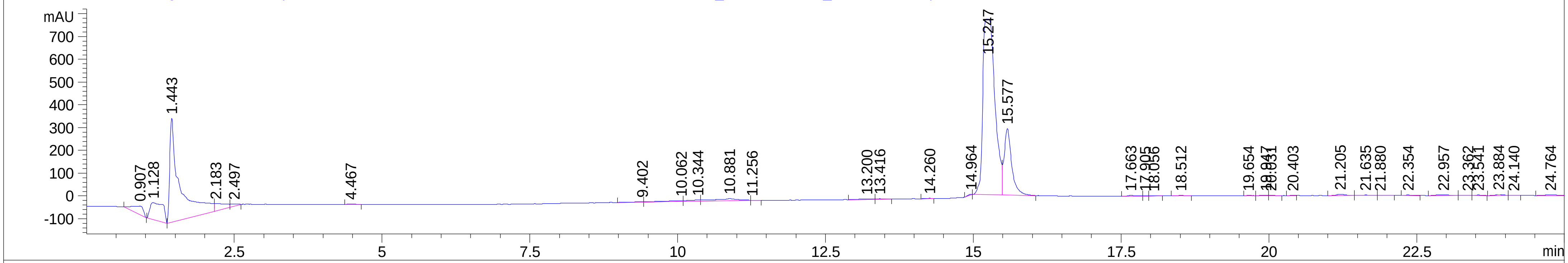

DAD1 G, Sig=280,4 Ref=off (D:\EZXDATA\MARIARODRIGUEZ\07-24\080724-10-75OVER20_PEPTIDES-C3_200M-29589.D)

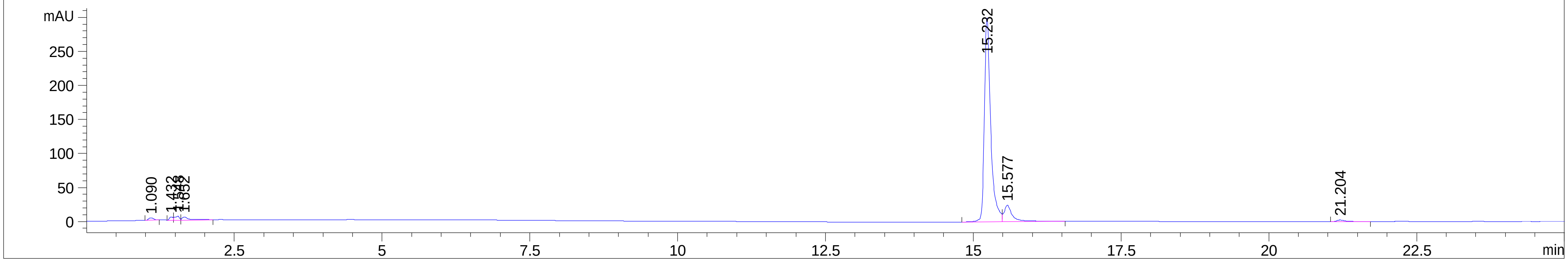

Data File D:\EZXDATA\MARIARODRIGUEZ\07-24\080724-10-75OVER20_PEPTIDES-C3_200M-29589.D
Sample Name: MR_Brd4BD2_L387A_M442C

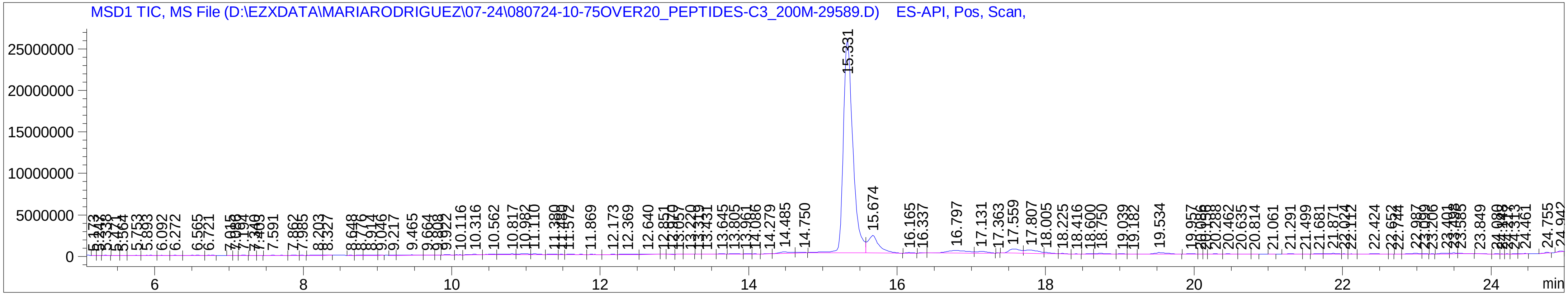

===
Area Percent Report
===

Sorted By : Signal
Multiplier : 1.0000
Dilution : 1.0000
Sample Amount: : 15.00000 [ng/ul] (not used in calc.)
Use Multiplier & Dilution Factor with ISTDs

Signal 1: DAD1 C, Sig=214,4 Ref=off

Peak #	RetTime [min]	Type	Width [min]	Area [mAU*s]	Height [mAU]	Area %
1	0.907	BB	0.1654	462.47467	38.16316	2.0954
2	1.128	BB	0.2170	1219.02869	75.25755	5.5233
3	1.443	BV	0.1590	5427.90283	455.60391	24.5931
4	2.183	VV	0.1414	361.13763	32.06916	1.6363
5	2.497	VB	0.1245	79.40338	8.69780	0.3598
6	4.467	BB	0.1110	11.08812	1.25643	0.0502
7	9.402	BV	0.1648	24.76394	1.89084	0.1122
8	10.062	VV	0.3112	98.96338	3.85947	0.4484
9	10.344	VV	0.1845	82.47421	5.64258	0.3737
10	10.881	VV	0.3652	278.55246	9.58192	1.2621

Peak #	RetTime [min]	Type	Width [min]	Area [mAU*s]	Height [mAU]	Area %
11	11.256	VB	0.0771	8.61661	1.45505	0.0390
12	13.200	BV	0.1764	39.41858	2.86756	0.1786
13	13.416	VB	0.1321	25.49668	2.39636	0.1155
14	14.260	BV	0.0963	10.80629	1.74461	0.0490
15	14.964	BB	0.1114	16.64950	2.27536	0.0754
16	15.247	BV	0.1951	1.09205e4	771.57135	49.4794
17	15.577	VB	0.1291	2574.58472	291.36703	11.6651
18	17.663	BV	0.1315	34.75997	3.57280	0.1575
19	17.905	VV	0.0611	5.88232	1.29122	0.0267
20	18.056	VB	0.1032	10.27396	1.25956	0.0465
21	18.512	VB	0.1325	18.22602	1.79437	0.0826
22	19.654	VV	0.1079	11.80325	1.35192	0.0535
23	19.947	VV	0.1155	14.63405	1.64907	0.0663
24	20.031	VB	0.0821	7.04688	1.17241	0.0319
25	20.403	BV	0.0798	6.24135	1.10742	0.0283
26	21.205	BB	0.1504	61.77280	5.79327	0.2799
27	21.635	BV	0.1678	26.25904	1.96674	0.1190
28	21.880	VB	0.1157	11.24270	1.19556	0.0509
29	22.354	VV	0.1572	24.56733	2.00168	0.1113
30	22.957	BB	0.1797	49.46235	3.44202	0.2241
31	23.362	BV	0.1128	13.94347	1.64661	0.0632
32	23.541	VB	0.1252	24.45579	2.51973	0.1108
33	23.884	BV	0.1301	45.72941	4.59675	0.2072
34	24.140	VB	0.0974	10.36670	1.29507	0.0470
35	24.764	BBA	0.1989	52.29575	3.26386	0.2369
Totals :				2.20708e4	1746.62017	

Signal 2: DAD1 G, Sig=280,4 Ref=off

Peak #	RetTime [min]	Type	Width [min]	Area [mAU*s]	Height [mAU]	Area %
1	1.090	BB	0.0950	21.53126	3.64087	0.8169
2	1.432	BV	0.0641	20.33381	4.92704	0.7715
3	1.548	VV	0.0766	32.88339	5.94184	1.2476
4	1.652	VB	0.1203	36.49252	4.51638	1.3845
5	15.232	BV	0.1118	2253.74805	299.43466	85.5057
6	15.577	VB	0.1412	246.20456	24.50475	9.3408
7	21.204	BB	0.1522	24.59469	2.38687	0.9331

Totals : 2635.78828 345.35240

Signal 3: MSD1 TIC, MS File

Peak #	RetTime [min]	Type	Width [min]	Area	Height	Area %
1	5.173	BV	0.0460	4.84220e4	1.85988e4	0.0161
2	5.247	VB	0.0335	3.63192e4	1.75738e4	0.0121
3	5.338	BB	0.0485	7.98216e4	2.74158e4	0.0266
4	5.471	BV	0.0617	5.58803e4	1.50841e4	0.0186
5	5.564	VB	0.0516	5.58617e4	1.73475e4	0.0186
6	5.753	BB	0.1148	1.53198e5	2.46079e4	0.0511
7	5.893	BV	0.0964	2.11223e5	2.83008e4	0.0704
8	6.092	VB	0.0864	1.46222e5	2.52192e4	0.0488
9	6.272	BB	0.0849	1.55973e5	2.95725e4	0.0520
10	6.565	BV	0.1791	2.48098e5	2.30837e4	0.0827
11	6.721	VB	0.0914	1.19457e5	2.17786e4	0.0398
12	7.015	BV	0.0493	7.41292e4	2.47366e4	0.0247
13	7.086	VB	0.0440	7.12277e4	2.77480e4	0.0238
14	7.194	BB	0.0648	1.52845e5	3.87256e4	0.0510
15	7.340	BV	0.0782	6.68999e4	1.42565e4	0.0223
16	7.403	VB	0.0403	3.84677e4	1.44994e4	0.0128
17	7.591	BB	0.0999	2.31582e5	2.98404e4	0.0772
18	7.862	BV	0.0744	1.18504e5	2.65610e4	0.0395
19	7.985	VB	0.0458	6.68740e4	2.43604e4	0.0223

Peak #	RetTime [min]	Type	Width [min]	Area	Height	Area %
20	8.203	BV	0.1321	2.02591e5	2.62684e4	0.0676
21	8.327	VB	0.0608	8.10035e4	2.02101e4	0.0270
22	8.648	BV	0.0520	5.82591e4	1.79194e4	0.0194
23	8.776	VV	0.0804	1.49642e5	2.83148e4	0.0499
24	8.914	VV	0.1356	3.42017e5	3.88179e4	0.1141
25	9.046	VB	0.0583	5.73717e4	1.64001e4	0.0191
26	9.217	BV	0.0566	5.10007e4	1.50118e4	0.0170
27	9.465	VV	0.1513	6.37071e5	5.23517e4	0.2125
28	9.664	VV	0.0909	1.90617e5	2.89407e4	0.0636
29	9.808	VB	0.0572	1.05786e5	3.25469e4	0.0353
30	9.922	BV	0.1053	3.68207e5	5.24551e4	0.1228
31	10.116	VB	0.0789	1.28249e5	2.29962e4	0.0428
32	10.316	BV	0.1370	5.40377e5	5.12865e4	0.1802
33	10.562	VB	0.1462	2.49652e5	3.10389e4	0.0833
34	10.817	BV	0.1168	5.73480e5	6.81912e4	0.1913
35	10.982	VV	0.1238	9.23480e5	1.07356e5	0.3080
36	11.110	VB	0.1216	6.33045e5	8.67894e4	0.2111
37	11.380	BV	0.1193	3.94394e5	5.62409e4	0.1315
38	11.480	VV	0.0606	1.34018e5	3.36034e4	0.0447
39	11.572	VV	0.1251	5.11436e5	5.15413e4	0.1706
40	11.869	VB	0.1003	3.04593e5	3.90957e4	0.1016
41	12.173	BV	0.1074	3.38485e5	4.45503e4	0.1129
42	12.369	VV	0.1221	5.41325e5	5.84232e4	0.1805
43	12.640	VV	0.1557	4.95029e5	4.07617e4	0.1651
44	12.851	VB	0.0481	4.97229e4	1.72336e4	0.0166
45	12.970	BB	0.0496	7.58490e4	2.20192e4	0.0253
46	13.057	BV	0.0628	8.09773e4	2.15064e4	0.0270
47	13.220	VB	0.0758	1.76011e5	3.32350e4	0.0587
48	13.319	BB	0.0354	4.00899e4	1.88991e4	0.0134
49	13.431	BV	0.0904	2.59039e5	3.95822e4	0.0864
50	13.645	VB	0.0870	3.06473e5	6.07162e4	0.1022
51	13.805	BV	0.1397	4.67107e5	5.17654e4	0.1558
52	13.961	VV	0.0757	2.70271e5	4.75108e4	0.0901
53	14.086	VB	0.0645	1.75868e5	4.06332e4	0.0587
54	14.279	BB	0.1059	1.81475e5	2.56625e4	0.0605
55	14.485	BV	0.1548	1.94841e6	1.79417e5	0.6498

Data File D:\EZXDATA\MARIARODRIGUEZ\07-24\080724-10-75OVER20_PEPTIDES-C3_200M-29589.D
Sample Name: MR_Brd4BD2_L387A_M442C

Peak #	RetTime [min]	Type	Width [min]	Area	Height	Area %
56	14.750	VB	0.1218	5.13135e5	5.55343e4	0.1711
57	15.331	BV	0.1362	2.27929e8	2.57184e7	76.0142
58	15.674	VB	0.1450	2.21504e7	2.12600e6	7.3871
59	16.165	BB	0.1134	5.99939e5	8.20025e4	0.2001
60	16.337	BB	0.0653	1.88159e5	4.28437e4	0.0628
61	16.797	BV	0.2367	6.09066e6	3.26043e5	2.0312
62	17.131	VB	0.1835	2.20354e6	2.00180e5	0.7349
63	17.363	BB	0.0372	3.59455e4	1.50591e4	0.0120
64	17.559	BV	0.1687	5.36873e6	4.94983e5	1.7905
65	17.807	VV	0.1607	5.01729e6	3.99046e5	1.6733
66	18.005	VB	0.1055	8.79477e5	1.38945e5	0.2933
67	18.225	BB	0.0654	2.32549e5	5.28509e4	0.0776
68	18.416	BB	0.0824	1.76138e5	3.78568e4	0.0587
69	18.600	BV	0.0995	5.24879e5	8.26724e4	0.1750
70	18.750	VV	0.1625	1.32796e6	1.11420e5	0.4429
71	19.039	VV	0.0898	4.11252e5	6.33287e4	0.1372
72	19.182	VB	0.0802	1.80871e5	3.19184e4	0.0603
73	19.534	BB	0.1664	2.78668e6	2.13359e5	0.9294
74	19.957	BV	0.1082	5.54420e5	7.62408e4	0.1849
75	20.086	VB	0.0432	3.25521e4	1.28003e4	0.0109
76	20.156	BV	0.0382	4.74791e4	2.07274e4	0.0158
77	20.288	VV	0.0959	3.38110e5	5.11416e4	0.1128
78	20.462	VB	0.0888	3.70207e5	5.77902e4	0.1235
79	20.635	BV	0.0830	1.37379e5	2.17667e4	0.0458
80	20.814	VB	0.0514	6.70905e4	2.17739e4	0.0224
81	21.061	BB	0.0547	7.33431e4	2.10195e4	0.0245
82	21.291	BB	0.1097	5.67480e5	6.92349e4	0.1893
83	21.499	BB	0.0480	6.53456e4	1.97287e4	0.0218
84	21.681	BV	0.1023	3.25667e5	4.81589e4	0.1086
85	21.871	VV	0.1432	8.77517e5	8.22357e4	0.2927
86	22.024	VB	0.0490	9.44610e4	3.21480e4	0.0315
87	22.112	BB	0.0567	1.21974e5	3.00653e4	0.0407
88	22.424	BB	0.1316	8.21757e5	7.84037e4	0.2741
89	22.652	BB	0.0375	3.75650e4	1.66971e4	0.0125
90	22.744	BV	0.0608	8.49820e4	2.35649e4	0.0283
91	22.987	VV	0.1248	8.19564e5	9.01035e4	0.2733

Peak #	RetTime [min]	Type	Width [min]	Area	Height	Area %
92	23.099	VB	0.0665	2.32856e5	5.68055e4	0.0777
93	23.206	BB	0.0360	6.49247e4	2.84331e4	0.0217
94	23.401	BV	0.1251	7.35303e5	8.84635e4	0.2452
95	23.498	VV	0.0796	6.50974e5	1.24818e5	0.2171
96	23.585	VB	0.0892	4.65713e5	8.70393e4	0.1553
97	23.849	BB	0.0980	2.70058e5	3.55375e4	0.0901
98	24.080	BV	0.0621	1.26512e5	3.50211e4	0.0422
99	24.148	VV	0.0437	5.66482e4	2.16026e4	0.0189
100	24.217	VB	0.0482	5.92997e4	2.05104e4	0.0198
101	24.313	BV	0.0482	9.38912e4	2.82202e4	0.0313
102	24.461	VB	0.0671	1.89150e5	4.56421e4	0.0631
103	24.755	BB	0.0614	2.92938e5	8.00465e4	0.0977
104	24.942	BBA	0.0676	3.85308e5	9.18979e4	0.1285
Totals :				2.99850e8	3.37387e7	

*** End of Report ***

```
    Easy-Access Method: '10-75over20-C3(200+m/Z)'
    =====================================================================
    Acq. Operator   : Maria Rodriguez
    Acq. Instrument : INSTRUMENT 1                     Location : P2-A-05
    Injection Date  : 7/8/2024 7:13:54 PM                   Inj :   1
                                                   Inj Volume : 3.000 µl
    Acq. Method     : C:\CHEM32\1\METHODS\10-75OVER20_PEPTIDE
    Last changed    : 7/8/2024 7:13:04 PM by Maria Rodriguez
                      (modified after loading)
    Analysis Method : C:\CHEM32\1\METHODS\10-75OVER20_PEPTIDES-C3.M
    Last changed    : 10/10/2024 10:16:18 AM by Kevin Haubrich
                      (modified after loading)
    Sample Info     : Easy-Access Method: '10-75over20-C3(200+m/Z)'


    =====================================================================
                          Deconvolution Parameters
    =====================================================================

    Adduct Ion(Positive): +H, 1.0079 Da
    Adduct Ion(Negative):  , 0.0000 Da
    Low MW:               10000
    DeconvStartChgMaximum Charge:       50
    Minimum Peaks in Set: 3
    Retain Residual:      No
    Ion PWHH:             0.6 Da
    MW Agreement:         0.05 %
    Noise Cutoff:         1000 counts
    Abundance Cutoff:     10 %
    MW Assign:            Curve fit
    MW Assign Cutoff:     40 %
    Envelope Cutoff:      50 %
```

Deconvolution of Spectrum # 1 @ 15.550 min

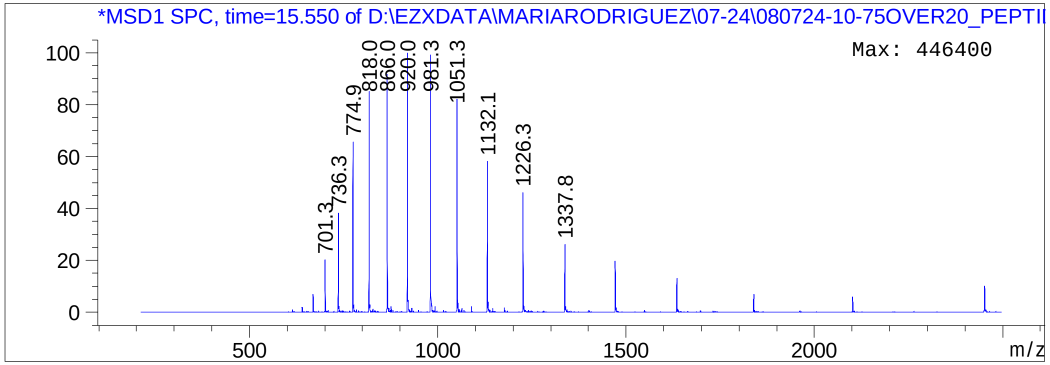

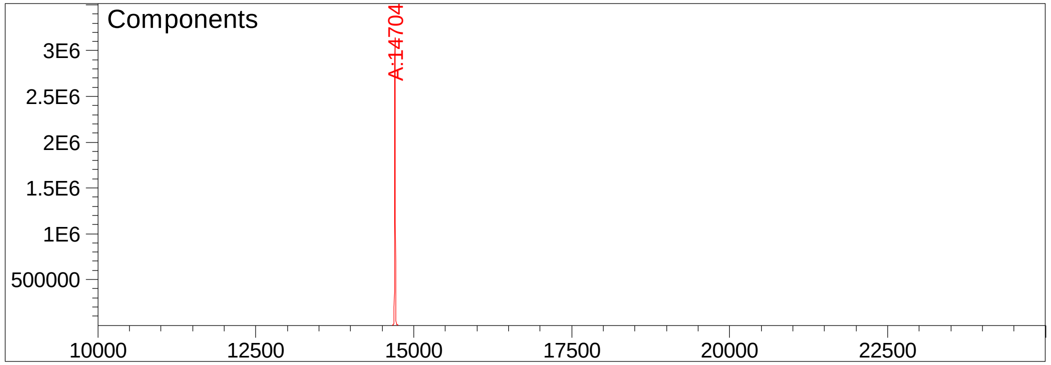

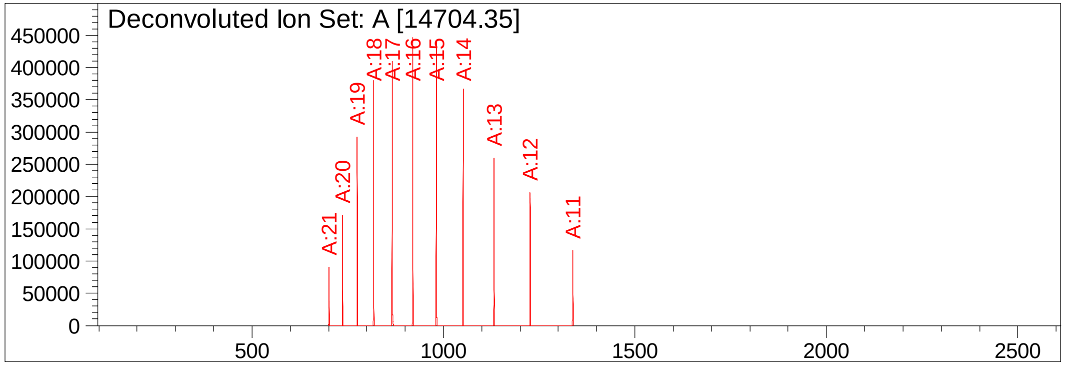

Component	Molecular Weight	Absolute Abundance	Relative Abundance
A	14704.35	3140243	100.00

*** End of Report ***

Spectrum Deconvolution
Data File D:\EZXDATA\MARIARODRIGUEZ\07-24\080724-10-75OVER20_PEPTIDES-C3_200M-29585.D
Sample Name: MR_Brd4BD2_L387A

```
Easy-Access Method: '10-75over20-C3(200+m/Z)'
=====================================================================
Acq. Operator   : Maria Rodriguez
Acq. Instrument : INSTRUMENT 1                    Location : P2-A-02
Injection Date  : 7/8/2024 5:44:11 PM                  Inj :   1
                                              Inj Volume : 3.000 µl
Acq. Method     : C:\CHEM32\1\METHODS\10-75OVER20_PEPTIDE
Last changed    : 7/8/2024 5:43:23 PM by Maria Rodriguez
                  (modified after loading)
Analysis Method : C:\CHEM32\1\METHODS\10-75OVER20_PEPTIDES-C3.M
Last changed    : 10/10/2024 10:15:04 AM by Kevin Haubrich
                  (modified after loading)
Sample Info     : Easy-Access Method: '10-75over20-C3(200+m/Z)'


=====================================================================
                      Deconvolution Parameters
=====================================================================

Adduct Ion(Positive): +H, 1.0079 Da
Adduct Ion(Negative):  , 0.0000 Da
Low MW:               10000
DeconvStartChgMaximum Charge:       50
Minimum Peaks in Set: 3
Retain Residual:      No
Ion PWHH:             0.6 Da
MW Agreement:         0.05 %
Noise Cutoff:         1000 counts
Abundance Cutoff:     10 %
MW Assign:            Curve fit
MW Assign Cutoff:     40 %
Envelope Cutoff:      50 %
```

Spectrum Deconvolution
Data File D:\EZXDATA\MARIARODRIGUEZ\07-24\080724-10-75OVER20_PEPTIDES-C3_200M-29585.D
Sample Name: MR_Brd4BD2_L387A

Deconvolution of Spectrum # 1 @ 15.207 min

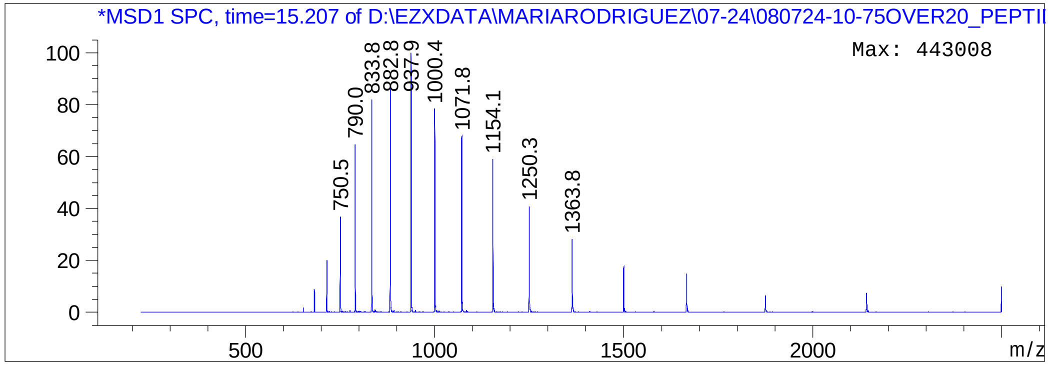

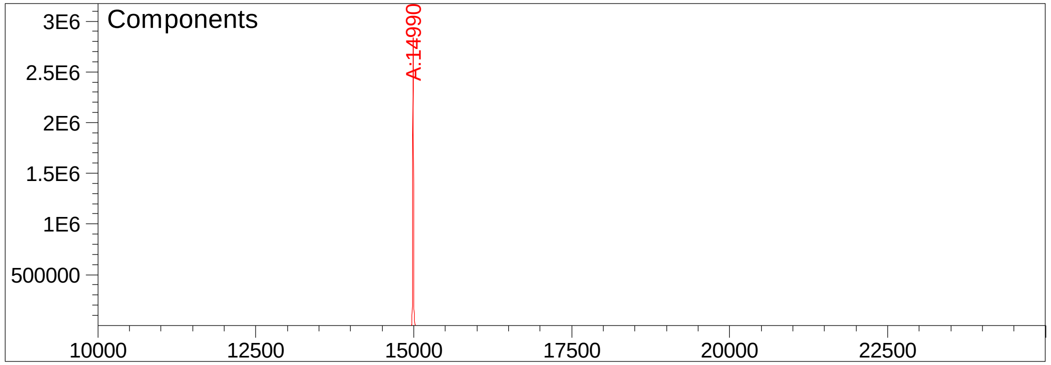

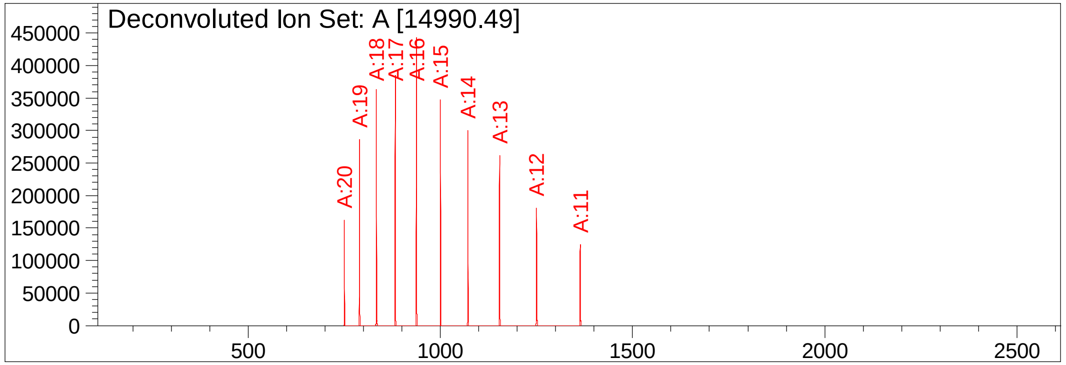

Component	Molecular Weight	Absolute Abundance	Relative Abundance
A	14990.49	2836756	100.00

*** End of Report ***

Data File D:\EZXDATA\MARIARODRIGUEZ\07-24\080724-10-75OVER20_PEPTIDES-C3_200M-29585.D
Sample Name: MR_Brd4BD2_L387A

===

Acq. Operator : Maria Rodriguez
Acq. Instrument : INSTRUMENT 1 Location : P2-A-02
Injection Date : 7/8/2024 5:44:11 PM Inj : 1
Inj Volume : 3.000 µl
Acq. Method : C:\CHEM32\1\METHODS\10-75OVER20_PEPTIDE
Last changed : 7/8/2024 5:43:23 PM by Maria Rodriguez
(modified after loading)
Analysis Method : C:\CHEM32\1\METHODS\10-75OVER20_PEPTIDES-C3.M
Last changed : 10/10/2024 10:16:46 AM by Kevin Haubrich
(modified after loading)
Sample Info : Easy-Access Method: '10-75over20-C3(200+m/Z)'

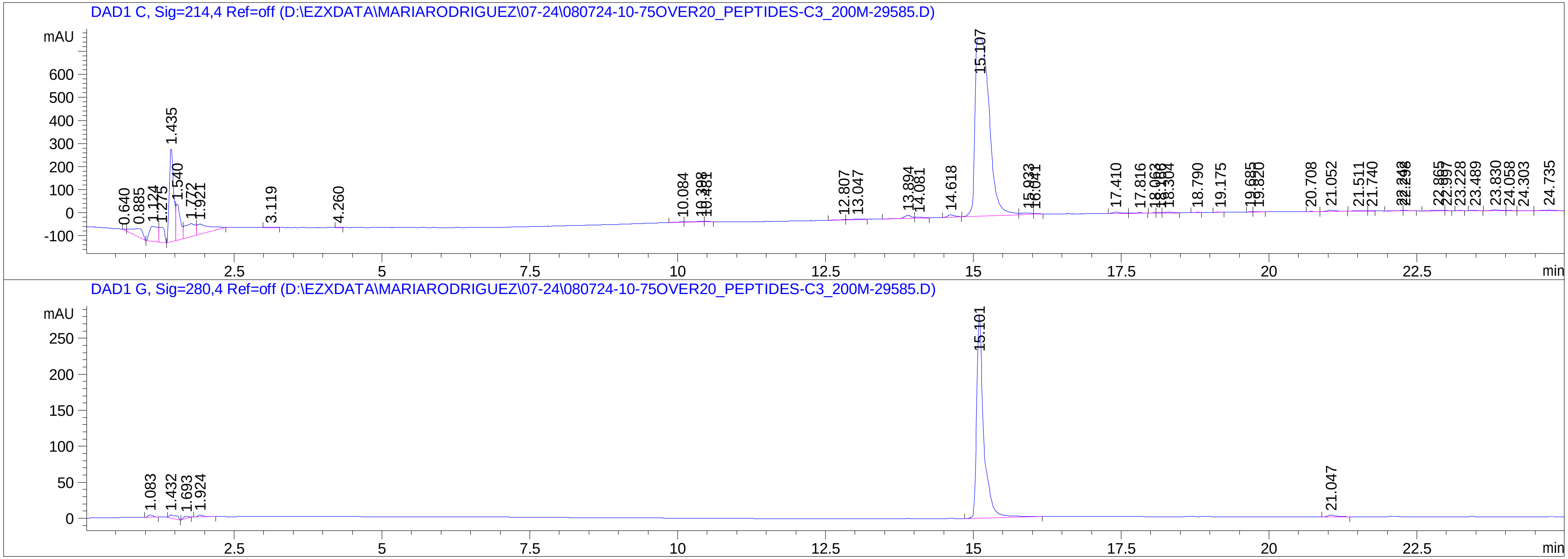

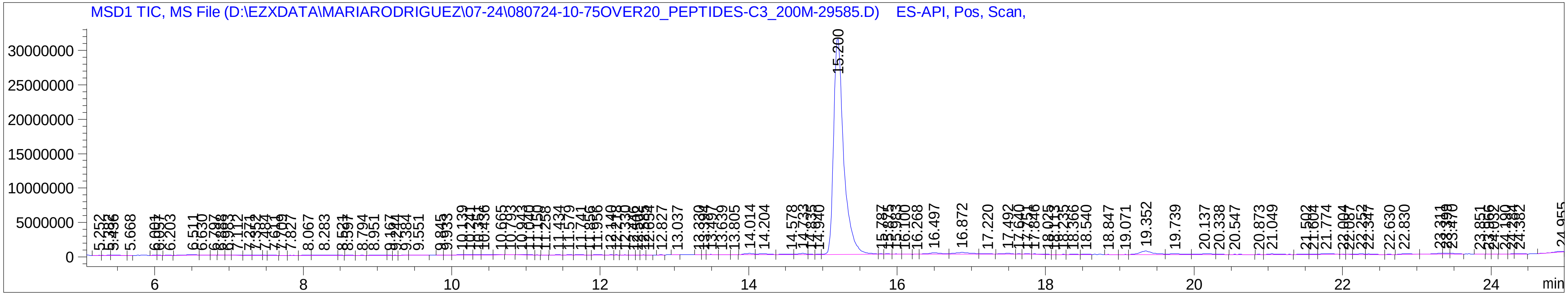

==

Area Percent Report

==

Sorted By : Signal
Multiplier : 1.0000
Dilution : 1.0000
Sample Amount: : 15.00000 [ng/ul] (not used in calc.)
Use Multiplier & Dilution Factor with ISTDs

Signal 1: DAD1 C, Sig=214,4 Ref=off

Peak #	RetTime [min]	Type	Width [min]	Area [mAU*s]	Height [mAU]	Area %
1	0.640	BV	0.0717	20.64107	4.03469	0.1050
2	0.885	VB	0.1853	457.90204	35.77332	2.3287
3	1.124	BV	0.1434	637.97217	65.62957	3.2445
4	1.275	VB	0.0977	413.07428	65.36630	2.1007
5	1.435	BV	0.0773	2049.44312	402.97418	10.4226
6	1.540	VV	0.0767	808.18872	155.36247	4.1101
7	1.772	VV	0.1688	708.94598	55.57455	3.6054
8	1.921	VB	0.1996	660.60529	43.88783	3.3596
9	3.119	VV	0.1350	11.81990	1.10358	0.0601
10	4.260	VV	0.0771	5.90996	1.05911	0.0301

Peak #	RetTime [min]	Type	Width [min]	Area [mAU*s]	Height [mAU]	Area %
11	10.084	BV	0.1100	14.46721	1.68734	0.0736
12	10.398	VV	0.1839	45.64279	2.99296	0.2321
13	10.481	VV	0.0872	15.93128	2.34728	0.0810
14	12.807	BV	0.1502	21.62688	1.85254	0.1100
15	13.047	VV	0.1667	45.95338	3.60635	0.2337
16	13.894	BV	0.1377	122.37447	13.25387	0.6223
17	14.081	VB	0.1193	42.11231	5.05015	0.2142
18	14.618	BB	0.1258	81.04530	10.08114	0.4122
19	15.107	BV	0.2466	1.28371e4	769.44727	65.2842
20	15.933	VV	0.1769	76.59408	5.42053	0.3895
21	16.041	VB	0.0850	19.06769	3.32017	0.0970
22	17.410	VV	0.1515	59.15967	5.58828	0.3009
23	17.816	VB	0.1563	26.18308	2.27521	0.1332
24	18.063	BV	0.0756	8.62496	1.53447	0.0439
25	18.166	VV	0.0597	7.58397	1.64756	0.0386
26	18.304	VB	0.1396	38.62696	4.11072	0.1964
27	18.790	VV	0.1051	8.56256	1.07252	0.0435
28	19.175	BV	0.0729	6.79854	1.22293	0.0346
29	19.685	BV	0.0635	5.49743	1.29534	0.0280
30	19.820	VV	0.1120	13.46236	1.53979	0.0685
31	20.708	VB	0.1154	11.31168	1.32735	0.0575
32	21.052	BB	0.1591	64.30230	5.99612	0.3270
33	21.511	BV	0.1635	38.35818	2.87783	0.1951
34	21.740	VV	0.0810	10.03179	1.52067	0.0510
35	22.242	BV	0.1045	18.91514	2.28604	0.0962
36	22.298	VB	0.0991	18.19696	2.33123	0.0925
37	22.865	BV	0.1939	48.92530	3.10336	0.2488
38	22.997	VB	0.0620	8.19249	1.99120	0.0417
39	23.228	BB	0.0745	6.11664	1.07348	0.0311
40	23.489	VB	0.1055	17.34661	2.11887	0.0882
41	23.830	BV	0.1696	55.59853	4.45414	0.2828
42	24.058	VV	0.1157	17.43042	2.00010	0.0886
43	24.303	VB	0.1397	16.12693	1.47315	0.0820
44	24.735	BBA	0.1986	61.66739	3.85580	0.3136
Totals :				1.96635e4	1706.52136	

Signal 2: DAD1 G, Sig=280,4 Ref=off

Peak #	RetTime [min]	Type	Width [min]	Area [mAU*s]	Height [mAU]	Area %
1	1.083	BB	0.0927	18.40048	3.21901	0.7408
2	1.432	BB	0.1384	43.38436	4.13407	1.7466
3	1.693	BB	0.1009	18.26765	2.84512	0.7354
4	1.924	BB	0.0948	14.53656	2.26658	0.5852
5	15.101	BB	0.1221	2367.21729	281.39673	95.2996
6	21.047	BB	0.1404	22.16773	2.34269	0.8924

Totals : 2483.97406 296.20420

Signal 3: MSD1 TIC, MS File

Peak #	RetTime [min]	Type	Width [min]	Area	Height	Area %
1	5.252	BV	0.0492	1.03920e5	3.04373e4	0.0298
2	5.382	VV	0.0759	2.21346e5	4.10726e4	0.0636
3	5.436	VB	0.1002	4.47233e5	5.74331e4	0.1284
4	5.668	BB	0.0345	5.72363e4	2.65722e4	0.0164
5	6.001	BV	0.0494	1.35750e5	3.95746e4	0.0390
6	6.057	VB	0.0459	1.25854e5	4.60884e4	0.0361
7	6.203	BB	0.0737	1.85155e5	4.73469e4	0.0532
8	6.511	BV	0.1616	1.03613e6	9.48496e4	0.2976
9	6.630	VB	0.0834	2.38884e5	4.77254e4	0.0686
10	6.797	BB	0.0513	7.86580e4	2.81720e4	0.0226
11	6.888	BV	0.0614	1.00673e5	2.47967e4	0.0289
12	6.983	VV	0.0465	1.05942e5	3.79414e4	0.0304
13	7.112	VB	0.0905	2.00948e5	3.75736e4	0.0577
14	7.271	BV	0.0698	2.64636e5	6.04243e4	0.0760
15	7.352	VV	0.0609	2.31921e5	5.25061e4	0.0666
16	7.484	VB	0.0378	4.58568e4	2.02027e4	0.0132

Peak #	RetTime [min]	Type	Width [min]	Area	Height	Area %
17	7.611	BB	0.0829	3.34118e5	7.11019e4	0.0960
18	7.709	BV	0.0691	1.26491e5	3.05294e4	0.0363
19	7.827	VV	0.0757	1.93301e5	3.39849e4	0.0555
20	8.067	VV	0.1156	3.36560e5	4.85263e4	0.0967
21	8.283	VV	0.1677	6.20946e5	4.57525e4	0.1783
22	8.521	VB	0.0504	7.36272e4	2.43281e4	0.0211
23	8.597	BB	0.0781	1.74095e5	3.16823e4	0.0500
24	8.794	BB	0.0572	1.07210e5	2.89590e4	0.0308
25	8.951	BV	0.1175	6.29329e5	6.79225e4	0.1807
26	9.167	VB	0.0368	6.43645e4	2.91285e4	0.0185
27	9.244	BB	0.0337	2.81120e4	1.34692e4	8.074e-3
28	9.384	BV	0.0781	1.63874e5	3.53327e4	0.0471
29	9.551	VB	0.1107	4.10050e5	4.72151e4	0.1178
30	9.845	BV	0.0641	1.19475e5	3.46869e4	0.0343
31	9.933	VB	0.0487	9.11769e4	2.70243e4	0.0262
32	10.139	BV	0.0794	1.78783e5	3.13957e4	0.0513
33	10.241	VV	0.0868	2.75654e5	5.07620e4	0.0792
34	10.351	VV	0.0822	3.54690e5	7.03906e4	0.1019
35	10.436	VV	0.0860	3.75277e5	6.08379e4	0.1078
36	10.665	VB	0.0999	2.51069e5	3.68682e4	0.0721
37	10.793	BB	0.0602	8.68563e4	2.19313e4	0.0249
38	10.943	BV	0.0861	1.99297e5	3.22581e4	0.0572
39	11.040	VB	0.0735	1.15955e5	2.62776e4	0.0333
40	11.150	BB	0.0505	5.85306e4	2.18383e4	0.0168
41	11.258	BB	0.0770	1.05631e5	2.60520e4	0.0303
42	11.434	BB	0.0975	2.73626e5	4.30750e4	0.0786
43	11.579	BV	0.0694	2.43255e5	5.12636e4	0.0699
44	11.741	VB	0.0928	3.28635e5	5.17517e4	0.0944
45	11.856	BV	0.0385	4.47827e4	1.93819e4	0.0129
46	11.956	VV	0.0774	3.54439e5	6.52273e4	0.1018
47	12.140	VV	0.0846	1.62652e5	3.41678e4	0.0467
48	12.218	VB	0.0490	8.48291e4	2.88819e4	0.0244
49	12.330	BB	0.0670	1.11812e5	2.99442e4	0.0321
50	12.446	BV	0.0624	1.01829e5	2.72144e4	0.0292
51	12.502	VB	0.0373	6.61715e4	2.75917e4	0.0190
52	12.585	BV	0.0404	7.37960e4	2.77118e4	0.0212

Peak #	RetTime [min]	Type	Width [min]	Area	Height	Area %
53	12.654	VB	0.0581	7.59277e4	2.17751e4	0.0218
54	12.827	BB	0.0509	8.28330e4	2.62199e4	0.0238
55	13.037	BB	0.0736	9.63692e4	2.23949e4	0.0277
56	13.330	BV	0.0596	7.57041e4	2.12255e4	0.0217
57	13.394	VB	0.0435	5.10429e4	2.03021e4	0.0147
58	13.497	BV	0.0999	2.92231e5	4.45660e4	0.0839
59	13.639	VV	0.1031	3.74785e5	4.95836e4	0.1076
60	13.805	VB	0.0686	1.89715e5	4.89863e4	0.0545
61	14.014	BV	0.1184	1.75802e6	1.96442e5	0.5049
62	14.204	VB	0.1311	1.22036e6	1.26730e5	0.3505
63	14.578	BV	0.1249	5.21201e5	6.27800e4	0.1497
64	14.733	VV	0.0987	1.08611e6	1.58357e5	0.3119
65	14.835	VB	0.0615	2.62318e5	7.15329e4	0.0753
66	14.940	BV	0.0463	1.60440e5	5.07357e4	0.0461
67	15.200	VV	0.1428	2.96638e8	3.14643e7	85.1947
68	15.787	VV	0.0582	1.62314e5	4.64505e4	0.0466
69	15.875	VB	0.0742	2.21466e5	5.09066e4	0.0636
70	15.983	BV	0.0606	1.52747e5	3.82864e4	0.0439
71	16.100	VB	0.0935	1.39970e5	2.49568e4	0.0402
72	16.268	BB	0.0603	6.96593e4	1.93783e4	0.0200
73	16.497	BV	0.1635	2.74267e6	2.20941e5	0.7877
74	16.872	VV	0.1925	3.76676e6	2.52884e5	1.0818
75	17.220	VB	0.1162	7.34433e5	8.38910e4	0.2109
76	17.492	BB	0.1056	8.45535e5	1.20081e5	0.2428
77	17.640	BV	0.0515	1.66497e5	5.18119e4	0.0478
78	17.751	VV	0.0939	3.72956e5	6.17413e4	0.1071
79	17.846	VV	0.1153	4.60227e5	6.65300e4	0.1322
80	18.025	VB	0.0476	1.21667e5	4.23214e4	0.0349
81	18.113	BV	0.0344	3.74456e4	1.81214e4	0.0108
82	18.235	VV	0.0867	1.32720e5	2.87005e4	0.0381
83	18.366	VV	0.0831	3.29486e5	5.97319e4	0.0946
84	18.540	VB	0.0734	1.57381e5	2.86839e4	0.0452
85	18.847	BB	0.0746	1.50952e5	2.90691e4	0.0434
86	19.071	BV	0.0893	2.84282e5	5.41401e4	0.0816
87	19.352	VV	0.1803	7.01985e6	5.37931e5	2.0161
88	19.739	VV	0.1860	2.34330e6	1.68135e5	0.6730

Peak #	RetTime [min]	Type	Width [min]	Area	Height	Area %
89	20.137	VV	0.1936	1.96088e6	1.24201e5	0.5632
90	20.338	VB	0.0977	5.27519e5	7.79485e4	0.1515
91	20.547	BB	0.1464	5.18004e5	4.63649e4	0.1488
92	20.873	BV	0.0746	2.33043e5	4.86858e4	0.0669
93	21.049	VB	0.1900	1.25761e6	1.10306e5	0.3612
94	21.502	BV	0.0950	5.14292e5	7.40867e4	0.1477
95	21.604	VV	0.0753	3.56645e5	6.31440e4	0.1024
96	21.774	VB	0.1383	1.42894e6	1.39414e5	0.4104
97	22.004	BB	0.0350	6.10176e4	2.77210e4	0.0175
98	22.087	BV	0.0487	6.83390e4	2.02918e4	0.0196
99	22.252	VV	0.0798	6.22800e5	1.10439e5	0.1789
100	22.347	VB	0.1076	7.05895e5	9.00760e4	0.2027
101	22.630	BB	0.0696	2.27839e5	5.22761e4	0.0654
102	22.830	BB	0.1368	1.15186e6	1.09466e5	0.3308
103	23.311	BV	0.1188	1.04931e6	1.16912e5	0.3014
104	23.399	VV	0.0758	6.79544e5	1.28441e5	0.1952
105	23.470	VB	0.0861	6.74933e5	1.02625e5	0.1938
106	23.851	BB	0.0796	1.53821e5	3.22079e4	0.0442
107	23.966	BV	0.0508	1.18204e5	3.87982e4	0.0339
108	24.032	VB	0.0485	1.05939e5	3.15757e4	0.0304
109	24.190	BB	0.0466	6.70571e4	2.10257e4	0.0193
110	24.284	BV	0.0406	1.28056e5	4.78172e4	0.0368
111	24.382	VB	0.0871	4.18748e5	7.14422e4	0.1203
112	24.955	BBA	0.2322	9.60052e5	6.89177e4	0.2757

Totals : 3.48188e8 3.81920e7

==

*** End of Report ***

Spectrum Deconvolution
Data File D:\EZXDATA\MARIARODRIGUEZ\07-24\090724-10-75OVER20_PEPTIDES-C3_200M-29595.D
Sample Name: Brd4BD2_WT

```
Easy-Access Method: '10-75over20-C3(200+m/Z)'
=====================================================================
Acq. Operator   : Maria Rodriguez
Acq. Instrument : INSTRUMENT 1                     Location : P2-B-03
Injection Date  : 7/9/2024 6:48:49 PM                   Inj :   1
                                                 Inj Volume : 2.000 µl
Acq. Method     : C:\CHEM32\1\METHODS\10-75OVER20_PEPTIDE
Last changed    : 7/9/2024 6:48:00 PM by Maria Rodriguez
                  (modified after loading)
Analysis Method : C:\CHEM32\1\METHODS\10-75OVER20_PEPTIDES-C3.M
Last changed    : 10/10/2024 10:31:15 AM by Kevin Haubrich
                  (modified after loading)
Sample Info     : Easy-Access Method: '10-75over20-C3(200+m/Z)'


=====================================================================
                      Deconvolution Parameters
=====================================================================

Adduct Ion(Positive): +H, 1.0079 Da
Adduct Ion(Negative):  , 0.0000 Da
Low MW:               10000
DeconvStartChgMaximum Charge:       50
Minimum Peaks in Set: 3
Retain Residual:      No
Ion PWHH:             0.6 Da
MW Agreement:         0.05 %
Noise Cutoff:         1000 counts
Abundance Cutoff:     10 %
MW Assign:            Curve fit
MW Assign Cutoff:     40 %
Envelope Cutoff:      50 %
```

Spectrum Deconvolution
Data File D:\EZXDATA\MARIARODRIGUEZ\07-24\090724-10-75OVER20_PEPTIDES-C3_200M-29595.D
Sample Name: Brd4BD2_WT

Deconvolution of Spectrum # 1 @ 15.332 - 15.987 min

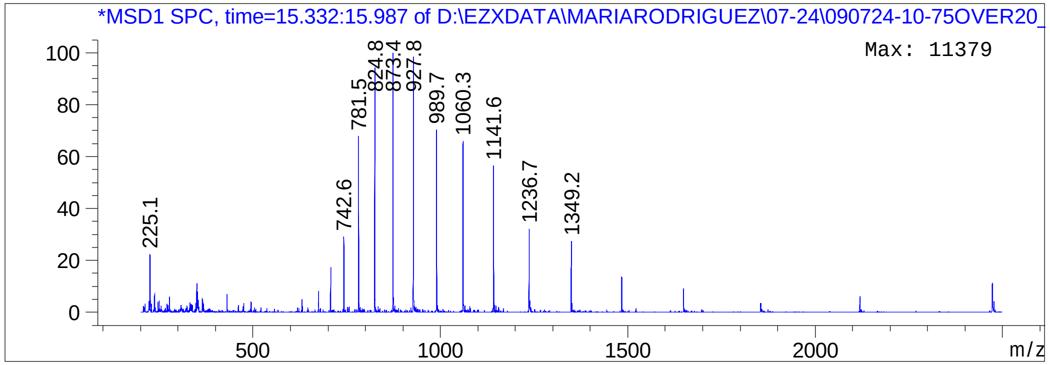

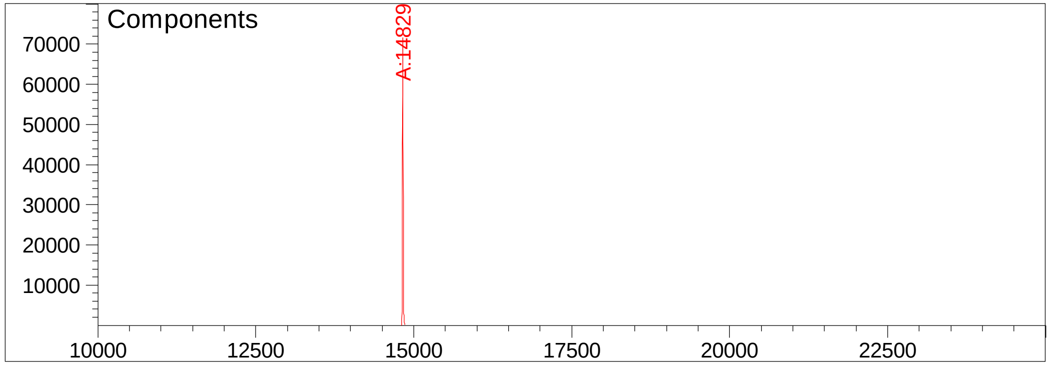

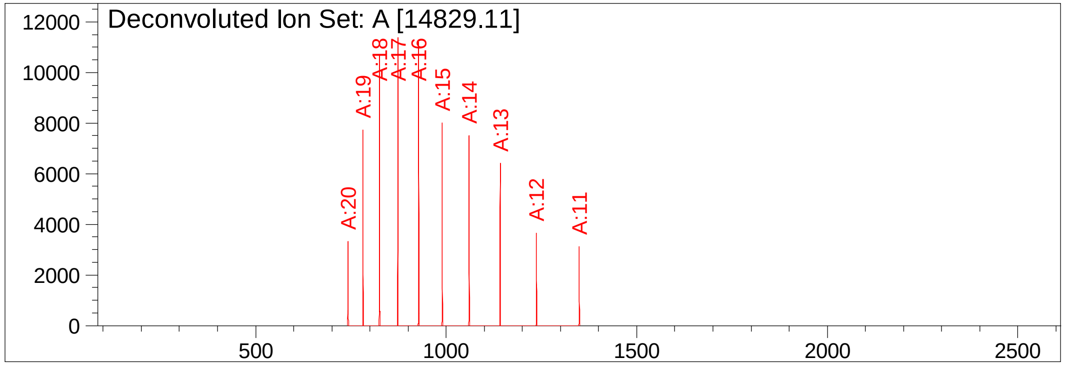

Component	Molecular Weight	Absolute Abundance	Relative Abundance
A	14829.11	71600	100.00

*** End of Report ***

Data File D:\EZXDATA\MARIARODRIGUEZ\07-24\090724-10-75OVER20_PEPTIDES-C3_200M-29595.D
Sample Name: Brd4BD2_WT

===

```
Acq. Operator   : Maria Rodriguez
Acq. Instrument : INSTRUMENT 1                    Location : P2-B-03
Injection Date  : 7/9/2024 6:48:49 PM                  Inj :    1
                                                Inj Volume : 2.000 µl
Acq. Method     : C:\CHEM32\1\METHODS\10-75OVER20_PEPTIDE
Last changed    : 7/9/2024 6:48:00 PM by Maria Rodriguez
                  (modified after loading)
Analysis Method : C:\CHEM32\1\METHODS\10-75OVER20_PEPTIDES-C3.M
Last changed    : 10/10/2024 10:31:15 AM by Kevin Haubrich
                  (modified after loading)
Sample Info     : Easy-Access Method: '10-75over20-C3(200+m/Z)'
```

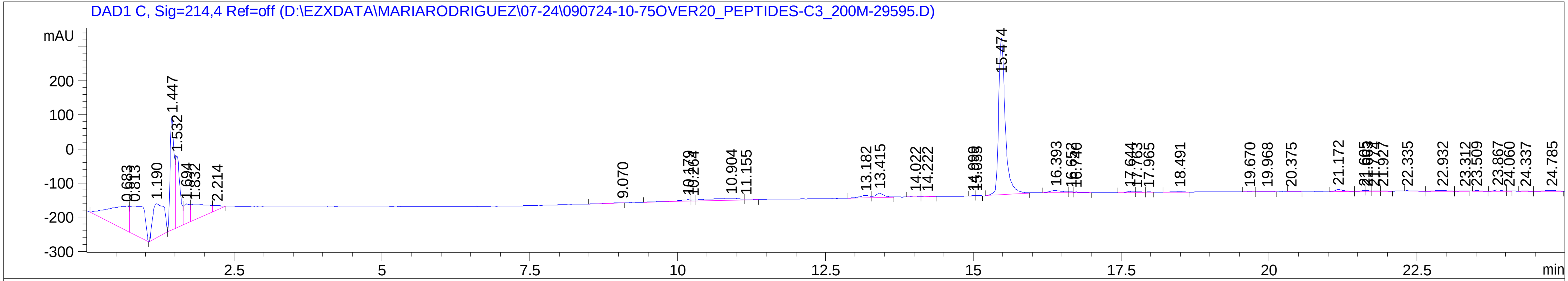

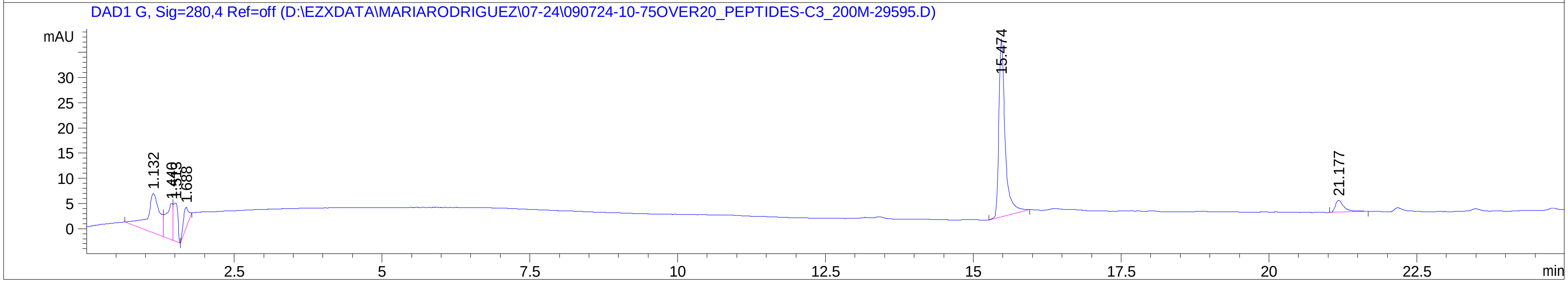

Data File D:\EZXDATA\MARIARODRIGUEZ\07-24\090724-10-75OVER20_PEPTIDES-C3_200M-29595.D
Sample Name: Brd4BD2_WT

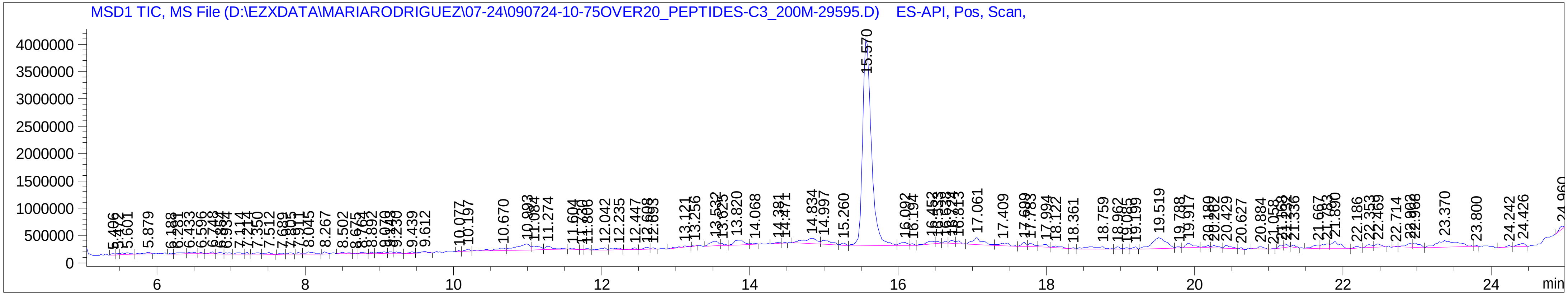

===

Area Percent Report

===

Sorted By : Signal
Multiplier : 1.0000
Dilution : 1.0000
Sample Amount: : 15.00000 [ng/ul] (not used in calc.)
Use Multiplier & Dilution Factor with ISTDs

Signal 1: DAD1 C, Sig=214,4 Ref=off

Peak #	RetTime [min]	Type	Width [min]	Area [mAU*s]	Height [mAU]	Area %
1	0.683	BV	0.2675	1554.73083	71.54913	11.9114
2	0.813	VB	0.2183	1458.30054	83.27843	11.1726
3	1.190	BB	0.1867	1279.75452	99.05791	9.8047
4	1.447	BV	0.0710	1560.35986	331.10132	11.9545
5	1.532	VV	0.0785	1123.52893	209.69371	8.6078
6	1.694	VV	0.0986	404.32394	57.09786	3.0977
7	1.832	VV	0.2179	803.18951	46.41286	6.1535
8	2.214	VB	0.1271	128.53290	13.25556	0.9847
9	9.070	BV	0.1682	14.44200	1.06518	0.1106
10	10.179	BV	0.2451	81.85651	4.24203	0.6271

Peak #	RetTime [min]	Type	Width [min]	Area [mAU*s]	Height [mAU]	Area %
11	10.264	VV	0.0512	14.47983	3.73555	0.1109
12	10.904	VV	0.4640	233.98143	6.03908	1.7926
13	11.155	VB	0.1036	17.34912	2.16287	0.1329
14	13.182	BV	0.1483	83.22591	7.23232	0.6376
15	13.415	VV	0.1449	121.50725	12.12260	0.9309
16	14.022	BV	0.1128	20.50737	2.37312	0.1571
17	14.222	VB	0.1117	14.86924	1.74074	0.1139
18	14.999	BV	0.0548	5.75221	1.56884	0.0441
19	15.055	VB	0.0568	5.18546	1.29346	0.0397
20	15.474	BV	0.1184	3566.75928	460.11609	27.3262
21	16.393	VV	0.1886	97.82332	6.32042	0.7495
22	16.652	VV	0.0633	10.88093	2.47462	0.0834
23	16.740	VB	0.1464	23.69060	2.08705	0.1815
24	17.644	BV	0.1168	29.65286	3.50140	0.2272
25	17.763	VV	0.0986	17.41111	2.19315	0.1334
26	17.965	VV	0.0884	9.93611	1.55745	0.0761
27	18.491	VB	0.1411	26.17830	2.36607	0.2006
28	19.670	BV	0.0927	10.56524	1.52812	0.0809
29	19.968	VB	0.1935	26.55933	1.68901	0.2035
30	20.375	VB	0.1105	8.85048	1.14065	0.0678
31	21.172	BB	0.1431	57.63920	5.84245	0.4416
32	21.605	BV	0.0760	14.01579	2.40316	0.1074
33	21.663	VV	0.0675	11.63214	2.36046	0.0891
34	21.774	VV	0.0888	10.88919	1.61177	0.0834
35	21.927	VB	0.0933	8.46644	1.15852	0.0649
36	22.335	VB	0.1356	17.42227	1.64459	0.1335
37	22.932	BB	0.1846	41.91306	2.76924	0.3211
38	23.312	BV	0.0974	11.33646	1.48021	0.0869
39	23.509	VB	0.1331	21.09675	2.21482	0.1616
40	23.867	BV	0.1411	40.41912	4.17057	0.3097
41	24.060	VV	0.0676	7.22749	1.57289	0.0554
42	24.337	BB	0.0935	8.54007	1.13834	0.0654
43	24.785	BB	0.1817	47.72354	3.28026	0.3656
Totals :				1.30525e4	1471.64388	

Signal 2: DAD1 G, Sig=280,4 Ref=off

Peak #	RetTime [min]	Type	Width [min]	Area [mAU*s]	Height [mAU]	Area %
1	1.132	BV	0.2037	114.31159	7.85904	22.0794
2	1.440	VV	0.1057	56.71268	7.21665	10.9541
3	1.513	VB	0.0783	38.25014	7.65647	7.3880
4	1.688	BB	0.0951	24.54965	4.02612	4.7418
5	15.474	BB	0.1097	259.72070	35.37355	50.1652
6	21.177	BB	0.1472	24.18573	2.36691	4.6715

Totals : 517.73049 64.49875

Signal 3: MSD1 TIC, MS File

Peak #	RetTime [min]	Type	Width [min]	Area	Height	Area %
1	5.406	BV	0.0509	8.65871e4	2.74006e4	0.1417
2	5.472	VV	0.0469	7.14280e4	2.53561e4	0.1169
3	5.601	VB	0.1253	2.29307e5	3.05088e4	0.3753
4	5.879	BB	0.0972	1.88375e5	2.64319e4	0.3083
5	6.188	BV	0.0488	5.00021e4	1.76796e4	0.0818
6	6.281	VV	0.1015	1.90463e5	2.54176e4	0.3117
7	6.433	VV	0.0776	1.68709e5	2.88496e4	0.2761
8	6.596	VB	0.0498	7.07801e4	2.30650e4	0.1158
9	6.748	BV	0.0536	1.09155e5	3.21759e4	0.1786
10	6.854	VV	0.0716	1.29267e5	2.85300e4	0.2116
11	6.934	VB	0.0671	1.33601e5	2.69753e4	0.2187
12	7.114	BV	0.1021	1.75096e5	2.94665e4	0.2866
13	7.214	VB	0.0366	7.49518e4	3.20788e4	0.1227
14	7.350	BV	0.1002	1.58639e5	2.74458e4	0.2596
15	7.512	VB	0.0717	1.92067e5	3.59256e4	0.3143
16	7.689	BV	0.0891	1.06606e5	2.08810e4	0.1745
17	7.805	VB	0.0571	1.23850e5	3.02697e4	0.2027

Peak #	RetTime [min]	Type	Width [min]	Area	Height	Area %
18	7.911	BV	0.0561	1.11336e5	3.31014e4	0.1822
19	8.045	VB	0.0916	2.67722e5	3.79643e4	0.4382
20	8.267	BB	0.0601	1.28607e5	3.62529e4	0.2105
21	8.502	BV	0.0906	1.60632e5	2.44662e4	0.2629
22	8.675	VB	0.0410	1.51629e4	6160.70850	0.0248
23	8.764	BB	0.0633	1.24962e5	2.95781e4	0.2045
24	8.892	BB	0.0428	6.92261e4	2.81008e4	0.1133
25	9.070	BV	0.0923	1.96590e5	2.97633e4	0.3217
26	9.143	VV	0.0661	1.46391e5	3.69180e4	0.2396
27	9.230	VB	0.0907	1.79932e5	3.30814e4	0.2945
28	9.439	BV	0.0819	1.63562e5	3.10928e4	0.2677
29	9.612	VB	0.1152	2.28739e5	2.90747e4	0.3744
30	10.077	BV	0.0482	5.30271e4	1.89181e4	0.0868
31	10.197	VB	0.0617	1.50127e5	3.67872e4	0.2457
32	10.670	BV	0.1623	5.01401e5	4.21303e4	0.8206
33	10.993	VV	0.1603	1.29014e6	1.06296e5	2.1115
34	11.084	VV	0.0978	5.32729e5	7.03098e4	0.8719
35	11.274	VB	0.1238	5.50819e5	6.40359e4	0.9015
36	11.604	BB	0.0639	9.60976e4	2.24656e4	0.1573
37	11.730	BV	0.0401	2.79047e4	1.16065e4	0.0457
38	11.806	VB	0.0473	8.38387e4	2.57838e4	0.1372
39	12.042	BV	0.0942	2.03662e5	3.86940e4	0.3333
40	12.235	VB	0.1203	2.64883e5	3.21303e4	0.4335
41	12.447	BB	0.0715	1.39320e5	3.07881e4	0.2280
42	12.608	BV	0.0718	1.83014e5	3.69524e4	0.2995
43	12.693	VB	0.0710	1.05635e5	2.58681e4	0.1729
44	13.121	BB	0.1201	1.81653e5	2.42544e4	0.2973
45	13.256	BB	0.0496	5.82451e4	2.03494e4	0.0953
46	13.532	BV	0.1168	6.92220e5	8.64736e4	1.1329
47	13.625	VB	0.0452	1.12310e5	3.65139e4	0.1838
48	13.820	BV	0.1081	7.32887e5	8.66317e4	1.1995
49	14.068	VB	0.0746	9.93223e4	2.26452e4	0.1626
50	14.381	BV	0.0732	1.04028e5	2.22752e4	0.1703
51	14.471	VB	0.0343	2.60539e4	1.22080e4	0.0426
52	14.834	BV	0.1584	1.18307e6	9.56307e4	1.9362
53	14.997	VB	0.1206	6.84417e5	7.49464e4	1.1201

Peak #	RetTime [min]	Type	Width [min]	Area	Height	Area %
54	15.260	BB	0.0746	1.78176e5	4.46777e4	0.2916
55	15.570	BV	0.1234	3.09594e7	3.79051e6	50.6690
56	16.092	VV	0.1012	4.19696e5	5.93475e4	0.6869
57	16.194	VB	0.0545	1.09732e5	3.35335e4	0.1796
58	16.452	BV	0.1154	4.55843e5	5.50324e4	0.7460
59	16.533	VB	0.0588	1.33532e5	3.88465e4	0.2185
60	16.638	BV	0.0497	1.22890e5	4.02602e4	0.2011
61	16.724	VV	0.0627	2.55754e5	6.12789e4	0.4186
62	16.813	VB	0.0657	2.10770e5	5.34445e4	0.3450
63	17.061	BV	0.1201	1.14817e6	1.26351e5	1.8791
64	17.409	VB	0.1372	4.76256e5	4.58369e4	0.7795
65	17.699	BV	0.0682	3.19268e5	7.52623e4	0.5225
66	17.783	VV	0.0795	3.03803e5	6.32197e4	0.4972
67	17.994	VB	0.1092	3.37245e5	4.58356e4	0.5519
68	18.122	BV	0.1058	2.12272e5	2.84475e4	0.3474
69	18.361	VB	0.0536	8.29360e4	2.77402e4	0.1357
70	18.759	BB	0.2182	8.08621e5	4.67400e4	1.3234
71	18.962	BV	0.0641	1.65633e5	4.26236e4	0.2711
72	19.085	VV	0.0533	9.17150e4	2.43535e4	0.1501
73	19.199	VB	0.0631	1.69460e5	4.45823e4	0.2773
74	19.519	BV	0.1860	2.67336e6	1.97478e5	4.3753
75	19.788	VB	0.0622	9.19934e4	2.51837e4	0.1506
76	19.917	BB	0.1091	5.55587e5	7.56621e4	0.9093
77	20.180	BV	0.0570	1.62411e5	3.97947e4	0.2658
78	20.262	VV	0.0994	2.74045e5	4.59562e4	0.4485
79	20.429	VB	0.0934	3.62418e5	5.66418e4	0.5931
80	20.627	BB	0.0414	7.51513e4	3.02465e4	0.1230
81	20.884	BB	0.0899	2.56704e5	3.94663e4	0.4201
82	21.058	BB	0.0322	1.47720e4	7657.53955	0.0242
83	21.168	BV	0.0602	1.88891e5	5.19495e4	0.3091
84	21.232	VB	0.0584	2.03846e5	5.81500e4	0.3336
85	21.336	BB	0.0563	1.45979e5	4.02945e4	0.2389
86	21.667	BV	0.0975	4.35743e5	6.45250e4	0.7132
87	21.783	VV	0.0925	5.77925e5	8.59091e4	0.9458
88	21.890	VB	0.1159	1.08985e6	1.24818e5	1.7837
89	22.186	BB	0.0703	1.57315e5	3.55622e4	0.2575

Peak #	RetTime [min]	Type	Width [min]	Area	Height	Area %
90	22.353	BV	0.0814	2.78758e5	5.18809e4	0.4562
91	22.469	VB	0.0819	3.48216e5	5.98241e4	0.5699
92	22.714	BB	0.0379	2.27033e4	9972.55859	0.0372
93	22.902	BV	0.0872	3.96666e5	6.91350e4	0.6492
94	22.968	VB	0.1085	5.12502e5	7.86950e4	0.8388
95	23.370	BV	0.2654	2.42071e6	1.21420e5	3.9618
96	23.800	VB	0.0360	3.50030e4	1.53370e4	0.0573
97	24.242	BV	0.0854	1.63495e5	3.41487e4	0.2676
98	24.426	VB	0.0993	3.80514e5	5.50725e4	0.6228
99	24.960	BBA	0.0609	2.06906e5	5.15140e4	0.3386
Totals :				6.11011e7	8.14292e6	

===

*** End of Report ***

Data File D:\EZXDATA\MARIARODRIGUEZ\10-24\091024-10-75OVER20_PEPTIDES-C3_200M-30035.D
Sample Name: DMSO-MUT73

```
=====================================================================
Acq. Operator   : Maria Rodriguez
Acq. Instrument : INSTRUMENT 1                  Location : P2-E-01
Injection Date  : 10/9/2024 6:16:34 PM               Inj :   1
                                              Inj Volume : 7.000 µl
Acq. Method     : C:\CHEM32\1\METHODS\10-75OVER20_PEPTIDE
Last changed    : 10/9/2024 6:15:43 PM by Maria Rodriguez
                  (modified after loading)
Analysis Method : C:\CHEM32\1\METHODS\10-75OVER20_PEPTIDES-C3.M
Last changed    : 10/10/2024 9:48:42 AM by Kevin Haubrich
                  (modified after loading)
Sample Info     : Easy-Access Method: '10-75over20-C3(200+m/Z)'
```

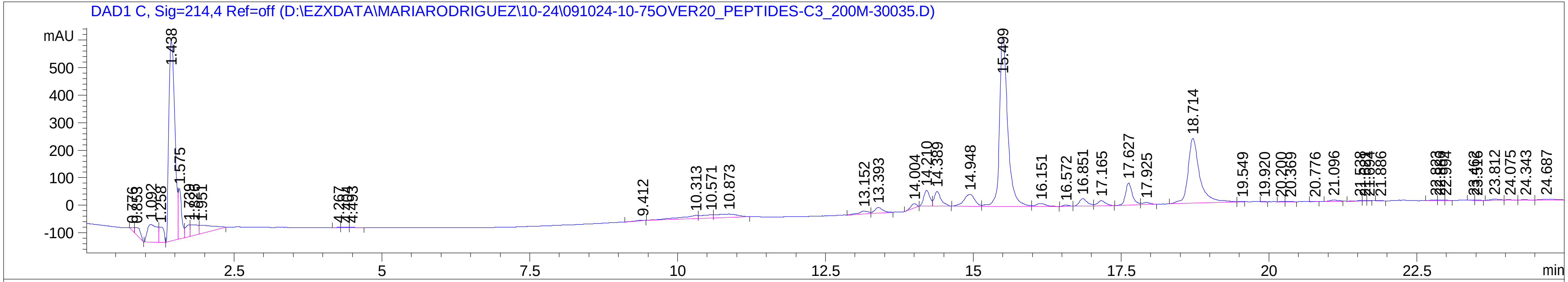

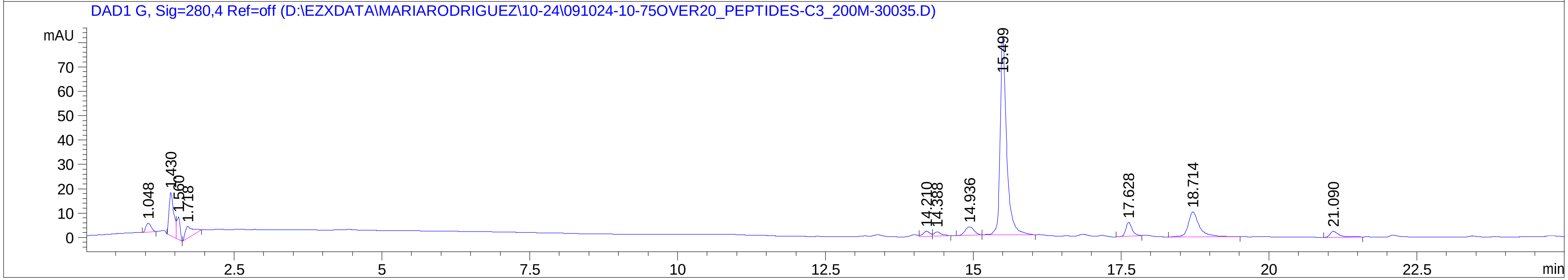

Data File D:\EZXDATA\MARIARODRIGUEZ\10-24\091024-10-75OVER20_PEPTIDES-C3_200M-30035.D
Sample Name: DMSO-MUT73

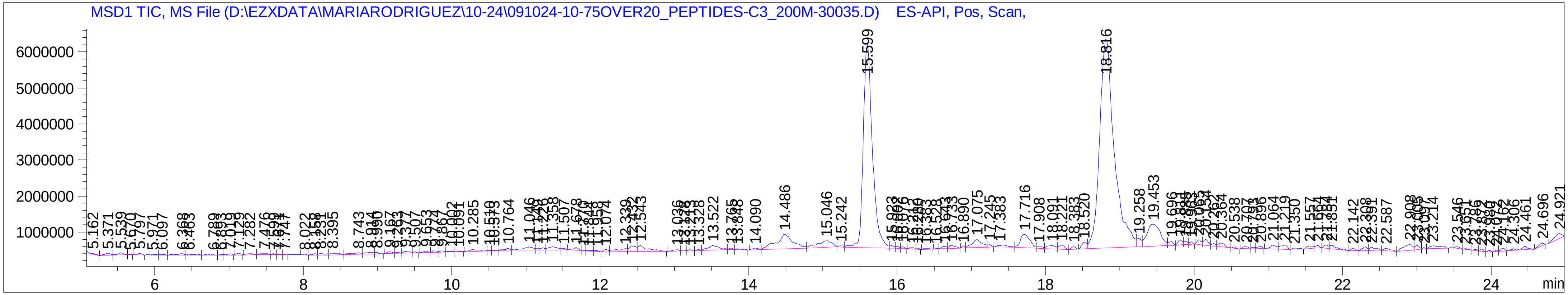

===

Area Percent Report

===

Sorted By : Signal
Multiplier : 1.0000
Dilution : 1.0000
Sample Amount: : 15.00000 [ng/ul] (not used in calc.)
Use Multiplier & Dilution Factor with ISTDs

Signal 1: DAD1 C, Sig=214,4 Ref=off

Peak #	RetTime [min]	Type	Width [min]	Area [mAU*s]	Height [mAU]	Area %
1	0.776	BV	0.0599	43.35289	10.12656	0.2008
2	0.853	VB	0.1132	195.89151	26.83252	0.9074
3	1.092	BV	0.1649	733.84766	64.44228	3.3993
4	1.258	VB	0.0925	318.58902	55.92080	1.4758
5	1.438	BV	0.1162	5092.07813	723.08478	23.5876
6	1.575	VV	0.0618	749.66638	182.96622	3.4726
7	1.739	VV	0.0787	224.60736	41.78071	1.0404
8	1.826	VV	0.1206	338.33044	37.72694	1.5672
9	1.951	VB	0.1877	429.00598	29.15334	1.9872
10	4.267	BV	0.0685	5.58440	1.19546	0.0259

Peak #	RetTime [min]	Type	Width [min]	Area [mAU*s]	Height [mAU]	Area %
11	4.404	VV	0.0938	12.13323	1.64961	0.0562
12	4.493	VB	0.0924	13.25643	1.92421	0.0614
13	9.412	BB	0.2057	19.53446	1.20150	0.0905
14	10.313	BV	0.2773	261.23181	11.66482	1.2101
15	10.571	VV	0.1775	168.70161	11.60892	0.7815
16	10.873	VB	0.3067	315.31955	12.40031	1.4606
17	13.152	BV	0.1599	120.25797	10.47704	0.5571
18	13.393	VB	0.1403	193.89584	20.50147	0.8982
19	14.004	BB	0.1120	103.53791	15.10628	0.4796
20	14.210	BV	0.1091	411.16946	57.69999	1.9046
21	14.389	VB	0.1189	441.13449	54.21389	2.0434
22	14.948	BV	0.2023	558.01599	43.32595	2.5849
23	15.499	VV	0.1476	5954.70020	611.62744	27.5835
24	16.151	VB	0.1798	132.17288	10.87807	0.6123
25	16.572	BB	0.1108	29.60848	4.27539	0.1372
26	16.851	BV	0.1395	248.84172	26.50374	1.1527
27	17.165	VB	0.1311	171.82030	18.36898	0.7959
28	17.627	BV	0.1201	648.35278	80.41986	3.0033
29	17.925	VB	0.1332	71.34019	7.62442	0.3305
30	18.714	VV	0.2047	3249.46460	235.90005	15.0522
31	19.549	VV	0.0856	11.85566	1.82875	0.0549
32	19.920	VB	0.0765	5.58846	1.15486	0.0259
33	20.200	BV	0.0799	8.10616	1.43439	0.0375
34	20.369	VB	0.1115	10.85225	1.27245	0.0503
35	20.776	VV	0.0784	6.11835	1.07505	0.0283
36	21.096	BV	0.1463	53.81184	5.21716	0.2493
37	21.538	BV	0.1062	17.95077	2.22366	0.0832
38	21.621	VV	0.0572	7.24885	1.79179	0.0336
39	21.694	VV	0.0599	7.74972	1.88783	0.0359
40	21.886	VB	0.0852	9.90696	1.53730	0.0459
41	22.823	BV	0.1048	19.09279	2.30218	0.0884
42	22.886	VV	0.0881	15.30673	2.34739	0.0709
43	22.994	VB	0.0491	5.15072	1.39428	0.0239
44	23.462	BV	0.0565	6.25489	1.44565	0.0290
45	23.516	VB	0.0614	7.93142	1.87342	0.0367
46	23.812	BV	0.1496	46.49776	4.00139	0.2154

Peak #	RetTime [min]	Type	Width [min]	Area [mAU*s]	Height [mAU]	Area %
47	24.075	VB	0.1138	13.40295	1.47844	0.0621
48	24.343	BB	0.1154	13.99213	1.54842	0.0648
49	24.687	BBA	0.2035	65.67786	3.96027	0.3042

Totals : 2.15879e4 2450.37622

Signal 2: DAD1 G, Sig=280,4 Ref=off

Peak #	RetTime [min]	Type	Width [min]	Area [mAU*s]	Height [mAU]	Area %
1	1.048	BB	0.1029	22.70266	3.63416	2.0819
2	1.430	BV	0.0834	98.62122	17.60406	9.0439
3	1.560	VB	0.0579	35.26675	9.37017	3.2341
4	1.718	BB	0.1263	42.05105	4.70220	3.8562
5	14.210	VV	0.1227	17.63819	2.12753	1.6175
6	14.388	VB	0.1264	15.68754	1.78766	1.4386
7	14.936	BB	0.1623	36.32675	3.46599	3.3313
8	15.499	BB	0.1112	619.26404	80.97622	56.7884
9	17.628	BB	0.1120	41.97107	5.69579	3.8489
10	18.714	BB	0.1929	136.03581	10.24267	12.4749
11	21.090	BB	0.1537	24.91153	2.42875	2.2845

Totals : 1090.47660 142.03520

Signal 3: MSD1 TIC, MS File

Peak #	RetTime [min]	Type	Width [min]	Area	Height	Area %
1	5.162	BB	0.0540	8.99913e4	2.77540e4	0.0483
2	5.371	BV	0.0797	3.09442e5	5.91823e4	0.1662

Peak #	RetTime [min]	Type	Width [min]	Area	Height	Area %
3	5.539	VV	0.0921	3.27657e5	5.92995e4	0.1760
4	5.670	VB	0.0387	4.64131e4	1.84306e4	0.0249
5	5.797	BB	0.0700	1.71947e5	4.09584e4	0.0924
6	5.971	BV	0.0466	6.90253e4	2.16167e4	0.0371
7	6.097	VV	0.0677	1.00136e5	2.17804e4	0.0538
8	6.368	VB	0.1073	2.88161e5	3.79734e4	0.1548
9	6.463	BV	0.0919	2.38351e5	3.37052e4	0.1280
10	6.789	VB	0.1315	2.02651e5	2.56818e4	0.1088
11	6.893	BV	0.0373	7.50841e4	3.35842e4	0.0403
12	7.019	VB	0.0900	1.90515e5	3.42594e4	0.1023
13	7.129	BB	0.0624	1.96576e5	5.25119e4	0.1056
14	7.282	BV	0.0893	2.29829e5	3.35642e4	0.1234
15	7.476	VB	0.1120	2.76791e5	3.64299e4	0.1487
16	7.599	BV	0.0465	7.94861e4	2.85569e4	0.0427
17	7.671	VV	0.0395	9.32369e4	3.60472e4	0.0501
18	7.747	VB	0.0422	4.42818e4	1.83664e4	0.0238
19	8.022	BB	0.0359	5.03211e4	2.21135e4	0.0270
20	8.158	BV	0.0764	1.44160e5	3.64385e4	0.0774
21	8.231	VV	0.0816	2.52064e5	4.06980e4	0.1354
22	8.395	VB	0.1108	4.28245e5	5.16196e4	0.2300
23	8.743	BV	0.1472	4.75677e5	4.65705e4	0.2555
24	8.914	VV	0.1052	4.19086e5	5.65234e4	0.2251
25	8.990	VB	0.0488	1.54597e5	4.57158e4	0.0830
26	9.167	BV	0.0772	2.52281e5	5.03237e4	0.1355
27	9.263	VB	0.0500	9.19646e4	2.98131e4	0.0494
28	9.377	BV	0.0764	2.37851e5	4.13975e4	0.1277
29	9.507	VB	0.0369	6.30701e4	2.67406e4	0.0339
30	9.653	BB	0.0699	1.74896e5	3.98576e4	0.0939
31	9.774	BV	0.0692	1.89473e5	4.00698e4	0.1018
32	9.867	VV	0.0564	1.45473e5	4.01086e4	0.0781
33	10.002	VB	0.0984	1.24484e5	2.10925e4	0.0669
34	10.091	BB	0.0528	6.81414e4	1.82872e4	0.0366
35	10.285	BV	0.1234	5.08779e5	5.42961e4	0.2733
36	10.510	VB	0.0402	4.40549e4	1.96416e4	0.0237
37	10.573	BB	0.0411	2.87494e4	1.35255e4	0.0154
38	10.764	BB	0.0722	2.67534e5	5.36570e4	0.1437

Peak #	RetTime [min]	Type	Width [min]	Area	Height	Area %
39	11.046	BV	0.1410	8.32849e5	8.60745e4	0.4473
40	11.149	VV	0.0452	1.08797e5	4.00968e4	0.0584
41	11.226	VV	0.0636	2.56917e5	6.04284e4	0.1380
42	11.358	VV	0.1222	7.48858e5	9.27904e4	0.4022
43	11.507	VB	0.0432	1.45958e5	5.02561e4	0.0784
44	11.678	BV	0.0728	3.80283e5	7.55130e4	0.2042
45	11.770	VV	0.0404	4.92857e4	1.85125e4	0.0265
46	11.844	VV	0.0846	1.07330e5	2.11460e4	0.0576
47	11.958	VB	0.0574	1.13509e5	3.42261e4	0.0610
48	12.074	BV	0.0668	2.73223e5	6.63191e4	0.1467
49	12.339	VV	0.1328	7.90034e5	8.46755e4	0.4243
50	12.432	VV	0.0883	9.78608e5	1.53642e5	0.5256
51	12.543	VB	0.1550	1.53549e6	1.65139e5	0.8247
52	13.036	BV	0.0807	2.10910e5	3.69231e4	0.1133
53	13.128	VV	0.0527	1.02432e5	2.75854e4	0.0550
54	13.213	VB	0.0603	1.29703e5	3.63508e4	0.0697
55	13.328	BV	0.0475	8.77545e4	2.68318e4	0.0471
56	13.522	VV	0.1444	1.39076e6	1.34127e5	0.7470
57	13.765	VB	0.0540	1.27645e5	3.94619e4	0.0686
58	13.848	BB	0.0830	1.28604e5	2.03652e4	0.0691
59	14.090	BV	0.0804	2.22462e5	3.91381e4	0.1195
60	14.486	VV	0.2028	6.14744e6	3.99846e5	3.3017
61	15.046	VB	0.1772	2.53498e6	1.86609e5	1.3615
62	15.242	BV	0.0642	1.42148e5	3.71692e4	0.0763
63	15.599	VV	0.1280	4.84270e7	5.65513e6	26.0096
64	15.923	VV	0.0651	2.92125e5	7.48245e4	0.1569
65	16.002	VV	0.0517	1.84425e5	5.94974e4	0.0991
66	16.076	VB	0.0487	1.61148e5	5.42720e4	0.0866
67	16.212	BV	0.0779	1.94522e5	4.16620e4	0.1045
68	16.280	VB	0.0373	9.11080e4	4.07278e4	0.0489
69	16.383	BB	0.0673	1.32714e5	2.67326e4	0.0713
70	16.528	BB	0.0347	3.75122e4	1.72468e4	0.0201
71	16.643	BV	0.0672	2.97259e5	6.52525e4	0.1597
72	16.733	VB	0.0819	4.48490e5	8.28288e4	0.2409
73	16.890	BV	0.0370	8.18217e4	3.44755e4	0.0439
74	17.075	VV	0.1235	1.94546e6	2.16695e5	1.0449

Peak #	RetTime [min]	Type	Width [min]	Area	Height	Area %
75	17.245	VB	0.0505	2.23633e5	6.34090e4	0.1201
76	17.383	BB	0.1205	3.79669e5	4.35041e4	0.2039
77	17.716	BV	0.1329	3.20627e6	3.73426e5	1.7221
78	17.908	VV	0.0772	2.66315e5	4.58102e4	0.1430
79	18.091	VV	0.1106	7.33027e5	9.80585e4	0.3937
80	18.221	VB	0.0832	5.83274e5	1.13801e5	0.3133
81	18.383	BB	0.0595	3.11526e5	7.99412e4	0.1673
82	18.520	BV	0.0794	8.39790e5	1.78377e5	0.4510
83	18.816	VV	0.2004	7.88456e7	5.81221e6	42.3471
84	19.258	VV	0.0592	1.01895e6	2.38504e5	0.5473
85	19.453	VV	0.1844	7.74665e6	6.14954e5	4.1606
86	19.696	VB	0.0553	4.64238e5	1.17954e5	0.2493
87	19.811	BV	0.0653	5.69764e5	1.42858e5	0.3060
88	19.885	VV	0.0567	3.34447e5	9.14517e4	0.1796
89	19.963	VB	0.0535	2.18019e5	6.43989e4	0.1171
90	20.065	BV	0.0598	5.19199e5	1.32265e5	0.2789
91	20.154	VV	0.0650	6.99654e5	1.60046e5	0.3758
92	20.262	VV	0.0554	2.39339e5	6.75807e4	0.1285
93	20.364	VB	0.0914	5.86836e5	1.08076e5	0.3152
94	20.538	BB	0.0450	9.54209e4	3.59620e4	0.0512
95	20.707	BB	0.0759	2.44170e5	4.77376e4	0.1311
96	20.793	BV	0.0447	7.36626e4	2.75492e4	0.0396
97	20.896	VB	0.0572	2.37402e5	5.78429e4	0.1275
98	21.064	BV	0.0839	5.79655e5	1.03792e5	0.3113
99	21.219	VB	0.0981	7.54034e5	1.17754e5	0.4050
100	21.350	BB	0.0887	1.79005e5	2.98400e4	0.0961
101	21.557	BV	0.0878	4.49916e5	7.59567e4	0.2416
102	21.664	VB	0.0589	2.62331e5	6.81594e4	0.1409
103	21.784	BV	0.0576	3.58112e5	9.59996e4	0.1923
104	21.851	VB	0.0992	6.52376e5	9.45777e4	0.3504
105	22.142	BB	0.0567	2.26697e5	6.97221e4	0.1218
106	22.308	BV	0.0753	4.33845e5	9.75049e4	0.2330
107	22.391	VB	0.0898	4.82402e5	8.18247e4	0.2591
108	22.587	BB	0.0934	4.32911e5	6.76710e4	0.2325
109	22.908	BV	0.1221	1.47220e6	1.68331e5	0.7907
110	23.005	VV	0.0660	4.48843e5	1.10670e5	0.2411

Peak #	RetTime [min]	Type	Width [min]	Area	Height	Area %
111	23.097	VB	0.0445	9.57994e4	3.64362e4	0.0515
112	23.214	BB	0.1560	8.84853e5	7.52264e4	0.4752
113	23.546	BB	0.0654	2.24388e5	5.09878e4	0.1205
114	23.651	BB	0.0612	8.47926e4	2.09703e4	0.0455
115	23.786	BV	0.0562	9.99300e4	2.76750e4	0.0537
116	23.872	VB	0.0535	2.02058e5	6.29720e4	0.1085
117	23.980	BB	0.0495	1.00437e5	3.30855e4	0.0539
118	24.072	BV	0.0383	8.61659e4	3.74868e4	0.0463
119	24.162	VB	0.0556	2.93305e5	8.24272e4	0.1575
120	24.306	BV	0.0608	1.56349e5	4.33727e4	0.0840
121	24.461	VB	0.0787	5.16551e5	9.31884e4	0.2774
122	24.696	BB	0.0817	3.20767e5	5.94239e4	0.1723
123	24.921	BBA	0.1030	9.64187e5	1.33492e5	0.5179

Totals : 1.86189e8 2.03250e7

===

*** End of Report ***

Spectrum Deconvolution
Data File D:\EZXDATA\MARIARODRIGUEZ\10-24\091024-10-75OVER20_PEPTIDES-C3_200M-30035.D
Sample Name: DMSO-MUT73

```
    Easy-Access Method: '10-75over20-C3(200+m/Z)'
    =====================================================================
    Acq. Operator   : Maria Rodriguez
    Acq. Instrument : INSTRUMENT 1                     Location : P2-E-01
    Injection Date  : 10/9/2024 6:16:34 PM                  Inj :   1
                                                     Inj Volume : 7.000 µl
    Acq. Method     : C:\CHEM32\1\METHODS\10-75OVER20_PEPTIDE
    Last changed    : 10/9/2024 6:15:43 PM by Maria Rodriguez
                      (modified after loading)
    Analysis Method : C:\CHEM32\1\METHODS\10-75OVER20_PEPTIDES-C3.M
    Last changed    : 10/10/2024 10:05:22 AM by Kevin Haubrich
                      (modified after loading)
    Sample Info     : Easy-Access Method: '10-75over20-C3(200+m/Z)'


    =====================================================================
                          Deconvolution Parameters
    =====================================================================

    Adduct Ion(Positive): +H, 1.0079 Da
    Adduct Ion(Negative):  , 0.0000 Da
    Low MW:               10000
    DeconvStartChgMaximum Charge:       50
    Minimum Peaks in Set: 3
    Retain Residual:      No
    Ion PWHH:             0.6 Da
    MW Agreement:         0.05 %
    Noise Cutoff:         1000 counts
    Abundance Cutoff:     10 %
    MW Assign:            Curve fit
    MW Assign Cutoff:     40 %
    Envelope Cutoff:      50 %
```

Spectrum Deconvolution
Data File D:\EZXDATA\MARIARODRIGUEZ\10-24\091024-10-75OVER20_PEPTIDES-C3_200M-30035.D
Sample Name: DMSO-MUT73

Deconvolution of Spectrum # 1 @ 15.628 min

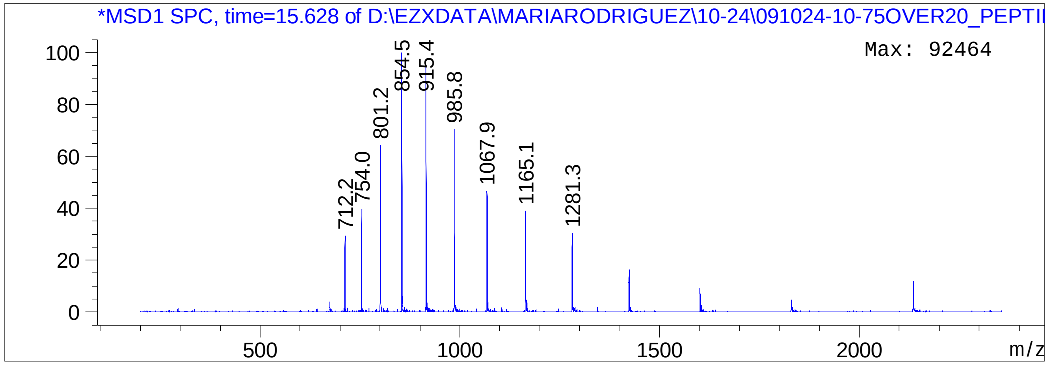

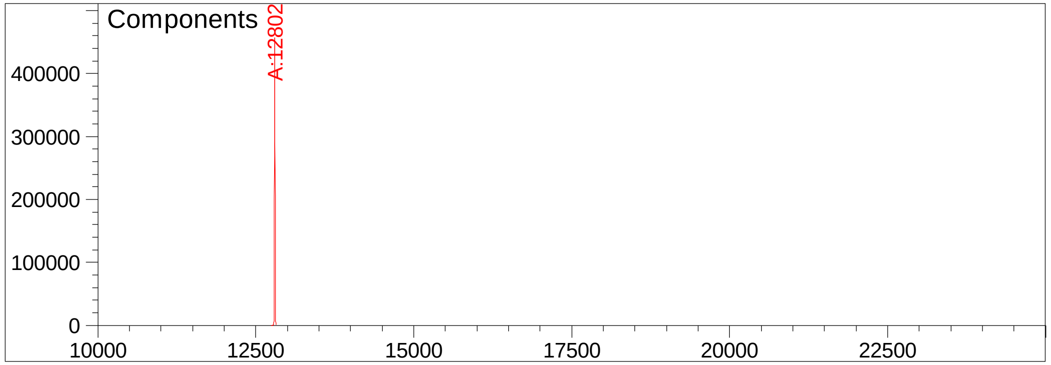

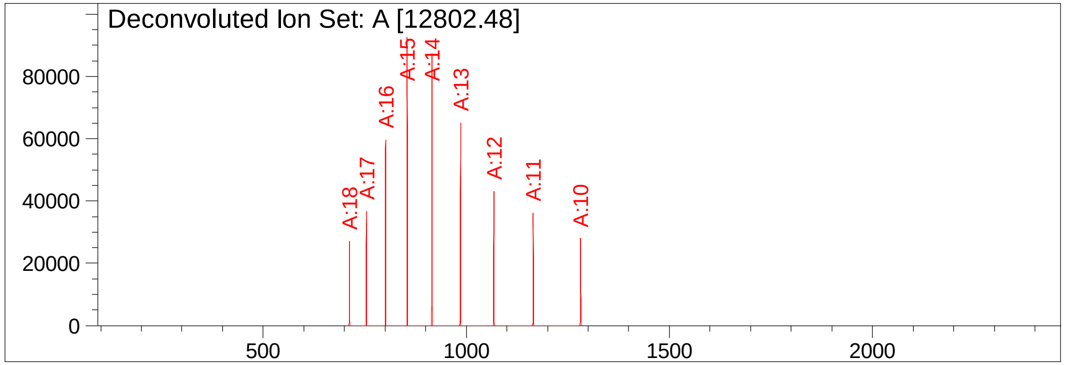

Component	Molecular Weight	Absolute Abundance	Relative Abundance
A	12802.48	457055	100.00

*** End of Report ***
