# Supplementary figures and images for: BromoCatch: a self-labelling tag platform for protein modification and live cell imaging
Source: Nat Commun. 2026 May 13;17:6406. doi: 10.1038/s41467-026-72539-w (PMC13376172; doi:10.1038/s41467-026-72539-w)

Brd4BD2 L387A,E438C + DMSO

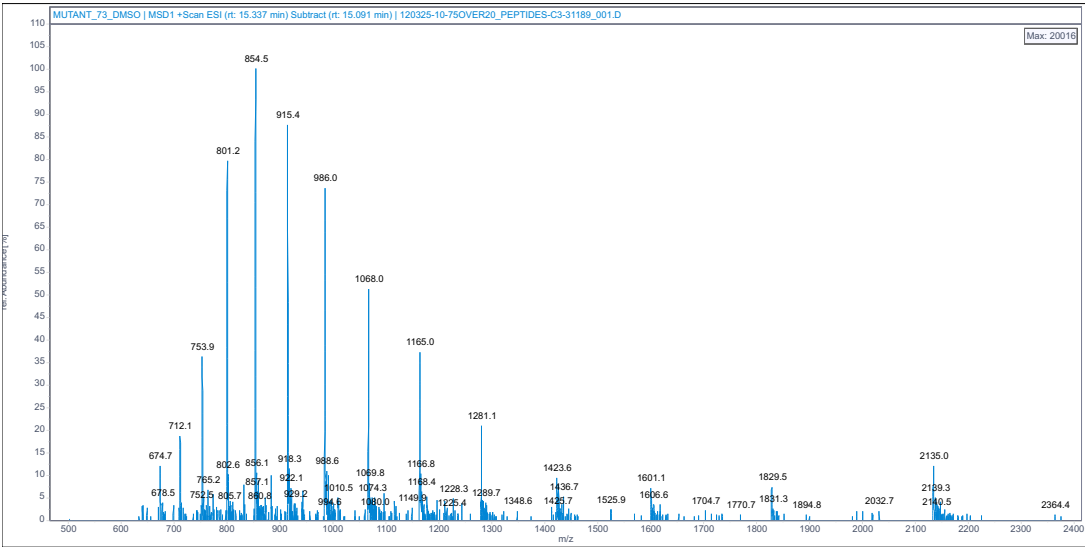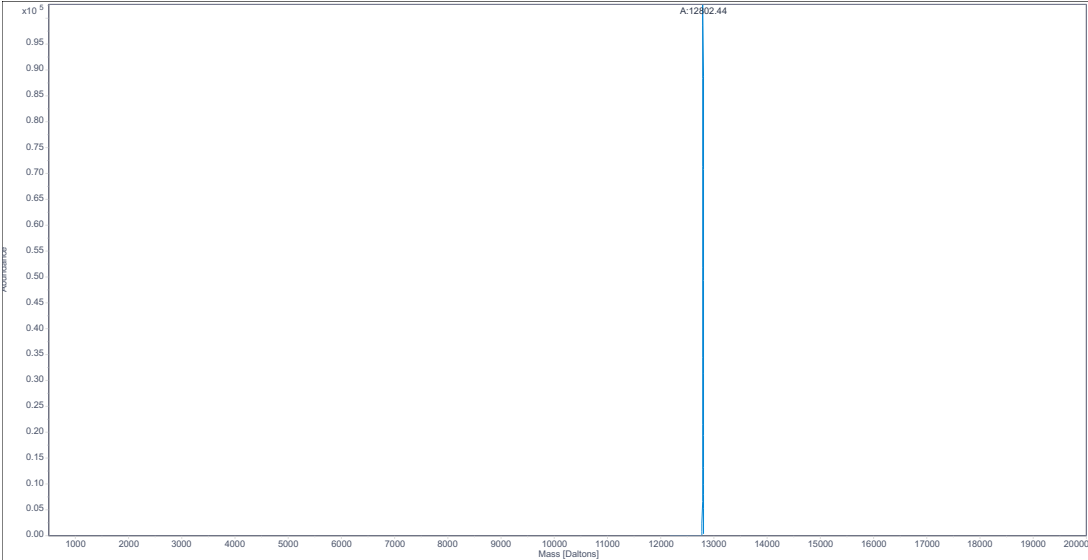

Brd4BD2 L387A,E438C + MR169

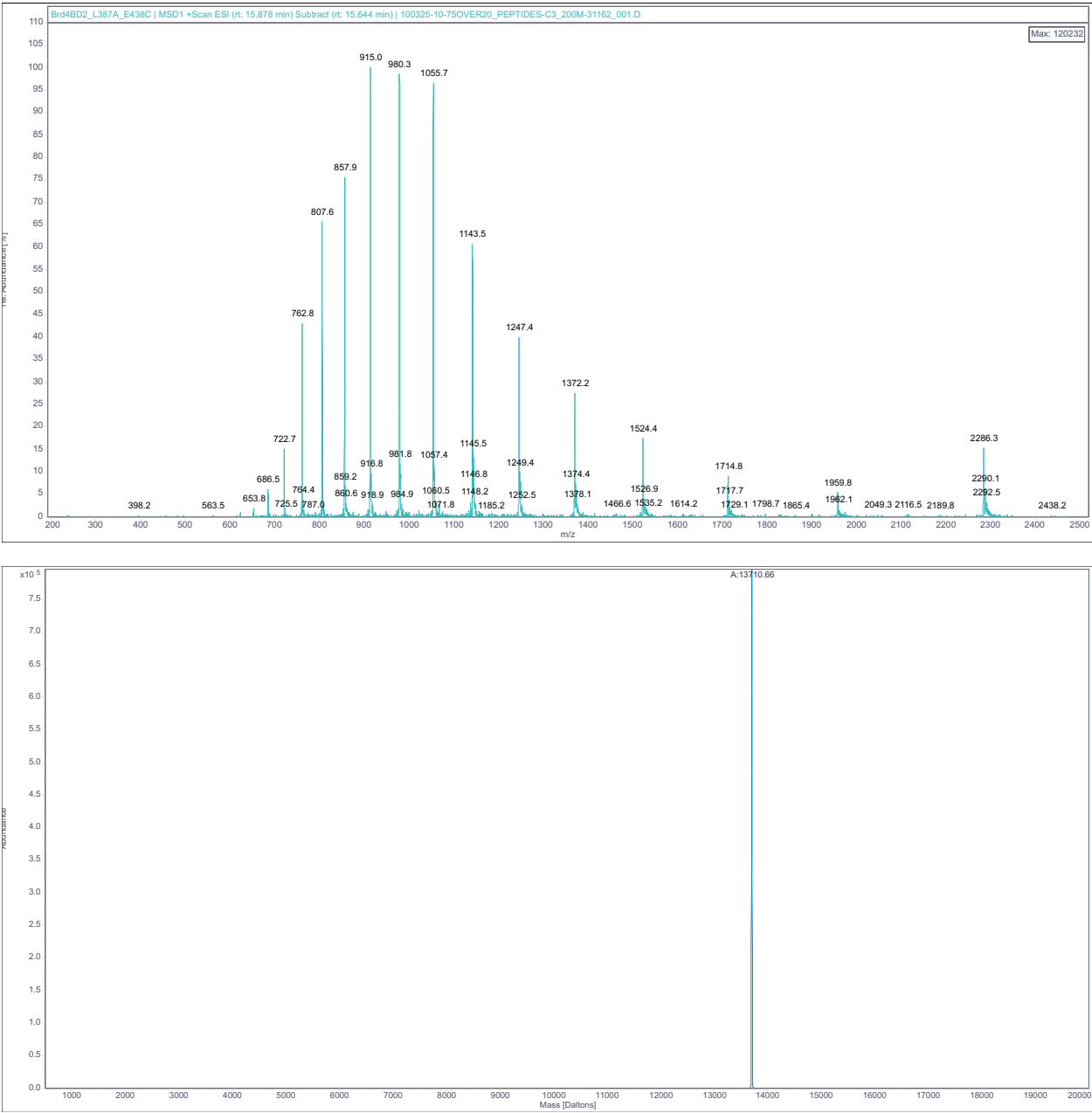

Supplement: Supplementary file 3 — Supplementary Data 1 [file 41467_2026_72539_MOESM3_ESM.zip › PUBLICATION INTACT MS/Supplementary Figure 7B - MR169 INTACT-MS reacted .pdf]
