# Supplementary material for: BromoCatch: a self-labelling tag platform for protein modification and live cell imaging
Source: Nat Commun. 2026 May 13;17:6406. doi: 10.1038/s41467-026-72539-w (PMC13376172; doi:10.1038/s41467-026-72539-w)
Supplement: Supplementary file 5 — Reporting Summary [file 41467_2026_72539_MOESM5_ESM.pdf]

nature portfolio

Corresponding author(s): Alessio Ciulli

Last updated by author(s): Mar 30, 2026

Reporting Summary

Nature Portfolio wishes to improve the reproducibility of the work that we publish. This form provides structure for consistency and transparency in reporting. For further information on Nature Portfolio policies, see our Editorial Policies and the Editorial Policy Checklist.

Statistics

For all statistical analyses, confirm that the following items are present in the figure legend, table legend, main text, or Methods section.

n/a	Confirmed	
☐	☒	The exact sample size (*n*) for each experimental group/condition, given as a discrete number and unit of measurement
☐	☒	A statement on whether measurements were taken from distinct samples or whether the same sample was measured repeatedly
☐	☒	The statistical test(s) used AND whether they are one- or two-sided<br>*Only common tests should be described solely by name; describe more complex techniques in the Methods section.*
☒	☐	A description of all covariates tested
☒	☐	A description of any assumptions or corrections, such as tests of normality and adjustment for multiple comparisons
☐	☒	A full description of the statistical parameters including central tendency (e.g. means) or other basic estimates (e.g. regression coefficient) AND variation (e.g. standard deviation) or associated estimates of uncertainty (e.g. confidence intervals)
☐	☒	For null hypothesis testing, the test statistic (e.g. *F*, *t*, *r*) with confidence intervals, effect sizes, degrees of freedom and *P* value noted<br>*Give P values as exact values whenever suitable.*
☐	☒	For Bayesian analysis, information on the choice of priors and Markov chain Monte Carlo settings
☒	☐	For hierarchical and complex designs, identification of the appropriate level for tests and full reporting of outcomes
☐	☒	Estimates of effect sizes (e.g. Cohen's *d*, Pearson's *r*), indicating how they were calculated

Our web collection on statistics for biologists contains articles on many of the points above.

Software and code

Policy information about availability of computer code

Data collection	X-ray diffraction data: Diffraction data were collected at Diamond Light Source beamline i03/i04 or The European Synchrotron Radiation Facility ID30-A1<br>Western blotting/SDS gels: ChemiDoc Touch imaging system (BioRad) operated by Image Lab (v2.4.0.03).<br>NMR spectroscopy: Avance III 500 MHz Bruker spectrometer<br>Fluorescence-based assays: BMG Labtech PHERAstar (firmware v1.33) and GloMax® Discover System (software v4.0.0, firmware v4.92)<br>LC-MS : LC Agilent 1290 Infinity/ Infinity III (Agilent) connected to a MS single quadrupole mass spectrometer (Agilent)<br>NanoBRET engagment and residence time assays: BMG Labtech PHERAstar (firmware v1.33) and GloMax® Discover System (software v4.0.0, firmware v4.92)<br>Confocal microscopy iimaging acquired on a Zeiss 880 Airyscan Confocal Microscope using Plan-Apochromat 63x/1.4 objective lens and equipped with 488/561/633-nm excitation laser lines.<br>HiBIT Lytic Assay : BMG Labtech PHERAstar (firmware v1.33)<br>FP competition assay : BMG Labtech PHERAstar (firmware v1.33)<br>Fluorescence emission and excitation spectrum measurements: FlexStation 3 Multi-Mode Microplate Reader<br>Proteomics analysis was acquired using an Orbitrap Ascend Tribrid mass spectrometer coupled with Thermo Fisher Scientific Vanquish Neo UHPLC.
Data analysis	X-ray structure solution: Diffraction images were processed with Xia2 Dials. The structure was solved by molecular replacement using a a search model. Subsequent iterative model building, and refinement was done according to standard protocols using COOT and CPP4. Ligand restraints were generated using the CPP4.<br>NMR spectroscopy: All NMR data were processed and analysed using TopSpin (Bruker) and analysed with Mnova .

Fluorescence-based and FP assays GraphPad Prism 10
LC-MS : ChemStation / DataAnalysis Agilent software
Fluorescence emission and excitation spectrum measurements: FlexStation 3 Multi-Mode Microplate Reader
Western blot imaging: Image Lab (Bio-Rad)
Confocal microscopy images and post-acquisition analysis was conducted with Fiji v 1.54
NanoBRET engagment, residence time assays and HiBIT Lytic assays analysed in GraphPad Prism 10.
The proteomics raw data were searched using SEQUEST HT search engines with Proteome Discoverer 3.0 (Thermo Fisher Scientific) and analysed using the Proteome Discoverer 3.0 (Thermo Fisher Scientific) and Perseus v2.1 . The analyzed data was used to generate volcano plots using GraphPad Prism software.

For manuscripts utilizing custom algorithms or software that are central to the research but not yet described in published literature, software must be made available to editors and reviewers. We strongly encourage code deposition in a community repository (e.g. GitHub). See the Nature Portfolio guidelines for submitting code & software for further information.

Data

Policy information about availability of data

All manuscripts must include a data availability statement. This statement should provide the following information, where applicable:

- Accession codes, unique identifiers, or web links for publicly available datasets
- A description of any restrictions on data availability
- For clinical datasets or third party data, please ensure that the statement adheres to our policy

Data availability
X-ray crystallographic data have been deposited in the Protein Data Bank, accession code of Brd2-BD2L383A,D434C in complex with compound MR116 is PDB 9QRK. [https://doi.org/10.2210/pdb9QRK/pdb]. All other data supporting the findings of this study are provided in the main figures and in the Source data file and Supplementary Information files, including uncropped gels. Plasmids generated in this study are available from the corresponding author upon reasonable request. All mass spectrometry proteomics (Biotin affinity pull-down mass spectrometry analysis in a transiently transfected BromoCatch-GFP HEK293 line) data has been deposited in the PRIDE data repository under accession codes: PXD074567 [http://proteomecentral.proteomexchange.org/cgi/GetDataset?ID= PXD074567]

Research involving human participants, their data, or biological material

Policy information about studies with human participants or human data. See also policy information about sex, gender (identity/presentation), and sexual orientation and race, ethnicity and racism.

Reporting on sex and gender	Not applicable
Reporting on race, ethnicity, or other socially relevant groupings	Not applicable
Population characteristics	Not applicable
Recruitment	Not applicable
Ethics oversight	Not applicable

Note that full information on the approval of the study protocol must also be provided in the manuscript.

Field-specific reporting

Please select the one below that is the best fit for your research. If you are not sure, read the appropriate sections before making your selection.

☒ Life sciences ☐ Behavioural & social sciences ☐ Ecological, evolutionary & environmental sciences

For a reference copy of the document with all sections, see nature.com/documents/nr-reporting-summary-flat.pdf

Life sciences study design

All studies must disclose on these points even when the disclosure is negative.

Sample size	Sample sizes were not predetermined using statistical analyses. Sample sizes were based on prior experience in the field and our previous studies (Zengerle et al. ACS Chem Biol 2015; Gadd et al. Nat. Chem. Biol. 2017; Kung et al. Nat Commun. 2019; Imaide et al. Nat Chem Biol. 2021).
Data exclusions	No data excluded
Replication	Unless stated in figure legends or method sections, all experiments were done at least twice and the reproduction were successful. The number of technical replicates and independent biological experiments are specified in the respective figure legends.
Randomization	There was no requirement for randomization of data.
Blinding	No blinding was performed, as no subjective measurements were done.

Reporting for specific materials, systems and methods

We require information from authors about some types of materials, experimental systems and methods used in many studies. Here, indicate whether each material, system or method listed is relevant to your study. If you are not sure if a list item applies to your research, read the appropriate section before selecting a response.

Materials & experimental systems

n/a	Involved in the study
☐	☒ Antibodies
☐	☒ Eukaryotic cell lines
☒	☐ Palaeontology and archaeology
☒	☐ Animals and other organisms
☒	☐ Clinical data
☒	☐ Dual use research of concern
☒	☐ Plants

Methods

n/a	Involved in the study
☒	☐ ChIP-seq
☒	☐ Flow cytometry
☒	☐ MRI-based neuroimaging

Antibodies

Antibodies used

The following primary antibodies were used for immunoblot analyses: Histone H2B (D2H6) Rabbit mAb #12364 (cell signalling technologies, 1:1000 dilution), and IRDye® 800CW Streptavidin (LICORbio, 926-32230), BRD4 EPR5150(2), Anti-HiBiT Monoclonal antibody N720A, anti-GFP polyclonal antibody DU 1574.

The following secondary antibodies used were IRDye® 800CW anti-rabbit (no. 926-32211, LiCor, 1:10000 dilution), and hFABTM rhodamine anti-tubulin (no. 12004165, Biorad, 1:5000 dilution), IRDye® 800CW anti-sheep (Thermo Fisher, SA5-10060)

Validation

Target specificity for the following antibodies has been confirmed by commercial vendors: Histone H2B (D2H6) Rabbit mAb #12364 (cell signalling technologies, 1:1000 dilution), and IRDye® 800CW Streptavidin (LICORbio, 926-32230)

Target specificity of anti-GFP polyclonal antibody GFP (S268B, DU 1574). This antibody has been validated by the Western Blotting assays in Supplementary Figure 12 and 28 where detection of GFP is absent in WT cells and detected in the BromoCatch-GFP transfected cell lines. This anti-GFP antibody was validated in previous work by R J Nichols et al. Biochem. J. (2010) 430, 393–404, where GFP inducible expressing cells showed absence of bands until GFP expression was induced.

Eukaryotic cell lines

Policy information about cell lines and Sex and Gender in Research

Cell line source(s)

HEK293, HEK293FT and U2OS cell lines were used in this paper. U2OS and HEK293 cells were obtained from ATCC, HEK293FT Cell Line was obtained from Invitrogen (Cat. #R70007).

Authentication

Cell lines were authenticated by vendors and routinely authenticated by visual inspection of cell morphology

Mycoplasma contamination

All used cell lines were routinely tested and confirmed negative for mycoplasma contamination.

Commonly misidentified lines
(See ICLAC register)

No commonly misidentified cell lines were used.

Plants

Seed stocks

Report on the source of all seed stocks or other plant material used. If applicable, state the seed stock centre and catalogue number. If plant specimens were collected from the field, describe the collection location, date and sampling procedures.

Novel plant genotypes

Describe the methods by which all novel plant genotypes were produced. This includes those generated by transgenic approaches, gene editing, chemical/radiation-based mutagenesis and hybridization. For transgenic lines, describe the transformation method, the number of independent lines analyzed and the generation upon which experiments were performed. For gene-edited lines, describe the editor used, the endogenous sequence targeted for editing, the targeting guide RNA sequence (if applicable) and how the editor was applied.

Authentication

Describe any authentication procedures for each seed stock used or novel genotype generated. Describe any experiments used to assess the effect of a mutation and, where applicable, how potential secondary effects (e.g. second site T-DNA insertions, mosiacism, off-target gene editing) were examined.
