# Supplementary material for: BromoCatch: a self-labelling tag platform for protein modification and live cell imaging
Source: Nat Commun. 2026 May 13;17:6406. doi: 10.1038/s41467-026-72539-w (PMC13376172; doi:10.1038/s41467-026-72539-w)
Supplement: Supplementary file 6 — Transparent Peer Review file [file 41467_2026_72539_MOESM6_ESM.pdf]

nature portfolio

Peer Review File

BromoCatch: a self-labelling tag platform for protein analysis and live cell imaging

Corresponding Author: Professor Alessio Ciulli

This file contains all reviewer reports in order by version, followed by all author rebuttals in order by version.

Attachments originally included by the reviewers as part of their assessment can be found at the end of this file.

Version 0:

Reviewer comments:

Reviewer #1

(Remarks to the Author)
This paper reports on a novel self-labeling tag, named BromoCatch, which is a variant of Brd4. The authors developed an orthogonal Brd4–covalent ligand pair using a bump-and-hole strategy combined with the introduction of a nucleophilic cysteine mutation, and successfully applied it for intracellular protein labeling. Expanding the repertoire of self-labeling tags is an important challenge in this field, and the authors' work is intriguing. However, most critically, it lacks direct comparison with existing self-labeling tag systems, quantitative characterization, and demonstration of compatibility for simultaneous use. As a result, readers are unable to properly evaluate the effectiveness of the authors' strategy. Additionally, the biological applications presented are rather conventional and lack academic novelty. In summary, this manuscript does not provide sufficient characterization of the strategy, and lacks both novelty and impact. Therefore, in its current form, it is not suitable for publication in Nature Communications. The authors should address the following points and consider resubmitting to a more specialized journal.
1. The authors should determine the second-order rate constant of the reaction and compare it with those of other tagging systems such as Halo-tag and SNAP-tag.
2. Acrylamide warheads are generally prone to non-specific reactions with intracellular proteins, and thus, the use of the probe at high concentrations is expected to generate substantial non-specific signals (ref. Cravatt et al, Nat Chem Biol. 2014 Sep;10(9):760-767.). The authors should present in-gel fluorescence images covering the full molecular weight range to evaluate the selectivity of the labeling. Additionally, a proteome-wide selectivity analysis using mass spectrometry should also be performed.
3. The authors should clarify whether BromoCatch is compatible with co-staining in combination with established self-labeling systems such as Halo-tag and SNAP-tag.
4. Demonstrating basic labeling capability alone is not sufficient for publication in a high-impact journal such as Nature Communications. The authors should clearly demonstrate how BromoCatch outperforms existing tagging systems in terms of performance or provides unique applications that cannot be achieved with current technologies.

Reviewer #2

(Remarks to the Author)
The manuscript by Rodriguez-Rios et al. describes the development of a novel Self-labelling protein (SLP) platform based on a small ~13 kDa bromodomain (BD) engineered with a nucleophilic cysteine for covalent ligand engagement (named Bromocatch). SAR studies using a series of electrophilic warheads on the reversible JQ1-derived inhibitor ET-JQ1-OMe revealed an acrylamide-bearing lead compound (MR116) that served as a basis for the development of biotin-based probe MR169 for biotin-based detection, and "always on" TMR probe MR202 for fluorescence-based detection of BromoCatch in cell lysates. Additionally, an environmental "switch-on" probe (C10852S) was developed, which features selective binding of a BromoCatch tagged protein in cells, and switch-on activation with minimal background fluorescence, demonstrating utility and suitability for live-cell imaging. The manuscript is well written, and the conclusions drawn from a plethora of biochemical, structural and live-cell data are sound. One drawback is that a number of figures in the main body and the supplementary data have been misassigned, requiring corrections in the text and/or renumbering of the figures.

Points to be addressed:

1. Figure 2G) “Covalent docking of MR116 (teal carbons) as predicted bound to Brd2-BD2(L383A,D434C) and 3D alignment with non-covalently bound Et-JQ1-OMe (grey carbon) in co-crystal structure 6YTM.”
The caption is misleading. It looks like covalent docking of MR116 into BRD4-BD2 (L387A/E438C) and overlay with BRD2-BD2(L383V) ET-JQ1-OMe (PDB 6YTM).

2. Page 8, line 207:” Importantly, the compounds did not covalently modify Brd4-BD2(WT) or Brd4-BD2(L387A), indicating high selectivity for the newly introduced cysteines.”
Please elaborate on how many cysteine residues exist in BRD4-BD2(WT) that could be subject to covalent attack. Also, since the scaffold is based on a pan-BET inhibitor (JQ1), was the binding potential of the lead compound MR116 tested against wildtype BD1 and BD2 of the other BET proteins?

3. Page 10, line 267: “… showed exquisite overlap with the reference crystal structure (Brd4-BD2(L383V)/ET-JQ1-OMe … …”
The reference crystal structure should read BRD2-BD2 (BD2(L383V)/ET-JQ1-OMe …

4. Page 11, line 292: “After a 10-minute incubation, the BRET signal was recorded (Figure 4A).”
I assume this should read Figure 6A.

5. Page 11, line 293”:”Among the tested ligands, MR116 exhibited superior intracellular potency, with an IC_{50} of 31 nM, significantly lower than the reversible ligand ET-JQ1-OMe (88 nM).”
The difference between 31 and 88 nM is not significant, i.e. delete “significant”. Also, a number of other warheads seemed to be working as good or better than MR116, particularly MR118 and MR112 (see point 7 below).

6. Page 11, line 306: “… no increase in BRET signal was observed for MR116 over 120 minutes (Figure 5B) …”.
This should read Figure 6B.

7. Page 9, line 247: “The acrylamide MR116 was selected as the specific complementary covalent ligand given the optimal specific covalent modification and superior stabilization observed with Brd4-BD2(L387A/E438C).”
Based on the DSF studies (Figure 4) and particularly the NanoBRET target engagement studies (Supplementary Figure 5, Supplementary Table 4) the chloroacetamides MR112 and MR118 show equal or better binding affinities for BRD4-BD2 than MR116. Why was MR116 chosen over MR112/MR118? Also, for the chloroacetamides the NanoBRET data suggest that both para and meta substituents are equally potent, while the acrylamide in meta (MR119) was significantly less active than the para substituent (MR116). What is a possible explanation for this discrepancy?

8. Page 12, line 330: “… we observed that only BromoCatch but not wild-type or L378A mutant (both lacking the nucleophilic cysteine) could be fluorescently labelled in vitro (Supplementary Figure 7).”
This should read Supplementary Figure 6.
I am having trouble seeing the differences as stated in the text. A fluorescence signal (smear) is seen for all proteins in the lanes upon labelling. Do the authors refer to the faint bands on top of the red smear for BromoCatch? If so, please indicate using an arrow for example.

9. Page 12, line 332: “To this end, we developed a biotin-functionalised bivalent probe (MR169, Fig 6A).
This should read Figure 7A.

10. Page 12, line 333: “The biotin probe maintained the high stabilising effect for BromoCatch (ΔTm of +20, Supplementary Figure 8) …”
This should read Supplementary Figure 7.

11. Page 12, line 341:”… even when the probe is at the highest concentrations (Figure 6C).”
This should read Figure 7C.

12. Page 13, line 364: “… based on the MR116 ligand (MR202, Figure 6D).”
This should read Figure 7D.

13. Page 13, line 368: ”… for which no binding was observed (Figure 6E).”
This should read Figure 7E.

14. Page 14, line 391: “… using Janelia Fluor® 635 (JF635) was prepared (Figure 7A).”
This should read Figure 8A

15. Page 15, line 399: “… while retaining the original linker (Figure 7B).”
This should read Figure 8B.

16. Page 16, line 414: “… despite the change of fluorophore (Figure 7C).”
This should read Figure 8C.

17. Page 16, line 424: “… or protein binding (Figure 7D,E, Supplementary Figure 10).”

Should read (Figure 8D, E, Supplementary Figure 8). Also note that the caption of Supplementary Figure 8 should read "Extended data from Figure 8" (not Figure 7).

18. Page 16, line 434: "… confirming its incompatibility with the molecular glue mechanism (Supplementary Figure 11, Supplementary Table 6)."
Should read Supplementary Figure 13 (or renumber current Supplementary Figures).

19. Page 17, line 457: "… of the bromodomain inhibitor ET-457 JQ1-OMe (Figure 9, Supplementary Figures 12-14).
Should read Supplementary Figures 10-12 (or renumber current Supplementary Figures).

20. Page 18, line 499: "additional crystallography data of the MR116/Brd3-BD2 L383A D434C co-crystal"
Should read MR116/Brd2-BD2.

Reviewer #3

(Remarks to the Author)
See file attached

Version 1:

Reviewer comments:

Reviewer #1

(Remarks to the Author)
With this revision, I believe that the overall quality of the manuscript has been substantially improved. In particular, the addition of data addressing this referee's main concern—kinetic analysis and multiplexing with HaloTag system—now enables readers to evaluate the utility of BromoCatch system both quantitatively and qualitatively. I consider the manuscript suitable for publication after the authors address the minor issues outlined below.
1.Supplementary Figure 6:
Panel D lacks a caption. In addition, some of the plots in panel B use identical colors, making them difficult to distinguish. Please use distinct colors and/or plot symbols for all datasets. The units of concentration should also be clearly indicated.
2.Supplementary Figure 11:
Please specify the units of concentration in the plots in panel C.
3.Figure 6C; Supplementary Figures 22, 23, and 25:
Several non-target bands are observed around ~10–15 kDa and ~65 kDa. What do these bands correspond to? Can they be assigned to ENO1, SURF6, or RBM14? If these bands potentially represent unidentified off-targets, this possibility should be explicitly mentioned in the main text or in the figure captions.
4.The authors conclude that ENO1, SURF6, and RBM14 are not off-targets because they are not detected in untransfected cell samples. However, this conclusion may appear to result from the use of an overly stringent p-value threshold (commonly p-value < 0.05, corresponding to $-\log10(\text{p-value}) \approx 1.3$, is used in proteomics research). In fact, several proteins in the untransfected cell samples meet the criteria of $\log2(FC) > 2$ and $p < 0.05$, indicating that off-target labeling is objectively present. Given that background labeling is expected when acrylamide is used as a warhead, I do not think it is necessary to strongly argue for the absence of off-targets. Instead, it would be more objective and scientifically appropriate to acknowledge the presence of potential off-targets and discuss that, nevertheless, the system remains suitable for imaging applications in overexpression settings. At a minimum, the current statements claiming the absence of off-targets should be toned down and rephrased accordingly.
5.Page 16, line 486:
"JF63" should be corrected to "JF635".
6.Figure 9:
To more clearly demonstrate the orthogonality between BromoCatch and HaloTag, the following control experiments should be included:
(i) treatment of cells expressing H2B–HaloTag alone with C10852L;
(ii) treatment of cells expressing H2B–BromoCatch alone with a Haloalkane probe.

Reviewer #2

(Remarks to the Author)
The authors have satisfactorily addressed the reviewers' comments, and the revised manuscript represents a significant improvement.

Reviewer #3

(Remarks to the Author)
Comments attached in formatted PDF

Version 2:

Reviewer comments:

Reviewer #1

(Remarks to the Author)
The authors have appropriately addressed the comments raised by this referee. I have no further comments.

Reviewer #3

(Remarks to the Author)
I thank the authors for clarifying all my questions. The manuscript is now suitable for publication (see one minor addition below).

Multiplexing: I understand that the experimental effort is too high to provide a triple multiplexing experiment. I would however like to encourage the authors to perform such experiments in a follow-up study as BromoCatch could indeed expand the family of SLP tools. Currently, only SNAP and HaloTag are used routinely and covalent TMP-tag and CLIP have not found widespread use. It might be possible that BromoCatch can indeed fill this niche.

Colocalization Analysis: We thank the authors a lot for including colocalization analysis as well as the analysis of cytoplasmic/nuclear ratios. These are common measurements performed in the imaging community and will greatly strengthen the impact of the paper.
With regard to the co-localization analysis, I would like to highlight one aspect. Given the authors conducted this analysis on transiently transfected cells the resulting R values are rather low (0.2/0.3). This mostly reflects the fact that all cells show Hoechst signal but only the Halo expressing cells show far-red signal. Hence R greatly underestimates the “true” colocalization. Ideally the analysis would exclude the non-transfected cells. However, as the authors compare R values among differently stained conditions but similarly transfected cells, this is ok. To avoid any confusion by readers, I would include a statement mentioning that non-transfected cells were not excluded for analysis.

Open Access This Peer Review File is licensed under a Creative Commons Attribution 4.0 International License, which permits use, sharing, adaptation, distribution and reproduction in any medium or format, as long as you give appropriate credit to the original author(s) and the source, provide a link to the Creative Commons license, and indicate if changes were made.
In cases where reviewers are anonymous, credit should be given to 'Anonymous Referee' and the source.
The images or other third party material in this Peer Review File are included in the article’s Creative Commons license, unless indicated otherwise in a credit line to the material. If material is not included in the article’s Creative Commons license and your intended use is not permitted by statutory regulation or exceeds the permitted use, you will need to obtain permission directly from the copyright holder.
To view a copy of this license, visit https://creativecommons.org/licenses/by/4.0/

We sincerely thank the reviewers for the insightful comments and comprehensive feedback. We are confident that these suggestions have significantly strengthened the quality of our manuscript, and we hope the additional data provided fully addresses all points raised.

Based on the reviewers' input, we focused on four key areas of improvement, each supported by new experiment.

- **Kinetics of the BromoCatch system.** The initial evidence of the fast kinetics of the BromoCatch system observed in our INTACT MS experiments was confirmed and expanded by a more comprehensive study of the kinetics by using fluorescence polarization assay to determine the apparent second order constant of the monovalent ligand MR116 and bivalent probes MR202 and MR169. We observed comparable kinetics to stablished systems such as HaloTag and SNAP-tag. (**new data now included in new Figures 5D-E, 6E and 7D-E-F**)

- **Specificity of our probes.** The evidence on specificity we had provided based on the western blotting and imaging with the bivalent probes was complemented with a proteomics pull down experiment to demonstrate the specificity of our BromoCatch biotin probe MR169 (**new Figure 6E**).

- **Fluorogenicity of Janelia Fluor probes.** We have found that shortening the linker significantly improved the fluorogenic character of the probes (see more info in response below). We have now included an improved version of the probe, C10852L, that contains a shorter linker compared to the previous C10852S compound and demonstrated substantial improvement in fluorogenic response upon labelling BromoCatch in vitro on both recombinant and cellular protein. We are currently developing a library of probes where the linker on this fluorogenic probes is modified.

- **Benchmarking and multiplexing.** We agree that benchmarking against other self-labeling protein (SLP) systems is important. Here, we selected HaloTag due to its widespread use and optimal properties. While BromoCatch does not outperform HaloTag, our data show that it is comparably en par, fully compatible, and enables multiplexing with additional tags such as HaloTag (**new data now included in new Figures 8E-F-G and 9**). Importantly, BromoCatch offers unique compatibility with the catalytic PROTAC degrader AGB1-an advantage not available in current SLP platforms (**Figure 5H**).

A detailed point by point response can be found below.

REVIEWER COMMENTS

Reviewer #1 (Remarks to the Author):

This paper reports on a novel self-labeling tag, named BromoCatch, which is a variant of Brd4. The authors developed an orthogonal Brd4–covalent ligand pair using a bump-and-hole strategy combined with the introduction of a nucleophilic cysteine mutation and successfully applied it for intracellular protein labeling. Expanding the repertoire of self-labeling tags is an important challenge in this field, and the authors' work is intriguing. However, most critically, it lacks direct comparison with existing self-labeling tag systems, quantitative characterization, and demonstration of compatibility for simultaneous use. As a result, readers are unable to properly evaluate the effectiveness of the authors' strategy. Additionally, the biological applications presented are rather conventional and lack academic novelty. In summary, this manuscript does not provide sufficient characterization of the strategy, and lacks both novelty and impact. Therefore, in its current form, it is not suitable for publication in Nature Communications. The authors should address the following points and consider resubmitting to a more specialized journal:

We appreciate the reviewer's insightful and constructive feedback. We have carefully considered their suggestions and, through extensive revisions, have addressed all key points raised with new data wherever possible, given the time constraints. We believe the revised manuscript is significantly improved and strengthens the qualification of BromoCatch as a valuable addition to the arsenal of self-labeling tag tools available to researchers.

Thank you once again for your valuable feedback, which has contributed to the overall improvement of our manuscript.

1. The authors should determine the second-order rate constant of the reaction and compare it with those of other tagging systems such as Halo-tag and SNAP-tag.

We concur and have now determined the apparent second-order kinetics of the monovalent ligand MR116 and the bivalent probes MR169 and MR202 using two complementary fluorescence polarization (FP) assay setups.

For MR116 and MR169, we employed a competition-based FP assay adapted from a previously reported protocol in our laboratory (Ramachandran et al. *Nat Commun.* 2023, ref. 49). The anisotropy data were fitted to a pseudo–first-order model, assuming rapid reversible equilibria that reached completion within the first 30 seconds of the reaction (the time of the initial read). The resulting rate constants indicate that both the monovalent ligand and bivalent probes exhibit kinetics comparable to those reported for HaloTag and SNAP-tag systems (**new data included in Figures 5D-E, 6E, and in the Supplementary Information**).

For MR202, we utilized a direct-binding FP assay without a competitor, following a protocol adapted from Wilhelm et al. *Biochemistry* 2021 (ref. 26). This approach enabled us to quantify the interaction kinetics

under simplified conditions (**new data included in Figures 5D-E, 6E, and in the Supplementary Information**).

Determining precise kinetic parameters for the fluorogenic probes C10852S and C10852L proved challenging due to the additional complexity from their environment-sensitive activation. Therefore, for these probes, we conducted time-dependent activation studies to characterize their switch-on profiles instead of calculating rate constants (**new data included in Figures 8E-F-G**).

2. Acrylamide warheads are generally prone to non-specific reactions with intracellular proteins, and thus, the use of the probe at high concentrations is expected to generate substantial non-specific signals (ref. Cravatt et al, Nat Chem Biol. 2014 Sep;10(9):760-767.). The authors should present in-gel fluorescence images covering the full molecular weight range to evaluate the selectivity of the labeling. Additionally, a proteome-wide selectivity analysis using mass spectrometry should also be performed.

Thank you for raising this important point. Our western blot analysis using the acrylamide bivalent probes has already shown a lack of significant off-target effects, with full membrane images available in **Supplementary Figures 21-22**. We tested two cell lines with different probes and observed no significant nonspecific signals indicating non-specific labeling.

Additionally, we conducted a proteomics analysis using a GFP-BromoCatch cell line, followed by a streptavidin pull-down. This data has been added to main manuscript **Figure 6E**. The results demonstrate: i) the specificity of the biotin probe MR169 for BromoCatch, with no significant protein enrichment in wild-type cells compared to DMSO treatment; ii) successful pull-down of our BromoCatch-GFP protein; and iii) that pretreatment with excess ET-JQ1-OMe prevented the pull-down of BromoCatch-GFP.

3. The authors should clarify whether BromoCatch is compatible with co-staining in combination with established self-labeling systems such as Halo-tag and SNAP-tag.

In response, we performed live-cell confocal imaging multiplexing experiments to assess the compatibility of BromoCatch with established self-labeling systems. HEK293 cells were co-transfected with GFP-BromoCatch and H2B-HaloTag, followed by a simultaneous treatment for 2 hours with C10852L (our leading Janelia fluorescent probe for BromoCatch) and the HaloTag JF549 probe. Counterstaining with Hoechst 33342, along with intrinsic GFP fluorescence, allowed us to independently verify the subcellular localization and probe “switch-on” for each system. As expected, H2B-HaloTag localized to the nucleus and co-stained with Hoechst, while GFP-BromoCatch exhibited clear colocalization with its corresponding C10852L signal. These findings demonstrate that BromoCatch and HaloTag function orthogonally and can be imaged simultaneously without detectable interference, confirming BromoCatch’s compatibility with established self-labeling protein systems for multiplexed live-cell imaging (**Figure 9**).

4. Demonstrating basic labeling capability alone is not sufficient for publication in a high-impact journal such as Nature Communications. The authors should clearly demonstrate how BromoCatch outperforms

existing tagging systems in terms of performance or provides unique applications that cannot be achieved with current technologies.

We appreciate this important point and emphasize that there is a growing need for new self-labeling protein (SLP) systems to enhance the toolbox for researchers. We believe BromoCatch is a powerful and complementary addition to existing platforms such as HaloTag and SNAP-tag.

A key differentiator of BromoCatch is its unique compatibility with small-molecule BromoTag degraders like AGB1, which we here show enables highly efficient and catalytic degradation also of BromoCatch. This capability is unprecedented among current platforms; to our knowledge, both SNAP PROTACs and HaloPROTACs rely on covalent mechanisms, and no catalytic degraders have been developed thus far. By introducing a system that supports catalytic degradation, we pave the way for new strategies and dynamic control of protein levels—applications that cannot be achieved with existing technologies.

The versatility and compatibility of BromoCatch are expected to be highly valuable for future multiplexing and induced-proximity approaches, where two orthogonal tags are used to bring two or more proteins together. This is particularly relevant for researchers seeking innovative tools for functional studies and therapeutic development. We are actively working on demonstrating the advantages of this utility in ongoing studies, which will be detailed in future publications.

Reviewer #2 (Remarks to the Author):

The manuscript by Rodriguez-Rios et al. describes the development of a novel Self-labelling protein (SLP) platform based on a small ~13 kDa bromodomain (BD) engineered with a nucleophilic cysteine for covalent ligand engagement (named Bromocatch). SAR studies using a series of electrophilic warheads on the reversible JQ1-derived inhibitor ET-JQ1-OMe revealed an acrylamide-bearing lead compound (MR116) that served as a basis for the development of biotin-based probe MR169 for biotin-based detection, and "always on" TMR probe MR202 for fluorescence-based detection of BromoCatch in cell lysates. Additionally, an environmental "switch-on" probe (C10852S) was developed, which features selective binding of a BromoCatch tagged protein in cells, and switch-on activation with minimal background fluorescence, demonstrating utility and suitability for live-cell imaging. The manuscript is well written, and the conclusions drawn from a plethora of biochemical, structural and live-cell data are sound.

We appreciate the reviewers for their careful reading of our manuscript and for capturing its salient features. Thank you for your vote of support!

One drawback is that a number of figures in the main body and the supplementary data have been misassigned, requiring corrections in the text and/or renumbering of the figures.

We appreciate you pointing out this issue. We have carefully checked all figures throughout the revised manuscript and reconciled the numbering accordingly.

Points to be addressed:

1. Figure 2G) "Covalent docking of MR116 (teal carbons) as predicted bound to Brd2-BD2(L383A,D434C) and 3D alignment with non-covalently bound Et-JQ1-OMe (grey carbon) in co-crystal structure 6YTM." The caption is misleading. It looks like covalent docking of MR116 into BRD4-BD2 (L387A/E438C) and overlay with BRD2-BD2(L383V) ET-JQ1-OMe (PDB 6YTM).

Thank you for the comment. This has now been rephrased to: "*Covalent docking of MR116 (teal carbons) bound to Brd2-BD2^{L383A,D434C} and 3D alignment with reversibly bound ET-JQ1-OMe (grey) in complex with Brd2-BD2^{L383V} in the original co-crystal 6YTM.*"

2. Page 8, line 207:" Importantly, the compounds did not covalently modify Brd4-BD2(WT) or Brd4-BD2(L387A), indicating high selectivity for the newly introduced cysteines."
Please elaborate on how many cysteine residues exist in BRD4-BD2(WT) that could be subject to covalent attack. Also, since the scaffold is based on a pan-BET inhibitor (JQ1), was the binding potential of the lead compound MR116 tested against wildtype BD1 and BD2 of the other BET proteins?

Thank you for this insightful comment. We have clarified the number and location of cysteine residues in BRD4-BD2(WT) and addressed the potential for off-target covalent engagement across BET family members.

The revised sentence reads:

Page 7, line 203: *"Importantly, no covalent adduct formation was detected for Brd4-BD2^{WT} or Brd4-BD2^{L387A}, confirming high selectivity for the engineered cysteines over other cysteine residues present within the tag protein (Cys356, Cys391 or Cys429)."*

Regarding orthogonality, while BET bromodomains share high sequence homology, there are no cysteine residues present in or near the ligand-binding pockets of Brd2 or Brd3 that could cause nonspecific covalent engagement with the ligand. This design principle is supported by our prior bump-and-hole ligand design. The wild-type Brd4-BD2 model serves as a representative scaffold for the BET family, and the engineered cysteine (E438C) is unique to our BromoCatch sequence, ensuring orthogonality with any wild-type BET bromodomain proteins.

Finally, the results from the proteomics pull-down assay, using the biotin-functionalized probe MR169, confirm that no BET proteins were enriched in wild-type cells compared to DMSO controls, further demonstrating the specificity and orthogonality of our system.

3. Page 10, line 267: "… showed exquisite overlap with the reference crystal structure (Brd4-BD2(L383V)/ET-JQ1-OMe … …"The reference crystal structure should read BRD2-BD2 (BD2(L383V)/ET-JQ1-OMe …
This has now been corrected.

4. Page 11, line 292: "After a 10-minute incubation, the BRET signal was recorded (Figure 4A)." I assume this should read Figure 6A.
This has now been corrected.

5. Page 11, line 293":"Among the tested ligands, MR116 exhibited superior intracellular potency, with an IC_{50} of 31 nM, significantly lower than the reversible ligand ET-JQ1-OMe (88 nM)." The difference between 31 and 88 nM is not significant, i.e. delete "significant". Also, a number of other warheads seemed to be working as good or better than MR116, particularly MR118 and MR112 (see point 7 below).

Although MR118 and MR112 (chloroacetamides) exhibited strong reactivity and enhanced NanoBRET potency, as confirmed by INTACT MS, we ultimately deprioritized these warheads despite their promising performance. In our experience, both compounds showed reduced stability during storage, raising concerns about their shelf life and reproducibility. Mechanistically, chloroacetamides are generally associated with lower selectivity toward target residues, increased susceptibility to hydrolysis, and the generation of chlorine-containing byproducts, which could compromise probe robustness and cellular compatibility.

To further evaluate intrinsic thiol reactivity, we conducted a glutathione (GSH) assay (**new data included in new Supplementary Table 4**), although this was not explicitly requested by the reviewers. Interestingly, the assay indicated comparable GSH half-lives for the para-acrylamide and para-chloroacetamide analogues, suggesting that our aromatic acrylamide is more electrophilic than typical acrylamides—likely due to electronic effects from the aromatic substituent. This finding implies that, in terms of intrinsic reactivity, both warheads could be similarly prone to thiol quenching in a high-GSH environment. However, GSH assays only serve as a proxy for intrinsic reactivity and do not directly measure proteome-wide selectivity.

Despite this similarity, we prioritized the para-acrylamide scaffold for downstream development due to its greater chemical stability, reduced risk of hydrolysis, and strong precedent in medicinal chemistry for selective covalent probe design. Additionally, MR119 (meta-acrylamide) showed a longer GSH half-life but exhibited lower stabilization in differential scanning fluorimetry (DSF) assays and reduced potency in cellular assays, further reinforcing our decision to advance MR116 as the lead compound.

To capture these important points, have included the following sentence in the main text:
"Although the chloroacetamides MR112 and MR118 demonstrated a slight improvement in potency, we ultimately prioritized the acrylamide scaffold because of its milder intrinsic reactivity. This choice was intended to minimize off-target covalent interactions and to enhance stability in cellulo, thereby providing a more selective and robust chemical probe"

6. Page 11, line 306: "… no increase in BRET signal was observed for MR116 over 120 minutes (Figure 5B) …".This should read Figure 6B.
This has now been corrected.

7. Page 9, line 247: "The acrylamide MR116 was selected as the specific complementary covalent ligand given the optimal specific covalent modification and superior stabilization observed with Brd4-BD2(L387A/E438C)." *Based on the DSF studies (Figure 4) and particularly the NanoBRET target engagement studies (Supplementary Figure 5, Supplementary Table 4) the chloroacetamides MR112 and MR118 show equal or better binding affinities for BRD4-BD2 than MR116. Why was MR116 chosen over MR112/MR118? Also, for the chloroacetamides the NanoBRET data suggest that both para and meta substituents are equally potent, while the acrylamide in meta (MR119) was significantly less active than the para substituent (MR116). What is a possible explanation for this discrepancy?*

The chloroacetamide warheads (MR112 and MR118) are slightly smaller, containing one fewer carbon atom than the acrylamide analogue. We hypothesized that the meta-acrylamide may be better accommodated within the BromoCatch binding pocket due to its reduced steric bulk and greater conformational flexibility, which allows it to accommodate the bulkier substituent more effectively. This hypothesis is supported by our docking studies, which indicated favorable positioning of the meta-chloroacetamide relative to the meta-acrylamide.

Although the chloroacetamides demonstrated a slight improvement in potency, we ultimately prioritized the acrylamide scaffold because of its milder intrinsic reactivity. This choice was made to minimize off-target covalent interactions and enhance stability in cellular environments, providing a more selective and robust chemical probe.

We have added a relevant sentence to the manuscript:
"Although the chloroacetamides MR112 and MR118 demonstrated a slight improvement in potency, we ultimately prioritized the acrylamide scaffold because of its milder intrinsic reactivity. This choice was intended to minimize off-target covalent interactions and to enhance stability in cellulo, thereby providing a more selective and robust chemical probe"

8. Page 12, line 330: "… we observed that only BromoCatch but not wild-type or L378A mutant (both lacking the nucleophilic cysteine) could be fluorescently labelled in vitro (Supplementary Figure 7)." This should read Supplementary Figure 6.I am having trouble seeing the differences as stated in the text. A fluorescence signal (smear) is seen for all proteins in the lanes upon labelling. Do the authors refer to the faint bands on top of the red smear for BromoCatch? If so, please indicate using an arrow for example.

The smear comes from the Cy5 azide sticking around in the gel. For clarity, we have repeated the experiment and now show the cleaner gel.

9. Page 12, line 332: "To this end, we developed a biotin-functionalised bivalent probe (MR169, Fig 6A). This should read Figure 7A.
Corrected

10. Page 12, line 333: "The biotin probe maintained the high stabilising effect for BromoCatch (ΔTm of +20, Supplementary Figure 8) …" This should read Supplementary Figure 7.
Corrected

11. Page 12, line 341:"… even when the probe is at the highest concentrations (Figure 6C)." This should read Figure 7C.
Corrected

12. Page 13, line 364: "… based on the MR116 ligand (MR202, Figure 6D)." This should read Figure 7D.
Corrected

13. Page 13, line 368: "… for which no binding was observed (Figure 6E)." This should read Figure 7E.
Corrected

14. Page 14, line 391: "… using Janelia Fluor® 635 (JF635) was prepared (Figure 7A)." This should read Figure 8A.
Corrected

15. Page 15, line 399: "… while retaining the original linker (Figure 7B)." This should read Figure 8B.
Corrected

16. Page 16, line 414: "… despite the change of fluorophore (Figure 7C)."This should read Figure 8C.
Corrected

17. Page 16, line 424: "… or protein binding (Figure 7D,E, Supplementary Figure 10)." Should read (Figure 8D, E, Supplementary Figure 8). Also note that the caption of Supplementary Figure 8 should read "Extended data from Figure 8" (not Figure 7).
Corrected

18. Page 16, line 434: "… confirming its incompatibility with the molecular glue mechanism (Supplementary Figure 11, Supplementary Table 6)." Should read Supplementary Figure 13 (or renumber current Supplementary Figures).
Corrected

19. Page 17, line 457: "… of the bromodomain inhibitor ET-457 JQ1-OMe (Figure 9, Supplementary Figures 12-14). Should read Supplementary Figures 10-12 (or renumber current Supplementary Figures).
Corrected

20. Page 18, line 499: "additional crystallography data of the MR116/Brd3-BD2 L383A D434C co-crystal" Should read MR116/Brd2-BD2.
Corrected

Reviewer #3 (Remarks to the Author):

Revisions "BromoCatch: a self-labelling tag platform for protein analysis and live cell imaging"

Overall comments

Rodriguez-Rios et al. report on the generation of a new self-labeling protein tag system derived from Brd4-BD2 called BromoCatch. Supported by computational docking experiments they generate protein variants carrying reactive cysteines as well as a panel of JQ1 derived molecules functionalized with electrophilic warheads. They synthesize and test a subset of 14 small molecules for their reactivity towards two protein variants using ESI-MS, DSF, and NanoBRET assays. They finally characterize one ligand in depth via competition assays as well as an x-ray crystal structure. They then further functionalize this ligand with different handles including alkynes (click-chemistry), VHL (E3 recruiter), fluorophores (TMR, JF635) and characterize the functionality of these molecules primarily via in vitro protein labeling, in gel fluorescence and western blots. They finally demonstrate the use of the JF635 analogue in confocal microscopy experiments of H2B fused BromoCatch.

The authors follow a well-conceived rational route to generate the BromoCatch system, which is an attractive new addition to the SLP family due to its small size (13kDa vs. 18 kDa eDHFR, 19.4 kDa SNAP-tag, 33 kDa HaloTag). The molecules are well characterized in vitro and the paper well written. The data is nicely presented, and the in vitro experiments were very rigorously performed. However, some of the western-blot data (see point 2 below) and microscopy data require improvement, need to be more thoroughly described and/or are not sufficiently analyzed complicating the interpretation of the data. The methods provided are extensive but require more detail in the microscopy section.

We appreciate the reviewer for their thoughtful and comprehensive evaluation of our manuscript. Thank you for recognizing the significance of BromoCatch as a promising addition to the self-labeling protein tag family. We are also grateful for your constructive comments regarding the characterization and analysis of our data. We have carefully considered your suggestions and have addressed all key points raised in subsequent individual responses. We believe these revisions enhance the robustness of our data and improve the clarity of our findings.

Major concerns

My major concern with the presented paper is that their system is not benchmarked in any way to other self-labeling protein tags. The authors nicely outline the shortcomings of other tags in their introduction: size (Halo), slow labeling kinetics (SNAP), fluorogenicity (SNAP), and cell permeability of the ligand (SNAP). They further state that they "aimed at addressing the limitation of current SLPs to expand the toolbox with an orthogonal platform for multiplexing". However, neither the orthogonality nor the overcoming of the limitations is clearly demonstrated:

- What are the labeling kinetics of their system (in vitro, point 1 below).

As per response to Reviewer 1's point 1:

We have now determined the apparent second-order kinetics of the monovalent ligand MR116 and the bivalent probes MR169 and MR202 using two complementary fluorescence polarization (FP) assay setups.

For MR116 and MR169, we employed a competition-based FP assay adapted from a previously reported protocol in our laboratory (Ramachandran et al. *Nat Commun.* 2023, ref. 49). The anisotropy data were fitted to a pseudo–first-order model, assuming rapid reversible equilibria that reached completion within the first 30 seconds of the reaction (the time of the initial read). The resulting rate constants indicate that both the monovalent ligand and bivalent probes exhibit kinetics comparable to those reported for HaloTag and SNAP-tag systems (**new data included in Figures 5D-E, 6E, and in the Supplementary Information**).

For MR202, we utilized a direct-binding FP assay without a competitor, following a protocol adapted from Wilhelm et al. *Biochemistry* 2021 (ref. 26). This approach enabled us to quantify the interaction kinetics under simplified conditions (**new data included in Figures 5D-E, 6E, and in the Supplementary Information**).

Determining precise kinetic parameters for the fluorogenic probes C10852S and C10852L proved challenging due to the additional complexity from their environment-sensitive activation. Therefore, for these probes, we conducted time-dependent activation studies to characterize their switch-on profiles instead of calculating rate constants (**new data included in Figures 8E-F-G**).

- How does cell permeability of their molecules compare to the one of SNAP-tag substrates? E.g. labeling in cells at identical concentrations with similar protein fusions (see point 1 below).

We chose the HaloTag system for benchmarking and multiplexing due to the availability of a wide range of probes. We developed a stable H2B-HaloTag expressing cell line in parallel with a stable H2B-BromoCatch cell line. Comparative labeling efficiency using the corresponding TMR probes was performed under identical conditions and analyzed by western blot. Both probes effectively labeled their respective constructs. Notably, the HaloTag TMR probe demonstrated the ability to label the target at lower concentrations, suggesting improved permeability of the HaloTag TMR ligand.

We recognize the importance of studying cell permeability in greater depth. A comprehensive evaluation of the cell permeability of our library of probes is underway. We are currently developing in cellulo target engagement assays using NanoLuc-BromoCatch for NanoBRET assays in both permeabilized and non-permeabilized conditions to determine the availability index. However, further optimization of this protocol is required, and we anticipate publishing these findings soon.

- How does fluorogenicity of this tag compare to SNAP-tag and HaloTag (from a first glance of Figure 8E: 4x not very favorably, point 4 and 6.

Thank you for highlighting this important point. At the time of manuscript submission, we were developing a series of different fluorogenic probes exploring structure-activity relationships (SAR) in the

linker. The fluorogenicity of Janelia Fluor is heavily dependent on the fluorophore's binding to the protein surface to effectively switch on. With this in mind, we shortened the linker length to two aliphatic carbons to enhance the proximity of the fluorophore to the protein upon binding. This probe, designated C10852L, has now been included in the manuscript and demonstrates an improvement compared to the previously disclosed C10852S probe.

To our knowledge, there is currently no commercially available Janelia Fluor 635 SNAP-tag probe for benchmarking our system, so we focused our comparisons on the HaloTag-JF635 haloalkane, using the same fluorophore as in our C10852 series. The benefit of shortening the linker is evident in the time-dependent activation experiments as well as the excitation and emission profiles shown in **Figure 8**.

We also compared the performance of our probes to that of recombinant GST-HaloTag in combination with the Janelia Fluor 635 haloalkane probe. While GST-HaloTag demonstrated superior performance compared to our C10852L-BromoCatch system, we observed a good signal-to-noise ratio with our probe, enabling its use in cellular applications. We continue to explore linker SAR to further improve the fluorogenicity of our compounds and are expanding our library of fluorophores for fluorogenic probe development.

- Does the smaller size allow to tag any of the proteins mentioned in the introduction (AGO2, COX8A, ATP5ME)?

We are currently developing relevant CRISPR-engineered cell lines and have made progress with AGO2. However, the development and validation of these targets are beyond the scope of this publication. Therefore, we have removed the original statement regarding these targets and included them as part of our future and ongoing work. For the purposes of this study, we selected GFP as an additional target due to its practical advantages for validation.

- Lastly, can the authors showcase a co-imaging experiment with one, two or even three of the other tags (HaloTag, SNAP-tag, eDHFR)?

As per response to Reviewer 1’s point 3:

In response, we performed live-cell confocal imaging multiplexing experiments to assess the compatibility of BromoCatch with established self-labeling systems. HEK293 cells were co-transfected with GFP-BromoCatch and H2B-HaloTag, followed by a simultaneous treatment for 2 hours with C10852L (our leading Janelia fluorescent probe for BromoCatch) and the HaloTag JF549 probe. Counterstaining with Hoechst 33342, along with intrinsic GFP fluorescence, allowed us to independently verify the subcellular localization and probe “switch-on” for each system. As expected, H2B-HaloTag localized to the nucleus and co-stained with Hoechst, while GFP-BromoCatch exhibited clear colocalization with its corresponding C10852L signal. These findings demonstrate that BromoCatch and HaloTag function orthogonally and can be imaged simultaneously without detectable interference, confirming BromoCatch’s compatibility with established self-labeling protein systems for multiplexed live-cell imaging (**Figure 9**).

If the authors could provide the above-mentioned data and thereby clearly benchmark BromoCatch over existing tags or show its suitability in advanced multiplexing experiments, the manuscript would greatly gain in impact.

We recognize the importance of benchmarking BromoCatch against existing tags and demonstrating its suitability for multiplexing experiments. To this end, we have included extensive additional data that address these points, thereby enhancing the impact of our findings and guiding ongoing and future research efforts for ourselves and many other researchers in the field.

Detailed comments on the manuscript's content Results and further experiments

1) An important parameter of SLP's is their labeling kinetics e.g. see reference 18. The authors state that kinetics are fast (e.g. line 297) but no experiments were performed for precise quantification. To be able to compare the performance of the BromoCatch with other systems it would be imperial to measure the labeling kinetics in vitro and maybe in cellulo. This should be added.

We have measured the pseudo-first-order kinetics of the MR116 ligand and the probes MR169 and MR202 to determine their rate constants (k_{app}). The kinetics for both the monovalent ligand and the bivalent probes correlate with the kinetics reported for HaloTag and SNAP-tag ligands in the literature. The new data for MR116 is included in **Figure 5** and **Supplementary Figure 6**. Kinetics for MR169 are presented in **Figure 6** and **Supplementary Figure 11**.

We are currently working on the kinetics of the fluorogenic probes C10852S and C10852L. However, quantifying their kinetics is challenging due to the additional complexity introduced by their fluorogenic character. For these probes, we have opted to perform time-dependent activation studies to illustrate the activation profile and switch-on character for this publication, with plans to evaluate their kinetics by INTACT LC-MS in future studies. The activation profiles for both fluorogenic probes have been incorporated into **Figure 9** of the manuscript.

2) Blots and Gels

a. CuAAC experiments SI Fig 6C: Why is there a huge Cy5 streak in both the WT and L387A samples? Please clarify.

The smear comes from the Cy5 azide sticking around in the gel. For clarity, we have repeated the experiment and have now shown the cleaner gel. This is now reflected in **Supplementary Figure 9**.

b. Biotin Blot MR169, Figure 7C. The blots are labeled inadequately. It is difficult to understand which antibodies and fluorophores were used. Please adapt the SI Figure and/or the Methods section. Also, the cells used are not indicated in the SI Figure. What are all the additional bands that are labeled in the "presumably" anti-strep blot (labeled H2B-Bromo-Catch)? Please clarify in the

Figures and legends have now been updated in the Supp. Information document.

c. Line 368 (Fig 7F): "exquisite selectivity" I think this statement is misleading as any non- covalently bound dye will be "washed" out on the protein gel. I don't think that this experiment allows any conclusions on selectivity but only shows which proteins form a covalent bond and which not. Please adapt the text.

As above, we have added the sentence: *"When the probe was titrated at increasing concentrations against the protein, maximal fluorescence intensity was achieved at stoichiometric levels of protein to probe (10 µM), selectively over Brd4-BD2 and Brd4-BD2^{L387A} (**Figure 7B**)…"*

d. Similarly (line 421): "SDS gel analysis showed maximal fluorescence at stoichiometric protein-to probe levels (10 µM), after which the signal plateaued despite increasing C10852S concentration." Again, residual probe that is not bound to protein will just be washed out and hence this is not surprising. Please revise the text.

We have revised the sentence as follows: *"This feature allowed us to evaluate the concentration dependent increase in fluorescence 'in-gel', which presented the same concentration dependent response to that of MR202. These results confirm the covalent binding to BromoCatch is maintained despite the change of fluorophore…."*

e. Figure 7F and SI Figure: Tubulin and MR202 were visualized with a spectrally identical fluorophore TMR and Alexa546. To tell which one is which, tubulin should be visualized separately. Please provide such an experiment.

The gel and the membranes were imaged just after gel transfer are always imaged prior any co-staining with the tubulin antibody. The additional membrane image was added (**Supplementary Figure 21-22**). The additional membrane/gel has been added to the SI.

3) Can the authors please speculate why MR170 does not lead to >90% degradation? Please add this to the PROTAC section.

To speculate the reason for the observed behavior, we have included the following sentence: *"This result is consistent with the known disadvantage of PROTACs that are covalent at their target protein, such as HaloPROTACs,* [52–54] *due to their loss of the catalytic, substoichiometric mode of action.*[55]*"*

4) The fluorogenicity of the generated system should be characterized further and be quantified by fluorescence excitation and emission (e.g. Figure 8E). Similarly, to show advantages over other SLP's this should be characterized in cells (see below). Please provide these additional measurements.

We have characterized the excitation and emission spectra of the system using a fluorimeter and included a comparison with the equivalent HaloTag system. Additionally, time-dependent fluorogenic activation was evaluated by monitoring the fluorescence intensity changes over time for the probes C10852S and C10852L (**Figure 8**), as described in the new sentence:

*"…To compare fluorogenic performance, activation of both probes was assessed in vitro at low probe concentrations to better resolve differences in signal. Both probes resulted in an increase in fluorescence intensity when the BromoCatch protein was present but C10852L demonstrated an improvement in signal-to-noise when compared to C10852S probe, likely due to the shorter linker (**Figure 8E**). The excitation and emission spectra of the probes (C10852L and C10852S, 1 µM) was measured in presence and absence of BromoCatch or Brd4BD2^{WT} (**Figure 8F**). Both probes activation by an excess BromoCatch (10 µM) resulted in an increment in the fluorescence intensity emitted across the expected emission spectrum, that displayed a maximum λ_{em} of at 660 nm. A similar trend was observed across the excitation wavelengths of the JF63 with a maximum λ_{ex} at 640 nm upon activation. Importantly, incubation with Brd4BD2^{WT} showed comparable fluorescence emission to that in buffer. The observed spectra are consistent with the reported JF635 fluorescence spectrum and comparable to that of the HaloTag equivalent system (JF635 haloalkane/GST-HaloTag, **Figure 8G**). C10852L gave a 27-fold increase in fluorescence emission, while C10852S was limited to 7-fold. The JF635 haloalkane switch on showed the greatest increase in the presence of HaloTag (98-fold). Together the data support enhanced fluorogenicity of the shorter-linked C10852L probe…"*

Further comparisons with the HaloTag system were performed in cellular imaging experiments (**Figure 9**).

5) Figure 8D: I cannot find any information on the experimental details of Figure 8D. Please add this information to the methods section. Importantly, which protein concentration was used?

This information has now been added to the relevant Supp Info section:

"Fluorogenicity of C10852S and C10852L probes" For quantification measurements to evaluate the fluorogenic character of probes C10852S or C10852L, Brd4-BD2, Brd4-BD2L387A, Brd4-BD2L387A,E438C (50 µL, 10 µM) in activity buffer were co-incubated for 2 hours with DMSO (1 µL) or in presence of increasing concentrations of the probe (1 µL) to give final concentrations of the probe ranging from 2.5 to 50 µM. The probe (2.5 to 50 µM) in 0.1% TFA in ethanol was used as a positive control of fully switched on C10852S. Samples (10 µL) were plated in triplicate in a CORNING® black 384-well low volume plate and relative fluorescence units (RFU) measured using a GloMax Promega microplate reader (JF635 channel λex: 625nm λem: 660–725 nm"

a. Furthermore, the authors state: "However, fluorescence levels were lower compared to the covalent system, suggesting covalency enhances fluorogenicity" As the probe likely has different affinity for the

two proteins this statement is not correct in my eyes. Please revise or clarify how you come to this conclusion.

Our statement is based on the observation from the DSF assays (**Figure 3**), which demonstrated a comparable stabilization effect of the acrylamide ligand MR116 and ET-JQ1 against the Brd4-BD2(L387A) variant. This suggests that the relative binding affinity is maintained, as well as orthogonality over the Brd4-BD2(WT). We know that the probes do not engage covalently, as indicated by our SDS gel analysis. Therefore, we hypothesized that the increase in signal observed in solution upon incubation with the bump-and-hole mutant can be attributed to reversible binding, which is precluded when incubating with Brd4-BD2(WT). This is captured in the following revised statement:

*"…Additionally, we assessed the fluorogenicity of the probes in solution prior to SDS separation (**Figure 8D, Supplementary Figure 13**). Concentration-dependent increase in fluorescence was observed upon titration against BromoCatch (10 µM), confirming activation upon binding. No fluorogenic activation occurred in buffer alone, where only low level of fluorescence was observed. The fluorescence intensity remained low and comparable to that in buffer when the probe was titrated against Brd4BD2^{WT} but not in the case of Brd4BD2^{L387A} which exhibited a noticeable switch-on effect, albeit weaker than that observed with BromoCatch, due to non-covalent binding…."*

b. "Notably, the fluorescence signal at 10 µM in the positive control was slightly lower than with the cysteine mutant, possibly due to poor solubility or protein binding (**Figure 7D,E**, **Supplementary Figure 10**)." What do the authors refer to here? Plot numbers are incorrect and complicated finding the indicated data/ and the positive control. Please clarify.

This section has been reworded as above.

6) Microscopy Figure 9 and SI Figures

a. Are the scale bars always 100 um? The WT U2OS look much smaller than the H2B- BromoCatch cells. Please double check.

We thank the reviewer for identifying this issue. While the images were scaled appropriately based on the standard settings in Fiji, we agree that some controls appeared to show substantially more numerous and smaller cells. To resolve this issue, we decided to reperform the imaging experiments and removed the previous data from our manuscript. We have now included only the new data, in which this discrepancy is no longer observed.

b. Are the images maximum projections, sum projections? Or just individual z-slices? Please update the Methods section or Figure caption.

We did not acquire full z-stacks for these experiments. Instead, we captured individual z-slice images, selecting the focal plane that exhibited maximal counterstain fluorescence. To enhance the robustness of

our quantification, we also collected three technical replicates per condition in the new microscopy datasets. We chose not to perform maximum-intensity projections, as this method could incorporate signal from planes above and below the nucleus, potentially inflating mean fluorescence intensity (MFI) values and artificially increasing apparent nuclear colocalization. The Methods section has been updated to explicitly state that single z-slices were acquired for all analyses.

c. The authors claim that fluorescence peaks at 8 h and 6 h but no quantification is given. This should be quantified. Please also note: normally such quantifications should not be made on individual z-slices but rather on sum projections of z-stacks or instead, a widefield microscope should be used.

We thank the reviewer for this important point. In the experiments presented, we did not acquire z-stacks; instead, we collected and analyzed single confocal z-slice images. To address the request for quantification, we have now performed unbiased mean fluorescence intensity (MFI) analysis across multiple cells from n = 3 independent experiments. We also acquired three technical replicate images for each condition to improve the robustness of our analysis, and we appreciate the reviewer for prompting this improvement.

As mentioned earlier, we chose not to use sum projections of z-stacks or widefield imaging. Sum projections from various depths in a z-stack could incorporate fluorescent data from regions above and below the nucleus, while widefield imaging aggregates all fluorescent intensities within the cell—both of which could bias our measurements toward artificially brighter nuclei. By analyzing individual Airyscan-acquired z-slices captured at the focal plane of maximal counterstain fluorescence, we ensured that quantification was restricted to a single, well-defined optical plane, thereby avoiding contributions from out-of-focus cytoplasmic signals. This approach provided consistent focal selection while minimizing artificial enhancement of fluorescence intensity.

Importantly, our MFI analysis employed an unbiased workflow, where Hoechst 33342 staining served as a binary selection mask to include both expressing and non-expressing cells. We believe this methodology is sufficient to demonstrate the 'switch-on' behavior of the Janelia Fluor probes and to support our comparative findings: specifically, that C10852L is brighter than C10852S and that both exhibit lower fluorescence compared to the HaloTag JF635 probe. The quantified data have been incorporated into the revised figure and legend.

d. The authors state that fluorescence was strictly confined to the nucleus. However, from the images provided in Figure 9 it is clear that there is substantial fluorescence in areas surrounding the nucleus? Could the authors please quantify this via a correlation coefficient with Hoechst.

We thank the reviewer for this helpful observation. While we did not perform a direct correlation analysis between the fluorescence signal and Hoechst 33342 staining for the microscopy images shown in Figure 9A—due to the lack of a corresponding cytoplasmic marker to delineate outside-nuclear signals without incorporating background—we have quantified Pearson’s and Manders’ correlation coefficients for our orthogonal multiplexing data. These analyses indicate that the BromoCatch-GFP signal shows a moderate-to-high positive correlation with the JF635 fluorescence channel. In comparison, Pearson’s and Manders’ coefficients for Hoechst 33342 versus JF549 show a more moderate correlation, consistent with our observation that while the JF549 signal is primarily nuclear, some fluorescence is present in the cytoplasm.

We interpret the cytoplasmic fluorescence observed in Figures 9A and 9C as resulting from a degree of leakiness due to the high expression of the H2B-BromoCatch and H2B-HaloTag constructs. This leakage leads to a small fraction of H2B-BromoCatch/H2B-HaloTag localizing outside the chromatin region. This interpretation is further supported by the absence of a "switch-on" signal in mock-transfected cells, indicating that the cytoplasmic fluorescence arises specifically from probe activation in the presence of H2B-BromoCatch/H2B-HaloTag, rather than from non-specific labeling.

e. What is the intensity scaling for the different images. Were the same settings used for all images? Please provide a color bar.

We thank the reviewer for this insight. We can confirm that consistent intensity scaling was applied to all images, and a calibration scale has been added to each image to demonstrate this.

f. Fluorogenicity should also be quantified in these experiments. E.g. Nucleus/cytosol ratio or transfected vs. untransformed cells (in no-wash experiments). Please add this quantification.

Mean fluorescence Intensity has been calculated to demonstrate the fluorogenicity of the probes in cellulo (C10852S, C10852L and JF635 haloalkane) – these can be found in **Figure 9B** and values quoted in the main text.

g. SI shows plots for two different proteins (333-460 and 351-460) but this is never explained. Please clarify in the main text what these proteins are.

Both versions of the BromoCatch construct were evaluated in cellular assays. Our initial designs utilized the longer 333-460 fragment to maintain consistency with the original BromoTag design and minimize structural changes during early development. Subsequently, we tested a shorter version (351-460), which performed effectively across multiple targets.

Based on these results, the H2B-BromoCatch construct used in **Figure 9**, **Supplementary Figure 15**, and **Supplementary Figure 17** is based on the shorter sequence. In contrast, the BromoCatch-GFP construct used in **Figure 6**, **Figure 9C**, and **Supplementary Figure 18** utilized the longer variant. Importantly, we observed no differences in performance between the long and short versions.

To streamline nomenclature, we now refer to both constructs simply as BromoCatch, with full sequence details provided in the Supp. Info.

Methods and supplementary information

1. Several graphs in the SI are of suboptimal quality:

o SI 2: extremely small labels in C and D

Font and size of the figure has been updated.

o SI 3: Poor image quality, can you please provide a less pixelated version.

This figure has been updated to be higher resolution.

2. SI numbers are numbered incorrectly in the main text

   o Line 299:“MR116 also exhibits very rapid covalent binding kinetics, as evidenced by complete conversion to the covalent adduct that was observed by intact MS upon as short as 5 min incubation time at room temperature (Supplementary Figure 6).”I am unable to find this data in the SI?

   Apologies, the data was misplaced, and it is now **Supplementary Figure 5**.

3. The microscopy section needs refinement. Please indicate which lasers were used for excitation (for instance none of the provided wavelengths is suitable for Hoechst). What was used for detection. Were images acquired simultaneously sequentially? Single slices, z-stacks?

   Thank you for highlighting this we have updated the methodology to reflect this**.**

Citations

1. The references for SNAP and CLIP tag are mixed up. Reference 16 talks about CLIP tag mostly.

   Addressed

2. Lines 88-91: which references substantiate these claims? Please add suitable references.

   The kinetics of a wide range of SNAP-tag and HaloTag substrate were evaluated by Wilhelm et al. and the reference is used throughout the publication, now added to this sentence to support it.[2]

3. Line 102-106: Introduced the BromoTag degron system: are all these references really required (e.g. 32-37 could be cut to reduce the number of citations in this publication that has already >60 references).

   We removed two of the references.

Phrasing and Clarifications

1) On multiple occasions the authors write about the most potent and stable adduct formed (e.g. line 27, line 44, line 245). What is bond potency? Similarly, the authors have not tested the stability of the adduct formed. Please rephrase.

We have amended this terminology:

Line 27-29: *"We qualify the para-acrylamide bumped derivative MR116 and the Brd4-BD2 double mutant L387A,E438C as the protein-ligand pair of choice"*

Line 44: *"that binds (typically covalently) selectively to the protein tag…"*

Line 244: *"…providing the highest percentage of covalent engagement."*

2) The authors state in the abstract that BromoCatch's kinetic fitness is beneficial. This is not characterized in the current paper – please rephrase or include data about labeling kinetics.

We have now included information on the kinetics and reworded where appropriate. See responses above.

3) Line 74: "Less likely to cause less perturbation": is a double negative. Please adjust.

Reworded to *"less likely to cause perturbation"*

4) Line 92: the authors talk about the TMP-Tag but Figure 1 shows the SpyCatcher/SpyTag which is not mentioned in the introduction. I think the TMP tag should be shown instead or the Spy system introduced in the text.

Thank you for pointing this. The SpyCatcher system has been added now in the text (lines 74 and 94).

5) Line 300: "We hypothesized that MR116's enhanced intracellular activity is due to its covalent binding." What "activity" are the authors refereeing to? Do they refer to the lower IC50? In my eyes this has nothing to do with covalent binding as binding is probably a prerequisite (rate determining step) for the covalent reaction. Please clarify.

We agree that the sentence is confusing. We have reworded the section:

*"…Among the tested ligands, MR116 exhibited optimal intracellular potency, with an IC_{50} of 31 nM, comparable to that of ET-JQ1-OMe (IC_{50} of 88 nM) (**Figure 5F**, **Supplementary Figure 7** and **Supplementary Table 5**). Given the short incubation time, this result suggests rapid cellular uptake of MR116, reflecting good cell permeability of the BromoCatch ligand. …."*

6) Line 306: over 120 min but figure 6B only shows 90 min.

Thank you for pointing this. Now reads 90 min in text.

7) Both SI and main figures are not numbered consecutively and the referencing to the figures in the text is incorrect in many instances.

Apologies for the mistake. This has now been addressed.

8) The Degradation and Fluorophore sections are split: PROTAC, TMR, Glue, JF635. Please structure this differently (PROTAG, Glue, TMR, JF635).

Thank you for your feedback. We have reorganized the data for a more logical flow. We moved the PROTAC section to the end because we successfully performed imaging using the C10852L probe. We believe it is relevant to introduce the probe before discussing its application in the Brd4-BromoCatch CRISPR cell line.

The new order is as follows: biotin (pull-down), TMR (always-on fluorescence), JF635 (fluorogenic imaging), PROTAC (degradation), and glue.

We sincerely thank the reviewers again for the insightful comments and comprehensive feedback on our revised manuscript. Based on the reviewers' suggestions on the newly performed or revised experiments, we have updated the manuscript to address the latest concerns raised by reviewers 1 and 3.

Reviewer #1 (Remarks to the Author):

With this revision, I believe that the overall quality of the manuscript has been substantially improved. In particular, the addition of data addressing this referee's main concern—kinetic analysis and multiplexing with HaloTag system—now enables readers to evaluate the utility of BromoCatch system both quantitatively and qualitatively. I consider the manuscript suitable for publication after the authors address the minor issues outlined below.

We appreciate the reviewer noting the major improvements at first revision, thank you. We also appreciate the additional minor issues raised, and have endeavored to respond to each of them below – including performing key re-analysis of data (MS proteomics) and new experiments (live-cell imaging), as detailed point-by-point below.

1. Supplementary Figure 6:
Panel D lacks a caption. In addition, some of the plots in panel B use identical colors, making them difficult to distinguish. Please use distinct colors and/or plot symbols for all datasets. The units of concentration should also be clearly indicated.

2. Supplementary Figure 11:
Please specify the units of concentration in the plots in panel C.

Response to points 1 and 2: Thank you for the suggestions. The colors and captions have been updated for clarity accordingly in both **Supplementary Figures 6** and **11**.

3. Figure 6C; Supplementary Figures 22, 23, and 25:
Several non-target bands are observed around ~10–15 kDa and ~65 kDa. What do these bands correspond to? Can they be assigned to ENO1, SURF6, or RBM14? If these bands potentially represent unidentified off-targets, this possibility should be explicitly mentioned in the main text or in the figure captions.

Response: Thanks for this comment. We have carefully reviewed and analysed the data in light of these observations.

The bands at ~10-15 KDa were occasionally observed in both the anti-H2B and anti-streptavidin blots. They are only seen in the transfected samples treated with the MR169 probe, but are absent in the samples from untransfected cells, suggesting they are not off-targets of the probe. Their intensities increase with increased concentration of the probe, and follow the same trend of intensity of the H2B-BromoCatch band. Taken together, these observations therefore make us believe these bands are most likely assigned as cellular fragmentation products of the constructs. We have added the following statement in the manuscript main text, lines 380-386: *"We sometimes observed additional bands at 10-15 KDa, in both the anti-H2B and anti-streptavidin blots (**Supplementary Figures 25A and 26D**). These lower molecular weight bands are absent in the samples from untransfected cells treated with the MR169 probe, suggesting they are not off-targets of the probe, and are likely fragmentation products of the constructs."*

With respect to the other band observed at ~65 kDa, this behaves distincivelyh. It is only observed in the anti-streptavidin blot, in both untransfected and transfected cells, only when treated with compound MR169 (**Supplementary Figure 26D**). This suggests that this band may correspond to a protein that gets modified by the MR169 probe. We have added the following statement int he manuscript main text, lines 384-386: *"Another band was observed at ~65 kDa in the anti-streptavidin blot in both untransfected and transfected cells, only when treated with compound, suggesting this could be an off-target of MR169 (**Supplementary Figure 26D**)."*

4. The authors conclude that ENO1, SURF6, and RBM14 are not off-targets because they are not detected in untransfected cell samples. However, this conclusion may appear to result from the use of an overly stringent p-value threshold (commonly p-value < 0.05, corresponding to −log10(p-value) ≈ 1.3, is used in proteomics research). In fact, several proteins in the untransfected cell samples meet the criteria of log2(FC) > 2 and p < 0.05, indicating that off-target labeling is objectively present. Given that background labeling is expected when acrylamide is used as a warhead, I do not think it is necessary to strongly argue for the absence of off-targets. Instead, it would be more objective and scientifically appropriate to acknowledge the presence of potential off-targets and discuss that, nevertheless, the system remains suitable for imaging applications in overexpression settings. At a minimum, the current statements claiming the absence of off-targets should be toned down and rephrased accordingly.

Response: Thanks for raising this point and for the constructive critique. We concur, and we have now re-analysed the MS proteomics pull-down data using less stringent p-value threshold, and applied a statistical cut-off of -log10 (p-value) of -1.5. This is while keeping the cut-offs of log2 (fold change) of 2 (corresponding to a 4-fold increase/decrease in quantitation. As a result of this less stringent cut-off, we now detect 54 proteins in addition to BromoCatch-GFP (**Figure 5G**), and 14 proteins from the untransfected cells (**Figure 5F**).

We found no overlap between these set of hits, consistent with absence of off-targets of the probe.

We agree with the reviewer that background labeling is expected in these experiments and indeed 11/13 hits from the untransfected cells are known common contaminants in MS pull downs as listed in the in the CRAPome database (https://reprint-apms.org/). All of the additional 54 hits pulled down by the probe were displaced (53/54 down to non-significant detection) upon pre-incubation with the competing ligand – suggesting specific interaction with the construct. However, extensive analysis of protein-protein interaction database exclude any of these proteins are specific interactors of Brd4 (e.g. via the BD2 domain which is the sequence BromoCatch is derived from). Therefore, we believe these are likely to be artefactual interactors of the over-expressed fusion protein constructs.

We have now re-drafted the section describing the MS-proteomics pull-down experiment in the manuscript main text, lines 406-426: *"To ensure confidence in the identified hits, during data analysis we used stringent cut-offs of log2 (fold change) of 2 (corresponding to a 4-fold increase/decrease in quantitation) and a statistical cut-off of -log10 (p-value) of -1.5 (**Figure 6G-H**, and **Supplementary Table 17**). We observed significant enrichment of BromoCatch-GFP protein in transfected cells treated with MR169 (**Figure 6G**) compared to untransfected cells (**Figure 6F**), consistent with western blot analysis (**Supplementary Figure 12**). Importantly, pretreatment with the competing ligand Et-JQ1-OMe at high concentration (25 μM) substantially diminished pulldown of BromoCatchGFP, confirming the probe's specificity for BromoCatch (**Figure 6H**). Interestingly, in addition to BromoCatch-GFP we see enrichment of another 54 proteins (**Figure 6G** and **Supplementary Table 17**), none of which were detected in the untrasfected cells, and which were all displaced with preincubation of the competing ligand (**Figures 6F, H** and **Supplementary Table 17**). These proteins could be recruited to BromoCatch-GFP, as interactors of the fusion protein, likely an artefact of the construct over-expression. The most enriched amongst these hits were ENO1, SURF6 and RBM14. Interaction database searches, e.g. BioGrid5.0, showed no evidence for specific interactions with BRD4, beyond minor associations,[58,59] suggesting these proteins are also artefactual background interactors. We also detected 13 proteins in the untrasfected control cells (**Figure 6F**). None of these proteins were pulled-down in the transfected cells (**Figure 6G-H**), and most of them appear as known frequent contaminants in streptavidin-based pulldowns,[60] suggesting minor background labeling. Together, the data shows that BromoCatch is efficiently and highly-selectively labelled by our biotin probe MR169 and pulled-down MS-proteomics applications qualified its clean profile suitable for imaging applications in overexpression settings.*

5. Page 16, line 486:
"JF63" should be corrected to "JF635".
This has now been updated.

6. Figure 9:

To more clearly demonstrate the orthogonality between BromoCatch and HaloTag, the following control experiments should be included:

(i) treatment of cells expressing H2B–HaloTag alone with C10852L;
(ii) treatment of cells expressing H2B–BromoCatch alone with a Haloalkane probe.

Response: We thank the reviewer for this helpful suggestion. In response, we have performed new live-cell imaging experiments including both requested controls: (i) treatment of H2B–HaloTag-expressing cells with C10852L, and (ii) treatment of H2B–BromoCatch/ GFP-Bromocatch-expressing cells with the Haloalkane probe. These data are now included in the revised **Supplementary Figure 21**. In both cases, no detectable nuclear labeling above background was observed, demonstrating that neither probe cross-reacts with the non-cognate tag. Together, these results confirm that BromoCatch and HaloTag operate as orthogonal and compatible systems in live cells.

Reviewer #2 (Remarks to the Author):

The authors have satisfactorily addressed the reviewers' comments, and the revised manuscript represents a significant improvement.

We appreciate the comment, thank you.

Reviewer #3 (Remarks to the Author):

Re-review

The authors have provided further data (kinetic characterization, repeated labeling experiments in cells) benchmarking their system better against HaloTag. They also performed one co-imaging experiment with HaloTag. However, BromoCatch has no clear advantage over the SNAP and HaloTag. It would therefore be crucial to showcase a triple multiplexing experiment with HaloTag and SNAP-tag (commercially available from NEB) as previously suggested.

We appreciate acknowledgement of the new data and thank you for the thorough re-review. Our goal with developing BromoCatch was to provide a novel, additional Self-labeling tag with utility for cellular protein labeling. We were not strictly aiming to do better than HaloTag or SNAP-tag, which are widely used and work. Rather, we wanted to offer a valuable addition to the arsenal of SLP tools, to also enable simultaneous orthogonality – as "the more the merrier". In doing so, we were keen to explore potential advantages of our new system e.g. small tag size, novel specific chemistry. We believe that our extensive characterisation in vitro and in cell qualifies BromoCatch as such valuable addition to the field.

We respect the reviewer's view and agree that showcasing a triple multiplexing experiment with HaloTag and SNAP-tag would be an interesting comparison and is aspirational. Here, we focused on demonstrating multiplexing and orthogonality between BromoCatch and HaloTag as a representative second tag. We believe this data is appropriate and adequate to benchmark BromoCatch and establish its utility and orthogonality for multiplexing. **While we agree that inclusion of a triple multiplexing experiment would be nice to have, it represents a substantial experimental effort within the timeframe of this revision, that we feel is beyond the scope of the current study. As advised by the Editors, we have therefore proceeded with a revision that does not include such experiments, that will be warranted as the focus of future studies.**

Specific comments:

1) Labeling kinetics

Comment: The authors now provide apparent rate constants for M116 and MR169 (non-fluorescent) and MR202 (fluorescent) and they are between 10^4 and 10^5 M-1 s-1. The authors state that this is within the same range as HaloTag and SNAP-tag. However, with most fluorophores HaloTag has an apparent rate constant of > 10^6. These are 2 orders or even 3 orders of magnitude higher. This needs to be clarified. The labeling kinetics of MR202 are in the order of SNAP-tag but not HaloTag. Please adjust the manuscript accordingly.

Response: we appreciate the reviewer raising this valid point. The following sections have been updated and now read:

P12 lines 310-313: "*In the context of established self-labeling tags, the BromoCatch ligand exhibits labeling kinetics rates in the range of* 10^5 M^{-1} s^{-1}*, comparable to SNAP-tag probes such as BG-TMR, while HaloTag7 can reach ~*10^7 M^{-1} s^{-1} *with optimized probes.*[26]"

P14 lines 399-400 "*The rate of labeling of this non-fluorescent biotin probe is consistent with the labelling rate reported for non-fluorescent probes for SNAP-tag and HaloTag (10^4 – 10^5 $M^{-1}s^{-1}$).*[26]"

p16 lines 463-467: *"Consistent with observations for the MR169 probe, the BromoCatch system displayed similar labeling kinetics for the TMR-based MR202 probe. This contrasts with the SNAP-tag and HaloTag systems where rhodamine-based fluorophores markedly enhance the labeling kinetics, while no fluorophore-induced kinetic improvement was observed for BromoCatch."*

2) Cell permeability

Comment: Line 532-434: The experiments performed to look at cell permeability (Supplementary Figure 17) should be discussed better in the main manuscript, currently they are only mentioned in passing.

Response: The following section has been added to the main text in the MR202 section (p15, lines 441-447):

*"The labelling efficiency of MR202 in the western blot was compared to that for H2B-HaloTag transfected HEK293-FT cells using the haloalkane JF635 probe under similar conditions. We observed that the HaloTag construct was labelled more efficiently at lower concentrations of the corresponding JF635 probe, which could suggest better permeability of the haloalkane probe (**Supplementary Figure 13**). Ongoing work is focused on systematically evaluating the permeability of BromoCatch probes to optimize intracellular labelling efficiency."*

3) Fluorogenicity

3.1 Comment: Line 491 and Figure 8F/G: The authors performed an in vitro fluorogenicity comparison and compared the fold changes in terms of emission. I cannot find the emission data for HaloTag. Figure G only provides excitation spectra. Please add this data.

Response: This was our oversight—good catch! The emission data for the HaloTag probe have now been added to the main figure. We have also updated the text to more clearly emphasize the relative fold improvements observed between probes, see updated text lines 510-515: *"C10852L gave threefold improvement over C10852S in signal-to-noise ratio, calculated to be 20- and 7-fold for C10852L and C10852S, respectively (Figure 8F). Indicating that the shorter linked C10852L probe is superior. Notably, the JF635 haloalkane probe achieved the greatest signal-to-noise ratio (100-fold, Figure 8G), attributable to its*

minimal background fluorescence in PBS, corresponding to an overall approximate fivefold improvement over C10852L."

In summary, C10852L shows an approximately 3-fold increase in fluorogenicity compared to C10852S, and in turn, the haloalkane 635 probe is ~5 fold more fluorogenic than C10852L itself.

During the source data file generation, we noted that the calculated fold changes were from a related experimental dataset. The values have now been updated using the data from **Figure 8F/G**, resulting in minor numerical adjustments that do not affect the reported trend or conclusions. The updated source data file includes the supporting datasets for **Figure 8F** and **Figure 8G**, as well as an additional independent repeat demonstrating the same trend.

3.2 Previous comment (from first review): Fluorogenicity should also be quantified in these experiments. E.g. Nucleus/cytosol ratio or transfected vs. untransformed cells (in no-wash experiments). Please add this quantification) Similarly, they did not perform correlation experiments to quantify the mislocalized signal outside of the nucleus.

Our previous response (from first revision): Mean fluorescence Intensity has been calculated to demonstrate the fluorogenicity of the probes in cellulo (C10852S, C10852L and JF635 haloalkane) – these can be found in Figure 9B and values quoted in the main text.

Response from reviewer (from second review): For fluorogenicity comparisons unwashed cells expressing BormoCatch and unwashed cells not-expressing BromoCatch but both treated with C10852L should be compared and not dye with vehicle control. Please provide the corresponding comparison (data is available just the analysis is missing).

Response: We thank the reviewer for this important clarification. In response, we first repeated the live-cell confocal imaging experiments incorporating the reviewer's previous technical suggestions to minimise potential chromatic aberration. We then re-performed quantitative analysis using summed Z-stack projections and directly compared transfected, probe-treated cells (C10852L, C10852S, and JF635 Haloalkane) with their corresponding non-transfected, probe-treated controls, as requested. The corresponding data and quantitative analyses have now been incorporated into the revised manuscript, supplementary material and updated figure panels (**Figure 9B, Supplementary Table 8**).

3.3 Previous comment (from first review): The authors state that fluorescence was strictly confined to the nucleus. However, from the images provided in Figure 9 it is clear that there is substantial fluorescence in areas surrounding the nucleus? Could the authors please quantify this via a correlation coefficient with Hoechst.)

Our previous response (from first revision): We thank the reviewer for this helpful observation. While we did not perform a direct correlation analysis between the fluorescence signal and Hoechst 33342 staining for the microscopy images shown in Figure 9A—due to the lack of a corresponding cytoplasmic marker to delineate outside-nuclear signals without incorporating background—we have quantified Pearson's and Manders' correlation coefficients for our orthogonal multiplexing data. These analyses indicate that the BromoCatch-GFP signal shows a moderate-to-high positive correlation with the JF635 fluorescence channel. In comparison, Pearson's and Manders' coefficients for Hoechst 33342 versus JF549 show a more moderate correlation, consistent with our observation that while the JF549 signal is primarily nuclear, some fluorescence is present in the cytoplasm.

We interpret the cytoplasmic fluorescence observed in Figures 9A and 9C as resulting from a degree of leakiness due to the high expression of the H2B-BromoCatch and H2B-HaloTag constructs. This leakage leads to a small fraction of H2B-BromoCatch/H2B-HaloTag localizing outside the chromatin region. This interpretation is further supported by the absence of a "switch-on" signal in mock-transfected cells, indicating that the cytoplasmic fluorescence arises specifically from probe activation in the presence of H2B-BromoCatch/H2B-HaloTag, rather than from non-specific labeling.

Response from reviewer (from second review): A correlation analysis does not require a cytoplasmic marker. The signal from the C10852L channel can simply be correlated to the Hoechst channel on the images provided in Figure 9A. Also the quantification provided on images in 9C are not helpful as BromoCatch is not targeted.

Response: We thank the reviewer for raising this important point and for clarifying that correlation analysis can be performed directly between the C10852L and Hoechst channels.

To further assess signal distribution and potential mislocalisation, we performed the following analyses:

(i) direct colocalization analysis between probe signal and Hoechst.

(ii)nuclear versus cytoplasmic intensity analysis using Hoechst-defined nuclear masks.

Using our newly acquired imaging dataset (**Figure 9A**), we performed correlation analysis exactly as suggested by correlating the C10852L (and corresponding probe) channel directly with the Hoechst channel, without the need for an additional cytoplasmic marker, and

instead measured. Pearson's correlation coefficients were calculated on central z-slices of Z-stacks using identical imaging conditions. Correlation analysis revealed minimal Hoechst–probe correlation in non-transfected controls, demonstrating that probe "switch-on" and nuclear colocalization are dependent on the presence of BromoCatch. Only marginal correlation was observed in the competitive control, likely due to incomplete competition (**Supplementary Figure 18**).

Nuclear–cytoplasmic analysis demonstrated strong nuclear enrichment for all probes, with C10852S showing the highest background (~16% cytoplasmic/background fluorescence) with C10852L and HaloTag JF635 Haloalkane having smaller cytoplasmic signal (**Supplementary Figure 19**).

In line with the reviewer's comment, we have also removed the quantification previously shown in **Figure 9C**, as BromoCatch is not targeted in that context.

The revised manuscript now includes the updated correlation and fluorogenicity analyses in **Figure 9** in both the main text (page 21-23) and the supplementary (**Supplementary Table 9** and **Supplementary Figure 21-22**).

4) Multiplexing:

Comment: The authors show multiplexing and orthogonality with HaloTag but not SNAP-tag.

Response: We acknowledge this point and agree that this would also be an interesting comparison. In the present study, we focused on demonstrating multiplexing and orthogonality between BromoCatch and HaloTag as a representative second tag, as a widely used self-labelling tag system. HaloTag was selected due to its rapid kinetics, well-characterised ligands, and extensive applications in multicolor live-cell imaging, making it a stringent reference for testing compatibility. We believe this data is appropriate and adequate to benchmark BromoCatch and establish its utility and orthogonality for multiplexing. We therefore feel that inclusion of a triple multiplexing experiment is beyond the scope of the current study.

While SNAP-tag orthogonality was not experimentally evaluated here, we concur that extension of multiplexing with SNAP-tag is interesting and certainly warranted for future studies. BromoCatch and SNAP-tag rely on fundamentally distinct chemistries and ligand scaffolds, so cross-reactivity is not anticipated. To anticipate this future work, we have clarified that *orthogonality has been experimentally demonstrated with HaloTag in this work, but compatibility with other self-labelling systems, including SNAP-tag, remains to be*

formally assessed in future studies. This clarification has been incorporated into the revised manuscript to appropriately define the scope of our orthogonality conclusions.

5) Fluorescence microscopy in general

Comment: Fluorescence intensity measurements like these are best performed on widefield images or summed z-stacks. By choosing a single z-slice the experimentator introduces artifacts by choosing the plane. The focal plane of the far-red and Hoechst channel are not the same due to chromatic aberration.

Response: We thank the reviewer for this important technical point. We have now repeated the imaging experiments capturing Z-stacks and performed quantitative analyses where possible with summed Z-stack projections rather than single optical sections. All fluorescence intensity measurements (MFI), and nuclear–cytoplasmic analysis are now performed on summed Z-stacks to ensure full nuclear sampling and to avoid bias introduced by selection of a single focal plane. The revised manuscript (pages 20-22, **Figure 9A** and **B**), and the supplementary information and methods have been updated accordingly to reflect these changes.

Minor points

1) What is the intensity scaling for the different images. Were the same settings used for all images? Please provide a color bar.

Comment: The provided color bars are extremely small and impossible to read.

Response: Thank you for this observation. We agree that the color bars in the original submission were too small to be easily legible. In the revised manuscript, we have increased the size and font of all color bars to ensure they are clearly readable at standard viewing and print magnifications and have moved them to the righthand side of imaging panels to not affect the visible space in the imaging figures. We have also verified that the scaling remains consistent within each channel across all relevant panels in all experiments (**Figure 9A, C** and **Supplementary Figures 17, 20 & 21**).

2) The microscopy section needs refinement. Please indicate which lasers were used for excitation (for instance, none of the provided wavelengths is suitable for Hoechst).

What was used for detection. Were images acquired simultaneously sequentially? Single slices, z-stacks?

Comment: The excitation wavelengths and information on simultaneous and sequential detection is still missing. Information for GFP and TMR imaging are missing.

Response: We thank the reviewer for highlighting this. We have now revised the microscopy methods section to include all complete details on excitation, emission, acquisition details and sequential detection parameters for all experiments performed on the confocal microscope.

Thank you to reviewers 1 and 3 for the positive feedback after the last revision and for the constructive suggestions through the revision process, the manuscript has greatly benefited from their input.

Reviewer #3 (Remarks to the Author):

I thank the authors for clarifying all my questions. The manuscript is now suitable for publication (see one minor addition below).

Multiplexing: I understand that the experimental effort is too high to provide a triple multiplexing experiment. I would however like to encourage the authors to perform such experiments in a follow-up study as BromoCatch could indeed expand the family of SLP tools. Currently, only SNAP and HaloTag are used routinely and covalent TMP-tag and CLIP have not found widespread use. It might be possible that BromoCatch can indeed fill this niche.

We thank the reviewer for the suggestion. We agree that this would be a very interesting comparative study to expand the scope of our system, that is warranted in future work.

Colocalization Analysis: We thank the authors a lot for including colocalization analysis as well as the analysis of cytoplasmic/nuclear ratios. These are common measurements performed in the imaging community and will greatly strengthen the impact of the paper. With regard to the co-localization analysis, I would like to highlight one aspect. Given the authors conducted this analysis on transiently transfected cells the resulting R values are rather low (0.2/0.3). This mostly reflects the fact that all cells show Hoechst signal but only the Halo expressing cells show far-red signal. Hence R greatly underestimates the "true" colocalization. Ideally the analysis would exclude the non-transfected cells. However, as the authors compare R values among differently stained conditions but similarly transfected cells, this is ok. To avoid any confusion by readers, I would include a statement mentioning that non-transfected cells were not excluded for analysis.

We thank the reviewer for highlighting this point. We have now included the following statement in the Results section when discussing colocalization: *"We observed modest Pearson's r values (≈0.2–0.3), which we attribute to our whole-field quantification that includes the non-transfected cells to avoid selection bias. Nevertheless, because this approach was applied consistently across all conditions, the comparisons remain valid."*

Revisions “BromoCatch: a self-labelling tag platform for protein analysis and live cell imaging”

Overall comments

Rodriguez-Rios et al. report on the generation of a new self-labeling protein tag system derived from Brd4-BD2 called BromoCatch. Supported by computational docking experiments they generate protein variants carrying reactive cysteines as well as a panel of JQ1 derived molecules functionalized with electrophilic warheads. They synthesize and test a subset of 14 small molecules for their reactivity towards two protein variants using ESI-MS, DSF, and NanoBRET assays. They finally characterize one ligand in depth via competition assays as well as an x-ray crystal structure. They then further functionalize this ligand with different handles including alkynes (click-chemistry), VHL (E3 recruiter), fluorophores (TMR, JF635) and characterize the functionality of these molecules primarily via in vitro protein labeling, in gel fluorescence and western blots. They finally demonstrate the use of the JF635 analogue in confocal microscopy experiments of H2B fused BromoCatch.

The authors follow a well-conceived rational route to generate the BromoCatch system, which is an attractive new addition to the SLP family due to its small size (13kDa vs. 18 kDa eDHFR, 19.4 kDa SNAP-tag, 33 kDa HaloTag). The molecules are well characterized in vitro and the paper well written. The data is nicely presented, and the in vitro experiments were very rigorously performed. However, some of the western-blot data (see point 2 below) and microscopy data require improvement, need to be more thoroughly described and/or are not sufficiently analyzed complicating the interpretation of the data. The methods provided are extensive but require more detail in the microscopy section.

Major concerns

My major concern with the presented paper is that their system is not benchmarked in any way to other self-labeling protein tags. The authors nicely outline the shortcomings of other tags in their introduction: size (Halo), slow labeling kinetics (SNAP), fluorogenicity (SNAP), and cell permeability of the ligand (SNAP). They further state that they “aimed at addressing the limitation of current SLPs to expand the toolbox with an orthogonal platform for multiplexing”. However, neither the orthogonality nor the overcoming of the limitations is clearly demonstrated:

- What are the labeling kinetics of their system (in vitro, point 1 below).
- How does cell permeability of their molecules compare to the one of SNAP-tag substrates? E.g. labeling in cells at identical concentrations with similar protein fusions (see point 1 below).
- How does fluorogenicity of this tag compare to SNAP-tag and HaloTag (from a first glance of Figure 8E: 4x not very favorably, point 4 and 6.
- Does the smaller size allow to tag any of the proteins mentioned in the introduction (AGO2, COX8A, ATP5ME)?
- Lastly, can the authors showcase a co-imaging experiment with one, two or even three of the other tags (HaloTag, SNAP-tag, eDHFR)?

If the authors could provide the above-mentioned data and thereby clearly benchmark BromoCatch over existing tags or show its suitability in advanced multiplexing experiments, the manuscript would greatly gain in impact.

Detailed comments on the manuscript's content

Results and further experiments

1) An important parameter of SLP's is their labeling kinetics e.g. see reference 18. The authors state that kinetics are fast (e.g. line 297) but no experiments were performed for precise quantification. To be able to compare the performance of the BromoCatch with other systems it would be imperial to measure the labeling kinetics in vitro and maybe in cellulo. This should be added.
2) Blots and Gels
    a. CuAAC experiments SI Fig 6C: Why is there a huge Cy5 streak in both the WT and L387A samples? Please clarify.
    b. Biotin Blot MR169, Figure 7C. The blots are labeled inadequately. It is difficult to understand which antibodies and fluorophores were used. Please adapt the SI Figure and/or the Methods section. Also, the cells used are not indicated in the SI Figure. What are all the additional bands that are labeled in the "presumably" anti-strep blot (labeled H2B-Bromo-Catch)? Please clarify in the text.
    c. Line 368 (Fig 7F): "exquisite selectivity" I think this statement is misleading as any non-covalently bound dye will be "washed" out on the protein gel. I don't think that this experiment allows any conclusions on selectivity but only shows which proteins form a covalent bond and which not. Please adapt the text.
    d. Similarly (line 421): "SDS gel analysis showed maximal fluorescence at stoichiometric protein-to probe levels (10 µM), after which the signal plateaued despite increasing C10852S concentration." Again, residual probe that is not bound to protein will just be washed out and hence this is not surprising. Please revise the text.
    e. Figure 7F and SI Figure: Tubulin and MR202 were visualized with a spectrally identical fluorophore TMR and Alexa546. To tell which one is which, tubulin should be visualized separately. Please provide such an experiment.
3) Can the authors please speculate why MR170 does not lead to >90% degradation? Please add this to the PROTAC section.
4) The fluorogenicity of the generated system should be characterized further and be quantified by fluorescence excitation and emission (e.g. Figure 8E). Similarly, to show advantages over other SLP's this should be characterized in cells (see below). Please provide these additional measurements.
5) Figure 8D: I cannot find any information on the experimental details of Figure 8D. Please add this information to the methods section. Importantly, which protein concentration was used?
    a. Furthermore, the authors state: "However, fluorescence levels were lower compared to the covalent system, suggesting covalency enhances fluorogenicity" As the probe likely has different affinity for the two proteins this statement is not correct in my eyes. Please revise or clarify how you come to this conclusion.
    b. "Notably, the fluorescence signal at 10 µM in the positive control was slightly lower than with the cysteine mutant, possibly due to poor solubility or protein binding (**Figure 7D,E**, **Supplementary Figure 10**)." What do the authors refer to here? Plot

numbers are incorrect and complicated finding the indicated data/ and the positive control. Please clarify.

6) Microscopy Figure 9 and SI Figures
   a. Are the scale bars always 100 um? The WT U-2 OS look much smaller than the H2B-BromoCatch cells. Please double check.
   b. Are the images maximum projections, sum projections? Or just individual z-slices? Please update the Methods section or Figure caption.
   c. The authors claim that fluorescence peaks at 8 h and 6 h but no quantification is given. This should be quantified. Please also note: normally such quantifications should not be made on individual z-slices but rather on sum projections of z-stacks or instead, a widefield microscope should be used.
   d. The authors state that fluorescence was strictly confined to the nucleus. However, from the images provided in Figure 9 it is clear that there is substantial fluorescence in areas surrounding the nucleus? Could the authors please quantify this via a correlation coefficient with Hoechst.
   e. What is the intensity scaling for the different images. Were the same settings used for all images? Please provide a color bar.
   f. Fluorogenicity should also be quantified in these experiments. E.g. Nucleus/cytosol ratio or transfected vs. untransformed cells (in no-wash experiments). Please add this quantification.
   g. SI shows plots for two different proteins (333-460 and 351-460) but this is never explained. Please clarify in the main text what these proteins are.

Methods and supplementary information

1) Several graphs in the SI are of suboptimal quality:
   - SI 2: extremely small labels in C and D
   - SI 3: Poor image quality, can you please provide a less pixelated version.
2) SI numbers are numbered incorrectly in the main text
   - Line299: “MR116 also exhibits very rapid covalent binding kinetics, as evidenced by complete conversion to the covalent adduct that was observed by intact MS upon as short as 5 min incubation time at room temperature (**Supplementary Figure 6**).”
     I am unable to find this data in the SI?
3) The microscopy section needs refinement. Please indicate which lasers were used for excitation (for instance none of the provided wavelengths is suitable for Hoechst). What was used for detection. Were images acquired simultaneously sequentially? Single slices, z-stacks?

Citations

1) The references for SNAP and CLIP tag are mixed up. Reference 16 talks about CLIP tag mostly.
2) Lines 88-91: which references substantiate these claims? Please add suitable references.
3) Line 102-106: Introduced the BromoTag degron system: are all these references really required (e.g. 32-37 could be cut to reduce the number of citations in this publication that has already >60 references).

Phrasing and Clarifications

1) On multiple occasions the authors write about the most potent and stable adduct formed (e.g. line 27, line 44, line 245). What is bond potency? Similarly, the authors have not tested the stability of the adduct formed. Please rephrase.
2) The authors state in the abstract that BromoCatch’s kinetic fitness is beneficial. This is not characterized in the current paper – please rephrase or include data about labeling kinetics.
3) Line 74: “Less likely to cause less perturbation”: is a double negative. Please adjust.
4) Line 92: the authors talk about the TMP-Tag but Figure 1 shows the SpyCatcher/SpyTag which is not mentioned in the introduction. I think the TMP tag should be shown instead or the Spy system introduced in the text.
5) Line 300: “We hypothesized that MR116's enhanced intracellular activity is due to its covalent binding.” What “activity” are the authors refereeing to? Do they refer to the lower IC50? In my eyes this has nothing to do with covalent binding as binding is probably a prerequisite (rate determining step) for the covalent reaction. Please clarify.
6) Line 306: over 120 min but figure 6B only shows 90 min.
7) Both SI and main figures are not numbered consecutively and the referencing to the figures in the text is incorrect in many instances.
8) The Degradation and Fluorophore sections are split: PROTAC, TMR, Glue, JF635. Please structure this differently (PROTAG, Glue, TMR, JF635).

Re-review

The authors have provided further data (kinetic characterization, repeated labeling experiments in cells) benchmarking their system better against HaloTag. They also performed one co-imaging experiment with HaloTag. However, BromoCatch has no clear advantage over the SNAP and HaloTag. It would therefore be crucial to showcase a triple multiplexing experiment with HaloTag and SNAP-tag (commercially available from NEB) as previously suggested.

Specific comments:

1) Labeling kinetics

   Comment: The authors now provide apparent rate constants for M116 and MR169 (non-fluorescent) and MR202 (fluorescent) and they are between 10^4 and 10^5 M-1 s-1. The authors state that this is within the same range as HaloTag and SNAP-tag. However, with most fluorophores HaloTag has an apparent rate constant of > 10^6. These are 2 orders or even 3 orders of magnitude higher. This needs to be clarified. The labeling kinetics of MR202 are in the order of SNAP-tag but not HaloTag. Please adjust the manuscript accordingly.

2) Cell permeability

   Comment: Line 532-434: The experiments performed to look at cell permeability (Supplementary Figure 17) should be discussed better in the main manuscript, currently they are only mentioned in passing.

3) Fluorogenicity

   - **Comment:** Line 491 and Figure 8F/G: The authors performed an in vitro fluorogenicity comparison and compared the fold changes in terms of emission. I cannot find the emission data for HaloTag. Figure G only provides excitation spectra. Please add this data.

   - Previous comment: Fluorogenicity should also be quantified in these experiments. E.g. Nucleus/cytosol ratio or transfected vs. untransformed cells (in no-wash experiments). Please add this quantification.) Similarly, they did not perform correlation experiments to quantify the mislocalized signal outside of the nucleus.

     Mean fluorescence Intensity has been calculated to demonstrate the fluorogenicity of the probes in cellulo (C10852S, C10852L and JF635 haloalkane) – these can be found in **Figure 9B** and values quoted in the main text.

     Response: For fluorogenicity comparisons unwashed cells expressing BormoCatch and unwashed cells not-expressing BromoCatch but both treated with C10852L should be compared and not dye with vehicle control. Please provide the corresponding comparison (data is available just the analysis is missing).

   - Previous comment: The authors state that fluorescence was strictly confined to the nucleus. However, from the images provided in Figure 9 it is clear that there is substantial fluorescence in areas surrounding the nucleus? Could the authors please quantify this via a correlation coefficient with Hoechst.)

We thank the reviewer for this helpful observation. While we did not perform a direct correlation analysis between the fluorescence signal and Hoechst 33342 staining for the microscopy images shown in Figure 9A—due to the lack of a corresponding cytoplasmic marker to delineate outside-nuclear signals without incorporating background—we have quantified Pearson's and Manders' correlation coefficients for our orthogonal multiplexing data. These analyses indicate that the BromoCatch-GFP signal shows a moderate-to-high positive correlation with the JF635 fluorescence channel. In comparison, Pearson's and Manders' coefficients for Hoechst 33342 versus JF549 show a more moderate correlation, consistent with our observation that while the JF549 signal is primarily nuclear, some fluorescence is present in the cytoplasm.

We interpret the cytoplasmic fluorescence observed in Figures 9A and 9C as resulting from a degree of leakiness due to the high expression of the H2B-BromoCatch and H2B-HaloTag constructs. This leakage leads to a small fraction of H2B-BromoCatch/H2B-HaloTag localizing outside the chromatin region. This interpretation is further supported by the absence of a "switch-on" signal in mock-transfected cells, indicating that the cytoplasmic fluorescence arises specifically from probe activation in the presence of H2B-BromoCatch/H2B-HaloTag, rather than from non-specific labeling.

Response: A correlation analysis does not require a cytoplasmic marker. The signal from the C10852L channel can simply be correlated to the Hoechst channel on the images provided in Figure 9A. Also the quantification provided on images in 9C are not helpful as BromoCatch is not targeted.

4) Multiplexing:

   Comment: The authors show multiplexing and orthogonality with HaloTag but not SNAP-tag.

5) Fluorescence microscopy in general

   Comment: Fluorescence intensity measurements like these are best performed on widefield images or summed z-stacks. By choosing a single z-slice the experimentator introduces artifacts by choosing the plane. The focal plane of the far-red and Hoechst channel are not the same due to chromatic aberration.

Minor things

1) What is the intensity scaling for the different images. Were the same settings used for all images? Please provide a color bar.

   Comment: The provided color bars are extremely small and impossible to read.

2) The microscopy section needs refinement. Please indicate which lasers were used for excitation (for instance none of the provided wavelengths is suitable for Hoechst). What was used for detection. Were images acquired simultaneously sequentially? Single slices, z-stacks?

   Comment: The excitation wavelengths and information on simultaneous and sequential detection is still missing. Information for GFP and TMR imaging are missing.
